# The medicinal plants of Myanmar

**DOI:** 10.3897/phytokeys.102.24380

**Published:** 2018-06-28

**Authors:** Robert A. DeFilipps, Gary A. Krupnick

**Affiliations:** 1 Deceased; 2 Department of Botany, National Museum of Natural History, Smithsonian Institution, PO Box 37012, MRC-166, Washington, DC, 20013-7012, USA

**Keywords:** Myanmar, medicinal plants, traditional knowledge, ethnobotany, checklist, conservation

## Abstract

A comprehensive compilation is provided of the medicinal plants of the Southeast Asian country of Myanmar (formerly Burma). This contribution, containing 123 families, 367 genera, and 472 species, was compiled from earlier treatments, monographs, books, and pamphlets, with some medicinal uses and preparations translated from Burmese to English. The entry for each species includes the Latin binomial, author(s), common Myanmar and English names, range, medicinal uses and preparations, and additional notes. Of the 472 species, 63 or 13% of them have been assessed for conservation status and are listed in the IUCN Red List of Threatened Species (IUCN 2017). Two species are listed as Extinct in the Wild, four as Threatened (two Endangered, two Vulnerable), two as Near Threatened, 48 Least Concerned, and seven Data Deficient. Botanic gardens worldwide hold 444 species (94%) within their living collections, while 28 species (6%) are not found any botanic garden. Preserving the traditional knowledge of Myanmar healers contributes to Target 13 of the Global Strategy for Plant Conservation.

## Introduction

In many parts of the world traditional knowledge and biodiversity still play an import role in health care, culture, religion, food security, environment, and sustainable development. Moreover, many widely used plant-based medicines are derived from traditional knowledge. Preserving, protecting, and promoting (if scientifically supported) traditional knowledge is of key importance. The Global Strategy for Plant Conservation (GSPC) calls for the sustainable and equitable use of plant diversity (CBD 2002). GSPC’s Target 13 aims for an increase in indigenous and local knowledge innovations and practices associated with plant resources to support customary use, sustainable livelihoods, local food security, and health care. It is with this aim that we compiled a list of plant species and their medicinal uses in Myanmar based on published accounts. The information contained in this compilation comes from popular knowledge and was not scientifically tested in terms of the efficacy of the uses of the plants listed.

### History of published accounts of Myanmar medicinal plants

Some of the earliest literature concerning the medicinal plants of Myanmar includes:

• Mason F (1850) The Natural Productions of Burma; or, Notes on the Fauna, Flora, and Minerals of the Tenasserim Provinces and the Burman empire. Moulmain.

• Lace JH, Roger A (1922) List of Trees, Shrubs, and Principal Climbers, etc., recorded from Burma. Rangoon.

• Rodger A (1951) A Handbook of the Forest Products of Burma. Rangoon.

• Report of the Committee of Enquiry into the Indigenous System of Medicine (1951) Rangoon.

• Sawyer AM, Daw Nyun (1955) Classified List of the Plants of Burma. Rangoon.

• Hundley HG, U Chit Ko Ko (1961) Trees, Shrubs, Herbs and Principle Climbers, etc. Rangoon.

In 1948, when the Union of Burma first gained its independence from the United Kingdom, the first Burmese government began to build a pharmaceutical factory, the Burma Pharmaceutical Industry (B.P.I.). B.P.I. was “large enough to cover the production of all essential medicines” for the population. This factory officially opened in 1958. Initially they had to depend almost entirely on imported raw materials. However, in 1955 the B.P.I. Raw Material Project was set up with the objective of providing as much of the raw material as possible from indigenous sources.

In 1957, Arnold Nordal was appointed as a United Nations advisor to assist the B.P.I. Raw Material Project with its work. From 1957 to 1961, Nordal studied the possible utilization of the medicinal plants in the Myanmar flora. For his study, Nordal contacted those he considered the most important representatives of the indigenous system of medicine. These included Buddhist monks, medicine men, and drug traders. Books and written sources were also used during his research resulting in the compilation of his 1963 publication, The Medicinal Plants and Crude Drugs of Burma. In the course of his work, he also built a herbarium of these medicinal plants, created a collection of the corresponding crude drugs, and collected as much information as possible on the medicinal tradition connected with the plants.

Subsequent work includes the following:

• Mya Bwin D, Sein Gwan U (1967) Burmese Indigenous Medicinal Plants. Burma Medicinal Research Institute, Rangoon.

• Perry LM (1980) Medicinal Plants of East and Southeast Asia: Attributed Properties and Uses. 620 pp. The MIT Press, Cambridge and London.

• Agricultural Corporation (1980) Burmese Medicinal Plants. 501 pp. Rangoon: Agricultural Corporation. (In Burmese).

• Department of Traditional Medicine (No date [199-]) Medicinal Plants of Myanmar. Monograph. Ministry of Health, Myanmar. Accessed from http://apps.who.int/medicinedocs/en/m/abstract/Js20298en/.

• Forest Department (1999) Medicinal Plants of Popa Mountain Park. 18 pp. Yangon, Myanmar: Ministry of Forestry.

• Kress WJ, DeFilipps RA, Farr E, Daw Yin Yin Kyi (2003) A Checklist of the Trees, Shrubs, Herbs, and Climbers of Myanmar. National Museum of Natural History, Washington DC.

• Thein Swe, Sein Win (2005) Herbal Gardens and Cultivation of Medicinal Plants in Myanmar. 5 pp. World Health Organization. Regional Office for South-East Asia. Pyongyang, DPR Korea.

• Tun, U Kyaw, U Pe Than et al. (Update 2006) Myanmar Medicinal Plant Database.

The Ministry of Health in Myanmar established the Department of Traditional Medicine in 1989, and it was upgraded and reorganized in 1998 (Thein Swe and Sein Win 2005).

Traditional medicine is widely practiced in Myanmar by the majority of the population either as an alternate or as a supplement to modern medicine (Thein Swe and Sein Win 2005). The social groups and traditional communities that have generated the knowledge of traditional medicine in Myanmar include Buddhist monks, sesayas (local doctors), ambulating medicine men, traders in the local drug bazaars, ambulating drug traders, and professional drug collectors (Nordal 1963). Old Burmese scriptures that contain medical traditions and health problems in addition to religious matters are written in a Burmese alphabet and language than can only be translated with special training. Buddhist monks have translated these scriptures, often written on palm leaves (*Corypha umbraculifera* L.) or on bamboo covered with the sap of the black-varnish tree (*Melanorrhoea usitata* Wall.), into ordinary Burmese and English (Nordal 1963). Sesayas are practitioners of local medical traditions whose knowledge has been handed down through their ancestors. Sesayas and their helpers prepare medicines in laboratories in their own homes. Ambulating medicine men are free lancers that travel from place to place accompanied by an apprentice. Drug traders of the local open-air bazaars are often prepared to share knowledge about the properties of their goods. Ambulating drug traders are mostly *Ghurkas* (people originating from Tibet) who would spread their products in the streets for display. Professional drug collectors make their living collecting crude drugs for the drug bazaars and for the sesayas, and they often have extensive and reliable knowledge of the medicinal local flora (Nordal 1963).

### History and knowledge of the Myanmar flora

Botanical exploration of the Southeast Asian country of Myanmar (formerly Burma), which spans both tropical and subtropical biomes, began in the 1880s when the country was under the rule of the British (Kress et al. 2003). The botanical study of the British colonial system, including India and parts of Asia, resulted in partial plant lists of Myanmar such as Kurz’s The Forest Flora of British Burma (1877) and Hooker’s Flora of the British India (1894). Botanical investigations of the region sharply decreased soon after World War II. Myanmar is exceptionally rich in plant diversity, but very few new plant collections had been made in this area during the second half of the 1900s (Kress et al. 2003). The first list of plants specifically for Myanmar was compiled in 1922 by J.H. Lace and published in the List of Trees, Shrubs, Herbs and Principal Climbers, etc., recorded from Burma. The original edition includes 2,483 species, and the last published edition of 1987 has about 7,000 species. Kress et al. (2003) provided a more comprehensive list based on an inventory of specimens from select herbaria, advice from taxonomic specialists, and records from regional floras. The treatment lists over 11,800 species. The knowledge of the flora is still growing, as the native status of many species is incomplete.

### The geology, climate, and vegetation types of Myanmar

Myanmar occupies an area of 678,033 sq. km in Southeast Asia. It is bordered by India, Bangladesh, and the Bay of Bengal on the west, China to the north and northeast, Laos and Thailand to the east, and the Andaman Sea to the south. With the exception of the centrally located Ayeyarwady valley and delta, the most populated area, the terrain is generally hilly and mountainous.

The climate is mostly monsoonal, with cloudy, rainy, hot humid summers (June to September, southwest monsoon) and less cloudy, scant rainfall, mild temperatures, lower humidity during the winter (December to April, northeast monsoon). Local climate, which has a major influence on the diversity and distribution of plant species, is determined by the combination of temperature, rainfall, and elevation. Geology and the resultant soils are major controlling factors in the local distribution of forest types and of individual species, although to some extent climate and soil counteract one another (Stamp 1925).

The vegetation consists of tropical lowland evergreen rain forest, primarily in the south; tropical hill evergreen rain forest and temperate evergreen rain forest above 900 m in the east, north, and west; semi-evergreen rain forest in a narrow belt bordering an arid central plain; mixed deciduous forest with teak (*Tetona grandis*) and dry dipterocarp forest centrally; coniferous forests in Shan and Chin States, with *Pinus khasya* between 1200–2500 m on dry slopes; oak and rhododendron forests on wetter slopes; and dry forest and scrub formations where average annual rainfall is below 100 cm. Additionally, large tracts of bamboo forest are scattered throughout the country.

As recently as 1931, Myanmar was nearly three-quarters forested (Murphy 1931). The Myanmar forest department estimates that closed and degraded forest together currently constitute 343,767 km or approximately 51% of the total area of the country. Myers (1988), quoting Forest Department figures, stated that about 1420 sq. km per annum of primary forest is transformed by shifting cultivation, while Kyaw Tint and Tun Hla (1981) have estimated that open forest increases annually by approximately 278,000 ha per year.

### Bringing Burmese text to an English reading audience

The information presented here was compiled utilizing data from written sources and databases on Asian and Myanmar medicinal plants; the Checklist of the Trees, Shrubs, Herbs, and Climbers of Myanmar (2003), which up-dates the largely unavailable earlier checklists with a more complete treatment of the grasses, orchids, and herbs; and, importantly, the English translation (provided by Thi Thi Ta) of Burmese Medicinal Plants (Agricultural Corporation 1980), an important and extensive book on Burmese medicinal plants, how they are utilized, and their specific preparations.

The families, genera, and species are arranged alphabetically under the following categories: Ferns, Gymnosperms, and Angiosperms. Under each genus, the species are listed under the Latin binomial followed by the author(s) and synonyms, English and Myanmar common names, global range and approximate distribution in Myanmar (including if cultivated), uses in Myanmar (for the many species from the newly translated Burmese publication, preparation is also included as well as detailed uses), notes, and references. If the species is listed in the IUCN Red List of Threatened Species (IUCN 2017), the conservation assessment of the species is included as well.

The family and genus names utilized here are in accordance with those given as taxa accepted in Angiosperm Phylogeny Website (Stevens 2017) and The Plant List (2013). Synonyms are included when the synonym is used in the original referenced texts.

Myanmar distributions presented here are those given by Kress et al. (2003). The distributions should only be considered approximate since, “due to lack of comprehensive herbarium collections of Myanmar plants, accurate determinations of the geographic distribution of taxa are still problematic” (Kress et al. 2003). Distributions are based on data from the original list, existing specimens, and estimates from taxonomic specialists. If the taxon is known to be common, the distribution is designated as “wide”. Common names given here come from the various sources, but most are those given in Kress et al. (2003).

### Conservation and sustainability of medicinal plant species

**Figure 1.**
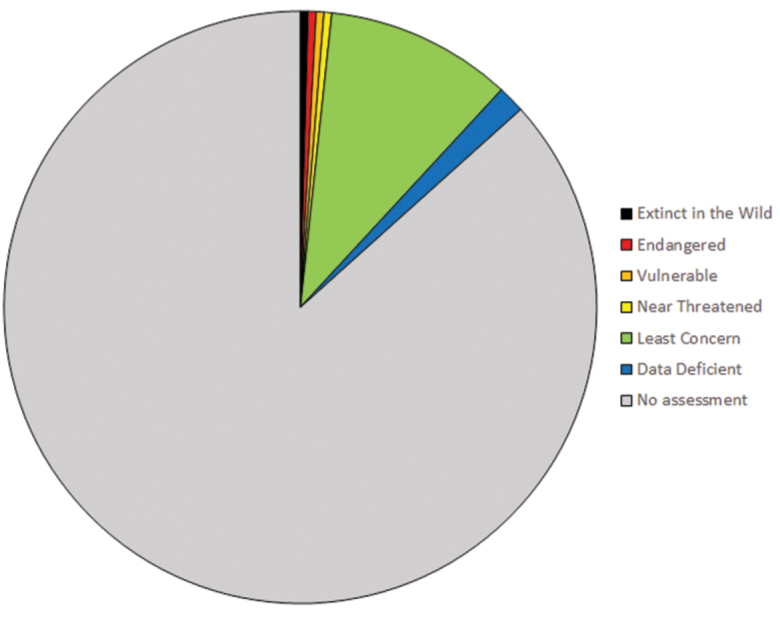
IUCN conservation assessments of the medicinal plant species treated in this study (IUCN 2017).

This list contains 123 families, 367 genera, and 472 species of medicinal plants. Of the 472 species, only 63 (13%) have been assessed for conservation status in the IUCN Red List of Threatened Species (IUCN 2017) (Figure 1). Two species are listed as Extinct in the Wild: *Brugmansia arborea* (L.) Steud. and *Brugmansia suaveolens* (Humb. & Bonpl. ex Willd.) Bercht. & J.Presl. Both species survive only in cultivation, and thus the size of wild populations of these species is zero. Four species are deemed threatened: *Coptis teeta* Wall. and *Cupressus goveniana* Gordon are listed as Endangered, and *Aquilaria malaccensis* Lam. and *Santalum album* L. are listed as Vulnerable. Exploitation, unregulated collection, and forest degradation are the primary threats to these species. Two species are listed as Near Threatened (*Cycas rumphii* Miq. and *Dimocarpus longan* Lour.), 48 species as Least Concerned, and seven species as Data Deficient.

According to Botanic Gardens Conservation International’s ThreatSearch database (BGCI 2017), which is a comprehensive list of threatened plant species at the global, national, and regional scales using both IUCN and non-IUCN methodologies, over 75% (355 species) of the medicinal plants listed here have been assessed for conservation status at one or more scales. These assessments include those listed in the Red Lists of Canada & the United States (NatureServe 2017), Central Asia (Eastwood et al. 2009), China (Wang and Xie 2004), Jordan (Taifour and El-Oqlah 2014), Luxembourg (Colling 2005), South Africa (SANBI 2017), and others, as well as preliminary assessments of the Lesser Antilles (Carrington et al. 2017), the Philippines (Fernando et al. 2008), Puerto Rico (Miller et al. 2013), and individual taxonomic treatments. Of the 355 species that have received national and global assessments, 101 species were deemed threatened (15 Critically Endangered, 31 Endangered, 55 Vulnerable), 66 Near Threatened, 257 Least Concerned, and 29 Data Deficient (totals do not add as most species received multiple assessments and were placed in multiple threat categories). Just under 25% (117 species) have not been assessed at any scale.

**Figure 2.**
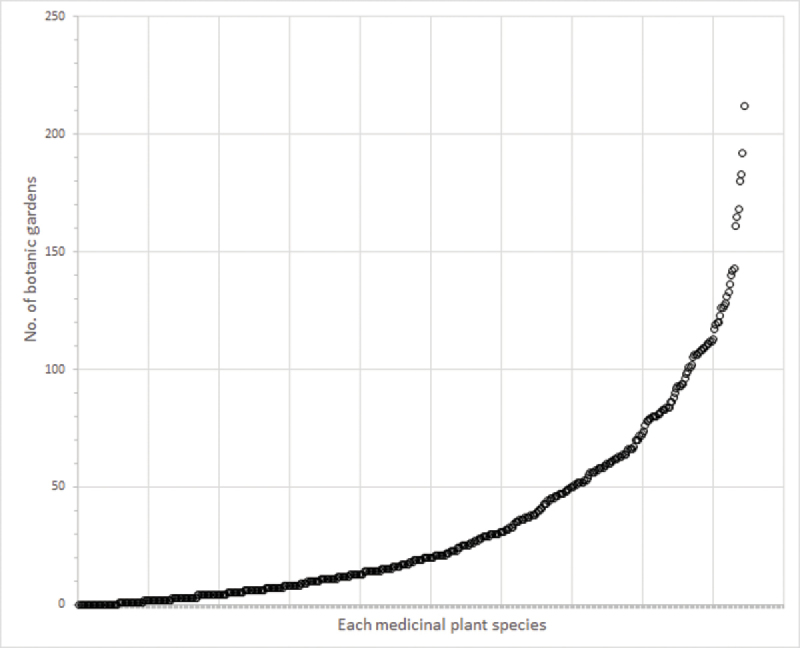
The number of botanic gardens worldwide that have digitally recorded accessions of each of the 472 medicinal plant species treated in this study.

According to BGCI’s PlantSearch database (BGCI 2017), a comprehensive list of the botanic garden accessions, 444 species (94%) of the medicinal plants listed here are held within the living collections of botanic gardens worldwide, while 28 species (6%) are not found any botanic garden (Figure 2). The median number of botanic gardens a medicinal plant species is found in is 18 gardens. Eighteen species are found in only one botanic garden, while 125 species are found in 2–10 botanic gardens. The species found in the greatest number of gardens is *Taxus baccata* L., which is found in 212 botanic gardens, while *Salvia officinalis* L. is found in 192 botanic gardens worldwide. Of the threatened species listed in the IUCN Red List, the Endangered species *Coptis teeta* is found in three botanic gardens and the Endangered *Cupressus goveniana* is found in 45 botanic gardens. The Vulnerable *Aquilaria malaccensis* is found in five gardens while the Vulnerable *Santalum album* is found in 22 gardens.

Mounce et al. (2017) argue for targeted strategies to enhance the value of living collections at botanic gardens, including a focus on under-represented phylogenetic lineages, environmental niches, life histories, and medicinal, ethnobotanical, and crop plants. Further, to reduce the pressures of harvesting plants from wild resources, there are calls for conservation strategies (e.g., in situ and ex situ conservation and cultivation practices) and resource management (e.g., sustainable use practices) to sustain wild populations of medicinal plant species (Schippmann et al. 2002, Chen et al. 2016).

### References cited in Introduction

Agricultural Corporation (1980) Burmese Medicinal Plants. Agricultural Corporation, Rangoon. [In Burmese]

BGCI (2017) ThreatSearch online database. Botanic Gardens Conservation International, Richmond. http://www.bgci.org/threat_search.php [accessed 29.08.2017]

Carrington CMS, Krupnick GA, Acevedo-Rodríguez P (2017) Herbarium-based preliminary conservation assessments of Lesser Antillean endemic seed plants reveal a flora at risk. The Botanical Review 83(2): 107–151. https://doi.org/10.1007/s12229-017-9182-5

Chen SL, Yu H, Luo HM, Wu Q, Li CF, Steinmetz A (2016) Conservation and sustainable use of medicinal plants: problems, progress, and prospects. Chinese Medicine 11: 37. https://doi.org/10.1186/s13020-016-0108-7

Colling G (2005) Red List of the Vascular Plants of Luxembourg. Musée National d’Histoire Naturelle, Luxembourg. https://ps.mnhn.lu/ferrantia/publications/Ferrantia42.pdf

Convention on Biological Diversity (CBD) (2002) Decision VI/9, Global Strategy for Plant Conservation, 2002–2010. Sixth Ordinary Meeting of the Conference of the Parties to the Convention on Biological Diversity (COP 6). The Hague, The Netherlands. http://www.cbd.int/decision/cop/?id=7183

Eastwood A, Lazkov G, Newton A (2009) The Red List of Trees of Central Asia. Fauna & Flora International, Cambridge. https://www.bgci.org/files/Worldwide/News/red_list_of_trees_of_central_asia.pdf

Fernando ES, Co LL, Lagunzad DA, Gruezo WS, Barcelona JF, Madulid DA, Lapis AB, Texon GI, Manila AC, Zamora PM (2008) Threatened plants of the Philippines: A preliminary assessment. Asian Life Sciences Suppl. 3: 1–52.

IUCN (2017) IUCN Red List of threatened species. IUCN, Gland. http://www.iucnredlist.org/ [accessed 01.08.2017]

Kress WJ, DeFilipps RA, Farr E, Daw Yin Yin Kyi (2003) A Checklist of the Trees, Shrubs, Herbs, and Climbers of Myanmar. Contributions from the United States National Herbarium 45: 1–590. http://botany.si.edu/pubs/CUSNH/vol_45.pdf

Kurz S (1877) Forest Flora of British Burma. Supdt., Government printer, Calcutta.

Kyaw Tint, Tun Hla (1991) Forest Cover of Myanmar, the 1988 Appraisal. National Forest Management and Inventory, FAO: MYA/85/003. Rome.

Miller SJ, Krupnick GA, Stevens H, Porter-Morgan H, Boom B, Acevedo-Rodríguez P, Ackerman J, Kolterman D, Santiago E, Torres C, Velez J (2013) Toward Target 2 of the Global Strategy for Plant Conservation: an expert analysis of the Puerto Rican flora to validate new streamlined methods for assessing conservation status. Annals of the Missouri Botanic Garden 99(2): 199–205. https://doi.org/10.3417/2011121

Mounce R, Smith P, Brockington S (2017) Ex situ conservation of plant diversity in the world’s botanic gardens. Nature Plants 3: 795–802. https://doi.org/10.1038/s41477-017-0019-3

Murphy M (1931) The geography of Burma. Journal of Geography 30: 17–33. https://doi.org/10.1080/00221343108987159

Myers N (1988) Threatened biotas: “Hotspots” in the tropical forestry. Environmentalist 8: 1–20. https://doi.org/10.1007/BF02240252

NatureServe (2017) NatureServe Explorer: An online encyclopedia of life [web application]. Version 7.1. NatureServe, Arlington, Virginia. http://explorer.natureserve.org [accessed 29.08.2017]

The Plant List (2013) Version 1.1. Published on the Internet. http://www.theplantlist.org/ [accessed 29.08.2017]

SANBI (2017) Red List of South African Plants version 2017.1. http://redlist.sanbi.org/index.php [accessed 29.08.2017]

Schippmann U, Leaman DJ, Cunningham AB (2002) Impact of cultivation and gathering of medicinal plants on biodiversity: Global trends and issues. In: FAO (Eds) Biodiversity and the Ecosystem Approach in Agriculture, Forestry and Fisheries. FAO, Rome, 142–167. http://www.fao.org/docrep/005/AA010E/AA010E00.HTM

Stamp LD (1925) The Vegetation of Burma from an Ecological Standpoint. Research Monograph No. I. Thacker, Spink and Co., Calcutta.

Stevens PF (2017) Angiosperm Phylogeny Website. Version 14, July 2017. http://www.mobot.org/MOBOT/research/APweb/ [accessed 29.08.2017]

Thein Swe, Sein Win (2005) Herbal Gardens and Cultivation of Medicinal Plants in Myanmar. 5 pp. World Health Organization. Regional Office for South-East Asia. Pyongyang, DPR Korea.

Taifour H, El-Oqlah A (2014) Jordan Plant Red List. Jordan Royal Botanic Garden, Amman. http://royalbotanicgarden.org/sites/default/files/files/Jordan%20Plant%20Red%20List%20(email)%20-%20Vol%201.pdf

Wang S, Xie Y (2004) China Species Red List. Vol. 1 Red List. Higher Education Press, Beijing, China.

## Ferns

### Dennstaedtiaceae (Bracken Fern family)

#### 1. *Pteridium* Gled. ex Scop.

##### Pteridium aquilinum

Taxon classificationPlantaePolypodialesDennstaedtiaceae

(L.) Kuhn

###### Names.

**Myanmar**: *boktaung, wetkyein*. **English**: brake, braken, hog-pasture brake, pasture brake.

###### Range.

Cosmopolitan.

###### Use.

*Stem*: Rhizome used as an anthelmintic.

###### Notes.

Perry (1980) discusses the medicinal uses of the species in China, Indo-China, and New Guinea.

Reported constituents include hydrocyanic acid, catechuic tannins, antivitamin B, antivitamin K, and pteridine. The rhizome contains filicic acid, essential oil, resin, some tannin, filicotannic acid, fatty oil, wax, aspidinol, sugar, gum, and starch (Perry 1980).

###### Reference.

Nordal (1963).

### Equisetaceae (Horsetail family)

#### 1. *Equisetum* L.

##### Equisetum ramosissimum subsp. debile

Taxon classificationPlantaeEquisetalesEquisetaceae

(Roxb. ex Vaucher) Hauke (= Equisetum debile Roxb. ex Vaucher)

###### Names.

**Myanmar**: *myet-sek*. **English**: weak horsetail.

###### Range.

Europe from Loire, southern Bavaria and central Russia southwards, in isolated localities in Brittany (France), the Netherlands and northern Germany; Asia; Africa; and America.

###### Use.

*Whole plant*: Used to treat gonorrhea.

###### Notes.

In India the whole plant is used for gonorrhea and as an abortifacient (Jain and DeFilipps 1991). In China the species is used internally to treat dysentery; also to improve eyesight (Duke and Ayensu 1985). In Malaysia it is used for pain, especially arthritic pain; in Indonesia it is used externally to treat bruises, fractures, and arthritis; and in Korea, China, Taiwan, and Indo-China it is used internally to treat dysentery (Perry 1980).

Reported constituents of *Equisetum* include fatty oil, silicic acid, linoleic acid, equisetonine, equisetic acid, and equisetine (Perry 1980).

###### Reference.

Nordal (1963).

### Gleicheniaceae (Forking Fern family)

#### 1. *Dicranopteris* Bernh.

##### Dicranopteris linearis

Taxon classificationPlantaeEquisetalesEquisetaceae

(Burm.f.) Underw. (= Gleichenia linearis (Burm.f.) C.B.Clarke)

###### Name.

**English**: savannah fern.

###### Range.

Malay Peninsula to Sumatra.

###### Uses.

*Whole plant*: Used as an antipyretic, antiasthmatic, and anthelmintic.

###### Notes.

In Indo-China the plant is considered to be anthelmintic. On the Malay Peninsula crushed leaves are applied as a poultice for fever, a decoction is used as an embrocation, or an infusion may be drunk (“large and strong doses are apparently injurious”) (Perry 1980).

###### Reference.

Nordal (1963).

## Gymnosperms

### Cupressaceae (Cypress family)

#### 1. *Cupressus* L.

##### Cupressus goveniana

Taxon classificationPlantaeGleichenialesGleicheniaceae

Gordon

###### Name.

**English**: California cypress.

###### Range.

California, in North America. Cultivated in Myanmar.

###### Conservation status.

Endangered [EN B2ab(ii,iii,v)] (IUCN 2017).

###### Uses.

Plant used for medicinal purposes (exact uses not given in Nordal 1963).

###### Notes.

A member of this genus, *Cypressus funebris*, is used in China to dispel colds; the leaves are antiperiodic and provide a remedy for bleeding piles, hematuria, and menorrhea. In Indo-China another member of the genus, *Cypressus hodginsii*, is known to have vaso-constrictory and astringent properties (Perry 1980).

The monocyclic sesquiterpene fokienol is a reported chemical constituent of *Cypressus hodginsii* Dunn (= *Fokienia hodginsii* (Dunn) Henry & Thomas) (Perry 1980).

###### Reference.

Nordal (1963).

### Cycadaceae (Cycad family)

#### 1. *Cycas* L.

##### Cycas rumphii

Taxon classificationPlantaeCycadalesCycadaceae

Miq.

###### Names.

**Myanmar**: *mondaing*. **English**: cycad.

###### Range.

Northern Australia and Malay Archipelago. In Myanmar, found in Taninthayi and Yangon.

###### Conservation status.

Near Threatened [NT] (IUCN 2017).

###### Uses.

*Male bracts*: Used as aphrodisiac, narcotic, and stimulant. *Fruit* or *Seed*:

Applied to ulcers, wounds (including malignant and varicose), skin lesions, and used for various skin diseases.

###### Notes.

The medicinal uses of this species in India are discussed in Jain and DeFilipps (1991). Medicinal uses of the species in Indo-China, Indonesia, the Philippines, Admiralty Island, New Guinea, and the Solomon Islands are discussed in Perry (1980). The application may be the poisonous juice of the fruit, the raw seed grated or macerated, or roasted, powdered and mixed with coconut oil (Perry 1980).

###### References.

Nordal (1963), Perry (1980).

### Taxaceae (Yew family)

#### 1. *Taxus* L.

##### Taxus baccata

Taxon classificationPlantaePinalesTaxaceae

L.

###### Names.

**Myanmar**: *kyauk-tinyu*. **English**: yew.

###### Range.

Europe, North Africa, western Asia. In Myanmar found in Chin and Shan.

###### Conservation status.

Least Concern [LC] (IUCN 2017).

###### Uses.

*Leaf, Fruit*: Used as an antispasmodic, sedative, and as an emmenagogue.

###### Notes.

In India the leaf and fruit are used as an antispasmotic, sedative, and emmenagogue (Jain and DeFilipps 1991). The leaf is also used as an aphrodisiac; to treat epilepsy, asthma, indigestion, and bronchitis. Other medicinal uses for this species include expectorant, pectoral, sedative, stomachic, tonic; abortifacient, antifertility (chemical found in plant shown to be effective for this purpose), contraceptive; for headache, bilious, calculus, for cancer, carminative, cyanogenetic, epilepsy, lithontriptic, medicine Tacholm; giddiness, nerves, spasm; poison, vermifuge, insecticide (Duke 2009).

The leaves and seeds of *Taxus* species contain the alkaloid taxine which is *poisonous*, “and while *Taxus* is sometimes used as medicine this also has caused instances of poisoning” (Perry 1980).

###### Reference.

Nordal (1963).

## Angiosperms

### Acanthaceae (Acanthus family)

#### 1. *Acanthus* L.

##### Acanthus ilicifolius

Taxon classificationPlantaeLamialesAcanthaceae

L.

###### Names.

**Myanmar**: *kaya-chon*, *kha-yar*, *kha-yar-chon*. **English**: holly-leaved acanthus, sea holly.

###### Range.

India to Polynesia and Australia. In Myanmar, found in Ayarwady, Rakhine, Taninthayi, and Yangon.

###### Conservation status.

Least Concern [LC] (IUCN 2017).

###### Uses.

*Shoot*: Used to treat snakebite. *Leaf*: Used for rheumatism.

###### Notes.

The medicinal uses of this species in India are discussed in Jain and DeFilipps (1991) as follows: Stem- anti-cancer; root- also anti-cancer, and for chronic fever. Perry (1980) discusses the medicinal uses of the species in China, Indo-China, Indonesia, and the Philippines.

###### Reference.

Perry (1980).

#### 2. *Andrographis* Wall. ex Nees

##### Andrographis paniculata

Taxon classificationPlantaeLamialesAcanthaceae

(Burm.f.) Nees

###### Names.

**Myanmar**: *sega-gyi*, *se-khar-gyi*, *hsay-kha gyi*, *ngayoke kha*. **English**: creat, creyat root, king of bitters.

###### Range.

Subcontinent of India. In Myanmar, found in Kachin, Kayin, Magway, Mandalay, and Sagaing.

###### Uses.

Cool and bitter in taste, controls phlegm and gall bladder function, stimulates appetite, reduces fever, and is particularly good as a remedy for children. *Whole plant*: Made into medicines that reduce fever, aid digestion, and give strength. The liquid from boiling the plant is used to treat headaches, indigestion, loose bowels, dysentery, shooting pains from gas in the intestines, and fevers; can also be mixed with powdered *zee-hpyu*, *hpan-khar* (*Terminalia chebula*) and *thit hseint* (*Terminalia bellirica*) to remedy edema, abdominal swelling, leprosy, headaches, stiff neck, and dizziness. *Leaf*: Used in medicines that lower fever, neutralize poisons, and treat the gall bladder, as well as in making of *shar-put-hsay* (commonly used traditional medicine in form of grayish brown powder rolled into nuggets). *Leaf* and *Root*: Used as febrifuge, stomachic, tonic and anthelmintic.

###### Notes.

The medicinal uses of this species in India are discussed in Jain and DeFilipps (1991). Medicinal uses of this species in China are discussed in Duke and Ayensu (1985).

###### References.

Nordal (1963), Agricultural Corporation (1980), Forest Department (1999).

#### 3. *Avicennia* L.

##### Avicennia officinalis

Taxon classificationPlantaeLamialesAcanthaceae

L.

###### Name.

**English**: gray mangrove.

###### Range.

Maritime. South and southeastern Asia, northern Australia, and East Africa.

###### Conservation status.

Least Concern [LC] (IUCN 2017).

###### Uses.

*Root*: Considered to be an aphrodisiac. *Seed*: Used in poultices.

###### Notes.

In Taiwan the fruit, mixed with butter and made into a paste, is smoothed on to prevent the bursting of smallpox pustules; in Indo-China the bark is used to heal cutaneous affections, especially scabies; in Indonesia a resinous substance exuded from the bark “acts as a contraceptive, and apparently can be taken all year long without ill effects”; and in the Philippines the seeds are a maturative and a cicatrizant of ulcers, also resin from the sapwood is applied locally to snakebites (Perry 1980).

The bark contains tannin and lapachol (Perry 1980).

###### Reference.

Perry (1980).

#### 4. *Barleria* L.

##### Barleria prionitis

Taxon classificationPlantaeLamialesAcanthaceae

L.

###### Names.

**Myanmar**: *leik-su-ywe*, *leik-hsu shwe*, *leik tha-shwe war*. **English**: barleria, porcupine flower.

###### Range.

Tropical Asia, Africa, and India. In Myanmar, found in Kachin, Magway, Mandalay, Sagaing, and Yangon, especially in fields and pastures.

###### Uses.

Bitter and astringent in taste, highly beneficial for skin and blood diseases. *Whole plant*: Crushed, cooked with sesame oil and applied to itches, ringworm and boils. *Whole plant, Leaf*: Used as diuretic in dropsy and as febrifuge. *Stem* and *Leaf*: Crushing the leaves together with the stems and branches, stewing them in a mixture of one part sesame oil to two parts water and straining the mixture provides an oil that can be applied to long-standing sores. *Leaf*: Made into an ash and taken with fermented rice washing water to bring down swelling from edemas and dropsy; mixed with butter and applied to longstanding sores, to help them heal quickly. Leaves boiled to make a strong tea, and the mixture held in the mouth to strengthen loose teeth. Juice from crushing leaves- applied to scorpion sting will neutralize the poison, also used to treat inflamed areas; mixed with either honey, sugar, or warm water and given to cure children with coughs, fever and bronchitis; also used to treat chronic cough. Juice from grinding the leaves applied to treat fungus infections on the soles of the feet and between the toes. *Roots*: Ground and applied to bring down inflammation and infection in swellings, bumps, and sores.

###### Note.

In India the root is placed on boils and glandular swellings; the bark is used for dropsy; and the leaf for toothache and rheumatism (Jain and DeFilipps 1991).

###### References.

Nordal (1963), Agricultural Corporation (1980).

#### 5. *Hygrophila* R.Br.

##### Hygrophila auriculata

Taxon classificationPlantaeLamialesAcanthaceae

(Schumach.) Heine (= Asteracantha longifolia Nees; Hygrophila spinosa T. Anderson)

###### Names.

**Myanmar**: *le-padu*, *su-padang*. **English**: hygrophila.

###### Range.

Wet places in Indo-China, Myanmar, Bangla Desh, India, Nepal, Sri Lanka, Pakistan Punjab, and Tropical Africa. In Myanmar, found in Bago.

###### Conservation status.

Least Concern [LC] (IUCN 2017).

###### Uses.

*Leaf*: Used in treating jaundice. *Leaf, Root, Seed*: Used as expectorant, and diuretic in dropsy. *Root*: Used to treat rheumatism. *Seed*: Employed as an aphrodisiac.

###### Notes.

The medicinal uses of this species in India are discussed in Jain and DeFilipps (1991) as follows: The whole plant is used for malarial fever; the leaf and seed as a diuretic, for jaundice, cough, dropsy, rheumatism, and urogenital diseases; the seed as an aphrodisiac; and the bulb for tubercular fistula, sores, skin cancer, dropsy, and swelling of the face and body. Primarily the leaves are used for poulticing fresh wounds, sprained limbs, swellings, abscesses, boils, and headaches (Perry 1980).

Reported constituents in species belonging to this genus include an alkaloid; various enzymes; and linoleic, oleic, and ricinoleic acids (Perry 1980).

###### References.

Nordal (1963), Perry (1980).

##### Hygrophila phlomoides

Taxon classificationPlantaeLamialesAcanthaceae

Nees

###### Names.

**Myanmar**: *hsay-dan*, *meegyaung-kun-hpat*, *migyaung-kunbat*. **English**: Burma linseed.

###### Range.

Temperate Asia: China and Tropical Asia: Indian subcontinent. In Myanmar, found in Bago, Taninthayi, and Yangon.

###### Uses.

*Seed*: Used for making medicines to cure sore eyes, for flatulence, and for discoloration and fungal infections of the skin. Crushed and used as a poultice over festering and long-standing sores.

###### Notes.

In India the leaf is used for boils and headache (Jain and DeFilipps 1991).

In East and Southeast Asia, primarily the leaves are used for poulticing fresh wounds, sprained limbs, swellings, abscesses, boils, and headache (Perry 1980).

###### Reference.

Agricultural Corporation (1980).

#### 6. *Justicia* L.

##### Justicia adhatoda

Taxon classificationPlantaeLamialesAcanthaceae

L. (= Adhatoda vasica Nees)

###### Names.

**Myanmar**: *my-yar-gyi*, *ye-magyi*, *htingra-hpraw* (Kachin), *hla brairot* (Mon). **English**: adulsa, Malabar nut tree.

###### Range.

India, Sri Lanka, Malaysia, Myanmar. Cultivated in the tropics. Widely distributed in Myanmar.

###### Uses.

*Whole plant*: Used in medicine to remove phlegm, and for excessive menses. *Leaf*: Astringent and bitter, the leaves have cooling properties that regulate phlegm and bile, ease diarrhea, alleviate coughing and coughing up blood, and relieve chronic asthma. They also alleviate coughing with fever, bad breath, and swellings in the lower extremities. To relieve pain and urinary infections, three tablespoons of liquid from boiling leaves, reduced to one-third starting volume, are ingested. Leaves dried in the shade, converted to ash, and ground to a fine powder can be pressed onto gums and teeth for toothaches, bleeding gums, and loose teeth. Leaves are also used to make medicines for eye ailments. Stewing the leaves and taking the liquid used to treat dysentery; also, for dysentery, male-related weaknesses, and excessive menstruation, liquid from boiling a handful of leaves in water reduced to one-third the starting volume is taken three times a day. The juice of crushed young leaves with either wine or honey is used to treat whooping cough. Leaf extract is antiseptic. *Flower*: About one tablespoon of the juice squeezed from the flowers and leaves can be taken with a moderate amount of rock sugar to for bile problems and for vomiting and otherwise passing blood. *Fruit*: For vomiting and otherwise passing blood, three tablespoons of liquid from *kyazu* (*Terminalia citirina*) fruit soaked in leaf juice can be taken. *Root* (or *Leaf*): To treat asthma and coughs, one tablespoon of juice from the crushed roots or leaves mixed with moderate amounts of rock sugar and rock salt can be taken. Black *mu yargyi* (probably *Adhatoda vasica* = *Justicia adhatoda*) root can be made into a paste with cold water and rubbed onto scorpion sting to neutralize the venom. The root is also a component in insecticides.

###### Notes.

In India the species is used in Ayurvedic medicine as a blood purifier and antispasmodic, as well as a treatment for bronchitis, asthma, tuberculosis, coughing, and intestinal worms (Jain and DeFilipps 1991).

“Reported constituents of the leaves are a very small amount of essential oil, vasicine (an alkaloid), and adhatodic acid. The first two have therapeutic properties. The alkaloid produces a slight fall in blood pressure followed by a rise to the original level, an increase persistent broncho-dilator effect.” Antiseptic and insecticidal properties are attributed to it (Perry 1980).

###### References.

Nordal (1963), Agricultural Corporation (1980), Perry (1980), Forest Department (1999).

#### 7. *Peristrophe* Nees

##### Peristrophe bicalyculata

Taxon classificationPlantaeLamialesAcanthaceae

(Retz.) Nees

###### Name.

**English**: panicled peristrophe.

###### Range.

Tropical Africa, Pakistan, India, Myanmar, Malaya, and Indo-China. In Myanmar, found in Bago.

###### Use.

*Whole plant*: Used as an antidote for snake-poison.

###### Note.

In India the whole plant, macerated in rice (*Oryza sativa*), is used as an antidote to snake poison (Jain and DeFilipps 1991).

###### Reference.

Nordal (1963).

#### 8. *Strobilanthes* Blume

##### Strobilanthes auriculatus

Taxon classificationPlantaeLamialesAcanthaceae

Nees

###### Names.

**Myanmar**: *hmaw-yan*, *paung-thaung*, *saingnan*. **English**: Mexican petunia.

###### Range.

Tropical Asia. Widely distributed in Myanmar.

###### Uses.

*Whole plant*: Used as an antidote for snake poison. *Leaf*: Used to treat intermittent fever.

###### Note.

In India “Pounded leaves are rubbed onto the body during the cold period of an intermittent fever.” (Jain and DeFilipps 1991).

###### Reference.

Nordal (1963).

#### 9. *Thunbergia* Retz.

##### Thunbergia erecta

Taxon classificationPlantaeLamialesAcanthaceae

(Benth.) T. Anderson

###### Names.

**Myanmar**: *kwa-nyo*. **English**: black-eyed Susan vine, bush clock-vine.

###### Range.

Tropical and southern Africa. In Myanmar, found in Bago, Mandalay, and Yangon.

###### Use.

*Leaf*: Used for treating bile disorders.

###### Note.

In India the leaf is used as an ingredient of headache poultices (Jain and DeFilipps 1991).

###### Reference.

Forest Department (1999).

##### Thunbergia laurifolia

Taxon classificationPlantaeLamialesAcanthaceae

Lindl.

###### Names.

**Myanmar**: *kyi-kan-hnok-thi*, *kyini-nwe*, *new-nyo*, *pan-ye-sut-nwe*. **English**: laurel clock vine, laurel-leaved clockvine, laurel-leaved thunbergia, purple allamanda.

###### Range.

Southeast Asia. In Myanmar, found in Bago, Kachin, Mandalay, and Yangon.

###### Use.

*Flower*: Said to be a good medicine for the eyes.

###### Notes.

In India leaf juice is placed in the ear to treat deafness and is drunk for menorrhagia (Jain and DeFilipps 1991). In China the leaves are used as a remedy for excessive mensus and are also applied to wounds and ulcers. On the Malay Peninsula juice from crushed leaves is taken and used in a poultice applied to cuts and boils; the juice is also put in the ear to treat deafness (Perry 1980).

###### Reference.

Perry (1980).

### Achariaceae (Acharia family)

#### 1. *Hydnocarpus* Gaertn.

##### Hydnocarpus kurzii

Taxon classificationPlantaeMalpighialesAchariaceae

(King) Warb.

###### Names.

**Myanmar**: *kalaw*, *kalaw-so*. **English**: chaulmoogra.

###### Range.

Tropical Asia. Found growing in natural gullies and mountain slopes of Myanmar, including in Chin, Kachin, Kayin, areas around Pyinmana, and other evergreen forests.

###### Conservation status.

Data Deficient [DD] (IUCN 2017).

###### Uses.

*Bark, Fruit*, and *Seed (oil)*: (bitter and hot) have healing properties. Can be used to induce vomiting and neutralize poisons, as well as to alleviate aches, indigestion, flatulence, and infections. *Bark*: An ingredient in medicines to reduce fever. *Fruit*: Eaten as a remedy for leprous sores, boils, and vomiting. Applied topically for aches and pains; the oil is known for its blood-purifying properties. As the oil has heat, it can kill germs and is most commonly used to treat leprosy and other skin infections.

###### Notes.

In India the bark is used for fever, the oil of the seed for leprosy (Jain and DeFilipps 1991). The species is a source of chaulmoogra oil.

###### Reference.

Agricultural Corporation (1980), Ministry of Health (2001).

### Acoraceae (Sweet-Flag family)

#### 1. *Acorus* L.

##### Acorus calamus

Taxon classificationPlantaeAcoralesAcoraceae

L.

###### Names.

**Myanmar**: *lin-ne*, *lin-lay*. **English**: calamus, flagroot, sweet flag.

###### Range.

Northern Hemisphere. Temperate and tropical Asia; found growing around ponds and streams in cool climates. In Myanmar, grows wild and is also cultivated for use in home medicinal remedies.

###### Conservation status.

Least Concern [LC] (IUCN 2017).

###### Uses.

Of the two varieties of this species, the larger is used in traditional medicines. *Rhizome*: Preparations made from the rhizome are used to promote urinary flow, relieve constipation, and cleanse impurities from the body. The stewed rhizome is given for fever, coughs, and poisoning. A mixture of the rhizome that has been roasted until charred with oil is used as a rub applied topically to ease stomachaches and bloating in children. A mixture of the rhizome with cashew oil is used as a rub to relieve swollen joints and sore muscles. A mixture of equal amounts of the dried rhizome with *samone hpyu* (*Trachyspermum ammi*) is burned to create smoke for inhaling as a cure for hemorrhoids. The rhizome powder is taken with warm milk for sore throat. A mixture of the rhizome with *hsay-khar-gyi* (*Andrographis paniculata*) is given to reduce fever. To expel worms, a mixture of equal amounts of the rhizome with baked *shein-kho* (*Gardenia resinifera*) is given to children. A mixture of the rhizome powder with dried ginger powder and honey is taken for partial paralysis of the mouth, chin, and cheek. A mixture of the rhizome powder with honey is licked as a cure for epilepsy and to treat loss of sanity.

###### Notes.

The medicinal uses of his species in India are discussed in Jain and DeFilipps (1991). Medicinal uses of this species in China are discussed in Duke and Ayensu (1985).

###### Reference.

Agricultural Corporation (1980).

### Adoxaceae (Moschatel family)

#### 1. *Sambucus* L.

##### Sambucus javanica

Taxon classificationPlantaeDipsacalesAdoxaceae

Blume

###### Names.

**Myanmar**: *pale-ban*. **English**: Chinese elder, elderberry, Javanese elderberry.

###### Range.

Japan, Taiwan, southeastern Asia, Malaysian Archipelago. In Myanmar found in Chin, Kachin, Sagaing, and Shan.

###### Uses.

*Leaf, Flower*: Diuretic, purgative.

###### Notes.

Medicinal uses of this species in China are discussed in Duke and Ayensu (1985). Here the whole plant is decocted for ague, bone ache, dropsy, spasms, swellings, and traumatic injuries; the leaf is used for pain and numbness, diseases of bones, and rheumatic problems; the fruit is employed as a depurative and purgative, and a decoction is used for injuries, skin disease, and swelling; the root is used for numbness, pain, rheumatic difficulties, and bone diseases.

###### Reference.

Nordal (1963).

### Altingiaceae (Sweet-gum family)

#### 1. *Altingia* Noronha

##### Altingia excelsa

Taxon classificationPlantaeSaxifragalesAltingiaceae

Noronha

###### Names.

**Myanmar**: *nantayok*. **English**: Burmese storax.

###### Range.

India and Myanmar to Java; also cultivated. In Myanmar, it is found in Kachin and Taninthayi.

###### Use.

*Stem*: Resin used as remedy for orchitus.

###### Notes.

In India the resin is used on leucoderma and scabies; also for an antiscorbutic, carminative, stomachic, and expectorant (Jain and DeFilipps 1991). In China it is used as a tonic, and liquid storax is used in a tonic and stimulant, considered especially good for chest complaints. On the Malay Peninsula it is mixed with other drugs, and used as a tonic. In Indonesia the natives use the leaves for medicinal purposes (Perry 1980).

Reported constituents include essential oil, vanilline, cinnamic acid, styrolene, naphthalene, and caoutchouc (Perry 1980).

###### Reference.

Perry (1980).

### Amaranthaceae (Cockscomb family)

#### 1. *Achyranthes* L.

##### Achyranthes aspera

Taxon classificationPlantaeCaryophyllalesAmaranthaceae

L.

###### Names.

**Myanmar**: *kyet-mauk-pyan*, *kyet-mauk-sue-pyan*, *naukpo*. **English**: devil’s horsewhip, prickly chaff.

###### Range.

China, Taiwan, Bhutan, Cambodia, India, Indonesia, Laos, Malaysia, Myanmar, Nepal, the Philippines, Sikkim, Sri Lanka, Thailand, and Vietnam. In Myanmar, found in Magway and Yangon.

###### Uses.

*Leaf, Flowering Spike, Seed*: Used as an emetic and antiasthmatic.

###### Notes.

The medicinal uses of this species in India are discussed in Jain and DeFilipps (1991) as follows: The whole plant is used for cough; an infusion of the leaf in alcohol is used for leucoderma; leaf also used as an antidote for snakebite. The seed is emetic for hydrophobia. The root (applied with the roots of *Heteropogon contortus*) is used for caries of teeth, atrophy, emaciation, cachexy (mixed with roots of three other species); rheumatism (ground with roots of *Solanum surattense* and pills of this mixture smoked), strangulation of the intestine (ground with the roots of *Randia uliginosa*, betel (*Piper betle*) leaf and catechu, mixed with spirit, and administered); scabies (with other ingredients); syphilis sores (cooked with in oil with fruit of *Datura* and applied); childbirth complaints (ground with flowers of *Artocarpus heterophyllus*); tiger and snakebite; diuretic; abortifcient, stops bleeding after abortion; bark of root use for malarial fever.

###### Reference.

Nordal (1963).

#### 2. *Aerva* Forssk.

##### Aerva javanica

Taxon classificationPlantaeCaryophyllalesAmaranthaceae

(Burm.f.) Juss. ex Schult. (= Aerva persica (Burm.f.) Merr.)

###### Names.

**Myanmar**: *on-hnye*. **English**: aerva, kapok bush, snow bush.

###### Range.

Widespread in drier parts of the tropics and subtropics of the Old World, from Myanmar, India and Sri Lanka westwards through Southwest Asia, across North Africa to Morocco and south to Cape Verde island and Cameroun Uganda and Tanzania to Madagascar. Introduced in Australia and elsewhere.

###### Use.

*Root*: Paste made and applied to acne-like conditions of the face.

###### Notes.

The species is used as a uricant (Burkill 1985); also to treat kidney stones and for inflammation (Zafar et al. 2006). The medicinal uses of another member of the genus *Aerva* in India are discussed in Jain and DeFilipps (1991) as follows: The whole plant is used for albumin in the urine; infant diarrhea; cholera; and dysentery. The leaf is used for earache; and the root is used for snakebite.

###### Reference.

Perry (1980).

#### 3. *Alternanthera* Forssk.

##### Alternanthera sessilis

Taxon classificationPlantaeCaryophyllalesAmaranthaceae

(L.) R.Br. ex DC.

###### Names.

**Myanmar**: *pazun-sar*, *pazun-za*. **English**: dwarf copperleaf, joyweed, sessile joyweed.

###### Range.

Native range Australia, Northen Mariana Islands, Federated States of Micronesia, Guam, Palau, the Philippines, Soloman Islands, and Singapore. Now very widespread in the tropics and subtropics of both the Old and New Worlds, especially in damp or wet locations. In Myanmar, found in Yangon.

###### Conservation status.

Least Concern [LC] (IUCN 2017).

###### Use.

*Leaf*, *Juice*: Used as a galactagogue.

###### Notes.

The medicinal uses of this species in India are discussed in Jain and DeFilipps (1991) as follows: The root is used for hazy vision and night blindness (in combination with four other species); postnatal complaints (ground with seeds of two other species and roots of a third); prolapsus and fistula ani (roots and leaves mixed with rice and salt); diarrhea (roots, bark, and fruit pulp of three other plants and some lime from shells); fever with intense thirst (in combination with other components); dog, jackal and lizard bite (with other plants); also, an unspecified plant part is used for dysentery. In China a broth of the plant is cooked with meat and taken for tuberculosis; a decoction with wine is used for internal injuries (Duke and Ayensu 1985).

###### Reference.

Nordal (1963).

#### 4. *Amaranthus* L.

##### Amaranthus cruentus

Taxon classificationPlantaeCaryophyllalesAmaranthaceae

L. (= Amaranthus paniculatus L.)

###### Names.

**Myanmar**: *hin-nu-nwe*. **English**: prince’s feather, purple amaranthus, red amaranthus, spiny amaranthus.

###### Range.

Original habitat is obscure, probably tropical America. Thought to have originated from *A. hybridus* (most probably in cultivation in Central America); also found in North and South America. As a spontaneous weed it occurs in Asia eastward from Malaya (Indonesia, New Guinea, the Philippines, etc.) and in tropical Africa. It is found throughout the warmer regions of the word as an ornamental, and in some regions it is grown as a grain crop. Reported from Myanmar.

###### Uses.

*Leaf*, *Seed*: Used as laxative, blood purifier, diuretic, and soporific.

###### Note.

In India the root of the species is used for dropsy (Jain and DeFilipps 1991).

###### Reference.

Nordal (1963).

##### Amaranthus spinosus

Taxon classificationPlantaeCaryophyllalesAmaranthaceae

L.

###### Names.

**Myanmar**: *hin-nu-new-subauk*, *khar-grope* (Mon). **English**: pigweed, soldier-weed, spiny amaranthus, spiny pigweed, thorny amaranthus.

###### Range.

Pantropical.

###### Uses.

*Whole plant*: Leaves, roots, and whole plant used as a laxative, blood purifier, diuretic, and soporific. Taking the crushed and squeezed juice from the plant will neutralize the venom in snake bites. Boiling the plant and taking it will keep help prevent miscarriages. *Leaf*: Cure nose bleeds. Eating the leaves cooked in a curry will cure pain in urination and kidney stones. Juice squeezed from leaves can be licked with honey to cure vomiting and passing of blood, excessive menstruation, white vaginal discharge, gonorrhea, and sores and bumps. *Root*: The paste of the root made with water will neutralize the poison if applied to the site of a scorpion sting. It can also be applied onto boils to cure them. Applying either the paste of the root or using the crushed root as a poultice will cure stiffness of the muscles. The paste made with water can be strained and taken once in the morning and once at night to cure excessive menstruation.

###### Notes.

Jain and DeFilipps (1991) discuss the medicinal uses of the species in India, including use of the root as a laxative and abortifacient, and use of the leaf as a laxative. Medicinal use of this species in China is discussed by Duke and Ayensu (1985).

###### References.

Nordal (1963), Agricultural Corporation (1980).

#### 5. *Celosia* L.

##### Celosia argentea

Taxon classificationPlantaeCaryophyllalesAmaranthaceae

L. (= Celosia cristata L.)

###### Names.

**Myanmar**: *kyet-mauk*. **English**: cock’s comb, crested cock’s comb, silver cock’s comb, wild cock’s comb.

###### Range.

Widely distributed in tropics; a common weed. Found in China, Bhutan, Cambodia, Japan, Korea, India, Laos, Malaysia, Myanmar, Nepal, the Philippines, Russia, Sikkim, Thailand, Vietnam; also tropical Africa. Widely distributed in Myanmar.

###### Uses.

*Leaf*, *Flower*, and *Seed*: Used as antipyretic, aphrodisiac, and vulnerary.

###### Notes.

In India the seed is used for eye diseases, clearing the eyes, to treat mouth sores and blood diseases, as an aphrodisiac, and for diarrhea (Jain and DeFilipps 1991). Medicinal uses of this species is China are discussed in Duke and Ayensu (1985). Here the flowers are used for hemoptysis, metrorhagia, dysentery, hemoptysis, hemorrhoids, leucorrhea, menorrhagia; the stem for a poultice on sores, skin eruptions, swellings, and boils; the seed for diarrhea, painful micturiton, cough, dysentery; and for opthalmia. The Chinese also poultice the seeds over broken bones and use the seed and herb as an anthelmintic an vermifuge. The whole plant is used for eye and liver ailments.

###### Reference.

Nordal (1963).

#### 6. *Chenopodium* L.

##### Chenopodium album

Taxon classificationPlantaeCaryophyllalesAmaranthaceae

L.

###### Names.

**Myanmar**: *myu.* **English**: goosefoot, lambsquarters, pigweed.

###### Range.

Europe, Asia, North America. Cultivated in Myanmar.

###### Uses.

*Root*: Paste used to treat diarrhea in children.

###### Notes.

In India the seed is used to treat skin diseases (Jain and DeFilipps 1991). In China juice from the stem is applied to freckles and sunburn; leaves are applied to insect bites, sunstroke, and as a wash for swollen feet; a decoction is used as a rinse for carious teeth (Duke and Ayensu 1985). In China, in addition to the uses of juice from the fresh plant previously mentioned, the seeds are eaten as an anthelmintic. In Indo-China the plant is used to treat blennorrhea in women (Perry 1980).

Reported chemical constituents include betaine, leucien, and essential oil (Perry 1980).

###### Reference.

Perry (1980).

#### 7. *Dysphania* R.Br.

##### Dysphania ambrosioides

Taxon classificationPlantaeCaryophyllalesAmaranthaceae

(L.) Mosyakin & Clemants (= Chenopodium ambrosioides L.)

###### Names.

**Myanmar**: *say-my*. **English**: Mexican tea, strong-scented pigweed, wormseed.

###### Range.

Tropical America. Cultivated in Myanmar.

###### Uses.

*Whole plant*: Used as an anthelmintic, especially for roundworms but also for hookworms, as well as a remedy for intestinal amoebae.

###### Notes.

Medicinal uses of this species in India are discussed in Jain and DeFilipps (1991). Perry (1980) discusses the medicinal uses of the species in general, and also gives its uses in Japan, Indo-China, and the Philippines. Medicinal use, chemical constituents, pharmacological action, and of this species in Indian Ayurveda are discussed in detail by Kapoor (1990).

Reported chemical constituents of the plant include volatile oil, ascaridol, geraniol, saponin, 1-limonene, p-cymene, and d-camphor (Perry 1980). The medicinal uses of this plant in the Caribbean region, as well as its chemistry, biological activity, toxicity and dosages, are discussed by Germosén-Robineau (1997). The chemistry, pharmacology, history and medicinal uses of this species in Latin America are discussed in detail by Gupta (1995). Details of the active chemical compounds, effects, herbal usage and pharmacological literature of this plant are given in Fleming (2000). Worldwide medicinal usage, chemical composition, and toxicity of this species are discussed by Duke (1986).

###### Reference.

Nordal (1963).

### Amaryllidaceae (Amaryllis family)

#### 1. *Allium* L.

##### Allium cepa

Taxon classificationPlantaeAsparagalesAmaryllidaceae

L.

###### Names.

**Myanmar**: *kyet-thun-ni oo-gyi*, *shakau* (Kachin), *kaisun* (Chin), *canone casaun* (Mon). **English**: garden onion, onion.

###### Range.

Original range unknown; now only known in cultivation. Cultivated in all parts of Myanmar with the exception of the extremely cold regions.

###### Uses.

Root (Bulb): Used in the treatment of flatulence, dysentery, and as a stimulant, diuretic and expectorant. Sweet and hot with some heating and diuretic properties, the onion is used to control flatulence, phlegm, fever and cough. It is also used to relieve nausea, stimulate the appetite, and fortify semen. Adults eat onion bulbs raw to alleviate urine blockages, but children with the same condition have roasted bulbs applied while still warm over the body area near the bladder. Children also drink onion juice mixed with sugar and chilled as a sherbet drink for diarrhea and infections that cause burning during urination. Mixed with a bit of sugar, half a tablespoon of fresh onion juice is ingested to treat bleeding hemorrhoids. Mixed with a bit of salt, onion juice is applied as eyedrops to alleviate night-blindness. For ear infections, either the warm juice of roasted onions or the juice of unroasted onions are used as eardrops. The milky liquid from cut onions, mixed with edible lime, is applied to scorpion sting to neutralize the venom. The onion is also used in mixtures to treat trembling and weakness in men (illness not specified in Agriculture Corporation 1980), thinness and weakness in women (illness not specified in Agriculture Corporation 1980), pain from flatulence, and illnesses that cause chest pain. *Seed*: To increase vitality, onion seeds are crushed and ingested.

###### Notes.

Medicinal uses of this species in India are discussed in Jain and DeFilipps (1991). Chemical constituents, pharmacological action, and medicinal use of this species in Indian Ayurveda are discussed in detail by Kapoor (1990).

Details of the active chemical compounds, effects, herbal usage and pharmacological literature of this plant are given in Fleming (2000). Toxicity of this species is discussed by Bruneton (1999). Traditional medicinal uses, chemical constituents and pharmacological activity of this species are discussed by Ross (2001). An extract of the dried plant was found to have a potent and prolonged hypoclycemic effect on artificially induced diabetes in rats and rabbits.

###### References.

Mya Bwin and Sein Gwan (1967), Agricultural Corporation (1980).

##### Allium sativum

Taxon classificationPlantaeAsparagalesAmaryllidaceae

L.

###### Names.

**Myanmar**: *kyet-thun hpyu*, *casaun-phet-tine*. **English**: garlic.

###### Range.

Central Asia. In Myanmar, grown mostly in Shan State as a cultivated plant.

###### Uses.

Root (Bulb): Garlic is used to support blood and eye health, alleviate fevers and skin disorders, increase perspiration and semen production, stimulate the bowel and the bladder, and to promote virility and longevity. A half teaspoon of garlic powder, steeped in honey and taken at bedtime, is used as a vitalizing tonic to stimulate appetite and promote healthy sleep. It is used to break up phlegm, as well as to strengthen the blood and the gall bladder. Sap from cut garlic bulbs is a remedy for skin conditions, including ringworm, scabies, eczema, freckles and similar facial skin discolorations. Garlic milk, made by boiling seven large bulbs in 40 ticals (ca. 0.5 kg) of pure milk, cooling the mixture for about 10 minutes, and boiling it a second time, is ingested daily for hypertension. A teaspoon of garlic juice mixed with a bit of water and sugar is used to treat whooping cough; garlic juice is taken for coughs, bloated stomachs, and sores on the stomach. To alleviate flatulence, garlic is soaked in sesame oil with a bit of salt and ingested before meals. Infants are given single roasted garlic bulbs for colic and indigestion. For goiter, two drops of garlic oil are applied to the throat, as well as ingested three times a day. Garlic juice mixed with salt is consumed or rubbed at the temples as a remedy for headaches. Because of its germicidal properties, garlic is used to treat lung problems, deep wounds and sores; its juice is also rubbed on the body to ease aches and pains. A mixture consisting of two cloves of garlic boiled in sesame oil is poured warm into the ear as a remedy for deafness, infections, and aches. Garlic is a component of medicines that treat incompletely healed wounds, irregular menstruation, and various malaises (term used where cause of illness not specified in Agriculture Corporation 1980) of men.

###### Notes.

Medicinal uses of this species in India are discussed in Jain and DeFilipps (1991). Chemical constituents, pharmacological action, and medicinal use of this species in Indian Ayurveda are discussed in detail by Kapoor (1990).

The medicinal uses of garlic in the Caribbean region, as well as its chemistry, biological activity, toxicity and dosages, are discussed by Germosén-Robineau (1997). The chemical constituents, pharmacological activities, and traditional medicinal uses of garlic on a worldwide basis are discussed in detail by Ross (1999). A pharmacognostical profile including medicinal uses of this species in Africa is given in Iwu (1993). Details of the active chemical compounds, effects, herbal usage and pharmacological literature of garlic are given in Fleming (2000). A detailed discussion of garlic, i.e., natural history, association with humanity, antiherbivory and insect defenses, and medicinal uses (antibiotic and antitoxin actions, cholesterol regulation), is found in Kaufman et al. (1999).

Garlic prolongs elasticity of the aorta (Leigh 1998), resulting in healthy functioning of the cardiovascular system. Garlic also has antimicrobial effects on *Candida albicans* and *Coccidioides immitis*; fresh garlic juice lowers cholesterol and triglycerides in the blood, which helps to prevent blood clotting and thus heart attack and strokes; garlic has free radical scavenging activity which amplifies the bodily antioxidant system; and, garlic lowers concentrations of nitrates, the precursors of the carcinogen nitrosamine, in the gastric juice of the stomach and provides protection against the development of stomach cancer (Lau 1996).

###### Reference.

Agricultural Corporation (1980).

#### 2. *Crinum* L.

##### Crinum asiaticum

Taxon classificationPlantaeAsparagalesAmaryllidaceae

L.

###### Names.

Myanmar: *koyan-gyi*. **English**: poison bulb, tree crinum.

###### Range.

Tropical Asia. Found in the warmer regions of Myanmar, growing naturally as well as under cultivation.

###### Uses.

*Leaf*: Boiled and used as a bath, or the juice applied as a thick liquid to treat edema. The leaves are wilted over hot charcoal and wrapped around the knees for swollen knees, or placed on the back for about one hour for backaches. *Leaf* and *Bulb*: Used to neutralize poisons and regulate flatulence, phlegm, and urine. *Bulb*: Ground (on a stone) to make a paste for reducing the heat from swellings or for weeping sores (this paste, however, causes some itching). For instances of poisoning, it is enough to rub the tongue with the bulb, which is also used as a special ingredient in *shar-put-hsay* (a commonly used form of traditional medicine consisting of a grayish brown powder roughly rolled into little nuggets rolled around the tongue until dissolve into its components).

###### Notes.

The medicinal uses of this species in India are discussed in Jain and DeFilipps (1991). Medicinal uses of this species in China are discussed in Duke and Ayensu (1985).

###### References.

Agricultural Corporation (1980), Forest Department (1999).

### Anacardiaceae (Cashew family)

#### 1. *Anacardium* L.

##### Anacardium occidentale

Taxon classificationPlantaeSapindalesAnacardiaceae

L. (= Acajuba occidentalis (L.) Gaertn.; Anacardium microcarpum Ducke)

###### Names.

**Myanmar**: *thiho-thayet*, *shitkale*, *mak-mong-sang-yip*. **English**: cashew nut.

###### Range.

Tropical America. Probably originating in Brazil. Cultivated in Myanmar.

###### Uses.

*Bark*: A restorative. *Bark, Leaf, Fruit*: Used as an anthelmintic, also for leucoderma and other skin diseases as well as for diabetes. *Fruit*: The kernel (nut) is a pain reliever.

###### Notes.

Medicinal uses of this species in India are discussed in Jain and DeFilipps (1991). Indigenous medicinal uses of this species in the Andaman and Nicobar Islands (India) are described by Dagar and Singh (1999). Medicinal uses of this species in China are discussed by Duke and Ayensu (1985).

The cashew nut, a true fruit, is rich in lipids, glucosides, calcium, phosphorus and vitamin B. It further yields a fair amount of protein, mineral salts, iron and fiber. The oil is a laxative and acts powerfully against intestinal worms; it is also excellent for use to treat premature aging of the skin. The irritating oil obtained after soaking the nuts in water is viscous-brown and contains 90% anacardic acid and 10% cardol which exhibits potent antibacterial activity against Gram positive bacteria. It is also used to treat sores, warts, ringworm and psoriasis (Beauvoir et al. 2001).

Used in cosmetics, the juice contains substances capable of capturing free radicals. It has value for hair conditioning due to its proteins and mucilage. Therefore it is an excellent scalp conditioner and tonic used for making lotions and scalp creams. The enlarged receptacle (cashew apple) with a waxy skin provides vitamins A, B, and C, a few amino acids, calcium and iron. It exhibits strong potential activity against Gram positive bacteria and somewhat less antifungal activity against molds. The juice made from the cashew apple cures influenza (Beauvoir et al. 2001). “Ingestion of raw cashew nuts can cause eczematous dermatitis that is generalized but especially severe on the palms” of the hands (Benezra et al. 1985).

The chemistry, pharmacology, history and medicinal uses of this species in Latin America are discussed in detail by Gupta (1995). The toxic properties, symptoms, treatment and beneficial uses of this plant, parts of which are poisonous, are discussed by Nellis (1997). Data on the propagation, seed treatment and agricultural management of this species are given by Katende et al. (1995).

The receptacle (pseudo-fruit) contains vitamin C; the main phenolic components of the oil from the shells are anacardic acid and cardol, which have antibacterial, molluscicidal and anthelminic properties; the inner bark has hypoglycemic action; tannins in the bark have anti-inflammatory properties; and, the essential oil of the leaves, which is comprised almost exclusively of alpha-pinene, acts as a depressant on the central nervous system (Mors et al. 2000). Details of the active chemical compounds, effects, herbal usage and pharmacological literature of this plant are given in Fleming (2000). Traditional medicinal uses, chemical constituents and pharmacological activity of this species are discussed by Ross (2001).

The seed of *Anacardium occidentale* contain anacardic acid which causes skin pustules or rashes, and also contains bilobol, which has antitumor activity (Lan et al. 1998).

###### References.

Nordal (1963), Perry (1980).

#### 2. *Buchanania* Spreng.

##### Buchanania lancifolia

Taxon classificationPlantaeSapindalesAnacardiaceae

Roxb.

###### Names.

**Myanmar**: *taung-thayet*, *thayet-thin-baung*, *thingbaung*. **English**: cheerojee-oil plant, chirauli nut.

###### Range.

China, India, Laos, Malaysia (peninsular), Myanmar, Nepal, Singapore, Thailand, Vietnam. In Myanmar, found in Rakine and Yangon.

###### Uses.

*Leaf, Seed, Root*: Used as laxative. *Seed*: Oil used as a substitute for almond oil.

###### Notes.

According to the *Materia Medica* (Latin translation of the Greek Pedanius Dioscorides’ famous 5-volume book, considered a precursor to all modern pharmacopeias), this species is used in combination with others (*Shorea robusta*, *Terminalia tomentosa*, and *Acacia catechu*) to soak extract of silajátu, a dark sticky unctuous substance (term applied to bituminous substances said to exude from certain rocks during hot weather; said to be produced in the Vindhya and other mountains where iron is abundant), which has been dried in the sun, to purify extract for use as tonic to treat urinary disease, diabetes, gravel, anemia, tuberculosis, cough, and skin diseases.

###### Reference.

Nordal (1963).

#### 3. *Lannea* A.Rich.

##### Lannea coromandelica

Taxon classificationPlantaeSapindalesAnacardiaceae

(Houtt.) Merr. (= Lannea grandis Engl.)

###### Names.

**Myanmar**: *latang*, *laupe*, *mai-hkam*, *nabe*, *taung-gwe*, *zun-burr*. **English**: jail, jhingam, jhingam poma, moi, monia, poma, wodier.

###### Range.

Sub-Himalayan tract to India, Myanmar, Assam, Sri Lanka, and the Andaman Islands; cultivated elsewhere in continental Southeast Asia. In Myanmar, found in Bago, Kayin, Mandalay, Rakhine, Shan, Taninthayi, and Yangon.

###### Uses.

*Bark*, *Gum*: Used as an astringent. *Leaf*, *Juice*: Used for local swellings and body pain.

###### Notes.

Reported medicinal uses of the species include: Antidote and astringent; for bruises, carbuncles, sores, swelling, and wounds; also for cholera, convulsion, diarrhea, dysentery, elephantiasis, hematuria; and rinderpest (Duke 2009).

###### Reference.

Nordal (1963).

#### 4. *Mangifera* L.

##### Mangifera indica

Taxon classificationPlantaeSapindalesAnacardiaceae

L. (= Mangifera austroyunnanensis Hu; Rhus laurina Nutt.)

###### Names.

**Myanmar**: *krek*, *kruk*, *la-mung*, *mak-mong*, *ma-monton*, *mamung*, *sagyaw*, *shagyaw*, *takau*, *thayet*, *thayet-phyu*, *umung*. **English**: mango.

###### Range.

Tropical Asia. Widely distributed in Myanmar.

###### Conservation status.

Data Deficient [DD] (IUCN 2017).

###### Uses.

*Bark*: Used as an astringent. *Fruit*: Ripe fruit used as laxative and rind used as tonic. *Seed*: Employed as an antiasthmatic.

###### Notes.

Medicinal uses of this species in India are discussed in Jain and DeFilipps (1991). Indigenous medicinal uses of this species in the Andaman and Nicobar Islands (India) are described by Dagar and Singh (1999). Medicinal uses of this species in China are discussed by Duke and Ayensu (1985).

Benezra et al. (1985) noted that: “People eating the fruit may suffer erythemato- vesicular eruptions of the lips and the entire face and neck…and sometimes the genitals. The peel, not the juice, seems to be responsible”; such dermatitis is known as “mango poisoning.”

The chemical constituents, pharmacological activities, and traditional medicinal uses of this plant on a worldwide basis are discussed in detail by Ross (1999). The toxic properties, symptoms, treatment and beneficial uses of this plant, parts of which are *poisonous*, are discussed by Nellis (1997). Data on the propagation, seed treatment, and agricultural management of this species are given by Katende et al. (1995) and Bekele-Tesemma (1993). Uses of this plant in the Upper Amazon region, where some Amerindian tribes use a brew of the leaves as a contraceptive and abortifacient, are given by Castner et al. (1998). All parts of the *Mangifera indica* plant contain resorcinol, an irritant to the mouth and tongue (Lan et al. 1998).

###### References.

Nordal (1963), Perry (1980).

#### 5. *Rhus* L.

##### Rhus chinensis

Taxon classificationPlantaeSapindalesAnacardiaceae

Mill. (= Rhus semialata Murray)

###### Names.

**Myanmar**: *chying-ma*, *mai-kokkyi*, *mai-kokkyin*. **English**: nutgall tree.

###### Range.

Temperate eastern Asia. In Myanmar, found in Chin, Kachin, Mandalay, Mon, Sagaing, and Shan.

###### Uses.

*Fruit*: Used to treat colic. *Galls*: Used as astringent.

###### Notes.

In India the flower buds are used for diarrhea; the fruit for stomachache; and the seed for stomachache and as a purgative, also on skin diseases (Jain and DeFilipps 1991). Duke and Ayensu (1985) discuss the uses of the the bark, leaf, and root bark of this species in China, as well as those of the whole plant.

The chemical constituents of the species include gallic acid and penta-m-digalloyl-beta-glucose (Duke and Ayensu 1985).

###### Reference.

Nordal (1963).

#### 6. *Semecarpus* L.f.

##### Semecarpus anacardium

Taxon classificationPlantaeSapindalesAnacardiaceae

L.f. (= Semecarpus heterophyllus Bl.; Semecarpus albescens (non Kurz) K. & V.; Semecarpus cinerea H.H.W. Pearson; Semecarpus glabrescens Heine; Melanochyla tomentosa (non Hook.f.) Engl.)

###### Names.

**Myanmar**: *che*, *chay-thee pin*, *thitsi-bo*, *mai-ka-aung* (Shan). **English**: markingnut tree, varnishtree.

###### Range.

Tropical Asia. Reported from Myanmar.

###### Uses.

Sweet and astringent, *Semecarpus anacardium* has heating properties that regulate bowels, aid digestion, control phlegm and respiratory function, heal sores, alleviate leprosy, and reduce hemorrhoids, bloating, and fevers. *Bark*: Used as an astringent. *Fruit*: Serves as a laxative. *Fruit*: Can be crushed together with lime (the chemical) as a poultice to heal sores. Three drops of the oily sap released by the heated fruit can be taken with milk for coughing. Children can be given just two drops of this sap twice a day to alleviate phlegm and coughing. Crushed fruit can be applied to joints to relieve inflammation. An ointment of the fruit mixed with resin from the “*in*” tree (*Dipterocarpus tuberculatus*) cooked with sesame oil can be used to treat rashes, itches, and cracks on the heels and soles of the feet. A paste of ground fruit and sesame oil remedies ringworm. The fruit is also used in medicines for motor paralysis and joint inflammation. The rind is used as a tonic. *Seed*: Used as an antiasthmatic, also to treat leprosy. Note: The fruit is included in the list of *toxic plants* and, therefore, should be used only after preparing systematically.

###### Note.

In India, the resin of this species is used for leprosy, nervous debility, skin diseases; and the fruit oil is used on warts and tumors; on cuts, sprains, piles, injuries; and for ascites, rheumatism, asthma, neuralgia, dyspepsia, epilepsy, psoriasis (Jain and DeFilipps 1991).

###### References.

Agricultural Corporation (1980), Perry (1980).

#### 7. *Spondias* L.

##### Spondias pinnata

Taxon classificationPlantaeSapindalesAnacardiaceae

(L.f.) Kurz (= Spondias magifera Willd.)

###### Names.

**Myanmar**: *bwe-baung*, *ding-kok*, *gwe*, *hpunnam-makawk*, *mai-kawk*, *mai-mak-kawk*. **English**: hog plum, wild mango.

###### Range.

Thought probably native to Indonesia and the Philippines; found in China, sub-Himalayan tract from Chenab eastwards; widely cultivated and naturalized in Bhutan, Cambodia, India, Indonesia, Laos, Malaysia (peninsular), Myanmar, Nepal, the Philippines, Singapore, Thailand, and Vietnam. Reported from Myanmar.

###### Uses.

*Bark*: Used for dysentery. *Fruit*: Used as antiscorbutic; considered a remedy for dyspepsia.

###### Notes.

The medicinal uses of this species in India are discussed in Jain and DeFilipps (1991) as follows: The bark is used for stomachache and as a refrigerant; the fruit as an astringent, antiscorbutic, and for bilious dyspepsia; and the root for regulating menstruation. Perry (1980) also discusses the medicinal uses of this species in Indo-China, the Malay Peninsula, and Indonesia.

###### References.

Nordal (1963), Perry (1980).

### Annonaceae (Soursop family)

#### 1. *Annona* L.

##### Annona squamosa

Taxon classificationPlantaeMagnolialesAnnonaceae

L.

###### Names.

**Myanmar**: *awzar*, *awsa* (Kachin), *azat* (Chin), *sot-maroat* (Mon), *mai-awza* (Shan). **English**: custard apple, sugar apple, sweetsop.

###### Range.

New World tropics. In Myanmar, originally a cultivar primarily of the central region; now found growing wild all over the country.

###### Uses.

*Whole plant*: Flowers, bark, leaves, fruit, seed, and root support vascular, respiratory, digestive, and excretory functioning, as well as alleviating fever symptoms and fever-related disorders. *Bark*: Tonic from the bark ingested for strength. *Leaf*: Crushed and consumed to expel intestinal worms, particularly threadworms; applied externally as a poultice for stiff, sore muscles; and the vapors from crushed leaves inhaled to ease dizziness and sinusitis. *Flower* and *Fruit*: Soups made from the flowers and the young fruit, combined with other ingredients, such as goat testes, pork, and/or beef, used to restore sexual functioning, strength, alertness, and wellbeing. *Fruit*: With binding properties, the green fruits are used to alleviate diarrhea, dysentery, and loose bowels. *Seed*: Pulverized into a powder and applied to sores as an antiseptic. Inhalation of the smoke from crushed and burned seeds provides an epilepsy treatment. *Root*: Consumption of root paste clears urinary infection and improves urinary functioning.

###### Notes.

Medicinal uses of this species in India are discussed in Jain and DeFilipps (1991). Pharmacognostic characters and Thai ethnomedical use of this species are discussed in Somanabandhu et al. (1986). Chemical constituents, pharmacological action, and medicinal use of this species in Indian Ayurveda are discussed in detail by Kapoor (1990). Indigenous medicinal uses of this species in the Andaman and Nicobar Islands (India) are described by Dagar and Singh (1999). The chemistry, pharmacology, history, and medicinal uses of this species in Latin America are discussed in detail by Gupta (1995). The seeds have post-coital anti-fertility activity; the most abundant free amino acids in the fruit pulp are L(+)citrulline, L(+)arginine, L(+)ornithine and GABA (gamma-aminobutyric acid); and, the predominant constituent of the essential oil from the bark is aromadendrene (Mors et al. 2000).

Data on the propagation, seed treatment, and agricultural management of this species are given by Katende et al. (1995).

###### Reference.

Agricultural Corporation (1980).

#### 2. *Artabotrys* R.Br.

##### Artabotrys hexapetalus

Taxon classificationPlantaeMagnolialesAnnonaceae

(L.f.) Bhandari (= Artabotrys odoratissimus R.Br.)

###### Names.

**Myanmar**: *kadat-ngan*, *padat-nygan*, *tadaing-hmwe*. **English**: climbing ylang-ylang.

###### Range.

Sri Lanka and southern India; cultivated widely in the tropics. Widely distributed in Myanmar.

###### Use.

*Leaf*: Used in cholera. (*Flower*: Used in perfumery).

###### Notes.

As a Chinese folk medicine, its root and fruit are used to treat malaria and scrofula.

Leaf extracts of this species are used for antifertility; flowers for a stimulating tea-like beverage and also to extact essential oil used in perfume. Fruits are eaten by indigenous people to maintain their health. Additional medicinal uses of this species include as an antifungal, cardiac depressor, for cholera, and as a hypotensive and weak estrogenic (Manjula et al. 2011).

###### Reference.

Nordal (1963).

#### 3. *Cananga* (DC.) Hook.f. & Thomson

##### Cananga odorata

Taxon classificationPlantaeMagnolialesAnnonaceae

(Lam.) Hook.f. & Thomson (= Cananga odoratum (Lam.) King)

###### Names.

**Myanmar**: *kadat-ngan*, *saga-sein*, ylang-ylang. **English**: cananga.

###### Range.

Southeast Asia.

###### Uses.

Plant contains antibacterial, antifungal, and cytotoxic compounds used in treatments for eye conditions, as well as for malaria, gout, and headache. *Flower*: Used in ophthalmia.

###### Notes.

Medicinal uses of this species in India are discussed in Jain and DeFilipps (1991). Perry (1980) discusses the uses of this species in other parts of Asia as follows: On the Malay Peninsula, a paste made from fresh flowers is prescribed to treat asthma and leaves rubbed on the skin are used as a remedy for itch; in Indonesia, the bark is used to treat scabies, dried flowers are used to treat malaria, and the seeds finely ground with other ingredients are applied to treat stomach disorders in intermittent fever; in the Solomon Islands, crushed leaves are applied to boils. Worldwide medicinal usage, chemical composition, and toxicity of this species are discussed by Duke (1986).

Steam-distilled flower petals are the source of the perfume oil known as “ylang-ylang”, made in Asia, Madagascar and the Mascarenes. Perfumes, colognes, and toilet waters containing ylang ylang oil are responsible for several cases of allergic contact dermatitis in sensitive individuals. (Benezra 1985).

###### References.

Nordal (1963), Kirtikar and Basu (1993), Duke (2009), Rahman et al. (2005a).

#### 4. *Polyalthia* Blume

##### Polyalthia longifolia

Taxon classificationPlantaeMagnolialesAnnonaceae

(Sonn.) Thwaites

###### Names.

**Myanmar**: *arthaw-ka*, *lan-tama*, *thinbaw-te*. **English**: false ashoka, green champa, Indian fir tree, Indian mast tree.

###### Range.

Sri Lanka and southern India; cultivated in India, Malaya, Pakistan and Tropical East Africa. Cultivated in Myanmar.

###### Use.

*Bark*: Used as febrifuge.

###### Notes.

Significant antimicrobial and antifungal activity of clerodane diterpenoids has been found from the seeds of this species (Marthanda Murthy et al. 2005). Methanolic extracts have yielded 20 known and two new organic compounds, some of which show cytotoxic properties (Chen et al. 2000).

###### Reference.

Nordal (1963).

### Apiaceae (Carrot family)

#### 1. *Anethum* L.

##### Anethum graveolens

Taxon classificationPlantaeApialesApiaceae

L. (= Peucedanum graveolens (L.) Hiern.)

###### Names.

**Myanmar**: *sameik*, *samon nyo*. **English**: dill, European dill, Indian dill.

###### Range.

Indigenous to Mediterranean region, but adventive and cultivated worldwide in tropical and temperate climates. Grows naturally and is also cultivated in Upper Myanmar.

###### Uses.

*Fruit*, *Seed*: Used as carminative, stomachic, and spasmolytic. *Leaf*, *Seed*: Hot-tasting seeds and leaves contain heating properties used to stimulate circulation and gall bladder function, as well as to alleviate fever, inflammation, and congestion. *Seed*: A boiled-water extract of the seeds is reduced to one-third the starting volume and taken for chest discomfort, shooting pains, and aches. The same extract is given to new mothers as a tonic for the heart and as a postnatal restorative. The roasted seeds are eaten plain or with rock sugar to stimulate lactation. Brushed with oil and roasted over a fire, the leaves are pulverized into an ointment applied to sores to reduce inflammation.

###### Notes.

This is a common plant widely cultivated for use as an herb, and for its fruit which is used in medicine as an aromatic stimulant and carminative. The medicinal uses of this species in China are discussed in Duke and Ayensu (1985).

###### References.

Nordal (1963), Agricultural Corporation (1980).

#### 2. *Apium* L.

##### Apium graveolens

Taxon classificationPlantaeApialesApiaceae

L.

###### Names.

**Myanmar**: *samut*, *tayokenan-nan*, *kum-bomb-kroke* (Mon). **English**: celery, cultivated celery, marsh parsley, wild celery.

###### Range.

Eurasia and worldwide. Although found growing naturally, it is cultivated all over Myanmar for use as a vegetable.

###### Conservation status.

Least Concern [LC] (IUCN 2017).

###### Uses.

*Whole plant*: The watery extract of the whole plant mixed with sugar or honey is used as a remedy for hypertension. *Seed*: With heating properties, the easily digestible yet bitter, sharp-tasting seeds are used to support digestion, increase sperm, promote circulation, control blood pressure, ease inflammation in the breathing passages, alleviate nausea and vomiting, and treat whooping cough and dropsy. Juice from chewing- the seeds wrapped in betel (*Piper betle*) leaf, is held in the mouth to treat dry coughs and coughs with mucus; the seeds alone, is swallowed to stop hiccups. The powder from pulverized seeds mixed with clove buds is ingested to alleviate nausea. Seeds with roasted salt are eaten to cure stomachaches. Seeds mixed with jaggery are shaped into pellets and taken for indigestion, overeating, and stomach distention.

###### Notes.

The medicinal uses of this species in India are discussed in Jain and DeFilipps (1991). Medicinal use of this species in China is discussed by Duke and Ayensu (1985).

In Thailand, researchers have shown that the seed extract is an effective larvicide for the dengue fever mosquito vector, *Aedes aegypti* (Tuetun et al. 2005, Choochote et al. 2004).

###### Reference.

Agricultural Corporation (1980).

#### 3. *Centella* L.

##### Centella asiatica

Taxon classificationPlantaeApialesApiaceae

(L.) Urb. (= Hydrocotyle asiatica L.)

###### Names.

**Myanmar**: *myin-hkwa*, *myin-khwar pin*, *ranjneh hnah* (Chin), *hlahnip chai* (Mon). **English**: Indian pennywort.

###### Range.

Throughout tropical and some subtropical parts of world. Widely distributed in Myanmar, especially in the cooler regions, and found all year near the water’s edge. Although it grows wild, it is also widely cultivated as it is much used.

###### Conservation status.

Least Concern [LC] (IUCN 2017).

###### Uses.

*Whole plant*: Used to treat diabetes, and as a laxative and diuretic. *Leaf*: Has a sweet, bitter, sharp, hot taste. Used to control phlegm, treat skin diseases, itching, rashes, sores, and leprosy. The juice squeezed from the leaves- is drunk together with sugar and honey daily to give strength and vitality; mixed with an equal amount of kerosene and massaged into cysts that form on joints; 1 teaspoon given to children to treat colds, fevers, and it will also loosen the bowels; applying or taking it can cure skin diseases. For injuries, applying the juice will reduce the inflammation. The leaves can be made into a drink taken to treat dysentery and urine retention, painful urination, and blood in the urine. Eaten with pepper and honey, they promote health. The leaf is also used in compounds for tonics, poison neutralizers, to treat sores, and as a medicine for sore eyes. Leaves are dried and used as an herbal tea to alleviate hyper- tension, and to treat severe sore eyes and hypersensitivity to strong light. The green leaves, are crushed, wrapped in a thin cloth and used as an eye mask, or the juice is squeezed and applied as eye drops. Additionally, leaves are dried in the shade, made into a powder, mixed together with an equal amount of honey, and licked at bedtime for a good night’s sleep. To treat coughs and tuberculosis in children, leaf powder is mixed with water, warmed, and applied to the chest.

###### Notes.

The medicinal uses of this species in India are discussed in Jain and DeFilipps (1991). Medicinal uses of this species in China are discussed in Duke and Ayensu (1985).

###### References.

Nordal (1963), Agricultural Corporation (1980), Forest Department (1999).

#### 4. *Coriandrum* L.

##### Coriandrum sativum

Taxon classificationPlantaeApialesApiaceae

L.

###### Names.

**Myanmar**: *nannan*, *phat-kyi*, *ta-ner-hgaw*. **English**: Chinese parsley, coriander.

###### Range.

Southern Europe. Cultivated in Myanmar (found as seasonal cultivar throughout country).

###### Uses.

*Seed*: Soaked in water together with *zee-hypu* (*Phyllanthus emblica*) in the early evening, strained the following morning and taken with rock candy to cure headaches; boiled with ginger and taken after meal to improve digestion; boiled with sugar, cooled and taken with rice washing water to treat symptoms of morning sickness in women, such as nausea, vomiting, and pain around heart; powder mixed with sugar and eaten to treat joint aches and pain. Seeds also chewed, and the liquid thus obtained swallowed to treat sore throat. Children can be given a mixture made with the liquid obtained from soaking the seeds and a small amount of sugar to treat bronchitis and asthma.

###### Notes.

Medicinal uses of this species in India are discussed in Jain and DeFilipps (1991). Medicinal uses of the species in China are discussed in Duke and Ayensu (1985).

###### Reference.

Agricultural Corporation (1980).

#### 5. *Daucus* L.

##### Daucus carota

Taxon classificationPlantaeApialesApiaceae

L.

###### Names.

**Myanmar**: *mon-la-ni*, *u-wa-yaing*. **English**: bird’s nest, devil’s plague, Queen Anne’s lace, wild carrot.

###### Range.

Eurasia; widely naturalized. Cultivated in Myanmar.

###### Use.

*Fruit*: Used as a diuretic.

###### Notes.

The species is used as a diuretic and to soothe the digestive tract. An infusion of the herb is employed to treat various complaints including digestive disorders, kidney and bladder disease, and to treat dropsy. An infusion of the leaves is used to counter cystitis and kidney stone formation, and to diminish already formed stones. A warm water infusion of the flowers is used in the treatment of diabetes. The grated raw root is used as a remedy for threadworms. The root is also used to encourage delayed menstruation, and to induce uterine contractions; a tea made from roots serves as a diuretic and is also used to treat urinary stones; and an infusion is used to treat edema, flatulent indigestion, and menstrual problems (Ross 2005).

###### Reference.

Nordal (1963).

#### 6. *Eryngium* L.

##### Eryngium caeruleum

Taxon classificationPlantaeApialesApiaceae

M. Bieb.

###### Name.

**English**: sea holly.

###### Range.

Southern Europe to West Asia.

###### Uses.

*Root*: Used to treat paralysis and as a tonic.

###### Notes.

The chemicals in this plant have been shown to be effective in the treatment of piles, and as a tonic and aphrodisiac (Duke 2009). The root is used as an aphrodisiac and as a nervine (Chopra et al. 1986).

###### Reference.

Nordal (1963).

#### 7. *Foeniculum* Mill.

##### Foeniculum vulgare

Taxon classificationPlantaeApialesApiaceae

Mill.

###### Names.

**Myanmar**: *samon-sabar*, *samon-saba*. **English**: fennel.

###### Range.

Native to the Old World. Now worldwide in tropical and temperate climates; perennial in temperate regions. Cultivated at altitudes up to 1.8 km. In Myanmar, found in Shan.

###### Uses.

*Whole plant*: Used as a digestive and circulatory stimulant, to promote good heart functioning, and to treat a sluggish bowel. *Leaf*: Juice from the crushed leaves consumed to improve urinary functioning and for urinary tract infections. *Fruit*: Used as galactogogue and stomachic. *Seed*: Oil extracted from the seeds is an ingredient in remedies for gastrointestinal problems, including flatulence. A water extract made from fennel seeds soaked overnight in water is sipped to reduce fever; seeds are also eaten to reduce phlegm, flatulence, coughs, nausea, and vomiting. A tea made from seeds steeped in boiling water and then cooled is given to babies with colic and indigestion. Fennel crushed together with young *bael* (*Aegle marmelos*) fruits is taken for indigestion and diarrhea. A mixture of equal parts fennel and sugar is taken at bedtime as a remedy for eye infections.

###### Notes.

Medicinal uses of this species in India are discussed in Jain and DeFilipps (1991). Medicinal use of this species in China is discussed by Duke and Ayensu (1985).

###### References.

Nordal (1963), Agricultural Corporation (1980).

#### 8. *Selinum* L.

##### Selinum wallichianum

Taxon classificationPlantaeApialesApiaceae

(DC.) Raizada & H.O. Saxena (= Selinum tenuifolium Salisb.)

###### Name.

**English**: Wallich milk parsely.

###### Range.

Himalayas, in India and West Pakistan; from Kashmir to Bhutan, 2–3962 m. In Myanmar, found in Kachin.

###### Uses.

*Leaf*: Has bechic, carminative, nervine, antiseptic, and anthelmintic properties. *Leaf* and *Root*: Used to regulate stomach and intestinal functions. Plant used for medicinal purposes (exact uses not given in Nordal 1963).

###### Note.

Perry (1980) discusses the general uses of the genus, including that “the drug is prescribed for colds and diarrhea”.

###### References.

Nordal (1963), Perry (1980).

#### 9. *Trachyspermum* Link

##### Trachyspermum ammi

Taxon classificationPlantaeApialesApiaceae

(L.) Sprague (= Carum copticum Benth & Hook. f.)

###### Names.

**Myanmar**: *samone hpyu*, *gyee baitwine* (Mon). **English**: bishop’s weed, lovage.

###### Range.

Worldwide in tropical and temperate climates. Cultivated in Myanmar.

###### Uses.

*Seed*: With heating properties similar to the seeds of *A. graveolens*, the seeds of *C. copticum* are used to promote appetite, digestion, and gall bladder and gastrointestinal functioning. The pulverized seeds, mixed with ground with pepper, rock salt, and hot water, are ingested as a treatment for stomachaches, dysentery, and sluggish digestion. Blended with yogurt, the seed powder is consumed to eradicate intestinal parasites. A mixture of the seeds and mother’s milk is given to children to alleviate vomiting and diarrhea. A thick paste made from ground seeds and water is applied two to three times daily to quell itching and to heal burns and rashes.

###### Notes.

The seeds of this species are considered antispasmodic, tonic, carminative, and are included in plasters to ease pain. Crushed with a variety of simples, they are prescribed as internal medicine for diseases of the stomach and liver, as well as for sore throats, coughs, and rheumatism (Perry 1980).

The seeds have been found to be an important source of thymol, “a well-known antiseptic” (Perry 1980).

###### Reference.

Agricultural Corporation (1980).

##### Trachyspermum roxburghianum

Taxon classificationPlantaeApialesApiaceae

(DC.) H. Wolff

###### Names.

**Myanmar**: *kant-balu*. **English**: wild celery.

###### Range.

Apparently native to South India. Cultivated as a spice throughout the Indian subcontinent, Southeast Asia, and Indonesia. Cultivated in Myanmar.

Apparently native to South India. Cultivated and adventive in China.

###### Use.

 Plant employed for culinary and medicinal purposes (exact uses not given in Perry 1980).

###### Note.

*Trachyspermum roxburghianum* reported to be used as a stimulant, cardiotonic, carminative, and for dyspepsia (Duke 2009).

In the case of another species in this genus, *T. ammi* (which occurs is Southwest Asia, India, and Northeast Africa), the seeds are considered to be antispasmodic, tonic, a stimulant, carminative, and are included in plasters to ease pain. Crushed with a variety of simples, the seeds are prescribed as internal medicine for diseases of the stomach and liver, for sore throats, coughs, rheumatism, and as a panacea. *T. ammi* seeds are an “important source of thymol, a well-known antiseptic” (Perry 1980).

###### Reference.

Perry (1980).

### Apocynaceae (Dogbane family)

#### 1. *Allamanda* L.

##### Allamanda cathartica

Taxon classificationPlantaeORDOFAMILIA

L.

###### Names.

**Myanmar**: *shwe-pan-new*, *shewewa-pan*. **English**: common allamanda, golden trumpet.

###### Range.

Origin probably in northern South America, but now widespread in tropical America. Cultivated in Myanmar.

###### Uses.

*Bark*: Hydragogue in ascites. *Leaf*: Cathartic (in moderate doses).

###### Note.

In India the bark is used as a hydragogue for ascites; the leaf as a cathartic (Jain and DeFilipps 1991).

###### Reference.

Nordal (1963).

#### 2. *Alstonia* R.Br.

##### Alstonia scholaris

Taxon classificationPlantaeORDOFAMILIA

(L.) R.Br.

###### Names.

**Myanmar**: *letpan-ga*, *taung-mayo*, *taung-meok*. **English**: devil tree, dita bark.

###### Range.

China, Cambodia, India, Malaysia, Myanmar, Nepal, New Guinea, the Philippines, Sri Lanka, Thailand, Vietnam; also Tropical Australia and Africa. In Myanmar, found in Bago, Kachin, Mandalay, Shan, Taninthayi, and Yangon. Grows naturally in the plains and on low hills, particularly in Lower Myanmar.

###### Conservation status.

Lower Risk/least concern [LC] (IUCN 2017).

###### Uses.

*Bark*: Used to treat asthma, heart disease, for chronic ulcers, and other ailments. The powder mixed with ginger is given to new mothers the first day after birthing to cleanse the blood and promote lactation. Bark paste is applied to boils and other sores to minimize inflammation and hasten healing. A bark extract made with boiling water and then mixed with *Cinnamomum obtusifolium* seed powder is sipped to expel intestinal parasites, such as threadworms and roundworms. Reduced to one-third the starting volume, a boiled-water bark extract is consumed to treat lung disease, sour stomach, paralysis, cerebral palsy, heart disease, asthma, fever, shooting pain, and stomachache. Remedies made from the components of the Devil’s tree are known for stimulating the circulatory and respiratory systems, promoting weight gain, and controlling heart disease, asthma, and skin conditions. *Latex*: Applied locally to ulcers, sores, yaws, the hollow of an aching tooth, to mature abscesses or boils, to kill maggots in wounds of cattle, and to draw out thorns and splinters. *Sap*: Applied to sores to stimulate healing; mixed with sesame oil and swabbed inside the ear to treat earache. *Bark*, *Sap*, *Leaf*: Used in treatments for fever, weakness, paralysis, sores, aches, pains, and gastric problems including dysentery. *Leaf*: Used in poultices; green leaves applied to back or dried leaves burned under beds to induce lacteal secretion; infusion of young leaves taken in the morning helpful in cases of beri-beri; leaf tips are taken with roasted coconut to treat stomatitis. Tender leaves are wilted over heat, crushed, and applied to infected sores to accelerate healing.

###### Notes.

Medicinal uses of this species in India are discussed in Jain and DeFilipps (1991) as follows: The bark is a bitter tonic, alterative, anthelmintic, and galactagogue; it is also used for fever, diarrhea, dysentery (powdered and mixed with honey), snakebite and skin diseases, heart disease, leprosy, leucoderma, tumors, rheumatism, cholera, bronchitis, and pneumonia; the juice is used on ulcers and for rheumatic pains; and the root for an enlarged liver. Medicinal use of this species in China is discussed by Duke and Ayensu (1985).

Reported constituents include the following alkaloids: echitamine (also called ditain), ditamine, echitenine, alstonamine, echitamidine (Perry 1980).

Investigators have reported activity against the snail vector, *Lymnaea acuminata*, of the parasitic flukes *Fasciola hepatica* and *F. gigantica* (Singh and Singh 2005), as well as anti-cancer activity in human cancer cell lines (Jagetia and Baliga 2006) and antibacterial activity (Khan et al. 2003).

###### References.

Nordal (1963), Agricultural Corporation (1980), Perry (1980), Forest Department (1999).

#### 3. *Asclepias* L.

##### Asclepias curassavica

Taxon classificationPlantaeORDOFAMILIA

L.

###### Names.

**Myanmar**: *shwedagon*. **English**: blood flower, butterfly weed, red milkweed.

###### Range.

Native of New World, from Florida to South America and West Indies. Widely introduced and cultivated elsewhere.

###### Uses.

*Leaf*: Juice pressed from the leaves for use as a vermifuge, sudorific, and anti- dysenteric. *Leaf* and *Flower*: Pounded leaves and flowers used as a dressing for wounds and sores. *Flower*: Decoction of the flowers is styptic. *Root*: Employed as a purgative, emetic and anthelmintic. Also, in the form of a powder or decoction, used as an emetic and purgative, also as an astringent in dysentery.

###### Notes.

Medicinal uses of this species in India are discussed in Jain and DeFilipps (1991). Medicinal uses of this species in China are discussed by Duke and Ayensu (1985). The listed medicinal uses of the root are the same for China, Indo-China, the Philippines, and Guam as they are for Myanmar; on the Malay Peninsula the flowers are crushed in cold water and used in a poultice for headache (Perry 1980). The toxic properties, symptoms, treatment and beneficial uses of this plant, parts of which are *poisonous*, are discussed by Nellis (1997).

The leaves contain a triterpinoid and an alkaloid. The active glycoside, asclepiadin, is *poisonous*, causing paralysis of the heart, and death (Perry 1980).

###### References.

Nordal (1963), Perry (1980).

#### 4. *Calotropis* R.Br.

##### Calotropis gigantea

Taxon classificationPlantaeORDOFAMILIA

(L.) Dryand. (= Calotropis gigantea (L.) R.Br. ex Schult.)

###### Names.

**Myanmar**: *mayo*. **English**: crown flower.

###### Range.

Tropical Asia, including Myanmar.

###### Uses.

*Sap*: Used in treating leprosy and as a purgative. *Bark*: Used as an anthelmintic. *Bark* and *Latex*: Used to treat skin diseases and as a vermifuge. *Flower*: Used as an antiasthmatic. *Root*: Root bark has been substituted for ipecac, especially to treat dysentery; also used in treating skin disease.

###### Notes.

Medicinal uses of this species in India are discussed in Jain and DeFilipps (1991). In China, the bark of the species is used as a medicine for the treatment of neurodermatitis and syphilis, and the leaves are employed as a poultice (Li et al. 1995).

The latex contains caoutchouc, resins, water soluble matter, and a residue. It yields digitalis-like principles (uscharin, calotropin, and calotoxin), and a nitrogen and sulphur-containg compound, gigantin, which depresses the heart. Calcium oxalate, traces of glutathione, and a proteolytic enzyme similar to papain have also been found (Perry 1980).

###### References.

Nordal (1963), Perry (1980).

##### Calotropis procera

Taxon classificationPlantaeORDOFAMILIA

(Aiton) Dryand.

###### Names.

**Myanmar**: *mayoe*. **English**: swallow-wart.

###### Range.

Tropical Africa and Asia. In Myanmar, along the banks of streams and rivers and along sand bars.

###### Use.

*Root*: Crushed root with water and pressed into aching tooth to cure toothaches. Crushed with the root of the cotton plant to neutralize snake venom. Either the seeds or the root can be made into a paste with water to neutralize scorpion venom. Crushed, slightly warmed and rubbed to cure stiff and aching thighs and calves. Powdered root together with honey will cure skin diseases and leprosy. The root is used as an inhaler for treating epileptic fits. *Flower*: Crushed with milk and taken everyday to cure kidney stones. Stir fried with sesame oil to regulate menstruation. The flowers are used in making medicines to cure cholera. *Latex*: Rubbed and massaged on aching and stiff knees. Crushed with the bark of *hsu-byu* (*Thevetia peruviana*) and applied around the navel and over the bladder to cure retention of urine. Made into a paste with turmeric to treat face discolorations. The latex and the sap of *thanat-taw* (*Garcinia heterandra*) can be made into a paste can reduce swelling of hives and other bumps on the skin. A paste made with *shein-kho* (*Gardenia resinifera*) can reduce unbearable pain. *Stem*: Used as medicine to treat internal hemorrhoids. The dried branch can ignited and the fumes inhaled to cure headaches and stiffness in the neck and back. *Leaf*: The juice from crushing the can be put into the ears to cure earaches. The juice from the crushed leaves taken with a bit of salt will reduce phlegm, asthma, stomach disorders, and distended stomach. Making up ointments to treat paralysis and strokes, and inflammation of joints.

###### References.

Agricultural Corporation (1980), Forest Department (1999).

#### 5. *Carissa* L.

##### Carissa spinarum

Taxon classificationPlantaeORDOFAMILIA

L. (= Carissa spinarum Lodd. ex A.DC.)

###### Names.

**Myanmar**: *khan*, *khanzat*, *taw-khan-pin*. **English**: natal plum.

###### Range.

India and Sri Lanka to Myanmar. Cultivated in Myanmar.

###### Uses.

*Root*: Used as antiseptic and purgative.

###### Notes.

In India the root is an ingredient of purgatives (Jain and DeFilipps 1991).

A tribe in India grinds the roots and uses them in combination with the roots of some other medicinal plants to treat rheumatism. The roots are also a strong purgative (a large dose may prove fatal). Additionally, roughly ground root powder is mixed with water and poured into holes of snakes to serve as a repellant (Parmar and Kaushal 1982).

###### Reference.

Nordal (1963).

#### 6. *Cascabela* Raf.

##### Cascabela thevetia

Taxon classificationPlantaeORDOFAMILIA

(L.) Lippold (= Thevetia peruviana (Pers.) K. Schum.)

###### Names.

**Myanmar**: *hset-hnayarthi*, *mawk-hkam-long* (Shan), *payaung-pan*, *sethnayathi*, *set-hnit-ya-thi*. **English**: exile oleander, lucky nut, Peruvian yellow oleander, yellow oleander.

###### Range.

South America, Neotropical. Found growing naturally throughout Myanmar; also cultivated there.

###### Uses.

Although *poisonous* if consumed by itself, *C. thevetia* is considered effective in preparations for eye infections, as well as for fever, leprosy, and hemorrhoids. *Bark*: Bark preparations are used for fevers, burns, ringworm, and rashes. *Bark*, *Seed*: Bark and seeds are used for a purgative and heart tonic. *Leaf*: The extract from crushed leaves is mixed with water and cooked with olive oil until all of the water evaporates; the resulting oil is used to alleviate joint aches and pains. *Leaf*, *Flower*: The extract from crushed flowers and/or leaves is mixed with water and cooked with olive oil until all of the water evaporates, and the resulting oil is used to treat rashes and other skin disorders. *Root*: Root paste cooked with mustard oil forms an ointment to heal skin problems; mixed with water it is applied as an antifungal to the skin to clear ringworm infections.

###### Notes.

Indigenous medicinal uses of this species in the Andaman and Nicobar Islands (India) are described by Dagar and Singh (1999). Medicinal uses of this species in China are discussed by Duke and Ayensu (1985).

The medicinal uses of this plant in the Caribbean region, as well as its chemistry, biological activity, toxicity and dosages, are discussed by Germosén-Robineau (1997). The chemistry, pharmacology, history and medicinal uses of this species in Latin America are discussed in detail by Gupta (1995). A pharmacognostical profile including medicinal uses of this plant in Africa is given in Iwu (1993). The toxic properties, symptoms, treatment and beneficial uses of this plant, parts of which are poisonous, are discussed by Nellis (1997).

Data on the propagation, seed treatment and agricultural management of this species are given by Katende et al. (1995). Toxicity of this species is discussed by Bruneton (1999). Worldwide medicinal usage, chemical composition and toxicity of this species are discussed by Duke (1986). All parts of the plant contain thevetin and peruvoside which can cause cardiac arrest; peruvoside is however used in medicine for cardiac insufficiency (Lan et al. 1998).

###### References.

Nordal (1963), Agricultural Corporation (1980), Forest Department (1999).

#### 7. *Catharanthus* G. Don

##### Catharanthus roseus

Taxon classificationPlantaeORDOFAMILIA

(L.) G. Don (= Vinca rosea L.)

###### Names.

**Myanmar**: *thinbaw-ma-hnyoe*, *thinbaw-ma-hnyo-pan*, *thinbaw-ma-hnyo-pan-aphyu*. **English**: Madagascar periwinkle, periwinkle, vinca.

###### Range.

Endemic to Madagascar (endangered), but cultivated and naturalized throughout the tropics of both hemispheres, sometimes extending to the subtropics. Found growing naturally around Myanmar; also cultivated.

###### Uses.

This plant is known for neutralizing poisons, facilitating digestion, and promoting weight gain. *Whole plant*: Used to treat diabetes. A boiled water extract of the five parts used to treat diabetes. *Leaf*: Drinking the aqueous extract of leaves alleviates hemor-rhaging during menstruation.

Although there are two kinds of plants – with white or reddish brown flowers – only the plant with the reddish brown flowers is used.

###### Notes.

Medicinal uses of this species in India are discussed in Jain and DeFilipps (1991) as follows: A tea made of the whole plant is used for chitis. The leaf is used for menorrhagia (infusion), wasp stings (juice), and diabetes. The root is used as a purgative and for hypertension; also for leukemia, and is considered anti-cancerous. Medicinal use of this species in China is discussed by Duke and Ayensu (1985). Here the plant is used as an astringent, bechic, depurative, diuretic, emmenagogue; also as an anti-cancer agent.

The species contains the alkaloid serpentine which, like reserpine, is hypotensive, sedative, and tranquilizing (Duke and Ayensu 1985).

*Catharanthus roseus* compounds have been used to develop anticancer drugs, including vinblastine and vincristine (van der Heijden et al. 2004, Ram and Kumari 2001). Duke and Ayensu (1985) extensively discuss the chemical constituents of the plant that are considered valuable in treating various cancers, noting that “More than 50 alkaloids have been identified from this major medicinal plant,” and the species contains several hypo-glycemic alkaloids (catharanthine, leurosine sulphate, lochnerine, tetrahydro- alstonine, vindoline, and vindolinine) used in treating various cancers.

###### References.

Nordal (1963), Agricultural Corporation (1980).

#### 8. *Dregea* E.Mey.

##### Dregea volubilis

Taxon classificationPlantaeORDOFAMILIA

(L.f.) Benth. ex Hook.f.

###### Names.

**Myanmar**: *kway-tauk nwai*, *gwedauk-nwe*. **English**: giant swallowart.

###### Range.

China, Bagladesch, Cambodia, India, Indonesia, Kashmir, Laos, Malaysia, Nepal, the Philippines, Sri Lanka, Thailand, Vietnam. Grows naturally over Myanmar.

###### Uses.

Known for its bitter taste and heating properties, *D. volubilis* is an ingredient in preparations given to regulate bowels, strengthen blood, promote virility, and stimulate appetite, as well as to alleviate sore throat, gonorrhea, asthma, and conditions caused by ingestion of rat poison. *Leaf*: Fire-roasted until limp and placed on sores and boils to reduce swelling, drain pus, and induce healing; given to alcoholics cooked with chicken to purge accumulated toxins. In soups or fried leaves are eaten to relieve flatulence and improve urine flow. The juice of crushed leaves is applied to herpes sores, and also used in a poultice to eliminate bumps and tumors. Pulverized with sugar they are applied to alleviate a stiff neck and similar problems. Fried with duck eggs (traditionally used more commonly than chicken eggs since considered more medicinally potent), they are consumed for strength and vitality. *Root*: Used in remedies for rabies as well as in emetic and in expectorant preparations.

###### Note.

Medicinal uses of this species in India are discussed in Jain and DeFilipps (1991).

###### References.

Agricultural Corporation (1980), Forest Department (1999).

#### 9. *Holarrhena* R.Br.

##### Holarrhena pubescens

Taxon classificationPlantaeORDOFAMILIA

Wall. ex G.Don. (= Holarrhena antidysenterica (Roth) Wall. ex A.DC.)

###### Names.

**Myanmar**: *dangkyam*, *danghkyam kaba*, *maiyang*, *mai-hkao-long*. **English**: rosebay, tellicherry bark.

###### Range.

Tropical Africa and in Southeast Asia, from Pakistan to Malaysia.

###### Conservation status.

Least Concern [LC] (IUCN 2017).

###### Use.

*Bark*: Used in stopping the bleeding related to internal piles. The paste of the bitter bark made with the liquid from yogurt can be taken to treat gall stones. Powered bark stirred into water can cure fever. Boil bark with a small amount of salt and *shein-kho* (*Gardenia resinifera*) to treat stomach pains. Crushed bark with milk will cure pain in passing urine and retention of urine. To cure earaches and ear infections, a small amount of powdered bark can be tipped into the ear followed by liquid droppings from crushed or squeezed leaves. Roasted powdered bark taken with honey and butter can cure muscle pains, knotted muscles, dysentery, and cholera. *Root*: A paste made with hot water can be taken twice a day to cure bloated or distended stomach. A paste made with alcohol and taken with salt can cure blood in the stool associated with smallpox. For sore throat associated with smallpox, the root must be crushed with salt and kept in the mouth. The powder of root and *zawet-thar* (*Dillenia indica*) can be taken with milk to cure gall stones. A paste made with water and taken with a bit of *eik-mwei* (*Embelia tsjeriam-cottam*) fruit can act as a de-worming medicine. *Flower*: Can facilitate digestion, and control flatulence, phlegm, bile, leprosy and infections.

###### References.

Agricultural Corporation (1980), Forest Department (1999).

#### 10. *Ichnocarpus* R.Br.

##### Ichnocarpus frutescens

Taxon classificationPlantaeORDOFAMILIA

(L.) W.T. Aiton

###### Names.

**Myanmar**: *taw-sabe*, *twinnet*, *twinnet-kado*. **English**: black creeper, kalisar, red sarsaparilla, sariva, sarsaparilla.

###### Range.

China, Bangladesh, Bhutan, Cambodia, India, Indonesia, Laos, Malaysia, Myanmar, Nepal, New Guinea, Pakistan, the Philippines, Sri Lanka, Thailand, Vietnam; also Australia. In Myanmar, found in Bago, Sagaing, Shan, Taninthayi, and Yangon.

###### Uses.

*Leaf*: Antipyretic. *Root*: Tonic.

###### Notes.

The medicinal uses of this species in India are discussed in Jain and DeFilipps (1991). Some of the uses follow: The bark is used for bleeding gums; the leaf for fever and headache. The root is used to purify blood; also to treat coughs (with linseed), haematuria, convulsions, night blindness, and ulcers on the tongue (with roots of *Michelia champaca* or *Celastrus* species) and palate; additionally, to treat sunstroke, atrophy, cachexy, enlarged spleen, sores, syphilis, dysentery, cholera, animal bites (with other plants), and smallpox.

###### References.

Nordal (1963), Forest Department (1999).

#### 11. *Kopsia* Blume

##### Kopsia fruticosa

Taxon classificationPlantaeORDOFAMILIA

(Roxb.) A.DC.

###### Names.

**Myanmar**: *kalabin*, *mai-lang*, *thinbaw-zalut*, *zalut-ni*, *zalut-panyaung*. **English**: shrub-vinca.

###### Range.

Malay Peninsula. Native to Myanmar; now widely cultivated. Cultivated in Myannmar.

###### Uses.

*Root*: Pounded root employed as poultice. Nordal (1963) lists species as having medicinal value, but exact uses not given.

###### Notes.

. The species is used medicinally for sores and syphilis; also cholinergic (chemical found in plant shown to be effective for this). *Kopsia fruticosa* contains latex used in arrow poison (Duke 2009).

A *very poisonous* alkaloid is found in the bark, leaves, and seeds. The alkaloid kopsine has been isolated from the leaves of plants of this species growing in India. Other alkaloids are also present (Perry 1980).

###### References.

Nordal (1963), Perry (1980).

#### 12. *Nerium* L.

##### Nerium oleander

Taxon classificationPlantaeORDOFAMILIA

L. (= Nerium indicum Mill.; Nerium odorum Soland.)

###### Names.

**Myanmar**: *nwei thargi*. **English**: oleander, rose of Sharon.

###### Range.

From Mediterranean to the Arabian Peninsula, Ethiopia, Niger, Afghanistan, Iran and Iraq to India and central China. Found all over Myanmar; naturalized, also cultivated as an ornamental plant.

###### Conservation status.

Least Concern [LC] (IUCN 2017).

###### Uses.

This plant is *poisonous* if ingested; it can be applied externally only.

*Leaf*: Powder from pulverized leaves used for ringworm, itchy skin, and other external inflammations; alternatively, the boiled water extract of leaves is used to alleviate inflammation. Liquid from crushed leaves is applied to snakebites to neutralize the venom, as well as to bites or stings from other venomous animals. *Root*: The root powder is applied to the skin to alleviate headache and neutralize poisons from scorpion and snakebites. Mixed with water, the root powder is applied as an ointment for skin cancer, ringworm and other fungal conditions, earache, infected lesions, and leprosy.

###### Notes.

Medicinal use of this species in China is discussed by Duke and Ayensu (1985).

In India the leaf is used as a cardiotonic and oil from the root bark is employed for skin diseases (Jain and DeFilipps 1991).

The bark contains glycosides with digitalis-like activity (Jain and DeFilipps 1991). *N. indicum* bark extract has activity against the snail vector, *Lymnaea acuminata*, of the parasitic flukes *Fasciola hepatica* and *F. gigantic* (Singh and Singh 1998), as well as antiviral activity against influenza and herpes simplex (Rajbhandari et al. 2001).

###### References.

Nordal (1963), Agricultural Corporation (1980).

#### 13. *Plumeria* L.

##### Plumeria rubra

Taxon classificationPlantaeORDOFAMILIA

L. (= Plumeria acutifolia Poir.; Plumeria acuminata W.T. Aiton)

###### Names.

**Myanmar**: *mawk-sam-ka*, *mawk-sam-pailong*, *sonpabataing*, *tayoksaga-ani tayok-saga* (red form). **English**: frangipani, pagoda tree, red plumeria.

###### Range.

Mexico, Central America, South Asia. Found growing naturally all over Myanmar except in very cool mountainous areas; also cultivated.

###### Uses.

Known to promote digestive, excretory, respiratory, and immune functioning, with activity against leprosy, infections, and stomach ailments. *Sap*: The milky sap from the branches and bark is used as a laxative; also in remedies for stomachache and bloating. *Bark* and *Leaf*: Used as laxative and for gonorrhea and venereal sores. *Leaf* and *Flower*: The leaves can be eaten, the flowers can either be boiled in water and eaten or boiled in tamarind (*Tamarindus indica*) juice and made into a salad to promote regular bowel movements and urine flow, as well as to control gas and phlegm. *Flower*: Used for treatment of asthma.

###### Notes.

Medicinal uses of this species in India are discussed in Jain and DeFilipps (1991). Medicinal use of this species in China is discussed by Duke and Ayensu (1985). Perry (1980) discusses the species’ medicinal uses in Indo-China, Indonesia, the Philippines, and Palau.

Researchers report cytotoxic activity against human cancer cell lines (Kardono et al. 1990), as well as molluscicidal and antibacterial activity (Hamburger et al. 1991).

Reported chemical constituents include agoniadin, plumierid, plumeric acid, cerotinic acid, and lupenol; the stem contains the alkaloid, triterpinoid. A new antibiotic, fluvoplumierine, which inhibits growth of *Mycobacterium tuberculosis*, has also been found (Perry 1980).

###### References.

Nordal (1963), Agricultural Corporation (1980), Forest Department (1999).

#### 14. *Rauvolfia* L.

##### Rauvolfia serpentina

Taxon classificationPlantaeORDOFAMILIA

(L.) Benth. ex Kurz

###### Names.

**Myanmar**: *bommayazar*, *bomma-yaza*. **English**: Indian snakeroot, serpent wood.

###### Range.

India to Java. In Myanmar, found in Bago, Chin, Kayin, Mandalay, Mon, and Yangon.

###### Uses.

This astringent, sharp, and bitter plant is used to improve digestion, relieve gas, and stimulate taste buds, as well as to alleviate paralysis, trembling, male-related disorders leading to excessive semen, and gonorrhea. It is also used for other venereal diseases, hypertension, anemia, heart palpitations, impotence, and lack of semen. *Leaf*: Fresh juice used in medicines for eye conditions. *Leaf*, *Root*: Used as sedative. *Root*: Remedies made from the root are well known for reducing blood pressure, especially in young people with anxiety-related palpitations and hypertension. Root remedies are also used as a tranquilizer to calm aggression, restlessness, and excitability in patients with mental disorders. In addition, the root is used in tonics, sleeping aids, carminatives, fever reducers, and poison neutralizers. Pulverized root, in equal amounts with *shein-kho* (*Gardenia resinifera*), *eikthara-muli* (*Euonymus kachinensis*), and *hsay-dan* (*Hygrophila phlomoides*), is either crushed with one betel (*Piper betle*) leaf or mixed with sesame oil and applied all over an infant’s body (with the exception of the palms of the hands and the soles of the feet) as an inhaled therapy to relieve bronchitis and vomiting. Alternatively, the powder on a person’s warmed hands is applied as a chest rub for children. It is noted that following use of medicine made from this plant, the patient should eat foods with heating properties and bathe regularly.

###### Notes.

Medicinal uses of this species in India are discussed in Jain and DeFilipps (1991). The species has been used for centuries in Indian Ayurveda medicine to treat snakebite and insanity. Ayurveda uses of *R. serpentina* (“sarpagandha”) are discussed in Kapoor (1990). Indigenous medicinal uses of this species in the Andaman and Nicobar Islands (India) are described by Dagar and Singh (1999).

*Rauvolfia serpentina* is the source of the first modern plant-derived antipsychotic and antihypertensive drug, reserpine, used in psychiatry and for lowering blood pressure (Shah 1995). Details of the active chemical compounds, effects, herbal usage and pharmacological literature of *R. serpentina* are given in Fleming (2000) and Duke (1986). Medicinal properties of this species are discussed by Blackwell (1990).

###### References.

Nordal (1963), Agricultural Corporation (1980), Forest Department (1999).

#### 15. *Tabernaemontana* L.

##### Tabernaemontana divaricata

Taxon classificationPlantaeORDOFAMILIA

(L.) R.Br. ex Roem. & Schult. (= Ervatamia coronaria (Willd.) Stapf)

###### Names.

**Myanmar**: *lashi*, *taw-zalat*, *zalat*, *zalat-seikya*. **English**: Adam’s apple, crape gardenia, crape jasmine, East Indian rosebay, linwheel flower, moonbeam.

###### Range.

Thought to be a native of India, but now cultivated throughout Continental and Southeast Asia. Cultivated in Myanmar.

###### Uses.

*Root*: Emmenagogue and tonic.

###### Notes.

In India the stem bark serves as a refrigerant; the leaf’s milky juice is used in the treatment of eye diseases; and the root is applied locally an anodyne, as well as chewed to relieve toothache (Jain and DeFilipps 1991). Perry (1980), noting that the species’ uses in each geographical division are diverse, discusses its uses in Indo-China, the Malay Peninsula, and Amboina.

Reported chemical constituents (alkaloids from the bark of the stem and root) are tabernaemontanine, coronarine, coronaridine, and dregamine; alkaloids also occur in all of the vegetative parts (Perry 1980).

###### Reference.

Nordal (1963).

#### 16. *Vallaris* Burm.f.

##### Vallaris solanacea

Taxon classificationPlantaeORDOFAMILIA

(Roth) Kuntze

###### Names.

**Myanmar**: *khinbok*, *nabu-nwe*. **English**: bread flower.

###### Range.

India and Sri Lanka. In Myanmar, found is Bago, Kachin, Mandalay, and Yangon.

###### Use.

*Juice*: Applied to sores.

###### Notes.

In India the bitter bark is employed as an astringent; the latex, an irritant, is applied on wounds and sores (Jain and DeFilipps 1991). In Indo-China the bark is used as a febrifuge (Perry 1980).

The plant has been found to contain cardiotonic glycosides (Perry 1980).

###### References.

Perry (1980), Forest Department (1999).

#### 17. *Wrightia* R.Br.

##### Wrightia arborea

Taxon classificationPlantaeORDOFAMILIA

(Dennst.) Mabb. (= Wrightia tomentosa Roem. & Schult.)

###### Names.

**Myanmar**: *danghkyam-kaii*, *lettok-thein*, *mai-lang*, *mai-yang-hka-oaun*, *taung-zalut*. **English**: woolly dyeing rosebay.

###### Range.

China, India, Laos, Malaysia, Myanmar, Sri Lanka, Thailand, and Vietnam. In Myanmar, found in Ayeyarwady, Bago, Mandalay, and Yangon.

###### Use.

*Bark*: Administered for renal complaints.

###### Notes.

The medicinal uses of this species in India are discussed in Jain and DeFilipps (1991) as follows: The bark is used as a substitute for *Holarrhena* bark, for stomachache and colic; the root is used for fever, dysentery (with root of *Cissampelos*); and an unspecified plant part is used for tumors. In Indo-China the species is used as an astringent and alexiteric (Perry 1980).

Tests for the presence of alkaloids in this species were negative (Perry 1980).

###### References.

Perry (1980), Forest Department (1999).

### Araceae (Arum family)

#### 1. *Amorphophallus* Blume ex Decne.

##### Amorphophallus paeoniifolius

Taxon classificationPlantaeORDOFAMILIA

(Dennst.) Nicolson (= Amorphophallus campanulatus Decne.)

###### Names.

**Myanmar**: *wa-u-bin*, *wa-u-pin*. **Japanese**: *shinasoo*. **English**: elephant yam, cobra plant.

###### Range.

Paleotropics. Found only in Myanmar’s temperate regions; grows naturally.

###### Conservation status.

Least Concern [LC] (IUCN 2017).

###### Uses.

*Tuber*: Used to prevent sagging belly in women and enlargement of the bladder. They are also used to trim the body and clear the complexion, to prevent palpitations in older people, and to stop the formation of excess fat and solidified fatty deposits in the body.

###### Note.

In its genus, this species is considered one of the most effective medicinally and subsequently one of the most desired by international buyers.

The medicinal uses of this species in India are discussed in Jain and DeFilipps (1991).

###### Reference.

Agricultural Corporation (1980).

#### 2. *Colocasia* Schott

##### Colocasia antiquorum

Taxon classificationPlantaeORDOFAMILIA

Schott

###### Names.

**Myanmar**: *mahuya-pein*, *pein*, *pein-u*. **English**: taro, kalo, dasheen, eddo.

###### Range.

Java. Cultivated in Myanmar.

###### Use.

*Juice*, *Corm*: Skin irritant.

###### Notes.

In India the tuber is hemostatic on injuries, cuts, burns, and honey bee stings (Jain and DeFilipps 1991).

###### Reference.

Nordal (1963).

#### 3. *Pothos* L.

##### Pothos scandens

Taxon classificationPlantaeORDOFAMILIA

L.

###### Names.

**Myanmar**: *pein-gya*. **English**: pothos.

###### Range.

Widespread from Madagascar, through India and the Himalayas to southwestern China, south through Indochina; also in Peninsular Malaysia, Borneo, and the Philippines. In Myanmar, found in Shan, Taninthayi, and Yangon.

###### Use.

*Leaf*: Used as antiasthmatic.

###### Notes.

The medicinal uses of this species in India are discussed in Jain and DeFilipps (1991) as follows: The root is fried in oil and applied on abscesses; the stem is smoked with camphor to treat asthma; and the leaf is powdered and used on smallpox pustules and fractures.

###### Reference.

Nordal (1963).

#### 4. *Typhonium* Schott

##### Typhonium trilobatum

Taxon classificationPlantaeORDOFAMILIA

(L.) Schott

###### Name.

**English**: Bengal arum.

###### Range.

Temperate China; tropical Bangladesh, India, Nepal, Sri Lanka; Indo-China; Malaysia. Naturalized elsewhere. In Myanmar, found in Yangon.

###### Uses.

*Root*: Acrid tubers applied in poultices as a counter-irritant, and also to destroy maggots in sores on cattle.

###### Notes.

The medicinal uses of this species in India are discussed in Jain and DeFilipps (1991) as follows: The root is used to treat snakebite, and is externally applied and orally administered (at the same time);, the root, eaten with bananas, is used to treat stomach complaints; also used as a stimulant, and as a remedy for piles. Perry (1980) gives medicinal uses for the species in Thailand and Indonesia.

###### Reference.

Perry (1980).

### Araliaceae (Ginsing family)

#### 1. *Schefflera* J.R.Forst. & G.Forst.

##### Schefflera venulosa

Taxon classificationPlantaeORDOFAMILIA

(Wight & Arn.) Harms

###### Names.

**English**: rubber tree, starleaf, umbrella tree.

###### Range.

Native to China, India, Myanmar, and Indo-China.

###### Uses.

*Leaf*: Infusion used for many internal diseases.

###### Notes.

The species is reported to be employed for toothache (Duke 2009). A decoction of the plant is used in Indo-China the first 15 days of puerperium (Duke and Ayensu 1985).

###### References.

Perry (1980), Duke and Ayensu (1985).

### Arecaceae (Palm family)

#### 1. *Caryota* L.

##### Caryota mitis

Taxon classificationPlantaeORDOFAMILIA

Lour.

###### Names.

**Myanmar**: *minbaw*, *tamibaw*. **English**: clustered fishtail palm, fishtail palm, wine palm.

###### Range.

Southeast Asia, from Myanmar to the Philippines.

###### Use.

*Fruit*: Irritant (*poisonous*).

###### Notes.

In Indo-China the fibers from the axils of the leaves are applied in the form of moxas for cauterization of bites of poisonous animals or insect stings; on the Malay Peninsula the fruits may be put into juice, mixed with bamboo hairs and toad-extract, and used to poison food. Even the fruit’s pulp causes skin irritation (Perry 1980).

###### Reference.

Nordal (1963).

### Aristolochiaceae (Birthwart family)

#### 1. *Aristolochia* L.

##### Aristolochia indica

Taxon classificationPlantaeORDOFAMILIA

L.

###### Names.

**Myanmar**: *eik-thara*, *eik-tha-ra-muli*, *thaya-muli*. **English**: Indian birthwort.

###### Range.

Native of India and eastward; sometimes cultivated in Indo-China. In Myanmar found in Bago, Mandalay, and Yangon.

###### Uses.

*Whole plant*: For children, a mixture of equal amounts of the leaf juice and the juice squeezed from the crushed five parts is given to heal throat blisters, mouth blisters, and canker sores. *Leaf*: For edema and dry coughs, the juice squeezed from the crushed leaves is taken with a small amount of salt once in the morning and once in the evening. The strained juice, made from two or three of the leaves crushed finely together with eight to ten peppercorns, is given at 15-minute intervals for venomous bites from snakes and scorpions, as well as from other sources. This medicine is also used to revive and stimulate circulation in patients who have severe colds, who have lost consciousness, or who have poor circulation. *Leaf* and *Root*: Medicines made from the roots and leaves are used to treat poisoning, coughs, heart disease, intestinal disorders in children, indigestion and gas problems, swollen and aching joints, irregular menstruation, blood irregularities, and dizziness. *Root*: The paste is applied topically to neutralize poison from snake, scorpion, and other venomous bites; a small amount is rubbed onto the tongue to alleviate fever from stomach upset in children and infants; and orally or rubbed on the tongue, used to quell delirium from high fevers and to alleviate heaviness of the lips, jaw, cheeks, and tongue. Root powder mixtures with black pepper powder, raw salt, and warm water, used to regulate menstruation and promote menstrual bloodflow; with equal parts of wheat ash and salt, taken orally with hot water or applied topically to swollen parts of the body to soothe aches, pains, and inflamed joints; and two parts of the root powder and one part ginger powder is given twice daily for dysentery or indigestion. The root is also used in preparations to ease childbirth, clear menstruation-related skin discolorations, and reduce fevers.

###### Notes.

The medicinal uses of this species in India are discussed in Jain and DeFilipps (1991) as follows: The whole plant is used for snakebite; leaf juice is used for snakebite, breast pain and suppuration, as an abortifacient; the seed is used for inflammation, joint pains; the root is used as a stimulant, emetic, emmenagogue, for fever, leucoderma (powdered and mixed with honey); to promote digestion, regulate menstruation (in small doses); on wounds, for diarrhea (paste), and for snakebite. An unspecified plant part is used to stimulate phagocytosis; also for cholera. In Indo-China the plant is used as a remedy for intermittent fever, dropsy, and loss of appetite; the root is used for the same purpose (Perry 1980).

The essential oil contains a trace of camphor, and sesquiterpenes, ishwarene, ishwarone, and ishwarol The roots contain an alkaloid, aristolochine, a yellow bitter principle, isoaristolochic acid, and allantoin (Perry 1980).

###### References.

Nordal (1963), Agricultural Corporation (1980), Perry (1980).

##### Aristolochia tagala

Taxon classificationPlantaeORDOFAMILIA

Cham.

###### Names.

**English**: Dutchman’s pipe, Indian birthwort.

###### Range.

China, Taiwan, Bangladesh, Bhutan, Cambodia, India, Indonesia, Japan, Nepal, Malaysia, Myanmar, the Philippines, Sikkim, Thailand, Vietnam; Soloman Islands and Queensland in Australia. In Myanmar, found in Chin, Kayin, Mandalay, Sagaing, and Yangon.

###### Uses.

*Whole plant*: Used for bowel complaints. *Fruit*: Used as a laxative and tonic.

###### Notes.

The medicinal uses of this species in India are discussed in Jain and DeFilipps (1991) as follows: The whole plant is used for bowel complaints; the fruit is used for rheumatism (paste applied and massaged in), malaria, dyspepsia, snakebite, toothache (paste applied); the uses of the root are the same as those of the fruit.

###### References.

Nordal (1963), Perry (1980).

### Asparagaceae (Asparagus family)

#### 1. *Agave* L.

##### Agave sisalana

Taxon classificationPlantaeORDOFAMILIA

Perrine

###### Names.

**Myanmar**: *nanat-gyi*, *na-nat-shaw*, *thinbauk-nanat*. **English**: sisal, sisal hemp.

###### Range.

Eastern Mexico. Cultivated in Myanmar.

###### Use.

*Whole plant*: Juice used as purgative.

###### Notes.

A pharmacognostical profile including medicinal uses of this plant in Africa is given in Iwu (1993). The toxic properties, symptoms, treatment and beneficial uses of this plant, *parts of which are poisonous*, are discussed by Nellis (1997). Data on the propagation, seed treatment and agricultural management of this species are given by Katende et al. (1995). Worldwide medicinal usage, chemical composition and toxicity of this species are discussed by Duke (1986).

###### Reference.

Nordal (1963).

##### Agave vera-cruz

Taxon classificationPlantaeORDOFAMILIA

Mill.

###### Names.

**Myanmar**: *thin-baw-na-nat*. **English**: blue aloe.

###### Range.

Mexico. Introduced into southern Europe, northwestern Africa, Mauritius, India, and Sri Lanka. Cultivated in Myanmar.

###### Use.

*Juice*: Used as purgative.

###### Note.

In India the whole plant is used as a purgative (Jain and DeFilipps 1991).

###### Reference.

Nordal (1963).

#### 2. *Asparagus* L.

##### Asparagus filicinus

Taxon classificationPlantaeORDOFAMILIA

Buch.-Ham. ex D.Don

###### Names.

**Myanmar**: *ka-nyut*. **English**: fern asparagus.

###### Range.

India and China. In Myanmar, found in Chin, Kachin, Magway, Mandalay, Sagaing, and Shan.

###### Conservation status.

Data Deficient [DD] (IUCN 2017).

###### Uses.

*Root*: Used as diuretic and anthelmintic.

###### Notes.

In India the root is used as an astringent and tonic (Jain and DeFilipps 1991). In China the root is used as an antipyretic, bechic, diuretic, expectorant, nervine, stimulant, and tonic; also for constipation, cough, hemoptysis, dry throat, pertussis (Yunnan); and cooked with pork for a tonic (Duke and Ayensu 1985). Perry (1980) notes the medicinal use of this species in Yunnan. She also states that the species has uses similar to *A. cochinchinensis*.

###### Reference.

Nordal (1963).

##### Asparagus officinalis

Taxon classificationPlantaeORDOFAMILIA

L.

###### Names.

**Myanmar**: *kannyut*, *sani kamat* (Mon). **English**: asparagus.

###### Range.

Europe, Asia, North Africa. Not common in Upper Myanmar. Found in humid locations; cultivated in hilly and cooler regions.

###### Conservation status.

Least Concern [LC] (IUCN 2017).

###### Uses.

*Whole plant*: Has cooling properties and a sweet taste. Leaves, stems, shoots, roots and fruits are all beneficial for humans. The plant is considered especially beneficial for new mothers, to fortify the blood and help prevent anemia. It is used to break up phlegm, as well as to control the gall bladder, external hemorrhaging, and vomiting of blood. *Shoot*: Eaten to eliminate gas and to strengthen the body. *Shoot* and *Root*: Considered especially useful for extra strength, either cooked on their own or incorporated into rice pudding with milk. *Root*: Bulbous, can be boiled to make a paste for external application as a remedy for inflamed joints, aches, and flatulence disorders. For urinary tract disorders and various liver and gall bladder diseases, the juice of the roots mixed with honey and/or milk is ingested. The juice mixed with an equal amount by weight of milk is consumed as a cure for long-standing kidney stones and gallstones. It is also taken as a cure for diseases caused by poisoning.

###### Notes.

The medicinal uses of this species in India are discussed in Jain and DeFilipps (1991). Medicinal uses of this species in China are discussed in Duke and Ayensu (1985). Medicinal uses of asparagus are also discussed in Perry (1980).

###### Reference.

Agricultural Corporation (1980).

#### 3. *Dracaena* L.

##### Dracaena angustifolia

Taxon classificationPlantaeORDOFAMILIA

(Medik.) Roxb.

###### Names.

**Myanmar**: *dan-la-ku*, *dandagu*, *dantalet*.

###### Range.

India and South China to the Solomon Islands. In Myanmar, found in Mandalay, Mon, and Sagaing.

###### Use.

*Leaf*: Used as a blood purifier.

###### Notes.

In the Philippines the roots are chewed, and the saliva swallowed as a remedy for centipede bites; additionally, a decoction of the roots is ingested to treat stomach problems. In the older literature, the medicinal uses of this species are listed as follows: A decoction of the leaves is ingested to treat dysentery, leucorrhea, and blennorrhea; also considered to be a galactagogue. A decoction of the roots along with *Tectaria crenata* (*Aspidium repandum*) is taken twice a day for a week to treat gonorrhea (Perry 1980).

###### Reference.

Nordal (1963).

### Asphodelaceae (Asphodelus family)

#### 1. *Aloe* L.

##### Aloe vera

Taxon classificationPlantaeORDOFAMILIA

(L.) Burm.f.

###### Names.

**Myanmar**: *men-khareek-leck-chuck* (Mon), *sha-zaung-let-pat*. **English**: aloe, bitter aloe.

###### Range.

Canary Islands and Arabian Peninsula.

###### Use.

*Leaf*: Used to treat menstrual disorders. The inner gelatinous flesh can be eaten sprinkled with a little salt obtained from making an ash of the five parts of the *pauk* plant (*Butea monosperma*), to cleanse the menstrual blood. Used against boils, edema, liver diseases, skin diseases, fevers, asthma, leprosy, jaundice, and bladder stones. Used as a powerful and effective as an ointment. If the inner flesh is used as a poultice against the stomach, it will draw out internal myomas and tumors. The inner gel can be placed on the eyes to cure eyes that are sore or ache. Squeezing out the inner gel, pouring it into the ear after warming it will cure earaches speedily. If a person suffering from jaundice eats the inner gel, it will give good bowel movements and encourage urination, curing the condition. If the inner gel is scraped off, soaked in rice washing water, and added to sugar, it can be taken to cure urinary disorders.

###### Notes.

Chemical constituents, pharmacological action, and medicinal use of this species in Indian Ayurveda are discussed in detail by Kapoor (1990). Indigenous medicinal uses of this species in the Andaman and Nicobar Islands (India) are described by Dagar and Singh (1999). The chemical constituents, pharmacological activities, and traditional medicinal uses of this plant on a worldwide basis are discussed in detail by Ross (1999). A pharmacognostical profile including medicinal uses of this plant in Africa is given in Iwu (1993). Details of the chemical compounds, effects, herbal usage and pharmacological literature of this plant are given in Fleming (2000). Worldwide medicinal usage, chemical composition and toxicity of this species are discussed by Duke (1986). Medicinal properties of this species are discussed by Blackwell (1990). *Aloe vera* leaves contain barbaloin, which is poisonous (Lan et al. 1998).

###### References.

Agricultural Corporation (1980), Forest Department (1999).

### Asteraceae (Sunflower family)

#### 1. *Ageratum* L.

##### Ageratum conyzoides

Taxon classificationPlantaeORDOFAMILIA

(L.) L.

###### Names.

**Myanmar**: *kado-po*, *kadu-hpo*. **English**: goatweed, tropical whiteweed.

###### Range.

New World Tropics. In Myanmar found in Mandalay, Shan, and Yangon.

###### Use.

*Leaf*: Serves as an antiseptic for skin diseases and leprosy.

###### Notes.

Medicinal uses of this species in India are discussed in Jain and DeFilipps (1991). Indigenous medicinal uses of this species in the Andaman and Nicobar Islands (India) are described by Dagar and Singh (1999). Medicinal uses of this species in China are discussed by Duke and Ayensu (1985). The chemistry, pharmacology, history and medicinal uses of this species in Latin America are discussed in detail by Gupta (1995). A pharmacognosti-cal profile including medicinal uses of this plant in Africa is given in Iwu (1993).

###### Reference.

Nordal (1963).

#### 2. *Artemisia* L.

##### Artemisia dracunculus

Taxon classificationPlantaeORDOFAMILIA

L. (= Artemisia glauca Pall. ex Willd.)

###### Names.

**Myanmar**: *dona-ban*. **English**: estragon, false tarragon, French tarragon, green sagewart, silky wormwood, tarragon.

###### Range.

Origin thought to have been Central Asia, probably Siberia. Current range southern Europe, Asia, United States, west to the Mississippi River.

###### Uses.

*Root*: Used as tonic, antiseptic, and antiasthmatic.

###### Notes.

The leaves and young shoots of the species are said to be of particular value for their beneficial effect on digestion. In addition to stimulating the digestive system and uterous, the leaves, and an essential oil obtained from them, lower fevers and destroy intestinal worms; they also serve as an antiscorbutic, diuretic, emmenagogue, febrifuge, hypnotic, odontalgic, stomachic, and vermifuge (Bown 1995). An infusion is used to treat indigestion, flatulence, nausea, and hiccups; and a poultice is employed to relieve rheumatism, gout, arthritis, and toothache. (Phillips and Foy 1990). Also, the plant is mildly sedative as is used to aid sleep (Chevallier 1996). The root is used to treat digestive and menstrual problems (Bown 1995). The medicinal uses of eight other members of the genus in China are discussed in Duke and Ayensu (1985). These too have many valuable uses as well as an important chemical composition.

###### Reference.

Nordal (1963).

#### 3. *Blumea* DC.

##### Blumea balsamifera

Taxon classificationPlantaeORDOFAMILIA

(L.) DC.

###### Names.

**Myanmar**: *bonmathane-payoke*, *hpon-mathein*, *phon-ma-thein*. **English**: dog bush, nagi camphor, shan camphor.

###### Range.

South and southeastern Asia, China, and Taiwan. Widespread in Myanmar.

###### Uses.

*Leaf*: Used as an expectorant, stomachic, antispasmodic, and antiseptic. Used to treat infantile illnesses. Bathing the body with water in which the leaves have been soaked gets rid of edema. Apply an ointment made by mixing the leaves with alcohol, rose water and lime juice to alleviate and cure muscles spasms and tics, paralysis of limbs, heaviness of limbs due to poor circulation of blood, and aches and pains in the body. *Sap*: Used in curing toothaches. *Root*: Used in treating colds.

###### Notes.

Medicinal uses of this species in China are discussed in Duke and Ayensu (1985) as follows: The whole plant is used as a stomachic, sudorific, tonic, expectorant, diaphoretic, anticatarrhal; also considered a potential antifertility plant. Juice from fresh leaves, or decocted dry leaves, is used for itch, sores, and wounds. In India a decoction of the whole plant is used as an expectorant; a warm infusion as a sudorific (Jain and DeFilipps 1991).

The reported chemical composition includes cineole and limonene; also palmitic acid, myristic acid, sesquiterpemne alcohol, dimethly ether, and pyrocaechic tannin (Perry 1980). Herbal extracts are phototoxic to *Saccharomyces cerevisiae*. “The aqueous extract is said to be efficacious as a vasodilator, sedative and hypotensive. Since it inhibits the sympatheric nervous system, it is used to relieve excitement and insomnia.” It is thought that the essential oil may be nearly pure borneol, or 75% camphor and 25% borneol (Duke and Ayensu 1985).

###### References.

Nordal (1963), Agricultural Corporation (1980).

#### 4. *Carthamus* L.

##### Carthamus tinctorius

Taxon classificationPlantaeORDOFAMILIA

L.

###### Names.

**Myanmar**: *hsu pan*. **English**: false saffron, safflower, wild saffron.

###### Range.

Origin thought to be the eastern Mediterranean. Currently known only in cultivation and as escapes. Found as a cultivar in Myanmar.

###### Uses.

*Leaf*: Considered bitter and sweet, with heating properties, can cause loose bowels but are known for promoting good vision, digestion, gall bladder function, and phlegm discharge. The leaves are consumed in a sour soup (fish or shrimp stock base, tamarind, and vegetables) to promote the flow of urine and to give vigor. *Flower*: Juice from the crushed flowers is taken to neutralize snake and scorpion venoms. Pulverized dried flowers are used as a remedy for jaundice. A mixture of crushed flowers and sugar is given to cure hemorrhoids and kidney stones. The boiled water extract of flowers is used to treat inflammation of nasal passages, as well as joint and muscle aches. A mixture of the flowers crushed with *dan-gyi* (*Tanacetum cinerariifolium*) leaves is applied to the soles of the feet and the palms of the hands to cure kidney stones. *Seed*: Known for imparting strength and energy. Pulverized to a powder, they are taken with milk to cure madness, as well as itches and rashes. The ash from burning a combination of the seeds and the bark from *hsu byu* (*Thevetia peruviana*) is mixed with jasmine oil and applied to the hair to promote growth and healthy texture. *Root*: Can be used as a diuretic.

###### Notes.

Medicinal uses of this species in India are discussed in Jain and DeFilipps (1991). Medicinal use of this species in China is discussed by Duke and Ayensu (1985).

###### Reference.

Agricultural Corporation (1980).

#### 5. *Chromolaena* DC.

##### Chromolaena odorata

Taxon classificationPlantaeORDOFAMILIA

(L.) R.M. King & H. Rob. (= Eupatorium odoratum L.)

###### Names.

**Myanmar**: *bezat*, *bizat*, *jamani-chon*, *taw-bizat*. **English**: buttefly-weed, jack in the bush, siamweed.

###### Range.

New World subtropics and tropics- Florida, Texas; Mexico; and West Indies. Pantropical weed. Widespread in Myanmar.

###### Use.

*Leaf*: Used to treat dysentery.

###### Notes.

In India the leaf is used to treat dysentery; also applied on fresh cuts and wounds to stop bleeding (Jain and DeFilipps 1991). The medicinal uses of this plant in the Caribbean region, as well as its chemistry, biological activity, toxicity and dosages, are discussed by Germosén-Robineau (1997). The chemistry, pharmacology, history and medicinal uses of this species in Latin America are discussed in detail by Gupta (1995). A pharmacological profile including medicinal uses of this plant in Africa is given in Iwu (1993).

An aqueous ethanol extract of the leaves of *C. odorata* were found to have antifungal activity. Chemical analysis of the extract and fractions showed the presence of biologically active constituents including some coumarines,flavonoids, phenols, tannins, and sterols. No toxic effect was noticed in the mice treated. (Ngono Ngane et al. 2006). Ethanol extracts of leaves of this species also showed antibacterial activites, inhibiting the growth of *Bacillus subtilis*, *Staphylococcus aureus*, and *Salmonella typhimurium*. The extract also was shown to reduce parasite number: antiprotozoal and cytotoxicity assays were done against *Trichomonas vaginalis* and *Blastocystis hominis*. Preliminary phytochemical screening showed the chemical compositon of the extracts to contain flavonoids, saponins, tannins and steroids (Vital and Rivera 2009).

###### Reference.

Nordal (1963).

#### 6. *Cyanthillium* Blume

##### Cyanthillium cinereum

Taxon classificationPlantaeORDOFAMILIA

(L.) H. Rob. (= Vernonia cinerea (L.) Less.)

###### Names.

**Myanmar**: *kadu-pyan*. **English**: little ironweed.

###### Range.

East, West-Central, West, and South tropical Africa; temperate and tropical Asia; and Australasia. Widely naturalized elsewhere. Widespread in Myanmar.

###### Uses.

*Whole plant*: Used as tonic and antiasthmatic.

###### Note.

In India, the whole plant is used as a diaphoretic “to remedy bladder spasms and strangury,” and in a decoction for promoting perspiration in fevers; plant juice is given for piles; the flower is used for conjunctivitis; the seed for as an alexipharmic and anthelmintic; and the root is used for dropsy (Jain and DeFilipps 1991).

###### Reference.

Nordal (1963).

#### 7. *Eclipta* L.

##### Eclipta prostrata

Taxon classificationPlantaeORDOFAMILIA

(L.) L. (= Eclipta alba (L.) Hassk)

###### Names.

**Myanmar**: *kyate-hman*, *kyeik-hman*. **English**: eclipta, false daisy, white eclipta, white heads, swamp daisy, yerba de tago.

###### Range.

North America (where flowers nearly year round, mostly summer to fall); Mexico; West Indies; Central America; South America; introduced in Asia, Africa, Pacific Islands, Australia, and Europe. Found growing naturally throughout Myanmar, rampantly like a weed in areas with much rain.

###### Conservation status.

Least Concern [LC] (IUCN 2017).

###### Uses.

Promotes vitality, health, and circulation; stimulates strong hair growth; used for respiratory illnesses, as well as for inflammation of eyes and other parts of the body. *Whole plant*: Used for asthma. Juice used as a tonic; in medicines for coughs, headaches, hepatitis, and inflammation of joint; in a poultice for skin disorders and sores; and as a black hair dye. Mixed with honey, the juice is given to children for coughs and colds. *Leaf*: Powder used to treat headaches, frontal baldness, boils and cysts, and venereal diseases. They are boiled with jaggery added to water, are reduced to one-third of the starting volume and taken to regulate menstrual periods. A mixture of the pulverized leaves and juice from *Vitex trifolia* is used to promote burn healing, prevent new scar tissue formation, and eliminate old scar tissue; mixed with milk they are consumed daily to improve vision and, it is said, to allow mute people to gain their voices, cause deaf people to hear, and stabilize shaky teeth; mixed with mother’s milk, they are given for intestinal worms, diarrhea, smallpox, chickenpox, and measles. A mixture of leaves with pulverized black sesame seeds is taken as a tonic to protect against diseases, promote longevity, and darken hair. Leaves crushed together with those from *Acalypha indica* and *Gardenia resinifera* are applied to the head to relieve congestion in children.

###### Notes.

The medicinal uses of this species (syn.: *E. prostata*) in India are discussed in Jain and DeFilipps (1991). Medicinal uses of this species in China are discussed in Duke and Ayensu (1985).

###### References.

Nordal (1963), Agricultural Corporation (1980), Forest Department (1999).

#### 8. *Elephantopus* L.

##### Elephantopus scaber

Taxon classificationPlantaeORDOFAMILIA

L.

###### Names.

**Myanmar**: *ka-tu-pin*, *ma-tu-pin*, *sin-che*. **English**: cucha cara, elephantopus, soft elephant’s-foot, yerba de caballo.

###### Range.

Tropical Africa, Eastern Asia, Indian Subcontinent, Southeast Asia, and Australia. In Myanmar, found in Magway, Mandalay, Sagaing, Shan, and Yangon.

###### Uses.

*Stem* and *Leaf*: A decoction made from these parts is used for menstrual disorders. *Root*: Used as an antipyretic, analgesic, and tonic.

###### Notes.

In Indian the leaf is used on cuts and to control vomiting; the root is used to check vomiting, for fever in children, on pimples, as an abortifacient, also for urinary problems, amoebic dysentery and other digestive disorders (Jain and DeFilipps 1991). Medicinal uses in other Asian countries follows: In China the plant is used to treat indigestion and swollen legs; in Taiwan the root is used to relieve pain in the chest; on the Malay Peninsula a decoction of the leaves is drunk to cure venereal diseases in women; in Indonesia the roots, either pounded in water or in decoction, are used as a remedy for leucorrhea, anemia in women and children, and during parturition; in the Philippines a decoction of the roots and leaves is used as an emollient, and leaves are heated and rubbed on the throat to relieve a bad cough; and in Guam the plant is used as a remedy for asthenic fever. Also, in Indo-China, Indonesia, and the Philippines, the plant is considered a diuretic and febrifuge; an infusion is taken to relieve anuria and blennorrhea and administered at parturition; a decoction of the whole plant is bechic, cleansing, and used to treat pulmonary diseases and scabies (Perry 1980).

The leaves contain a bitter principle; the plant has no alkaloid, but a white crystalline substance, apparently of glycoside nature, has been extracted. Also, an extract of the leaves has been shown to have antibiotic activity against *Staphylococcus* (Perry 1980).

###### References.

Nordal (1963), Perry (1980).

#### 9. *Emilia* Cass.

##### Emilia sonchifolia

Taxon classificationPlantaeORDOFAMILIA

(L.) DC. ex DC.

###### Names.

**English**: lilac tasselflower, red tasselflower.

###### Range.

Old World tropics; naturalized in southern Florida. In Myanmar, found in Mandalay and Yangon.

###### Uses.

*Whole plant*: Used as febrifuge, for eye diseases, and as an anthelmintic.

###### Notes.

The medicinal uses of this species in India are discussed in Jain and DeFilipps (1991) as follows: The leaf is used on wounds, bruises, and eye diseases; the root for diarrhea and gangrene (with leaf also). Medicinal uses of this species in China are discussed in Duke and Ayensu (1985). Here the plant is use as a detoxicant, diuretic, febrifuge, refrigerant, and sudorific. The whole plant is decocted for abscesses, boils, colds, dysentery, eneteritis, influenza, larengitis, numbness, pharyngitis, scales, snakebites, and traumatic injuries. The leaf is used for dysentery.

###### Reference.

Nordal (1963).

#### 10. *Enydra* Lour.

##### Enydra fluctuans

Taxon classificationPlantaeORDOFAMILIA

Lour.

###### Names.

**Myanmar**: *kana-hpaw*. **English**: marsh herb, water cress.

###### Range.

Occurs in both hemispheres from the Philippines, Indochina, and tropical Africa to Argentina, Brazil, Paraguay, Peru, Ecuador and Columbia. Introduced into Mexico. Found growing naturally at freshwater edges throughout Myanmar, except in very cold areas.

###### Conservation status.

Least Concern [LC] (IUCN 2017).

###### Uses.

*Whole plant*: All parts are used, but particularly the leaves. For edema, the plant’s five parts are boiled and eaten. The juice is given for pox-like diseases, skin problems, and disorders of the marrow and synovial fluids. A mixture of the juice with honey is taken for smallpox. To alleviate weak liver, the broth from the whole plant boiled together with rice, water, mustard oil, and a bit of salt is ingested. *Leaf*: Used in a steam bath. Preparations made from the leaves are also given for leprous sores, other skin disorders, coughing, and fever. Their juice can be taken with either cow’s or goat’s milk for urinary tract infections and associated limb heaviness.

###### Note.

In India the leaf is used as a laxative, demulcent, and is antibilious; it is also used for nervous conditions and the skin (Jain and DeFilipps 1991).

###### Reference.

Agricultural Corporation (1980).

#### 11. *Grangea* Adans.

##### Grangea maderaspatana

Taxon classificationPlantaeORDOFAMILIA

(L.) Poir.

###### Names.

**Myanmar**: *taw-ma-hnyo-lon*, *ye-tazwet*. **English**: madras wormwood.

###### Range.

Widespread in tropical and subtropical Africa, Madagascar, and Asia. In Myanmar, found in Bago and Yangon.

###### Conservation status.

Least Concern [LC] (IUCN 2017).

###### Uses.

*Leaf*: Used as anthelmintic, antipyretic, and antispasmodic.

###### Note.

Medicinal uses of this species in India are discussed in Jain and DeFilipps (1991) as follows: The leaf is used as an infusion and electuary for obstructed menses and hysteria, for anodye and antiseptic fomentations; also an antispasmodic, stomachic and deobstruent.

###### Reference.

Nordal (1963).

#### 12. *Senecio* L.

##### Senecio densiflorus

Taxon classificationPlantaeORDOFAMILIA

Wall.

###### Names.

**English**: butterweed, yellowtop.

###### Range.

China, Bhutan, India, Myanmar, Nepal, and Thailand. Widely distributed in Myanmar.

###### Uses.

*Leaf*: Used as emollient and maturant in boils.

###### Notes.

In India plant used in treating skin afflictions as follows: leaves ground and applied as paste on boils; decotion of aerial parts used as wash for burning sensations and gonorrhea (Begum and Nath 2000).

###### Reference.

Nordal (1963).

#### 13. *Sigesbeckia* L.

##### Sigesbeckia orientalis

Taxon classificationPlantaeORDOFAMILIA

L.

###### Names.

**English**: divine herb, Indian weed, sigesbeckia, yellow crown-head.

###### Range.

Africa, Asia, Australasia/ Pacific, naturalized in Madagascar. In Myanmar, found in Kachin, Mandalay, Sagaing, and Shan.

###### Uses.

*Whole plant*: Used for treating skin diseases and as a stimulant.

###### Notes.

The medicinal uses of this species in India are discussed in Jain and DeFilipps (1991) as follows: A tincture of the (whole) plant with glycerine is used for ringworm and other skin disease, ulcers, and sores; as a diaphoretic and cardiotonic; also for renal colic and rheumatism. Medicinal uses of this species in China are discussed in Duke and Ayensu (1985). Here the whole plant is used for arthritis, a bad back, boils, dermatitis, hemiplegia, hypertension, leg ache, rheumatism, side ache, sciatica, and weak knees. It is ground and taken alone or with other plants for convulsions, paralytic stroke, and rheumatoid arthritis. It is also used for insect, dog, tiger, and snakebites, and ulcers. Additionally, it is decocted for malignant tumors, malaria, and numbness. The root is used externally for abscesses.

The plant has a hypoglycemic property (Jain and DeFilipps 1991). The root contains an essential oil, a substance suggesting salicylic acid, and a bitter glycoside (darutosdie). Also, extracts are said to have antiviral, hypoglycemic, and insecticidal properties (Duke and Ayensu 1985).

###### Reference.

Nordal (1963).

#### 14. *Tagetes* L.

##### Tagetes erecta

Taxon classificationPlantaeORDOFAMILIA

L.

###### Names.

**Myanmar**: *dewali-pan*, *kala-pan*. **English**: African marigold, Aztec marigold, marigold.

###### Range.

Mexico and Central America. Cultivated in Myanmar.

###### Uses.

*Leaf*: Used as an analgesic and antiseptic.

###### Notes.

Medicinal uses of this species in India are discussed in Jain and DeFilipps (1991) as follows: The leaf is applied to carbuncles ad boils; leaf juice is used for earache; the flower is used as a remedy for eye diseases and ulcers; flower juice is used for bleeding piles; flowers are also taken as a blood purifier. Medicinal uses of this species in China are discussed by Duke and Ayensu (1985). Here the leaf is used to treat sores and ulcers; the flower heads are decocted for colds, conjunctivitis, cough, mastitis, mumps, and sore eyes; they are also cooked with chicken liver to improve vision.

###### Reference.

Nordal (1963).

#### 15. *Tanacetum* L.

##### Tanacetum cinerariifolium

Taxon classificationPlantaeORDOFAMILIA

(Trevir.) Sch. Bip. (= Chrysanthemum cinerariifolium (Trevir.) Vis.)

###### Names.

**Myanmar**: *hsay gandamar*. **English**: dancing daisy, pyrethrum.

###### Range.

Subtropical, temperate. In Myanmar, prefers temperate climates and can be cultivated at up to 1065–2135 m in altitude; thrives in Chin State, Shan State, Kachin State, Kokang area, Wa area, Naga hills, Mogok, Kyatpyin and Pyin Oo Lwin.

###### Conservation status.

Least Concern [LC] (IUCN 2017).

###### Uses.

Stimulates appetite and heart functioning. *Leaf*: Crushed and mixed with black pepper, they are taken for urination problems. They are also used to treat cracked lips, gonorrhea, vomiting, and bleeding. *Flower*: Antiparasitic; used in pesticides and repellents effective against the mosquito vectors of dengue hemorrhagic fever and vectors of other infectious diseases.

###### Notes.

The species is used as an insecticide. The old Chinese use of the genus *Chrysanthemum* was to treat “liver weakness”, clarify vision, and act as a circulatory tonic. The present use is to “benefit the blood”; treat minor infection; and for digestive, circulatory, and nervous disorders as well as for menstrual disorders and night blindness (Perry 1980).

###### References.

Nordal (1963), Agricultural Corporation (1980).

### Basellaceae (Malabar Spinach family)

#### 1. *Basella* L.

##### Basella alba

Taxon classificationPlantaeORDOFAMILIA

L. (= Basella rubra L.)

###### Names.

**Myanmar**: *kin peint*, *ginbeik*. **English**: Indian spinach.

###### Range.

Asia and Africa. Found growing naturally in Myanmar’s hot regions (such as Bago and Mandalay).

###### Uses.

*Whole plant*: A decoction is used to alleviate labor during childbirth. *Flower*: Used as an antidote to poisons. *Leaf*: Juice and paste from the crushed leaves is applied to sores to promote healing. The juice is also ingested to relieve diarrhea, fever, and urinary tract infections. *Root*: Boiled in water and consumed to alleviate vomiting associated with the gall bladder problems.

###### Note.

Medicinal uses of this species in India are discussed in Jain and DeFilipps (1991).

###### References.

Agricultural Corporation (1980), Duke and Ayensu (1985), Forest Department (1999), Manandhar and Manandhar (2002).

### Berberidaceae (Barberry family)

#### 1. *Berberis* L.

##### Berberis nepalensis

Taxon classificationPlantaeORDOFAMILIA

Spreng.

###### Names.

**Myanmar**: *khaing-shwe-wa*, *khine-shwe-war*. **English**: mahonia.

###### Range.

Eastern Asia. Cultivated in Myanmar.

###### Use.

*Fruit*: Berries used as diuretic.

###### Notes.

In India the fruit is employed as a diuretic and demulcent, also edible; the root “extract yields a product ‘rasaut’ with the same properties as *Berberis*.” (Jain and DeFilipps 1991). A decoction of the bark is used for eyedrops to treat inflammation of the eyes (Manandhar and Manandhar 2002). The fruit is used in the treatment of dysentery (Chopra et al. 1986).

Berberine, present in the rhizomes, has been shown to have a marked antibacterial effect and is used as a bitter tonic. It is used orally in the treatment of various enteric infections, especially bacterial dysentery. Berberine has also been shown to have antitumor activity (Duke and Ayensu 1985).

###### Reference.

Nordal (1963).

### Betulaceae (Birch family)

#### 1. *Alnus* Mill.

##### Alnus nepalensis

Taxon classificationPlantaeORDOFAMILIA

D.Don

###### Names.

**Myanmar**: *hyang*, *mai-bau*, *nbau*, *ning-bau*, *yang-bau*. **English**: alder.

###### Range.

Eastern Himalayas and western China. In Myanmar, found in Chin and Kachin.

###### Conservation status.

Least Concern [LC] (IUCN 2017).

###### Use.

*Bark*: Used as an astringent.

###### Notes.

In India, the bark is used to treat dysentery and stomachache; the leaf is employed on cuts and wounds; and the root is used for diarrhea (Jain and DeFilipps 1991).

###### Reference.

Nordal (1963).

### Bignoniaceae (Catalpa family)

#### 1. *Markhamia* Seem. ex Baill.

##### Markhamia stipulata

Taxon classificationPlantaeORDOFAMILIA

(Wall.) Seem.

###### Names.

**Myanmar**: *kwe*, *ma-hlwa*, *mai-kye*, *mayu-de*, *pauk-kyn*. **English**: Asian markhamia.

###### Range.

China, Cambodia, Laos, Myanmar, Thailand, and Vietnam. Widely distributed in Myanmar.

###### Use.

Plant used as a cure for psora.

###### Note.

Phenolic glycosides have been found in this species as follows: five verbascoside derivatives (markhamiosides A-E) and one hydroquinone (markhamioside F) were isolated together with 13 known compounds from the leaves and branches of this species (Kanchanapoom et al. 2002).

###### Reference.

Perry (1980).

#### 2. *Mayodendron* Kurz

##### Mayodendron igneum

Taxon classificationPlantaeORDOFAMILIA

(Kurz) Kurz

###### Names.

**Myanmar**: *egayit*, *egayit-ni*, *hpun-hpawk*, *mai-pyit*, *sumhtung*, *sumtungh-kyeng*. **English**: *peepthong*.

###### Range.

China, Taiwan, Laos, Myanmar, Thailand, and Vietnam. Widely distributed in Myanmar.

###### Use.

*Bark*: Used as antidote in alcohol poisoning.

###### Notes.

An ethanal extract of the leaves of this species was found to exhibit significant anti-inflammatory and analgesic activites (Hashem et al. 2007).

###### Reference.

Nordal (1963).

#### 3. *Oroxylum* Vent.

##### Oroxylum indicum

Taxon classificationPlantaeORDOFAMILIA

(L.) Kurz

###### Names.

**Myanmar**: *kyaung shar*, *sot-gren-itg* (Mon), *maleinka* (Mak) (Shan). **English**: Indian trumpet flower.

###### Range.

Subtropical and tropical. Found from India to tropical China, south into Southeast Asia. Found growing naturally throughout Myanmar up to 1220 m altitude.

###### Uses.

*Bark*: A mixture of the bark powder with the juice of ginger and honey is given for asthma and bronchitis. The filtered liquid made from this powder is soaked in hot water for 2 hours and taken morning and night for chronic indigestion. The water from soaked bark is used as a mouthwash to relieve dry throat and cracked skin around the mouth. Bark of trunk and root used as an astringent and a tonic in dysentery, diarrhea, and rheumatism. *Leaf*: The juice is taken as a remedy for opium toxicity. Leaves are boiled and eaten to stimulate bowel movements. *Fruit*: Boiled or roasted, it is taken for indigestion, goiter, flatulence and hemorrhoids. It is eaten in a salad to alleviate boils on the skin. A mixture of fruit cooked with chicken is eaten to cure asthma. Consuming the fruit cooked with banded snakehead fish (*Ophiocephalus striatus*) is considered a cure for cholera that gives vitality as well as curing indigestion and diarrhea. As a remedy for palpitations or fatigue brought on by a weak heart, a mixture of fruit cooked with prawns is eaten. To reduce edema, increase weight, and strengthen a weak heart, a mixture of the fruit and hilsa fish (*Hilsa ilisha*) is eaten. A combination of the fruit cooked with the fish *nga-mway-toh* (*Mastacembelus armatus*) is ingested to cure dysentery associated with weakness in men and menstruation in women, as well as hemorrhoids. *Root*: A paste formed from grinding is applied to treat sores that continue to fester even though the skin has healed. Root bark is used to treat fever, joint pain, stomach bloating, and stomach pain.

###### Notes.

Medicinal uses of this species in India are discussed in Jain and DeFilipps (1991). Medicinal use of this species in China is discussed by Duke and Ayensu (1985).

In Indo-China and the Philippines the bark of the trunk and root are used in the same way as in Myanmar. On the Malay Peninsula the bark is used for dysentery. A decoction of the leaves is drunk for stomach disorders, rheumatism, and wounds; and is made into hot fomentations to treat cholera, fever, and rheumatic swellings. The cooked leaves are used as poultices for various ailments during and after childbirth; also for dysentery, and to relieve headache and toothache. In Indonesia the bitter bark serves as a remedy for stomach problems, and also as a tonic and appetizer. Additionally, the bark is chewed as a depurative, especially after parturition. The flowers are used as a remedy for inflammation of the eyes. The pith serves as a styptic. In the Philippines the juice from the crushed bark is rubbed on the back to relieve the ache accompanying malaria (Perry 1980).

Oroxylin, isolated from the bark and seeds, has been found to be a mixture of three flavones, baicalein, 6-methylbaicalein, and chrysin. Oroxylin-A consists of phtalic and benzoic acids, and phloroglucinol (Perry 1980).

###### References.

Agricultural Corporation (1980), Perry (1980), Forest Department (1999).

#### 4. *Millingtonia* L.f.

##### Millingtonia hortensis

Taxon classificationPlantaeORDOFAMILIA

L.f.

###### Names.

**Myanmar**: *mai-long-ka-hkam*, *sum-tung-hpraw*, *htamone-chort*. **English**: jasmine tree, Indian cork tree.

###### Range.

Cambodia, Laos, Myanmar, Thailand, Vietnam; commonly cultivated throughout India, Indonesia, and Malaysia, occasionally naturalized. Found growing naturally all over Myanmar, except in cold areas.

###### Use.

*Leaf*: Boiled in water and eaten, or made into a stir-fry, for menstruation and hypertension. *Flower* and *Shoot*: Drinking a soup made with the flowers or eating the shoots will cure hypertension and heart palpitations. *Root*: Taking the paste of the root after adding salt or sugar will cure heart palpitations and dizziness; drawing circles around the eyes with a paste made from the root and bark will cure sore eyes; applying a paste made from the root will cure gas disorders; drinking the liquid in which the fresh root has been boiled with jaggery will cure vitiligo; rubbing a paste of the root or bark onto the tongue will cure alcoholic intoxication.

###### References.

Agricultural Corporation (1980), Forest Department (1999).

#### 5. *Stereospermum* Cham.

##### Stereospermum chelonoides

Taxon classificationPlantaeORDOFAMILIA

(L.f.) DC.

###### Names.

**English**: fragrant padri-tree, padri, yellow snakeroot.

###### Range.

India to the Malay Peninsula.

###### Use.

*Leaf*, *Flower*, and *Root*: Used as a febrifuge.

###### Notes.

In India the bark is tonic, diuretic; used for stomachache, cholera, malaria, and liver problems. The root is used for chest and brain afflictions, also intermittent and puerperal fevers (Jain and DeFilipps 1991). The leaves, flowers, and roots are used as a febrifuge in Indo-China (except Vietnam) (Perry 1980).

###### Reference.

Perry (1980).

##### Stereospermum colais

Taxon classificationPlantaeORDOFAMILIA

(Buch.-Ham. ex Dillwyn) Mabb. (= Stereospermum tetragonum DC.)

###### Names.

**Myanmar**: *hingut-pho*, *hingut-po*, *kywe-ma-gyo-lein*, *sin-gwe*, *thakut-pho*, *thakut-po*, *thande*, *than-tat*, *than-tay*. **English**: trumpet flower, yellow snake tree.

###### Range.

China, Bangladesh, Bhutan, Cambodia, India, Indonesia, Laos, Malaysia, Myanmar, Nepal, Sikkim, Sri Lanka, Thailand, and Vietnam. Widespread in Myanmar.

###### Use.

*Leaf*, *Flower*, *Root*: Used as febrifuge.

###### Note.

In India the leaf is used for dyspepsia; the root for asthma, cough, and excessive thirst (Jain and DeFilipps 1991).

###### Reference.

Nordal (1963).

#### 6. *Tecoma* Juss.

##### Tecoma stans

Taxon classificationPlantaeORDOFAMILIA

(L.) Juss. ex Kunth (= Tecomella stans Seem)

###### Names.

**Myanmar**: *sein-takyu*. **English**: trumpet-bush, yellow-bells, yellow-elder, yellow trumpet-bush.

###### Range.

New World tropics.

###### Uses.

*Bark*: Utilized as an antisyphilitic and as an antidote in alcohol poisoning. *Leaf*: Used for hypoglycemic properties.

###### Notes.

Reported uses of the species include stomachache, alcoholism, atony, biliousness, diabetes, diuretic, dysentery, gastritis, inappetence, indigestion, intoxicant, pain, stomachic, syphilis, tonic, and vermifuge (Duke 2009). In India the root is used to treat scorpion sting; also snake and rat bite (Jain and DeFilipps 1991).

Pods of *T. stans* have been shown to contain tecomine and tecostanine, which have the effect of lowering blood sugar levels (Lan et al. 1998). Research has provided evidence that the main antidiabetic effect of the aqueous extract is due to intestinal a-glucosidase inhibition by decreasing the postprandial hyper-glycaemia peak. Additionally, the aqueous extract sub-chronic administration was found to reduce triglycerides and cholesterol without modifying fasting glucose (Anguilar-Santamaría et al. 2009).

###### References.

Nordal (1963), Mya Bwin and Sein Gwan (1967).

### Bixaceae (Annatto family)

#### 1. *Bixa* L.

##### Bixa orellana

Taxon classificationPlantaeORDOFAMILIA

L.

###### Names.

**Myanmar**: *thinbaw-tidin*. **English**: achiote, annatto, lipstick-tree.

###### Range.

Tropical America.

###### Uses.

*Seed*: Used as a febrifuge and astringent.

###### Notes.

Medicinal uses of this species in India are discussed in Jain and DeFilipps (1991). Indigenous medicinal uses of this species in the Andaman and Nicobar Islands (India) are described by Dagar and Singh (1999).

The medicinal uses of this plant in the Caribbean region, as well as its chemistry, biological activity, toxicity, and dosages, are discussed by Germosèn-Robineau (1997). The chemistry, pharmacology, history and medicinal uses of this species in Latin America are discussed in detail by Gupta (1995). The red dye from the seed arils contains a mixture of stereoisomers of bixin, a C-24 diapocarotenoid [having purgative action (Lan et al. 1998)]; and, the leaf-oil is a rich source of numerous terpenes (Mors et al. 2000).

###### Reference.

Nordal (1963).

### Boraginaceae (Heliotrope family)

#### 1. *Cordia* L.

##### Cordia dichotoma

Taxon classificationPlantaeORDOFAMILIA

G.Forst.

###### Names.

**Myanmar**: *hpak-mong*, *kal*, *kasondeh*, *thanat*, *thanut*, *tun-paw-man*. **English**: Sebastian tree.

###### Range.

Southern China, Taiwan south to northeastern Australia and New Caledonia. In Myanmar, found in Mandalay, Shan, and Yangon.

###### Uses.

*Fruit*: Cooling, anthelmintic, diuretic, purgative, and expectorant. *Bark*: Used to treat catarrh.

###### Notes.

The medicinal uses of this species in India are discussed in Jain and DeFilipps (1991) as follows: The leaf is used for cough, cold, fever, and ulcers; the fruit as an expectorant, and for stomachache, lung and urinary disease. Perry (1980) discusses the medicinal uses of the species in China, Hainan, Indo-China, Indonesia, and the Philippines.

###### References.

Nordal (1963), Perry (1980).

##### Cordia myxa

Taxon classificationPlantaeORDOFAMILIA

L.

###### Names.

**Myanmar**: *taung-thanut*, *thanat*. **English**: Assyrian plum, clammy cherry, Indian cherry, sapistan, Sebesten plum, selu.

###### Range.

India to Australia. In Myanmar, found in Mandalay, Taninthayi, and Yangon.

###### Uses.

*Leaf*: Used in manufacture of “Burmese cheroots.”

###### Notes.

The fruit of this species is used throughout its range for its sticky mucilaginous pulp which is eaten to suppress cough, for chest complaints, to treat a sore throat, and as a demulcent; also applied as an emollient to mature abscesses, to calm rheumatic pain, and as an anthelmintic. In Tanzania the fruit pulp is applied on ringworm. In Mali and the Ivory Coast the leaves are applied to wounds and ulcers. A macerate of the leaves is taken to treat trypanosomiasis, and is externally applied as a lotion to tse-tse fly bites. In the Comoros the powdered bark is applied to the skin in cases of broken bones before a plaster is applied, to improve healing. Bark powder is used externally in the treatment of skin disease; bark juice, together with coconut oil, is taken to treat colic.

Chemical screening of both leaves and fruits shows that pyrrolizidine alkaloids, coumarins, flavonoids, saponins, terpenes, and sterols are present. The principle fatty acids in the seed are palmitic, stearic, arachidic, behenic, oleic, and linoleic. Petroleum ether and alcoholic extracts shows significant analgesic, anti-inflammatoroy, and anti-arthritic activities is tests with rats. Four flavonoid glycosides, a flavonoid aglycone, and two phenolic derivatives were isolated. Ethanol extracts from fruits and leaves show significant antioxidant activities due to the carotenoids, but no antidmicrobial activity against bacteria (Oudhia 2007).

###### Reference.

Nordal (1963).

#### 2. *Heliotropium* L.

##### Heliotropium indicum

Taxon classificationPlantaeORDOFAMILIA

L.

###### Names.

**Myanmar**: *sin-hna-maung*, *sin-let-maung.* **English**: Indian heliotrope, turnsole.

###### Range.

Pantropical. In Myanmar, found in Yangon.

###### Uses.

*Whole plant*: Used as diuretic. A decoction used in treating gonorrhea; one is also used for the treatment of diabetes by Kawkareik inhabitants. *Leaf*: Applied to boils, ulcers, and wounds.

###### Notes.

In India the whole plant is used for ulcers, boils, insect bites, and throat infection; the leaf for insect and reptile bites (Jain and DeFilipps 1991). In China the plant is widely used for poulticing, boils, carbuncles, and herpes; also anti-cancer (Duke and Ayensu 1985). Perry (1980) discusses the medicinal uses of the species in China, Indo-China, the Malay Peninsula, and the Philippines.

The species contains an important anti-cancer ingredient, indicine-N-oxide, which shows significant activity against the P388 leukemia. “It is also active against the B16 melanoma, L1210 leukemia, and Walker 256” and “in 1976, no negative histopathologyic findings indicative of the heptotoxicology usually associated with pyrrolizidine alkaloids, had been demonstrated for indicine-N-oxide.” Also, acetyl indicine, indicinine, and indicinine have been reported for this species (Duke and Ayensu 1985).

###### References.

Mya Bwin and Sein Gwan (1967), Perry (1980).

### Brassicaceae (Mustard family)

#### 1. *Brassica* L.

##### Brassica oleracea

Taxon classificationPlantaeORDOFAMILIA

L.

###### Names.

**Myanmar**: *kobi-dok*. **English**: cabbage, kohlrabi, wild cabbage.

###### Range.

Native to western Europe; cultivated worldwide.

###### Conservation status.

Data Deficient [DD] (IUCN 2017).

###### Uses.

*Leaf*: Used in the treatment of skin diseases as well as in diuretic and laxative preparations. *Seed*: Used to promote appetite and digestion; also used as a diuretic and laxative.

###### Notes.

Medicinal uses of this species in India are discussed in Jain and DeFilipps (1991). Details of the active chemical compounds, effects, herbal usage and pharmacological literature for this plant are given in Fleming (2000).

###### Reference.

Nordal (1963).

#### 2. *Sinapis* L.

##### Sinapis alba

Taxon classificationPlantaeORDOFAMILIA

L. (= Brassica alba (L.) Rabenh.)

###### Names.

**Myanmar**: *chying-hkrang-ahpraw*, *antamray*, *rai baitine*. **English**: Chinese mustard, white mustard.

###### Range.

North Africa, Europe, Southwest and Central Asia; widely introduced. Cultivated in Myanmar.

###### Use.

Hot and bitter in taste with heating properties, effective, aids digestion, calms the phlegm, cures vomiting of blood, passing of blood, leprosy, itching and rashes. *Seed*: A paste made from mixing the seeds together with *kunsar-gamone* (*Alpinia galanga*) can be rubbed on to cure inflammation of the joints. *Oil*: A small amount of the oil can be poured into the ear to cure earaches. Cook oil, the juice from *mayoe* (*Calotropis procera*) leaves, and some turmeric rhizome together and filter out the oil, which can then be rubbed on to cure skin diseases like ringworm, and itching. Cooking oil with menthol will produce a rub to use for children getting stomachaches, catching chest colds, and coughs and colds. The oil can be rubbed on directly to afflicted areas to cure enlarged spleen, cysts and tumors, edema, hemorrhoids, flatulence and shooting abdominal pains. Applying a small amount of the oil into the nostrils at bedtime will cure sinusitis. The oil can be applied on the nape of the neck to cure a stiff neck or across the bridge of the nose and along the brow line to cure aching eyes. An ointment can be made by mixing one part of mustard oil and one part of sesame oil with mountain goat or wild goat lard, which can be used to cure numbness, muscular spasms, and cramps.

###### Reference.

Agricultural Corporation (1980).

### Burseraceae (Gumbo Limbo family)

#### 1. *Garuga* Roxb.

##### Garuga pinnata

Taxon classificationPlantaeORDOFAMILIA

Roxb.

###### Names.

**Myanmar**: *chinyok*, *mai-kham*, *sinyok*, *taesap*. **English**: garuga.

###### Range.

China, East Pakistan, Bangladesh, Cambodia, India, Laos, Myanmar, Thailand, Vietnam, Malaya, and the Philippines. In Myanmar, found in Bago, Mandalay, and Rakhine.

###### Use.

*Juice*: Used to treat asthma.

###### Notes.

The medicinal uses of this species in India are discussed in Jain and DeFilipps (1991) as follows: Juice from the stem is used in an eye-drop for opaque conjunctiva; leaf juice mixed with honey is used for asthma; the fruit is used as a stomachic. In Indo-China the bark is used with honey to treat asthma (Perry 1980).

###### Reference.

Perry (1980).

### Calophyllaceae (Calophyllum family)

#### 1. *Calophyllum* L.

##### Calophyllum inophyllum

Taxon classificationPlantaeORDOFAMILIA

L.

###### Names.

**Myanmar**: *ponenyet*. **English**: Alexandrian laurel, Indian laurel, laurel-wood.

###### Range.

Africa, temperate and tropical Asia, Australasia, and Pacific. Found growing naturally in lower Myanmar, but also thrives well in coastal areas with hot and wet climates. It is cultivated in some areas.

###### Conservation status.

Lower Risk/least concern [LC] (IUCN 2017).

###### Uses.

*Whole plant*: Preparations made from the five parts used to regulate bile and phlegm, as well as to bind the blood. *Leaf*: Water from soaking the leaves is used for eye drops to alleviate burning. *Bark*: Liquid from boiling the bark is taken to relieve constipation and to stop hemorrhaging. Sap extracted from the bark is used to compound medicines for treating wounds and sores. *Seed*: Oil extracted from the seeds is used to make remedies for aches, pains, gonorrhea, leprosy, and other skin diseases.

###### Note.

The medicinal uses of this species in India are discussed in Jain and DeFilipps (1991).

###### Reference.

Agricultural Corporation (1980).

#### 2. *Mesua* L.

##### Mesua ferrea

Taxon classificationPlantaeORDOFAMILIA

L.

###### Names.

**Myanmar**: *guntgaw*, *gau-gau*, *maiting* (My) (Kachin), *kaw-ta-nook* (Kayin), *ar ganui* (Mon), *jai-nool* (Mon), *kam kan* (Mai) (Shan). **English**: Ceylon ironwood, cobra’s saffron, Indian rose-chestnut, ironwood tree.

###### Range.

Tropical Asia, India. Found throughout Myanmar, but especially in Tanintharyi Division, growing naturally in tropical evergreen forests up to altitudes of 1065 m; also grown in gardens for ornamental purposes.

###### Uses.

*Whole plant*: Flowers, stamens, seeds, roots, bark and oils are made into preparations to support digestion, improve complexion, cure blood disorders, reduce edema, neutralize poisoning, and alleviate heart and bladder pains. *Leaf*: Used to treat snakebites. *Bark*, *Root*: Used in tonics taken for strength. *Flower*: Used as an astringent. A mixture of the flowers with butter and sugar is taken for burning sensations in the body and for hemorrhoids. Flowers are used in medicines that neutralize toxins for cases of poisoning and for venomous bites and stings; dried, they are used in treatments for coughs, stomach problems, and excessive perspiration and phlegm. The anthers are used in remedies for fevers and excessive menstrual bleeding. A mixture of crushed anthers and rock sugar rolled with top oil (liquid that rises to top when slow-cooking substances, such as butter, etc.) is used to treat hemorrhoids and cracked skin on the soles of the feet. Ground together with *thanakha* (*Hesperethusa crenulata*) they form a paste used topically on boils and other skin conditions. *Seed*: Their oil is used as an ointment to treat inflammation of joints and as a remedy for scabies, eczema, and other skin problems, including infected sores.

###### Notes.

The medicinal uses of this species in India are discussed in Jain and DeFilipps (1991). Perry (1980) discusses the medicinal uses of this species on the Malay Peninsula and in Indonesia.

###### References.

Nordal (1963), Agricultural Corporation (1980), Perry (1980), Ministry of Health (2001).

### Cannabaceae (Hemp family)

#### 1. *Cannabis* L.

##### Cannabis sativa

Taxon classificationPlantaeORDOFAMILIA

L.

###### Names.

**Myanmar**: *bhang*, *se-gyauk*. **English**: grass, gallow grass, marihuana, pot, red-root, soft hemp, true hemp.

###### Range.

Asia. Cultivated in Myanmar.

###### Uses.

*Whole plant*: Intoxicant, analgesic, sedative, and anodyne.

###### Notes.

The medicinal uses of this species in India are discussed in Jain and DeFilipps (1991). Medicinal uses of this species in China are discussed in Duke and Ayensu (1985). Perry (1980) discusses the general uses of the species in eastern and southeastern Asia (including Myanmar). Especially in China and Indo-China, all parts of the plant are used. The seeds are used as tonic, alterative, emmenagogue, laxative, demulcent, diuretic, anththelmintic, narcotic, and anodyne; also they are prescribed in fluxes, for post partum problems, obstinate vomiting, and used externally on eruptions, ulcers, wounds, and favus. The plant is also “considered of great value in treating tetanus. It is a true sedative of the stomach, used to treat dyspepsia with painful symptom, cancers, ulcers; also to treat migraine, neuralgia, and rheumatism. After special preparation, the seeds are prescribed for uterine prolapse, to aid parturition, and as a febrifuge.”

The flowering twigs contain an essence of sesquiterpene, cannabin, solid alcohol, and hydrate of cannabin. Contents of the seeds include protein, lipids, choline, trigonlline, xylose, inosite, many acids and enzymes, phosphates, and phytosterols. Two active substances found in the resin are cannabinol and cannabidiol, *both toxic* (Perry 1980).

###### Reference.

Nordal (1963).

### Cannaceae (Canna family)

#### 1. *Canna* L.

##### Canna indica

Taxon classificationPlantaeORDOFAMILIA

L.

###### Names.

**Myanmar**: *budatharana*, *ar-do*, *adalut.* **English**: canna, Indian shot, Queesland arrowroot.

###### Range.

Tropical America. Found growing throughout Myanmar; also cultivated.

###### Uses.

*Sap*: Aids in regulating bowels and healing sores. *Rhizome*: Employed as a diaphoretic, demulcent, and to treat fever and dropsy. Thinly sliced, dried, made into a preserve with jaggery (sugar made from juice of the toddy palm, *Borassus flabellifer*, inflorescence), and stored in a glass jar after adding the powder of five kinds of spices (names not specified in Agricultural Corporation 1980); then ball the size of a betel (*Piper betle*) nut eaten every morning and evening to treat male and female disorders, imbalance in the blood, diarrhea, menopause symptoms, insufficient blood circulation, hemorrhoids, impotence, poor complexion, loss of strength, backache, general aches and pains, and jaundice. About half a cup of the liquid in which the rhizome has been boiled together with raw sugar, taken once in the morning and one at night, used to treat menstrual disorders, stiffness in the ligaments and tendons, bloated stomach, and urinary tract disease. *Flower* and *Fruit*: Young flowers and fruits, lightly boiled in water and eaten with a dip or in a salad, used to treat too little urine and difficulty in passing urine; also to treat a fever. Eating a curry into which liquid from boiling the flowers has been added during cooking is used to treat a stiff neck, stiffness in the fingers and toes, and backache, as well as mucus in the stool, diarrhea, and loss of appetite. *Root*: Taking about a quarter cup of the liquid in which the roots have been boiled after adding some roasted salt, used to treat fever, sore throat, and mucus in the respiratory system; about a half cup of liquid in which the roots have been boiled together with jaggery, used to treat edema, body aches, and sharp spasmodic pain in the bowels.

###### Note.

Medicinal uses of this species in China are discussed in Duke and Ayensu (1985).

###### References.

Nordal (1963), Agricultural Corporation (1980).

### Capparaceae (Caper family)

#### 1. *Capparis* L.

##### Capparis flavicans

Taxon classificationPlantaeORDOFAMILIA

Kurz

###### Names.

**Myanmar**: *saungkyan*, *saung-chan*. **English**: caper bush.

###### Range.

Myanmar, Vietnam, Cambodia, Laos, and Thailand. In Myanmar, found in Magway, Mandalay, and Sagaing.

###### Use.

*Leaf*: Used as a galactagogue.

###### Reference.

Perry (1980).

##### Capparis zeylanica

Taxon classificationPlantaeORDOFAMILIA

L.

###### Names.

**Myanmar**: *mai-nam-lawt*, *mani-thanl-yet*, *nwamni-than-lyet*. **English**: Ceylon caper.

###### Range.

India to Indo-China, East Java, the Lesser Sunda Islands, and the Philippines. In Myanmar, found in Magway, Mandalay and Shan.

###### Uses.

*Bark*: Used to treat cholera. *Leaf*: Used as a counter-irritant. *Root*: Applied to sores. *Root Bark*: Used to as a stomachic.

###### Notes.

In the Philippines the leaves are used as a counter-irritant; additionally, the leaves (rubbed with salt and sometimes pounded) are used on the forehead and/or the temples as a remedy for headache. In Indo-China the plant is used for the same stimulant properties as the Cruciferae, also used as an antiscorbutic and for gastritis (Perry 1980).

Reported constituents include alkaloid, phytosterol, mucilaginous substance, and water-soluble acid (Perry 1980).

###### Reference.

Perry (1980).

#### 2. *Crateva* L.

##### Crateva religiosa

Taxon classificationPlantaeORDOFAMILIA

G.Forst.

###### Names.

**Myanmar**: *lè-seik-shin*. **English**: sacred garlic pear.

###### Range.

India to Indo-China and the Ryukyus, south through Moluccas and New Guinea, east to Polynesia. Reported from Myanmar.

###### Use.

*Bark*: A paste from grinding the bark together with *paranawar* (*Boerhavia diffusa*) root is taken to cure chronic sores and boils. *Leaf*: Crushed, mixed with water and warmed, is applied to areas with aches and pain. The juice from the crushed leaves can be mixed in equal amounts with crushed betel (*Piper betle*) leaves and butter and the mixture is taken to cure inflammation of the joints. The leaves can be pickled and eaten with a fish paste or fish sauce dip or as a salad to cure gas and digestion problems. *Flower*: Pickled and eaten as a stomachic. *Root*: Boiled in water until reduced to one fourth, and taken to treat diabetes and kidney stones. If cane sugar is added to this liquid and drunk, it can cure inflammation of the bladder and kidney stones. Also used to treat high fevers.

###### Notes.

In China the leaf is used as a tonic, stomachic, resolvent; also used for dysentery, headache, and stomachache (Duke and Ayensu 1985). In Taiwan a decoction of the stem and leaves is used to treat dysentery, headache, and stomachache; in China the leaves are considered to be stomachic; in Indo-China the leaves are used as a tonic and resolutive; in the Soloman Islands the liquid from the bark macerated with water is used to treat constipation and heated leaves are applied as a remedy for earache (Perry 1980).

Reported constituents of the bark include lupeol (a triterpene) and beta-sitosterol. (Perry 1980, Duke and Ayensu 1985). The leaves contain calcium, phosphorus, iron, beta-carotene equivalent, thiamine, riboflavin, niacin, and ascorbic acid (Duke and Ayensu 1985).

###### References.

Agricultural Corporation (1980), Perry (1980).

### Caricaceae (Papaya family)

#### 1. *Carica* L.

##### Carica papaya

Taxon classificationPlantaeORDOFAMILIA

L.

###### Names.

**Myanmar**: *thinbaw*, *sang-hpaw*, *shanghpaw*, *shang hap-wsi* (Kachin), *mansi* (Chin), *crot-kyeei*, *hla-crote kyee* (Mon), *mak-sang-hpaw* (Shan). **English**: papaw, papaya, pawpaw.

###### Range.

Tropical America. Cultivated in Myanmar.

###### Conservation status.

Data Deficient [DD] (IUCN 2017).

###### Uses.

Known for binding and heating properties, the fruit, seeds, sap, leaves, and roots are used. *Leaf*: A mixture of the juice from crushed leaves and a small amount of opium is used to relieve muscle stiffness. Leaves blanched in hot water or wilted over heat are applied to affected body parts to relieve aches and pains of menstruation. Roasted leaves with a fish paste or fish sauce dip are prepared in a lepet [tea leaves steamed, pressed, fermented, mixed with oil (usually peanut oil); this added to salad] salad to alleviate buzzing in the ear and other ear problems. *Fruit*: Sweet and easily digestible ripe fruit stimulates hunger, facilitates digestion, promotes healthy urinary function, increases phlegm, benefits the heart, cleanses the blood, calms the bile, and protects against urinary diseases and gallstones. It promotes health and longevity, and protects against diseases. Soaking the fruit in water and taking the liquid three times daily alleviates enlargement of the spleen; eating the ripe fruit also alleviates enlargement of the spleen, as well as enlargement of the liver and hemorrhoids. Nearly ripe but still firm fruit is eaten cooked or in a salad to encourage healthy bowel and urinary functioning. A small amount of powder made from the dried, young fruit is used to alleviate chronic diarrhea. Juice from cut green fruit is applied to scorpion sting to neutralize the poison. The young fruit dipped in salt is eaten as a remedy for diphtheria. Children are given a small amount of the fruit sap together with milk or for indigestion. The milky sap from the green fruit is applied to relieve itching, rashes, ringworm, and other skin problems, including sores caused by venereal disease. The sap, which is also considered the best medicine for improving the function of many parts of the body, such as bone, marrow, and muscle, is used to treat stomach and intestinal pains from ulcers and other conditions. *Seed*: Ingested in amounts proportionate to the patient’s age, used for deworming. *Root*: Preparations made from the roots are used to regulate menstruation.

###### Notes.

Medicinal uses of this species in India are discussed in Jain and DeFilipps (1991). Chemical constituents, pharmacological action, and medicinal use of this species in Indian Ayurveda are discussed in detail by Kapoor (1990). Medicinal uses of this species in China are discussed by Duke and Ayensu (1985).

The medicinal uses of this plant in the Caribbean region, as well as its chemistry, biological activity, toxicity and dosages, are discussed by Germosén-Robineau (1997). The chemical constituents, pharmacological activities, and traditional medicinal uses of this plant on a worldwide basis are discussed in detail by Ross (1999). A pharmacog- nostical profile including medicinal uses of this plant in Africa is given in Iwu (1993). Data on the propagation, seed treatment and agricultural management of this species are given by Katende et al. (1995). Details of the active chemical compounds, effects, herbal usage and pharmacological literature of this plant are given in Fleming (2000). Worldwide medicinal usage, chemical composition and toxicity of this species are discussed by Duke (1986).

The latex of *Carica papaya* contains chymopapain, an enzyme which does not produce fever (non pyrogenic), and which dissolves protein (proteolytic). In modern medicine, the drug “chymodiactin”, obtained from the chymopapain-containing latex of the plant, is administered as an injection into the center of a protruding disk in the spine, in order to relieve the symptoms of pressure from “herniated lumbar intervertebral disks”, i.e., to relieve the symptoms of pressure on nerve ends in the lower back. The latex of *Carica papaya* also contains another proteolytic enzyme, papain. It is used as a prominent ingredient in “panafil” ointment, a pharmaceutical preparation which helps to debride a wound (to digest dead and infected tissue, while leaving healthy tissue unaffected) and maintain a clean wound base, and to promote healing. In the preparation, the papain is combined with urea, which activates its digestive function (Bertran 1997).

The leaves contain an alkaloid, carpaine, which in small doses slows down the heart and reduces blood pressure, whereas in higher doses produces vasoconstriction; and that carpaine has spasmolytic action on smooth muscle, as well as being a strong amoebicide (Mors et al. 2000). Seeds and leaves of *Carica papaya* also contain glucotropaeolin, a bound toxin (Lan et al. 1998). Uses of this plant in the Upper Amazon region, including the eating of its grated unripe fruit with aspirin to induce an abortion, are given by Castner et al. (1998).

###### References.

Nordal (1963), Agricultural Corporation (1980), Perry (1980).

### Caryophyllaceae (Pink family)

#### 1. *Vaccaria* Wolf

##### Vaccaria hispanica

Taxon classificationPlantaeORDOFAMILIA

(Mill.) Rauschert (= Saponaria vaccaria L.)

###### Names.

**English**: cowcockle, cowherb, cow soapwort.

###### Range.

Asia and Europe.

###### Use.

*Leaf*: Used to treat skin diseases.

###### Notes.

In China the fruits and seeds are considered to be vulnerary, discutient, styptic; anodyne to treat cuts, to draw thorns from wounds, to apply to boils and scabies; and, used internally, a galactagogue. The shoot, leaves, flowers, and root have the same properties as the seeds (Perry 1980).

Reported constituents as of the seeds include saponin and a carbohydrate, lactosin (Perry 1980).

###### Reference.

Nordal (1963).

### Casuarinaceae (Casuarina family)

#### 1. *Casuarina* L.

##### Casuarina equisetifolia

Taxon classificationPlantaeORDOFAMILIA

L.

###### Names.

**Myanmar**: *kabwi*, *pinle-kabwe*, *pinle-tinyu*. **English**: Australian pine, beefwood, casuarina, common ironwood.

###### Range.

Tropical Asia to Australia and Oceania. Cultivated in Myanmar.

###### Uses.

*Bark*: Used to treat chronic diarrhea and dysentery.

###### Notes.

The medicinal uses of this species in India are discussed in Jain and DeFilipps (1991). Medicinal uses of this species in China are discussed in Duke and Ayensu (1985).

###### Reference.

Nordal (1963).

### Celastraceae (Staff-tree family)

#### 1. *Celastrus* L.

##### Celastrus paniculatus

Taxon classificationPlantaeORDOFAMILIA

Willd.

###### Names.

**Myanmar**: *hpak-ko-suk*, *myin-gaung-nayaung*, *myin-gondaing*, *myin-lauk-yaung*, *new-ni*. **English**: black oil plant.

###### Range.

India to southern China south (not in Borneo) to Australia and New Caledonia. In Myanmar, found in Chin, Kachin, Mandalay, and Yangon.

###### Uses.

*Leaf*: Used as an opium antidote. *Seed*: Used as a stimulant.

###### Notes.

In India the bark is used for wounds, cough, colds, and fever; the leaf and root for headache; and the seed for piles and digestive trouble (oil), rheumatic pain, and as a stimulant (Jain and DeFilipps 1991). In Indo-China the oil from the seeds is used to treat beri-beri; in Indonesia the leaves are used in treating dysentery; and in the Philippines the pulverized seeds are employed as a nerve stimulant, and to treat rheumatism and paralysis (Perry 1980).

Reported chemical constituents include phytosterol, celastrol, a resinous substance in the aril of the seed, and a semi-solid fat. Two alkaloids, celastrine and paniculatin, have been isolated from the oil cake, but were not found in the oil expressed from the seeds (Perry 1980).

###### References.

Perry (1980), Forest Department (1999).

#### 2. *Euonymus* L.

##### Euonymus kachinensis

Taxon classificationPlantaeORDOFAMILIA

Prain

###### Names.

**Myanmar**: *mashawt pin*. **English**: winterberry.

###### Range.

Temperate Asia. Grows naturally in Myanmar; most abundant in Kachin state.

###### Uses.

*Leaf*: Used as stimulant. Eaten after consumption of questionable foods to neutralize toxins instantly. They are also eaten immediately after bee stings or bites from venomous snakes and scorpions to prevent the venom from reaching the heart. Pulp from the chewed leaves is applied as a poultice to bites and stings. To promote healing of broken bones, the leaves are eaten rather than applied topically because topical application in the case of broken bones is thought to cause “retraction of bad blood”, pain, and infection. However, for bleeding injuries, a poultice of the masticated leaves is applied in a circle around or directly over the wound to stimulate healing. Note: Eating the leaves in the absence of need is thought to lead to lethargy and heaviness of the body.

###### References.

Nordal (1963), Agricultural Corporation (1980).

### Chloranthaceae (Chloranthus family)

#### 1. *Chloranthus* Sw.

##### Chloranthus elatior

Taxon classificationPlantaeORDOFAMILIA

Link (= Chloranthus officinalis Blume)

###### Names.

**Myanmar**: *thanat-kha*, *yuzara*. **English**: chloranthus.

###### Range.

Southeastern Asia to as far south as New Guinea. Cultivated in Myanmar.

###### Use.

*Leaf*: Used as stimulant.

###### Notes.

The species is an aromatic. On the Malay Peninsula the dried crushed leaves or roots are used to make a tea for use as a sudorific and a febrifuge; also, after boiling, the roots are powdered and rubbed on the body to treat fever. In Indonesia little packets (stem with root and leaves) are used as a valued remedy for fever and as a restorative in some phases of venereal diseases. The plant is a stimulant; additionally, mixed with the bark of *Cinnamomum*, it is used as an antispasmodic during parturition (mostly a decoction of the crushed roots is used, but an infusion of the leaves in also mentioned) (Perry 1980).

###### Reference.

Nordal (1963).

### Cleomaceae (Cleome family)

#### 1. *Cleome* L.

##### Cleome gynandra

Taxon classificationPlantaeORDOFAMILIA

L. (= Gynandropsis gynandra (L.) Briq.; Gynandropsis pentaphylla (L.) DC.)

###### Names.

**Myanmar**: *caravalla*, *gangala*, *hingala*, *taw-hingala*. **English**: spiderflower, spiderwisp.

###### Range.

300 m; Himalayas, India, Sri Lanka, east to China and Malaysia. Widespread in Myanmar.

###### Uses.

*Leaf*: Rubefacient and vesicant. *Seed*: Febrifuge.

###### Notes.

In India the whole plant is used for scorpion sting; the leaf for rheumatism, neuralgia, stiff neck, diseases of the ear, pyorrhea, skin diseases, also vermicidal; the seed is used for cough; and an unspecified plant part is used for asthma and fever (Jain and DeFilipps 1991).

###### Reference.

Nordal (1963).

### Clusiaceae (Garcinia family)

#### 1. *Garcinia* L.

##### Garcinia × mangostana

Taxon classificationPlantaeORDOFAMILIA

L.

###### Names.

**Myanmar**: *mingut*. **English**: mangosteen.

###### Range.

Malay region; cultivated in the tropics. Cultivated in Myanmar.

###### Uses.

*Bark*, *Fruit*: Either bark or pericarp (fruit rind) used to treat diarrhea and dysentery.

###### Notes.

Most parts of the tree are astringent, but the powdered rind of the dried fruit is the most efficacious. In India, Indo-China south including Indonesia and the Philippines, the bark and fruit (pericarp) are used in the same ways as they are in Myanmar. On the Malay Peninsula a decoction of the root is given for irregular menstruation, and a decoction of the leaves with unripe bananas and benzoin is applied externally to wounds such as those of circumcision. Additionally, in Indonesia the external application of the prepared peicarp is as in a clyster and a sitz bath, and is also used to treat atonic ulcers and swollen tonsils (Perry 1980).

###### Reference.

Perry (1980).

##### Garcinia xanthochymus

Taxon classificationPlantaeORDOFAMILIA

Hook.f.

###### Names.

**Myanmar**: *daungyan*, *dawyan-ban*, *hmandaw*, *madaw*. **English**: garcinia.

###### Range.

Western Himalayas, northern India. Widely distributed in Myanmar.

###### Uses.

*Fruit*: A preparation of the fruit is given to treat bilious conditions, diarrhea, and dysentery.

###### Notes.

An extract from the bark of this species was found to stimulate the growth of neurons or nerve tissues in culture studies (Chanmahasathien et al. 2003). Research has also been conducted on the anti-inflammatory activity of the leaves, which were found to contain high levels of xanthones, reported to possess antibacterial and antimalarial properties (Pal et al. 2005).

###### Reference.

Perry (1980).

### Colchicaceae (Colchicum family)

#### 1. *Gloriosa* L.

##### Gloriosa superba

Taxon classificationPlantaeORDOFAMILIA

L.

###### Names.

**Myanmar**: *hsee mee-tauk*. **English**: climbing lily, flame lily, superb lily.

###### Range.

Tropical Africa and Asia. Grows naturally all over Myanmar, but more common in the temperate regions.

###### Conservation status.

Least Concern [LC] (IUCN 2017).

###### Uses.

Bitter, astringent and sharp in taste with heating properties, this plant is used to control flatulence and phlegm, promote urine production, treat bladder conditions, poisoning, leprosy, hemorrhoids, bloating and lung problems. *Leaf*: Powdered leaves are applied to wounds and sores to kill germs and promote healing. They are also ingested with jaggery to expel roundworms and threadworms. Mixed with lime juice, the leaf powder is used as a swab for the inside of the ear or as drops for earaches and ear infections. *Root*: The tuber serves as an abortifacient, and is used to treat ulcers, leprosy, and piles. Washed thoroughly, the tubers are crushed together with water, and the resulting mixture is applied to the navel and over the uterus area to induce fast and easy labor in childbirth. Tuber paste is also applied to relieve bruises and inflammation. The liquid from powdered tubers soaked in water is ingested to cure gonorrhea. (Note: Because the tubers contain a powerful *poison*, they should be used only under the direction of experienced and able physicians).

###### Note.

The medicinal uses of this species in India are discussed in Jain and DeFilipps (1991).

###### References.

Nordal (1963), Agricultural Corporation (1980), Forest Department (1999).

### Combretaceae (West Indian Almond family)

#### 1. *Combretum* Loefl.

##### Combretum indicum

Taxon classificationPlantaeORDOFAMILIA

(L.) DeFilipps (= Quisqualis indica L.)

###### Names.

**Myanmar**: *dawe-hmaing-nwe*, *tanah-pacow-kawaing angine* (Mon), *mawk nang-nang*, *nang-mu* (Shan). **English**: Chinese honeysuckle, Rangoon creeper.

###### Range.

Southeast Asia to the Philippines and Papua New Guinea. Grows naturally in the hot and humid areas of Myanmar.

###### Uses.

*Leaf*: Effective against dysentery. Utilized in the treatment of diabetes; lightly boiled in water, eaten in a salad to quickly alleviate dysentery with mucus or blood. Liquid from boiling leaves is taken to relieve indigestion and shooting pains. *Seed*: Two or three are crushed and taken with honey for deworming. They are also eaten as a remedy for severe illness accompanied by diarrhea.

###### Notes.

In China the fruit is primarily used as a vermifuge; also for abdominal distention, dyspepsia, and marasmus, leucorrhea; macerated in oil, it is applied to skin ailments due to parasites; the ripe seed is roasted and used to treat diarrhea and fever (Duke and Ayensu 1985). In India the seed is used as an anthelmintic (Jain and DeFilipps 1991). Dagar and Singh (1999) describe indigenous medicinal uses of this species in the Andaman and Nicobar Islands (India).

Extracts show antitumor and cathartic activity (Duke and Ayensu 1985).

###### References.

Nordal (1963), Agricultural Corporation (1980).

#### 2. *Terminalia* L.

##### Terminalia bellirica

Taxon classificationPlantaeORDOFAMILIA

(Gaertn.) Roxb.

###### Names.

**Myanmar**: *hroirwk*, *mai-hen*, *mai-mahen*, *mai-naw*, *makalaw*, *tawitho*, *thiag-riang*, *thit-seint*. **English**: belleric, myrobalan.

###### Range.

India to Indo-China south through Indonesia. In Myanmar found in Bago, Magway, and Mandalay.

###### Uses.

The flowers, bark, fruit, and seed kernel are used in medications to relieve constipation, treat heart disease, cure eye infections, strengthen hair, protect the voice from deterioration, and clear blood irregularities, as well as to relieve sore throat and coughing. However, ingesting too much is known to cause vomiting and dizziness. *Flower*: Liquid from boiling the flowers is taken for spleen enlargement, excessive bowel movements, and chest pains. *Bark*: Made into a paste, it is applied topically as a remedy for vitiligo and taken orally for anemia. Liquid from boiling the bark is held in the mouth to relieve toothaches and gum inflammation. *Fruit*: Dried and used to treat cough and eye diseases. Applied topically to circles under the eyes, the fruit paste is used to relieve aching. A mixture of honey and the paste made from the fruit skin is licked to cure asthma and coughs. Powdered fruit mixed with cane sugar is taken daily for impotence. The fruit itself is eaten as a tonic to give strength and as a remedy for hemorrhoids, edema, leprosy, diarrhea, shooting stomach pain, and headaches. *Seed*: A paste made from the seed kernel mixed with alcohol is taken to relieve pain from urination and from kidney stones. The warmed kernel paste is applied topically to reduce swelling and to relieve aches and pains caused by injuries.

###### Notes.

In India the bark is used as a diuretic; also for high fever, cold dysuria, sunstroke, cholera (with the bark of two other species), snakebite (with the bark of one other species); the resin is used for cramps; the gum is a demulcent, purgative, and soothes itches. The fruit is used as an astringent, brain tonic, for measles (with plant parts from two other species), cough, asthma, stomach and liver disorders, piles, leprosy, dropsy, fever; also, half-ripe fruit is purgative, but ripe fruit has the opposite property. The oil is used on rheumatic pain; fruit pulp (with honey) is used on opthalmia; and the seeds are used for gastric problems (Jain and DeFilipps 1991). In Indo-China the species is used as an astringent and tonic, as a purgative when green, and as a narcotic (in large doses). In Indonesia the ripe fruit, with seed removed, is roasted and powdered, then used to protect the navel after the umbilical cord has fallen off, also part of a complicated medicine to treat women’s illnesses (Perry 1980).

The fresh fruit yields glucose, tannin, and three glycosidal fractions (Perry 1980).

###### References.

Agricultural Corporation (1980), Perry (1980).

##### Terminalia catappa

Taxon classificationPlantaeORDOFAMILIA

L.

###### Names.

**Myanmar**: *badan*, *banda*. **English**: Indian almond, Malabar almond, tropical almond, West Indian almond.

###### Range.

Tropical Asia to Northern Australia and Polynesia, and cultivated in many places. Cultivated in Myanmar.

###### Uses.

*Whole plant*: Astringent, also used in treating dysentery. Nordal lists this plant as having medicinal value, but does not give use(s).

###### Notes.

Medicinal uses of this species in India are discussed in Jain and DeFilipps (1991). Indigenous medicinal uses of this species in the Andaman and Nicobar Islands (India) are described by Dagar and Singh (1999). Medicinal uses of the species in East and Southeast Asia are discussed in Perry (1980). Some of these uses follow: In Indonesia the leaves are used as a dressing for swollen rheumatic joints; in the Philippines, the red leaves are used as a vermifuge, sap of the young leaves is cooked with oil from the kernel to treat leprosy, leaves mixed with oil is rubbed on the breast to relieve pain, or heated and applied to rheumatic an numb parts of the body; in the Solomon Islands leaves are used to treat yaws, bark and root bark are used for bilious fevers, diarrhea, dysentery, and as remedy for sores and abscesses; in Indonesia, the plant it is used as a mild laxative and a galactagogue for women.

Unripe fruits of *T. catappa* contain tannin and terminalin, which are toxic to cattle and sheep when eaten, causing kidney necrosis (Lan et al. 1998). The bark is rich in tannin; oil from the kernel contains olein, palmitin, and stearin; from fruit grown in Puerto Rico, myristic and linoleic acids were extrated; also, the leaves show some antibiotic activity against *Staphylococcus* (Perry 1980).

###### References.

Nordal (1963), Perry (1980).

##### Terminalia chebula

Taxon classificationPlantaeORDOFAMILIA

Retz.

###### Names.

**Myanmar**: *hpan-khar-thee*, *mai-mak-na*, *mai-man-nah*, *mana*, *panga*, *phan-kha*, *thankaungh*. **English**: myrobalan.

###### Range.

Native to India, Indo-China, Myanmar, and Thailand. Cultivated and imported elsewhere. Reported from Myanmar.

###### Uses.

*Fruit*: Used as astringent, antidysenteric, laxative, and tonic. After soaking crushed fruit in water overnight, the clear liquid is used as an eye drop to cure aching eyes. Drinking the fruit powder dissolved in milk daily promotes longevity. *Seed*: Made into a paste to treat pimples. *Leaf*: Used to cure eye problems and to make laxatives, carminatives, and thway-hsay (literally means “blood medicine”), the traditional blood purification mixture. Used to treat various male and female related disorders, and to treat hemorrhoids. *Bark*: Boiled and the liquid taken to treat diarrhea and dysentery. Crushed and used as a poultice to prevent excessive bleeding.

###### Notes.

Medicinal uses of this species in India are discussed in Jain and DeFilipps (1991). Medicinal uses of this species in China are discussed in Duke and Ayensu (1985).

Perry (1980) discusses uses of the species in East and Southeast Asia. In China, it is used as a laxative and tonic, deobstruent, carminative, astringent, expectorant, and as a remedy for salivating and heartburn; in Indo-China, the fruit is used as a purgative; on the Malay Peninsula, in addition to the uses listed above, the fruits (imported from India) are considered to be antidiarrheic, styptic, antibilious, and antidysenteric; and in Indonesia the unripe and half-ripe fruit (also imported) and galls from this plant are used as an astringent; the flowers are used in a large number of remedies for dysentery.

Reported constituents include oil, tannin, and chebulic and ellagic acids (Perry 1980).

###### References.

Nordal (1963), Agricultural Corporation (1980), Perry (1980), Forest Department (1999).

##### Terminalia citrina

Taxon classificationPlantaeORDOFAMILIA

(Gaertn.) Roxb.

###### Names.

**Myanmar**: *kya-su*, *hpan-kha-ngai*. **English**: black chuglam, citrine myrobalan.

###### Range.

From India to the Philippines. Found growing naturally all over Myanmar, especially in Taninthayi.

###### Uses.

*Fruit*: Of its five tastes - sour, astringent, bitter, savory, and hot - astringency is the strongest. Eaten raw, it stimulates bowel movements and can cause diarrhea; eaten boiled, it can cause constipation. The juice is consumed to promote longevity; it is also used for treating sore eyes and is considered good for the voice. A mixture of powder made from the fruit and honey is licked to cure gas. Pounded it is smoked in a pipe as a remedy for asthma; consumed in a blanc mange-like confection, it alleviates intermittent diarrhea and diarrhea caused by indigestion. For burns, a mixture of ground fruit, water, honey and sesame seed oil is applied topically. The powder can be used as a toothpaste to whiten teeth and cure tooth diseases. Liquid from boiling the fruit with *sha-zay* (resin from *Acacia catechu*) is used as a mouthwash to strengthen the teeth; liquid from boiling it in water until the water is reduced to one-fifth the starting volume is given with honey to for various disorders of the mouth and palate; and liquid from fruit boiled with water and reduced to one-fifth the starting volume is used to wash flesh-eroding sores. Crushed fruit is applied to the head for migraine headaches. Liquid from soaking it in water overnight is used the following day as a rinse to cool the eyes and strengthen vision. Fruit powder is rolled with juice from *mu-yar gyi* (*Adhatoda vasica* = *Justicia adhatoda*) leaves to form seven pellets, which are dried in the sun; the pellets are then rolled in honey and licked to stop vomiting and bleeding. The powder licked with honey, or rolled together with jaggery into pellets, is taken as a remedy for acid stomach. Boiled in cow urine, fruit is given as a cure for anemia and other debilitating diseases.

###### Note.

In Indonesia a decoction made from this species and “adaspoelasari” is taken as a treatment for abdominal illness; in the Philippines, the fruit is considered an astringent, and a decoction is used in treating thrush and obstinate diarrhea (Perry 1980).

###### Reference.

Agricultural Corporation (1980).

##### Terminalia tomentosa

Taxon classificationPlantaeORDOFAMILIA

Wight & Arn.

###### Names.

**Myanmar**: *dap*, *mai-hok-hpa*, *merokwa*, *paung*, *taukkyan*, *tauk-kyant*. **English**: beddome.

###### Range.

India, Sri Lanka, and Myanmar. Widespread in Myanmar.

###### Uses.

*Bark*: Used to treat diarrhea; also as an astringent, diuretic, and cardiotonic.

###### Note.

Perry (1980) notes that this is one of the less medicinally useful species in the genus and lists the uses of six other members of the genus in East and Southeast Asian countries.

###### References.

Nordal (1963), Perry (1980).

### Commelinaceae (Dayflower family)

#### 1. *Commelina* L.

##### Commelina paludosa

Taxon classificationPlantaeORDOFAMILIA

Blume (= Commelina obliqua Buch.-Ham. ex D.Don)

###### Name.

**English**: dayflower.

###### Range.

Pakistan, India, Sri Lanka, Bangladesh to Malaysia, Indonesia and the Philippines. Cultivated in Myanmar.

###### Uses.

*Root*: Used to treat vertigo, fevers, and bilious afflictions.

###### Reference.

Nordal (1963).

#### 2. *Tradescantia* L.

##### Tradescantia spathacea

Taxon classificationPlantaeORDOFAMILIA

Sw. (= Rhoeo discolor (L’Hér.) Hance)

###### Names.

**Myanmar**: *mi-gwin-gamone*. **English**: boat lily, Moses in a cradle, oyster plant.

###### Range.

Southern Mexico, Belize, Guatemala and West Indies. Grows throughout Myanmar; cultivated.

###### Uses.

*Whole plant*: One teaspoon of the liquid obtained from pounding the plant mixed with a little sugar (taken three times a day) used to cure coughs and loosen mucus. *Stem* and *Leaf*: Liquid obtained from boiling crushed stems and leaves down to 1/3, together with a little raw sugar (1 tablespoon taken three times a day), used to treat vomiting of blood. *Leaf*: Used to remedy burns, scalds, and dysentery.

###### Notes.

In China the plant is used as a poultice on swellings and wounds; the flower is used to treat dysentery, enterorrhagia, and hemoptysis (Duke and Ayensu 1985).

###### References.

Agricultural Corporation (1980), Forest Department (1999).

### Convolvulaceae (Morning Glory family)

#### 1. *Convolvulus* L.

##### Convolvulus arvensis

Taxon classificationPlantaeORDOFAMILIA

L.

###### Names.

**Myanmar**: *kauk-yoe nwai*, *kauk-yo-nwe*, *tike-tot-grine* (Mon). **English**: deer’s foot, field bindweed, morning glory.

###### Range.

Mediterranean Europe native; temperate and dry subtropical climates. Found growing naturally around lakes, ponds, streams, and in cultivated fields. In Myanmar, found in Magway and Mandalay.

###### Uses.

*Whole plant*: Known for a bitter and sweet taste, as well as heating properties, all five parts (root, stem, leaf, flower and fruit) used in preparations to support urinary function, increase libido, alleviate chronic anemia and coughs, and treat a swollen penis. To relieve bone and joint aches, all five parts are mashed, wrapped in cloth, and placed on the painful areas. For mouth sores, liquid from boiling the five parts is held in the mouth; the liquid is also used as a wash for old sores. *Leaf*: Mashed and applied with a bandage to bumps, cysts, and other skin sores. The juice is used for rashes and itching. *Root*: Used in laxative medicines.

###### Notes.

In Indonesia all parts of the plant are used as a purgative, and the roasted seeds are anthelmintic, diuretic, and antibilious; on the Malay Peninsula a poultice is applied to the head in cases of jungle fever; and in the Philippines a decoction of the roots is used as a mouthwash for toothache (Perry 1980). The medicinal uses of the species in India are discussed in Jain and DeFilipps (1991).

###### Reference.

Agricultural Corporation (1980).

#### 2. *Cuscuta* L.

##### Cuscuta reflexa

Taxon classificationPlantaeORDOFAMILIA

Roxb.

###### Names.

**Myanmar**: *shwe-new*, *shwe-nwe-pin* (Hsay). **English**: dodder, giant dodder.

###### Range.

Afganistan, throughout northern India to Yunnan China, Java, and Sri Lanka. Found growing naturally in upper Myanmar, Pyin Oo Lwin, and in the upper Chindwin area.

###### Uses.

Sweet-tasting; used to treat diseases of the bile as well as to increases strength and the sperm count; also considered to promote longevity. *Whole plant*: The liquid from boiling it is either drunk or rubbed onto the abdomen to treat inflammation and hardening of the liver. Equal parts of the powdered plant mixed with dried ginger powder are mixed with butter and applied to longstanding sores to heal them. After crushing the plant and making a paste with water, it is applied to cure itches and rashes. The plant is also used to treat irregularities of the blood. Used as a shampoo, it cools the scalp, clears the brain, and cures dandruff and head lice.

###### Note.

In India the whole plant is used to reduce swellings and for headaches; the stem is used for jaundice and wounds (Jain and DeFilipps 1991).

###### Reference.

Agricultural Corporation (1980).

#### 3. *Evolvulus* L.

##### Evolvulus alsinoides

Taxon classificationPlantaeORDOFAMILIA

(L.) L.

###### Names.

**Myanmar**: *kyauk-hkwe-pin*. **English**: slender dwarf morning-glory, speedwell.

###### Range.

Florida, tropical America. In Myanmar, found in Mandalay and Yangon.

###### Uses.

*Leaf*, *Root*: Used as a tonic, anthelmintic, and antiasthmatic.

###### Notes.

In India the whole plant is used as a febrifuge and vermifuge; the leaf is used to treat asthma and bronchitis (Jain and DeFilipps 1991). In the Philippines an infusion of the species is used to treat certain bowl irregularities; it is also used as a vermifuge and febrifuge (Perry 1980).

###### Reference.

Nordal (1963).

#### 4. *Ipomoea* L.

##### Ipomoea alba

Taxon classificationPlantaeORDOFAMILIA

L. (= Ipomoea bona-nox L.)

###### Names.

**Myanmar**: *kyahin*, *kyan-hin pin*, *hla-kanin kyam* (Mon), *nwe-kazun-phyu*. **English**: Indian jalap, moon flower, tropical white morning-glory, turpeth root.

###### Range.

Central and southern China; Bangladesh, Cambodia, India, Indonesia, Laos, Malaysia, Myanmar, Nepal, New Guinea, Pakistan, the Philippines, Sri Lanka, Thailand, Vietnam; Africa; Australia; Carribbean Territories; North America; South America; amd Pacific Islands. Found growing naturally all over Myanmar; also cultivated.

###### Uses.

Sweet, bitter, and astringent, with heating properties; used to expel and cure flatulence disorders, as well as to treat leprosy. *Whole plant*: Shoots are made into a soup with chicken bones or *din-gyi* (*Oroxylum indicum*) for urinary problems. The juice is consumed with milk and sugar for kidney stones. It is also used to make medicines to treat eye diseases, flatulence, and chest pain. *Root*: Bark from the root is crushed, mixed with milk, and taken as a laxative. A mixture of roots, ginger, and black pepper is given for leprosy, edema, and male diseases.

###### Notes.

The medicinal use of this species in India is discussed in Jain and DeFilipps (1991). In Indo-China an infusion of the roots and seeds is used as a purgative (Perry 1980).

###### Reference.

Agricultural Corporation (1980).

##### Ipomoea aquatica

Taxon classificationPlantaeORDOFAMILIA

Forssk.

###### Names.

**Myanmar**: *kazun-galay*, *kazun yoe-n*, *kazun-ywet*, *ye-kazun*. **English**: Chinese waterspinach, rabbit greens, swamp morning-glory, waterspinach.

###### Range.

Native to central and south China. Widespread in Myanmar, where it is found growing in freshwater ditches, streams, ponds, and paddy field; and is also grown as a cultivated plant.

###### Conservation status.

Least Concern [LC] (IUCN 2017).

###### Uses.

*Leaf*: Sweet with cooling properties, stimulates lactation, protects against germs found in water, works as an expectorant, and neutralizes poisons. Leaves are used to treat burning, thirst, and fevers associated with urinary diseases, as well as to treat wounds caused by burns. For dysentery, they are cooked and eaten. Crushed together with equal amounts of gourd (*Lagenaria siceraria*) leaves, tamarind (*Tamarindus indica*) leaves, and fine rice powder, they are used to make a poultice placed above the pubic region to induce urination in cases of difficulty urinating when the bladder is full; the same poultice is used to stop excessive menstrual bleeding. Together with gourd leaves, they are soaked in water and applied to chronic sores. Liquid from the boiled leaves is taken for diarrhea and indigestion; boiled together with ripe tamarind (*Tamarindus indica*) fruit and salt, they are given as a cure for kidney stones, as well as for all other urinary diseases.

###### Notes.

The medicinal uses of this species in India are discussed in Jain and DeFilipps (1991). Medicinal uses of this species in China are discussed in Duke and Ayensu (1985). Perry (1980) covers the medicinal uses of the species in China and Indonesia.

The leaves are considered a good source of minerals and vitamins, especially carotene. Hentriacontane, sitosterol, and sitosterol glycoside have been separated from the lipoids (Perry 1980).

###### Reference.

Agricultural Corporation (1980).

##### Ipomoea hederifolia

Taxon classificationPlantaeORDOFAMILIA

L. (= Ipomoea coccinea L.)

###### Names.

**Myanmar**: *mat-lay*. **English**: red morning-glory, star ipomoea.

###### Range.

Native range the Americas. In Myanmar, found in Yangon.

###### Use.

*Root*: Sternutative.

###### Notes.

In India the root is a sternutatory (Jain and DeFilipps 1991). In the Philippines an infusion of the species is used to treat certain bowl irregularities; it is also used as a vermifuge and febrifuge (Perry 1980).

###### Reference.

Nordal (1963).

##### Ipomoea pes-caprae

Taxon classificationPlantaeORDOFAMILIA

(L.) R.Br.

###### Names.

**Myanmar**: *pinle-kazun*. **English**: beach morning glory, goat’s foot creeper.

###### Range.

Pantropical; seashores. In Myanmar, found in Ayeyarwady, Bago, Rakhine, Taninthayi, and Yangon.

###### Uses.

*Leaf*: Serves as a laxative and emetic. Decocted leaves are applied as a poultice to treat colic.

###### Notes.

The medicinal uses of this species in India are discussed in Jain and DeFilipps (1991). Indigenous medicinal uses of this species in the Andaman and Nicobar Islands (India) are described by Dagar and Singh (1999). Medicinal uses of the species in Indo-China, the Malay Peninsula, Indonesia, the northwestern Solomon Islands, Palau, New Guinea, and the Philippines are covered in Perry (1980).

No alkaloids were found, but there was 1.2% resin content. Magnesium, potassium, iron, and calcium were found in the ash. A volatile oil was also found (0.048%) (Perry 1980).

###### References.

Nordal (1963), Perry (1980).

### Coriariaceae (Coriaria family)

#### 1. *Coriaria* Niss. ex L.

##### Coriaria nepalensis

Taxon classificationPlantaeORDOFAMILIA

Wall.

###### Name.

**English**: mussoorie berry.

###### Range.

China, Bhutan, India, Kashmir, Myanmar, Nepal, Pakistan. In Myannmar, found in Kachin and Shan.

###### Uses.

*Leaf*: Laxative (*poisonous*).

###### Notes.

Species belonging to the genus *Coriaria* have little or no medicinal value in East and Southeast Asia, but both the leaves and fruit are *poisonous*; and, since the fruits are attractive, children are poisoned by eating them (Perry 1980).

Reported chemical constituents of the seeds include tutin, pseudotutin, and coriamyrtin. Coriamyrtin is considered to be “a violent convulsive poison” (Perry 1980).

###### Reference.

Nordal (1963).

### Costaceae (The Costus family)

#### 1. *Cheilocostus* C.D. Specht

##### Cheilocostus speciosus

Taxon classificationPlantaeORDOFAMILIA

(J.Koenig) C.D. Specht (= Costus speciosus (J.Koenig) Sm.)

###### Names.

**Myanmar**: *palan-taunghmwe*. **English**: Indian spiral ginger, crepe ginger.

###### Range.

Southeast Asia. In Myanmar, found in Bago, Kachin, Mandalay, Sagaing, Shan, Taninthayi, Yangon.

###### Use.

*Stem*: Rhizome used as laxative.

###### References.

Nordal (1963), Forest Department (1999).

### Crassulaceae (Air Plant family)

#### 1. *Bryophyllum* Salisb.

##### Bryophyllum pinnatum

Taxon classificationPlantaeORDOFAMILIA

(Lam.) Oken (= Bryophyllum calycinum Salisb.; Kalanchoe pinnata (Lam.) Pers.)

###### Names.

**Myanmar**: *ywet-kya-pin-bauk*. **English**: air plant, floppers, leaf of life, life plant.

###### Range.

Old World tropics; exact origin unknown. Widely distributed in Myanmar.

###### Use.

*Leaf*: Used to treat alopecia. Apply leaf juice to areas affected by impetigo, erysipelas and boils to treat sores. Roasted and stuck on the wound to stop the flow of blood and to promote healing. Roasted and stuck onto contusions to alleviate and heal inflammation. Crushing one or two leaves together with a bit of pepper and taking the mixture orally will treat retention of urine and other symptoms caused by hemorrhoids and venereal diseases. Crushing the leaf and taking the resulting juice will help treat cholera. Applying the juice of the leaf will heal dislocations, knotted muscles, and burns. Crushed and placed over eyes to treat eye ailments. Juice from the leaf together with rock sugar to treat blood in the urine and dysentery. Juice from the leaf can be ground together with salt and pressed into a scorpion bite to neutralize the poison.

###### Notes.

Crushed leaves are cooling and used as a disinfectant by indigenous cultures. From southern China to Guam, they are used on suppurating boils, wounds, skin diseases, burns, scalds, corns, and also (with friction) for rheumatism, neuralgia, and pain. Leaves are placed on the forehead for headaches, and on the chest for cough and pain. They are mixed with leaves from other species for a poultice applied to the abdomen for bowl troubles. Similar uses are recorded from the Philippines. Juice from heated leaves and stems is squeezed on body areas infected with scabies (Perry 1980). In India the leaf is used for acidity and other gastric trouble; also on wounds and insect bites (Jain and DeFilipps 1991). The medicinal uses of this species in the Andaman and Nicobar Islands (India) are described by Dagar and Singh (1999).

The medicinal uses of this plant in the Caribbean region, as well as its chemistry, biological activity, toxicity and dosages, are discussed by Germosén-Robineau (1997). Mors et al. (2000) discuss the immunosuppressive effect of extracts of this species in the form of an inhibitory action on human lymphocyte proliferation. The “active constituent is bryophylline, a substance used to treat intestinal troubles caused by bacteria” (Perry 1980).

###### References.

Agricultural Corporation (1980), Forest Department (1999).

### Cucurbitaceae (Melon family)

#### 1. *Benincasa* Savi

##### Benincasa hispida

Taxon classificationPlantaeORDOFAMILIA

(Thunb.) Cogn. (= Benincasa cerifera Savi)

###### Names.

**Myanmar**: *kyauk-pha-yon*, *lun-tha*, *pora-mat*. **English**: ash pumpkin, wax gourd, white gourd.

###### Range.

Tropical Asia. Cultivated all over Myanmar up to altitudes of 1220 m.

###### Uses.

Known for a sweet and slightly salty taste, giving strength and controlling bile, the flowers, seeds, roots and especially the fruits are used in medicinal preparations. *Flower*: Crushed and ingested as a cure for cholera. *Fruit*: Has restorative properties important in the treatment of weaknesses from lung disease. The ripe fruit promotes bowel movements, cleanses the bladder, and alleviates diseases of the blood. The juice is used to stop bleeding, vomiting of blood, and otherwise excreting blood, and it is given for epilepsy, strokes, and in the treatment of insanity. It is also given, together with a small amount of *shein-kho* (*Gardenia resinifera*) and wheat ash (obtained from burning grains in closed receptacles so more of the structure is retained), to alleviate bladder inflammation and dissolve kidney stones. *Seed*: Used for deworming. *Root*: A mixture of root powder and hot water is taken for coughing, bronchitis, and asthma.

###### Notes.

The medicinal uses of this species in India are discussed in Jain and DeFilipps (1991). Medicinal uses of this species in China are discussed in Duke and Ayensu (1985). The medicinal uses of the species in China, Indonesia, and the Philippines are discussed in Perry (1980).

Reported constituents include fixed oil, starch, the alkaloid cucurbitine, an acid resin, proteins (myosin and vitellin), and sugar (Perry 1980).

###### Reference.

Agricultural Corporation (1980).

#### 2. *Coccinia* Wight & Arn.

##### Coccinia grandis

Taxon classificationPlantaeORDOFAMILIA

(L.) Voigt (= Cephalandra indica (Wight & Arn.) Naudin; Coccinia indica Wight & Arn.)

###### Names.

**Myanmar**: *kinmon*, *kin pone*, *hla cawi bactine* (Mon), *taw-kinmon*. **English**: ivy gourd, wild snake gourd.

###### Range.

Africa, temperate and tropical Asia, Australasia, Pacific. Found growing wild throughout Myanmar; found growing up trees and hedges.

###### Uses.

Of the two kinds of kin pone, bitter and sweet, the bitter kind is the most used in medicines. All five parts (root, stem, leaf, flower and fruit) are employed. *Whole plant*: The liquid from the whole boiled plant is well-known as an effective expectorant. *Fruit*: The bitter fruit, known for cooling and laxative properties, is considered good for phlegm and bile. *Leaf*: The astringent and bitter leaves stimulate nerves and promote growth. The green leaves are stir-fried and eaten by diabetics. Leaves boiled with equal parts of coriander seeds are used in deworming preparations and as a laxative. They are also used in medicines to treat bile problems and lung ailments. The juice is applied frequently on cold sores to cure them. *Fruit*: Used to promote lactation in new mothers, to alleviate gas and blood diseases, and to treat asthma and bronchitis. *Root*: Can be used to reduce fever and to treat diarrhea.

###### Notes.

The medicinal uses of this species in India are discussed in Jain and DeFilipps (1991). Perry (1980) discusses the medicinal uses of this species in Indo-China and Indonesia.

###### References.

Nordal (1963), Agricultural Corporation (1980).

#### 3. *Cucumis* L.

##### Cucumis sativus

Taxon classificationPlantaeORDOFAMILIA

L.

###### Names.

**Myanmar**: *tha-khwar-thi*. **English**: cucumber.

###### Range.

Southern Asia. Cultivated in Myanmar.

###### Uses.

*Fruit*: Used as an anthelmintic. *Seed*: Used as diuretic.

###### Notes.

In India the fruit is used as a demulcent and the seed as a diuretic, tonic, and coolant (Jain and DeFilipps 1991). In Korea, the stalk of the unripe fruit is used as a remedy for dropsy, nasal disorders, epilepsy, and cough, also as an emetic; the fruit is used for cooling and as a diuretic; a cucumber soup is used to relieve retention of urine; a salve is used for skin disorders, scalds, and burns; a decoction of the dried roots is used as a diuretic and to treat beri-beri; juice from the crushed leaves is used as an emetic in acute indigestion of children. In Indo-China young fruit cooked in sugar is prescribed for children with dysentery. In Indonesia fruit and juice are considered beneficial for sprue and to treat gallstones; fruit and seeds are cooling, used both externally and internally (Perry 1980).

Reported constituents include a small amount of saponin, a proteolytic enzyme, and glutathione (Perry 1980),

###### Reference.

Nordal (1963).

#### 4. *Luffa* Mill.

##### Luffa cylindrica

Taxon classificationPlantaeORDOFAMILIA

(L.) M.Roem. (= Luffa aegyptiaca Mill.)

###### Names.

**Myanmar**: *kawe-thi*, *tawbut*. **English**: luffa, sponge gourd, smooth loofah, vegetable sponge.

###### Range.

Old World tropics. Cultivated in Myanmar.

###### Uses.

*Fruit*: Employed as a laxative and also used in the treatment of leprosy.

###### Notes.

In India the seed is used as a cathartic and emetic (Jain and DeFilipps 1991). Perry (1980) discusses the species’ medicinal uses in China, Indo-China, the Malay Peninsula, and in general.

Reported constituents include a bitter principle, saponin, mucilage, xylan, mannan, galactan, lignin, fat, and protein (Perry 1980). Chemical constituents, pharmacological action, and medicinal use of this species in Indian Ayurveda are discussed in detail by Kapoor (1990). The chemistry, pharmacology, toxicology, and use of this species as a hunting poison and medicinal plant in Africa are discussed by Neuwinger (1994). Details of the active chemical compounds, effects, herbal usage, and pharmacological literature are given in Fleming (2000).

###### Reference.

Nordal (1963).

#### 5. *Momordica* L.

##### Momordica charantia

Taxon classificationPlantaeORDOFAMILIA

L.

###### Names.

**Myanmar**: *kyet-hinga*, *kyet-hin-kha*, *gaiyin* (Kachin), *sot-cawee-katun* (Mon). **English**: balsam-apple, balsam-pear, bitter cucumber, bitter gourd, bitter melon, wild balsam apple.

###### Range.

Tropical Asia. Cultivated throughout Myanmar; a small variety grows naturally.

###### Uses.

Bitter, rather hot and sharp, with cooling properties, and easily digested, this plant is considered good for bowel movements. It is used to defeat germs, control bile and phlegm, and stimulate hunger, as well as to alleviate anemia and eye, venereal, and urine-related diseases. *Whole plant*: Both the fruit and the whole plant are used in the treatment of diabetes. In folk medicines, the root, seeds, and fruits are used as a cathartic, abortive, aphrodesiac, analgesic, antipyretic, antirheumatic, emetic, digestant, anti-ulcerogenic, and anti-malarial. *Leaf*: Has the property of controlling fevers. Juice from crushed leaves is ingested as a remedy for stomach germs. A mixture of the juice and ground *hpan-kar* (*Terminalia chebula*) fruit is taken for jaundice and hepatitis. The juice is used as an emetic and purgative, given for bile problems, and also used as a cure for dengue hemorrhagic fever. Additionally, it is ingested as an antidote to rabid dog bites, and is also applied as a poultice on the bite and as a rinse for the area around the bite. A mixture of the leaves with salt and jaggery, boiled in water to one-third the starting volume, is taken for ague, chills, and fever. Crushed leaves are inhaled to cure giddiness. Also used as a laxative and an anthelmintic; to induce abortion (the fruits can cause severe vomiting and *may be lethal*) . *Leaf* and *Fruit*: Used in deworming preparations, as well as in medicines for piles, leprosy, and jaundice. *Fruit*: Used as a laxative, anthelmintic, and for diabetes. Dried and stone-ground to make a paste applied to the throat to treat goiter. A mixture of the juice and oil is taken for cholera, whereas a mixture of the juice with honey is used to alleviate edema. The juice from young fruits is warmed and applied to the joints to soothe inflammation. *Root*: Used as an astringent and also in preparations for hemorrhoids.

###### Notes.

Medicinal uses of this species in India are discussed in Jain and DeFilipps (1991). Indigenous medicinal uses of this species in the Andaman and Nicobar Islands (India) are described by Dagar and Singh (1999). Medicinal uses of this species in China are discussed by Duke and Ayensu (1985).

The medicinal uses of this plant in the Caribbean region, as well as its chemistry, biological activity, toxicity and dosages, are discussed by Germosén-Robineau (1997). The chemistry, pharmacology, history and medicinal uses of this species in Latin America are discussed in detail by Gupta (1995).

The chemical constituents, pharmacological activities, and traditional medicinal uses of this plant on a worldwide basis are discussed in detail by Ross (1999). The chemistry, pharmacology, toxicology, and use of this species as a hunting poison and medicinal plant in Africa are discussed by Neuwinger (1994). The toxic properties, symptoms, treatment and beneficial uses of this plant, *parts of which are poisonous*, are discussed by Nellis (1997). Worldwide medicinal usage, chemical composition and toxicity of this species are discussed by Duke (1986).

This plant is a well known traditional anti-diabetic remedy, its hypoglycemic properties based on peptides and terpenoids in the fruit juice (Marles and Farnsworth 1995). A polypeptide of molecular weight 11,000 is the basis of the blood sugar lowering properties of the fruit (Mors et al. 2000). Toxicity of this species is discussed by Bruneton (1999).

###### References.

Nordal (1963), Mya Bwin and Sein Gwan (1967), Agricultural Corporation (1980).

##### Momordica cochinchinensis

Taxon classificationPlantaeORDOFAMILIA

(Lour.) Spreng.

###### Names.

**Myanmar**: *hpak-se-saw*, *samon-nwe*, *taw-thabut*, *tha-myet*. **English**: Chinese bitter-cucumber, Chinese-cucumber, spiny bitter-cucumber, spiny bittergourd.

###### Range.

Temperate and tropical Asia, from China to the Moluccas; Australia. In Myanmar, found in Bago, Rakhine, and Yangon.

###### Uses.

*Fruit*: Used as a laxative. *Seed*: Used to treat chest problems and in parturition.

###### Notes.

The medicinal uses of this species in India are discussed in Jain and DeFilipps (1991). Medicinal uses of this species in China are discussed in Duke and Ayensu (1985). Perry (1980) discusses the medicinal uses of the species in East and Southeast Asian countries as follows: In China, where the seeds are used for abdominal illnesses, liver and spleen disorders, and hemorrhoids as well as bruises, swellings, skin trouble, ulcers, lumbago, chronic malaria, breast cancer, abscesses, and as a resolvent, and the root is used as an expectorant; Indo-China, where the seeds are ground and soaked in alcohol and water, then used as a resolvent of furuncles, abscesses, buboes, and mumps, and also in the treatment of edema of the legs and a kind of rheumatism; the Malay Peninsula, where “the Chinese living there use the plant in same way as in China”; Indonesia, where the juice the leaves is put in fresh palm wine, or the leaves are cooked in wine and used as remedy for weary, swollen legs; and in the Philippines, where the seeds are used as a pectoral, and the root as a substitute for soap and also to kill head lice.

Medicinal uses in the Guianas (Guyana, Surinam, French Guiana) are discussed in DeFilipps et al. (2004).

Reported chemical constituents include momordin, a-spinasterol, and sesquibenihiol. The seeds have a fixed oil comprised of stearic, palmitic, oleic, linoleic, and ricinoleic acids, and also trehalose, resinous, and pectic substances; and that the root contains momordine (Perry 1980).

###### References.

Nordal (1963), Perry (1980).

#### 6. *Trichosanthes* L.

##### Trichosanthes tricuspidata

Taxon classificationPlantaeORDOFAMILIA

Lour. (= T. palmata Roxb.)

###### Names.

**Myanmar**: *kyee-arh pin*. **English**: creeper.

###### Range.

Eastern Himalayas, India, east to China, Japan, Malaysia, tropical Australia. Found growing naturally all over Myanmar, except in cold areas.

###### Uses.

*Fruit*: Known for its bitter and slightly sweet taste, can be *harmful to the heart*. A mixture of crushed fruits boiled with coconut oil is used as an eardrop and nasal drop preparation. The juice stimulates bowel movements. Crushed dried fruits are mixed in smoking cheroots and pipes with tobacco to treat asthma. The fruit is also used for throat problems, indigestion, coughing, and leprosy, as well as chronic and gastric diseases. *Root*: Ground to form a paste rubbed onto the tongue to reduce phlegm. Tubers boiled and taken with honey for urinary disorders.

###### Notes.

In Indo-China the species is used as a strong purgative and emetic; on the Malay Peninsula the leaves are used to poultice boils; in Indonesia the leaves are one ingredient in a group of fresh plant parts from which the juice is extracted and used for medicines, the leaf juice is also drunk by children to treat diarrhea (Perry 1980). The medicinal uses of this species in India are discussed in Jain and DeFilipps (1991).

###### Reference.

Agricultural Corporation (1980).

### Cyperaceae (Sedge family)

#### 1. *Cyperus* L.

##### Cyperus scariosus

Taxon classificationPlantaeORDOFAMILIA

R.Br.

###### Names.

**Myanmar**: *nwar myay yinn*, *wet-myet-nyo*. **English**: Annie’s lace.

###### Range.

Damp and marshy places in temperate zone. Also reported from Myanmar.

###### Uses.

This astringent plant, sharp in taste with cooling properties, induces perspiration, urination (and constipation). *Root*: Tubers used for phlegm, bile, fever and bowel problems. Their use protects against loss of appetite, thirst, burning sensation, and asthma. Tuber paste given orally or applied externally provides a remedy for venomous snakebites. The paste is also used for nausea, gastric ailments, sour stomach, swollen limbs, itching, leprosy, herpes, and scabies. Combined with a bit of salt, the paste is used as an antidote for poisoning caused by ingesting the wrong medicines or foods. Tuber paste is brushed onto a thu-nge-sar banana (smaller and shorter variety of banana than “standard banana” found in the United States), which is roasted and given to children with high fevers. Boiled by itself, the tuber is taken as a cure for gonorrhea; boiled together with *oo-pat thagar* (*Butea monosperma*), it is a component of a syphilis remedy. Tuber powder is used to relieve the swelling caused by scorpion venom. Drinking the milk made by stewing tubers in milk and water until only milk is left provides a cure for dysenteric stomachaches with discharge of mucus or diarrhea with of bits of blood.

###### Notes.

The species is used in the treatment of abdomenal tumors (Duke 2009). Medicinal uses of this species in India are discussed in Jain and DeFilipps (1991).

Anti-inflammatory activity of the oil isolated from *C. scariosus* has been noted (Gupta et al. 1972).

###### Reference.

Agricultural Corporation (1980).

### Dilleniaceae (Dillenia family)

#### 1. *Dillenia* L.

##### Dillenia indica

Taxon classificationPlantaeORDOFAMILIA

L.

###### Names.

**Myanmar**: *thabyu*, *maisen* (Kachin), *khwati* (Kayin), *haprut* (Mon). **English**: elephant apple.

###### Range.

Temperate and tropical Asia. Found growing naturally in lower Myanmar, along woods, hills, and especially stream banks.

###### Uses.

*Fruit*: The green fruit is used in preparations to regulate phlegm, reduce fevers, and alleviate shooting chest pains and fatigue. The fruit is mixed with rock sugar to make a cordial used to relieve coughs, bring down high fevers, and cleanse the bowels. The juice from the squeezed fruit is given as a remedy for epilepsy and rabies.

###### Note.

The medicinal uses of this species in India are discussed in Jain and DeFilipps (1991).

###### Reference.

Agricultural Corporation (1980).

### Dioscoreaceae (Yam family)

#### 1. *Dioscorea* L.

##### Dioscorea bulbifera

Taxon classificationPlantaeORDOFAMILIA

L.

###### Names.

**Myanmar**: *kway*, *ah-lu-thi*, *putsa-u*. **English**: aerial yam, air potato, potato yam.

###### Range.

Tropical Africa and Asia. In Myanmar, found in Chin, Kachin, Mandalay, Mon, Sagaing, and Shan.

###### Use.

In Upper Myanmar, the plant is considered to be a galactagogue.

###### Notes.

In China the tubers are considered cooling and antidotal; used internally and externally as remedies for sore throat, boils, swelling, and poisonous snakebites In the Philippines the powder obtained from scraping the axial fruit (bulblets) is rubbed on the abdomen (Perry 1980). Medicinal uses of this species in China are also discussed by Duke and Ayensu (1985). Medicinal uses of the species in India are discussed in Jain and DeFilipps (1991). Chemical constituents, pharmacological action, and medicinal use of this species in Indian Ayurveda are discussed in detail by Kapoor (1990). Indigenous medicinal uses of this species in the Andaman and Nicobar Islands (India) are described by Dagar and Singh (1999). The medicinal uses of this plant in the Caribbean region, as well as its chemistry, biological activity, toxicity and dosages, are discussed by Germosén-Robineau (1997).

The tubers contain tannin, saponin, and alkaloids (*poisonous*); also, both the bulblets and the tubers contain a *toxic principle* removable by repeated washings and cooking (Perry 1980). The chemistry, pharmacology, toxicology, and use of this species as a hunting poison and medicinal plant in Africa are discussed by Neuwinger (1994). The toxic properties, symptoms, treatment and beneficial uses of this plant, parts of which are poisonous, are discussed by Nellis (1997).

###### References.

Perry (1980), Forest Department (1999).

##### Dioscorea pentaphylla

Taxon classificationPlantaeORDOFAMILIA

L.

###### Names.

**Myanmar**: *kyway-u*, *put-sa-u*. **English**: five-leaved yam.

###### Range.

Widespread- China, including Taiwan; Bangladesh, India, Indonesia, Japan (Okinawa), Laos, Malaysia, Myanmar, Nepal, New Guinea, Philippines, Vietnam; Africa, Australia, Pacific islands. In Myanmar, found in Bago, Kachin, Mandalay, and Yangon.

###### Use.

*Root*: Tuber used to reduce swellings.

###### Notes.

The species can be made edible by prolonged washing alternately in salt and fresh water and then cooked, or by prolonged boiling with ashes of wood. The plant is also used for some medicinal purposes (exact uses not listed in Perry 1980).

Tubers of the genus contain tannin, saponin, and alkaloids, some in greater, some in less quantity than others (the alkaloids are *poisonous*, but may be washed out in a long tedious process) (Perry 1980).

###### Reference.

Nordal (1963).

### Ebenaceae (Ebony family)

#### 1. *Diospyros* L.

##### Diospyros malabarica

Taxon classificationPlantaeORDOFAMILIA

(Desr.) Kostel. (= D. embryopteris Pers.; D. glutinosa J.Koenig ex Roxb.)

###### Names.

**Myanmar**: *bok-pyin*, *yengan-bok*. **English**: Indian persimmon, mountain ebony.

###### Range.

India to Indonesia. In Myanmar, found in Ayeyarwady, Mon, and Taninthayi.

###### Uses.

*Bark*: Used to treat diarrhea and chronic dysentery, and greatly diluted extract used as injections for vaginal discharge. *Fruit*: Unripe astringent fruit used for same purposes as bark. Juice of the fruit is used to treat sores and wounds.

###### Notes.

The medicinal uses of this species in India are discussed in Jain and DeFilipps (1991) as follows: The bark is an astringent, used for intermittent fever and dysentery; the fruit astringent, infusion of fruits is gargled for sore throat and aphthae, the juice is applied to ulcers and wounds; oil from the seed is used as a remedy for dysentery and diarrhea. Perry (1980) discusses the medicinal uses of the species in China and Indo-China as similar to those in Myanmar.

###### Reference.

Perry (1980).

##### Diospyros mollis

Taxon classificationPlantaeORDOFAMILIA

Griff.

###### Name.

**Myanmar**: *te*.

###### Range.

Myanmar and Thailand. In Myanmar, found in Bago and Mandalay.

###### Use.

*Fruit*: The fresh fruit, or an extract of the fruit is used as an anthelmintic.

###### Notes.

Perry (1980) discuses the uses of the fruit in Thailand and Indo-China, and the seed in Cambodia.

Reported chemical constituents of this species are tannins, sterols, organic acids, aphrogenic principle, invertine, emulsine, a hydroquinonic principle, and diospyroquinone. The vermicidal property of the fruit is due to the presence of diospyroquinone (Perry 1980).

###### Reference.

Perry (1980).

### Ericaceae (Heath family)

#### 1. *Rhododendron* L.

##### Rhododendron moulmainense

Taxon classificationPlantaeORDOFAMILIA

Hook.

###### Names.

**Myanmar**: *zalat-pyu*. **English**: Westland’s rhododendron.

###### Range.

Southern China, northeastern India, Indonesia, Malaysia, Myanmar, Thailand, and Vietnam. In Myanmar, it is found in Mon.

###### Use.

The plant has narcotic properties.

###### Notes.

Perry (1980) discusses several other members of the genus that are used for various medicinal purposes in East and Southeast Asian countries, including Korea, China, and the Philippines. She notes that honey collected where *Rhododendron moulmainense* is abundant is sometimes stupefying.

###### Reference.

Perry (1980).

### Euphorbiaceae (Spurge family)

#### 1. *Acalypha* L.

##### Acalypha indica

Taxon classificationPlantaeORDOFAMILIA

L.

###### Names.

**Myanmar**: *kyaung-yo-thay pin*, *kyaung-se-pin*, *kyaung-yo-the*. **English**: Indian acalypha, copperleaf.

###### Range.

Old World tropical regions. Found growing on plains all over Myanmar, except in cold mountainous regions.

###### Uses.

*Leaf*: A mixture of the juice and that of the leaves from the *neem* tree (*Azadirachta indica*) acts as an expectorant and is given for bronchitis, diarrhea, and vomiting. Cooked leaves are eaten to alleviate asthma, hypertension, impurities in the blood, and to treat various illnesses in infants. Other preparations are taken to relieve inflammation of the joints, fevers caused by chest colds and infections, asthma, and a burning sensation in the windpipe. A decoction is used as an emetic to cure pleurisy, cleanse and clear breathing passages, and alleviate swelling of the windpipe, as well as to cure asthma, hypertension, and skin problems caused by impurities in the blood. The juice is considered a remedy for ringworm, scabies, and rashes; a mixture of the juice and *neem* (*Azadirachta indica*) oil is used for various skin diseases that cause itching. A mixture of the leaves and castor oil is applied to relieve joint aches. Leaf juice is also used as eardrops for ear infections, earaches, and other ear problems. Crushed and applied as a poultice, leaves are used to heal sores. Stir-fried, they are eaten with large prawns to alleviate exhaustion and fatigue but with dried *nga-mway-toh* (*Mastacembelus armatus*) fish to prevent inflammation of the appendix; the same mixture is used to alleviate constipation, diarrhea, and nagging stomachaches. Boiled leaves made into a salad are eaten to treat lung disease, neurological disease, ringing in the ear, earache, gastric pain, and stomach-ache.

###### Notes.

The medicinal uses of this species in India are discussed in Jain and DeFilipps (1991). Perry (1980) lists the uses of the species in India, Indo-China, the Malay Peninsula, Indonesia, and the Philippines.

A cyanogenetic glucoside, triacetonamine, and quebrachitol have been islolated from South African material of this species (Perry 1980).

###### Reference.

Agricultural Corporation (1980).

##### Acalypha wilkesiana

Taxon classificationPlantaeORDOFAMILIA

Müll. Arg.

###### Names.

**Myanmar**: *saydan-kya*. **English**: copperleaf, Jacob’s coat, firedragon.

###### Range.

Pacific Islands, the exact origin is unknown. Cultivated in Myanmar.

###### Uses.

The species has medicinal uses in Myanmar, but Nordal (1963) does not list them.

###### Notes.

The species has medicinal uses for ache, swelling, as a testicle altschul, and as a bacterioside (chemical found in plant shown to be effective for this purpose) (Duke 2009).

Gallic acid, corilagin, and geraniin were isolated from an ethnol extract of the leaves of this species. These compounds were found to be responsible for the observed antimicorbioal activity (Adesina et al. 2000).

###### Reference.

Nordal (1963).

#### 2. *Chrozophora* Neck. ex A.Juss.

##### Chrozophora plicata

Taxon classificationPlantaeORDOFAMILIA

(Vahl) A.Juss. ex Spreng.

###### Name.

**Myanmar**: *gyo-sagauk*.

###### Range.

Tropical Africa to northern South Africa, Egypt, Syria, Palestine and western Arabia. In Myanmar, found in Bago and Mandalay.

###### Uses.

*Whole plant*: Decoction taken for gonorrhea.

###### Reference.

Perry (1980).

#### 3. *Claoxylon* A.Juss.

##### Claoxylon indicum

Taxon classificationPlantaeORDOFAMILIA

Hassk. (= C. polot Merr.)

###### Name.

**English**: claoxylon.

###### Range.

China, India, Indonesia, Malaysia, New Guinea, Thailand, and Vietnam. In Myanmar, found in Bago, Kayin, Mon, Rakhine, and Taninthayi.

###### Uses.

*Bark* and *Leaf*: Finely ground and smeared on the chest to treat tightness. *Leaf*: Used as a purgative.

###### Notes.

In China a decoction of the leaf is taken internally for various ailments. From Hainan and Myanmar to Sumatra the leaves are used as a purgative, and the finely ground bark mixed with macerated leaves is rubbed onto the chest for congestion (Duke and Ayensu 1985).

###### Reference.

Perry (1980).

#### 4. *Croton* L.

##### Croton persimilis

Taxon classificationPlantaeORDOFAMILIA

Müll. Arg. (= C. oblongifolius Roxb.)

###### Names.

**Myanmar**: *thetyin-gyi*, *casauboh* (Mon), *ha-yung*, *mai-sat-lang* (Shan), *umawng* (Kachin). **English**: croton.

###### Range.

Nepal, India, Sri Lanka, Myanmar, southern China, and Indo-China. In Myanmar, found growing naturally throughout the country.

###### Uses.

Hot and bitter in taste, used to control flatulence, regulate bowels, and cure diarrhea, clotting of blood, dysentery and boils. The plant, either taken orally or as an external application, is also considered very useful for inflammation. *Bark*: Used to treat edemas with attendant fever. Made into paste to treat snakebites. Also used to treat enlarged liver, hepatitis, hepatomegaly, pyexia, and considered excellent antidote for snakebite. *Bark*, *Seed*, and *Root*: Used as a purgative, for liver disease, and high blood pressure. *Leaf*: Hot fomentations made and applied to relieve inflammation; crushed and applied as a poultice over old and rotting sores with pus; also used for scabies. Boiling the tender leaves and eating them with a dip used to regulate gas and bowels, and to treat stomachache associated with dysentery and stomachaches in general. *Fruit* and *Seed*: Both used as a purgative. *Seed*: Used for diarrhea and edema. *Root*: Used in making medicines for flatulence and disorders of phlegm. Can be soaked together with jaggery, and the liquid taken daily to regulate gas and bowels. It can also be used to cure alcoholism and protect against disease. Root and bark taken internally or used externally as a rub for inflammation or enlargement of the liver as well as for inflammation, edema, and pain in the joints. A paste made of the root and lime juice is taken for male related disorders and hemorrhoids. The root bark is employed for pneumonitis, hepatitis, hepatomegaly, and arthritis.

###### Notes.

Perry (1980) discusses the uses of the species in Indo-China. She also notes that *C. robustus* has medicinal uses in Myanmar, but does not specify what they are.

###### References.

Nordal (1963), Agricultural Corporation (1980), Perry (1980).

##### Croton tiglium

Taxon classificationPlantaeORDOFAMILIA

L.

###### Names.

**Myanmar**: *kanakho*, *mai-hkang*.

###### Range.

Temperate and tropical Asia. Can be cultivated in the hot and humid parts of Myanmar, to altitudes of 610 m.

###### Uses.

*Seed*: Bitter, used to stimulate appetite; correct imbalances in phlegm and gas; prevent jaundice, fainting, and facial paralysis; also taken as a laxative to rid the body of impurities. Ground seed paste is applied to scorpion stings to neutralize the venom. A mixture of oil from the seeds and ginger juice is used as medicine for whooping cough in children. One part of their oil is mixed with eight parts of coconut oil and used as a rub for aching joints. The oil can also be used for stomach disorders, hypertension, fevers, inflammation, infections, and diseases of the throat and ear.

###### Notes.

The medicinal uses of this species in India are discussed in Jain and DeFilipps (1991). Medicinal uses of this species in China are discussed in Duke and Ayensu (1985). The species is important medicinally and economically since the seeds yield croton oil, a powerful purgative (Bailey and Bailey 1976). Perry (1980) discusses the uses of the species on the Malay Peninsula, Indonesia, and in the Himalayas. She also notes that *all parts of the plant are somewhat poisonous*; *especially the seeds and oil*, which are also used in a fish and arrow poison.

Chemical work done on the seeds and oil “reveals two active principles, one purgative but with non-irritant properties, the other (resin) irritant or vesicant”. The oil also contains oleic, linolic, arachidic, myristic, stearic, palmitic, acetic and formic acids, with traces of lauric, tiglic, valeric and butyric acids. The kernel, in addition, contains two toxic proteins, croton-globulin and carton-albumin; sucrose; and a glycoside, crotonoside. “The glycoside, at least in small doses, has no harmful physiological action.” The leaves contain hydrogen cyanide and a triterpinoid (Perry 1980).

###### References.

Agricultural Corporation (1980), Forest Department (1999).

#### 5. *Euphorbia* L.

##### Euphorbia antiquorum

Taxon classificationPlantaeORDOFAMILIA

L.

###### Names.

**Myanmar**: *kun*, *tazaung-gyi*, *tazaung-pyathat*. **English**: milkhedge, fleshy spurge.

###### Range.

Native of Southeast Asia, especially India. Widespread in Myanmar.

###### Uses.

*Stem*: Branch sliced, dried, powdered, and administered to check profuse lochial discharge; *Sap*: Latex applied to warts. *Root*: Root bark used as a purgative.

###### Notes.

In India the whole plant used for skin infections; latex, for dropsy, as nerve tonic, and for bronchitis (with ginger and bulb of *Thysanolaena*); pith for syphilis, dropsy, anasarca; bark (in combination with bark of two other species) on venereal sores; and the leaf for deafness (Jain and DeFilipps 1991). In China the whole plant is used in a decoction to treat bladder inflammation; raw plant tissues are used internally for cholera; the stem latex is applied to warts, and the stem is compressed onto large boils (Duke and Ayensu 1985). Perry (1980) discusses the uses of this species in China, Indo-China, and the Malay Peninsula, as well as Myanmar.

Chemical constituents of the plant include cycloartenol, epifriedelanol, euphol, euphorbol, friedelan-3alpha-ol, friedelan-3beta-ol, taraxerol, and taraxerone (Duke and Ayensu 1985). The therapeutic use of this species is about the same as *E. neriifolia* (see below), but it is somewhat more poisonous; it is also used as a fish poison. This species and *E. neriifolia* appear to contain the same elements and have similar poisonous properties. Reported constituents of the latex are euphorbon, resin, rubber, malic acid, and gum (Perry 1980).

###### Reference.

Perry (1980).

##### Euphorbia hirta

Taxon classificationPlantaeORDOFAMILIA

L.

###### Names.

**Myanmar**: *kywai-kyaung min hsay*, *kywai-kyaung min thay*, *hsay min kyaung*, *kanah-tanow pryin* (Mon). **English**: Australian asthma weed, milk weed, Queensland asthma herb.

###### Range.

Pantropical weed. Widely distributed throughout Myanmar, growing naturally.

###### Uses.

*Whole plant*: A decoction is given for asthma and bronchitis. New mothers eat it any way they like to promote lactation. In a salad or with fish paste or fish sauce dip, it is consumed to alleviate stomach pains from heat stroke, as well as to strengthen nerves and blood vessels along the breathing passages. Juice from crushing the five parts (stem, leaf, flower, fruit, and root) is used to treat fatigue in asthmatics, is taken with water after every meal to promote digestion, and is considered beneficial for the heart and the air passages. It is used to treat vomiting of blood, loose stools, and chest pain. *Sap*: Described as sweet, bitter, sharp and salty, with heating properties, it is known to increase semen and stabilize pregnancy, as well as to alleviate fevers, coughs, colds, and runny noses. Applied topically, it is used to clear pimples and scabies. *Leaf*: Sweet and astringent, used to control heat, and also applied topically for ringworm, scabies, itching, and other skin disorders. The juice is used widely to treat mucus within the chest in, inflammation of air passage, and coughs in children. A decoction of the leaves is mixed with a large amount of sugar and ingested to alleviate bleeding dysentery.

###### Notes.

The medicinal uses of this species in South China, the Malay Peninsula, Indonesia, and Indo-China are discussed in Perry (1980).

Reported chemical constituents of the species include quercetin, triacontane, phytosterol, phytosterolin, jambulol (now identified as ellagic acid); melissic, gallic, palmitic, linolic, and oleic acids; euphosterol; also an alkaloid, xanthorhamnine. The plant also contains hydrogen cyanide and a triterpinoid, an extract of which “has some antibiotic activity on *Staphylococcus*” (Perry 1980). A pharmacognostical profile including medicinal uses of this plant in Africa is given in Iwu (1993).

###### Reference.

Agricultural Corporation (1980).

##### Euphorbia neriifolia

Taxon classificationPlantaeORDOFAMILIA

L.

###### Names.

**Myanmar**: *shazaung-myin-na*, *ta-zaung*, *zizaung*. **English**: hedge euphorbia, Indian spurgetree, oleander-leaved euphorbia.

###### Range.

India; perhaps also East Indies. Cultivated in Myanmar and elsewhere.

###### Use.

*Leaf*: Used to treat asthma.

###### Note.

Perry (1980) discusses the uses of the species in Taiwan, the Malay Peninsula, the Philippines, and Indonesia.

###### Reference.

Forest Department (1999).

#### 6. *Jatropha* L.

##### Jatropha curcas

Taxon classificationPlantaeORDOFAMILIA

L.

###### Names.

**Myanmar**: *thin-baw-kyetsy*, *kyetsi-gyi*, *kyet-su-gyi*, *makman-yoo*, *siyo-kyetsu*, *thinbaw-kyetsu*, *tun-kong*. **English**: Barbados nut, physic nut, purging nut, wild oil nut.

###### Range.

Tropical America. Cultivated in Myanmar.

###### Uses.

*Leaf*: Used as a galactagogue. *Fruit* and *Seed*: Employed as an anthelmintic. *Seed*: Aperient.

###### Notes.

Medicinal uses of this species in India are discussed in Jain and DeFilipps (1991). Indigenous medicinal uses of this species in the Andaman and Nicobar Islands (India) are described by Dagar and Singh (1999). Medicinal uses of this species in China are discussed by Duke and Ayensu (1985).

The medicinal uses of this plant in the Caribbean region, as well as its chemistry, biological activity, toxicity and dosages, are discussed by Germosén-Robineau (1997). The chemistry, pharmacology, history and medicinal uses of this species in Latin America are discussed in detail by Gupta (1995). The chemical constituents, pharmacological activities, and traditional medicinal uses of this plant on a worldwide basis are discussed in detail by Ross (1999). The chemistry, pharmacology, toxicology, and use of this species as a hunting poison and medicinal plant in Africa are discussed by Neuwinger (1994).

A pharmacognostical profile including medicinal uses of this plant in Africa is given in Iwu (1993). The toxic properties, symptoms, treatment and beneficial uses of this plant, parts of which are poisonous, are discussed by Nellis (1997). Data on the propagation, seed treatment and agricultural management of this species are given by Katende et al. (1995). Worldwide medicinal usage, chemical composition and toxicity of this species are discussed by Duke (1986). Seeds of Jatropha curcas contain curcin, a poisonous chemical constituent which can cause death if ingested; plant sap can cause irritating dermatitis (Lan et al. 1998).

###### References.

Nordal (1963), Perry (1980).

##### Jatropha gossypiifolia

Taxon classificationPlantaeORDOFAMILIA

L.

###### Names.

**Myanmar**: *kyetsu-kanako*, *taw-kanako*, *thinbaw-kanako*. **English**: physic nut, bellyache bush.

###### Range.

Mexico to South America; West Indies. Cultivated in Myanmar.

###### Uses.

*Leaf*: Used to treat skin diseases. *Root*: Used as a purgative.

###### Notes.

Medicinal uses of this species in India are discussed in Jain and DeFilipps (1991). Indigenous medicinal uses of this species in the Andaman and Nicobar Islands (India) are described by Dagar and Singh (1999).

The medicinal uses of this plant in the Caribbean region, as well as its chemistry, biological activity, toxicity and dosages, are discussed by Germosén-Robineau (1997). The chemistry, pharmacology, history and medicinal uses of this species in Latin America are discussed in detail by Gupta (1995). The toxic properties, symptoms, treatment and beneficial uses of this plant, parts of which are *poisonous*, are discussed by Nellis (1997). Worldwide medicinal usage, chemical composition and toxicity of this species are discussed by Duke (1986). This species produces jatrophone, a macrocyclic diterpenoid with tumor inhibiting properties (Mors et al. 2000).

###### Reference.

Nordal (1963).

##### Jatropha multifida

Taxon classificationPlantaeORDOFAMILIA

L.

###### Names.

**Myanmar**: *bein-hpo*, *semakhan*. **English**: coral bush, physic nut.

###### Range.

Tropical and subtropical America. Cultivated in Myanmar.

###### Use.

*Whole plant*: Latex used for treating granulation. Unspecified plant part used in treatment of fractures, to improve union of bones. Decoction taken orally for fractures, external application with resin.

###### Notes.

Medicinal uses of this species in India are discussed in Jain and DeFilipps (1991) as follows: Latex from the stem is applied to skin ulcerations and wounds; the leaf is used for scabies; the seed is used as an emetic and purgative; and the seed-oil is used internally and externally as an aborifacient. Indigenous medicinal uses of this species in the Andaman and Nicobar Islands (India) are discussed by Dagar and Singh (1999).

###### References.

Forest Department (1999), Ministry of Health (2001).

#### 7. *Mallotus* Lour.

##### Mallotus nudiflorus

Taxon classificationPlantaeORDOFAMILIA

(L.) Kulju & Welzen (= Trewia nudiflora L.;)

###### Names.

**Myanmar**: *setkadon*, *ye-hmyok*. **English**: petari.

###### Range.

China, South and Southeast Asia. Naturalized in Myanmar.

###### Use.

*Root*: Used to treat gout.

###### Notes.

In India the whole plant is used to remedy bile, phlegm, and swellings; a decoction of the root is applied to rheumatic areas and gout, as well as drunk to relieve flatulence (Jain and DeFilipps 1991). The species is used in other countries as well for the previously cited reasons. It is also used as a carminative (Duke 2009).

###### Reference.

Perry (1980).

##### Mallotus philippensis

Taxon classificationPlantaeORDOFAMILIA

(Lam.) Müll. Arg.

###### Names.

**Myanmar**: *hpadawng*, *hpawng-awn*, *mai-hpawng-tun*, *palannwe*, *po-thi-din*, *taw-thi-din*. **English**: Indian kamala, kamala tree.

###### Range.

From southern China to New Guinea and Australia. In Myanmar, found in Bago, Chin, Mandalay, and Yangon.

###### Uses.

Fruit used as an anthelmintic and laxative.

###### Notes.

The medicinal uses of this species in India are discussed in Jain and DeFilipps (1991) as follows: The glandular hairs on the fruit used as a purgative. They are said to destroy tapeworms, and are also employed on ringworm, scabies and other skin diseases. Additionally, they have been found to reduce fertility in experimental animals. The fruit is used for dysentery and constipation; the root as tonic for pregnant women. Medicinal uses of this species in China are discussed in Duke and Ayensu (1985). Perry (1980) discusses the species uses in China, India, the Malay Peninsula, and the Philippines. She especially notes that, in the Philippines, the glands of *M. philippensis* are mixed with the charred bark and flowers of *Pterospermum diversifolium*, and employed in smallpox to cause suppuration.

Research has shown that the dye from this species is an antioxidant; rottlerin is an antifertility factor, isorottlerin less active; the fruit extract is bactericidal; and the seeds contain 18.5–20% protein, 23.7–25.8% fat (Duke and Ayensu 1985). In the Philippines an extract of kamala (the powder), the active principle of which is rottlerin, and hexachlorethane “gave encouraging results in treating fascioliasis (liver fluke infestation) in cattle and Indian buffaloes, with the conclusion that the effect of the drug deserved further study” (Perry 1980).

###### Reference.

Nordal (1963).

#### 8. *Ricinus* L.

##### Ricinus communis

Taxon classificationPlantaeORDOFAMILIA

L.

###### Names.

**Myanmar**: *kyet-hsu*, *kyetsu*, *thinbaw kyet-hsu*, *kyet-hsu yoe-ni*, *shapawing* (Kachin), *tanah toung* (Mon), *toon* (Mon), *mai-kong-leng* (Shan). **English**: castor bean, castor oil plant, wonder-tree.

###### Range.

Tropical Africa. Although found wild in nature, now cultivated widely for the extraction of oil from the seeds. In Myanmar, does well in Sagaing, Mandalay, and Shan; prefers a warm temperate climate, but can also thrive in hot and dry areas. Found growing naturally on the banks of rivers, lakes and streams.

###### Uses.

Sweet and rather bitter with heating properties, the plant is considered difficult to digest but generally effective at increasing sperm, regulating bowel movements, and controlling flatulence and phlegm. *Leaf*: Used in remedies for headaches and in poultices for sores and wounds. A decoction of leaves reduced to one-third the starting volume is ingested to alleviate strong gas and phlegm; also used for testes enlargement, bladder aches and pains, sore throat, and bile problems. *Seed*: They and their oil (*lethal in their natural form*) are used in oral medications *after detoxifying*. The detoxified, ground seeds are applied as a paste to neutralize venom from scorpion stings. They are also employed in anthelmintic remedies; and in medicines for flatulence, fever, cough, stomach bloating, liver disease, shooting abdominal pains, dysentery, back and bladder conditions, head-aches, asthma, leprosy, edema, and a general weakening malaise in men. Detoxified seed oil is additionally used to make laxative preparations, as well as to facilitate childbirth, and to strengthen hair.

###### Notes.

Medicinal uses of this species in India are discussed in Jain and DeFilipps (1991). Chemical constituents, pharmacological action, and medicinal use of this species in Indian Ayurveda are discussed in detail by Kapoor (1990). Indigenous medicinal uses of this species in the Andaman and Nicobar Islands (India) are described by Dagar and Singh (1999). Medicinal uses of this species in China are discussed by Duke and Ayensu (1985).

The medicinal uses of this plant in the Caribbean region, as well as its chemistry, biological activity, toxicity and dosages, are discussed by Germosén-Robineau (1997). Traditional medicinal uses, chemical constituents and pharmacological activity of this species are discussed by Ross (2001). The chemistry, pharmacology, history and medicinal uses of this species in Latin America are discussed in detail by Gupta (1995). Worldwide medicinal usage, chemical composition and toxicity of this species are discussed by Duke (1986).

A pharmacognostical profile including medicinal uses of this plant in Africa is given in Iwu (1993). The toxic properties, symptoms, treatment and beneficial uses of this plant, parts of which are poisonous, are discussed by Nellis (1997). Data on the propagation, seed treatment and agricultural management of this species are given by Katende et al. (1995) and Bekele-Tesemma (1993). Details of the active chemical compounds, effects, herbal usage and pharmacological literature of this plant are given in Fleming (2000).

The plant and its seeds can cause skin irritation (contact dermatitis). “The pomace (residue after extracting the oil from castor beans) can cause asthma, urticaria, and dermatitis among castor oil extractors…(Castor oil used in) lipstick can also be the source of contact dermatitis resulting in cheilitis…Cases of allergy to castor oil, contact dermatitis of the face due to a makeup remover and contact dermatitis due to sulfonated castor oil have recently been described…Ricinoleic acid has been claimed to be the agent causing lipstick dermatitis.” The seed contains a poisonous substance, the protein “ricin”, which is not present in castor oil, but is “probably responsible for certain allergies related to the plant” (Benezra et al. 1985).

It has been reported that “Ricin”, a white crystalline compound isolated from castor beans (*Ricinus communis*), is listed by the FBI (Federal Bureau of Investigation, USA) as the third most *poisonous* substance known, behind plutonium and the botulism toxin. Toxicity of this species is discussed by Bruneton (1999). Ricin and ricinine contained in the seeds and leaves make this one of the most toxic plants known, and as noted by Lan et al. (1998): “A single seed of 0.25 g contains a lethal dose. The toxins are stable to proteolytic enzymes and hence are not destroyed when taken orally.”

###### References.

Nordal (1963), Agricultural Corporation (1980).

### Fabaceae (Bean family)

#### 1. *Abrus* Adans.

##### Abrus precatorius

Taxon classificationPlantaeORDOFAMILIA

L.

###### Names.

**Myanmar**: *chek-awn*, *ywe*, *ywe-nge*, *ywe-nwe*, *ywenge*. **English**: chicken eyes, crab eyes, jequirity, red bean vine, rosary pea.

###### Range.

Pantropical; widely naturalized. Widely distributed in Myanmar.

###### Uses.

(*Whole plant*: *poisonous*). *Leaf*: Used to cure a sore throat. *Seed*: Emetic and purgative. *Root*: Employed as an expectorant. After being crushed with water and steamed, the distillate is taken with sugar to treat hemorrhoids. Soaked in water overnight, filtered through a cloth, and the filtered liquid taken once in the morning and once in the evening to treat white vaginal discharge. *Leaf*: Crushed together with mustard oil and used either by rubbing on, or tied around as a poultice, to cure swollen joints and stiff muscles. Crushed with oil and rubbed on to treat aches and pains. Juice from squeezing the leaves together with milk can treat excessive urination in diabetics. *Seed*: Made into a powder and inhaled to cure severe headaches. Making the seeds and root into a powder and taking the mixture with coconut water can treat hemorrhoids.

###### Notes.

Medicinal uses of this species in India are discussed in Jain and DeFilipps (1991). Chemical constituents, pharmacological action, and medicinal use of this species in Indian Ayurveda are discussed in detail by Kapoor (1990). Indigenous medicinal uses of this species in the Andaman and Nicobar Islands (India) are described by Dagar and Singh (1999). Medicinal uses of this species in China are discussed by Duke and Ayensu (1985).

Since the broken seed is conventionally known to be poisonous due to the necrotic action of its constituent chemical “abrin”, care must be taken in its use. Symptoms of the poisoning (which can happen, for example, from chewing or sucking on a necklace made of the beads) appear after a latent period which can vary from three hours to two days, whereupon severe gastroenteritis with diarrhea, cramps and vomiting occurs. Bleeding from the retina (of the eye) and serous (mucous) membranes is a characteristic symptom of the poisoning. In this connection it is notable that the seeds, under the name “semen jequirity”, were formerly used in medicine, especially ophthalmology, to cause inflammation of mucosa (Frohne and Pfander 1984). Frohne and Pfander (1984) further advise that: “On the other hand, intact seeds, because of their hard testa (seed coat), when swallowed whole are harmless.”

“The seeds are poisonous, but it is said that, if boiled, their toxic principle (toxalbumin) is destroyed. After this precautionary measure the seeds have been (known to be) boiled again in milk (which is used) as a tonic [in Dominica].” (Honychurch 1980). The chemical constituents, pharmacological activities, and traditional medicinal uses of this plant on a worldwide basis are discussed in detail by Ross (1999).

A pharmacognostical profile including medicinal uses of this plant in Africa is given in Iwu (1993). The toxic properties, symptoms, treatment and beneficial uses of the plant, parts of which are poisonous, are discussed by Nellis (1997). Worldwide medicinal usage, chemical composition and toxicity of this species are discussed by Duke (1986). In connection with this plant’s usage in ophthalmology, a seed infusion was formerly used in Brazil to treat trachoma and corneal opacity, but the use of it was abandoned since it was too dangerous, sometimes leading to loss of eyesight (Mors et al. 2000). Details of the active chemical compounds, effects, herbal usage and pharmacological literature of this plant are given in Fleming (2000). Toxicity of this species is discussed by Bruneton (1999).

###### References.

Nordal (1963), Agricultural Corporation (1980), Perry (1980).

#### 2. *Acacia* Mill.

##### Acacia catechu

Taxon classificationPlantaeORDOFAMILIA

(L.f.) Willd.

###### Names.

**Myanmar**: *mung-ting*, *nya*, *sha*, *shaji*, *tun-sa-se*. **English**: black cutch, catechu, cutch, wadalee-gum tree.

###### Range.

West Pakistan to Myanmar. In Myanmar, found in Magway and Mandalay.

###### Uses.

Bark used as an astringent. *Wood*: An extract is used to treat ulcers and chest problems.

###### Notes.

In India the bark is used to treat sores in the mouth, chest pain, strangulation of the intestine, and to facilitate childbirth. The heartwood is applied in a thick decoction for cancerous sores (Jain and DeFilipps 1991). In China the resin is used as a febrifuge, sialogogue, stimulant, styptic, antiphlogistic, astringent, corrective, and expectorant (Duke and Ayensu 1985). Perry (1980) also discusses the medicinal uses of the species in China.

The species contains tannin and catechin (Duke and Ayensu 1985). Reported chemical constituents also include catechutannic acid, acacatechin, catechu red, and quercetin. In research on vitamin P, “the isomer 1-epi-catechin is reported to be especially active even in minute doses.”, and is “The most important source of this substance in the heartwood of *A. catechu*” (Perry 1980).

###### References.

Nordal (1963), Perry (1980).

##### Acacia concinna

Taxon classificationPlantaeORDOFAMILIA

(Willd.) DC.

###### Names.

**Myanmar**: *hpah-ha* (Kachin), *hing-hang* (Chin), *hla pruckkha* (Mon), *sot lapoot* (Mon), *janah lapoot* (Mon), *hpak ha* (Shan), *sum-hkawn* (Shan), *kin-pun chin*, *kinmun-gyin*. **English**: soap pod.

###### Range.

Tropical and temperate Asia. Grows naturally throughout Myanmar, but most commonly in tropical evergreen forests; also cultivated.

###### Uses.

*Leaf*: Sour, with heating properties. Used to treat symptoms of heat stroke and to relieve diarrhea. The liquid from lightly boiling the leaves in water is used to treat malaria, as well as constipation and bloating. A mixture made with salt, tamarind (*Tamarindus indica*) fruit, and chili pepper, crushed together with the young leaves that have soaked in black pepper water, is taken to alleviate symptoms of jaundice and gall bladder disease. The young leaves are also soaked in water overnight and taken to cure maladies that cause fatigue and bloating. Additionally, they are crushed and applied externally to alleviate symptoms caused by a swollen liver. *Flower*: With cooling properties, the sweet flowers are used to reduce phlegm. *Fruit*: Bitter and with cooling properties, used to treat skin infections and promote digestion as well as to alleviate constipation, gastric disease, stomachaches caused by gas, and circulatory problems. The ripe fruit is used as detergent for washing hair. *Leaf* and *Fruit*: A decoction of leaves and fruits is taken for constipation. A decoction of fruit is used in shampoo to strengthen the hair. Crushed fruit, applied topically as a remedy for skin problems, is also an ingredient in preparations used to neutralize venomous snakebites. One cup of liquid from the fruit decoction is used to induce vomiting to save those who have attempted suicide by ingesting arsenic and lime juice.

###### Notes.

The medicinal uses of this species in India are discussed in Jain and DeFilipps (1991). The medicinal uses of the species in China, Indo-China, the Malay Peninsula, and Indonesia are discussed in Perry (1980).

###### References.

Agricultural Corporation (1980), Perry (1980).

##### Acacia farnesiana

Taxon classificationPlantaeORDOFAMILIA

(L.) Willd.

###### Names.

**Myanmar**: *nan-lon-kyaing*, *mawk-nawn-hkam* (Shan). **English**: cassie, sponge-tree, sweet acacia, West Indian blackthorn.

###### Range.

Subtropical and tropical America; now pantropical. Cultivated in Myanmar.

###### Uses.

*Bark*: Sharp and bitter with heating properties. Effective against poisons and beneficial in treating abnormalities in the blood, itching and sores. Liquid from boiling the bark in water down to half used as mouthwash or held in the mouth to treat toothaches, inflammation, infections and bleeding of the gums. Also, bark boiled and a small amount of the liquid taken to treat severe diarrhea. *Sap*: Said to give vitality and increase virility. *Leaf*: Crushed tender leaves are made into balls and taken, one in morning and one at night, to treat gonorrhea. *Root*: A paste is made and applied to the hooves of cattle to kill or prevent an attack of parasites.

###### Notes.

The medicinal uses of this species in India are discussed in Jain and DeFilipps (1991). Medicinal uses of this species in China are discussed in Duke and Ayensu (1985). Medicinal uses of the species in Indo-China, the Malay Peninsula, Indonesia, and the Philippines are discussed in Perry (1980).

The essence contains alcohol, sesquiterpene, and farnesol (Perry 1980).

###### References.

Agricultural Corporation (1980), Perry (1980), Forest Department (1999).

##### Acacia leucophloea

Taxon classificationPlantaeORDOFAMILIA

(Roxb.) Willd.

###### Names.

**Myanmar**: *tanaung*. **English**: white-barked acacia.

###### Range.

Western Pakistan, India, Sri Lanka, Myanmar, Siam, Indonesia, and Java. In Myanmar, found in Bago, Magway, Mandalay, and Shan.

###### Use.

*Bark*: Used as an astringent.

###### Note.

In India the bark is used as an astringent (Jain and DeFilipps 1991).

###### References.

Nordal (1963), Perry (1980).

##### Acacia nilotica

Taxon classificationPlantaeORDOFAMILIA

(L.) Delile (= A. arabica (Lam.) Willd.)

###### Names.

**Myanmar**: *babu*, *babul*, *subyu*. **English**: babul, gum-arabic, Indian gum tree, suntwood.

###### Range.

Tropical Africa; widely naturalized in India. Naturalized in Myanmar.

###### Use.

*Bark*: Used as an astringent.

###### Note.

In India the bark is employed as an astringent (Jain and DeFilipps 1991).

###### Reference.

Nordal (1963).

##### Acacia pennata

Taxon classificationPlantaeORDOFAMILIA

(L.) Willd.

###### Names.

**Myanmar**: *hsu bok gyi*, *htaura* (Kachin), *hangnan* (Chin), *hla-pruck-hka-hnoke* (Mon), *hpak-ha-awn* (Shan), *suboke-gyi*, *suyit*. **Thai**: *cha-om*.

###### Range.

In Asia, found in Bangladesh, Bhutan, Cambodia, China, India, Laos, Myanmar, Sri Lanka, Thailand, and Vietnam; also Indian Ocean- Andaman Islands. In Myanmar, found growing naturally throughout the country, but also cultivated.

###### Uses.

*Bark*: Used to treat asthma and bronchitis. Mixed with other medicinal ingredients to neutralize snake venom. *Leaf*: Ingested to prevent formation of calluses and to control gas, as well as to treat indigestion and bleeding gums. *Leaf* and *Root*: Bitter and astringent, they are employed to correct irregularities in the blood, treat gas and bile problems, relieve coughs, stimulate appetite, and alleviate female disorders. *Root*: Made into a paste, together with the gall bladder of a python, and used to cure tongue sores or roughness. Also, an ingredient in medicines used to treat urinary disorders and enlargement of the testicles.

###### Note.

In India the bark is used for dandruff and as an antidote to snake poison (Jain and DeFilipps 1991).

###### References.

Nordal (1963), Agricultural Corporation (1980).

#### 3. *Adenanthera* L.

##### Adenanthera pavonina

Taxon classificationPlantaeORDOFAMILIA

L.

###### Names.

**Myanmar**: *mai-chek*, *ywe*, *ywe-gyi*, *ywe-ni*. **English**: bead tree, coral pea, coral wood, red sandalwood.

###### Range.

Southeastern Asia- primarily in India, southeastern China and Malasia to the Moluccas. Widely distributed in Myanmar.

###### Use.

*Seed*: Used for poulticing.

###### Notes.

Perry (1980) discusses the medicinal uses of the species on the Malay Peninsula, in Indonesia, and in the Philippines.

“An alcoholic extract of air-dried leaves showed an alkaloidal substance” (Perry 1980).

###### Reference.

Perry (1980).

#### 4. *Albizia* Durazz.

##### Albizia lebbeck

Taxon classificationPlantaeORDOFAMILIA

(L.) Benth.

###### Names.

**Myanmar**: *anya-kokk*, *kokko*. **English**: woman’s tongue.

###### Range.

India and Southeast Asia.

###### Uses.

*Bark*: Used to treat dysentery and boils. *Leaf* and *Seed*: Used for ophthalmia.

###### Notes.

In India the bark is used for diarrhea and dysentery; the leaf for night blindness; the flower is put on boils, carbuncles, swellings; the seed is used for plies, diarrhea, and gonorrhea; and the root is placed on spongy, ulcerated gums (Jain and DeFilipps 1991). Indigenous medicinal uses of this species in the Andaman and Nicobar Islands (India) are described by Dagar and Singh (1999). In Indo-China the bark and seeds are used to treat dysentery, diarrhea, and hemorrhoids; the flowers are emollient, and applied in poultices to boils (Perry 1980).

###### References.

Nordal (1963), Perry (1980).

##### Albizia odoratissima

Taxon classificationPlantaeORDOFAMILIA

(L.f.) Benth.

###### Names.

**Myanmar**: *mai-kying-lwai*, *mai-tawn*, *meik-kye*, *taung-magyi*, *thit-magyi*. **English**: Ceylon rosewood.

###### Range.

Sri Lanka and India to Thailand. Widely distributed in Myanmar.

###### Uses.

*Bark*: Considered a remedy for ulcers; *Leaf*: Used to treat coughs.

###### Notes.

In India the bark, externally applied, is considered a good remedy for leprosy and for persistent ulcers; the leaf is applied as a poultice for ulcers (Jain and DeFilipps 1991).

The bark is rich in tannin (Perry 1980).

###### Reference.

Perry (1980).

#### 5. *Alysicarpus* Desv.

##### Alysicarpus vaginalis

Taxon classificationPlantaeORDOFAMILIA

(L.) DC.

###### Names.

**Myanmar**: *than-manaing-kyauk-manaing*.

###### Range.

Paleotropics. Found naturally in Myanmar, especially in the hot regions.

###### Use.

Has binding properties, brings down edema, causes dullness, cures diarrhea, dysentery, kidney stones and inability to pass urine. It also draws out the pus from sores. *Leaf*: Giving children the juice (squeezed from the leaves) in milk will cure them of dull stomach pains. Taking the dried leaves soaked in water will cure such disorders as diarrhea, dysentery, passing of blood, and white vaginal discharge. *Whole plant*: The juice from the plant can be boiled or the dried parts taken as tea to cure urinary disorders and gallstones. Fresh plant can be mixed in equal amounts with cooked rice, crushed and applied as a poultice to cure breast sores as it will draw out the pus.

###### Reference.

Agricultural Corporation (1980).

#### 6. *Amherstia* Wall.

##### Amherstia nobilis

Taxon classificationPlantaeORDOFAMILIA

Wall.

###### Names.

**Myanmar**: *thawka*, *thawka-gyi*. **English**: pride of Burma, queen of flowering trees.

###### Range.

Endemic to Myanmar (temperate southeastern Asia). Found in southern Myanmar, in Kayin and Taninthayi.

###### Uses.

Plant used for medicinal purposes (exact uses not given in Nordal 1963).

###### Reference.

Nordal (1963).

#### 7. *Arachis* L.

##### Arachis hypogaea

Taxon classificationPlantaeORDOFAMILIA

L.

###### Names.

**Myanmar**: *myay-pe*. **English**: earth nut, grass nut, groundnut, monkey nut, peanut.

###### Range.

Southern Brazil. Now widely cultivated throughout the tropics. Cultivated in Myanmar.

###### Use.

*Seed*: Used for production of peanut oil. Oil aperient, emollient.

###### Notes.

In India the fruit is used as an astringent (its oil is also astringent to the bowels), an aperient, and an emollient; also, unripe nuts are used for a lactagogue (Jain and DeFilipps 1991). Indigenous medicinal uses of this species in the Andaman and Nicobar Islands (India) are described by Dagar and Singh (1999). Medicinal uses of this species in China are discussed by Duke and Ayensu (1985). These include the use of the seed for an oil aperient, emollient, and for gonorrhea (given in milk); applied externally for rheumatism; considered demulcent, pectoral, and peptic. “ In China this widely cultivated species is considered to be nutritive, peptic, demulcent, and pectoral (Perry 1980).

“The oilseed cake is a good source of the amino acid arginine … and glutamic acid, which is used in treating mental deficiencies” (Perry 1980). Details of the active chemical compounds, effects, herbal usage and pharmacological literature of this plant are given in Fleming (2000). Toxicity of this species is discussed by Bruneton (1999).

###### Reference.

Nordal (1963).

#### 8. *Archidendron* F. Muell.

##### Archidendron jiringa

Taxon classificationPlantaeORDOFAMILIA

(Jack) I.C. Nielsen (= Pithecellobium lobatum Benth.)

###### Names.

**Myanmar**: *tanyin*, *danyin*. **English**: dog fruit, ngapi nut.

###### Range.

Believed to have originated, and is widely distributed in Indonesia, Malaysia and southern Thailand; also in Bangladesh. Reported from Myanmar.

###### Uses.

*Seed*: Used to treat diabetes; eaten, but *poisonous in any quantity*.

###### References.

Nordal (1963), Perry (1980).

#### 9. *Bauhinia* L.

##### Bauhinia acuminata

Taxon classificationPlantaeORDOFAMILIA

L.

###### Names.

**Myanmar**: *mahahlega-phyu*, *maha-hlega-byu*, *palan*, *swe-daw*. **English**: dwarf white bauhinia.

###### Range.

India, Myanmar, China, Malaysia. Widely distributed in Myanmar.

###### Conservation status.

Least Concern [LC] (IUCN 2017).

###### Use.

*Flower*: Used as a laxative.

###### Notes.

Root extract used as a poultice.

The rhizomes and root have been used for their insecticidal properties and have shown antifungal activity as well. Chemicals found in this species have been shown to be effective in the treatment of cold, cough, and sore throat; also for cataplasm and ulcers (Duke 2009).

###### Reference.

Nordal (1963).

##### Bauhinia purpurea

Taxon classificationPlantaeORDOFAMILIA

L.

###### Names.

**Myanmar**: *maha-hlega-ni*, *maha-hlega-byu*, *swe-daw*, *swedaw-ni*. **English**: butterfly tree, camel’s foot tree.

###### Range.

India, Myanmar, China, Malaysia. Cultivated in Myanmar.

###### Conservation status.

Least Concern [LC] (IUCN 2017).

###### Uses.

*Bark*: Astringent. *Flower*: Employed as a laxative.

###### Note.

In India the bark is used as an ingredient in medicine for dropsy, scorpion sting and insect bites, rheumatism, convulsions, stomach tumors, and as an antidote to certain toxins and poisons; the flower is used for indigestion (Jain and DeFilipps 1991).

###### References.

Nordal (1963), Perry (1980).

#### 10. *Butea* Roxb. ex Willd.

##### Butea monosperma

Taxon classificationPlantaeORDOFAMILIA

(Lam.) Taub. (= B. frondosa Roxb.)

###### Names.

**Myanmar**: *paukpin*, *shagan changgan* (Kachin), *pawpan* (Kayin), *tanom khapore* (Mon), *kao mai*, *kikao*, *maikao* (Shan). **English**: bastard-teak, flame-of-the-forest.

###### Range.

Tropical Asia. Found growing naturally throughout Myanmar, with the exception of the mountainous areas; grows most commonly by the sides of streams, rivers, ponds, and lakes.

###### Uses.

The parts are used in preparations stimulating digestion, increasing sperm production, promoting repair of broken bone, and improving urinary flow. *Bark*: Knobs are powdered, rolled in honey, and formed into pellets that are taken for strength and longevity. *Sap*: Fresh sap is applied topically as an ointment to relieve sores, rashes, and bumps. It is also used to make remedies taken orally for diarrhea. *Gum* and *Leaf*: Used as an astringent. *Leaf*: Used to make tonics. *Flower*: Liquid from soaking flowers overnight in cold water is mixed with sugar and taken orally to alleviate anal pain, blood in the urine, and nosebleeds. Flowers stewed in water are applied to the navel area while still warm to ease bladder inflammation and promote urination. The dried flowers are brewed into a tea taken to relieve fatigue, as well as to cleanse the blood and body systems. The flowers are also used in remedies for urinary infections and leprosy. *Seed*: An ointment made from the crushed seeds mixed with lime juice is used for ringworm. After soaking in water and removing the seed coats, the inner seed kernels are dried and powdered; the powder is given twice daily for four days, and a laxative is also given on the fourth day to expel intestinal worms. *Seed* and *Bark*: Used in remedies for neutralizing snake venoms.

###### Notes.

The medicinal uses of this species in India are discussed in Jain and DeFilipps (1991). In addition to Myanmar, medicinal uses of the species in Indo-China and Indonesia are discussed in Perry (1980).

###### References.

Agricultural Corporation (1980), Perry (1980).

##### Butea superba

Taxon classificationPlantaeORDOFAMILIA

Roxb.

###### Names.

**Myanmar**: *kao-hko*, *kosot-lot*, *pauk-new*, *paw-tohkaw*.

###### Range.

East Indies. In Myanmar, found in Bago, Mandalay, and Yangon.

###### Uses.

*Bark*: Used as a remedy for snake and other bites.

###### Notes.

In Indo-China a decoction of the stem and leaves is used in a local bath to treat hemorrhoids; also considered sedative in a large bath and sprinkled over the body of a person with convulsions. It is also used for erectile dysfunction (Perry 1980). Additionally, the species has been reported as used to treat diarrhea and dysuria (Duke 2009).

###### Reference.

Perry (1980).

#### 11. *Caesalpinia* L.

##### Caesalpinia pulcherrima

Taxon classificationPlantaeORDOFAMILIA

(L.) Sw. (= Poinciana pulcherrima L.)

###### Names.

**Myanmar**: *daung-sok*, *sein-pan-gale*. **English**: Barbados flower, dwarf poinciana, pride of Barbados.

###### Range.

Original range variously ascribed to tropical America or tropical Asia.

###### Uses.

*Bark*: Used as an astringent. *Leaf*: Used as a purgative and emmenagogue.

###### Notes.

Medicinal uses of this species in India are discussed in Jain and DeFilipps (1991).

###### Reference.

Nordal (1963).

#### 12. *Canavalia* DC.

##### Canavalia ensiformis

Taxon classificationPlantaeORDOFAMILIA

(L.) DC.

###### Names.

**Myanmar**: *pe-dalet*, *pe-dama*. **English**: horse bean, jack bean, sword bean.

###### Range.

Pantropical.

###### Uses.

*Fruit*: Used as tonic and digestive.

###### Note.

*Fresh immature seeds are considered poisonous*.

###### Notes.

In China, the whole plant is pounded and applied to boils; the seed is used as a bechic, stomatic, and tonic, also to strengthen the kidney (Duke and Ayensu 1985).

The medicinal uses of this plant in the Caribbean region, as well as its chemistry, biological activity, toxicity and dosages, are discussed by Germosén-Robineau (1997).

###### Reference.

Nordal (1963).

#### 13. *Cassia* L.

##### Cassia fistula

Taxon classificationPlantaeORDOFAMILIA

L.

###### Names.

**Myanmar**: *mai-lum*, *ngu*, *ngu-shwe*, *ngushwe-ama*, *ngu pin*, *gawhgu* (Kachin), *ka-zo* (Kayin). **English**: golden shower tree, Indian laburnum, pudding pipe tree, purging cassia.

###### Range.

India, Sri Lanka. Grows naturally all over Myanmar; prefers a hot and humid climate but also does well in hot and dry climates; can be found and cultivated up to 1220 m altitude; also grown as ornamental trees.

###### Uses.

*Whole plant*: The five parts – roots, bark, fruit, flower, and leaf – are mixed with water to form a paste and applied to ringworm, scabies, and skin disorders stemming from impurities in the blood. *Leaf*: Sweet yet bitter with a strong taste, act as a laxative. The tender leaves can be made into a soup and taken for constipation. Heated leaves are used as a poultice over swollen joints. Liquid from leaves stone-ground with vinegar is applied to treat leprosy and other skin diseases. Juice from crushed leaves is applied liberally as a remedy for herpes facialis. *Fruit*: Used as a laxative. Stimulates the tastebuds, alleviates leprosy, and controls phlegm. The pulp is taken either alone or mixed with an equal amount of tamarind (*Tamarindus indica*) fruit pulp to promote regular bowel movements. Paste from pulp is applied around the navel of infants to alleviate colic and bloated stomach; for others, the pulp paste is rubbed onto the navel to treat urinary disorders, pain around the urethra and during urination, and blood in the urine. Liquid from boiling the pulp is used as eardrops to clear infections. *Root*: Used as a purgative. Milk in which roots have been boiled is taken as a remedy for flatulence.

###### Notes.

Medicinal uses of this species in India are discussed in Jain and DeFilipps (1991). Chemical constituents, pharmacological action, and medicinal use of this species in Indian Ayurveda are discussed in detail by Kapoor (1990). Medicinal uses of this species in China are discussed by Duke and Ayensu (1985).

The chemistry, pharmacology, history, and medicinal uses of this species in Latin America are discussed in detail by Gupta (1995). Details of the active chemical compounds, effects, herbal usage, and pharmacological literature of this plant are given in Fleming (2000). *C. fistula* bark, leaves and seeds contain chrysarobin, an irritant and allergen (Lan et al. 1998).

###### References.

Nordal (1963), Agricultural Corporation (1980), Forest Department (1999).

#### 14. *Chamaecrista* (L.) Moench.

##### Chamaecrista pumila

Taxon classificationPlantaeORDOFAMILIA

(Lam.) K. Larsen (= Cassia pumila Lam.)

###### Names.

**Hawaiian**: *chota aura*

###### Range.

Tropical Asia, tropical Africa and Australia. In Myanmar found in Yangon.

###### Use.

*Seed*: A laxative.

###### Note.

The medicinal use of this species, as well as those of several other members of the genus, is noted in Perry (1980).

###### Reference.

Perry (1980).

#### 15. *Clitoria* L.

##### Clitoria ternatea

Taxon classificationPlantaeORDOFAMILIA

L.

###### Names.

**Myanmar**: *pe-nauk-ni*, *aug-mai-hpyu*, *aung-me-nyo*. **English**: blue pea, butterfly pea.

###### Range.

Origin uncertain, probably tropical Africa or Asia. In Myanmar, found in Kachin, Mandalay, Sagaing, and Yangon.

###### Uses.

*Whole plant*: The powder and the powder of *eikthara-mooli* (*Aristolochia indica*) can be mixed and taken to neutralize snake venom. *Leaf*: Crushed and placed on abscess on the tip of the finger and bound with moist bandage to treat infection. *Root*: Mixed with roots from other medicinal plants to make medicines to treat edema. Roasted, made into a powder and taken with warm water to treat inflammation of the liver, inflammation of the spleen and general edema. Used in making medicines to prevent miscarriage, and to treat lumps on the throat, passing and hemorrhaging of blood, vitiligo, and cataracts. Juice from the male root is taken with cold milk to treat chronic coughing. *Bark*, *Root*: Used as purgative and diuretic. *Flowers*: Crushed together with milk and the juice used to paint circles around the eyes to treat sore eyes associated with infantile diseases. *Fruit*: Juice from the green fruit can be tipped into the nostrils to cure headaches that affect only one side of the head. *Seeds*: Used to treat inflammation of the testes, and hiccups.

###### Notes.

In India the leaf is used on swellings, the seed as a laxative, the root for goiter and leprosy, and an unspecified part for snakebite (Jain and DeFilipps 1991). Perry (1980) discusses the uses of the species in Indo-China, the Malay Peninsula, and Indonesia. She notes that medicinal use of the species is primarily in Java and India.

Perry (1980) lists the chemical constituents of the species.

###### References.

Nordal (1963), Agricultural Corporation (1980).

#### 16. *Cullen* Medik.

##### Cullen corylifolium

Taxon classificationPlantaeORDOFAMILIA

(L.) Medik. (= Psoralea corylifolia L.)

###### Names.

**Myanmar**: *babchi*, *nehle*. **English**: prairie turnip, scuffy pea.

###### Range.

Pakistan, India, Sri Lanka, Myanmar, China, Arabia, Somomali Republic, Socotra. In Myanmar, found in Magway and Mandalay.

###### Uses.

*Fruit*, *Seeds*, *Root*: Used as diuretic, antiasthmatic, and laxative.

###### Notes.

In India the leaf is used for diarrhea; the seed as an anthelmintic, diuretic, deobstruent; for stomach problems, skin diseases, leucoderma, leprosy, scorpion sting, and snakebite (Jain and DeFilipps 1991). In China the fruit is used as an aphrodisiac and tonic to the genital organs. The seed is used as an aphrodisiac, stimulant, and tonic in arthritis, dysmenorrhea, enuresis, fever, impotence, leprosy, leucoderma, leucorrhea, lumbago, polyuria, premature ejaculation, spermatorrhea, and splenits. It is used externally for callosities, vitiligo, and other skin ailments such as leucoderma, leprosy, and psoriasis. The root is used for caries (Duke and Ayensu 1985). Medicinal uses of the seeds in China, Indo-China, and the Malay Peninsula are discussed in Perry (1980). She notes that, from the literature, it appears the seeds of this species are an ancient Hindu medicine.

In India, oleorsin extract is used locally on leprosy (Jain and DeFilipps 1991). According to one study, a 30% alcohol extract of the seeds applied to spots of leucoderma showed “enough improvement to justify further study”. Others have observed that the essential oil has a powerful effect against cutaneous streptococci. The seeds contents are reported to include fixed oil, essential oil, oleoresin, psoralen, isopsoralen, and psoralidin (Perry 1980).

###### Reference.

Nordal (1963).

#### 17. *Cynometra* L.

##### Cynometra ramiflora

Taxon classificationPlantaeORDOFAMILIA

L.

###### Names.

**Myanmar**: *myinga*, *ye-minga*. **English**: cynometra.

###### Range.

India, Indo-China, and Malesia. In Myanmar, found in Ayeyarwady, Rakhine, and Taninthayi.

###### Uses.

*Leaf*: Used as an antiherpetic. *Root*: Employed as a purgative.

###### Notes.

Perry (1980) discusses the medicinal uses of this species in East and Southeast Asia. Duke (2009) notes use the species for dermatosis, scabies, and leprosy. In India the leaf is boiled in cow’s milk and mixed with honey into a lotion, then applied externally for skin diseases, scabies, and leprosy; oil from the seed is applied externally for the same afflictions; and the root is used as a purgative and cathartic (Jain and DeFilipps 1991).

###### Reference.

Perry (1980).

#### 18. *Delonix* Raf.

##### Delonix regia

Taxon classificationPlantaeORDOFAMILIA

(Hook.) Raf.

###### Names.

**Myanmar**: *jaw-gale*, *seinban*. **English**: flamboyant, gold mohur, royal poinciana.

###### Range.

Seasonally dry areas of western and northern Madagascar. Cultivated in Myanmar.

###### Conservation status.

Least Concern [LC] (IUCN 2017).

###### Use.

Nordal (1963) lists this species as having medicinal properties, but the plant parts and uses are unspecified.

###### Notes.

The bark of this species is employed as a febrifuge in Indo-China. The gum which oozes from it “is similar to gum arabic” (Perry 1980).

The leaves contain saponin and alkaloid (Perry 1980).

Data on the propagation, seed treatment, and agricultural management of this species are given by Katende et al. (1995) and Bekele-Tesemma (1993).

###### Reference.

Nordal (1963).

#### 19. *Entada* Adans.

##### Entada phaseoloides

Taxon classificationPlantaeORDOFAMILIA

(L.) Merr.

###### Names.

**Myanmar**: *do*, *gon-nyin*. **English**: sword bean.

###### Range.

Pantropical. Reported from Myanmar.

###### Uses.

*Seed*: Used as an emetic and febrifuge; also as a fish poison.

###### Notes.

In China the plant is considered anti-cancer; also used for splenititis with high temperature and as a wash for itch, pityriasis, and wounds. The seed is used to treat hemorrhoids in children (Duke and Ayensu 1985). In India the juice from the bark and wood is applied externally for ulcers and the stem is used as an emetic; the seeds are used as an anthelmintic, tonic, antiperiodic, and emetic; a paste made from them is locally applied to inflamed glandular swellings (Jain and DeFilipps 1991). Medicinal uses of the species in additional East and Southeast Asian countries follow: In Mongolia the plant is used to treat illnesses with a high temperature in the spleen; on the Malay Peninsula ashes of pods are applied to the abdomen for severe internal complaints; in Indonesia the pounded roots are rubbed on, and the juice from the stem is drunk to treat a feverish abdomen and dysentery, roasted seeds are eaten by women as a depurative in post partum and are administered in small doses for stomachache, as an emetic, and are a component in some compound medicines; and in the Philippines a decoction of the roots is drunk to treat a rigid abdomen and smashed seeds are used to poultice abdominal complaints, such as colic of children (Perry 1980).

The seeds contain oil with palmitic-, stearic-, lignoceric-, linoleic-, and oleic acid, raffinoe, traces of alkaloid, and steroids; the seed, stem, and bark contain saponin A and B; and the stem and root bark contain HCN. Also, the seed has entagenic acid, a saponin active against a type of carcinosarcoma in rats (Duke and Ayensu 1985). “Much of the medicinal use of the species is due to the presence of saponin in the bark, wood, and seeds.” Seeds are edible after proper preparation: “They must be roasted until the seedcoat bursts, washed in water for 24 hours, and boiled before eating.” Reported chemical constituents include saponins and a heteroside, also a poisonous alkaloid. “Two saponins, nearly alike in chemical and pharmacological properties, have a strong hemolytic action on human red blood cells; stem, seeds, and bark are poisonous” (Perry 1980).

###### References.

Nordal (1963), Perry (1980).

#### 20. *Erythrina* L.

##### Erythrina variegata

Taxon classificationPlantaeORDOFAMILIA

L. (= E. indica Lam.)

###### Names.

**Myanmar**: *kathit*, *in-kathit*. **English**: Indian coral tree.

###### Range.

Tanzania to India, Asia, Australia and the Pacific Islands (var. orientalis).

###### Conservation status.

Least Concern [LC] (IUCN 2017).

###### Uses.

*Bark*: Used as an antipyretic and, in a decoction, to treat liver problems. *Bark*, *Leaf*, *Root*: Used to treat dysentery and inflammation.

###### Notes.

In India the bark is used for convulsion and for paralysis of the tongue (given with roots of two other plants); also for pimples, cough and cold, and snakebite (Jain and DeFilipps 1991). In China the leaf is used as an anthelmintic, antisyphilitic, diuretic, emmenagogue, lactagogue, and laxative; leaf juice for earache, toothache, and worms. Stem-bark is employed as an analgesic for arthritis, neuralgia, and rheunmatism; also as a febrifuge, cholagogue, expectorant, ophthalmic, hepatic, and vermifuge (Duke and Ayensu 1985). Perry (1980) notes that the bark and leaves are the parts most often used. She discusses the uses of the species in Indo-China, Indonesia, the Philippines, New Guinea, and the Solomon Islands.

Chemical constituents include hydrocyanic acid in the stems, leaves, fruit, and roots; and two alkaloids, erythraline and hypaphorine, in the seeds. Resins, fixed oils, fatty acids, hypaphorine, betaine, choline, potassium chloride, and potassium carbonate are present in the bark (Perry 1980). The poisonous alkaloid fraction shows anti-convulsive activity, inhibits neuromuscular activity, weakens the smooth muscles, and paralyzes the central nervous system; HCN occurs in most parts of the plant. The bark is bacteriostatic against *Staphylococcus aureus* (Duke and Ayensu 1985).

###### References.

Nordal (1963), Perry (1980).

#### 21. *Flemingia* Roxb. ex W.T. Aiton

##### Flemingia chappar

Taxon classificationPlantaeORDOFAMILIA

Buch.-Ham. ex Benth.

###### Names.

**Myanmar**: *bahon*, *gyo-pan*, *kyabahon*, *se-laik-pya*.

###### Range.

Cambodia, China, India, Laos, Myanmar, Nepal, and Thailand.Widely distributed in Myanmar.

###### Uses.

*Root*: Used as a sedative and analgesic.

###### Notes.

This species has been studied for its anti-cancer and antiviral activities (Rastogi and Dhawan 1990). Rao (1990) has reviewed root flavonoids, including those of this species, as a source of pharmaceuticals. Adityachaudhury and Gupta (1973) have found a new pterocarpan and coumestan in the roots of *F. chappar*. They briefly discuss the antimicrobial activies and biosynthetic pathways of these compounds.

###### Reference.

Nordal (1963).

##### Flemingia strobilifera

Taxon classificationPlantaeORDOFAMILIA

(L.) W.T. Aiton (= Moghania strobilifera (L.) J. St.-Hill.)

###### Names.

**Myanmar**: *se-laik-pya*, *thingu-gyat*. **English**: wildhops.

###### Range.

India to the Philippines. In Myanmar, found in Ayeyarwady and Yangon.

###### Use.

*Root*: Used to treat epilepsy.

###### Note.

On the Malay Peninsula and in the Philippines, a decoction of the root of this species is administered as a post partum protective medicine, and the leaves are employed at the same time to wash the body; also used in a lotion to treat rheumatism., Additionally, in the Philippines a decoction or infusion of the leves and flowers is prescribed for tuberculosis (Perry 1980).

###### Reference.

Perry (1980).

#### 22. *Glycine* Willd.

##### Glycine max

Taxon classificationPlantaeORDOFAMILIA

(L.) Merr. (= G. hispida (Moench) Maxim.; G. soja Sieb. & Zucc.)

###### Names.

**Myanmar**: *ber-hrum*, *hsan-to-nouk*, *ngasee*, *pe-bok*, *pe-ngapi*. **English**: soja bean, soy bean, soya bean.

###### Range.

Southeast Asia. Now widely cultivated in the Orient and elsewhere. Cultivated in Myanmar.

###### Uses.

*Seed*: used as a tonic and carminative.

###### Notes.

The seeds are regarded as a tonic, diuretic, febrifuge, and antidote. Also, the seeds in combination with other drugs are used to treat a large number of ailments. “It was observed many years ago that natives in the Orient ate infested meat products without ill effects, if soy sauce was a part of the meal” (Perry 1980).

The species is said to assist the flow of digestive juices, increase the assimilation of high protein foods, and to be a source of riboflavin, thiamin, niacin, panthotheic acid, and choline. An antibiotic, canavalin, has been found in the plant, which is useful in treating certain pneumococci. Results of research by the Soya Corporation of America have lead to the production of an “edible antibiotic that counteracts various types of harmful bacteria through implantation of beneficial intestinal flora”. Raw soybeans contain a *toxic principle* with hemolytic activity which is destroyed by heat (Perry 1980).

###### Reference.

Nordal (1963).

#### 23. *Indigofera* L.

##### Indigofera cassioides

Taxon classificationPlantaeORDOFAMILIA

DC. (= I. pulchella Roxb.)

###### Names.

**Myanmar**: *kan-tin*, *mawk-kham*, *taw-mevaing*. **Hawaiian**: *sakina*. **English**: kathu.

###### Range.

Pakistan, India, Myanmar, China, Siam, and Indochina. In Myanmar, found in Bago, Chin, Mandalay, and Shan.

###### Use.

*Roots*: Used for coughs.

###### Notes.

In India the powdered root of this species is externally applied for chest pain; a decoction of the root is used for coughs. Medicinal uses for several other species belonging to this genus are also discussed (Jain and DeFilipps 1991). Medicinal uses of the species in China are discussed in Duke and Ayensu (1985); and medicinal uses in South China, China, Taiwan, Indonesia, and the Philippines are discussed in Perry (1980).

###### Reference.

Perry (1980).

#### 24. *Lablab* Adans.

##### Lablab purpureus

Taxon classificationPlantaeORDOFAMILIA

(L.) Sweet (= Dolichos lablab L.)

###### Names.

**Myanmar**: *nwai-pe*. **English**: Bonavista bean, Egyptian bean, hyacinth bean, Indian bean, lablab bean, lubia bean.

###### Range.

Probably Old World; now widespread.

###### Uses.

*Seed*: Used as a febrifuge, stomachic, and antispasmodic.

###### Notes.

In India the seed is used for a febrifuge, an antispasmodic, a stomachic, and an aphrodisiac (Jain and DeFilipps 1991). In China the whole plant is decocted for use in alcoholic intoxication, cholera, diarrhea, globefish poisoning, gonorrhea, leucorrhea, nausea, and thirst. The stem is used for cholera. The flower is used for leucorrhea, menorrhagia, and dysentery; as an antivinous, alexiteric, and carminative; and for “summer heat disorders”. Fruit juice is employed for inflamed ears and throats. The “white seeds” are taken with vinegar for cholera morbus; also as an anthelmintic, astringent, digestive, and stomachic. It is further noted that the seeds are reportedly alexiteric, antispasmodic, aphrodisiac, febrifuge, stomachic, and used for menopause (Duke and Ayensu 1985). Perry (1980) discusses the species medicinal uses in China, the Malay Peninsula, and Indonesia.

###### Reference.

Nordal (1963).

#### 25. *Leucaena* Benth.

##### Leucaena leucocephala

Taxon classificationPlantaeORDOFAMILIA

(Lam.) de Wit (= L. glauca Benth.)

###### Names.

**Myanmar**: *aseik-pye*, *aweya*, *bawzagaing*, *baw-sagaing*. **English**: lamtoro, leucaena, wild tamarind.

###### Range.

Tropical America, Asia. Found in Upper Myanmar, in Mandalay, Sagaing, and Yangon.

###### Uses.

*Whole plant*: The five parts (root, stem, leaf, flower and fruit) are used to make antidotes for poisons. A mixture of the crushed five parts, or the roots with butter, is used as an ointment applied topically to aching areas around a snakebite to neutralize the venom. *Bark*: Taken to treat internal aches and pains. *Leaf*: The heating properties are known to stimulate the blood, as well as control gas and neutralize poison; also made into a paste and applied to poisonous bites and stings. The tender leaves and pods (without the seeds) are boiled and eaten with fish paste or fish sauce as dip to regulate bowels and cure aches related to male disorders. *Seed*: Used in medicines for aches, pains, and edema. *Root* and *Bark*: Decoction used in preparations to prevent miscarriages.

###### Notes.

The medicinal uses of this species in India are discussed in Jain and DeFilipps (1991). Medicinal uses of the species in Indonesia and the Philippines are discussed in Perry (1980).

###### References.

Agricultural Corporation (1980), Perry (1980).

#### 26. *Millettia* Wight & Arn.

##### Millettia pachycarpa

Taxon classificationPlantaeORDOFAMILIA

Benth.

###### Names.

**Myanmar**: *mi-gyaung-nwe*, *nhtau-ru*, *semein*, *hon.* **English**: fish poison climber, millettia.

###### Range.

China; Bangladesh, Bhutan, India, Nepal; Myanmar, Thailand. In Myanmar, found in Kachin, Mandalay, and Taninthayi.

###### Use.

*Root*: Used as fish poison.

###### Notes.

In China the whole plant is used as a tonic and to induce the growth of red blood cells (Duke and Ayensu 1985). Medicinal uses of the species in East and Southeast Asia include as an antianemic, a tonic, and to induce growth of red blood cells. It is also employed as an insecticide and to stun fish (Perry 1980).

*Millettia pachycarpa* contains the antitumor compound rotenone (Duke and Ayensu 1985).

###### Reference.

Nordal (1963).

#### 27. *Mimosa* L.

##### Mimosa pudica

Taxon classificationPlantaeORDOFAMILIA

L.

###### Names.

**Myanmar**: *hi-ga-yone*, *tikayon*, *kaya* (Kachin), *hta-muck* (Mon), *nam ya-hai-awn* (Shan). **English**: mimosa, sensitive plant, shame weed, touch-me-not.

###### Range.

Pantropical, originating in the Neotropics (thought probably native to South America). Grows naturally all over Myanmar.

###### Conservation status.

Least Concern [LC] (IUCN 2017).

###### Uses.

*Whole plant*: Bitter and astringent in taste with cooling properties, the five parts (root, stem, leaf, flower and fruit) are known to “calm” (reduce) phlegm and bile. A mixture of the crushed plant and water is applied topically to reduce edema. The liquid extracted from the whole plant is applied to treat inflamed sores; also used to make tonics and medicines to treat vomiting of blood, hemorrhaging, and asthma. The whole plant is also employed as a diuretic and antiseptic. *Leaf*: Crushed and applied as a poultice over the pubic region to treat excessive urination. A mixture of the powdered leaves and milk is taken for hemorrhoids. *Root*: Paste is applied topically to heal sores. A root decoction is given to dissolve gall stones and to promote urinary function.

###### Notes.

The medicinal uses of this species in India are discussed in Jain and DeFilipps (1991). Indigenous medicinal uses of this species in the Andaman and Nicobar Islands (India) are described by Dagar and Singh (1999).

Seeds of *M. pudica* contain L-Djenkolic acid which if consumed in sufficient quantities can lead to acute kidney malfunction, and also contain L-Mimosine which may impart goitrogenic effects (Lan et al. 1998).

###### References.

Nordal (1963), Agricultural Corporation (1980), Forest Department (1999).

#### 28. *Mucuna* Adans.

##### Mucuna pruriens

Taxon classificationPlantaeORDOFAMILIA

(L.) DC. (= M. prurita (L.) Hook.)

###### Names.

**Myanmar**: *gwin-nge*, *hko-mak-awa*, *khwele*, *khwe-ya*, *khwe-laya*, *to-ma-awn*, *pwekonclaw* (Mon), *ra*, *yan-nung* (Chin), *hko-ma-awn* (Shan). **English**: common cowitch, cowhage, cowitch, velvet bean.

###### Range.

Himalyas, India, Sri Lanka, Southeast Asia, and Malaysia. In Myanmar, found in Bago, Chin, Kayin, Mandalay, Sagaing, Shan, and Yangon.

###### Uses.

Known for a bitter-sweet taste, cooling properties, and control of flatulence and gall bladder. *Leaf*: Boiled, eaten with fish paste or fish sauce as a dip, is used as a remedy for male maladies; it is also given to mothers to increase lactation, prevent vomiting, and stop bleeding. *Fruit*: Used as a de-worming medicine; also pulverized and mixed with water, then ingested as a remedy for urination problems. *Seed*: Used in a tonic. The seeds and seed cases are used in preparations to increase sperm, stimulate lactation, improve circulation, promote vitality and weight gain, expel intestinal worms, and strengthen the senses. Seed cases are rubbed on affected areas to alleviate numbness. Stir-fried or otherwise cooked young seeds are eaten to stop vomiting and bleeding. Fried in butter, they are given to promote strength and weight gain. Crushed seeds are used to make a poultice applied to scorpion and centipede bites. They are also used in medicines to increase strength and vitality, to cure venereal diseases and paralysis, and to stimulate formation of new tissue in the healing of sores and wounds. A mixture of powdered seeds and milk is used to increase sperm and stimulate lactation, and one of equal amounts of the pulverized seeds, root, and sugar is taken for health and vitality; it is also considered extremely beneficial for the vitality of semen. *Root*: Serves as an emmenagogue, tonic, aphrodisiac, and purgative. Boiled in water and reduced to one-third the starting volume, given with honey for cholera. With diuretic properties, they are used in preparations to strengthen the blood vessels. Root powder mixed with water is taken for dysentery. To treat edema in the abdominal area, crushed root is rubbed onto the stomach; to reduce edema in the joints of fingers and toes, it is formed into pieces and tied to the affected areas; the juice can be taken daily to cure paralysis and atrophied arms. Filtered oil from cooking root powder is rubbed onto affected areas to alleviate enlargement and hardening from elephantiasis.

###### Notes.

In India the root is used as a tonic, diuretic, purgative; for nervous and renal diseases, dropsy; and for elephantiasis. The hairs on the pods are employed for stomach worms; the seed is used for impotency, urinary calculus, tonic, and as an aphrodisiac (Jain and DeFilipps 1991). In Pakistan the root is also employed to remedy nervous disorders, and delirium (Neptune-Rouzier 1997). In China, Guam, Indonesia, the Philippines, the Malay Peninsula, and Indonesia the uses of this species are noted as being similar to those of the other species in the genus (Perry 1980).

The chemical constituents, pharmacological activities, and traditional medicinal uses of this plant on a worldwide basis are discussed in detail by Ross (1999), who notes that the chemical compound mucunaine, found in this species, is an irritant causing pruritus. The chemistry, pharmacology, toxicology, and use of this species as a hunting poison and medicinal plant in Africa are discussed by Neuwinger (1994). Details of the active chemical compounds, effects, herbal usage and pharmacological literature of this plant are given in Fleming (2000).

###### References.

Nordal (1963), Agricultural Corporation (1980), Forest Department (1999).

#### 29. *Phyllodium* Desv.

##### Phyllodium pulchellum

Taxon classificationPlantaeORDOFAMILIA

(L.) Desv. (= Desmodium pulchellum (L.) Benth.)

###### Names.

**Myanmar**: *bahon*, *pan-letwa*, *se-leik-pya*, *tabyetse*, *taung-damin*. **English**: tick clover, tick trefoil.

###### Range.

China, Japan, Taiwan; India, Nepal, Sri Lanka; Indo-China; Malesia; Australia. Widely distributed in Myanmar.

###### Conservation status.

Least Concern [LC] (IUCN 2017).

###### Uses.

*Bark*: Used as an astringent and in eye diseases.

###### Notes.

In China the root is used for burning sensation in the abdomen (Duke and Ayensu 1985). In South China the plant is used to for rheumatic fever, convulsion in infants, and to treat rheumatism, toothache, dissolve blood clots, “build new red blood cells”, and aid digestion; on the Malay Peninsula, a decoction of the roots is used as a post partum protective medicine; and in Indonesia and the Philippines, the leaves are applied to pocks and ulcers (Perry 1980).

###### Reference.

Nordal (1963).

#### 30. *Pithecellobium* Mart.

##### Pithecellobium dulce

Taxon classificationPlantaeORDOFAMILIA

(Roxb.) Benth.

###### Names.

**Myanmar**: *kala-magyi*. **English**: manila tamarind, guaymochil.

###### Range.

Mexico to northwestern South America. Introduced and cultivated in India and Pakistan. Introduced into Myanmar.

###### Uses.

*Leaf*: Used as an abortive and as a digestive.

###### Note.

In India the bark is used in a decoction as an enema (Jain and DeFilipps 1991).

###### Reference.

Nordal (1963).

#### 31. *Saraca* L.

##### Saraca indica

Taxon classificationPlantaeORDOFAMILIA

L.

###### Names.

**Myanmar**: *thawka*, *thawka-po*. **English**: asoka tree, sorrowless tree.

###### Range.

India, Pakistan, Sri Lanka, Myanmar, Malaya. Cultivated in Myanmar.

###### Uses.

*Bark*: Used as anthelmintic and astringent. It is also used to treat menorrhagia.

###### Notes.

Medicinal use of the species in East and Southeast Asia are discussed in Perry (1980).

###### References.

Nordal (1963), Perry (1980).

#### 32. *Senna* Mill.

##### Senna alata

Taxon classificationPlantaeORDOFAMILIA

(L.) Roxb. (= Cassia alata L.)

###### Names.

**Myanmar**: *beeda khutdai*, *sok* (Mon), *hpak-lam-mon-long* (Shan), *mezali-gyi*, *pwesay-mezali*, *thinbaw-mezali*. **English**: candle bush, empress candle plant, ringworm cassia, ringworm shrub.

###### Range.

Tropical America; now pantropical. Widely distributed in Myanmar.

###### Use.

*Leaf*: Powder can be mixed with honey and licked to promote weight gain and increase strength and vitality. Skin disorders such as scabies, ringworm and eczema can be cured by rubbing them with the leaves twice a day over a period of time. Crushed and applied as a poultice over the bite to poisonous or venomous animals to neutralize the poison. Crushed and squeezed juice of leaves applied to visible symptoms of venereal disease. Boiled down to make a strong potion which when kept in the mouth while warm cures gum boils and inflammation of the gums. Mixed with *mu-yar-gyi* (*Adhatoda vasica* = *Justicia adhatoda*) leaves, chewed and kept in the mouth or the juice swallowed to cure dry coughs. Crushed with lime juice and applied to cure eczema. Pounded, mixed with the juice of *neem* (*Azadirachta indica*) leaves, and applied to cure ringworm and leprosy. Drinking the liquid obtained from boiling the buds and the leaves will cure inflammation of the breathing passages and asthma, cause loose bowels, encourage urination and discharge of mucus in the stool). *Flower*: Crushed fine and applied as a rub to cure skin diseases. *Seed*: Astringent, can cure itching, coughs, asthma, ringworm, skin disorders, kills disease causing germs, promote good urination and cure leprosy. *Root*: Made into a paste, mixed with boric acid powder and *hpan-kar* (*Terminalia chebula*) fruit powder and applied to cure ringworm.

###### Notes.

Medicinal uses of this species in India are discussed in Jain and DeFilipps (1991) as follows: The whole plant is an anti-inflammatory (excluding the root); the twig is used on eczema sores; the leaf is used for ringworm (leaf-juice with lime juice), also as an insecticide, abortifacient, anthelmintic, taenifuge, snakebite, and diuretic (decoction); decoctions with flowers and leaves are used for bronchitis, asthma, and (in a wash) for eczema; the seed is used as a vermifuge; the root is used as a purgative and for rheumatism; an unspecified part is used for snakebite, ascariasis, ringworm, and leprosy. Medicinal uses of this species in China are discussed in Duke and Ayensu (1985). Here the stem wood is used for hepatitis, loss of appetite, urticaria, and rhinitis; the leaf is used much as it is in India, also poulticed onto boils and ulcers; the flower is purgative; and the seed is taken internally for skin ailments. The plant is considered anti-cancer. Perry (1980) gives its medicinal uses from India east to Indo-China, south through southeastern Asia to Guam and Palau.

The medicinal uses of this plant in the Caribbean region, as well as its chemistry, biological activity, toxicity and dosages, are discussed by Germosén-Robineau (1997). The chemical constituents, pharmacological activities, and traditional medicinal uses of this plant on a worldwide basis are discussed in detail by Ross (1999).

The plant contains chrysarobin, and chrysarophanic acid; rhein in the leaf; and oxymethyl anthraquinone in the fruit; sometimes with HCN (Duke and Ayensu 1985).

###### References.

Nordal (1963), Agricultural Corporation (1980), Forest Department (1999).

##### Senna alexandrina

Taxon classificationPlantaeORDOFAMILIA

Mill. (= Cassia acutifolia Delile; Cassia angustifolia M. Vahl)

###### Names.

**Myanmar**: *pwe-gaing*, *thinbaw-mezali*. **English**: Alexandrian senna, Arabian senna, Indian senna, tinnevelly senna.

###### Range.

Egypt, Sudan to Nigeria. Cultivated in India and Myanmar.

###### Use.

*Leaf*: Used in treating dull stomach pain, liver disease, dropsy, bile, indigestion, leprosy, coughing with phlegm, and aches and pains in the joints. Taking the leaves with the liquid from boiling dried ginger root will cure indigestion. If the leaves are taken with the juice from *zee-hpyu* fruit (*Phyllanthus emblica*), it will cure leprosy and edema. One tablespoon of the liquid in which it has been boiled rather strongly can be mixed into a cup of milk and taken in order use as a laxative.

###### Notes.

The leaflets of this species contain cassic acid or “hein,” an antibiotic substance effective against *Staphylococcus aureus* (Perry 1980).

###### References.

Nordal (1963), Agricultural Corporation (1980).

##### Senna auriculata

Taxon classificationPlantaeORDOFAMILIA

(L.) Roxb. (= Cassia auriculata L.)

###### Names.

**Myanmar**: *peik-thingat*. **English**: avaram, mataran tea, Tanner’s cassia, Tanner’s tea.

###### Range.

Pakistan Madhya Pradesh and Western Peninsula, India, Myanmar, and Sri Lanka. Cultivated in Myanmar.

###### Uses.

*Bark*: Used as an astringent. *Leaf* and *Pod*: Sometimes an adulterant of senna. *Seed*: Used as a refrigerant.

###### Notes.

In India a decoction of the whole plant is used for diabetes and diuresis; the bark is astringent in skin diseases, also used for sore throat (gargle); the leaf and fruit are anthelmintic; a decoction of the flower buds, or whole plant, is used for diabetes and diuresis; the seed is used for ophthalmia, diabetes and chylous urine, as well as for conjunctivitis (finely powdered decorticated seeds as dusting powder); and the root is astringent (Jain and DeFilipps 1991).

###### References.

Perry (1980), Forest Department (1999).

##### Senna italica

Taxon classificationPlantaeORDOFAMILIA

Mill. (= Cassia obtusa Roxb.)

###### Names.

**Myanmar**: *dangywe*, *kathaw-pok*, *nawnam*, *shan-kazaw*. **English**: golden cassia.

###### Range.

Native to Chile. Widespread in Myanmar.

###### Use.

*Leaf*: Used as a laxative.

###### Note.

This species is used in East and Southeast Asian countries as a laxative (Perry 1980).

###### Reference.

Nordal (1963).

##### Senna siamea

Taxon classificationPlantaeORDOFAMILIA

(Lam.) H.S. Irwin & Barneby (= Cassia siamea Lam.)

###### Names.

**Myanmar**: *mai-mye-sili*, *mejari*, *mezali*, *taw-mezali*. **English**: kassod tree, Siamese cassia.

###### Range.

Southeast Asia and East Indies. Widely distributed in Myanmar.

###### Uses.

*Leaf*, *Flower*, *Fruit*: Made into a soup which is drunk as a tonic and to relieve stomach pains.

###### Notes.

In Indonesia a decoction of the young leaves is used to treat malaria. In a number of Asian countries, stem wood is an ingredient in recipes used to make a decoction to treat liver trouble, urticaria (nettle rash), loss of appetite from gastrointestinal trouble, and rhinitis (Perry 1980).

Chemical research has revealed the presence of a poisonous alkaloid (Perry 1980).

###### References.

Perry (1980), Forest Department (1999).

##### Senna sulfurea

Taxon classificationPlantaeORDOFAMILIA

(Collad.) H.S. Irwin & Barneby (= Cassia glauca Lam.)

###### Names.

**Myanmar**: *pyiban-nyo*, *pyidban-shwe*, *yong* (Mon). **English**: smooth senna.

###### Range.

Cultivated in Myanmar.

###### Uses.

*Leaf*: Bitter and astringent in taste with cooling properties, promotes urination and cures gonorrhea. If the liquid obtained from squeezing the leaves is taken with milk and sugar, it will cure pain in passing urine and gonorrhea. Eating a salad made from the boiled leaves with dried prawns will cure many gas problems such as flatulence and shooting pains, as well as fevers, diabetes, and gonorrhea. Taking the powder made from the leaves will cure gas problems, illnesses due to heat, and pain in passing urine. Consuming a clear soup with the leaves added can cure passing mucus with the stool, dysentery, illnesses caused by gas, indigestion and degeneration of bile, and will also give strength.

###### Reference.

Agricultural Corporation (1980).

##### Senna tora

Taxon classificationPlantaeORDOFAMILIA

(L.) Roxb. (= Cassia tora L.)

###### Names.

**Myanmar**: *dangywe*, *dant-kywei*, *dinghkri*, *myay-pe-naw-nam*, *ngusat*. **English**: metal seed, sicklepod.

###### Range.

West Indies, Central and South America, and parts of North America. In Myanmar, found in Kachin, Mandalay, Sagaing, and Yangon.

###### Uses.

*Leaf*: Used as a laxative and vermifuge.

###### Notes.

In India the leaf is used for skin diseases, as a laxative (decoction), on cuts, for eczema (paste) and bone fracture (leaves pounded with egg albumen, and applied as plaster), as a vermicide (infusion), and for indigestion (powder); also, young leaves are eaten to prevent skin disease; the seed is used for skin diseases, ringworm, and for eczema (Jain and DeFilipps 1991). In China old leaves are used for ringworm; the fruit is used for dysentery, opthalmia, several eye ailments (cataracts, conjunctivitis, glaucoma), headache, hepatitis, herpes, furnunculoid sores, and arthritis. The seeds are used for boils, and as an external and internal medicine for eye diseases (Duke and Ayensu 1985).

The species contains aloe-emodin (antitumor), aurantio obtusin, chrysophanol, emodin, obtusin, physcion, rhein, rubrofusarin, torachryon, toralactone. Also, due to unnamed glycosides, aqueous and ethanol seed extracts possess hypotensive and bradycardiac actions (Duke and Ayensu 1985).

###### References.

Nordal (1963), Perry (1980).

#### 33. *Sesbania* Scop.

##### Sesbania grandiflora

Taxon classificationPlantaeORDOFAMILIA

(L.) Pers.

###### Names.

**Myanmar**: *pauk-pan-byu*. **English**: scarlet wisteria tree, vegetable humming-bird, West Indian pea tree.

###### Range.

Tropical Asia; naturalized in southern Florida and the West Indies; and widely cultivated in the tropics. Cultivated in Myanmar.

###### Uses.

*Bark*: Used for anemia. *Leaf*: Used in medicines to treat stomach bloating, tumors, fevers, sores, diabetes, skin irregularities caused by blood problems, and throat ailments, as well as to protect against colds, leprosy, spleen inflammation, and germs. They are also used in remedies to neutralize venom from scorpion stings; and eaten to ease constipation, clear the mind, alleviate shooting pains, neutralize poisons, and prevent lung and heart disease. Preparations containing the leaves are taken to cleanse the blood. The juice from crushed leaves, mixed in equal amounts with dried ginger, *peik-chin* (*Piper longum*), and cane sugar, is inhaled to ease restlessness. For fever or influenza, the stir-fried leaves and onions are eaten. A mixture of the liquid from the leaves and the seed kernels from *kyee-ni thee* (*Barringtonia acutangula)* is eaten as a cure for impotency; a mixture of the crushed leaves and cow urine is inhaled as a cure for epileptic seizure. *Leaf* and *Flower*: For headaches on one side of the head, the juice from crushed flowers and leaves is inhaled through the nostril on the affected side. *Flower*: Boiled and given orally for night blindness. The juice from the crushed flowers is used as an eye drop solution for dim vision and watery eyes. Remedies made from the flowers are given to reduce fever. *Root*: For joint inflammation, a warmed root paste is applied topically.

###### Notes.

Uses of this species in India, Indo-China, the Malay Peninsula, Indonesia, and the Philippines are discussed in Perry (1980).

###### References.

Nordal (1963), Agricultural Corporation (1980).

##### Sesbania sesban

Taxon classificationPlantaeORDOFAMILIA

(L.) Merr. (= S. aegyptiaca (Poir.) Pers.)

###### Names.

**Myanmar**: *ye-tha-gyi*. **English**: common sesban, Egyptian rattlepod.

###### Range.

Old World tropics; tropical Asia. In Myanmar found in Sagaing.

###### Uses.

*Bark*: Used for skin conditions, liquid from the crushed bark is given orally, and the seed paste is applied topically. It is also used to clear infections, promote new tissue formation, and heal chronic sores. *Leaf*: Used in maturative poultices. Leaf also used to treat poisoning, edema, and eye infections; to purify breast milk, open blocked mammary glands, and increase lactation. New mothers eat the leaves in a variety of forms, including in clear soups, boiled lightly, in salad, fried as fritters, or pickled. Juice from the crushed leaves is used as an eye drop solution to clear infection and to reduce fever. For swollen joints, aches, and pains, the liquid from boiled leaves is taken orally. Powder from the dried leaves is taken with honey or in sweet liqueurs as a tonic for strength and vitality. *Seed*: Component of remedies for irregular menstrual periods, liver inflammation, and lung infections. *Root*: Used in medicines to treat stomach bloating, tumors, fevers, sores, diabetes, skin irregularities caused by blood problems, and throat ailments, as well as to protect against colds, leprosy, spleen inflammation, and germs. They are also used in remedies to neutralize venom from scorpion stings.

###### Notes.

In India the leaf is used in a poultice for suppuration of boils and rheumatic swelling. The seed is employed as a stimulant and astringent emmenagogue; also for diarrhea, spleen enlargement, and in ointments for skin eruptions (Jain and DeFilipps 1991).

Extracts from the flower of this species show antifertility activity (Jain and DeFilipps 1991).

###### References.

Agricultural Corporation (1980), Perry (1980).

#### 34. *Spatholobus* Hassk.

##### Spatholobus parviflorus

Taxon classificationPlantaeORDOFAMILIA

(DC.) Kuntze (= S. roxburghii Benth.)

###### Names.

**Myanmar**: *da-ma-nge*, *labanru*, *nwe-ni*, *pauk-nwe*, *rubanru*. **Hawaiian**: *maula*, *maulu*.

###### Range.

Asia: China; Indian subcontinent, including Bhutan, Bangladesh, India, Nepal, and Sri Lanka; Indo-China, including Cambodia, Laos, Myanmar, Vietnam, and Thailand. In Myanmar, found in Bago, Magway, Mandalay, Taninthayi, and Yangon.

###### Conservation status.

Least Concern [LC] (IUCN 2017).

###### Use.

*Leaf*: Used for medicinal purposes (exact uses not given in Perry 1980).

###### Notes.

In Indonesia two other members of the genus are used medicinally: 1. *S. ferrugineus* is drunk to treat colic; and, after childbirth, a decoction of the pounded stem, leaves, or the sap is ingested as a remedy for faulty menstruation and uterine hemorrhage. 2. An infusion of the sap of *S. littoralis* is drunk, and the feet are washed with it as a remedy for difficulty in moving the legs (Perry 1980).

###### Reference.

Perry (1980).

#### 35. *Tadehagi* H.Ohashi

##### Tadehagi triquetrum

Taxon classificationPlantaeORDOFAMILIA

(L.) H.Ohashi (= Desmodium triquetrum (L.) DC.)

###### Names.

**Myanmar**: *lauk-thay*, *moko-lanma*, *shwe-gu-than-hlet*, *thagya-hlandin*. **English**: begar’s-tick, tick clover, tick trefoil.

###### Range.

Asia- Bhutan, China, Hong Kong, India, Indonesia, Laos, Myanmar, Peninsular Malaysia, the Philippines, Ryukyu Island, Sri Lanka, and Taiwan; Australasia; Indian Ocean Islands; Pacific Ocean Islands. In Myanmar, found in Chin, Kachin, Kayin, Mandalay, Sagaing, Shan, and Yangon.

###### Use.

*Root*: The liquid from stewing the root with a bit of pepper can cure blood in the urine. *Leaf*: Eating leaves can cure dysentery, bloated stomach, stomachache in children due to worms, and feeling of fullness and indigestion. Taken as a tea, the leaves can cure urinary and skin disorders. The leaves of the plant and the leaves of the *dawai-hmaing* (*Combretum indicum*) can be lightly boiled in water to cure urinary disorders, dysentery, bleeding hemorrhoids, and hemorrhaging during menstruation. The dried leaves of the plant and the dried leaves of *hpalan-taung-mwei* (*Cheilocostus speciosus*) can be mixed in equal amounts, made into a powder, dissolved in coconut oil, and kept in the sun; the clear top oil can then be used as ear drops to cure ear infections with pus and earaches; if used as an ointment, the oil can cure scabies, impetigo, erysipelas, open sores and seborrhoeic dermatitus of the scalp. If the leaves are mixed with dried flowers of *saga-sein* (*Cananga odorata*), steeped in sesamum oil and the oil used as hair oil, it will cure headaches, fever, dandruff, itching of the scalp, and head lice. *Plant*: Used to kill worms.

###### Notes.

In India the leaf is used for cough, cold, and abdominal pain; the root for snakebite (Jain and DeFilipps 1991). In China the plant is applied to abscesses; used as a tonic for dyspepsia, hemorrhoids, and infantile spasms; and also employed as an insecticide and vermicide (Duke and Ayensu 1985). In South China the species is used as a medicine for infantile spasms, a tonic for dyspepsia, an application against abscesses, a remedy for hemorrhoids, and as a vermicide and insecticide; in Indonesia, an infusion of the dried and powdered leaves is taken or sometimes the powder is made into pills, the leaves are used externally to treat lumbago and internally (with the pods) as a diuretic in treating gravel (Perry 1980).

The leaves have been found to contain tannin, silicic acid, and potassium oxide (Perry 1980).

###### References.

Agricultural Corporation (1980), Perry (1980), Forest Department (1999).

#### 36. *Tamarindus* L.

##### Tamarindus indica

Taxon classificationPlantaeORDOFAMILIA

L.

###### Names.

**Myanmar**: *beng-kong*, *magyeng*, *ma-gyi*, *mai-kyaing*, *mak-k yeng*, *manglon*. **English**: tamarind.

###### Range.

Origin unknown, possibly tropical Asia or Africa. Cultivated in Myanmar.

###### Use.

*Root*: Used in treating gonorrhea, urinary diseases, hemorrhoids, jaundice, and shooting or dull pains in the stomach. *Bark*: The entire bark can be made into an ash and taken with water after meals to cure vomiting and gastic problems. The bark ash can be mixed with honey to cure shooting or dull stomach pains. Indigestion can be cured if the outer bark is baked until burnt, made into a powder, and taken with warm water. Applying a paste made from the bark with water will cure sore eyes, sores, and bites of venomous creatures. *Leaf*: The juice from the leaves can be cooked with sesame oil and a small amount applied into the ear to cure earaches. Taking one tablespoon of the juice squeezed from the crushed leaves to cure urinary disorders. The juice squeezed from crushed leaves can be applied to heat rashes. One part of the juice squeezed from the leaves can be mixed with two parts of rock salt to neutralize snake venom. The leaves can be eaten with *kalain* (*Caesalpinia crista*) seeds to cure excessive perspiration and body odor. *Fruit*: The pulp of the fruit is used in making up laxatives and tonics. Equal amounts of old tamarind fruit, garlic that has been soaked in yogurt liquid, and *chay-thee* (*Semecarpus anacardium*) is to be mixed and ground up, made into pellets and dried in the shade; taking one pellet together with one teaspoon of garlic juice every 15 minutes will cure cholera. *Seed*: Soaked in water overnight, outer skin discarded, kernel crushed and taken with milk to cure white vaginal discharge and excessive urination. A seed kernel paste can be taken to cure diarrhea and dysentery, and can be applied to a scorpion bite to neutralize the venom. The skin of a mature seed can be mixed with cumin and rock sugar, made into a powder and taken to cure dysentery.

###### Notes.

Medicinal uses of this species in India are discussed in Jain and DeFilipps (1991). Medicinal uses of the species in China are discussed by Duke and Ayensu (1985).

Pharmacognostic characters and Thai ethnomedical use of this species are discussed in Somanabandhu et al. (1986). Chemical constituents, pharmacological action, and medicinal use of this species in Indian Ayurveda are discussed in detail by Kapoor (1990). The chemical constituents, pharmacological activities, and traditional medicinal uses of this plant on a worldwide basis are discussed in detail by Ross (1999). A pharmacognostical profile including medicinal uses of this plant in Africa is given in Iwu (1993). Data on the propagation, seed treatment and agricultural management of this species are given by Katende et al. (1995) and Bekele-Tesemma (1993). Details of the active chemical compounds, effects, herbal usage and pharmacological literature of this plant are given in Fleming (2000). The fruit yields some potassium tartrate, gelatin, citric acid, malic acid and glucides. All parts of the *T. indica* plant contain cyanogenic glycosides which cause diarrhea and vomiting when ingested in large quantities (Lan et al. 1998).

###### References.

Nordal (1963), Agricultural Corporation (1980), Forest Department (1999).

#### 37. *Tephrosia* Pers.

##### Tephrosia purpurea

Taxon classificationPlantaeORDOFAMILIA

(L.) Pers.

###### Names.

**Myanmar**: *me-yaing*. **English**: bastard indigo, wild indigo.

###### Range.

Southern Asia, Australia, tropical Africa, south to Natal; introduced in tropical America. In Myanmar, found in Bago, Magway, Mandalay, Sagaing, and Yangon.

###### Uses.

*Whole plant*: Used as an anthelmintic and antipyretic.

###### Notes.

In India the whole plant is used as a tonic for impotency and gonorrhea; a decoction, employed as a vermifuge, is made from the fruit. Oil obtained from the seeds is used for scabies, itch, eczema, and other skin diseases. The root is used for dyspepsia, diarrhea, rheumatism, fever, snakebite, asthma, urinary disorders, colic; also as a liniment on elephantiasis. An unspecified plant part is used as a tonic, laxative, and diuretic; also for bronchitis, febrile effects, bleeding piles, boils, and pimples (Jain and DeFilipps 1991).

###### Reference.

Nordal (1963).

#### 38. *Xylia* Benth.

##### Xylia xylocarpa

Taxon classificationPlantaeORDOFAMILIA

(Roxb.) Taub. (= X. dolabriformis Benth.)

###### Names.

**Myanmar**: *hpat*, *mai-salan*, *pkhay*, *praing*, *pran*, *prway*, *pyin*, *pyinkado*. **English**: Burmese ironwood, irul.

###### Range.

Native to Bangladesh, Cambodia, eastern India, Laos, Myanmar, Thailand, and Vietnam. Introduced into Africa, Philippines, Singapore. Widely distributed in Myanmar.

###### Uses.

*Bark*: Used as an astringent. *Seed*: Oil used to treat rheumatism.

###### Notes.

In India the bark is used to treat gonorrhea, diarrhea, stop vomiting, and as a vermifuge (Jain and DeFilipps 1991).

###### Reference.

Nordal (1963).

### Gentianaceae (Gentian family)

#### 1. *Exacum* L.

##### Exacum tetragonum

Taxon classificationPlantaeORDOFAMILIA

Roxb.

###### Names.

**Myanmar**: *pa-deing-ngo*. **English**: bicolor Persian violet.

###### Range.

India and China south to New Guinea. In Myanmar, found in Bago, Chin, Kachin, Taninthayi, and Yangon.

###### Use.

*Whole plant*: Used in a tonic for fever.

###### Note.

In India the whole plant is used as a tonic for fevers and as a stomachic (Jain and DeFilipps 1991).

###### Reference.

Perry (1980).

#### 2. *Swertia* L.

##### Swertia chirayita

Taxon classificationPlantaeORDOFAMILIA

(Roxb.) Buch.-Ham. ex C.B.Clarke

###### Names.

**English**: bitter stick, clearing nut tree, Indian gentian.

###### Range.

Eastern Asia - Himalayas.

###### Uses.

A bitter. Plant [part(s) not given] used as an aperient and as a tonic. Dried plant imported to Indo-China and Malaya where it is used as a febrifuge. Used with success in a majority of fevers, especially typhoid.

###### Notes.

In India the whole plant is used as a bitter, stomachic, anthelmintic, febrifuge, as well as for malarial fever, asthma, and liver disorders. Also taken with sandalwood in a paste to heal internal hemorrhage of stomach. A decoction of the root (with root of *Acorus calamus*) is used as a remedy for intermittent fever, leprosy, leucoderma, scabies and other skin diseases. An unspecified plant part is used for gravel in urine, atrophy, bronchitis, consumption, gonorrhea, bleeding gums, emaciation, puerperal fever, and also cooling, and curing thirst, biliousness, and inflammation (Jain and DeFilipps 1991).

Reported constituents include chiratin, chiratogenin, ophelic acid, resin, and tannin (Perry 1980).

###### Reference.

Perry (1980).

### Hydroleaceae (Waterleaf family)

#### 1. *Hydrolea* L.

##### Hydrolea zeylanica

Taxon classificationPlantaeORDOFAMILIA

(L.) Vahl

###### Name.

**English**: Ceylon hydrolea.

###### Range.

Tropical America, Africa, and southeastern Asia. In Myanmar, found in Bago and Yangon.

###### Conservation status.

Least Concern [LC] (IUCN 2017).

###### Use.

*Leaf*: Beaten to a pulp to make a dressing for foul ulcers (thought to have antiseptic and cleansing properties).

###### Notes.

In Cambodia, India, Sri Lanka, and elsewhere, leaves are used for intestinal disorders; macerated leaves are applied as poultice to callous difficult ulcers for soothing and healing properties; also said to possess some antiseptic properties (Kham 2004).

###### Reference.

Perry (1980).

### Hypericaceae (Hypericum family)

#### 1. *Cratoxylum* Blume

##### Cratoxylum formosum

Taxon classificationPlantaeORDOFAMILIA

(Jacq.) Benth. & Hook.f. ex Dyer (= C. prunifolium Dyer)

###### Names.

**Myanmar**: *bamachet*, *ma-chyangai*, *mye-mu-se*, *sa-thange-ohnauk*. **Thai**: *tiu khon tree*.

###### Range.

Tropical Asia. Widely distributed in Myanmar.

###### Conservation status.

Lower Risk/least concern [LC] (IUCN 2017).

###### Use.

*Bark*, *Leaf*, *Root*: Given as a protective remedy to a women after childbirth.

###### Notes.

Several species in the genus *Cratoxylum* appear to have some medicinal use. In Indo-China, the species *C. pruniflorum* is thought to have “marked digestive properties”, and, in combination with with *Artemisia* leaves, is administered to women in parturition (Perry 1980).

###### Reference.

Nordal (1963).

### Lamiaceae (Mint family)

#### 1. *Callicarpa* L.

##### Callicarpa macrophylla

Taxon classificationPlantaeORDOFAMILIA

Vahl

###### Names.

**Myanmar**: *daung-satpya*, *kyun-nalin*, *lahkylk*, *mai-hpa*, *mai-put*, *makpa nakeching*, *pebok*, *sigyi*, *tawngto-nao*. **English**: beautyberry.

###### Range.

China, Bhutan, India, Myanmar, Nepal, Sri Lanka. Thailand, and Vietnam. In Myanmar, found in Chin, Kachin, Kayin, and Sagaing.

###### Use.

*Bark*: Provides a medication for skin disease. *Root*: Used as a stomachic.

###### Note.

On the Malay Peninsula the pounded leaves are used to poultice sores and a decoction is drunk to relieve stomachache; in China this species is used by herbalists to treat influenza in infants (Perry 1980).

###### References.

Nordal (1963), Perry (1980).

#### 2. *Clerodendrum* L.

##### Clerodendrum indicum

Taxon classificationPlantaeORDOFAMILIA

(L.) Kuntze (= C. siphonanthus R.Br.)

###### Names.

**Myanmar**: *ngayant patu*, *nygayan-padu*. **English**: tubeflower.

###### Range.

Temperate and tropical Asia; grows naturally all over Myanmar; especially reported from Kachin and Magway.

###### Uses.

*Resin*: Used to treat syphilitic rheumatism. *Leaf*: Remedies made from the leaves are used for fevers and respiratory problems, including coughs; they are also used to improve menstrual flow and cleanse residual menstrual discharge. Boiled leaves made into salads are eaten to promote regularity. The leaves are also used to make de-worming medicines. *Leaf* and *Root*: Used in preparations to stimulate circulation, as well as to treat leprosy and female disorders; also for asthma and fever. *Seed*: Preparations are used to treat joint inflammation related to sexually transmitted diseases. *Root*: The paste mixed with ginger powder is ingested for lung infections. The root is also used as a component in medicines for male disorders, gonorrhea, asthma, bronchitis, aches, and pains.

###### Notes.

The medicinal uses of this species in India are discussed in Jain and DeFilipps (1991). Medicinal uses of this species in China are discussed in Duke and Ayensu (1985).

###### References.

Nordal (1963), Agricultural Corporation (1980), Perry (1980), Forest Department (1999).

##### Clerodendrum infortunatum

Taxon classificationPlantaeORDOFAMILIA

L.

###### Names.

**English**: hill glory bower.

###### Range.

South and southeastern Asia. Widely distributed in Myanmar.

###### Uses.

*Leaf* and *Root*: Used as a febrifuge.

###### Notes.

In India the leaf is used for headache; also ground with leaves of *Commelina bengalensis* and applied as a plaster for sores on head. The flower (ground with fresh shoots of *Bombax ceiba*, made into pills, and these smeared with cream from cow milk) is used for ulcers of the palate. The root is used for rheumatism; ground with black pepper and used for involuntary cramps; and ground with leaves, roots, bulb, and bark of various other species, and given to drink with refuse of molasses for gravel (Jain and DeFilipps 1991). In Indo-China this species is used in a decoction as a remedy for leucorrhea (Perry 1980).

Reported constituents of the leaves of this species include clerodin (anthemintic property); glycerides of linolenic, oleic, stearic, and lignoceric acids; a sterol; a proteinase; and a peptidase (Perry 1980).

###### Reference.

Perry (1980).

##### Clerodendrum thomsoniae

Taxon classificationPlantaeORDOFAMILIA

Balf.f.

###### Names.

**Myanmar**: *tike-pan*, *taik-pan-gyi*. **English**: bag-flower, bleeding-heart vine, glory tree, tropical bleeding heart.

###### Range.

West and West-Central tropical Africa. Cultivated in Myanmar.

###### Uses.

Plant used for medicinal purposes (exact uses not given in Nordal 1963).

###### Notes.

Other members of the genus are reported as used medicinally in India, China, Thailand, Korea, and Japan for the treatment of such diseases as syphilis, typhoid, cancer, jaundice, and hypertension (Shrivastava and Patel 2007).

Major chemical compounds have been reported from this genus. These include phenolics, steroids, di- and tri-terpenes, flavonoids, volatile oils, etc. (Shrivastava and Patel 2007).

###### Reference.

Nordal (1963).

#### 3. *Colebrookea* Sm.

##### Colebrookea oppositifolia

Taxon classificationPlantaeORDOFAMILIA

Sm.

###### Names.

**Myanmar**: *chying-htawng-la*. **English**: Indian squirrel tail, opposite-leaf drysophylia.

###### Range.

China, India, Myanmar, Nepal, and Thailand. In Myanmar, found in Chin and Kachin.

###### Uses.

*Root*: Used to treat epilepsy and as an antiseptic.

###### Note.

In India the stem is used for cough; the leaf to treat wounds and eye problems (Jain and DeFilipps 1991).

###### Reference.

Nordal (1963).

#### 4. *Gmelina* L.

##### Gmelina arborea

Taxon classificationPlantaeORDOFAMILIA

Roxb.

###### Names.

**Myanmar**: *mai-saw*, *thebla*, *thun-vong*, *yemane*. **English**: gmelina, gumhar, Malay bush-beech.

###### Range.

From India to southeastern Asia.

###### Uses.

*Leaf*: The juice is used as a treatment for ulcers. *Root*: Used as a stomachic.

###### Notes.

In India the bark is used for cholera, swelling and choking in the throat (with garlic), rheumatism, epilepsy, dropsy, and anasarca, convulsion (with bark of *Bauhinia purpurea*), syphilis (with shoots, leaves and roots from a combination of species), bronchitis (with many plants), intoxication or stupor, bites of poisonous insects and other animals (with bark of two other plants), and diarrhea; the leaf is a carminative; and the root is used as a tonic, laxative, and for rheumatism (Jain and DeFilipps 1991). Medicinal uses of the species in Indo-China are discussed in Perry (1980).

###### Reference.

Perry (1980).

#### 5. *Leucas* R.Br.

##### Leucas cephalotes

Taxon classificationPlantaeORDOFAMILIA

(Roth) Spreng.

###### Names.

**Myanmar**: *pin-gu-hteik-peik*. **English**: gumma.

###### Range.

Eastern Asia: Himalayas from Afghanistan to western China. In Myanmar, found in Ayeyarwady, Bago, Chin, Kayah, Mandalay, Sagaing, Shan, Taninthayi, and Yangon.

###### Uses.

*Whole plant*: Used to treat bronchitis, asthma, dyspepsia, and jaundice. Headaches can be cured by brushing the forehead with the liquid from crushing all plant parts with a bit of pepper. The liquid can also be mixed with honey to cure coughs in children. The liquid from the plant boiled with one or two cloves will bring down fever. For jaundice and inability to produce semen, the plant can be utilized in several ways such as being boiled and taken; the liquid from crushing the plant taken; the root made into a paste or crushed and taken; the leaves, flowers and fruits eaten with a fish sauce dip, in a salad, or cooked. *Leaf*: Liquid from crushed leaves taken orally or poured into the nose will neutralize snake bite venom and cause its effects to wane. A little bit of the liquid from crushing the leaves mixed with *peik-chin* (*Piper longum*) fruit powder can be taken to cure inflammation of joints, tendons and ligaments. Use juice from crushed leaves as an ointment to cure itching.

###### Notes.

In India the whole plant is used as a diaphoretic and stimulant; the juice for scabies. The leaf is used to treat dysentery and diarrhea; the flower for cough syrup and fever. A twig with flowers and seed is pounded in mustard oil and 2–3 drops are put in the ear to stop pus formation (Jain and DeFilipps 1991).

###### References.

Nordal (1963), Agricultural Corporation (1980).

#### 6. *Mentha* L.

##### Mentha arvensis

Taxon classificationPlantaeORDOFAMILIA

L.

###### Names.

**Myanmar**: *payoke-aye*, *pusi-nan*, *budi-nan*. **English**: corn mint, field mint, japanese mint, wild mint.

###### Range.

Europe and Asia. Cultivated throughout Myanmar, but thrives most in temperate climates.

###### Conservation status.

Least Concern [LC] (IUCN 2017).

###### Uses.

Sharp and efficacious in taste with fragrant smell. *Whole plant*: Five parts of the plant are used to control phlegm, help menstrual blood to descend, strengthen the kidneys, treat asthma, for liver and spleen diseases, and for inflammation of the joints. When the whole plant is dried, prevents thirst and fevers, aids digestion and promotes urination. The plant is used in making medicines to treat gas disorders, distended and bloated stomach, fevers, and muscle twitches. It cal also be boiled and taken to cure stomachaches. *Leaf*: Liquid obtained from leaves can be mixed with honey and licked to cure loose bowels. They can be boiled and taken to cure inflammation and aching joints, sore throat, and coughing. Boiled with dried ginger, they are used to treat colds. Crushed young leaves are used as an inhaler and to treat a dazed dizzy feeling, and also to clear the brain. Liquid from the leaf is rubbed on like an ointment to relieve aching eyes. Liquid from distilling them can be given to cure stomachaches in children and to treat hypertension. They can be chewed and pressed onto a cat’s bite to disinfect it. Adding leaves to an anti-nausea medicine will speed its action. The solid obtained from their oil is used as an additive in toothpaste and soap in order to augment their properties.

###### Notes.

The medical uses of this species in India are discussed in Jain and DeFilipps (1991). Medicinal uses of this species in China are discussed in Duke and Ayensu (1985).

###### References.

Agricultural Corporation (1980), Forest Department (1999).

#### 7. *Ocimum* L.

##### Ocimum americanum

Taxon classificationPlantaeORDOFAMILIA

L. (= O. canum Sims)

###### Names.

**Myanmar**: *pin-sein*, *pin-sein hmway*. **English**: hoary basil.

###### Range.

Tropical and subtropical. Asia, tropical Africa. Found naturally all over country, especially in the hot zone. Grows up to 915 m altitude. Cultivated.

###### Uses.

Can control gas and phlegm, congestion, and indigestion; can degrade bile. Plant also used as a diuretic. *Whole plant*: Used to treat skin diseases and as a febrifuge. Soaked in water and the steam inhaled to treat paralysis due to strokes and inflammation of the joints. Monkey meat can be roasted, and together with many basil leaves, used to treat lung disease, impotency, eye diseases, coughing, and asthma. *Leaf*: The juice obtained from crushing them used for coughs, skin disease, loss of appetite, and stomach pain due to gastritis. Leaves crushed and squeezed until liquid comes out and this brushed onto the temples and forehead to cure headaches. They can be stir fried with dried *ngagyi chaul* (*Heteropneustes fossilis*, a small freshwater catfish) to treat vomiting, fatigue in women, a prolapsed uterus, blockage of milk glands, itching of the body and limbs, pain in passing urine, and infections occurring after childbirth. To neutralize very venomous snake and other venomous bites, equal amounts of the leaves and *pyin-daw* (*Clausena* sp.), and basil leaves are crushed together and made into balls taken as pills, also crushed leaves are made into a poultice to place on the bites. Slightly smoked basil and betel (*Piper betle*) leaves crushed together with some tumeric powder are used as an ointment to treat children with hot foreheads. *Seed*: Equal parts of basil, sesame seeds, and jaggery are ground together and mixed with honey, made into balls the size of betel nuts, then swallowed twice a day to give relief from and cure diseases that occur in the intestine, heart, and kidney, as well as diseases producing excess gas and phlegm, toothaches, inflammation of the gums, hemorrhoids, too little urine, and skin diseases such as ringworm, scabies, and eczema. *Seed*: Dried, slightly crushed seeds, taken together with milk and sugar are used to treat urinary diseases and menstruation with coagulated blood. The seeds can be soaked in water and added to soft drinks to treat hepatitis, promote urination, and ease fatigue.

###### Note.

The medicinal uses of this species in India are discussed in Jain and DeFilipps (1991).

###### References.

Nordal (1963), Agricultural Corporation (1980), Perry (1980).

##### Ocimum tenuiflorum

Taxon classificationPlantaeORDOFAMILIA

L. (= O. sanctum L.)

###### Names.

**Myanmar**: *kala-pi-sein*, *pin-sein-net*. **English**: holy basil, sacred basil.

###### Range.

Old World tropics. Cultivated in Myanmar.

###### Uses.

*Leaf*: Used as an expectorant and stomachic; also, in a decoction, as a mild febrifuge and carminative for infant diarrhea. *Seed*: Used to treat kidney diseases. *Root*: Employed as a diaphoretic.

###### Notes.

The medicinal uses of this species in India are discussed in Jain and DeFilipps (1991) as follows: The leaf is used as a stimulant, antiperiodic, diaphoretic, expectorant; also for fever, hemiplegic, constipation, liver disorders, cough (with black pepper and rice), diarrhea, and colds; the oil for antibacterial and insecticidal purposes. An infusion is used for digestive problems. Also used locally for ringworm and earache. The seed is used as a demulcent, laxative, and for urinary problems. The root is used for sudden collapse and in a decoction for malaria as a diaphoretic. Medicinal uses of the species in Indo-China, the Malay Peninsula, Indonesia, and the Philippines are discussed in Perry (1980).

Reported constituents of the volatile oil of *O. tenuiflorum* include methyl chavicol, cineole, linalool, methyl homo-antisic acid, caryophyllene, eugenol, eugenol methyl ether, and carvacrol. The mucilage contains hexuronic acid, pentoses, and ash; also, after hydolysis, xylose (Perry 1980).

###### References.

Nordal (1963), Perry (1980).

#### 8. *Orthosiphon* Benth.

##### Orthosiphon aristatus

Taxon classificationPlantaeORDOFAMILIA

(Blume) Miq. (= O. stamineus Benth.)

###### Names.

**Myanmar**: *hsee-cho*, *thagyar makike*, *si-cho*. **English**: cat’s whiskers, Java tea, kidney tea plant.

###### Range.

Temperate and tropical Asia, Australia. Found cultivated throughout Myanmar.

###### Uses.

This plant is most well-known as a diuretic and as a medicine for diabetes.

*Leaf*: Prepared as a herbal tea to alleviate kidney disorders, bladder diseases, and urinary problems as well as to treat aching joints.

###### Notes.

In India the leaf is used as a diuretic, for nephrosis, and for edema; also used in an infusion for kidney and bladder diseases and rheumatism (Jain and DeFilipps 1991). The medicinal uses of the species from Taiwan south to Palau, in the Philippines, and on the Malay Penisula are discussed in Perry (1980).

Reported chemical constituents include a glucoside and orthosiphon. The leaves contain volatile and essential oils; both the leaves and stems have a high potassium content, urea, and ureids (Perry 1980). An extract of the leaf has been found to lower blood sugar (Jain and DeFilipps 1991).

###### References.

Nordal (1963), Agricultural Corporation (1980).

#### 9. *Pogostemon* Desf.

##### Pogostemon cablin

Taxon classificationPlantaeORDOFAMILIA

(Blanco) Benth. (= P. patchouli Pellet.)

###### Names.

**Myanmar**: *thanat-pyit-see*. **English**: patchouli.

###### Range.

Native of southeastern Asia. Cultivated in Myanmar.

###### Uses.

*Leaf*: Used to treat kidney and bladder diseases. Used in making diuretics and medicines to cure shooting pains in the stomach. Juice taken with small amount of marijuana leaves when there is blood in the urine. Juice taken to relieve pain during menstruation.

###### Notes.

In India an infusion of the leaf is used for menstrual troubles (Jain and DeFilipps 1991). In China the whole plant is used for abdominal pain, cold, diarrhea, halitosis, headache, and nausea (Duke and Ayensu 1985). Medicinal uses of the species in China, on the Malay Peninsula, and in the Philippines are discussed in Perry (1980).

The species has been used in China for 100 years. The branches and leaves of *P. cablin* (introduced into China) are used as drug which is considered superior to the commercial drug consisting of dried aerial parts of *Agastache rugosa* (cultivated in China). The drug is considered carminative, stomachic, antivinous, antiemetic, and depurtive. It is useful in treating influenza and colds, headache, indigestion, fever, cholera, and the nausea of pregnancy (Perry 1980).

The whole plant is antiseptic and the oil is bactericidal (Duke and Ayensu 1985). The chemical constituents of its volatile oil include patchouli alcohol, cadinene, coerulein, benzaldehyde, and eugenal (Perry 1980).

###### References.

Nordal (1963), Agricultural Corporation (1980).

#### 10. *Premna* L.

##### Premna amplectens

Taxon classificationPlantaeORDOFAMILIA

Wall. ex Schauer

###### Names.

**Myanmar**: *sagale-amauk*, *yinbya-byu*, *wee-ek*, *hpak-si-so*. **English**: surfacea, tatea.

###### Range.

Pakistan and Sri Lanka to Myanmar. Now also in other Southeast Asian countries. Reported from Myanmar.

###### Uses.

*Root*: Used as a decoction after childbirth.

###### Notes.

Most members of this genus are employed in the treatment of fever; also headache, stomachache, and toothache. Other frequent uses are as a diuretic and laxative, for cold and cough, and also for boils (Duke 2009).

###### Reference.

Nordal (1963).

##### Premna mollissima

Taxon classificationPlantaeORDOFAMILIA

Roth (= P. latifolia Roxb.)

###### Names.

**Myanmar**: *kyetyo*, *kyun-nalin*, *seiknan-gyi*. **English**: black plum.

###### Range.

China, Cambodia, India, Indonesia, Laos, Myanmar, Philippines, and Vietnam. Widely distributed in Myanmar.

###### Use.

*Root*: A paste of the root is used for a local application after parturition.

###### Notes.

In India the stem-bark is used for ringworm and blisters in the mouth; the leaf as a diuretic and for dropsy; and the root for syphilis and gonorrhea (Jain and DeFilipps 1991). Medicinal uses of this species in China, Indo-China, Indonesia, the Philippines, New Guinea, and the Solmon Islands are discussed in Perry (1980).

The bark of the trunk contains two alkaloids, premnine and ganiarin. Premnine has bee found to lessen the force of heart contraction and dilate the pupils of the eyes (Perry 1980).

###### Reference.

Perry (1980).

##### Premna serratifolia

Taxon classificationPlantaeORDOFAMILIA

L. (= P. integrifolia L.)

###### Names.

**Myanmar**: *kywe-thwe*, *taung-tangyi*.

###### Range.

Himalaya (Nepal to Bhutan), India. In Myanmar, found in Mandalay, Rakhine, Taninthayi.

###### Uses.

*Whole Plant*: Decoction used to treat fever, neuralgia, and rheumatism. *Root* and *Stem Bark*: Used as laxative, carminative, stomachic. *Root*: Used to treat diabetes and liver complaints.

###### Note.

In India the leaf is used as a carminative, galactagogue, and in a decoction for flatulence and colic; the root is used as a laxative, stomachic, tonic, and is a component of the Ayurvedic drug dasmula used for fever (Jain and DeFilipps 1991).

###### References.

Nordal (1963), Forest Department (1999).

#### 11. *Rotheca* Raf.

##### Rotheca incisa

Taxon classificationPlantaeORDOFAMILIA

(Klotzsch) Steane & Mabb. (= Clerodendrum macrosiphon Hook f.)

###### Names.

**Myanmar**: *ngayan-padu*. **English**: tubeflower.

###### Range.

Tropical Africa. Cultivated in Myanmar.

###### Uses.

*Leaf*: Used in treating venereal diseases.

###### Notes.

In Africa, leaf-sap and a root-decoction are drunk as an anti-malarial (Burkill 1985).

###### Reference.

Nordal (1963).

##### Rotheca serrata

Taxon classificationPlantaeORDOFAMILIA

(L.) Steane & Mabb. (= Clerodendrum serratum (L.) Moon)

###### Names.

**Myanmar**: *bebya*, *begyo*, *yinbya*, *yinbya-net*, *prang-gadawn* (Kachin). **English**: blue fountain bush.

###### Range.

South and southeastern Asia, and eastern Africa. Found growing naturally throughout the country, but especially in Upper Myanmar.

###### Uses.

*Leaf*: Boiled lightly in water, the leaves are eaten in salads to relieve female-related disorders. New mothers eat the boiled-leaf salads to support healing, increase strength, and promote lactation. *Leaf* and *Root*: Used in preparations for fever, asthma, coughs, colds, and infected sores. They are also used to stimulate the appetite, improve digestion, and expel uterine leiomyomas. *Root*: For fevers and colds, they are crushed and brewed with water; used in a decoction after childbirth. Oil from cooking the roots is filtered and applied around the eyes to treat inflammation, itching, and infections. A mixture of- the roots with equal amounts of dried ginger and coriander seeds is boiled to half the starting volume and the reduction is ingested in the mornings and evenings to relieve bloating and nausea; one part powdered roots with 12 parts yogurt is boiled to half the starting volume and taken in small amounts in the mornings and evenings to alleviate edema; equal amounts of the powdered roots and powdered, dried ginger is taken with fresh ginger juice for colds, asthma, whooping cough, and bronchitis. To treat internal inflammations, such as those caused by diphtheria, and cysts arising from other conditions, a paste made from the powdered roots and rice washing water is applied externally at frequent intervals. Note: The powdered roots must be consumed only in very small amounts ranging from ~1.0 g to ~3.0 g.

###### Notes.

Medicinal uses of this species in India are discussed in Jain and DeFilipps (1991). The plant’s medicinal uses in Indo-China, Indonesia, and the Malay Peninsula are discussed in Perry (1980).

###### References.

Nordal (1963), Agricultural Corporation (1980), Perry (1980), Forest Department (1999).

#### 12. *Salvia* L.

##### Salvia officinalis

Taxon classificationPlantaeORDOFAMILIA

L.

###### Names.

**English**: common sage, garden sage, kitchen sage, sage.

###### Range.

Northern and central Spain to West Balkan Peninsula and Asia Minor. Cultivated in Myanmar.

###### Conservation status.

Least Concern [LC] (IUCN 2017).

###### Uses.

Species used as a topical antiseptic and orally as a carminative and spasmolytic. *Leaf*: Used as a diaphoretic and stomachic.

###### Notes.

The species is astringent, a stimulant, and is put into a gargle for sore throat (Perry 1980). In India the species is used for thrush and gingivitis; an infusion is used as a gargle and diaphoretic (Jain and DeFilipps 1991).

The leaf and tops of young shoots yield an oil, which is carminative (Jain and DeFilipps 1991).

###### Reference.

Nordal (1963).

#### 13. *Tectona* L.f.

##### Tectona grandis

Taxon classificationPlantaeORDOFAMILIA

L.f.

###### Names.

**Myanmar**: *kyun*, *kyun-pin*, *mai-sak* (Kachin), *pahi* (Kayin), *klor* (Chin), *mai-sa-lan* (Shan). **English**: teak.

###### Range.

Asia: India and Myanmar to Java, occasional on other islands. Species grows naturally throughout Myanmar below 915 m altitude.

###### Uses.

*Bark*: Used as an astringent. Water from soaking the bark overnight is given for white vaginal discharge. Liquid from soaking bark powder in warm water is ingested for chronic diarrhea. A paste made from ground bark is applied topically to relieve bloating and edema related to gall bladder problems. A second paste, made from ground bark powder mixed with cashew nut oil, is also applied topically to relieve inflammation. A third paste, made from the ground bark, ground charcoal, and rice cooking water, is applied repeatedly to treat herpes. *Bark*, *Wood*, *Fruit*: Components of medicines used to reduce phlegm, cure gonorrhea, treat leprosy, alleviate bloating, and stop hemorrhaging. *Wood*: Pul-verized and used on swellings. *Fruit*: A paste, made by grinding the fruit with cooking oil, is used to alleviate itching and rashes. A second paste, made by grinding the fruit with rice washing water, is applied topically to clear clogged milk glands. Finely crushed fruit is cooked, applied as a poultice over the navel, and bound there with a cloth to treat urinary problems. Oil of fruit is used as a remedy for skin diseases. *Root*: Used to treat urinary discharges.

###### Notes.

The medicinal uses of this species in India are discussed in Jain and DeFilipps (1991). Medicinal uses of this species in Indo-China, the Malay Peninsula, Indonesia, and the Philippines (where introduced) are discussed in Perry (1980).

###### References.

Nordal (1963), Agricultural Corporation (1980), Perry (1980).

#### 14. *Vitex* L.

##### Vitex glabrata

Taxon classificationPlantaeORDOFAMILIA

R.Br.

###### Names.

**Myanmar**: *mak-lok-kaing*, *panameikli*, *tauksha*, *thauk-kya*. **English**: blackberry tree, smooth chastetree.

###### Range.

Bangladesh, India; Laos, Myanmar, Thailand, Vietnam; Indonesia, Malaysia, Singapore; Australia; cultivated and naturalized elsewhere. Reported from Myanmar.

###### Use.

*Bark* and *Root*: Used as an astringent.

###### Note.

In India the bark and root are used as an astringent (Jain and DeFilipps 1991).

###### Reference.

Perry (1980).

##### Vitex negundo

Taxon classificationPlantaeORDOFAMILIA

L.

###### Names.

**Myanmar**: *kyaungban-gyi*. **English**: five-leaved chaste tree, Indian privit.

###### Range.

Southeastern Africa, Madagascar, eastern and southeastern Asia, Philippine Islands, Guam; naturalized in Florida.

###### Use.

*Fruit*: Used as a sedative.

###### Notes.

In China the stem-twigs are decocted for burns and scalds, and a twig infusion is used for anxiety, convulsions, cough, headache, and vertigo; the leaf is astringent, sedative, used for cholera, eczema, and gravel; the fruit for angina, cold, cough, deafness, gonorrhea, hernia, leucorrhea, and rheumatic difficulties; the root for colds and rheumatic ailments. The plant is also said to prevent malaria, and is used for bacterial dysentery and chronic bronchitis (Duke and Ayensu 1985). The medicinal uses of the species in China, Indo-China, Indonesia, the Philippines, and Palau are discussed in Perry (1980).

The leaves are bactericidal and insecticidal, and yield essential oil with aldehydes and ketones, phenolic derivatives, and cineol (Duke and Ayensu 1985).

###### Reference.

Nordal (1963).

##### Vitex trifolia

Taxon classificationPlantaeORDOFAMILIA

L.

###### Names.

**Myanmar**: *kyaung-pan*. **English**: Indian wild pepper.

###### Range.

Asia to Australia. Found growing in warmer parts of Myanmar, up to 915 m altitude.

###### Uses.

*Leaf*: Used to treat skin infections, disorders of the spleen, and rheumatism. Also used in preparations to regulate menstruation and bowel function, stimulate healing of sores, control fevers, neutralize poisons, and promote vitality. The crushed leaf juice and stir-fried leaves are used to treat varicose veins and other circulatory conditions. The leaf juice is applied topically to heal chronic sores; mixed with a bit of sesame oil and honey, and swabbed inside the ear to alleviate earaches and to clear ear infection; taken by itself for skin conditions and together with the juice from ground roots of *thet-yin-gyi* (*Croton persimilis*) for bloating and edema. Water from boiling the leaves is ingested for weakness and weight loss, malaria, menstrual problems, and conditions related to birthing, as well as for coughs and colds in infants and young children. A salad of the leaves mixed with garlic is eaten to relieve bloating, indigestion, and dysentery. Pillows stuffed with the dried leaves are used for insomnia and brain conditions. *Leaf* and *Flower*: Used as febrifuge and emetic. *Root*: Ground, and a paste made from them is given to children for ingesting or inhaling to reduce fever and treat cooking fume-related sickness.

###### Notes.

The medicinal uses of the species in India are discussed in Jain and DeFilipps (1991). Medicinal uses of the species in China are discussed in Duke and Ayensu (1985). Perry (1980) covers the medicinal uses of the species in the Malay Peninsula, Korea, China, and Indo-China, and Mongolia.

The essential oil of this species yields camphene, and pinene, terpenylacetate; the leaves contain aucubin, agunuside, casticin, orientin, isoorientin, and luteolin-7-glucoside; and the fruit contains vitricine. Leaf extracts have been found to inhibit the tuberculosis organism and also show anti-cancer activity (Duke and Ayensu 1985).

###### References.

Nordal (1963), Agricultural Corporation (1980), Perry (1980), Forest Department (1999).

#### 15. *Volkameria* L.

##### Volkameria inermis

Taxon classificationPlantaeORDOFAMILIA

L. (= Clerodendrum inerme (L.) Gaertn.)

###### Names.

**Myanmar**: *kywe-yan-nge*, *pinle-kyauk-pan*. **English**: garden quinine, glory bower.

###### Range.

Seacoast. South and southeastern Asia, Australia, and Pacific Islands. Cultivated in Myanmar.

###### Uses.

*Leaf* and *Root*: Used in fumigation after childbirth and for asthma and fever; also for scrofulous and venereal infections.

###### Notes.

In India the fruit is used for infertility; the root for venereal disease (Jain and DeFilipps 1991). In China the leaf is used as a depurative, a wash for skin diseases, and as a decoction for beri-beri; the seed is employed as an antidote for poisonous fish, crabs, etc. The plant is used in Guam and Samoa for fever, headache, hematemesis, pneumonia, stomachache, and wounds; and in the Solomon Islands, fumes from the steaming leaves are used to treat eye ailments, including blindness. Elsewhere the species is used for opthalmia and rheumatism (Duke and Ayensu 1985). Medicinal uses of this species in South China, Taiwan, Palau, Indonesia, the Philippines, and the Solomon Islands are discussed in Perry (1980).

The leaves contain an alkaloid-like compound, sterols, an aliphatic alcohol, an aliphatic ketone with glucose, fructose, sacccharose, resin, and gum (Duke and Ayensu 1985).

###### References.

Nordal (1963), Perry (1980).

### Lauraceae (Laurel family)

#### 1. *Cinnamomum* Schaeff.

##### Cinnamomum bejolghota

Taxon classificationPlantaeORDOFAMILIA

(Buch.-Ham.) Sweet (= C. obtusifolium (Roxb.) Nees)

###### Names.

**Myanmar**: *na-lin-gyaw*, *maza* (Kachin), *nakzik* (Chin), *hman-thein*, *lulin-gyaw*, *tauku-ywe*, *thit-kyabo*. **English**: wild cassia.

###### Range.

Tropical and temperate Asia. Grows naturally throughout Myanmar, with the exception of the hot zone; especially found in Bago, Mandalay, and Sagaing.

###### Uses.

Note: The interaction of the bark powder with jaggery can be fatal. Use of the bark powder for any treatment requires avoiding consumption of jaggery and all other sweet foods. *Bark*: Both the tree and root bark “open up vapors” and have cooling properties with activity against toxins. The ground bark is mixed with water and a small amount of salt to make a paste applied topically to deliver vapors of the medicine to alleviate scorpion stings and spider bites, aching body parts, areas of inflammation, and itchy patches. The paste is also applied externally or taken orally for other conditions, including exposure to detrimental cooking fumes, illnesses caused by persistent sores, and high fever with delirium. The paste with added salt is ingested for constipation. Bark, formed into balls with cooked rice, is toasted and soaked in water; the water from soaking is then used to make bark paste, which is taken for stomach bloating and distension, as well as for diarrhea. Bark paste made with water is given as a treatment for diphtheria, dengue hemorrhagic fever, severe diarrhea, female malaise, weakness, and fatigue. Bark paste made with commercially available menthol balm is applied topically or taken orally for problems experienced by those over the age of 50, including limb heaviness, aches and pains, tingling of the knees from excessive movement, pins and needles from sitting too long, and fatigue from exertion. Liquid from boiled bark is used as a wash for to accelerate healing of sores caused by threadworm infections. The paste is applied topically, in a circle around the eyes, as a remedy for aching eyes and dimming vision. A mixture of the powder and lemongrass powder is applied topically to alleviate soreness of breasts and taken orally to heal inflammation in the liver, lungs, and intestines. Bark powder is also inhaled to clear stuffy noses and sinus infections. A mixture of bark powder and water reserved from washing rice is used as a remedy for gonorrhea, intestinal and urinary infections, heart irregularities, dry lips, and dry throat.

###### Note.

The medicinal uses of this species in India are discussed in Jain and DeFilipps (1991).

###### Reference.

Agricultural Corporation (1980).

##### Cinnamomum camphora

Taxon classificationPlantaeORDOFAMILIA

(L.) J.Presl

###### Names.

**Myanmar**: *payuk*, *payoke-pin*. **English**: camphor, camphor tree.

###### Range.

China, Taiwan, Japan. Cultivated all over Myanmar; also, grows naturally in the temperate northern parts of the country.

###### Uses.

*Wood* and *Leaf*: Serve as an antispasmodic, diaphoretic, and stimulant. *Leaf*: Oil extracted from leaves is mixed with *shein-kho* (*Gardenia resinifera*) and made into pellets taken during an asthma attack. The oil is also used in making medicines to treat dizziness, aches and pains, and various male and female related disorders. Camphor is placed on the teeth to relieve toothaches. It can be crushed with water and applied on scorpion sting; and, soaked in rose water, it is given orally to treat arsenic poisoning.

###### Notes.

Medicinal uses of this species in India are discussed in Jain and DeFilipps (1991). Indigenous medicinal uses of this species in the Andaman and Nicobar Islands (India) are described by Dagar and Singh (1999). Medicinal uses of this species in China are discussed by Duke and Ayensu (1985). The medicinal uses of the species in Korea, China, and Indo-China are discussed in Perry (1980).

Chemical constituents, pharmacological action, and medicinal use of this species in Indian Ayurveda are discussed in detail by Kapoor (1990). Details of the active chemical compounds, effects, herbal usage and pharmacological literature of this plant are given in Fleming (2000). Worldwide medicinal usage, chemical composition and toxicity of this species are discussed by Duke (1986).

###### References.

Nordal (1963), Agricultural Corporation (1980).

##### Cinnamomum tamala

Taxon classificationPlantaeORDOFAMILIA

(Buch.-Ham.) T. Nees & Eberm.

###### Names.

**Myanmar**: *thit-jaboe*. **English**: Ceylon cinnamon.

###### Range.

Himalayas, in Bhutan, India, Nepal, and West Pakistan. In Myanmar, a cultivar that thrives in Tanintharyi Division, upper Chindwin, northern Shan State, Bamaw, and Rakhine State.

###### Use.

*Bark*: Effective against disorders of bile, diarrhea, excessive bleeding, sweating, vomiting, nausea and motion sickness. Taking the bark powder together with *Acacia catechu* cures diarrhea. A paste of the bark is mixed with other medicines and given to patients to cure influenza, coughing, lack of semen, and dysentery. Boiled and drunk, it can cure dysentery. *Oil*: Pressed into an aching tooth to cure the pain. The oil can be used as ear drops to treat earaches. Up to 2–4 drops of the oil can be taken to treat bloated stomachs. About 2 drops of the oil can be given two to three times a day to treat typhoid.

###### Reference.

Agricultural Corporation (1980).

##### Cinnamomum verum

Taxon classificationPlantaeORDOFAMILIA

J.Presl (= C. zeylanicum Blume)

###### Names.

**Myanmar**: *hmanthin*, *thit-kyabo*. **English**: cinnamon.

###### Range.

Sri Lanka and southwestern India. Found growing naturally not only in evergreen tropical forests, but also in other places around Myanmar.

###### Uses.

*Bark*: Used as a digestive and aphrodisiac. *Seed*: A paste made from the seeds used around the eyes to treat eye disorders. The paste taken with a liquid such as yogurt for seven days is used to treat chronic diarrhea. Taken with milk, it is used to treat gonorrhea. Paste made with distilled water can be taken to control excessive urination. A small amount of seed ash together with sugar is used for hemorrhoids.

###### Notes.

Indigenous medicinal uses of this species in the Andaman and Nicobar Islands (India) are described by Dagar and Singh (1999). Medicinal uses of the species in Indo-China, the Malay Peninsula, and India are discussed in Perry (1980),

The medicinal uses of this plant in the Caribbean region, as well as its chemistry, biological activity, toxicity and dosages, are discussed by Germosén-Robineau (1997). Details of the active chemical compounds, effects, herbal usage, and pharmacological literature of this plant are given in Fleming (2000). Worldwide medicinal usage, chemical composition, and toxicity of this species are discussed by Duke (1986).

“The bark is official in many modern pharmacopeias.” and the species has been used in medicine and as a spice since ancient times. Reported constituents of its volatile oil include cinnamic aldehyde, hydrcinnamic aldehyde, benzaldehyde, cuminic aldehyde, nonylic aldehyde, eugenol, caryophyllene, 1-phellandrine, p-cymene, pinene, methyl-n-amyl ketone, and 1-linalol (Perry 1980).

###### References.

Nordal (1963), Agricultural Corporation (1980).

### Laxmanniaceae (Laxmannia family)

#### 1. *Cordyline* Comm. ex R.Br.

##### Cordyline fruticosa

Taxon classificationPlantaeORDOFAMILIA

(L.) A. Chev. (= C. terminalis (L.) Kunth)

###### Names.

**Myanmar**: *zawgyi taung whay pin*, *zawma*, *kone-line*, *kun-linne.* **English**: boundary mark, dragon’s blood, ti plant.

###### Range.

Eastern Asia, East Indies and South Pacific Islands to Hawaii. Found throughout Myanmar, especially Mandalay and Shan; cultivated.

###### Uses.

*Whole plant*: The plant’s five parts are stewed with sugar and taken to restore regular menstruation; boiled, mixed with the water from boiling *kazun-ywet* (*Ipomoea aquatica*) leaves with sugar, and taken daily for lung ailments; or crushed for juice, which is mixed with ginger and jaggery syrup in equal parts to make a tonic taken by women to treat menopausal symptoms, clear the complexion, and for stamina and overall health. *Leaf*: The leaves of the plant, an astringent with cooling properties, are boiled in water and taken for vomiting of blood, passing of blood, and hemorrhaging. To regulate the bowels, the leaves are stewed with sugar and ingested, or water from boiling the roots is taken. For intestinal and liver inflammation, the leaves are stewed with jaggery. Tender young leaves are eaten as a remedy for dysentery or as a bowel regulator. Boiled with human milk, the leaves are taken for lung, liver, and kidney infections. For chest pains, leaves are boiled with cow’s milk. *Root*: As treatment for nosebleeds and sinusitis, the roots are made into a paste and inhaled. A root paste is also used for wet and dry scabies, as well as for sores and cracks in the groin; mixed with a bit of salt, the root paste makes an ointment to heal tongue sores. *Stem*: Rhizome used in diarrhea and dysentery.

###### Note.

In India the rhizome is eaten with betel (*Piper betle*) nut to cure diarrhea (Jain and DeFilipps 1991).

###### References.

Nordal (1963), Agricultural Corporation (1980), Forest Department (1999).

### Lecythidaceae (Brazil-nut family)

#### 1. *Barringtonia* J.R.Forst. & G.Forst.

##### Barringtonia acutangula

Taxon classificationPlantaeORDOFAMILIA

(L.) Gaertn.

###### Names.

**Myanmar**: *kyi*, *kyi-ni*, *ye-kyi*. **English**: Indian oak.

###### Range.

India to northern Australia. Widely distributed in Myanmar.

###### Uses.

*Leaf*: Used to treat dysentery and diarrhea. *Fruit*: Used for blood diseases. *Seed*: Used to treat opthalmia. Root: An aperient.

###### Notes.

In India a decoction of the bark is used as a mouthwash for toothache and gum pain; the stem is used for toothache; leaf juice is used for diarrhea; the fruit is used for nasal catarrh; the seed for liver problems; and an unspecified part, in a mixture with herbs, is used to treat cholera (Jain and DeFilipps 1991). Medicinal uses of the species in Indo-China and the Philippines are discussed in Perry (1980).

###### References.

Nordal (1963), Perry (1980).

#### 2. *Careya* Roxb.

##### Careya arborea

Taxon classificationPlantaeORDOFAMILIA

Roxb.

###### Names.

**Myanmar**: *bambwe*, *hou-no*, *mai-pinngo*, *sangawn-gmawt*, *thelaw*. **English**: patana oak, slow match tree, tummy wood.

###### Range.

Myanmar to the Malay Peninsula. Widely distributed in Myanmar.

###### Uses.

*Bark*: Used to treat snakebite. *Leaf*: Used to treat ulcers.

###### Notes.

In India the bark is used for snakebite; the flower for prolapsus ani and fistula ani, also in preparations for cold and cough (Jain and DeFilipps 1991). In Indo-China the bark is an ingredient in an emollient embrocation utilized as an antipyretic and antipruritic during the eruption of smallpox and chickenpox (Perry 1980).

###### Reference.

Perry (1980).

### Liliaceae (Lily family)

#### 1. *Fritillaria* L.

##### Fritillaria cirrhosa

Taxon classificationPlantaeORDOFAMILIA

D.Don (= F. roylei Hook.)

###### Names.

**Myanmar**: *gamone-kyet-thon-phyu*, *gamon-kyeethun-phyu*, *machit oo*, *machyit* (Kachin). **English**: fritillaria.

###### Range.

Eastern Asia - Himalayas. Cultivated in Myanmar. Found abundantly in Kachin State and other northern parts covered in ice; plants live under the ice and emerge only with melting of the ice.

###### Uses.

Root (Bulb): With a bitter yet savory taste, the bulbs are said to promote longevity. They are considered very important to humans, and help to increase waning body heat. The plant is used to prevent and alleviate sores, asthma, anemia, dry coughs, cysts, problems with blood vessels and varicose veins; also aching joints, urination problems, chronic illnesses, and fevers. To cure asthma and leprosy, the bulb is powdered, boiled together with orange (tangerine) skin, and ingested. One teaspoon of a mixture of bulb powder soaked in half a large bottle (most likely 750 ml) of honey is taken (once in the morning and once at night) for male-related conditions. The bulb powder is also used to promote good sleep, appetite, and longevity.

###### Notes.

The species has been recorded as medicinally useful for abcess, snakebite and as a scorpion and spider antidote; as an expectorant and for cough, asthma, fever, eye, viscera; labor, lactogogue; rheumatism, dysuria, hemorrhage, marrow, cancer, tuberculosis, syphilis; poison (Duke 2009). In China there are at least seven species of *Fritillaria*, all used in the same way. The bulbs are considered to be “especially good for the lungs” and to dissolve phlegm; they are also used to treat swollen throat (Peritonsillar abscess) (Perry 1980).

###### Reference.

Agricultural Corporation (1980).

### Linaceae (Flax family)

#### 1. *Linum* L.

##### Linum usitatissimum

Taxon classificationPlantaeORDOFAMILIA

L.

###### Names.

**Myanmar**: *bi-thawar*, *hnan-kyat*, *migyaung-kumbat*, *paiksan*. **English**: flax, linseed.

###### Range.

Probably Asia; an ancient cultigen, widely grown in temperate regions for fiber, and seed for linseed oil. Cultivated in Myanmar.

###### Uses.

*Seed*: Used to treat ulcers and for production of linseed oil; oil used as a base for ointments.

###### Notes.

In India the bark and leaf are used to treat gonorrhea; the flower is a cardiac tonic and nervine; dried ripe seeds are used as a demulcent poultice for rheumatism and gout, as well as employed internally for gonorrhea and urinogenital irritations; and the seed’s oil is mixed with limewater and applied to burns (Jain and DeFilipps 1991). In China the whole plant and its oil are used in making medicines; the seed is used for emollient cataplasm and catarrh; and oilseed cake is used to treat mental deficiencies in adolescents (Duke and Ayensu 1985).

The oilseed cake contains the amino acid arginine and 4% dry weight glutamic acid. L-glutamic acid is used in its free state in the treatment of metal deficiencies in infants and adolescents (Perry 1980). The genus *Linum* contains the anti-cancer agents 3’-demethylpodophyllotoxin, podophyllotoxin, and beta-sitosterol (Duke and Ayensu 1985).

###### References.

Nordal (1963), Perry (1980).

### Loganiaceae (Strychnine family)

#### 1. *Strychnos* L.

##### Strychnos potatorum

Taxon classificationPlantaeORDOFAMILIA

L.f.

###### Names.

**Myanmar**: *khabaung yay-kyi*, *mango-taukpa-tit* (Mon). **English**: clearing nut tree, water-filter nut.

###### Range.

Tropical Africa, tropical Asia, especially eastern India and eastern Myanmar. Found growing naturally not only in evergreen tropical forests, but also elsewhere around the country.

###### Uses.

Note: *This plant can cause blindness*; *caution is required to avoid contact with the eyes when using it to treat eye disorders and other conditions*.

*Seed*: Astringent and sweet, the easily digestible seeds are known to clarify water (similar to alum) and to relieve thirst and heat, neutralize poison, alleviate eye infections, and kill germs. A paste made from the ground seeds is applied topically in a circle around the eyes to treat eye disorders, improve vision, and clear blood spotting from the whites of the eyes; combined with honey it is applied topically in a circle around the eyes for cataracts. A mixture of seed paste with liquid yogurt taken for seven days is considered a cure for chronic, treatment-resistant diarrhea. A mixture of milk and seed paste is given as a remedy for gonorrhea. A mixture of seed ash and sugar is taken to alleviate bleeding hemorrhoids. The paste made with distilled water is used to treat excessive urination. Powdered seed coats are used to induce vomiting and treat dysentery.

###### Note.

In India a paste made from the root is applied locally to painful areas (mainly due to internal injury); the seed is used for a tonic, demulcent, stomachic, sedative, emetic and also for diarrhea, dysentery, gonorrhea, and eye troubles (Jain and DeFilipps 1991).

###### Reference.

Agricultural Corporation (1980).

##### Strychnos wallichiana

Taxon classificationPlantaeORDOFAMILIA

Steud. ex A.DC. (= S. cinnamomifolia Thwaites)

###### Name.

**Chinese**: *chang zi ma quia*.

###### Range.

China, Bangladesh, India, Indonesia, Sri Lanka, and Vietnam. In Myanmar, found in Bago and Mandalay.

###### Uses.

*Root*: Used to treat elephantiasis and epilepsy.

###### Note.

In India a decoction made from the root is used for elephantiasis, ulcers, rheumatism, epilepsy, and fever (Jain and DeFilipps 1991).

###### Reference.

Nordal (1963).

### Lythraceae (Henna family)

#### 1. *Lagerstroemia* L.

##### Lagerstroemia speciosa

Taxon classificationPlantaeORDOFAMILIA

(L.) Pers.

###### Names.

**Myanmar**: *pyinma-ywetthey*. **English**: queen’s crape myrtle.

###### Range.

India to Southeast Asia and Australia.

###### Uses.

*Bark* and *Leaf*: Purgative. *Leaf*: Used to treat diabetes. *Seed*: A narcotic. *Root*: Astringent.

###### Notes.

In India the bark and leaf are used as a purgative; the fruit is applied locally for aphthae of the mouth; the seed is used as a narcotic; and the root as a febrifuge, stimulant, and astringent (Jain and DeFilipps 1991). In Indo-China the root and bark are used as an astringent, and the leaves and fruit have hypoglycemic properties in treating diabetes mellitus. On the Malay Peninsula a decoction of the bark is ingested to treat abdominal pain and dysentery; the leaves are made into poultices to treat malaria and cracked feet. In Indonesia a cold infusion of the bark is used to treat diarrhea. In the Philippines the leaves are pounded or rubbed with salt and applied to the forehead and temples as a remedy for headache; a decoction of the old leaves and ripe fruit, taken orally, is considered to be the best antidiabetic part of the plant (if not available, younger and mature leaves can be used as a substitute); a decoction of the bark is drunk for hematuria, and that of the roots is drunk for jaundice as well as during puerperium (Perry 1980).

Reported constituents of leaves include tannin, glucose, and an antidiabetic principle; also an unnamed alkaloid has been found in the seed (Perry 1980).

###### References.

Nordal (1963), Perry (1980).

#### 2. *Punica* L.

##### Punica granatum

Taxon classificationPlantaeORDOFAMILIA

L.

###### Names.

**Myanmar**: *thale*. **English**: pomegranate.

###### Range.

Southeastern Europe to South Asia. Also naturalized, and widespread in cultivation.

###### Conservation status.

Least Concern [LC] (IUCN 2017).

###### Uses.

*Fruit*: Used as an anthelmintic and astringent.

###### Notes.

The plant is widely cultivated for its edible fruit and medicinal uses: The bark is used in a gargle for sore throat, bad breath, and as a wash for nosebleed (for the first two illnesses a decoction of the rind is used); a decoction of tender leaves serves as a gargle and another of the leaves and roots is drunk as a remedy for irregular menses; a plaster of the crushed leaves is applied to itch; crushed stem is similarly used; the fruit is rich in tannin (and thus astringent); a decoction of the rinds or fruit is used for diarrhea and dysentery and may also be applied as a wash or an injection against hemorrhoid and leucorrhea; the buds, flowers, and bark of the flowers mixed with sesame oil makes a dressing for burns; the fruit is both bechic and laxative; the root bark is used throughout the East as a specific for tapeworm, and is also anthelmintic against other intestinal worms (Perry 1980).

The medicinal uses of this species in India are discussed in Jain and DeFilipps (1991). Medicinal uses of the species in China are discussed by Duke and Ayensu (1985).

Chemical constituents, pharmacological action, and medicinal use of this species in Indian Ayurveda are discussed in detail by Kapoor (1990). Indigenous medicinal uses of this species in the Andaman and Nicobar Islands (India) are described by Dagar and Singh (1999).

The bark contains the alkaloids pelletierine, isopelletierine, methylpelletierine, pseudopelletierine, and considerable tannin; it has also been reported that the plant has a bacteriostatic effect (Perry 1980). Seeds and leaves of *Punica granatum* contain the hepatotoxic compound punicalagin, an oestrogenic chemical known as oestrone, and a form of pelletierine which is used for the expulsion of tapeworms (Lan et al.1998).

The chemical constituents, pharmacological activities, and traditional medicinal uses of this plant on a worldwide basis are discussed in detail by Ross (1999). A pharmacognostical profile including medicinal uses of this plant in Africa is given in Iwu (1993). Data on the propagation, seed treatment and agricultural management of this species are given by Katende et al. (1995). Details of the active chemical compounds, effects, herbal usage and pharmacological literature of this plant are given in Fleming (2000).

###### References.

Nordal (1963), Perry (1980).

#### 3. *Woodfordia* Salisb.

##### Woodfordia fruticosa

Taxon classificationPlantaeORDOFAMILIA

(L.) Kurz

###### Names.

**Myanmar**: *pan-le*, *panswe*, *pattagyi*, *yetkyi*. **English**: fire-flame bush, loosestrife, woodfordia.

###### Range.

Southeast Asia, including Madagascar, India, Pakistan, Sri Lanka, China, and Indonesia. In Myanmar found in Chin and Mandalay.

###### Conservation status.

Lower Risk/least concern [LC] (IUCN 2017).

###### Use.

*Flower*: Used to treat bowel complaints.

###### Notes.

On the Malay Peninsula the species is as an ingredient of a preparation to make a barren women fertile, a powder spread on a mother’s abdomen, and a drink given at the time of childbirth. In Indonesia the charred and pulverized fruit-bearing twigs provide an astringent powder sprinkled on wounds, and on the navel cord of newborn babies; the flower, leaf and fruit are used as an astringent to treat dysentery and sprue, as a diuretic against rheumatism, and also in treating dysuria and hematuria (Perry 1980).

Reported constituents include a tannin and a red pigment (Perry 1980).

###### Reference.

Perry (1980).

### Magnoliaceae (Magnolia family)

#### 1. *Magnolia* L.

##### Magnolia champaca

Taxon classificationPlantaeORDOFAMILIA

(L.) Baill. ex Pierre (= Michelia champaca L.)

###### Names.

**Myanmar**: *saka-wah*, *chyamka*, *laran* (Kachin), *kyom par* (Mon), *sam lung*, *mawk* (Shan). **English**: golden champak, michelia, yellow champak.

###### Range.

Temperate and tropical Asia. Plant grows naturally in Myanmar.

###### Conservation status.

Least Concern [LC] (IUCN 2017).

###### Uses.

Plant sweet and astringent with cooling properties, the flowers, leaves, fruits, bark, and roots are employed in medicines to increase sperm, promote heart function, and control bile and phlegm, as well as in preparations to alleviate vomiting and hemorrhaging of blood, urethral pain, leprosy, poisoning, itching, rashes, and sores. *Bark*: Used as an antidote, anthelmintic, and diuretic; to treat intermittent fever; also used in medicines to treat leprosy. The powdered bark is mixed with honey and licked to cure dry coughs. A decoction of bark is used as a remedy for chronic gas disorders and inflammation of the joints. *Leaf*: Used to treat colic. Water from soaking the young leaves is used as eye drops to cleanse the eyes and strengthen vision. A mixture of the juice from the crushed leaves and honey is given to ease chest pain and expel parasites, including threadworm and roundworm. *Flower*: Used to treat leprosy. A mixture of the crushed flowers and cold water is used as a diuretic and as a remedy for urinary tract and bladder problems. A decoction of the flowers is taken for gastric pain, gas disorders, kidney conditions, and gonorrhea. *Fruit*: The skin of the fruit is used in medicines to treat leprosy. *Fruit*, *Seed*: A paste made with water and either the fruits or the seeds is applied to heal cysts and boils on the thighs. *Root*: A mixture of yogurt with the crushed dried root or bark is applied as a poultice to heal sores.

###### Notes.

The medicinal uses of this species in India are discussed in Jain and DeFilipps (1991). Perry (1980) gives the medicinal uses of the species in China, Indo-China, the Malay Peninsula, and Indonesia.

Reported chemical constituents of the species include volatile oil, cineole, isoeugenol, benzoic acid, benzyl alcohol, benzaldehyde, p-cresol methyl ether, and alkaloid (alkaloid of the bark tested and found to not be poisonous) (Perry 1980).

###### References.

Nordal (1963), Agricultural Corporation (1980), Perry (1980), Forest Department (1999).

### Malpighiaceae (West Indian Cherry family)

#### 1. *Hiptage* Gaertn.

##### Hiptage benghalensis

Taxon classificationPlantaeORDOFAMILIA

(L.) Kurz

###### Names.

**Myanmar**: *bein-nwe*, *nwe-nathan-gwin*. **English**: hiptage.

###### Range.

Sri Lanka, southeastern Asia, Philippine Islands, Taiwan. From Myanmar to Timor. Cultivated in the tropics.

###### Uses.

*Bark*: A bitter. *Leaf*: Used as a remedy for skin diseases.

###### Notes.

In Indonesia the pounded bark is applied to fresh wounds (Perry 1980).

The medicinal uses of this species in India are discussed in Jain and DeFilipps (1991).

A glycoside-like substance, hiptagin, has been found in this species (Perry 1980).

###### Reference.

Perry (1980).

### Malvaceae (Mallow family)

#### 1. *Abelmoschus* Medik.

##### Abelmoschus esculentus

Taxon classificationPlantaeORDOFAMILIA

(L.) Moench (= Hibiscus esculentus L.)

###### Names.

**Myanmar**: *yonbade*. **English**: lady’s finger, wild okra.

###### Range.

Tropical Asia. Cultivated in Myanmar.

###### Uses.

*Fruit*: Used as stomachic and emollient.

###### Notes.

In India the root is used in a decoction for impotency (Jain and DeFilipps 1991). Indigenous medicinal uses of this species in the Andaman and Nicobar Islands (India) are described by Dagar and Singh (1999). Perry (1980) discusses the medicinal uses of the species in China, Indo-China, and the Philippines.

Medicinal uses of this plant in the Caribbean region, as well as its chemistry, biological activity, toxicity and dosages, are discussed by Germosén-Robineau (1997).

###### Reference.

Nordal (1963).

##### Abelmoschus moschatus

Taxon classificationPlantaeORDOFAMILIA

Medik.

###### Names.

**Myanmar**: *balu-wah*, *kon-kado*, *taw-wah*. **English**: musk mallow.

###### Range.

Tropical Asia. In Myanmar, found in Magway, Mandalay, Shan, and Yangon.

###### Uses.

*Leaf* and *Root*: Use for poultice. *Flower* and *Fruit*: Said to be a remedy for spermatorrhea. *Seed*: Said to have stomachic, tonic, diuretic, antihysteric, stimulant, and antispasmodic properties. *Root*: Pulverized and used to poultice boils and swellings.

###### Notes.

In India the seed is used as a stimulant, antispasmodic, stomachic, tonic, carminative, and aphrodisiac (Jain and DeFilipps 1991). Perry (1980) discusses the medicinal uses of the species in China, the Malay Peninsula, the Philippines, and Indonesia.

###### Reference.

Perry (1980).

#### 2. *Abroma* Jacq.

##### Abroma augustum

Taxon classificationPlantaeORDOFAMILIA

(L.) L.f.

###### Names.

**Myanmar**: *mway-ma-naing*, *mway-say*, *mway-seik-phay-pin*, *nga-be*, *ulat-kam-bala*. **English**: devil’s cotton, Indian hemp.

###### Range.

Himalayas, northern India, east to China, Micronesia, and Malaysia. In Myanmar, found in Kachin.

###### Use.

The plant is used for menstrual disorder (part unspecified by Nordal 1963).

###### Notes.

In India fresh or dried root-bark is used as a uterine tonic and emmenagogue; fresh juice is used for congestive and neuralgic dysmenorrhea (Jain and DeFilipps (1991). In Indonesia the root of this species is applied for itch; in the Philippines the root is used as an emmenagogue, and is considered especially useful for various forms of dysmenorrhea (Perry 1980).

The root-bark contains little alkaloid, much glucoside, resinous matter, much magnesium salts, calcium, and phosphates (Perry 1980).

###### Reference.

Nordal (1963).

#### 3. *Bombax* L.

##### Bombax ceiba

Taxon classificationPlantaeORDOFAMILIA

L. (= Salmalia malabarica (DC.) Schott & Endl)

###### Names.

**Myanmar**: *kadung*, *kawl-tung-peng*, *kroik*, *letpan*, *let-pau*, *mai-nio*. **English**: bombax, Indian kapok, red cottontree, red silk-cotton, silk-cottontree, simal.

###### Range.

Tropical Asia. Widely distributed in Myanmar.

###### Uses.

*Bark*: Astringent and diuretic. *Leaf*, *Flower*: Used for diabetes. *Root*: Astringent and diuretic; considered to have tonic properties (including sometimes the young root).

###### Notes.

Medicinal uses of this species in India are discussed in Jain and DeFilipps (1991). Perry (1980) discusses the uses of this species in China, Indo-China, Indonesia, and the Philippines.

###### References.

Nordal (1963), Perry (1980).

#### 4. *Ceiba* Mill.

##### Ceiba pentandra

Taxon classificationPlantaeORDOFAMILIA

(L.) Gaertn.

###### Names.

**Myanmar**: *le-moh-pin*, *lewah*, *thinbaw-letpan*. **English**: capoc, ceiba, kapok, silk-cottontree, white silk-cottontree.

###### Range.

Nicolson (1979) regards the original range as pantropical. Bornstein (1989) indicates that it is native from Mexico south to northern South America and the West Indies, and introduced and more or less naturalized in the Old World. Villiers (1973) notes an American origin for the plant, and that its presence in Gabon, West Africa is rarely in primary forest, and it is a species of zones occupied or cultivated by man. Cultivated in Myanmar.

###### Uses.

*Leaf*: Used in the treatment of gonorrhea. *Root*: Useful tonic; also employed as a diuretic. Juice from the roots is used to treat diabetes. The gum is used as a tonic, astringent, laxative, and restorative.

###### Notes.

Medicinal uses of this species in India are discussed in Jain and DeFilipps (1991). Indigenous medicinal uses of this species in the Andaman and Nicobar Islands (India) are described by Dagar and Singh (1999). Perry (1980) discusses the medicinal uses of the species in Indo-China, the Malay Peninsula, and the Philippines.

Data on the propagation, seed treatment, and agricultural management of this species are given by Katende et al. (1995) and Bekele-Tesemma (1993).

###### References.

Mya Bwin and Sein Gwan (1967), Perry (1980).

#### 5. *Gossypium* L.

##### Gossypium barbadense

Taxon classificationPlantaeORDOFAMILIA

L.

###### Names.

**Myanmar**: *nu-wah*. **English**: kidney cotton, sea island cotton, tree cotton.

###### Range.

Tropical America; said to have originated in South America. Cultivated in Myanmar.

###### Uses.

The seeds, roots, flowers, and leaves are employed. *Whole plant*: All parts used to alleviate skin problems, snakebites, scorpion stings, and shooting uterine pains. *Bark*: A decoction is taken to alleviate excessive menstrual bleeding. For white vaginal discharge, a paste made of the root with water reserved from washing rice is considered a remedy. *Leaf*: Preparations are used to control diseases involving gas, increase blood, promote urinary function, and protect against ear infections. Juice from crushed leaves is taken for diarrhea with indigestion. *Flower*: The bud, which is considered sweet, with cooling properties, is known for promoting weight gain, stimulating lactation, controlling bile and phlegm, alleviating thirst, supporting the memory, and focusing the mind. The flowers are used in a sherbet drink to alleviate mental disturbance or disease. Ash from the flower is pressed into sores to stimulate healing and new tissue formation. *Seed*: Used to increase lactation and virility. An ointment made from the crushed seed kernel is applied to soothe burns. Seed kernels stewed in milk are given for weakness of the brain. A paste made with the seeds, dried ginger, and water is used for inflammation of the testes. A decoction is used as a mouthwash or rinse to soothe toothaches. Roasted, pressed seeds are applied as a poultice to cure calluses and boils. *Root*: A decoction is given to clear urinary infections causing symptoms of burning sensation during urination and pain in passing urine.

###### Notes.

Medicinal uses of this species in India are discussed in Jain and DeFilipps (1991). Indigenous medicinal uses of this species in the Andaman and Nicobar Islands (India) are described by Dagar and Singh (1999).

Worldwide medicinal usage, chemical composition and toxicity of this species are discussed by Duke (1986).

###### Reference.

Agricultural Corporation (1980).

##### Gossypium hirsutum

Taxon classificationPlantaeORDOFAMILIA

L.

###### Names.

**Myanmar**: *wah.* **English**: American upland cotton, cotton tree.

###### Range.

Origin in Central America, Mexico and Greater Antilles

###### Uses.

Same as *Gossypium barbadense*.

###### Notes.

Medicinal uses of “*Gossypium* spp.” in China are discussed in Duke and Ayensu (1985). Details of the active chemical compounds, effects, herbal usage, and pharmaco- logical literature of the species *G. herbaceum* are given in Fleming (2000). Indigenous medicinal uses of the species in the Andaman and Nicobar Islands (India) are described by Dagar and Singh (1999).

The toxic properties, symptoms, treatment and beneficial uses of *Gossypium hirsutum*, *parts* of which are *poisonous*, are discussed by Nellis (1997). Details of the active chemical compounds, effects, herbal usage and pharmacological literature of this plant are given in Fleming (2000). Worldwide medicinal usage, chemical composition and toxicity of this species are discussed by Duke (1986).

###### Reference.

Agricultural Corporation (1980).

#### 6. *Grewia* L.

##### Grewia asiatica

Taxon classificationPlantaeORDOFAMILIA

L.

###### Names.

**English**: falsa, phalsa.

###### Range.

Native to southern India. Now widely cultivated in tropical countries. Reported from Myanmar.

###### Uses.

*Bark*: Demulcent. *Leaf*: Used as an application for eruptions. *Root*: Used for medicinal purposes. *Fruit*: Astringent.

###### Note.

In India the bark is demulcent; the leaf is put on eruptions; the fruit is astringent, cooling, and stomachic; and the root-bark is used for rheumatism (Jain and DeFilipps 1991).

###### Reference.

Perry (1980).

##### Grewia hirsuta

Taxon classificationPlantaeORDOFAMILIA

Vahl

###### Names.

**Myanmar**: *kyet-tayaw*, *tayaw*. **English**: hairy indigo.

###### Range.

China, Bangladesh, Cambodia, India, Laos, Malaysia, Myanmar, Nepal, Sri Lanka, Thailand, and Vietnam. In Myanmar, found in Bago, Mandalay, and Shan.

###### Use.

*Root*: Used for medicinal purposes (exact uses not given in Perry 1980).

###### Note.

Duke (2009) lists the following medicinal uses as given for this species: Treatment of diarrhea, dysentery, and wounds; also a suppuative.

###### Reference.

Perry (1980).

##### Grewia nervosa

Taxon classificationPlantaeORDOFAMILIA

(Lour.) Panigrahi (= G. microcos L.)

###### Names.

**Myanmar**: *myat-y*a, *mya-yar*, *myin-kahpan*, *ye-mya-yar*. **English**: microcos.

###### Range.

China, Cambodia, India, Indonesia, Laos, Malaysia, Myanmar, Sri Lanka, Thailand, and Vietnam. In Myanmar, found in Ayeyarwady, Bago, Mandalay, Mon, Taninthayi, and Yangon.

###### Uses.

*Whole plant*: Used for skin diseases and indigestion.

###### Note.

In India the whole plant is used for syphilitic ulcers and eczema, and the leaf is narcotic (Jain and DeFilipps 1991).

###### Reference.

Nordal (1963).

##### Grewia polygama

Taxon classificationPlantaeORDOFAMILIA

Roxb.

###### Names.

**English**: dysentery bush, emu-berry, turkey bush.

###### Range.

Northwestern Himalayas east to Bangladesh and Sri Lanka.

###### Use.

*Leaf*: Used for dysentery.

###### Notes.

Reported medicinal uses for this species include treatment of headache, tiger bite, carbuncle, cholera, diarrhea, dysentery, eye, and sores (Duke 2009). The seed is said to produce a sub-acid drink when boiled (Perry 1980).

###### Reference.

Perry (1980).

#### 7. *Helicteres* L.

##### Helicteres isora

Taxon classificationPlantaeORDOFAMILIA

L.

###### Names.

**Myanmar**: *thunge-che*, *tingkyut*. **English**: East Indian screw tree.

###### Range.

Malay Archipelago. In Myanmar, found in Kachin and Taninthayi.

###### Uses.

*Bark* and *Root*: Stomachic. *Fruit*: Ingredient of a liniment.

###### Notes.

In India the leaf is used for stomachache; the fruit for stomach disorders and rickets in babies; the seed for stomach pain and dysentery, also the oil is massaged on body to relieve pain; the root for stomachache on sores and carbuncles (in combination with other plants), and for colic (Jain and DeFilipps 1991). Perry (1980), in addition to Myanmar, lists the medicinal uses of the species in the Malay Peninsula, Indonesia, South China, and Taiwan.

###### Reference.

Perry (1980).

#### 8. *Hibiscus* L.

##### Hibiscus cannabinus

Taxon classificationPlantaeORDOFAMILIA

L.

###### Names.

**Myanmar**: *chin-baung-gyi*, *chin-baung-kha*, *kenaf*. **English**: bastard jute, bimli jute, bimlipatum jute, Bombay hemp, Deckaner hemp, Indian hemp.

###### Range.

Probably Africa. Cultivated in Myanmar.

###### Use.

*Leaf*: Used a as a laxative.

###### Notes.

The medicinal uses of this species in India are discussed in Jain and DeFilipps (1991) as follows: The leaf is used as a purgative; the juice of the flower is used with black pepper and sugar to cure acidity and biliousness; the seed is applied externally to bruises and pains; also used as an aphrodisiac and as a fattening substance. This species also yields a good fiber, much like jute, and is similarly used; also the seeds yield an oil that is burned in Africa (Bailey and Bailey 1976).

###### Reference.

Nordal (1963).

##### Hibiscus sabdariffa

Taxon classificationPlantaeORDOFAMILIA

L.

###### Names.

**Myanmar**: *bilat-chinbaung*, *chinbaung-ni*, *chin-bong*, *chinebaune*, *phat-swon-pan*, *sum-bawng*. **English**: Indian sorrel, Jamaica sorrel, rozelle, sorrel.

###### Range.

Tropical Africa; now widely cultivated and naturalized throughout the tropics. Cultivated in Myanmar.

###### Uses.

*Leaf*: Used as an emollient. *Seed*: Used to treat debility.

###### Notes.

In India the enlarged succulent calyx is boiled in water, and the resulting drink used for biliousness; the leaf, calyx and seed are used as an antscorbutic and diuretic; and the fruit is used as an antiscorbutic (Jain and DeFilipps 1991). Medicinal uses ot the species in Taiwan and in the Philippines are given in Perry (1980).

Due to its high intestinal antiseptic action, the species is used in treating arteriosclerosis (Perry 1980).

###### Reference.

Perry (1980).

##### Hibiscus schizopetalus

Taxon classificationPlantaeORDOFAMILIA

(Dyer) Hook.f.

###### Names.

**Myanmar**: *khaung-yan*, *khaung-yan-ywet-hla*, *mawk-manu*, *mawkmnae*, *pan-swe-le*. **English**: fringed hibiscus, rose of China, shoe flower.

###### Range.

Tropical East Africa. Cultivated in Myanmar.

###### Uses.

*Fruit*: Used as stomachic and emollient.

###### Notes.

The medicinal uses of this species in India are discussed in Jain and DeFilipps (1991) as follows: The leaf is used as an emollient, anodyne, and laxative; the flower as an emollient, aphrodisiac, and decoction for bronchial catarrh; also for excessive menstruation, fever, and skin disease. The root is used to treat gonorrhea. Medicinal uses of this species in China are discussed in Duke and Ayensu (1985). Here the leaves and flowers are made into a paste and used as a poultice on cancerous swellings and mumps; the flowers are also used for carbuncles, mumps, fever, fistula, and cancerous and other sores. Perry (1980) discusses the medicinal uses of the species in China, Indo-China, the Malay Peninsula, Indonesia, and the Philippines.

Duke and Ayensu (1985) include a significant amount of information on the chemistry of the species.

###### Reference.

Nordal (1963).

##### Hibiscus vitifolius

Taxon classificationPlantaeORDOFAMILIA

L.

###### Names.

**Myanmar**: *thin-paung*. **English**: tropical fanleaf, tropical rose mallow.

###### Range.

Tropical and subtropical regions of Old World. In Myanmar, found in Yangon.

###### Uses.

*Fruit*: Used as stomachic and emollient.

###### Note.

Antiviral activity has been detected utilizing an extract of the plant (Vijyan et al. 2004).

###### Reference.

Nordal (1963).

#### 9. *Kleinhovia* L.

##### Kleinhovia hospita

Taxon classificationPlantaeORDOFAMILIA

L.

###### Names.

**Myanmar**: *o-dein*, *pashu-phet-wun*. **English**: guest tree.

###### Range.

Tropical Asia, tropical eastern Africa, and Australia. Cultivated in Myanmar.

###### Use.

*Seed*: Used to treat dysentery.

###### Notes.

In the Philippines a decoction of the leaves of this species provides a treatment for scabies, and also locally for all forms of dermatisis (Perry 1980).

The species contains prussic acid, a triterpinoid, and an essential oil (Perry 1980). The chemical betulin, extracted from the fruit, has been found to be an anti-carcinomic, anti-feedant, anti-flu, anti-HIV, anti-inflammatory, anti-tumor, anti-viral, cytotoxic, hypolipemic, a prostaglandin-synthesis inhibitor, and topoisomerase-I-inhibitor (Duke 2009).

###### Reference.

Nordal (1963).

#### 10. *Kydia* Roxb.

##### Kydia calycina

Taxon classificationPlantaeORDOFAMILIA

Roxb.

###### Names.

**Myanmar**: *baluma-shaw*, *dwabok*, *magan*, *magan-kaja*, *magap*, *mickyat*, *phet-wun-ni*, *tabo*, *tayaw-ni*. **English**: kydia.

###### Range.

Sikkim to Indochina. Also cultivated; propagated by seeds and cuttings. In Myanmar, found in Chin, Kachin, Mandalay, and Yangon.

###### Use.

*Leaf*: Included in making an embrocation.

###### Notes.

The species is used as anodyne, for pain, and as a sialogogue (Duke 2009). The seed contains the following acids: Lauric, myristic, palmitic, stearic, arachidic, behenic, oleic, linoleic, and cyclopropenoid fatty acid (Daulatabad et al. 1999).

###### Reference.

Perry (1980).

#### 11. *Malvastrum* A.Gray

##### Malvastrum coromandelianum

Taxon classificationPlantaeORDOFAMILIA

(L.) Garcke

###### Names.

**Myanmar**: *taw-pilaw*. **English**: threelobe false mallow.

###### Range.

Tropical regions in both Old and New Worlds. In Myanmar, found in Kachin and Sagaing.

###### Uses.

*Whole plant*: Used as an expectorant and emollient.

###### Note.

In India the leaf is used as a salve to both cool and heal inflamed wounds and sores; the flower is used as a diaphoretic and pectoral (Jain and DeFilipps 1991).

###### Reference.

Nordal (1963).

#### 12. *Mansonia* J.R.Drumm.

##### Mansonia gagei

Taxon classificationPlantaeORDOFAMILIA

J.R.Drumm.

###### Names.

**Myanmar**: *kala-met*, *ka-la-mak*. **English**: bastard sandalwood.

###### Range.

India, Myanmar, Thailand. In Myanmar, found in Mandalay and Taninthayi.

###### Uses.

*Wood* and *Root*: Ground into a paste, and applied externally or taken orally to eliminate phlegm and treat heart diseases, urinary disorders, and anemia. The paste is also applied topically to the body for a cooling effect and to alleviate itches.

###### Note.

Several medicinally useful chemicals have been extracted from the heartwood of this species: Among these are coumarin derivitives, mansorins and mansonones, which have shown antiestrogenic activity; also mansorins which have shown antifungal, antioxidant, and antilarval activity (Tiew et al. 2003).

###### References.

Agricultural Corporation (1980), Forest Department (1999).

#### 13. *Pterospermum* Schreb.

##### Pterospermum acerifolium

Taxon classificationPlantaeORDOFAMILIA

(L.) Willd.

###### Names.

**Myanmar**: *magwinapa*, *sinna*, *taung-petwun*, *taw-kalamet*. **English**: *kanack champa* (adopted Hindi name).

###### Range.

India to Java. Widely distributed in Myanmar.

###### Uses.

*Bark*, *Leaf*: Used in skin diseases (smallpox). *Leaf*: Used as a styptic. *Flower*: Used as a tonic.

###### Notes.

In India the plant is considered antiseptic, depurative, and tonic; also employed for eruptions, fever, inflammation, leprosy, menorrhagia, puerperium, smallpox, sores, and tumors (Jain and DeFilipps 1991).

In South China a tincture of the root of another species in the genus, *Pterospermum heterophllum*, is drunk to treat rheumatism and ostealgia; on the Malay Peninsula, the bark of *P. javanicum* is used in a poultice for abdominal complaints; in the Philippines the bark and flowers of *P. diversifolium* are charred and mixed with the glands of another species to cause suppuration for smallpox (Perry 1980).

###### References.

Nordal (1963), Perry (1980).

#### 14. *Sida* L.

##### Sida spinosa

Taxon classificationPlantaeORDOFAMILIA

L.

###### Names.

**Myanmar**: *katsi-ne*, *nagbala*, *thabyetsi-bin*. **English**: prickly fanpetals.

###### Range.

Pantropical.

###### Use.

*Root*: Tonic, diaphorectic, gonorrhea.

###### Reference.

Nordal (1963).

#### 15. *Triumfetta* L.

##### Triumfetta rhomboidea

Taxon classificationPlantaeORDOFAMILIA

Jacq. (= T. bartramia L.)

###### Names.

**Myanmar**: *kat-si-ne*, *katsine-galay*. **English**: burrbush.

###### Range.

Throughout the tropics. In Myanmar, found in Bago, Chin, Kachin, Mandalay, and Yangon.

###### Use.

*Leaf*, *Flower*, *Fruit*, *Root*: Used to facilitate childbirth.

###### Notes.

In China the plant is used for abscesses and other skin problems (Duke and Ayensu 1985). In Tonga the species is used for burns and scalds; in the Philippines, for internal ulcers; in Latin America, due to its mucilaginous property, it is used to make a refrigerant beverage (Duke and Ayensu 1985). Elsewhere, other species of the genus are used for blennorrhagia, boils, carbuncles, catarrh, colds, convulsion, diarrhea, dyspepsia, dysuria, earache, gastroenteritis, gonnorrhea, hangover, headaches, hepatosis, impotency, infertility, itch, jaundice, leprosy, leucorrhea, opthalmia, parturition, piles, renosis, snakebite, sores, sore throat, tumors, and venereal disease (Duke and Ayensu 1985).

###### Reference.

Nordal (1963).

#### 16. *Urena* L.

##### Urena lobata

Taxon classificationPlantaeORDOFAMILIA

L.

###### Names.

**Myanmar**: *kat-say-nei*, *kat-sine*, *nwar-mee-kat*, *popee* (Chin). **English**: aramina, bur mallow, Caesar weed, congo-jute, hibiscus burr.

###### Range.

Tropical regions of both hemispheres. Grows naturally throughout Myanmar.

###### Uses.

*Bark*: Dried and powdered, combined in equal amounts with sugar, and taken with milk twice daily to increase virility and sperm production. *Twig*: Chewed for toothaches. *Leaf*: A mixture of the crushed leaves and black pepper is taken once each morning and each night to remedy weight loss and low energy or with equal amounts of black sesame seeds and cooked over a slow fire to make an ointment applied to reduce edema. *Leaf*, *Root*: Used as a diuretic and expectorant. With equal parts of sweet, sour, astringent, hot, and spicy tastes, the leaves and roots are used in medicines to reduce phlegm and fever, prevent sores, for bile problems, to control venereal and urinary tract infections, and alleviate leprosy and skin diseases. Used to treat rheumatism. A decoction in ten times their weight in water is reduced to half its starting volume and given orally two to three times daily to reduce fever. A paste of the ground root with water applied twice daily is considered a cure for drooping breasts. Root powder, mixed vigorously in milk to form froth, is taken twice daily for asthma and bronchitis. The powder is also taken with hot water daily for chronic indigestion. A decoction of the roots is taken for fevers; and reduced to half its starting volume it is taken for inflamed and aching joints. A decoction of root bark is used to treat venereal disease and other debilitating conditions.

###### Notes.

Reported medicinal uses for this species include the treatment of headache, stomachache, gastritis, diarrhea, sore throat, fever, inflammation, colic, bronchitis, pneumonia, and as an expectorant; for sores, wounds, eruptions, boils, swelling, burns; as diuretic, for bladder and urogenital problems, and gonorrhea; for blennorrhagia, cataplasm, dysentery, hepataitis, pleurisy, dysentery, hematochezia, and yaws; as hemostat, emmenagogue, and anodyne; also as an emollient, for gingivitis, and for hangovers (Duke 2009). The medicinal uses of this species in India are covered in Jain and DeFilipps (1991). Medicinal used of this species in China are discussed in Duke and Ayensu (1985). Perry (1980) discusses the uses of the species in China, the Malay Peninsula, Indo-China, and the Philippines.

###### References.

Nordal (1963), Agricultural Corporation (1980), Perry (1980).

### Marantaceae (Arrowroot or Prayer-Plant family)

#### 1. *Maranta* L.

##### Maranta arundinacea

Taxon classificationPlantaeORDOFAMILIA

L.

###### Names.

**Myanmar**: *taung-sun*, *thinbaw-adalut*. **English**: American arrowroot, arrowroot, maranta.

###### Range.

Tropical America; now pantropic in distribution. Cultivated in Myanmar.

###### Use.

*Stem*: Rhizome used as a rubefacient; yields arrowroot.

###### Notes.

The rhizome, rich in starch, serves as a food for invalids. It is also used as an emollient, for diseases of the urinary tract, and for bowel complaints (Perry 1980).

Medicinal uses of this species in India are discussed in Jain and DeFilipps (1991).

Details of the active chemical compounds, effects, herbal usage, and pharmacological literature of this plant are given in Fleming (2000). Worldwide medicinal usage, chemical composition and toxicity of this species are discussed by Duke (1986).

###### Reference.

Nordal (1963).

### Martyniaceae (Martynia family)

#### 1. *Martynia* L.

##### Martynia annua

Taxon classificationPlantaeORDOFAMILIA

L. (= M. diandra Gloxin)

###### Names.

**Myanmar**: *se-kalon*. **English**: devil’s claw, iceplant, tiger’s claw.

###### Range.

China, Cambodia, India, Laos, Myanmar, Nepal, Pakistan, Sri Lanka, Vietnam; native of Central America, introduced and naturalized elsewhere. Cultivated in Myanmar.

###### Uses.

*Fruit*: Used in tuberculosis and for inflammation.

###### Note.

Reported medicinal uses for this species include alexiteric, adenopathy, alopecia, carbuncle, epilepsy, inflammation, scabies, sores, sore throat, and gargle (Duke 2009).

###### Reference.

Nordal (1963).

### Melastomataceae (Melastome family)

#### 1. *Melastoma* L.

##### Melastoma malabathricum

Taxon classificationPlantaeORDOFAMILIA

L.

###### Names.

**Myanmar**: *kyet-gale*, *linda-pabyin*, *myetpye*, *nyaung-ye-o-pan*, *sahkao*, *shame*, *wachyang*, *wagangga*. **English**: Indian rhododendron, melastoma.

###### Range.

India, southeastern Asia, Malay Archipelago, New Guinea, and the Philippines. In Myanmar, found in Ayeyarwady, Taninthayi, and Yangon.

###### Uses.

*Leaf*: Used in for chronic diarrhea and dysentery.

###### Notes.

In India the bark of the species is used for skin diseases; the leaf for smallpox and wounds; and the root for diarrhea and dysentery (Jain and DeFilipps 1991). In China the leaf is used as a febrifuge and for rickets (Duke and Ayensu 1985). In Taiwan the plant is used as a febrifuge and for rickets; on the Malay Peninsula for its astringent property and, in combination with the leaves of *Ageratum conyzoides* and *Hedyotis capitellata*, to treat dysentery (Perry 1980). In Indo-China the plant is used for treating diarrhea, leucorrhea, and dysentery; the leaves, flowering tops and roots are an astringent drug (Perry 1980).

###### Reference.

Nordal (1963).

#### 2. *Memecylon* L.

##### Memecylon edule

Taxon classificationPlantaeORDOFAMILIA

Roxb.

###### Names.

**Myanmar**: *byin-gale*, *lee-ko-kee*, *me-byaung*, *miat*, *mi-nauk*. **English**: ironwood tree.

###### Range.

Tropical India. In Myanmar, found in Kayin, Rakhine, Taninthayi, Yangon.

###### Uses.

*Bark*: Used in a fomentation. *Leaf*: Astringent.

###### Notes.

In Indo-China an infusion of the bark and leaves is used to treat fever; in India a decoction of the roots is used as an emmenagogue, and an infusion of the leaves is astringent and used to treat ophthalmia (Khare 2007). The leaves have strong anti-inflamatory and analgesic properties (Nualkaew et al. 2009).

###### Reference.

Perry (1980).

### Meliaceae (Mahogany family)

#### 1. *Aglaia* Lour.

##### Aglaia cucullata

Taxon classificationPlantaeORDOFAMILIA

(Roxb.) Pellegr. (= Amoora cucullata Roxb.)

###### Names.

**Myanmar**: *myauk-le-sik*, *thit-ni*. **English**: amoora, Pacific maple.

###### Range.

Bangladesh, India, Indonesia, Malaysia, Myanmar, Nepal, Papua New Guinea, the Philippines, Singapore, Thailand, and Vietnam. In Myanmar, found in Ayeyarwady and Rakhine.

###### Conservation status.

Data Deficient [DD] (IUCN 2017).

###### Uses.

*Leaf*: Used for inflammation. *Seed*: Used to treat rheumatism.

###### Notes.

Potent cytotoxic rocaglamide derivatives have been extracted from the fruits of this species (Chumkaew et al. 2006). Five compounds were isolated from an extract of the stem bark of *A. cucullata*. These included fridelin, stigmasterol, B-sitosterol, betulinic acid, and caffeic acid (Rahman et al. 2005b).

###### Reference.

Perry (1980).

#### 2. *Aphanamixis* Blume

##### Aphanamixis polystachya

Taxon classificationPlantaeORDOFAMILIA

(Wall.) R. Parker (= A. rohituka (Roxb.) Pierre)

###### Names.

**Myanmar**: *chaya-kaya*, *ta-gat-net*, *than-that-gyi*, *thit-ni*. **English**: rohituka, white cedar.

###### Range.

Low to middle elevations in mountainous regions. Sri Lanka, southeastern Asia, Sumatra; Pacific Islands (Solomon islands). In Myanmar, found in Taninthayi and Yangon.

###### Conservation status.

Lower Risk/least concern [LC] (IUCN 2017).

###### Use.

*Bark*: Used as an astringent.

###### Notes.

In Taiwan oil pressed out of the seed is used in medicine, also industry; in Indonesia a decoction of the bark is ingested as a remedy for chest pain associated with a cold (Perry 1980).

Powderd bark is used to treat diseases of the liver, including jaundice; enlarged spleen; anemia; internal tumors; abdominal diseases, including ascitis; intestinal worms; and urinary disorders; also a root paste is used for leucorrhoea (Khare 2004).

The sap from a tapped tree is said to be *poisonous*; also, traces of alkaloid and a poisonous bitter substance have been found in the fruit wall (Perry 1980).

###### Reference.

Perry (1980).

#### 3. *Azadirachta* A.Juss.

##### Azadirachta indica

Taxon classificationPlantaeORDOFAMILIA

A.Juss.

###### Names.

**Myanmar**: *tama*, *tamaga*, *margosa*, *neem*. **English**: Indian lilac.

###### Range.

Tropical Asia; also cultivated. Grows naturally in the hot regions of Myanmar.

###### Uses.

*Whole plant*: Bitter in taste, hot and sharp when digested, and with cooling properties, the flowers, sap, oil, bark, leaves, fruits, stems, and twigs are known to dispel gas, phlegm, and bile. *Sap*: Used in making tonics and digestives. The oil, which is applied topically for itching and rashes, is ingested for deworming. *Gum*: Used as a demulcent and tonic. *Bark*: Used as a tonic. Also, made into a paste and taken with salt to reduce fever. The inner bark is also made into a paste but applied topically to alleviate joint aches and pains. A decoction of the bark reduced to one-third its starting volume is used as a mouthwash to relieve toothaches. *Leaf*, *Bark*, and *Oil*: Used in treatment of skin diseases; also, as a tonic, anthelmintic, and insecticide. *Leaf*: Crushed leaves are made into a poultice applied as a remedy for scabies and boils. A decoction of the leaves is used as a wash to alleviate rashes, itching, and bumps on the skin. Their juice is used as an eyewash, and to relieve itching and heat. Powdered after roasting until charred leaves are mixed with salt and used daily as toothpaste to prevent toothaches, as well as to whiten and strengthen teeth; the bare twigs are used as toothpicks to help keep the teeth clean. Pulped leaves are applied to psora and other pustular eruptions. *Oil*, *Leaf* and *Fruit*: Utilized as a local stimulant and as an insecticide. *Flower*: Used as a stomachic; also, inhaled to alleviate dizziness. *Fruit*: Eaten daily as a remedy for urinary infections.

###### Notes.

The medicinal uses of this species in India are discussed in Jain and DeFilipps (1991). Medicinal uses of this species in East and Southeast Asia are discussed in Perry (1980). Indigenous medicinal uses of this species in the Andaman and Nicobar Islands (India) are described by Dagar and Singh (1999).

Details of the active chemical compounds, effects, herbal usage, and pharmacological literature of this plant are given in Fleming (2000). Traditional medicinal uses, chemical constituents, and pharmacological activity of this species are discussed by Ross (2001).

###### References.

Nordal (1963), Agricultural Corporation (1980), Perry (1980).

#### 4. *Chukrasia* A.Juss.

##### Chukrasia tabularis

Taxon classificationPlantaeORDOFAMILIA

A.Juss.

###### Names.

**Myanmar**: *kin-thabut-gyi*, *taw-yinma*, *yinma*. **English**: Chittagong wood, golden mahogany.

###### Range.

Myanmar, Andamans, China, Bhutan, India, Indonesia, Laos, Malaysia, Nepal, Sri Lanka, Thailand, Vietnam, and Pakistan. In Myanmar, found in Mandalay, Shan, and Yangon.

###### Conservation status.

Lower Risk/least concern [LC] (IUCN 2017).

###### Uses.

*Bark*: Used as an astringent and antidiarrheic.

###### Notes.

In India the bark is used as a tannin-containing astringent (Jain and DeFilipps 1991). The medicinal uses of the species in Indonesia are listed in Perry (1980).

###### References.

Nordal (1963), Perry (1980).

#### 5. *Heynea* Roxb.

##### Heynea trijuga

Taxon classificationPlantaeORDOFAMILIA

Roxb. ex Sims

###### Names.

**Myanmar**: *taagat-ta-gyi*. **English**: gargu.

###### Range.

Hainan and North Vietnam. In Myanmar, found in Bago, Mandalay, and Yangon.

###### Use.

*Bark*, *Leaf*: Used as a tonic.

###### Note.

In Hainan and North Vietnam, as well as on the Malay Peninsula, a decoction of the leaves is given to treat cholera; the seeds are *poisonous* (Perry 1980).

###### Reference.

Nordal (1963).

#### 6. *Sandoricum* Cav.

##### Sandoricum koetjape

Taxon classificationPlantaeORDOFAMILIA

(Burm.f.) Merr.

###### Names.

**Myanmar**: *santal*, *thitto*. **English**: donka, lolly fruit, red santol, santol, sentol, sentul.

###### Range.

Believed originally native to former Indochina and Peninsular Malaysia. Rare wild, but commonly cultivated from Thailand and Indo-China into southeastern Asia. In Myanmar found in Ayeyarwady, Kayin, Mon, Taninthayi, and Yangon.

###### Use.

*Root*: Used to treat dysentery.

###### Note.

In India the root is used for dysentery and diarrhea; it is an astringent, aromatic, antispasmodic, stomachic and carminative (Jain and DeFilipps 1991).

###### Reference.

Perry (1980).

#### 7. *Toona* (Endl.) M.Roem.

##### Toona sureni

Taxon classificationPlantaeORDOFAMILIA

(Blume) Merr.

###### Names.

**Myanmar**: *kashit-ka*, *latsai*, *mai-yum*, *taung-tama*, *thit-kado*. **English**: Australian red cedar, moulmein cedar, red cedar.

###### Range.

India and Indo-China south to Southeast Asia. In Myanmar, found in Bago, Mandalay, Shan, and Yangon.

###### Use.

*Bark*: Used as a strong astringent.

###### Notes.

In India the bark is applied externally to ulcers, used for chronic infantile dysentery, antiperient, tonic, and astringent; the flower is used as an enmenagogue (Jain and DeFilipps 1991). In Indo-China the bark is considered to be tonic, antiperiodic, and antirheumatic; in Indonesia the bark of the red form is used as an astringent and tonic, considered good for treating chronic diarrhea, dysentery, and other intestinal problems (Perry 1980).

An extract of the leaves has antibiotic activity against *Staphylococcus*; leaf tips and *Curcuma* are applied to swellings (Perry 1980).

###### Reference.

Perry (1980).

#### 8. *Walsura* Roxb.

##### Walsura pinnata

Taxon classificationPlantaeORDOFAMILIA

Hassk. (= W. elata Pierre)

###### Name.

**English**: heynea.

###### Range.

Myanmar, Thailand, Vietnam, South China, Taiwan, Indo-China, Peninsular Malaysia, Sumatra, Java, Borneo, Philippines, Moluccas, and New Guinea.

###### Uses.

*Bark*: Part of a compound decoction for diarrhea and dysentery.

###### Note.

The bark (rich in tannin) is astringent (Perry 1980).

###### Reference.

Perry (1980).

#### 9. *Xylocarpus* (Lam.) M.Roem.

##### Xylocarpus granatum

Taxon classificationPlantaeORDOFAMILIA

J.Koenig

###### Name.

**English**: cannonball mangrove.

###### Range.

Mangrove forests. China, India, Indonesia, Malaysia, Papua New Guinea, Philippines, Sri Lanka, Thailand, Vietnam; East Africa; and West Pacific islands. In Myanmar found in Ayeyarwady, Rakhine, Taninthayi, and Yangon.

###### Conservation status.

Least Concern [LC] (IUCN 2017).

###### Uses.

*Whole plant*: Astringent. *Bark*: Remedy for dysentery. *Fruit* and *Seed*: An antidiarrheic; peels of fruits or seed used as poultice on swellings, and ash of seed applied to itches. *Bark* and *Root*: Strong astringent. *Root*: Used to treat cholera.

###### Notes.

The medicinal uses of this species in India are discussed in Jain and DeFilipps (1991) as follows: The bark is used as an astringent and febrifuge; also for diarrhea, dysentery, and abdominal problems. The fruit is used to treat elephantiasis and breast swelling; the seed kernel for a bitter tonic, and the seed (mixed with sulfur and coconut oil) in an ointment for itch.

###### Reference.

Perry (1980).

##### Xylocarpus moluccensis

Taxon classificationPlantaeORDOFAMILIA

(Lam.) M.Roem.

###### Names.

**Myanmar**: *kyana*, *kyat-nan*, *pinle-ohn*, *pinle-on*. **English**: puzzlenut tree.

###### Range.

Throughout most of Old World tropics to Australia, Fiji, and Tonga.

###### Conservation status.

Least Concern [LC] (IUCN 2017).

###### Uses.

*Whole plant*: Astringent. *Bark*: Remedy for dysentery. *Fruit* and *Seed*: An antidiarrheic; peels of fruits or seed used as poultice on swellings, and ash of seed applied to itch. *Bark* and *Root*: Strong astringent. *Root*: Used to treat cholera.

###### Note.

Perry (1980) discusses the uses of this species from Myanmar to the Philippines.

###### Reference.

Perry (1980).

### Menispermaceae (Moonseed family)

#### 1. *Cissampelos* L.

##### Cissampelos pareira

Taxon classificationPlantaeORDOFAMILIA

L.

###### Names.

**Myanmar**: *kywet-nabaung*. **English**: false pareira brava, velvet leaf.

###### Range.

Pantropic, especially India and Pakistan. In Myanmar, found in Chin, Kachin, Sagaing, and Taninthayi.

###### Uses.

*Whole plant*: A paste is made and applied locally to treat inflammatory conditions of the eye. *Leaf*: Used for cooling. *Root*: used as a febrifuge, diuretic, tonic, stomachic, and in prolapsus uteri.

###### Notes.

In Indo-China a decoction of the roots is used for colic and blennorrhea; in the Philippines leaves are antiscabious, also applied to snakebites; a decoction of the roots is diuretic, lithontriptic, pectoral, febrifuge, diaphoretic, emmanagogue, tonic, and sedative; roots are chewed and juice swallowed for abdominal pains and dysentery (Perry 1980). The medicinal uses of this species in India are discussed in Jain and DeFilipps (1991). Medicinal uses of the species in China are discussed in Duke and Ayensu (1985).

The chemical composition of the species includes alkaloids, hayatine, hayatinine, quecitol, and sterol Perry (1980).

###### References.

Nordal (1963), Perry (1980).

#### 2. *Tinospora* Miers

##### Tinospora cordifolia

Taxon classificationPlantaeORDOFAMILIA

(Willd.) Miers.

###### Names.

**Myanmar**: *hsin-doan manwai*, *sindon-ma-nwe*. **English**: heart-leaved moonseed.

###### Range.

Tropical Asia. Naturalized and cultivated throughout tropical and subtropical regions of Pakistan, India, Myanmar, and Sri Lanka. Found growing naturally throughout Myanmar in damp forests and on hills.

###### Uses.

*Whole plant*: Hot, spicy, bitter, and astringent in taste, the five parts (root, stem, leaf, flower and fruit) are known for promoting strength and longevity, “calming the blood”, stimulating appetite, promoting digestion, and controlling fevers, sores, and urinary disorders. A decoction reduced to one-third the starting volume is taken to neutralize poisons. The plant can be mixed and boiled together with *myin-hkwar* (*Centella asiatica*) leaves to alleviate heart palpitations and anxiety. Thin slices of the plant are eaten frequently to stop vomiting of blood; a decoction can be reduced to one-fourth its starting volume is used to ease chronic joint inflammation; plant also used in making medicines to treat gas and bile problems, urinary tract infections, menstrual disorders, earaches, and phlegm imbalances. *Stem*, *Leaf*: Used as stomachic and cholagogue. *Leaf*: Juice from crushed leaves is slightly warmed and used as an ear wash to alleviate earaches. A mixture of the leaves with equal parts of *lauk thay* (*Desmodium triquetrum*), *ohn hnwai* (*Aerva javanica*), *thinbaw maizali* (*Senna alata*), and *kone hti-kayone* (*Mimosa pudica*) leaves is made into a tea to promote longevity and prevent illnesses.

###### Note.

The medicinal uses of this species in India are discussed in Jain and DeFilipps (1991).

###### References.

Nordal (1963), Agricultural Corporation (1980), Forest Department (1999).

### Moraceae (Fig. family)

#### 1. *Antiaris* Lesch.

##### Antiaris toxicaria

Taxon classificationPlantaeORDOFAMILIA

Lesch.

###### Names.

**Myanmar**: *hmya-seik*, *hkang-awng*, *aseik*. **English**: upas tree.

###### Range.

Tropical Africa, Madagascar, tropical Asia to Philippine Islands and Fiji. In Myanmar, found in Bago, Chin, Mandalay, Mon, Sagaing, and Yangon.

###### Uses.

*Latex*: Used as a heart tonic and febrifuge; also as an arrow poison. *Seed*: Has good febrifuge and antidysenteric properties (these good uses have also been mentioned for the leaves and bark).

###### Notes.

In India the seed is used for dysentery and as a febrifuge (Jain and DeFilipps 1991). A tribe in Borneo uses the latex in decoction as a febrifuge; they also apply it to festering wounds and snakebites (Perry 1980). The leaves and bark are said to have good febrifuge and antidysenteric properties; also the seed (Perry 1980).

Reported chemical constituents of this species include a toxic glycoside; alpha-, beta-, gamma-antiarin; antiarol; and fats (Perry 1980). Throughout the East, the *toxic* sap (latex) from this species is known for its use as an arrow or dart poison, and much has been written about it. It proves fatal, however, only when it reaches the bloodstream, and can be taken into the mouth without any ill effects (Perry 1980). The juice, *in very small quantity*, is a mild circulatory and cardiac stimulant, but in large doses it acts as a myocardial *poison*; and has a strong digitalis-like action (Perry 1980).

###### References.

Nordal (1963), Perry (1980).

#### 2. *Artocarpus* J.R.Forst. & G.Forst.

##### Artocarpus heterophyllus

Taxon classificationPlantaeORDOFAMILIA

Lam.

###### Names.

**Myanmar**: *mak-lang*, *mung-dung*, *ndung*, *pa-noh*, *panwe*, *peinne*. **English**: jackfruit.

###### Range.

India. Cultivated in Myanmar.

###### Uses.

*Bark*: Employed as poultice to treat ulcers and abscesses. *Sap*: Utilized for same purposes as the bark. *Seed*: Used to treat indigestion. *Root*: Used to treat diarrhea, and in a compound extract for fever.

###### Notes.

In India the leaf is fried with the leaves of *Emblica* and *Azadirachta*, mixed with mustard oil and applied on sores, smallpox, carbuncles, and used as an anthelmintic; the flower us employed during childbirth to clear the fetus (Jain and DeFilipps 1991). In China latex from the stem is used for abscesses and ulcers; the bark is employed as a gargle; the leaf is used for diarrhea; and the ash made from the root is used for diarrhea and worms, and is also taken after childbirth (Duke and Ayensu 1985). The fruit pulp and seeds are considered cooling, tonic, and pectoral. In Indo-China the wood is used as a sedative to treat convulsions, boiled leaves are given to both animals and women to activate the secretion of milk, and the sap is considered antisyphilitic and a vermifuge. On the Malay Peninsula and in the Philippines, the ashes of the leaves, with or without oil, are applied to treat ulcers and wounds (Perry 1980).

The latex contains caoutchouc, resin, and cerotic acid (Duke and Ayensu 1985). The wood contains a yellow pigment, morin, cyanomaclurin; the bark has tannin; cerotic acid is found in the latex; and the fruit an pulp have sugar, protein, fiber, and ash (Perry 1980). Chemical constituents, pharmacological action and medicinal uses of this species in Indian Ayurveda are discussed in detail by Kapoor (1990).

Data on the propagation, seed treatment, and agricultural management of this plant are given in Katende et al. (1995).

###### References.

Perry (1980), Forest Department (1999).

##### Artocarpus lakoocha

Taxon classificationPlantaeORDOFAMILIA

Wall. ex Roxb.

###### Names.

**Myanmar**: *mai-mak-hat*, *mayauklok-ni*, *meik-mahot*, *myauk-laung*, *myauk-lok*. **English**: monkey-jack.

###### Range.

India to Myanmar. Cultivated for edible fruit. In Myanmar, found in Chin, Mandalay, Taninthayi, and Yangon.

###### Uses.

*Juice* and *Seed*: Used as a purgative. *Bark*: An astringent.

###### Notes.

In India the bark and exudation are used externally for spleen complaints; the seed is used as a purgative (Jain and DeFilipps 1991). In Indo-China the root is employed as a tonic and deobstruent, and the leaves are used in treating dropsy (Perry 1980).

The stem yields two triterpenes, B-amyrin acetate and lupeol acetate (Perry 1980).

###### Reference.

Perry (1980).

#### 3. *Ficus* L.

##### Ficus benjamina

Taxon classificationPlantaeORDOFAMILIA

L.

###### Names.

**Myanmar**: *kyet-kadut*, *nyaung-lun*, *nyaung-thabye*. **English**: Benjamin tree, Java flower, laurel, small-leaved rubber plant, tropical laurel, weeping laurel.

###### Range.

India, southeastern Asia, the Malay Archipelago, and northern tropical Australia. In Myanmar, found in Rakhine and Yangon.

###### Use.

*Leaf*: Applied to ulcers.

###### Notes.

In India the milky juice of the plant is used to treat whitening of the cornea of the eye; a decoction of the leaf, mixed with oil, is applied externally to ulcers (Jain and DeFilipps 1991). In Indo-China the latex is mixed with alcohol and prescribed for shock, and the pounded roots are applied to poison arrow wounds (Perry 1980).

Cerotic acid has been found in the milky sap (Perry 1980).

###### Reference.

Perry (1980).

##### Ficus hispida

Taxon classificationPlantaeORDOFAMILIA

L.f.

###### Names.

**Myanmar**: *ka-aung*, *kadut*. **English**: country fig, hairy fig.

###### Range.

Tropical Asia from India to northern Australia. In Myanmar, found in Bago, Mandalay, Taninthayi, and Yangon.

###### Uses.

Used to treat diabetes (plant part not given in Nordal 1963). *Fruit*: Used in poultices.

###### Notes.

In India the bark, fruit, and seed are employed as an emetic and purgative (Jain and DeFilipps 1991). In China latex from the stem is used for diarrhea, dysuria, and applied to cracks in the soles of the feet; the fruit is applied to warts (with *Allium* and *Sesbania*) (Duke and Ayensu 1985). In Malaya a leaf decoction is used for fever and parturition and a bark decoction for stomachaches, pounded leaves are applied to boils and ulcerated noses; in Indonesia latex is used for diarrhea and dysuria, and bark and turmeric are mixed with rice water for eczema (Duke and Ayensu 1985). Ayurvedics use the plant for anemia, biliousness, blood disorders, dysentery, epistaxis, hemorrhoids, jaundice, stomatorrhagia, and ulcers; the fruit is used as an emetic, aphrodisiac, lactagogue, and tonic (Duke and Ayensu 1985). On the Malay Peninsula a decoction of the leaves is given as a protective medicine after childbirth and to treat fever, a decoction of the bark with that of several other plants is used as another remedy for fever, pounded leaves are applied to boils and (in a compound) to an ulcerated nose; in Indonesia the latex is ingested to treat diarrhea and painful urination and externally applied to cracks in the soles of the feet, fruit mixed with red onions and *Sesbania* leaves is used on warts, and a mixture made from the bark and *Curcuma* ground together with water from red rice is applied to pustulous eczema (Perry 1980).

The bark contains tannin, wax, a caoutchouc, and a glucoside principle; the latex contains an alcohol extract and a chloroform extract (Duke and Ayensu 1985).

###### References.

Nordal (1963), Perry (1980), Duke and Ayensu 1985.

##### Ficus religiosa

Taxon classificationPlantaeORDOFAMILIA

L.

###### Names.

**Myanmar**: *nyaung bokdahae*, *bodhi nyaung*, *lagat* (Kachin), *mai-nyawng* (Shan), *nyaung-bawdi*. **English**: bo tree, sacred fig tree.

###### Range.

Tropical Asia. Grows naturally throughout Myanmar; also cultivated there.

###### Uses.

*Whole plant*: Bitter and astringent in taste with cooling properties, drying, and difficult to digest; the bark, roots, fruits, leaves, and sap are known for bringing out brilliance in complexion, cleansing the uterus, and controlling bile and phlegm as well as alleviating heat-induced illnesses, sores, asthma, leprosy, plague, and fistulas. *Sap*: Used to treat female-related disorders. *Bark*: Considered binding, promotes weight gain. A decoction of bark- reduced to one-half the starting volume is taken for many skin problems, rashes, and itching; also used as a mouthwash to cure tooth diseases. Dried and powdered inner bark is applied to fistulae to stimulate healing and new tissue formation. Ash from the bark is sprinkled onto genital sores caused by venereal diseases to promote drying and healing; ash from young bark filtered through fine cloth is rubbed on chronic sores to expedite healing. Bark is also used in medicines to treat burns, breast problems, lock-jaw, and snakebites in animals. *Sap*: Used to alleviate toothaches and gum pain. *Sap* and *Leaf*: An anti-emetic. Used to cleanse the blood; also used in preparations to treat boils in the groin, hemorrhaging, and cracked tongues and lips. A decoction of the leaves with jaggery is taken for fatigue to promote strength and well-being. A mixture of the juice from the crushed leaves and the sap is applied topically to treat cracks in the feet. *Fruit*: The ripe fruit, which has cooling properties, is considered beneficial for the heart. It is used to treat blood diseases, “heat” or bile conditions, nausea, lung infections, and loss of appetite. A mixture of the crushed dried fruit and water is taken for asthma and bronchitis. *Root*: The root bark is stewed in water, reduced to one-half the starting volume, and given for herpes infections. The roots are ground to form a paste applied topically as a remedy for leprosy and other sores. A root decoction with rock salt is taken to alleviate asthma and congestion. A mixture of the root powder and ginger powder is given for diseases involving gas, asthma, coughing, nausea; also to treat elephantiasis.

###### Notes.

The medicinal uses of this species in India are discussed in Jain and DeFilipps (1991). Medicinal uses of this species in China are discussed in Duke and Ayensu (1985).

###### References.

Nordal (1963), Agricultural Corporation (1980).

##### Ficus retusa

Taxon classificationPlantaeORDOFAMILIA

L.

###### Names.

**Myanmar**: *nyaung-ok*. **English**: Chinese banyan, Indian laurel, Malay banyan, Malay laurel.

###### Range.

Malay Peninsula to Borneo. Widely distributed in Myanmar.

###### Use.

*Leaf* and *Root*: Used to treat wounds.

###### Notes.

In China “The fruits, in liquor, are both internally and externally an anodyne to treat contusions; the boiled leaves and buds are a treatment for conjunctivitis…”; the aerial roots are part of a lotion rubbed on rheumatic parts and swollen feet; and the ashes (after burning in bamboo) are used as an application for toothache (Perry 1980). In Taiwan the bark and aerial roots are used to treat tuberculosis and to reduce fever (Perry 1980).

###### Reference.

Perry (1980).

##### Ficus rumphii

Taxon classificationPlantaeORDOFAMILIA

Blume

###### Names.

**Myanmar**: *nyaung-phyu*. **English**: Rumphf’s fig tree.

###### Range.

China, Pakistan, Bhutan, India, Bangladesh, Indonesia, Malaysia, Myanmar, Nepal, Sikkim, Thailand, and Vietnam. In Myanmar found in Bago, Rakhine, and Yangon.

###### Use.

*Fruit*: Used to reduce fever.

###### Notes.

The medicinal uses of this species in India are discussed in Jain and DeFilipps (1991) as follows: Juice form the whole plant is used to kill worms; it also is taken internally with turmeric, pepper and ghee to treat asthma. Bark is used for snakebite. Medicinal uses of this species in Indonesia are discussed in Perry (1980).

###### Reference.

Nordal (1963).

##### Ficus semicordata

Taxon classificationPlantaeORDOFAMILIA

Buch.-Ham. ex Sm. (= F. cunia Buch.-Ham. ex Roxb.)

###### Names.

**Myanmar**: *kyet-kadut*, *ka-dut*, *lamai*, *mai-hpang*, *mai-lusang*, *tha-dut*, *ye-ka-on*. **English**: drooping fig.

###### Range.

Tropical Asia. In Myanmar found in Bago, Kachin, and Yangon.

###### Use.

*Fruit*: Used in aphtous complaints.

###### Note.

In India the bark and fruit are made into a bath for the treatment and cure of leprosy; the fruit is used for aphthous complaints; and juice from the root is used for bladder maladies, juice also boiled in milk for visceral disorders (Jain and DeFilipps 1991).

###### Reference.

Nordal (1963).

#### 4. *Streblus* Lour.

##### Streblus asper

Taxon classificationPlantaeORDOFAMILIA

Lour.

###### Names.

**Myanmar**: *hkajang-nai*, *mai-hkwai*, *okhne*. **English**: Siamese rough bush.

###### Range.

China, Bhutan, Cambodia, India, Indonesia, Laos, Malaysia, Nepal, Philippines, Sikkim, Sri Lanka, Thailand, and Vietnam. In Myanmar, found in Bago, Sagaing, and Taninthayi.

###### Uses.

*Bark*: Used as a remedy to treat diarrhea. *Leaf*: Decoction of the dried leaves administered for dysentery. *Root*: Used to treat ulcers.

###### Notes.

The medicinal uses of this species in India are discussed in Jain and DeFilipps (1991) as follows: The latex is employed for pneumonia, as astringent and antiseptic for curing sore heels, swellings, applied on temples as a sedative for neuralgia; the bark is used for diarrhea, slow pulse, gravel (with two other species), other urinary diseases, colic, menorrhagia, cholera (with one other species), and dysentery; the stem is used for toothache; the leaf as a galactagogue, poutice for swellings, and for eye diseases; the seed is used for piles, diarrhea, epistaxia, and locally on leucoderma; the root is used on ulcers, boils, and swellings, and for dysentery. Perry (1980) discusses the medicinal uses of this species in Thailand, Indo-China, Indonesia, and the Philippines.

The bark “contains a bitter material resembling the poisonous principle of *Antiaris toxicaria*, but the leaves are not poisonous”; also, the latex contains considerable resin and a little rubber (Perry 1980).

###### Reference.

Perry (1980).

### Moringaceae (Horseradish Tree family)

#### 1. *Moringa* Adans.

##### Moringa oleifera

Taxon classificationPlantaeORDOFAMILIA

Lam.

###### Names.

**Myanmar**: *dan-da-lun*, *sort-htmaine* (Mon). **English**: Ben nut, drumstick tree, horseradish tree.

###### Range.

India. Widely cultivated and naturalized in the tropics. Found throughout Myanmar. Also, cultivated there as a vegetable.

###### Uses.

*Sap*: Held in mouth to treat tooth decay. *Bark*: Slightly sweet and efficacious, stimulates the palate and is good for digestion. Used as an astringent. Freshly obtained liquid applied in the ear to treat earaches and ear infections. *Bark* and *Leaves*: Used as a heart stimulant. *Leaf*: Made into a soup with garlic, galangal (*Alpina galanga* or *A. officinarum*), and *meik-thalin* (*Zingiber cassumnar*) for arrested menstruation. When boiled in water down to a third of the original volume, and taken as a soup, will bring down high blood pressure. *Root*: Crushed, then 1 tablespoon of the liquid taken to treat laryngitis and sore throat; crushed and mustard seed added in equal amounts, soaked in water, and taken three times a day for indigestion and bloated stomach; boiled in water down to a third and tablespoon taken daily to treat cancer of the stomach. *Root*: Crushed into powder and combined with *paranawar* (*Boerhavia diffusa*) root powder in equal amounts, cooked with coconut milk and honey, and one tablespoon taken in morning and evening as a tonic to give strength and longevity; crushed and used as a poultice for inflammation; and liquid from crushed root taken with milk to treat diabetes. *Flower*: Used in making medicines to treat edema, dropsy, boils, sores, and gas. *Fruit*: Cooked and given to children to keep them free of round and thread worms; made into a powder and combined with sugar to treat excessive urination. *Seed*: Used to cure headaches and for poisoning. Also, made into a powder and applied to the ear to cure earaches and infections. Oil from the seed is used in treating sores, rashes, and itches.

###### Notes.

The medicinal uses of this species in India are described in Jain and DeFilipps (1991). Indigenous medicinal uses of this species in the Andaman and Nicobar Islands (India) are described by Dagar and Singh (1999).

Chemical constituents, pharmacological action, and medicinal use of this species in Indian Ayurveda are discussed in detail by Kapoor (1990). The medicinal uses of *Moringa oleifera* in the Caribbean region, as well as its chemistry, biological activity, toxicity and dosages, are discussed by Germosén-Robineau (1997). A pharmacognostical profile, including medicinal uses of this plant in Africa, is given in Iwu (1993). The chemical constituents, pharmacological activities, and traditional medicinal uses of *M. oleifera* on a worldwide basis are discussed by Ross (1999). The toxic properties, symptoms, treatment and beneficial uses of this plant, parts of which are poisonous, are discussed by Nellis (1997).

Data on the propagation, seed treatment, and agricultural management of this species are given by Katende et al. (1995) and Bekele-Tesemma (1993). Details of the active chemical compounds, effects, herbal usage, and pharmacological literature of this plant are given in Fleming (2000).

###### References.

Nordal (1963), Agricultural Corporation (1980).

### Myristicaceae (Nutmeg family)

#### 1. *Myristica* Gronov.

##### Myristica fragrans

Taxon classificationPlantaeORDOFAMILIA

Houtt.

###### Names.

**Myanmar**: *zar-date-hpo*, *zar-pwint*. **English**: mace, nutmeg.

###### Range.

East Indies. A cultivar that thrives in Tanintharyi Division, Myeik and Mawlamyaing townships; likes hot and humid climates; prefers ravines close to coastal areas.

###### Conservation status.

Data Deficient [DD] (IUCN 2017).

###### Uses.

*Myristica fragrans* has an astringent, bitter, and hot taste. It is used in preparations for semen control and hemorrhoid relief, and also considered an important component of thway-hsay (literally means “blood medicine”), the traditional blood purification mixture, as well as tonics and medicines for male and female maladies. Unspecified plant parts are taken orally with warm water and sugar for blood purification, indigestion, insomnia, and tumors; with warm water alone, the mixture is used for gas, colic, diarrhea, and menstrual disorders. *Oil*: Easily digestible and fragrant, nutmeg oil stimulates appetite, increases strength, and controls fevers. *M. fragrans* is combined with *tha-na-kha* (*Limonia acidissima)*, *taungtan-gyi* (*Premna integrifolia*), and turpentine oil for external use in the treatment of tumors. *Fruit*: Given as a remedy for chronic diarrhea, digestive problems, spleen inflammation, and gas pain. *Seed*: A paste of ground seeds and honey is eaten to strengthen a weak heart and alleviate male-related dysentery. The paste made with cold water is eaten, licked, or applied all over the body to cure cholera; it is applied to the outer ear to relieve inflammation, and licked to overcome nausea. Seed paste applied topically clears pimples.

###### Notes.

Medicinal uses of this species in India are discussed in Jain and DeFilipps (1991). Chemical constituents, pharmacological action, and medicinal use of this species in Indian Ayurveda are discussed in detail by Kapoor (1990). Indigenous medicinal uses of this species in the Andaman and Nicobar Islands (India) are described by Dagar and Singh (1999). Medicinal uses of this species in China are discussed by Duke and Ayensu (1985).

The medicinal uses of this plant in the Caribbean region, as well as its chemistry, biological activity, toxicity and dosages, are discussed by Germosén-Robineau (1997).

Traditional medicinal uses, chemical constituents and pharmacological activity of this species are discussed by Ross (2001). A pharmacognostical profile including medicinal uses of this plant in Africa is given in Iwu (1993). Details of the active chemical compounds, effects, herbal usage, and pharmacological literature of this plant are noted in Fleming (2000). Worldwide medicinal usage, chemical composition and toxicity of this species are discussed by Duke (1986).

Nutmeg contains myristicin, a hallucinogenic substance that is dangerous when ingested in large amounts (fewer than three seeds). One product of the fruits and flowers of *Myristica fragrans* is nutmeg oil, which causes convulsions after being ingested and has hypnotic activity from the chemical isolemicin; fruits and leaves also contain the reputedly psychotomimetic compound myristicin, borneol which affects the central nervous system, and the low grade hepatocarcinogen known as safrole (Lan et al. 1998). The grated or powdered seed is the source of nutmeg, and the aril provides the source of mace.

###### References.

Agricultural Corporation (1980), Ministry of Health (2001).

### Myrtaceae (Clove family)

#### 1. *Eucalyptus* L’Her.

##### Eucalyptus globulus

Taxon classificationPlantaeORDOFAMILIA

Labill.

###### Names.

**Myanmar**: *hnget-chauk*. **English**: Australian fever tree, blue gum, southernblue gum, Tasmanian blue gum.

###### Range.

Tasmania, Australia. Grows as a cultivar in Myanmar’s temperate zone, but can also be cultivated throughout the country.

###### Uses.

Sharp and hot in taste, the leaves, oil, sap, and roots are used in medicinal preparations. *Sap*: Given as a cure for asthma, to relieve constipation, to control bloating and flatulence, and to clear the brain. *Leaf*: For bacterial skin infections, impetigo and erysipelas, the juice is applied topically, or the leaves are used as a poultice. The oil is also used for skin sores and infections; mixed with equal amounts of olive oil, it is applied topically to relieve inflamed or aching joints. Made into an ointment, it is used to treat burns and as a rub for asthma. Vapors from a decoction of the leaves are inhaled to relax and open airways constricted during asthma attacks. The leaves are used to treat bronchitis, fever, poisoning, whooping cough, and surgical wounds. They are also boiled to create a steam bath used as a remedy for colds and headaches. *Root*: Used to make laxatives.

###### Notes.

Medicinal uses of this species in India are discussed in Jain and DeFilipps (1991). Medicinal uses of this species in China are discussed by Duke and Ayensu (1985).

A pharmacological profile including medicinal uses of this plant in Africa is given in Iwu (1993). The medicinal uses of this plant in the Caribbean region, as well as its chemistry, biological activity, toxicity and dosages, are discussed by Germosén-Robineau (1997). Details of the active chemical compounds, effects, herbal usage, and pharmacological literature of this plant are given in Fleming (2000). Traditional medicinal uses, chemical constituents, and pharmacological activity of this species are discussed by Ross (2001). Worldwide medicinal usage, chemical composition, and toxicity of this species are discussed by Duke (1986).

###### Reference.

Agricultural Corporation (1980).

#### 2. *Melaleuca* L.

##### Melaleuca cajuputi

Taxon classificationPlantaeORDOFAMILIA

Powell

###### Name.

**English**: cajeput.

###### Range.

Cultivated in China, Taiwan, Indonesia, Malaysia, Thailand, and Vietnam. Reported from Myanmar.

###### Uses.

*Oil*: Combined with camphor and considered beneficial for gout; internally, considered to be a diffusible stimulant quickening the heart action.

###### Notes.

In China the species is used as a disinfectant; in Indo-China it is used in an embrocation for rheumatism and joint pain, as a local analgesic, and the oil may be inhaled for rhinitis and colds, also used in surgery; in Cambodia “the leaves of a special variety are used in an infusion to treat dropsy”; on the Malay Peninsula a minute portion of the oil is dropped on sugar to treat colic and cholera, and is also a fragrant stomachic and an anodyne (Perry 1980). In Indonesia it is used externally to treat colic, headache, toothache, earache, leg cramps, various types of pains, skin disease, fresh wounds, and burns; internally, a small dose serves as a diaphoretic, an antispasmodic, and a stimulant; softened bark is used to ripen abscesses and draw out pus; the fruit is used with leaves of *Baechkea frutesces* to treat stomach problems (Perry 1980). In the Philippines the leaves are used to treat asthma; in New Guinea the oil is rubbed on the body for malaria (Perry 1980).

Reported constituents include cajuputol (“identical with eucalyptol or cineole”), terpenol, 1-pinene, and aldehydes (Perry 1980).

###### Reference.

Perry (1980).

#### 3. *Psidium* L.

##### Psidium guajava

Taxon classificationPlantaeORDOFAMILIA

L.

###### Names.

**Myanmar**: *malaka*, *mankala*. **English**: guava.

###### Range.

New World tropics.

###### Use.

*Leaf* and *Fruit*: Used in the treatment of diabetes.

###### Notes.

Medicinal uses of this species in India are discussed in Jain and DeFilipps (1991). Indigenous medicinal uses of this species in the Andaman and Nicobar Islands (India) are described by Dagar and Singh (1999). Medicinal uses of this species in China are discussed by Duke and Ayensu (1985).

The medicinal uses of this plant in the Caribbean region, as well as its chemistry, biological activity, toxicity and dosages, are discussed by Germosén-Robineau (1997). The chemistry, pharmacology, history, and medicinal uses of this species in Latin America are discussed in detail by Gupta (1995).

The chemical constituents, pharmacological activities, and traditional medicinal uses of this plant on a worldwide basis are discussed in detail by Ross (1999). A pharmacognostical profile including medicinal uses of this plant in Africa is given in Iwu (1993). Data on the propagation, seed treatment, and agricultural management of this species are given by Katende et al. (1995) and Bekele-Tesemma (1993).

Uses of this plant in the Upper Amazon region, including preparations of the flowers for helping to regulate menstrual periods, are given by Castner et al. (1998). Mors et al. (2000) note that studies of the flavonoid components of leaf extracts of this species on guinea pig ileum demonstrated an inhibition of contractions, which may explain the antidiarrheic activity of this species.

###### Reference.

Nordal (1963).

#### 4. *Syzygium* P.Browne ex Gaertn.

##### Syzygium aromaticum

Taxon classificationPlantaeORDOFAMILIA

(L.) Merr. & L.M. Perry (= Eugenia caryophyllata Thunb.)

###### Names.

**Myanmar**: *lay-hnyin*. **English**: clove, clove tree.

###### Range.

The Moluccas. Widely cultivated in warm regions. Cultivated in Myanmar.

###### Uses.

*Flower*: Buds (cloves sun-dried buds) are sharp, spicy and bitter in taste; regarded as having the following properties: carminative, stomachic, antiemetic, antinauseant, febrifuge, vermifuge, emmenagogue, and tonic. They are used as an aid in treating diseases of the arteries, for lung problems, and as a general stimulant and excitant of the digestive functions.

Paste made from cloves is mixed with rock sugar syrup and licked to cure morning sickness. Cloves are crushed together with *hsay-kha gyi* (*Andrographis paniculata*) and taken with hot water to treat fevers and fatigue; a mixture of crushed cloves together with honey is used as eyedrops for sore eyes and cataracts; they can be crushed with water, warmed and taken for nausea, dry mouth, and loss of taste. Cloves taken together with sour pomegranate juice are used to treat vomiting during an epileptic fit as well as ordinary vomiting. An ointment for sores, such as boils, pimples, or rashes that neither erupt nor subside, is made by mixing cloves with equal amounts of turmeric powder and crushing them together. They are roasted, crushed and mixed with honey and licked to treat whooping cough. Clove oil, or a paste, is used for toothaches. The oil mixed with mustard oil is used as a rub for aching joints and can also be rubbed onto the forehead for headaches.

###### Notes.

The medicinal uses of this species in India are discussed in Jain and DeFilipps (1991). Medicinal uses of this species in China are discussed in Duke and Ayensu (1985). Perry (1980) notes that medicinal uses of the species “are very much in common throughout the various geographic regions” and lists some of these uses.

###### References.

Agricultural Corporation (1980), Perry (1980).

##### Syzygium cumini

Taxon classificationPlantaeORDOFAMILIA

(L.) Skeels (= Eugenia jambolana Lam.)

###### Names.

**Myanmar**: *tame*, *thabye-kyet-chi*, *thabye-phyu*, *wa-passan*. **English**: black plum, jambolan plum, jambu, Java plum.

###### Range.

India and Sri Lanka, east to Malay Archipelago. Cultivated in tropical regions. In Myanmar, found in Bago, Kachin, Magway, Mandalay, and Yangon.

###### Uses.

*Bark*: Astringent and sweet with binding properties, easily digestible. Used in the compounding of medicines to treat conditions with white vaginal discharge or discharge due to venereal disease. A paste of the bark made with milk is mixed with some honey and a tablespoon is taken to cure severe diarrhea. *Bark*, *Leaf*, *Fruit* and/or *Seed*: Used to treat diarrhea and dysentery. *Bark* and *Seed*: Used in treating diabetes. *Shoot*: Has cooling, drying, and binding properties; used for indigestion and bloating. *Leaf*: A decoction is used for sore eyes. Fresh tender leaves are crushed with water and held in the mouth to cure gum boils and other mouth sores; crushed and taken with milk to treat bleeding hemorrhoids; and crushed with water and taken to neutralize the effects of opium. Juice from the leaves is applied to scorpion stings. *Fruit*: Sweet and astringent, it diminishes indigestion. Juice of crushed fruit can be taken, once in the morning and once at night, for inflammation of the spleen. Juice of ripe fruit is squeezed, strained, and fermented, then taken as a treatment for gas. Eating the ripe fruit is used as a treatment for diabetes. *Seed*: Made into a powder and taken with cooled boiled water to treat (mild) diabetes mellitus; paste made from the dried seeds applied 2–3 times a day to sores associated with venereal disease; and paste, also made with water, applied to sores that are difficult to heal. Together with mango seeds and *krazu* (*Terminalia citrina*) seeds can be mixed in equal amounts, roasted and made into a powder taken to cure diarrhea.

###### Notes.

The medicinal uses of this species in India are discussed in Jain and DeFilipps (1991). Perry (1980) discusses the medicinal uses of the species in India, Myanmar, Indo-China, the Malay Peninsula, and the Philippines.

Reported constituents include gallic acid, tannin, volatile oil, fat, antimellin, jambuol, olein, linolein, palmitin, sterarin, phytosterin, myricyl alcohol, and hentriacontane (Perry 1980). It is thought that ellagic and gallic acid, and tannin “may be responsible for the medicinal value of the seeds”; also, the leaves have been found to have a slightly antibiotic action against *Staphylococcus* (Perry 1980).

###### References.

Agricultural Corporation (1980), Perry (1980).

##### Syzygium jambos

Taxon classificationPlantaeORDOFAMILIA

(L.) Alston (= Eugenia jambos L.)

###### Names.

**Myanmar**: *hnin-thi-pin*, *thabyu-thabye*, *thabyu-thaby*, *wa-pasang* (Kachin), *tame* (Kayin), *sot-crin* (Chin), *mak-spye* (Shan). **English**: rose apple.

###### Range.

Indo-Malaysian region. Cultivated in Myanmar.

###### Uses.

*Bark* and *Seed*: Used in treating diabetes. *Leaf*: A decoction is used to treat sore eyes.

###### Note.

In India the bark is employed for rheumatism and pneumonia; the leaf as a decoction for eye sores; the fruit for liver problems (Jain and DeFilipps 1991).

###### References.

Nordal (1963), Perry (1980).

##### Syzygium nervosum

Taxon classificationPlantaeORDOFAMILIA

A.Cunn. ex DC. (= Eugenia operculata Roxb.; Cleistocalyx operculatus (Roxb.) Merr. & L.M. Perry)

###### Names.

**Myanmar**: *kon-thabye*, *thabye-shin*, *ye-thabye*. **English**: *rai jamun* (Hindi).

###### Range.

From China south throughout Southeast Asia, and northern Australia. In Myanmar, found in Bago, Chin, Kachin, Rakhine, and Shan.

###### Uses.

*Leaf*: Used in fomentation. *Fruit*: Used to treat rheumatism. *Root*: Used in an embrocation.

###### Notes.

In India the bark is used for rheumatism and pneumonia; the leaf for rheumatism and dry fomentation; the fruit for rheumatism; and the root boiled and rubbed on joints (Jain and DeFilipps 1991). Perry (1980) discusses the uses of this species in China and Indo-China.

Chemical constituents of the plant include aromatic volatile oil, a little tannin, traces of methylchavicol, and alkaloid similar to caffeine (Perry 1980).

###### Reference.

Perry (1980).

### Nyctaginaceae (Bougainvillea family)

#### 1. *Boerhavia* L.

##### Boerhavia diffusa

Taxon classificationPlantaeORDOFAMILIA

L.

###### Names.

**Myanmar**: *pa-yan-na-war*. **English**: spreading hogweed.

###### Range.

Pantropical. In Myanmar grows naturally on plains throughout the country.

###### Uses.

*Whole plant*: Take with liquid from the leaves of *kyeik-hman* (*Eclipta prostrata*) to cure female-related disorders. Mix with the seeds of *dant-kywei* (*Senna tora*) and either eaten or used as an ointment to cure ringworm. *Leaf*: When mixed with milk, it will cure pain in passing urine, gonorrhea, asthma and fevers, give longevity, and keep a person strong and looking youthful. Eaten and cooked with *nga-gyin* fish (*Cirrhinus mrigala*) to cure partial paralysis. New mothers having difficulty in lactating will produce milk quickly by drinking soup to which the leaves have been added; sore and aching breasts and general weakness and fatigue will also be cured. Cooked or made into a soup mixed with *nga-panaw* fish (*Channa punctata*) to cure heart disease, pleurisy, typhoid, bloating, dropsy, hemorrhoids, flatulence, phlegm, and indigestion. Pounded and used as a poultice for external inflammations. *Root*: Eating powdered root with sugar will cure coughing and whooping cough; mixed with honey will cure asthma.

###### Notes.

The medicinal uses of this species in India are discussed in Jain and DeFilipps (1991) as follows: The whole plant us used as a laxative and diuretic; the leaf as an appetizer, alexiteric, and to control bleeding after childbirth; and the seed as a tonic, carminative and for lumbago, scabies, purifying the blood, and hastening delivery. The root is employed as a diuretic, laxative, expectorant, stomachic; for asthma, edema, anemia, jaundice, internal inflammation, anasarca, as an antidote to snake venom, for dropsy, gonorrhea, ulcers, guineaworm, abdominal tumors, and cancer; also in many herbal preparations for fever (decoction), and an antispasmodic for heart and kidney ailments. Medicinal uses of this species in Dominica are described in DeFilipps (1998).

Perry (1980) discusses the uses of the species in Indo-China, India, and Indonesia.

The plant contains an active alkaloid, punarnavine, and it is believed that a high content of potassium salts enhances the powerful diuretic action of the alkaloid (Perry 1980).

###### References.

Nordal (1963), Agricultural Corporation (1980), Forest Department (1999).

#### 2. *Bougainvillea* Comm. ex Juss.

##### Bougainvillea spectabilis

Taxon classificationPlantaeORDOFAMILIA

Willd.

###### Names.

**Myanmar**: *sekku-pan*. **English**: great bougainvillea.

###### Range.

Native of Brazil. Cultivated elsewhere.

###### Uses.

Plant used for medicinal purposes (exact uses not given in Nordal 1963).

###### Notes.

Traditional practitioners in Mandsaur use the leaves for a variety of disorders, including the treatment of diarrhea and to reduce stomach acidity; the species is used elsewhere as follows- for cough and sore throat, a decoction of dried flowers (in water) is used for blood vessels and leucorrhea, a decoction of dried stems (in water) is used for hepatitis (Edwin et al. 2007).

###### Reference.

Nordal (1963).

#### 3. *Commicarpus* Standl.

##### Commicarpus chinensis

Taxon classificationPlantaeORDOFAMILIA

(L.) Heimerl (= Boerhavia repanda Willd.)

###### Names.

**Myanmar**: *pa-yan-na-war*. **English**: diffuse hogweed, spreading hogweed.

###### Range.

China, India, Indonesia, Malaysia, Myanmar, Pakistan, Thailand, and Vietnam. Widely distributed in Myanmar.

###### Use.

*Root*: Used as galactagogue.

###### Note.

In Indonesia the crushed leaves of the species are smeared onto spots of scabies previously scoured open (Perry (1980).

###### Reference.

Nordal (1963).

#### 4. *Mirabilis* L.

##### Mirabilis jalapa

Taxon classificationPlantaeORDOFAMILIA

L.

###### Names.

**Myanmar**: *lay-naryi pan*, *myitzu pan pin*. **English**: four o’clock, marvel of Peru.

###### Range.

Tropical America. Cultivated in Myanmar.

###### Uses.

*Whole plant*: A decoction of the five parts mixed with sugar and reduced to one-third the starting volume given for urinary infections and bladder stones. *Leaf*: Known for promoting virility, leaves are also used to treat bumps and sores. The juice is applied to rashes to relieve itching. Leaves crushed with cold water are used as a poultice for broken and fractured bones, dislocations, and knotted muscles. *Root*: The tuber is used in medicines for impotence. Powdered tuber, dried ginger, pepper, and *peik-chin* (*Piper longum*) fruit are mixed with honey and licked for gonorrhea.

###### Notes.

The medicinal uses of this species in India are discussed in Jain and DeFilipps (1991). Medicinal uses of this species in China are discussed by Duke and Ayensu (1985). Perry (1980) discusses the medicinal uses of the species in China, Indo-China, and the Malay Peninsula.

The toxic properties, symptoms, treatment, and beneficial uses of this plant are discussed by Nellis (1997). The roots contain an alkaloid, and the *roots and seeds are poisonous* (Perry (1980).

###### Reference.

Agricultural Corporation (1980).

### Nymphaeaceae (Water-lily family)

#### 1. *Nymphaea* L.

##### Nymphaea rubra

Taxon classificationPlantaeORDOFAMILIA

Roxb. ex Andrews

###### Names.

**Myanmar**: *kya-ni*. **English**: Indian red water-lily.

###### Range.

Cambodia, India, Indonesia, Laos, Malaysia, Myanmar, Philippines, Sri Lanka, Thailand, and Vietnam. Common in warmer parts of Myanmar.

###### Conservation status.

Least Concern [LC] (IUCN 2017).

###### Uses.

*Flower*: Used as a blood purifier and febrifuge.

###### Notes.

The medicinal uses of this species in India are discussed in Jain and DeFilipps (1991) as follows: The flower is decocted and used for heart palpitations; the rootstock is powdered and used for piles, diarrhea, and dyspepsia. Perry (1980) discusses the uses of *Nymphaea* species in Indo-China and the Philippines.

###### Reference.

Nordal (1963).

### Olacaceae (Olax family)

#### 1. *Olax* L.

##### Olax scandens

Taxon classificationPlantaeORDOFAMILIA

Roxb.

###### Names.

**English**: olax, *dheniaani* (Ayurvedic), rimil-beer (Folk).

###### Range.

Sri Lanka, India, southeastern Asia, West Malesia.

###### Uses.

*Bark*: Used to treat fever.

###### Note.

The species is used as a laxative (Burkill 1966); also for anemia and fever (Duke 2009).

###### Reference.

Perry (1980).

### Oleaceae (Olive family)

#### 1. *Jasminum* L.

##### Jasminum humile

Taxon classificationPlantaeORDOFAMILIA

L.

###### Name.

**English**: yellow jasmine.

###### Range.

Himalayas of western China. In Myanmar, found in Shan.

###### Use.

*Root*: Used to treat skin diseases such as ringworm.

###### Note.

In India the milky juice from the whole plant is given to destroy the unhealthy lining-walls of chronic fistulas and sinuses; the flower is employed as an astringent and tonic for bowels and heart; and the root is used for ringworm (Jain and DeFilipps 1991).

###### Reference.

Nordal (1963).

##### Jasminum multiflorum

Taxon classificationPlantaeORDOFAMILIA

(Burm.f.) Andrews

###### Names.

**Myanmar**: *kadawn*, *kadawnla*, *sabe-hmwe-sok*, *tawsabe*. **English**: downy jasmine.

###### Range.

India. In Myanmar found in Chin, Kachin, Shan, and Yangon.

###### Uses.

*Leaf*: Used to treat ulcers. *Root*: Used for snakebite.

###### Notes.

In India a poultice is made from dried leaves soaked in water and placed on indolent ulcers to promote healing; the flower is used as an emetic (Jain and DeFilipps 1991). In Indonesia an infusion of the plant is employed to treat catarrh of the bladder and also used as a febrifuge (Perry 1980). The plant is known to have an astringent effect on the bowels; and is used to treat fever, dysentery, stomach- ache, stomach ulcers, and kidney stones (Perry 1980).

A tannin-like bitter principle has been found, and an amorphous substance “which seems to be an alkaloid” has been isolated (Perry 1980).

###### Reference.

Perry (1980).

#### 2. *Nyctanthes* L.

##### Nyctanthes arbor-tristis

Taxon classificationPlantaeORDOFAMILIA

L.

###### Names.

**Myanmar**: *hseik hpalu*, *seik-palu*. **English**: coral jasmine, night jasmine.

###### Range.

Asia; cultivated in many places. Plant found throughout Myanmar; cultivated as an ornamental plant.

###### Uses.

*Whole plant*: The bark, five parts, flowers, and leaves are used in preparations that stimulate weight gain, promote fetal growth, inhibit hemorrhoid formation, alleviate female disorders, prevent hair loss, and reduce fevers. The boiled five parts are used for spleen problems. *Bark*: In particular, used in medicines to treat eye problems, bronchitis, fever, and skin disorders. *Flower*: Boiled and taken together with the liquid from boiling to alleviate joint inflammation. *Leaf*: Juice from the crushed leaves- taken with honey or sugar for gall bladder problems and chronic fevers; with a bit of salt, used as a de-worming medicine; with a bit of fresh ginger, taken as a malaria cure; ingested to neutralize venom from snakebite; also used to relieve diarrhea and loose bowels in infants. After cooling, water from briefly boiling the leaves is given to infants for fever and sickness. For muscle strain in the buttocks, leaves are simmered over low heat in water and ingested. A preparation of the leaves crushed together with black pepper is taken to relieve excessive menstruation. A reduction of the leaves boiled in water to half the starting volume is taken for excessive urination. Topically the crushed leaves- are applied to treat ringworm; together with milk, applied to relieve itching and rashes.

###### Notes.

The medicinal uses of this species in India are discussed in Jain and DeFilipps (1991). Perry (1980) also discusses the uses of the species, and notes that it is “much used in medicine of India”.

The bitter leaves contain tannic acid and methyl salicylate; the later may be an active agent against rheumatism (Perry 1980).

###### References.

Nordal (1963), Agricultural Corporation (1980).

### Orobanchaceae (Broom-rape family)

#### 1. *Aeginetia* L.

##### Aeginetia indica

Taxon classificationPlantaeORDOFAMILIA

L.

###### Names.

**Myanmar**: *kauk-hlaing-ti*. **English**: aeginetia.

###### Range.

From Taiwan to the Philippines. Reported in Myanmar.

###### Use.

*Whole plant*: Used for treating diabetes.

###### Notes.

In the areas within its range, the species is employed mostly as a tonic and hematic to treat impotence and barrenness or sterility; also a decoction of the plant is used a tonic and an antipyretic drunk as a remedy for dysmenorrhea, and to stimulate the secretion of hormones (Perry 1980). It is noted in that the roots and flowers are used medicinally for clearing away heat and toxic materials (Wu and Raven 1998).

###### Reference.

Nordal (1963).

### Oxalidaceae (Wood-Sorrel family)

#### 1. *Averrhoa* L.

##### Averrhoa carambola

Taxon classificationPlantaeORDOFAMILIA

L.

###### Names.

**Myanmar**: *mak-hpung*, *zaung-ya*. **English**: blimbing, caramba, carambola, country gooseberry.

###### Range.

Malay region; cultivated and often naturalized in the tropics. Cultivated in Myanmar.

###### Uses.

*Fruit*: Used for bleeding piles and fever.

###### Note.

In India the fruit is an antiscorbutic, and cooling; dried fruit is used for fever; ripe fruit is used for bleeding piles, to relieve thirst, and to calm febrile excitation (Jain and DeFilipps 1991).

###### Reference.

Nordal (1963).

### Papaveraceae (Poppy family)

#### 1. *Argemone* L.

##### Argemone mexicana

Taxon classificationPlantaeORDOFAMILIA

L.

###### Names.

**Myanmar**: *khaya*. **English**: Mexican prickly poppy, prickly poppy, yellow prickly poppy.

###### Range.

Florida to Central America; West Indies.

###### Uses.

The juice is used as a treatment for edema. *Seed*: Used in laxative and expectorant preparations. *Root*: Used in the treatment of skin diseases.

###### Notes.

Medicinal uses of this species in India are discussed in Jain and DeFilipps (1991). Chemical constituents, pharmacological action, and medicinal uses of this species in Indian Ayurveda are discussed in detail by Kapoor (1990). Indigenous medicinal uses of this species in the Andaman and Nicobar Islands (India) are described by Dagar and Singh (1999).

The medicinal uses of this plant in the Caribbean region, as well as its chemistry, biological activity, toxicity and dosages, are discussed by Germosén-Robineau (1997). The toxic properties, symptoms, treatment and beneficial used of this plant, parts of which are poisonous, are discussed by Nellis (1997). Worldwide medicinal usage, chemical composition and toxicity of this species are discussed by Duke (1986).

While the oil of this species is not toxic in small amounts, a toxic substance has been isolated from it; two alkaloids, berberine and protopine, are present (Perry 1980). In India L-glutamic acid (6% of defatted meal of oilseed cake) is used in its free state in treating mental deficiencies in infants and adolescents (Perry 1980).

###### References.

Nordal (1963), Perry (1980).

### Passifloraceae (Passion Flower family)

#### 1. *Passiflora* L.

##### Passiflora foetida

Taxon classificationPlantaeORDOFAMILIA

L.

###### Names.

**Myanmar**: *suka*, *taw-suka-ban*. **English**: fetid passionflower, love-in-a-mist, red-fruit passionflower, running-pop, wild water-lemon.

###### Range.

New World tropics. Native to the West Indies and northern South America. Naturalized in Myanmar.

###### Uses.

*Leaf*: Used to treat asthma and hysteria.

###### Notes.

Medicinal uses of this species in India are discussed in Jain and DeFilipps (1991). The toxic properties, symptoms, treatment and beneficial uses of this plant, *parts* of which are *poisonous*, are discussed by Nellis (1997). The species has been found to contain C-glycosylflavonoids (Mors et al. 2000).

###### Reference.

Nordal (1963).

##### Passiflora quadrangularis

Taxon classificationPlantaeORDOFAMILIA

L.

###### Names.

**Myanmar**: *aka-wadi*. **English**: giant granadilla.

###### Range.

Native to New Word tropics. Cultivated in Myanmar.

###### Use.

*Root*: Used in small doses as a vermifuge; in larger doses, it is poisonous.

###### Note.

Worldwide medicinal usage, chemical composition, and toxicity of this species are discussed by Duke (1986).

###### Reference.

Perry (1980).

### Pedaliaceae (Sesame family)

#### 1. *Sesamum* L.

##### Sesamum indicum

Taxon classificationPlantaeORDOFAMILIA

L. (= S. orientale L.)

###### Names.

**Myanmar**: *hnan*, *hmam-gyi*. **English**: sesame.

###### Range.

Tropics. Cultivated in Myanmar.

###### Uses.

*Seed*, *Oil*: Emollient, nutritive, expectorant, laxative, diuretic, abortive (in large doses), antirheumatic, and emmenagogue. The black seeds are preferred.

###### Notes.

In India the seed is used in a “poultice applied externally to ulcers; for piles; as an emmenagogue in a decoction; for a lactagogue, emollient, diuretic, and tonic. Seeds and oil are mixed with other medicines for use as a demulcent for urinary problems and dysentery” (Jain and DeFilipps 1991). Perry (1980) discusses the medicinal uses of this species in China as well as the general medicinal uses of the species.

Reported chemical constituents include fixed oil, lethicin, choline, phytin, globulin, sesamin, and the amino acid arginine (Perry 1980, Duke and Ayensu 1985).

###### References.

Nordal (1963), Perry (1980).

### Pentaphylacaceae (Pentaphylax family)

#### 1. *Anneslea* Wall.

##### Anneslea fragrans

Taxon classificationPlantaeORDOFAMILIA

Wall.

###### Names.

**Myanmar**: *gangawlwe*, *mai-mupi*, *meiktun*, *ngal-hjyang*, *pan-ma*, *pon-nyet*, *taung-gnaw*. **Chinese**: *cha li shu*.

###### Range.

China, Cambodia, Laos, Malaysia, Myanmar, Thailand, and Vietnam. In Myanmar, found in Bago, Chin, Kachin, Kayin, Mandalay, and Shan.

###### Use.

*Flower*: Used as blood purifier.

###### Note.

In Indo-China the bark, mixed with other ingredients (from other plant species), is antidysenteric, also a vermifuge; the flowers are part of a complex preparation to treat fever (Perry 1980).

###### Reference.

Nordal (1963).

### Phyllanthaceae (Phyllanthus family)

#### 1. *Bischofia* Blume

##### Bischofia javanica

Taxon classificationPlantaeORDOFAMILIA

Blume

###### Names.

**Myanmar**: *aukkyu*, *aukkywe*, *hka-shatawi*, *kywe-tho*, *po-gaungsa*, *tayok-the*, *ye-padauk*, *yepadon*. **English**: bishop’s wood.

###### Range.

Tropical Asia. In Myanmar, found in Kachin, Mandalay, and Shan.

###### Use.

*Leaves*, *Juice*: Used as an antiseptic.

###### Notes.

In India juice from the leaf is used to cure sores (Jain and DeFilipps 1991). In China the leaf is used to treat ulcers and boils; sap from the stem is applied to sores; a tonic made from the fruit is used for babies; and the root is employed as a diuretic and for nocturnal emission (Duke and Ayensu 1985).

###### Reference.

Nordal (1963).

#### 2. *Bridelia* Willd.

##### Bridelia retusa

Taxon classificationPlantaeORDOFAMILIA

(L.) A.Juss.

###### Names.

**Myanmar**: *hle-kanan*, *mak-kawng-tawn*, *seik-chi*, *seikchi-bo*. **English**: asana.

###### Range.

China, Bhutan, Cambodia, India, Indonesia, Laos, Myanmar, Nepal, Sri Lanka, Thailand, and Vietnam. Widely distributed in Myanmar.

###### Uses.

*Bark* and *Root*: Used in the removal of urinary concretions and as an astringent.

###### Notes.

In India the bark is used as a liniment for rheumatism (with gingili oil), and as a contraceptive (Jain and DeFilipps 1991). Perry (1980) discusses the uses of the species in Taiwan, the Malay Peninsula, Indo-China, and the Philippines.

###### References.

Nordal (1963), Perry (1980).

#### 3. *Phyllanthus* L.

##### Phyllanthus acidus

Taxon classificationPlantaeORDOFAMILIA

(L.) Skeels (= P. distichus (L.) Müll. Arg.)

###### Names.

**Myanmar**: *mak-hkam-sang-paw*, *thinbaw-zibyu*. **English**: gooseberry tree, Otaheite gooseberry, star gooseberry.

###### Range.

Southern Asia. Naturalized in the West Indies and southern Florida. Reported from Myanmar.

###### Uses.

*Sap*: Milky; used as an emetic and purgative. *Fruit*: An aperient (its vitamin C content approaches in quantity the amount in lemon and grapefruit).

###### Notes.

In India the leaf is used as an antidote to viper poison; the fruit is an astringent; the seed cathartic; and the root cathartic and an antidote to viper poison (Jain and DeFilipps 1991). Perry (1980) notes that the various plant parts have different medicinal uses in different countries. In the Philippines the leaves are applied to urticaria at the same time the astringent fruit is eaten, also a decoction of the bark is used to treat bronchial catarrh; in Indo-China the leaves are used to treat an illness resembling smallpox if accompanied by a cough; in Indonesia the leaves are used as poultices to treat lumbago and sciatica, and the bark heated with coconut oil is spread on eruptions on fingers and hands; and on the Malay Peninsula the root (which is *somewhat poisonous*) is boiled and the steam inhaled as a remedy for cough, and is also used to treat psoriasis of on the soles of the feet (Perry 1980).

###### References.

Perry (1980), Forest Department (1999).

##### Phyllanthus emblica

Taxon classificationPlantaeORDOFAMILIA

L. (= Emblica officinalis Gaertn.)

###### Names.

**Myanmar**: *chay-ahkya*, *chyahkya*, *set-kalwe*, *set-thalwe*, *shabyu*, *tasha*, *taya*, *zee-hpyu*, *zibyu*, *htakyu* (Kachin), *ku-hlu* (Chin), *sot-talwe* (Mon), *hkam mai*, *mai-mak-hkam* (Shan). **English**: emblic, Indian-gooseberry, myrobalan.

###### Range.

Tropical and temperate Asia. Found growing naturally throughout Myanmar, but more commonly in Upper Myanmar and temperate regions.

###### Uses.

Sweet, sour, and astringent in taste, with cooling properties to control agitation, promote circulation, and calm “heat”. *Whole plant*: A laxative. Preparations of the fruits, leaves, and seeds are used to aid digestion and urinary function. A decoction of the five parts (stem, leaf, flower, fruit, and root) is taken to cure diabetes. *Bark* and *Root*: astringent. *Leaf*: A decoction reduced to one-third the starting volume is used as a mouthwash for cracks on the tongue and inside the mouth, as well as for gum boils and gingivitis. Young leaves are eaten with rice vinegar or with nipa palm vinegar (made from the sap of *Nypa fruticans*) to alleviate indigestion and diarrhea. The powder is sprinkled on burns and scalded skin to treat them. A mixture of coconut oil and leaves roasted until burnt is used for sores in infants. *Fruit*: Used to promote longevity; alleviate coughs, asthma, and bronchitis. Also used as an anti-scorbutic, diuretic, and laxative. Juice used to treat inflammation of the eyes. The powder can be eaten mixed together with jaggery, honey, and/or molasses to cure urinary infections. Juice extracted from crushed fruit is taken with lime juice for instant relief from dysentery. A mixture of dried fruit cooked together with eel is also used for dysentery. A mixture of the paste from the dried or fresh fruit with ginger and a small amount of lime juice is applied topically for itches, rashes, ringworm and other fungal skin infections and freckles; it is also used with hsee-cho (*Orthosiphon aristatus*) for discoloration of the cheeks. For nosebleeds, fruit is crushed very finely and applied to the head as a poultice. *Seed*: A wash made from crushed seeds and boiling water is used for eye infections.

###### Notes.

Medicinal uses of this species in India are discussed in Jain and DeFilipps (1991) as follows: The bark is used on sores and pimples; tubercular fistula (in combination with bark fro three other species); and for cholera, dysentery (with other plants), and diarrhea. The leaf is used for diarrhea and sores. The fruit is used as a diuretic and laxative, as well as for indigestion, gonorrhea (with two other plants); raw fruit is used as an aperient, dried and used in haemorrhagia, diarrhea, as a liver tonic, for scurvy, and the juice as eye drops. The seed is used for asthma and stomach disorders. Perry (1980) discusses the medicinal uses of this species in South China, Indo-China, Indonesia, and India.

The fruit is considered the richest natural source known of vitamin C (“The juice contains nearly 20 times as much vitamin C as orange juice.”); the “tannin (containing gallic acid, ellagic acid, and glucose) naturally present in the fruit retards the oxidation of the vitamin, so the fruit “is a valuable antiscorbutic either fresh or dry” (Perry 1980).

###### References.

Nordal (1963), Agricultural Corporation (1980), Perry (1980), Forest Department (1999).

##### Phyllanthus niruri

Taxon classificationPlantaeORDOFAMILIA

L.

###### Names.

**Myanmar**: *flor-de-joja*, *kyet-tha-hin*, *yaung-ma-ywet*. **English**: gale of the wind.

###### Range.

West Indies. Widely distributed in Myanmar.

###### Uses.

The plant is used as a diuretic and for menorrhagia.

###### Notes.

In India, the whole plant is used as a diuretic, for urinogenital tract diseases, gonorrhea, and dropsy; the milky juice is applied to putrescent sores; the leaf is used as a stomachic; the fresh root is used for jaundice; powdered roots and leaves are made into a poultice with rice-water and used to reduce ulcers and edematous swellings; and infusion of young stems used for dysentery (Jain and DeFilipps 1991). Perry (1980) discusses the uses of the species in general and from Hainan to Indonesia; also the Malay Peninsula, Indonesia, Guam, and the Northwest Solomon Islands.

Reported chemical constituents include potassium and phyllanthin (a substance said to poison fish) (Perry 1980). An extract has shown some antibiotic activity on *Staphylococcus* (Perry 1980).

###### Reference.

Nordal (1963).

### Piperaceae (Pepper family)

#### 1. *Piper* L.

##### Piper betle

Taxon classificationPlantaeORDOFAMILIA

L.

###### Names.

**Myanmar**: *kun*, *pu* (Shan), *bu*, *buru* (Kachin). **English**: betel, betel pepper, betel vine.

###### Range.

Old World tropics.

###### Uses.

*Leaf*: Bitter, astringent, and hot in taste, known for whetting the appetite, reducing phlegm, controlling flatulence, promoting vitality and virility, neutralizing poison, supporting heart and bowel functions, and curing coughs and heart disease. Children are given a mixture of honey and the juice from the crushed leaves to alleviate indigestion, gas, diarrhea, fevers, and other illnesses. Juice from crushed leaves is taken with milk as a remedy for emotional distress related to the menstrual cycle. A mixture of the juice from the crushed leaves, rock salt, and a decoction of ginger is used for asthma, chest pain, indigestion, and whooping cough. The juice from the crushed leaves is applied as eyedrops for night blindness, sore or inflamed eyes, and other eye problems. A leaf decoction made with turmeric and a bit of salt is taken for fevers and illnesses. Roasted until limp but not dry, leaves or applied with coconut oil in compresses on the soft spots of children’s heads to cure runny noses. A decoction of the leaves with jaggery and salt is taken for fever caused by heat stroke.

###### Notes.

The medicinal uses of this species in India are discussed in Jain and DeFilipps (1991). Medicinal uses of this species in China are discussed in Duke and Ayensu (1985).

###### Reference.

Agricultural Corporation (1980).

##### Piper cubeba

Taxon classificationPlantaeORDOFAMILIA

L.f.

###### Names.

**Myanmar**: *sayo pin*. **English**: cubeb pepper, Java pepper.

###### Range.

Tropical Asia. Grows naturally in Myanmar; thrives in wet and humid areas.

###### Uses.

*Whole plant*: Sharp, hot, bitter, and easily digestible, the flowers, fruits, roots, stems, and whole plant are employed in preparations to aid digestion, kill germs, and control the phlegm and gas. *Stem*: A steamed mixture of the stems, rice dough, and a little salt is eaten to purify blood, promote vitality, ease aches and pains, and alleviate male- and female-related disorders. The same preparation is considered particularly suitable for people convalescing from malaria. *Flower*: Used in medicines to treat coughs and asthma. *Fruit*: Used to alleviate stomach distension, coughs, and colds; also in digestives and tonics. *Root*: Used to neutralize poisons; also to treat coughs, bronchitis, asthma, hemorrhoids, and gas disorders in the stomach.

###### Notes.

The medicinal uses of this species in India are discussed in Jain and DeFilipps (1991). Medicinal uses of this species in China are discussed in Duke and Ayensu (1985).

###### Reference.

Agricultural Corporation (1980).

##### Piper longum

Taxon classificationPlantaeORDOFAMILIA

L.

###### Names.

**Myanmar**: *peik-chin*, *nga-yok-kaung*, *tanwhite* (Mon). **English**: Indian long pepper.

###### Range.

Himalayas (Nepal to Bhutan), India, Sri Lanka, and Malaysia. Grows naturally throughout Myanmar, but especially in the mountainous northern part of the country in the shade of large trees; also cultivated.

###### Uses.

*Fruit*: Used as a digestive. Paste made with water used to cure a stiff neck; when applied to bites of venomous animals, it neutralizes the venom. Powder taken with hot water used in deworming and to relieve pain in the chest. Licking the powder mixed with honey reduces excessive passing of blood. Boiling the fruit pod with jaggery and ginger, and drinking the liquid reduces fever and eases aches and pains in cases of malaria, ague, and influenza. The pod is also chewed to relieve toothaches. *Root*: A small amount of root powder taken with warm water is used to relieve inflammation of the joints as well as backaches. The root is also used to aid digestion.

###### Notes.

The medicinal uses of this species in India are discussed in Jain and DeFilipps (1991). Medicinal uses of this species in China are discussed in Duke and Ayensu (1985).

###### References.

Nordal (1963), Agricultural Corporation (1980), Forest Department (1999).

##### Piper nigrum

Taxon classificationPlantaeORDOFAMILIA

L.

###### Names.

**Myanmar**: *ngayoke-kaung*, *mawrite nawa* (Mon). **English**: black pepper.

###### Range.

Tropical Asia. Cultivated along Myanmar’s coastal areas and Kayin State; thrives in temperatures between 10 and 37.8 degrees Celsius, with at least 1.5 m of rainfall annually.

###### Uses.

*Fruit*: Used as a digestive. *Seed*: Spicy hot, the seeds (peppercorns) are used to stimulate taste buds, whet the appetite, support liver function and circulation, and to reduce phlegm and gas. Powdered peppercorns are mixed with honey and licked to relieve coughs, asthma, and bronchitis and to promote lactation in nursing mother; mixed with *shein kho* (*Gardenia resinifera*) and opium and taken for chronic diarrhea; mixed with liquid yogurt and sugar to treat nosebleeds and runny noses; and mixed with seeds from *anyar-khayar* (either *Bombax ceiba* or *Ceiba pentandra* seeds) to neutralize bites from rabid dogs. A paste made of peppercorns and yogurt is used as eye drops to treat night blindness. As a cure for the hiccups, the fumes from heated peppercorns are inhaled. Pepper is eaten to promote digestion, to support urinary function, and to alleviate stomach distension and hemorrhoids. A mixture of powdered pepper and the powdered, dried stems from *new-cho* (*Albizia myriophylla*) is licked to relieve palpitations and abdominal pains caused by gas.

###### Notes.

The medicinal uses of this species in India are discussed in Jain and DeFilipps (1991). Medicinal uses of this species in China are discussed in Duke and Ayensu (1985).

###### References.

Nordal (1963), Agricultural Corporation (1980), Forest Department (1999).

### Plantaginaceae (Plantain family)

#### 1. *Digitalis* L.

##### Digitalis lanata

Taxon classificationPlantaeORDOFAMILIA

Ehrh.

###### Names.

**English**: Grecian foxglove, woolly foxglove.

###### Range.

Danube region and Greece; naturalized in northeastern North America. Cultivated in Myanmar.

###### Use.

*Leaf*: Used as heart tonic.

###### Notes.

In India the leaf is used as a cardiac stimulant and tonic (Jain and DeFilipps 1991).

Reported chemical constituents include the cardiac glycosides, dioxin, gitoxin, and dilanane (Perry 1980). Dried leaves are a source of the drug digitalis. Two European species of *Digitalis* are cultivated in the Far East as a well known source of the heart tonic digitalin (Perry 1980). *D. lanata* is said to have greater strength that *D. pupurea* (Perry 1980).

###### Reference.

Nordal (1963).

##### Digitalis purpurea

Taxon classificationPlantaeORDOFAMILIA

L.

###### Names.

**Myanmar**: *tila-pup-hpi*. **English**: apricot blush foxglove, common foxglove, digitalis, purple foxglove.

###### Range.

A polymorphic species centered in the Mediterranean region. Naturalized elsewhere, including northern Africa; northern, middle, and southeastern Europe; also cultivated. Cultivated in Myanmar.

###### Use.

*Leaf*: Used as heart tonic.

###### Notes.

Dried leaves are a principle source of the drug digitalis. In India the leaf of this species is used for heart and kidney disease; also applied locally on wounds and burns (Jain and DeFilipps 1991). Reported uses for the species include as a bactericide, cardiotonic, cardiostimulant, tonic, diuretic, sedative, stimulant; also for dropsy, edema, fever, insanity, neuralgia, palpitation, renitis, and tumor; also, a poison (Duke 2009).

Research has shown that chemicals found in this plant are effective as a bacteriocide and cardiotonic (Duke 2009).

###### Reference.

Nordal (1963).

#### 2. *Plantago* L.

##### Plantago major

Taxon classificationPlantaeORDOFAMILIA

L.

###### Names.

**Myanmar**: *a-kyaw ta-htaung*, *bar-kyaw pin*, *hsay-kyaw gyi*. **English**: broad-leaved plantain, cart-track-plant, common plantain, great plantain, plantain, ribwort, white man’s foot.

###### Range.

Europe and Asia; considered a cosmopolitan weed. In Myanmar, grows naturally in cold places at high altitudes, such as Pyin-oo-lwin and surrounding areas.

###### Conservation status.

Least Concern [LC] (IUCN 2017).

###### Uses.

Leaves, roots, stems, flowers, and fruits are used. *Whole plant*: Consuming the five parts stewed in water regularly is considered a cure for diabetes; drinking the juice of the five parts every morning and evening is considered a cure for lung disease. The plant can be used either as an oral or external medicine to cure inflammation and aches in the joints, stomach pain, and general aches and pains. It is also widely used as a tonic for strength. *Leaf* and *Root*: A decoction of the leaves and the root is given for fever of long duration and intermittent fever. *Leaf*: The leaves have cooling properties that promote urination. Finely crushed leaves are used as a poultice on bee, wasp, and other stings to neutralize venom quickly, as well as to stop bleeding from cuts and other injuries. A decoction of the leaves is used as a wash to cleanse sores and stimulate new tissue formation. The leaf decoction is also used warm as a mouthwash and gargle for oral inflammation, swollen and infected gums, and gingivitis. For earaches and ear infections exuding pus, juice from the crushed leaves is used as eardrops applied 2–3 times daily. Juice from the crushed leaves is also given to cure malaria. Steam from cooked leaves is used for steam baths to remedy white vaginal discharge, gonorrhea in men and women, hemorrhoids, and bloating. Leaves roasted until limp are applied twice daily to draw out embedded thorns and to heal sores quickly. Ingesting the leaf decoction with sugar alleviates urinary problems, prickly heat, impetigo (caused by species of *Staphylococcus* and *Streptococcus* bacteria), erysipelas (caused by *Streptococcus*), intestinal disease/ inflammation.

###### Notes.

Medicinal uses of this species in India are discussed in Jain and DeFilipps (1991). Indigenous medicinal uses of the species in the Andaman and Nicobar Islands (India) are described by Dagar and Singh (1999).

The medicinal uses of this plant in the Caribbean region, as well as its chemistry, biological activity, toxicity and dosages, are discussed by Germosén-Robineau (1997). The chemistry, pharmacology, history, and medicinal uses of this species in Latin America are discussed in detail by Gupta (1995). Worldwide medicinal usage, chemical composition, and toxicity of this species are discussed by Duke (1986).

###### Reference.

Agricultural Corporation (1980).

#### 3. *Scoparia* L.

##### Scoparia dulcis

Taxon classificationPlantaeORDOFAMILIA

L.

###### Names.

**Myanmar**: *dar-na-thu-kha*, *dana-thuka*, *thagya-bin*. **English**: licorice weed, sweet-scented broom.

###### Range.

Pantropical to subtropical. In Myanmar, found in Bago, Chin, Mandalay, Taninthayi, and Yangon.

###### Uses.

*Whole plant*: Used to treat toothaches; dried and used as a herbal tea to treat blood in urine; crushed and mixed with salt, and applied to sores to aid in healing. Drug prepared from this plant is used in the treatment of diabetes. *Leaf*: Used to treat fevers and nausea. *Root*: Used for excessive menstruation and gonorrhea, also to treat nausea and dizzy spells. Raw root crushed and pressed on tooth for toothaches.

###### Notes.

Medicinal uses of this species in India are discussed in Jain and DeFilipps (1991). Indigenous medicinal uses of this species in the Andaman and Nicobar Islands (India) are described by Dagar and Singh (1999). Medicinal uses of this species in China are discussed by Duke and Ayensu (1985). The chemistry, pharmacology, history, and medicinal uses of this species in Latin America are discussed in detail by Gupta (1995).

The following are given in the literature as medicinal uses for this species: Treatment of rashes, sores, wounds, bruises, eczema; earache, headache, toothache, sore throat, cough, bronchitis, fever; spasm; for tumor, albuminuria, amygdalosis, anemia, blennorrhagia, conjunctivitis, diabetes, diarrhea, dysentery, dysmenorrhea, gonorrhea, gravel, grip, hyperglycemia, inflammation, jaundice, ketonuria, kidney problems, mange, marasmus, menorrhagia, metroxenia, nerves, ophthalmia, piles, retinitis, snakebite; for use as an antidote, antiseptic, astringent, depurative, diuretic, emetic, purgative; also used as an insecticide (Duke 2009).

Research has shown chemicals found in this plant to be effective in the treatment of albuminuria, anemia, diabetes, hyperglycemia, and retinitis (Duke 2009).

###### References.

Mya Bwin and Sein Gwan (1967), Agricultural Corporation (1980), Forest Department (1999).

### Plumbaginaceae (Leadwort family)

#### 1. *Plumbago* L.

##### Plumbago indica

Taxon classificationPlantaeORDOFAMILIA

L. (= P. rosea L.)

###### Names.

**Myanmar**: *kant-choke-ni*, *kangyok*. **English**: fire plant, rosy leadwort, scarlet leadwort.

###### Range.

Southeast Asia. Found growing all over Myanmar except in the hot and very cold regions; grows naturally but can be also found cultivated in hedges for use as a medicinal plant.

###### Uses.

The five parts (root, stem, leaf, flower, and fruit) are used. The plant has a sharp hot taste and is considered good for digestion and strength. The entire plant is known for slowing aging and supporting longevity. The crushed whole plant is used topically for eye ailments, scabies, and leucoderma. *Root*: Used as an expectorant, also promotes well-being, appetite, and weight gain. The root is used to treat leprosy, venereal disease, and menstrual disorders. A mixture of crushed roots and mild oil is applied topically to alleviate joint soreness and partial paralysis. The root is also used in medicines for digestive disorders, anemia, throat cancer, bloating, edema, and skin disorders.

###### Notes.

The medicinal uses of this species in India are discussed in Jain and DeFilipps (1991). Medicinal uses of this species in China are discussed in Duke and Ayensu (1985).

###### References.

Agricultural Corporation (1980), Forest Department (1999).

##### Plumbago zeylanica

Taxon classificationPlantaeORDOFAMILIA

L.

###### Names.

**Myanmar**: *kan-gyok-phyu*, *tanah-con-kamor* (Mon). **English**: Ceylon leadwort, white leadwort.

###### Range.

Southeast Asia. Found growing naturally throughout Myanmar; also cultivated. there.

###### Uses.

The entire plant, root, and sticky sap are used. *Whole plant*: Used to stimulate the palate and promote digestion. The plant in its entirety is used as an ingredient in medicines for diarrhea, gastric diseases, and herpes-like skin disorders. *Sap*: The milky sap is also used topically for skin problems, including ringworm and boils. *Leaf*: Sweet with a sharp taste, used for dissolving phlegm. *Root*: Used for gas, phlegm, and bile problems; and used in deworming and blood purification medicines. It can also be used to cure dysentery, leucoderma, lung diseases, bloating, wasting, and aches and pains, as well as skin problems, such as eczema, scabies, and ringworm. A mixture of crushed roots, milk, and vinegar or salt is applied topically as a remedy for leprosy and other skin infections. The juice of the roots is used to induce sweating. A mixture of the root and other ingredients is used to heal boils and sores.

###### Notes.

The medicinal uses of this species in India are discussed in Jain and DeFilipps (1991). Medicinal uses of this species in China are discussed in Duke and Ayensu (1985).

###### References.

Agricultural Corporation (1980), Forest Department (1999).

### Poaceae (Grass family)

#### 1. *Arundo* L.

##### Arundo donax

Taxon classificationPlantaeORDOFAMILIA

L.

###### Names.

**Myanmar**: *alo-kyu*, *kyu*, *kyu-ma*, *mai-aw-awn* (Shan), *maiaw* (Kachin). **English**: giant reed, nana cane, Spanish cane, switch cane.

###### Range.

Mediterranean region; also in tropical America. In Myanmar, found growing naturally all over up to 1 km altitude, most common in Bhamaw, Katha, Pyin-oo-lwin and Thayet areas.

###### Conservation status.

Least Concern [LC] (IUCN 2017).

###### Uses.

With cooling properties, as well as bitter, sweet and astringent tastes, this plant facilitates digestion, clears phlegm, repels bile, purifies blood, and diminishes “heat”. It relieves aches and pains in the heart, bladder and uterus, in addition to curing herpes, stimulating appetite, increasing sperm, purifying urine and strengthening breathing.

*Leaf*: When dried can be brewed with tea leaves and taken to stimulate appetite, promote virility, stop vomiting, remedy passing of blood, and relieve muscle aches, pains and stiffness. *Root*: Used as diuretic, for urine purification, gonorrhea, itchy skin, and menstrual flow stimulation; the root mass is boiled in water, and the resulting liquid is ingested. Adding the powder of the tiger cowry (*Cypraea tigris*) to the liquid in which the root mass has been boiled and ingesting the mixture used to treat women for the red or white discharges of gonorrhea. Because this plant promotes urination, it is an ingredient in many diuretics. A mixture containing ten parts of the root mass, five parts tiger cowry, two parts rock salt, five parts *hsin-hnamaung* (*Heliotropium indicum* or *Tournefortia roxburghii*) and one part sting ray is made into balls the size of betel (*Piper betle*) nuts, and dried in the sun as a treatment for kidney stones, bladder or urination pain, blood in the urine, incomplete urination in males, and dysentery in females. The mixture is taken once in the morning and once at night for symptom relief and to promote health.

###### Notes.

The medicinal uses of this species in India are discussed in Jain and DeFilipps (1991). In Indo-China the rhizome serves as a lactifuge (Perry 1980).

A reported chemical constituent of the species is the alkaloid gramine (donaxine). Research has indicated that this alkaloid causes weak parasympathomimetic action (Perry 1980).

###### References.

Nordal (1963), Agricultural Corporation (1980), Perry (1980), Forest Department (1999).

#### 2. *Bambusa* Schreb.

##### Bambusa bambos

Taxon classificationPlantaeORDOFAMILIA

(L.) Voss (= B. arundinacea Willd.)

###### Names.

**Myanmar**: *kyakat-wa*, *nga-chat-wa*. **English**: giant thorny bamboo, spiny bamboo.

###### Range.

India to China, south through Thailand and Indo-China; cultivated elsewhere. Reported from Myanmar.

###### Use.

*Shoot*: Applied as poultice; also edible.

###### Notes.

In China the species is used as a treatment for jaundice, indigestion, and water retention; also, “The sap of the stem or a decoction of the unfolding leaves is administered as a treatment for fevers and rheumatic affections” (Perry 1980). In Indo-China refreshing emollient leaves are used to treat fever, sore throat, and cough; finely chopped bark serves as an astringent for hemorrhage, menorrhea, nausea, and vomiting; roots and buds are emollient, diuretic, diaphoretic, and depurative, and are given for obstructions, and urinary and venereal problems; fresh roots, mixed with tobacco and *Piper betle* leaves and macerated in oil, serve as an unguent effective on hard tumors and cirrhosis; bark is bechic; and juice from young branches passed through fire are used to give relief for inflamed bronchial tubes (Perry 1980).

###### Reference.

Perry (1980).

#### 3. *Coix* L.

##### Coix lacryma-jobi

Taxon classificationPlantaeORDOFAMILIA

L.

###### Names.

**Myanmar**: *ka-leik*, *kalein*, *kalein-thi*, *kyeik*. **English**: adlay, adlay millet, Job’s tears.

###### Range.

Southeast Asia. In Myanmar found in Kachin and Yangon.

###### Uses.

*Seed*: Used to reduce body weight and as a diuretic.

###### Notes.

The medicinal uses of this species in India are discussed in Jain and DeFilipps (1991). Medicinal uses of this species in China are discussed in Duke and Ayensu (1985).

Perry (1980) covers the species’ uses in China, Japan, and India to the Philippines, and states that the kernels, separated from the shell, are used as a diuretic, stomachic, tonic; also to treat lung and chest complaints, rheumatism, dropsy, and gonorrhea.

The seeds contain coicin, glutamic acid, histidin, arginin, leucin, lycin, and tyrosin; the acetone extract of the seeds is said to show a growth-inhibiting activity, or an antitumor component, coixenolide (Perry 1980).

###### Reference.

Nordal (1963).

#### 4. *Cymbopogon* Spreng.

##### Cymbopogon citratus

Taxon classificationPlantaeORDOFAMILIA

(DC.) Stapf.

###### Names.

**Myanmar**: *sapalin*, *hkum-bang-pan* (Kachin), *wine-baing* (Mon). **English**: citronella grass, fever grass, lemon grass.

###### Range.

Southern India and Sri Lanka. Cultivated in Myanmar; grows all over, up to 610 m altitude.

###### Uses.

Bitter and astringent in taste, plant is used for heart and throat problems, flatulence and phlegm conditions, sicknesses that cause blood vomiting, cholera, coughs and fevers with chest congestion. It promotes healthy gall bladder function, circulation and digestion. *Whole plant*: Crushed and wrapped in a cloth, the plant is pressed over inflamed areas to ease pain. The oil is rubbed vigorously into joints to relieve inflammation. Where malaria is endemic, the oil is heated together with wax to make an ointment used topically as a mosquito repellent. *Stem*: Crushed stems mixed with peppercorns are formed into pellets that are ingested daily to cure fever and malaria. Also, the liquid from boiling a handful of stems (without the tips or roots) in water to one-third the starting volume is taken at least three times a day for 3 days to cure jaundice. The juice from lemon grass is also used to treat indigestion and promote appetite.

###### Notes.

The medicinal uses of this species in India are discussed in Jain and DeFilipps (1991). Medicinal uses of this species in China are discussed in Duke and Ayensu (1985).

###### Reference.

Agricultural Corporation (1980).

##### Cymbopogon jwarancusa

Taxon classificationPlantaeORDOFAMILIA

(Jones) Schult.

###### Names.

**English**: iwarancusa grass, millet, oilgrass.

###### Range.

Africa; Asia.

###### Uses.

*Oil*: Considered a valuable liniment to treat rheumatism. *Root*: Thought to be of great value in curing a number of fevers, including malaria.

###### Notes.

In India the leaf is used for cough, rheumatism, and cholera; also as a tonic for dyspepsia and to purify blood (Jain and DeFilipps 1991).

The oil from the roots contains DL-piperitone and D-∆​4-carene (Perry 1980).

###### Reference.

Perry (1980).

##### Cymbopogon nardus

Taxon classificationPlantaeORDOFAMILIA

(L.) Rendle

###### Names.

**Myanmar**: *sabalin-hmwe*, *myet-hmwe*. **English**: Ceylon citronella, citronella, citronella grass.

###### Range.

Native to southern India and Sri Lanka. Introduced elsewhere as a crop plant; commonly cultivated. Cultivated throughout Myanmar, up to 610 m altitude.

###### Uses.

Bitter and sweet in taste, the plant can cause loose bowels, and feelings of hunger. It can be used to control flatulence and to treat leprosy, epilepsy, and diseases associated with the intestines. *Whole plant*: Used as an antispasmodic, carminative, and diaphoretic. *Oil*: Used topically to relieve joint inflammation; on the scalp to stop hair loss; and on the skin to treat scabies, rashes and other conditions. *Leaf*: Liquid from soaking the leaves in hot water can be taken for shooting stomach pains. Juice from crushed leaves is applied to treat arm or leg paralysis. Liquid from leaves boiled in water to one-fourth the starting volume is ingested for fevers, coughs, and colds.

###### Note.

Medicinal uses of this species in India are discussed in Jain and DeFilipps (1991).

###### References.

Nordal (1963), Agricultural Corporation (1980).

#### 5. *Dactyloctenium* Willd.

##### Dactyloctenium aegyptium

Taxon classificationPlantaeORDOFAMILIA

(L.) Willd.

###### Names.

**Myanmar**: *didok-chi*, *myet-lay-gwa*. **English**: Egyptian grass.

###### Range.

Southeastern Europe; northern Africa; Macaronesia; Atlantic, Pacific and western Indian Ocean islands; temperate Asia; Arabia; China; India; Indo-China; Malesia; Australia; North America; Mexico; South and Meso-America; Caribbean. In Myanmar, found in Bago, Kachin, Mandalay, Taninthayi, and Yangon.

###### Uses.

*Seed*: Used an anodyne and antispasmodic.

###### Notes.

Medicinal uses of this species in China are discussed in Duke and Ayensu (1985). In India parched grains are eaten by women suffering from post-childbirth stomachache (Jain and DeFilipps 1991). The species has astringent properties and, in the Philippines, is used internally in a decoction to treat dysentery and acute hemoptysis (Perry 1980).

###### Reference.

Nordal (1963).

#### 6. *Phragmites* Adans.

##### Phragmites karka

Taxon classificationPlantaeORDOFAMILIA

(Retz.) Trin. ex Steud.

###### Names.

**Myanmar**: *kyu*, *kyu-a*, *kyu-kaing*, *kyu-wa-kaing*. **English**: carrizo, common reed.

###### Range.

Widely distributed in the warm and temperate zones; common in marshes and wet places. Reported from Myanmar.

###### Conservation status.

Least Concern [LC] (IUCN 2017).

###### Uses.

*Root*: Used as a diuretic and diaphoretic.

###### Notes.

The many medicinal many uses of the species in China are discussed in Duke and Ayensu (1985) as follows: The leaf is used for bronchitis, cholera; ash for foul sores The flower is decocted in water to treat cholera, fish and shrimp poisoning, ashes styptic. The stem shoot is antidotal, antiemetic, antipyretic, refrigerant, for cholera; ash is applied to foul sores. The root is decocted as an antiemetic, antipyretic, diuretic, febrifuge, sialogogue, stomachic for abscess, arthritis, cough, earache, fever, hematuria, hiccups, nausea, pulmonary abscess, sore throat, sunstroke, and toothache. They additionally note that the herb is said to be used in Chinese medicine for leukemia. Perry (1980) discusses the medicinal uses of the species in China and the Malay Peninsula.

Reported constituents include asparagine, proteins, and glycosides (Perry 1980).

###### Reference.

Nordal (1963).

#### 7. *Zea* L.

##### Zea mays

Taxon classificationPlantaeORDOFAMILIA

L.

###### Names.

**Myanmar**: *pyaung-bu*. **English**: corn, maize.

###### Range.

New World, probably Mexico. Cultivated in Myanmar.

###### Use.

*Flower*: A fermented preparation from the style of the plant is said to have a strong hypoglycemic effect.

###### Notes.

In India the grain is used in the diet of consumptive patients, for treating relaxed bowels, as an astringent, and as a resolvent (Jain and DeFilipps 1991). In China a decoction of the leaf and roots is used for dysuria. Corn silks are used as a diuretic in dropsy, to treat diabetes mellitus, and decocted with banana and watermelon peel for hypertension. A cob decoction is used for epistaxis and meorrhagia. The seed is widely used for cancers, tumors, and warts. A decoction of the root is used for blenorrhea and dyusuria (Duke and Ayensu 1985).

In Haiti an infusion of the styles is used as a diuretic and for kidney problems; a decoction or maceration of the styles is used for inflammations and edema; the ground grains are used in a warm compress on traumatized areas and swellings; a cataplasm of the ground grains is applied to fractures; and, split ears of corn are made into an infusion as an antihypertensive (Neptune-Rouzier 1997). Among Afro-Cuban religions, in the Ocha Rule (also called Santeria), this species is a sacred plant belonging to all the orishas (“saints”); “It is considered a sign of good luck when maize grains spontaneously sprout around a house” (Fuentes 1992). The medicinal uses of this plant in the Caribbean region, as well as its chemistry, biological activity, toxicity and dosages, are discussed by Germosén-Robineau (1997). Details of the active chemical compounds, effects, herbal usage and pharmacological literature of this plant are given in Fleming (2000).

###### Reference.

Mya Bwin and Sein Gwan (1967).

### Polygonaceae (Buckwheat family)

#### 1. *Fagopyrum* Mill.

##### Fagopyrum esculentum

Taxon classificationPlantaeORDOFAMILIA

Moench

###### Names.

**Myanmar**: *shari-mam*. **English**: brank, buckwheat, common buckwheat, notch-seeded buckwheat.

###### Range.

Central or northern Asia. Widely grown as cultigen in cool temperate regions, and easily escaping.

###### Uses.

*Fruit*: Used to treat colic and diarrhea.

###### Note.

Medicinal uses of this species in China are discussed in Duke and Ayensu (1985).

###### Reference.

Nordal (1963).

#### 2. *Persicaria* Mill.

##### Persicaria chinensis

Taxon classificationPlantaeORDOFAMILIA

(L.) H.Gross (= Polygonum chinense L.)

###### Names.

**Myanmar**: *boktaung*, *wetkyein*, *maha-gar-kyan-sit.* **English**: Chinese knotweed, Chinese smartweed.

###### Range.

North temperate. Found in China, Bhutan, India, Indonesia, Japan, Malaysia, Myanmar, Nepal, Philippines, Sikkim, Thailand, and Vietnam. In Myanmar found in Ayeyarwady, Bago, Kachin, Mandalay, and Yangon.

###### Use.

*Whole plant*: Used as an antiscorbutic.

###### Note.

Medicinal uses of this species in China are discussed in Duke and Ayensu (1985).

###### Reference.

Nordal (1963).

##### Persicaria pulchra

Taxon classificationPlantaeORDOFAMILIA

(Blume) Soják (= Polygonum pulchrum Blume)

###### Names.

**Myanmar**: *mahaga-kyansit*. **English**: curltop ladysthumb, curlytop knotweed, curlytop smartweed, dock-leaf smartweed, nodding smartweed, pale smartweed, smartweed.

###### Range.

China, Taiwan, India, Indonesia, Malaysia, Myanmar, Philippines, Sri Lanka, Thailand; and Australia. In Myanmar found in Mandalay and Yangon.

###### Use.

*Root*: A decoction is used for stomach problems in children.

###### Note.

On the Malay Peninsula the leaves are used as tonic (Perry 1980).

###### Reference.

Perry (1980).

### Pontederiaceae (Water-Hyacinth family)

#### 1. *Monochoria* C.Presl

##### Monochoria vaginalis

Taxon classificationPlantaeORDOFAMILIA

(Burm.f.) C.Presl

###### Names.

**Myanmar**: *beda*, *le-padauk*, *kadauk-sat*. **English**: cordate monochoria, oval-leaf monochoria, oval-leaf pondweed, pickerel weed.

###### Range.

Throughout China, Bhuton, Cambodia, India, Indonesia, Japan, Korea, Laos, Malaysia, Nepal, Pakistan, the Philippines, Sri Lanka, Thailand, Vietnam; Russia (Siberia); Africa; and Australia. In Myanmar, the species in found in Taninthayi and Yangon.

###### Conservation status.

Least Concern [LC] (IUCN 2017).

###### Uses.

*Whole Plant*: Provides a medicine used to treat diseases of the digestive organs, asthma, and toothache. *Leaf*: Juice used for fever. *Flower*: Edible and has a cooling effect. *Root*: Used for toothache and asthma; juice used to treat stomach and liver problems.

###### Notes.

In India the bark is eaten with sugar to relieve asthma; the root is chewed to relieve toothache (Jain and DeFilipps 1991). In China the plant is used for cholera, stomachache, and sunstroke; the flower is edible and serves as a refrigerant (Duke and Ayensu 1985). Perry (1980) discusses the medicinal uses of the species in China, Taiwan, the Malay Peninsula, Indonesia, and the Philippines.

###### References.

Nordal (1963), Perry (1980).

### Portulacaceae (Purslane family)

#### 1. *Portulaca* L.

##### Portulaca oleracea

Taxon classificationPlantaeORDOFAMILIA

L.

###### Names.

**Myanmar**: *myet-htauk*, *myay-byit*. **English**: common purslane, duckweed, garden purslane, little hogweed, purslane, pursley, wild portulaca.

###### Range.

Thought probably originally native to southwestern United States, and now widely distributed in warm temperate, tropical, and subtrobical regions throughout world. Cosmopolitan weed; also cultivated, and with many medicinal uses. Much variation in the species.

###### Uses.

*Leaf*: Used in kidney disease treatment; also, as a laxative and digestive.

###### Notes.

Medicinal uses of this species in India are discussed in Jain and DeFilipps (1991). Indigenous medicinal uses of this species in the Andaman and Nicobar Islands (India) are described by Dagar and Singh (1999). Medicinal uses of this species in China are discussed by Duke and Ayensu (1985).

The chemical constituents, pharmacological activities, and traditional medicinal uses of this plant on a worldwide basis are discussed in detail by Ross (1999). A pharmacognostic profile including medicinal uses of this plant in Africa is given in Iwu (1993). Uses of this plant in the Upper Amazon region, including for gonorrhea, hepatitis and herpes, are given by Castner et al. (1998). This species contains high concentrations of catecholamine derivatives such as (-)-noradrenaline, DOPA and dopamine (Mors et al. 2000).

###### Reference.

Nordal (1963).

### Primulaceae (Primrose family)

#### 1. *Ardisia* Sw.

##### Ardisia humilis

Taxon classificationPlantaeORDOFAMILIA

Vahl

###### Names.

**Myanmar**: *shadwe*, *kyet-maok*, *kyet-ma-oak*. **English**: low shoebutton.

###### Range.

China, Philippines, and Vietnam. In Myanmar, found in Bago, Mandalay, Rakhine, and Taninthayi.

###### Uses.

*Whole plant*: All parts of the plant are used in treating menstrual disorders. *Leaf*: Used as carminative and stimulant.

###### Note.

This species is reported as used in the treatment of diarrhea, fever, and rheumatism (Duke 2009).

###### References.

Nordal (1963), Perry (1980).

### Putranjavaceae (Putranjiva family)

#### 1. *Putranjiva* Wall.

##### Putranjiva roxburghii

Taxon classificationPlantaeORDOFAMILIA

Wall.

###### Names.

**Myanmar**: *badi-byu*, *daukyat*, *mai-mot*, *mai-motawn*, *taukyat*, *ye-padi*. **English**: officinal drypetes, putranjiv tree.

###### Range.

India. In Myanmar, found in Mandalay, Mon, and Yangon.

###### Use.

*Leaf*: Used to treat diabetes.

###### Note.

In India the leaf and fruit (including the stones) are used as a decoction for fevers and colds (Jain and DeFilipps 1991).

###### References.

Nordal (1963), Forest Department (1999).

### Ranunculaceae (Buttercup family)

#### 1. *Clematis* L.

##### Clematis smilacifolia

Taxon classificationPlantaeORDOFAMILIA

Wall.

###### Names.

**Myanmar**: *khwar-nyo-gyi*.

###### Range.

China, Bangladesh, Bhutan, Cambodia, India, Indonesia, Malaysia, Myanmar, West Nepal, New Guinea, Philippines, Sikkim, Sri Lanka, Thailand, and Vietnam. In Myanmar, found in Chin, Kachin, Mandalay, Sagaing, Shan, and Taninthayi.

###### Use.

*Root*: Used as an antirheumatic.

###### Note.

In China the plant is used to relieve itch and a decoction of the root is used to treat lumbago (Perry 1980).

###### Reference.

Nordal (1963).

#### 2. *Coptis* Salisb.

##### Coptis teeta

Taxon classificationPlantaeORDOFAMILIA

Wall.

###### Names.

**Myanmar**: *khan tauk*. **English**: goldthread, Indian goldthread.

###### Range.

Temperate Asia. Grows naturally in northeastern Myanmar at altitudes exceeding 2440 m.

###### Conservation status.

Endangered [EN A2cd] (IUCN 2017).

###### Uses.

The plant’s bitter taste creates a heating sensation in the stomach. *Bark* and *Root*: Used in preparations to relieve constipation, regulate bowel movements, promote digestion, reduce fever, treat malaria, and increase vitality. *Root*: Crushed, ground together with pepper, and formed into pea-sized pellets; one pellet is taken each morning and evening to alleviate excessive phlegm, asthma, bronchitis, and coughs. A mixture of the crushed roots, ground pepper, and juice from *bauk hkway* (*Abutilon indica*) leaves is shaped into pellets the size of peppercorns; two of these pellets are swallowed twice daily to reduce edema, promote digestion, and alleviate diarrhea and other intestinal problems. The roots soaked in country liquor are taken for malaria. A thick paste formed from ground roots is used to draw circles around the eyes to remedy sore eyes and other eye problems. A mixture of the roots, crushed together with a bit of sap from *Aloe vera* leaves or sap from *mayoe* (*Calotropis procera*), is applied topically to snakebites, followed by ingestion of a second mixture, made from crushed roots combined with pepper and a bit of the tuberous roots from *ma aye chintaung* (a grass species with a triangular stem), to neutralize the venom. The root, one clove, and one peppercorn are ground into a paste using mother’s milk and given to children for pneumonia. Equal amounts of the root bark, the bark from *shwe tataing* (the scientific name of this plant could not be ascertained per Thi Thi Ta, *personal communication*), and the bark from *bauk hkway* (*A. indica*) are powdered and inhaled to alleviate asthma, bronchitis, and coughs.

###### Notes.

The medicinal uses of this species in India are discussed in Jain and DeFilipps (1991). Medicinal uses of this species in China are discussed in Duke and Ayensu (1985).

###### Reference.

Agricultural Corporation (1980).

#### 3. *Nigella* L.

##### Nigella sativa

Taxon classificationPlantaeORDOFAMILIA

L.

###### Names.

**Myanmar**: *samon-net*. **English**: black cumin, nutmeg flower, Roman coriander, small fennel.

###### Range.

Eastern Mediterranean to northeastern India; also cultivated. In Myanmar found in Kachin and Sagaing.

###### Uses.

*Seed*: Used as a carminative and galactagogue; also mixed with other drugs, since warm and stimulating.

###### Notes.

On the Malay Peninsula the seeds are a component of poultices for abscesses, rheumatism, orchitis, ulcerated nose, headache; part of a lotion to wash fever patients and a gargle; and taken internally in combination with other drugs as an antiemetic and laxative (Perry 1980). Additionally, “They are in prescriptions in the *Medical Book of Malayan Medicine* for debility, blood poisoning, enlarged liver, nausea, colic, constipation, for women after childbirth, and various other troubles.” In Indonesia, they are added to astringent medicines for abdominal disease (Perry 1980).

###### Reference.

Perry (1980).

### Rhamnaceae (Buckthorn family)

#### 1. *Gouania* Jacq.

##### Gouania leptostachya

Taxon classificationPlantaeORDOFAMILIA

DC.

###### Names.

**Myanmar**: *pi-khum*, *tayaw-nyo-nye*. **English**: liane savon.

###### Range.

China, Bhutan, India, Laos, Malaysia, Mayanmar, Nepal, Philippines, Singapore, Thailand, and Vietnam.

###### Use.

*Leaf*: Ingredients in poultices for treating sores.

###### Notes.

In Indonesia bark with water serves as a wash for the hair and kills vermin in it; pulped root, stem and leaves are applied to treat certain skin complaints (Perry 1980).

The bark and leaves of this species contain a small amount of alkaloid which has been found to have a tetanizing effect on toads (Perry 1980).

###### Reference.

Perry (1980).

#### 2. *Ventilago* Gaertn.

##### Ventilago denticulata

Taxon classificationPlantaeORDOFAMILIA

Willd. (= V. calyculata Tul.)

###### Names.

**Myanmar**: *tayaw-nyo*. **Chinese**: *mao guo yi he guo*.

###### Range.

China, Bhutan, India, Nepal, Thailand, and Vietnam. Widely distributed in Myanmar.

###### Use.

*Root*: A paste made with the root is applied to promote granulation of wounds.

###### Notes.

Seeds of this species were analyzed and found to contain protein, reducing sugars (as glucose), 40% fixed oil (oleic acid a major constituent; others included palmitic, linolenic, linoleic, lauric, stearic, and small amounts of caprylic acids), sterols, glycosides, and free acids. The unsaponfiable matter contained B-amyrin and lupeol as well as traces of two unidentified hydrocarbons (Grover and Rao 1981).

###### Reference.

Perry (1980).

#### 3. *Ziziphus* Mill.

##### Ziziphus jujuba

Taxon classificationPlantaeORDOFAMILIA

Mill. (= Z. vulgaris Lam.)

###### Names.

**Myanmar**: *eng-si*, *jujube*, *mahkaw*, *makhkaw-hku*, *zi*, *zi-daw-thi*. **English**: Chinese date, Chinese jujube, common jujube.

###### Range.

Native to temperate East Asia, also warmer climates including Indo-China (Cambodia). Cultivated in Myanmar.

###### Conservation status.

Least Concern [LC] (IUCN 2017).

###### Uses.

*Bark*: Used as a remedy for diarrhea. *Leaf*: Used for scorpion stings. *Leaf*, *Fruit*: Used as a laxative and blood purifier. *Fruit*: Considered to be pectoral. *Root*: Used for fever.

###### Notes.

Perry (1980) discusses the medicinal uses of the species in two Asian countries as follows; In Korea the stone seeds are used for hypnotics and narcotics. In China the fruits or kernel of the seeds are considered the most important part of the plant in medicine, although other parts are used as well; the fruit of the wild variety (var. spinosa) is an astringent, that of the cultivar (var. inermis) less so, but both serve the same purpose; the drug also acts as adjuvant with other drugs which are combined in medicines. The fruit is used in brewing medicines to make them less poisonous, also to modify flavor and lessen the effect of stimulants. It is also said to have nervine, tonic, roborant, stomachic, sedative, laxative, bechic, antipyretic, and diuretic properties; it relieves insomnia, night sweats, and neurasthenia, promotes hair growth, and serves as a collyrium. A decoction of the woody root is take to relieve sensation of fullness in the stomach and to aid digestion; cooked with pork, the broth is drunk as a galactagogue and used to cure hemoptysis.

The seeds of this species contain no alkaloid; the oil contains oleic, linoleic, and palmitic acids, and phytosterol (Perry 1980).

###### References.

Nordal (1963), Perry (1980).

##### Ziziphus rugosa

Taxon classificationPlantaeORDOFAMILIA

Lam.

###### Names.

**Myanmar**: *mak-kok*, *myauk-zi*, *sammankaw*, *taw-zi*, *zi-ganauk*, *zi-talaing*. **English**: wild jujube.

###### Range.

Pakistan, China, Myanmar, India, Laos, Sri Lanka, Thailand, and Vietnam. Widespread in Myanmar.

###### Use.

*Flower*: Used to treat menorrhagia.

###### Note.

In India the bark is used for diarrhea, bleeding gums, sores in the mouth and on the tongue, venereal sores, and carbuncles; the flower is employed for menorrhagia (Jain and DeFilipps 1991).

###### Reference.

Perry (1980).

### Rhizophoraceae (Red Mangrove family)

#### 1. *Carallia* Roxb.

##### Carallia brachiata

Taxon classificationPlantaeORDOFAMILIA

(Lour.) Merr.

###### Names.

**Myanmar**: *maniawga*, *hpun*, *yat.* **English**: carallia, freshwater mangrove.

###### Range.

China, South Bhutan, Cambodia, India, Laos, Malaysia, Myanmar, Philippines, Sri Lanka, Thailand, Vietnam; northern Australia, Madagascar, East Nepal, New Guinea, and Pacific islands. In Myanmar, found growing naturally all over the country, especially near rivers and streams.

###### Uses.

*Bark*: Used in medications given orally to clear eye infections; and in the prevention of pox and other infections. It is also used in thwayhsay (blood fortifying preparations) and fever-reducing remedies. Made into a paste, the bark is applied topically to relieve itching. *Fruit*: Used to treat infected wounds.

###### Notes.

The medicinal uses of this species in India are discussed in Jain and DeFilipps (1991). Perry (1980) lists the medicinal uses for this species in Indo-China and the Malay Peninsula.

###### References.

Agricultural Corporation (1980), Forest Department (1999).

#### 2. *Rhizophora* L.

##### Rhizophora mucronata

Taxon classificationPlantaeORDOFAMILIA

Lam.

###### Names.

**Myanmar**: *baing-daung*, *byu-chidauk*, *payon-ama*, *pyu.* **English**: mangrove.

###### Range.

Along coasts of the Old World tropics. In Myanmar, found in Ayeyarwady and Taninthayi.

###### Conservation status.

Least Concern [LC] (IUCN 2017).

###### Use.

*Bark*: Used to treat hematuria.

###### Notes.

In China and Japan a decoction of the bark is antidiarrheic; in Indo-China the root is antihemorrhagic, as is the bark (the latter is also a treatment for angina); on the Malay Peninsula a decoction of old leaves is given at childbirth, also of bark, at the same time giving a little of the decoction of the root to the child (Perry 1980).

###### Reference.

Perry (1980).

### Rosaceae (Rose family)

#### 1. *Agrimonia* L.

##### Agrimonia eupatoria

Taxon classificationPlantaeORDOFAMILIA

L.

###### Names.

**English**: agrimony, cocklebur, harvest-lice.

###### Range.

Mostly North Temperate Zone. In Myanmar, found in Mandalay.

###### Uses.

Plant (part unspecified in Nordal 1963) used as diuretic and astringent.

###### Note.

In India the leaf is used as an anthelmintic; the root as a diuretic, tonic, and astringent (Jain and DeFilipps 1991).

###### Reference.

Nordal (1963).

#### 2. *Prunus* L.

##### Prunus cerasoides

Taxon classificationPlantaeORDOFAMILIA

Buch.-Ham. ex D.Don (= P. puddum Roxb. ex Brandis; Cerasus cerasoides (D.Don) S.Ya.Sokolov)

###### Name.

**English**: Himalayan wild cherry.

###### Range.

Himalayas, China. Reported from Myanmar.

###### Conservation status.

Least Concern [LC] (IUCN 2017).

###### Uses.

*Seed*: kernel used as remedy for stone and gravel.

###### Note.

In India the bark is used for venereal diseases, fever, and diarrhea; the seed yields an oil used for stones and gravel (Jain and DeFilipps 1991).

###### Reference.

Perry (1980).

### Rubiaceae (Coffee family)

#### 1. *Catunaregam* Wolf

##### Catunaregam spinosa

Taxon classificationPlantaeORDOFAMILIA

(Thunb.) Tirveng. (= Randia spinosa (Thunb.) Poir.)

###### Names.

**Myanmar**: *tha-min-sa-hpru-thi*. **English**: common emetic nut, emetic nut.

###### Range.

Found from India to South China, south into southeastern Asia.

###### Uses.

*Fruit*: Used as an emetic. *Bark*: Used to treat fever.

###### Notes.

In China the root and fruit are considered emetic; on the Malay Peninsula the pericarps are used in a wash, the leaves pounded with sugar or molasses are used as an effective application for swellings, the inside of the fruit is rubbed on exposed parts of the body to ward off leeches, and the drug is put into a hot bath to treat mosquito and other bites; and in Indo-China a tea-like infusion of the bark is used to regulate menses, and water in which fruits are crushed is used to get eliminate leeches or worms if spread on the soil (Perry 1980).

Experiments have shown that the alcoholic extract contains unidentified water-soluble fatty acids, essential oil, green coloring matter, an acid saponin, and an acid resin; also, that the pharmacologically active constituent is a neutral saponin (Perry 1980).

###### Reference.

Perry (1980).

#### 2. *Coffea* L.

##### Coffea arabica

Taxon classificationPlantaeORDOFAMILIA

L.

###### Names.

**Myanmar**: *ka-phi*. **English**: Arabian coffee, Arabica coffee, coffee.

###### Range.

Northeast Tropical Africa- Ethiopia, Sudan; East Tropical Africa- Kenya. Widely cultivated in tropics, and sometimes naturalized.

###### Use.

*Seed*: Unripe seeds are used to relieve migraine headaches.

###### Notes.

The medicinal uses of this plant in the Caribbean region, as well as its chemistry, biological activity, toxicity and dosages, are discussed by Germosén-Robineau (1997). Details of the active chemical compounds, effects, herbal usage and pharmacological literature of this plant are given in Fleming (2000). Worldwide medicinal usage, chemical composition and toxicity of this species are discussed by Duke (1986). The seeds (“beans”) of *Coffea arabica* contain L-aspartic acid, a dietary amino acid which produces neuro-excitatory symptoms if ingested in large doses (Lan et al. 1998).

###### Reference.

Nordal (1963).

#### 3. *Haldina* Ridsdale

##### Haldina cordifolia

Taxon classificationPlantaeORDOFAMILIA

(Roxb.) Ridsdale (= Adina cordifolia Hook. f.)

###### Names.

**Myanmar**: *hnaw*, *yangmaw*. **English**: yellow teak.

###### Range.

Africa and Asia. In Myanmar, found in Bago, Mandalay, and Yangon.

###### Uses.

*Flower*: Buds used to treat headache, also to eliminate maggots from sores.

###### Note.

In Indo-China a decoction of the root is astringent, and is used to treat diarrhea and dysentery (Perry 1980).

###### Reference.

Perry (1980).

#### 4. *Hymenodictyon* Wall.

##### Hymenodictyon orixense

Taxon classificationPlantaeORDOFAMILIA

(Roxb.) Mabb. (= H. excelsum (Roxb.) Wall.)

###### Names.

**Myanmar**: *dumsa-gyaw*, *khu-than*, *mai-son-pu*. **English**: bridal couch tree.

###### Range.

India, Myanmar, Indo-China, Thailand, Peninsular Malaysia, Sumatra, Java, the Lesser Sunda Islands, the Philippines, Sulawesi, and the Moluccas. In Myanmar, found in Bago, Mandalay, and Yangon.

###### Uses.

*Bark*: Used as a febrifuge and tonic.

###### Notes.

In Indo-China the bark is used as tonic; also, the species apparently has two varieties- var. subglabrum Pierre, of which the pulverized wood is found in native pharmacies as a remedy for skin diseases, and var. velutinum Pierre, which is especially used as a women’s remedy (Perry 1980). In the Philippines the species is a substitute for *Cinchona* due to its antiperiodic effects, also the leaves are applied as a poultice for headache (Perry 1980).

Reported constituents include a catechol tannin containing phloraglucin, some phlobaphenes, traces of catechol tannin without phloroglucin (analogous to quinatannic acid) not combined with alkaloid, oxycoumarin, B-mannose, methyl sugar, and heteroside of which some elements could not be isolated (Perry 1980).

###### References.

Perry (1980), Forest Department (1999).

#### 5. *Ixora* L.

##### Ixora chinensis

Taxon classificationPlantaeORDOFAMILIA

Lam.

###### Names.

**Myanmar**: *pon-na-yeik*. **English**: Chinese ixora.

###### Range.

Malay Peninsula and China. In Myanmar found in Yangon.

###### Uses.

*Flower*: Used to treat tuberculosis and hemorrhage.

###### Notes.

In China the plant is used as an anodyne and resolvent; for abscesses, bruises, extravasated blood, rheumatism, wounds; also considered beneficial to bone marrow and the uterus of pregnant women (Duke and Ayensu 1985).

###### Reference.

Nordal (1963).

##### Ixora coccinea

Taxon classificationPlantaeORDOFAMILIA

L.

###### Names.

**Myanmar**: *pan-thawka*, *pan-zayeik*, *pon-na-yeik*. **English**: flame-of-the-woods, jungle flame ixora, jungle geranium, scarlet ixora.

###### Range.

South India. Cultivated in Myanmar.

###### Uses.

*Root*: Used as an appetizer and stomachic.

###### Note.

In India the root is used as a stomachic, for acute dysentery, loss of appetite, chronic ulcers, and applied on sores; the flower is used for dysentery, catarrhal bronchitis, and leucorrhea (Jain and DeFilipps 1991).

###### Reference.

Nordal (1963).

#### 6. *Mitragyna* Korth.

##### Mitragyna speciosa

Taxon classificationPlantaeORDOFAMILIA

(Korth.) Havil.

###### Names.

**Myanmar**: *bein-sa*. **English**: kratom.

###### Range.

Native to Southeast Asia. In Myanmar, found in Chin and Taninthayi.

###### Use.

*Leaf*: used to induce stupor.

###### Notes.

In Thailand chewed leaves are reputed to act as a stimulant to help person endure fatigue and long-lasting periods without food. It is also used as an opium substitute, “but is habit-forming” (Perry 1980). On the Malay Peninsula, in addition to chewing the leaves or drinking an infusion, the residue is dehydrated and smoked; all have the same effect (Perry 1980). The leaves, heated with those of *Morinda citrifolia*, *Blumea balsamifera*, and *Oroxylum indicum*, are applied hot to an enlarged spleen; pounded leaves are used as a poultice for wounds or to expel worms from children (Perry 1980).

Reported chemical constituents include mitragynine and mitraphylline; the former is said to be a local anesthetic (Perry 1980).

###### Reference.

Perry (1980).

#### 7. *Morinda* L.

##### Morinda angustifolia

Taxon classificationPlantaeORDOFAMILIA

Roxb.

###### Names.

**Myanmar**: *nlung*, *latloot*, *hla ponyork*. **English**: morinda.

###### Range.

China, India, Myanmar, and Sri Lanka. In Myanmar, found growing naturally all over the country but especially in Upper and Lower Myanmar.

###### Uses.

*Leaf*: Eating boiled leaves with a dip can help eliminate gas and cure stomachaches, burning sensation in the mouth, irregularity in bile, and high blood pressure. Eating the leaves boiled together with the *nga-mway-toh* (*Mastacembelus armatus*) fish will cure diarrhea. New mothers eating the leaves in a salad will be cured of blocked mammary glands, drying up of breast milk, aches and pains in the pelvic area, twisting pain in the abdomen, and nosebleeds. Eating the leaves in a soup with the leaves of *dant-dalun* (*Moringa oleifera*) will cure heart disease, hemorrhaging of blood, and diabetes. *Fruit*: Beaten and taken with honey will cure coughs and asthma. Eaten with jaggery will cure indigestion. Boiled young fruit and eaten in a salad will cure shooting or dull pains in the stomach due to gas, and hypertension.

###### References.

Agricultural Corporation (1980), Forest Department (1999).

##### Morinda citrifolia

Taxon classificationPlantaeORDOFAMILIA

L.

###### Names.

**Myanmar**: *nibase*, *noni*, *nyagyi*. **English**: Indian mulberry.

###### Range.

East Indies and Australia. Cultivated in Myanmar.

###### Uses.

*Leaf* and *Fruit*: Used to alleviate arthritis, as an emmenagogue, and to promote menstrual flow.

###### Notes.

Medicinal uses of this species in India are discussed in Jain and DeFilipps (1991). Indigenous medicinal uses of this species in the Andaman and Nicobar Islands (India) are discussed by Dagar and Singh (1999).

The medicinal uses of this plant in the Caribbean region, as well as its chemistry, biological activity, toxicity and dosages, are discussed by Germosén-Robineau (1997). Details of the active chemical compounds, effects, herbal usage, and pharmacological literature of this plant are given in Fleming (2000). Traditional medicinal uses, chemical constituents, and pharmacological activity of this species are discussed by Ross (2001).

###### Reference.

Nordal (1963).

##### Morinda coreia

Taxon classificationPlantaeORDOFAMILIA

Buch.-Ham. (= M. tinctoria Roxb.)

###### Names.

**Myanmar**: *nee-par hsay-pin*.

###### Range.

From India and Sri Lana to Malay Archipelago. In Myanmar, grows naturally in the hot zone and at the base of the Bago Yoma Hills.

###### Uses.

*Leaf*: Crushed and used as a poultice over sores; if the sore is newly formed, the inflammation will go down and if it is mature, it will come to a head, expel the pus and be cured. Boiled and taken to cure fever. The liquid from boiled leaves is mixed with mustard seeds and given to children suffering from dysentery. The leaves or the bark can be crushed and the resulting liquid applied to cure stiff and knotted muscles, swelling in the joints and in other painful areas. *Fruit*: Roasted, crushed with a moderate amount of salt, and used as a toothpaste, it will firm up gums and teeth. Pressing dried fruit powder to sores to stop bleeding. *Root*: Used in making laxatives.

###### Reference.

Agricultural Corporation (1980).

#### 8. *Mussaenda* L.

##### Mussaenda macrophylla

Taxon classificationPlantaeORDOFAMILIA

Wall.

###### Names.

**Myanmar**: *jyula*, *pwint-tu-ywet-tu*, *lelu*. **English**: mussaenda.

###### Range.

China, Taiwan, Nepal, Myanmar, Malaysia, and the Philippines. In Myanmar, found in Chin, Kachin, Magway, Mandalay, Sagaing, and Yangon.

###### Uses.

*Leaf*: Used to treat dysentery.

###### Notes.

Four new triterpenoid glycosides were isolated from the root bark of this species. Some of the compounds showed inhibitory activity against a periodonotpathic bacterium, *Porphyromonas gingivalis* (Kim et al. 1999). The genus *Mussaenda* is considered an important source of medicinal natural products, especially iridoids, triterpenes, and flavonoids. The phytochemestry of the species in this genus have been studied extensively since the 1990s; the most recognized of the species’compounds are the iridoids and triterpene saponins (Vidyalakshmi et al. 2008).

###### Reference.

Nordal (1963).

#### 9. *Nauclea* L.

##### Nauclea orientalis

Taxon classificationPlantaeORDOFAMILIA

(L.) L. (= Sarcocephalus cordatus (Roxb.) Miq.)

###### Names.

**Myanmar**: *ma-u*, *ma-u-gyi*, *ma-u-kadon*, *prung*. **English**: Leichhardt-pine.

###### Range.

Australasia. In Myanmar, found in Chin, Mandalay, and Yangon.

###### Uses.

*Bark*: Used as tonic, antipyretic, and for menstrual disorders.

###### Note.

Reported medicinal uses of this species include as a piscicide, tonic, and vulnerary; also for headache, fever, and tumor (Duke 2009).

###### Reference.

Nordal (1963).

#### 10. *Neolamarckia* Bosser

##### Neolamarckia cadamba

Taxon classificationPlantaeORDOFAMILIA

(Roxb.) Bosser (= Anthocephalus cadamba (Roxb.) Miq.; Anthocephalus morindifolius Korth.)

###### Names.

**Myanmar**: *hkala-shwang*, *lash-awng*, *ma-u*, *ma-u-let-tan-she*, *mau-phyu*, *prung*, *ye-ma-u*, *yema-u*. **English**: burrflower tree, cadamba, kadam tree, laran.

###### Range.

India to Indo-China south to New Guinea. In Myanmar, found in Bago, Magway, Mandalay, Sagaing, and Yangon.

###### Uses.

*Bark*: A febrifuge. *Leaf*: An ingredient of a gargle.

###### Notes.

In Indo-China the bark is tonic and bechic; on the Malay Peninsula a leaf-poultice or an oiled, heated leaf is applied to the chest or abdomen to treat fever or malaria (Perry 1980).

###### Reference.

Perry (1980).

#### 11. *Oldenlandia* L.

##### Oldenlandia corymbosa

Taxon classificationPlantaeORDOFAMILIA

L.

###### Names.

**Myanmar**: *hingalar*, *su-la-na-pha*, *su-lar-na-phar*. **English**: flat-top mille grains.

###### Range.

Pantropical.

###### Use.

*Whole plant*: febrifuge, anthelmintic, in jaundice.

###### Reference.

Nordal (1963).

#### 12. *Paederia* L.

##### Paederia foetida

Taxon classificationPlantaeORDOFAMILIA

L. (= P. scandens (Lour.) Merr.; P. tomentosa Blume)

###### Names.

**Myanmar**: *pe-bok-new*. **English**: skunk vine, stink vine, stinky opal berry.

###### Range.

Himalayas, Central and East India; Indo-China, Malayaia. In Myanmar, found in Chin, Kachin, Mandalay, Sagaing, Shan, and Yangon.

###### Uses.

*Whole plant*: In a bath. *Juice* or *Leaf*: Used as an antirheumatic, also to treat paralysis and increase fertility.

###### Notes.

In China the leaves are eaten to aid digestion and the sap, or the entire plant, is used as a remedy for poisonous insect bite;. the root (boiled with pigs’ feet) is used to aid circulation and soothe articular and muscular pains in elderly people and also used as a medicine to expel gas and treat ague; utilized in epidemics and said to have great restorative power (Perry 1980). In Japan juice from the bruised fruit is rubbed “into that portion [of the body] having cold injury”; in Indo-China the leaves are used both internally and externally to treat anuria and fever; the leaves and roots are considered to be tonic, stomachic, digestive, and aperitive and “especially are anti-inflammatory against tenesmus (Perry 1980).

###### Reference.

Perry (1980).

#### 13. *Pavetta* L.

##### Pavetta indica

Taxon classificationPlantaeORDOFAMILIA

L.

###### Names.

**Myanmar**: *myet-hna-pan*, *myet-na-myin-gyin*, *ponnayeik*, *se-baung-gyan*, *za-gwe-pan*. **English**: white pavetta.

###### Range.

India, southern China, Malay Archipelago, northern Australia. In Myanmar, found in Mandalay and Yangon.

###### Uses.

*Leaf*: Used in a fomentation. *Root*: Used as a laxative and to treat dropsy, as an aperient.

###### Notes.

The medicinal uses of this species in India are discussed in Jain and DeFilipps (1991). In Indo-China a decoction of wood chips is used to treat rheumatism, and also applied on an abscess; on the Malay Peninsula crushed leaves are made into a poultice for boils, and crushed roots for itch; the leaves also serve as a lotion for ulcerated nose (a little may be drunk) (Perry 1980). In the Philippines the bark, powdered or in a decoction, is given to correct visceral obstructions especially of children, and the decocted leaves are used externally to relieve the pain of hemorrhoids (Perry 1980).

Reported constituents of the stem include an alkaloid, essential oil, resin, tannin, pectic principle; those of the roots are resin, starch, organic acid, and a bitter glycoside resembling salicin (Perry 1980).

###### References.

Nordal (1963), Perry (1980).

#### 14. *Rubia* L.

##### Rubia cordifolia

Taxon classificationPlantaeORDOFAMILIA

L.

###### Names.

**English**: Indian madder, munjeet.

###### Range.

Southern Europe to Africa and Asia. In Myanmar, found in Chin, Magway, Mandalay, and Shan.

###### Use.

*Root*: Used as tonic.

###### Notes.

The medicinal uses of this species in India are listed in Jain and DeFilipps (1991) as follows: The leaf and stem are used in a decoction as a vermifuge; the leaf is used on ulcers; the root as an astringent, for urinary trouble, for inflammation, and for sting of poisonous insects; the root and rootstock are employed as a tonic, antidysenteric, antiseptic, and deobstruent. Medicinal uses of this species in China are discussed in Duke and Ayensu (1985). Here the root is used an anodyne, diuretic, emrhea; for arthritis, dysmenorrhea, edema, epistaxis, fractures, hematuria, rhea, hemoptysis, hemorrhoids, hemorrhage, jaundice, menorrhagia, rheumatism, and traumatic injuries; also a diuretic for bladder and kidney ailments and stones.

Duke and Ayensu (1985) also extensively discuss the chemical composition of this species. They note that the root is bacteriostatic against *Staphylococcus aureus*.

###### Reference.

Nordal (1963).

#### 15. *Spermacoce* L.

##### Spermacoce hispida

Taxon classificationPlantaeORDOFAMILIA

L. (= Borreria hispida (L.) K. Schum.)

###### Names.

**Myanmar**: *gangala*. **English**: landrina, shaggy button weed.

###### Range.

China, Hong Kong, Taiwan; Japan- Ryukyu Islands; India; Indochina, Myanmar, Thailand; Malesia. In Myanmar, found in Bago, Mandalay, Magway, and Yangon.

###### Use.

*Root*: Alterative (restores to normal health).

###### Note.

Reported medicinal uses for this species include for earache, eye problems, blindness, ophthalmia, fever, inflammation, dysentery, splenitis, otisis, pimpes, sores, stings, and gingivitis (Duke 2009).

###### Reference.

Nordal (1963).

#### 16. *Tamilnadia* Tirveng. & Sastre

##### Tamilnadia uliginosa

Taxon classificationPlantaeORDOFAMILIA

(Retz.) Tirveng. & Sastre (= Randia uliginosa (Retz.) Poir.)

###### Names.

**Myanmar**: *hman-phyu*. **English**: tamilnadia.

###### Range.

Himalayas (Garhwal to Sikkim), India, Myanmar, and Indo-China. In Myanmar, found in Ayeyarwady, Bago, and Yangon.

###### Use.

*Fruit* and *Root*: Used as a medication for dysentery.

###### Note.

The following medicinal uses have been given for this species: Astringent, deobstruent, diuretic, piscicide, tonic, and refrigerant; also used for eye problems, boils, otitis, inflammation, biliousness, colic, intestine, diarrhea, and dysentery (Duke 2009).

###### Reference.

Perry (1980).

### Rutaceae (Citrus family)

#### 1. *Aegle* Corrêa

##### Aegle marmelos

Taxon classificationPlantaeORDOFAMILIA

(L.) Corrêa

###### Names.

**Myanmar**: *hpun ja*, *kia-bok*, *mak-phyn*, *okshit*. **English**: bael tree, ball tree, bela tree, Bengal quince, golden apple, Indian bael.

###### Range.

India, Myanmar. Occasionally cultivated in tropics. In Myanmar, found in Bago, Chin, Kachin, Kayin, Magway, Sagaing, Shan, Taninthayi, and Yangon.

###### Uses.

*Fruit*: Ripe fruit diminishes phlegm and is used to treat indigestion. Also, used to regulate bowels and cure fevers. *Leaves*: Children may be treated with one tablespoon of the distillate of leaves for diarrhea, bronchitis, and mucus in the breathing passages and treated with juice from crushed leaves for intestinal worms. Juice from the crushed leaves may be used twice a day to treat fevers and coughs, used as poultice to treat sores and bumps, and drunk or applied to cure edemas. Young leaves eaten in a salad to treat bleeding from the ears. *Fruit*: Inner pulp eaten with sugar to treat severe diarrhea. Crushed pulp from ripe fruit taken with rice washing water to treat morning sickness. A drink from the pulp: used to regulate the bowels and to treat severe constipation; (or eating the leaves cooked as curry) used to treat sunstroke; and milk is used to treat bleeding gums, canker sores, and sore gums. The tender fruit, crushed together with dry ginger and stewed, is used to cure excessive urination.

###### Notes.

Medicinal uses of this species in China are discussed in Duke and Ayensu (1985).

###### References.

Agricultural Corporation (1980), Forest Department (1999).

#### 2. *Citrus* L.

##### Citrus aurantiifolia

Taxon classificationPlantaeORDOFAMILIA

(Christm.) Swingle

###### Names.

**Myanmar**: *thanbayar*, *lawihkri-shalwai* (Kachin), *sot-parite-sanut* (Mon), *maksun-ting* (Shan). **English**: key lime, lime, Mexican lime.

###### Range.

India and Southeast Asia. Found throughout Myanmar as a cultivar.

###### Uses.

*Bark*: Boiled in water to half the starting volume, and taken once in the morning and once in the evening to reduce fever. *Fruit*: The sour fruit is used to stimulate the appetite and aid digestion, as well as to control vomiting, coughing, sore throat, asthma, and bloating. Fresh lime juice is consumed to alleviate vomiting and fatigue; it is also squeezed into the nostrils to stop bloody noses and taken to protect against diseases, especially those that affect the stomach. Lime juice taken with added sugar is used as a remedy for coughing due to too much fat, weak bile, and aches and pains in the joints. Lime juice with a small amount of sugar is taken twice daily, in the mornings and evenings, to cure bleeding gums. A paste made from crushing together the fruit, charred from roasting over hot coals with one clove, is applied to the base of the teeth for toothaches. Consumption of great volumes of the juice mixed with small amounts of sugar is considered a cure for opium overdoses, alcohol toxicity, and food poisoning. Lime juice mixed with ash from baked cowry shells (*Cypraea tigris*) is taken as a remedy for difficulty and pain in passing urine. Hot lime juice mixed with honey is taken twice daily to alleviate sore throats. Drinking lime juice every day is considered a cure for dizziness that occurs upon sitting or standing. As a very strong tea, lime juice is taken as a remedy for headaches. The fruit’s green skin is ingested to relieve chest and stomach pains. The fruit can be sliced in half and applied to the skin as a cure for ringworm, discoloration, hair loss, itching, and rashes. Lime pickle (after slightly dried, fruit preserved in oil and spices such as cumin, coridander, and mustard seed) ingested regularly after meals is considered a cure for inflammation of the spleen. *Seed*: Crushed and rubbed onto the temples to treat headaches affecting only one side of the head.

###### Notes.

The oil in the peel of limes, i.e., oil of bergamot, contains psoralen, a chemical which can cause phototoxic reactions such as blistering and burning of human skin when exposed to sunlight after eating limes, affecting areas around a person’s chin, cheeks, and chest. Oil of bergamot is used in Egypt as a folk remedy for vitiligo, a skin disease causing loss of skin pigment, and it is currently being investigated for its ability to remedy severe psoriasis (Martin 1993). The medicinal uses of this plant in the Caribbean region, as well as its chemistry, biological activity, toxicity and dosages, are discussed by Germosén-Robineau (1997). Data on the propagation, seed treatment and agricultural management of this species are given by Katende et al. (1995) and Bekele-Tesemma (1993).

###### Reference.

Agricultural Corporation (1980).

##### Citrus × aurantium

Taxon classificationPlantaeORDOFAMILIA

L.

###### Names.

**Myanmar**: *lein-maw*. **English**: bitter orange, Seville orange, sour orange.

###### Range.

South Vietnam. Cultivated in Myanmar.

###### Use.

*Fruit*: Used as a digestive.

###### Notes.

The medicinal uses of this plant in the Caribbean region, as well as its chemistry, biological activity, toxicity and dosages, are discussed by Germosén-Robineau (1997). Details of the active chemical compounds, effects, herbal usage, and pharmacological literature of this plant are given in Fleming (2000).

In the classification of this species espoused by Mabberley (1997), the sour orange or Seville orange is in *Citrus aurantium* Sour Orange Group; the sweet orange is in *Citrus aurantium* Sweet Orange Group; and the grapefruit is in *Citrus aurantium* Grapefruit Group, whereas in the current treatment we have retained a traditional arrangement in which the sour orange or Seville orange is recognized as *Citrus aurantium*; the sweet orange as *Citrus sinensis*; and the grapefruit as *Citrus paradisi*.

###### Reference.

Nordal (1963).

##### Citrus limon

Taxon classificationPlantaeORDOFAMILIA

(L.) Osbeck (= C. medica var. limon L.)

###### Names.

**Myanmar**: *than-bayo*, *shauk*, *shauk-waing*, *hla-parite-baikayah* (Mon). **English**: lemon.

###### Range.

Southeast Asia. Cultivated in Myanmar.

###### Uses.

*Fruit*: These sour fruits are thought to “clear the heart and cleanse the blood”, aid digestion, alleviate fatigue, inhibit formation of bumps and tumors, control coughs, stimulate appetite, relieve nausea, and remedy laryngitis. Epilepsy is believed to be cured by inhaling a mixture of equal amounts of the fruit juice and leaves of *kyaung-pan* (*Vitex trifolia*). Fruit segments mixed with sour pomegranate sap are ingested to treat dizziness and feelings of heaviness or dullness. Fruit segments are eaten with rock salt in the mornings and evenings to alleviate kidney stones. A mixture of the juice with honey and *zawet-thar* (*Dillenia indica*) is taken for coughs, asthma, and bronchitis. A mixture of the fruit together with jaggery is taken for dizziness and weakness during menstruation. To make a medicine for gas, the fruit can be boiled in one viss (~1.6 kg) of rice washing water until the liquid has evaporated and the fruit is tender. After filtering through a sieve, about 10 ticals (~ 0.1 kg) of the pulp can be mixed with a small amount of salt, dried in the sun, crushed into a powder, and ingested.

###### Notes.

Indigenous medicinal uses of this species in the Andaman and Nicobar Islands (India) are described by Dagar and Singh (1999).

The lemon is possibly a hybrid (backcross) between lime and citron (Swingle 1943, Mabberley 1997). Data on the propagation, seed treatment and agricultural management of this species are given by Katende et al. (1995). Details of the active chemical compounds, effects, herbal usage, and pharmacological literature of this plant are discussed in Fleming (2000).

###### Reference.

Agricultural Corporation (1980).

#### 3. *Clausena* Burm.f.

##### Clausena excavata

Taxon classificationPlantaeORDOFAMILIA

Burm.f.

###### Names.

**Myanmar**: *daw-hke*, *pyin-daw-thein*, *seik-nan*. **English**: clausena.

###### Range.

Asia, Australia, and tropical South Africa. Widely distributed in Myanmar.

###### Uses.

Plant considered a good remedy for stomach trouble. *Leaf*: Bitter and astringent, promotes good digestion. Used to treat diseases caused by “abnormal blood”. A drink of milk in which the leaves were stewed used to neutralize poisons. Leaves also used in making up carminatives and to control leprosy. *Root*: Used as an antispasmodic.

###### Notes.

The medicinal uses of this species in China are discussed in Duke and Ayensu (1985). In India the stem is used as diuretic and for digestion (Jain and DeFilipps 1991). In Taiwan a decoction of the root is sudorific and the leaves are insecticidal (Perry 1980). In Indo-China the plant is used as a tonic, astringent, and emmenagogue; a poultice of the leaves is applied to treat paralysis; and an infusion of the stem (roots, or the flowers and leaves) is taken for colic (Perry 1980). On the Malay Peninsula the pounded root is used as a poultice for sores; the leaves are employed for headache and ulcerated nose (for the latter, fumigation from burning leaves and bark is another treatment), and a decoction of the leaves is administered post partum; in Indonesia the juice, pressed or pounded out of the leaves, is used both as a medication for fever and a vermifuge, and may be given to “lying-in” women (Perry 1980).

###### References.

Nordal (1963), Agricultural Corporation (1980), Perry (1980), Forest Department (1999).

#### 4. *Limonia* L.

##### Limonia acidissima

Taxon classificationPlantaeORDOFAMILIA

L.

###### Names.

**Myanmar**: *kwet*, *mak-pyen-sum*, *thi*, *san-phak* (Kachin), *sanut-khar* (Mon), *sansph-ka*, *thanakha*, *thi-ha-yaza*. **English**: Chinese box tree, elephant apple, wood apple.

###### Range.

Widely distributed on all continents. In Myanmar, grows naturally in hot zone, in townships such as Pakokku, Myin-kyan, Pyay, Shwe-bo, Sagaing, Myaing, Nwa-hto-gyi, and Taungthar. Can also be found in some of the semi-desert dry and scrubby areas of Upper Myanmar.

###### Uses.

*Bark*: Used as a medication for biliousness. *Leaf*: Considered to be carminative. Used in treating epilepsy. Patients bathed in water the leaves have been boiled in and this is followed up by inducing a sweat. Leaves dried and made into a powder used to cure edema, sores and other diseases. *Fruit*: Considered to be stomachic. Used in making medicine for neutralizing poisons, strength-giving tonics, and high fevers. *Root*: Used in laxatives and medicines to induce sweating. Used as a purgative. Paste made of root, along with tumeric, used to treat female related disorders. Paste with salt used for tired sore muscles. Paste, together with water in which betle (*Piper betle*) leaves have been soaked, given to children with bronchitis. Licking 3 ticals (c. 30 g) of root powder mixed with sugar and honey used to neutralize toxins in the stomach. Taking 5 pei (1/16^th^ tical) each of the root and *pan-nu* (*Hemistrepta lyratat* or *Saussurea affinis*) used to neutralize the venom of snakebites. *Fruit*: Tonic.

###### Notes.

In Indo-China the ripe fruit is cooling, astringent, tonic, “very efficacious” to treat salivation and ulcers in the mouth; a decoction of the aromatic leaves is taken as stomachic and carminative; the bark, chewed with that of *Barringtonia acutangula*, is applied to bites and stings, and also used to treat nausea; an infusion of the thorns with other ingredients is ingested as hemostatic to treat metrorrhagia (Perry 1980).

Marmosin has been isolated from the bark, feronialactones from the bark and roots, bergapten from the leaves, and stigmasterol from the leaves and unripe fruits (Perry 1980).

###### References.

Agricultural Corporation (1980), Perry (1980).

#### 5. *Zanthoxylum* L.

##### Zanthoxylum acanthopodium

Taxon classificationPlantaeORDOFAMILIA

DC.

###### Names.

**Myanmar**: *chy-inbawngla*, *jangbawngla*, *jingbawngla*, *lan-salat*, *tabu*. **English**: Japanese pepper.

###### Range.

China, Bangladesh, Bhutan, India, Indonesia, Laos, Malaysia, Myanmar, Nepal, Thailand, and Vietnam. In Myanmar, found in Bago, Chin, kachin, Magway, Sagaing, Shan, and Yangon.

###### Uses.

*Seed*: Used as febrifuge and sudorific.

###### Notes.

In China the fruit is used for dysentery and stomachache; the seed as a sudorific, febrifuge, and for tooth powder. Medicinal uses if this species in China are discussed in (Duke and Ayensu 1985).

###### Reference.

Nordal (1963).

### Salicaceae (Willow family)

#### 1. *Flacourtia* Comm. ex L’Her.

##### Flacourtia jangomas

Taxon classificationPlantaeORDOFAMILIA

(Lour.) Rausch. (= F. cataphracta Roxb. ex Willd.)

###### Names.

**Myanmar**: *kyetyo-po*, *mak-kyen*, *naywe*. **English**: puneala plum.

###### Range.

Old World tropics. Sub-himalayan foot hill zone in India extending to southeastern Asia, China. “… not known in the wild state, but is cultivated around villages in tropical countries of SE Asia (occasional in Java, Sumatra, Borneo, and Luzon)” (Perry 1980). In Myanmar found in Mandalay, Taninthayi, and Yangon.

###### Uses.

*Leaf*: Used for stomatitis, diaphoretic; *Fruit*: Used for nausea and biliousness.

###### Notes.

Medicinal uses of this species in India are discussed in Jain and DeFilipps (1991) as follows: The bark is used as a prenatal and postnatal treatment for women to purify the blood (with roots of two other plants); the fruit is used for biliousness and liver complaints. Perry (1980) discusses the uses of this species in Indo-China and the Malay Peninsula.

###### References.

Nordal (1963), Perry (1980).

#### 2. *Salix* L.

##### Salix tetrasperma

Taxon classificationPlantaeORDOFAMILIA

Roxb.

###### Names.

**Myanmar**: *hkamari*, *mai-hkai*, *mai-keik*, *mangrai*, *momakha*, *tnhlium*, *yene*, *ye-thabye*. **English**: willow.

###### Range.

China, India, Indonesia, Malaysia, Myanmar, Pakistan, Philippines, Thailand, and Vietmam. In Myanmar, found in Bago, Kachin, Mandalay, and Sagaing.

###### Use.

Plant used as a febrifuge (no specific part given in Perry 1980).

###### Note.

On the Malay Peninsula a cold decoction of the leaves is used as a lotion for an ulcerated nose (Perry 1980).

###### Reference.

Perry (1980).

### Santalaceae (Sandalwood family)

#### 1. *Santalum* L.

##### Santalum album

Taxon classificationPlantaeORDOFAMILIA

L.

###### Names.

**Myanmar**: *nanttha hpyu*, *natha hpyu*, *sandakoo*, *santagu*, *mawsanku* (Shan). **English**: Indian sandalwood, sandalwood, true sandalwood, white sandalwood.

###### Range.

Tropical Asia and Australasia. Grows throughout Myanmar where annual precipitation is 63.5–89 cm and temperatures range between 10–32 degrees Celsius, at altitudes of 610–915 m. Brought to Myanmar from India; cultivated in Yangon, around the Kaba Aye pagoda, in Pyin Oo Lwin and around the base of Mount Popa.

###### Conservation status.

Vulnerable [VU A1d] (IUCN 2017).

###### Uses.

*Oil*: A mixture of the oil and lime juice is applied topically to relieve itching. *Wood*: Used in treatment of gonorrhea. *Inner wood*: A paste made from the inner wood- mixed with menthol is applied topically to the head for high fevers and hot water burns on the body, as well as to the limbs to ease fatigue, aches, and pains; mixed with rice washing water, honey, and sugar the paste is given to alleviate pain during urination and diarrhea.; made with water or rosewater, and mixed with coriander seeds, it is used for flaky scalp conditions and for impetigo; and made with rice washing water mixed with rock candy, it is given to relieve hiccups.

###### Notes.

The medicinal uses of this species in India are discussed in Jain and DeFilipps (1991). Medicinal uses of this species in China are discussed in Duke and Ayensu (1985). Perry (1980) discusses the species medicinal uses in Indonesia, China, and Korea.

###### References.

Nordal (1963), Agricultural Corporation (1980), Forest Department (1999).

#### 2. *Viscum* L.

##### Viscum cruciatum

Taxon classificationPlantaeORDOFAMILIA

Sieber ex Boiss.

###### Names.

**Myanmar**: *kyibaung*, *taung-kyibaung*. **English**: mistletoe.

###### Range.

In Europe, northern Asia, and northern Africa. In Myanmar, found in Ayeyarwady, Magway, and Shan.

###### Use.

*Leaf*: In Upper Myanmar, leaves are powdered and a paste is made for use in a local antiphylogistic application.

###### Notes.

In India the whole plant is used for “puss formation”; from the leaf a poultice is made for neuralgia, and ash is placed on itching skin (Jain and DeFilipps 1991). In Indo-China young children are bathed in a decoction of the plant to treat fevers (Perry 1980).

###### Reference.

Perry (1980).

### Sapindaceae (Soapberry family)

#### 1. *Cardiospermum* L.

##### Cardiospermum halicacabum

Taxon classificationPlantaeORDOFAMILIA

L.

###### Names.

**Myanmar**: *kala-myetsi*, *malame*, *moot maiboa* (Mon). **English**: balloon vine, heart’s pea, heart-seed, winter cherry.

###### Range.

Pantropical.

###### Uses.

*Whole plant*: Used to treat rheumatism and fever, as well as tumors. Boiled in water to one-third the starting volume, and the resulting decoction taken with sugar to cure urinary tract disorders and diseases, as well as laryngitis, fever, aches and pains. Liquid from boiling the plant and jaggery cooled, a cloth bundle of five kinds of fennel soaked in the liquid, and roasted salt added; the resulting preparation is taken three times a day for urinary diseases, indigestion and gas, eye disorders, heart disease, uterine ailments, edema, muscle fatigue and aches, throat problems (possibly cancer), and weakness. *Shoot* and *Leaf*: Boiled and eaten as a diuretic. *Leaf*: Decoction ingested as a remedy for rheumatism or applied in an oil as an embrocation. Most uses of the leaves are external. Juice from the crushed leaves applied around the eyes or mixed with mother’s milk and used as eye drops to treat eye disorders caused by anemia, sore eyes, and cataracts. Juice from the crushed leaves is also used to make thanakha, a paste applied to the face and body to alleviate skin disorders, such as ringworm, discoloration, and acne, as well as rashes related to menstrual irregularities. Equal amounts of powder from the dried leaves and garlic clove are mixed into a paste that is rolled, dried in the sun, and used as an inhalant to clear nasal passages; it is also rubbed on the tongue and inside the mouth to heal sores, to alleviate problems caused by eating the wrong foods or from inhaling cooking fumes, and to treat bronchitis. In addition, the same preparation is dissolved in sesame oil and applied topically as a remedy for skin disorders, such as scabies and eczema, edema, varicose veins, anemia, chills, and fever, as well as for thrush, indigestion, and bloating in infants. *Root*: Employed as a laxative, diuretic, emetic, purgative, and diaphoretic; also administered to treat catarrh of the bladder and urinary tract.

###### Notes.

Medicinal uses of this species in India are discussed in Jain and DeFilipps (1991). Medicinal uses of this species in China are discussed by Duke and Ayensu (1985).

###### References.

Nordal (1963), Agricultural Corporation (1980), Perry (1980).

#### 2. *Dimocarpus* Lour.

##### Dimocarpus longan

Taxon classificationPlantaeORDOFAMILIA

Lour. (= Euphoria longana Lam.)

###### Names.

**Myanmar**: *ga-naing-gyo*, *longan*, *taw-kyetmauk*, *taw-longan*, *tayok-kyetmauk*. **English**: eyeball tree, logan.

###### Range.

East Asia; cultivated elsewhere. In Myanmar, found in Bago, Mandalay, Mon, and Shan.

###### Conservation status.

Lower Risk/near threatened [NT] (IUCN 2017).

###### Use.

*Fruit*: Used as a brain stimulant.

###### Notes.

In China the fleshy part of the fruit is used as a nutrient-roborant, benefiting the spleen, heart, kidneys, lungs, and mental faculties, and is also employed as an antidote and anthelmintic; the powdered kernel is used as a styptic (Perry 1980). In Indo-China the seed as an alexiteric, and oil from the seed is used on snakebites;. an infusion of dried flowers is used for kidney trouble and leucorrhea, that of the sliced roots to treat gonorrhea and glycosuria; the fresh dried aril is licked to stop hiccups (Perry 1980).

###### Reference.

Nordal (1963).

#### 3. *Dodonaea* Mill.

##### Dodonaea viscosa

Taxon classificationPlantaeORDOFAMILIA

(L.) Jacq.

###### Names.

**Myanmar**: *hmaing*. **English**: hopseed, hopseed bush.

###### Range.

Arizona to South America, West Indies, and widely distributed in the Old World Tropics. In Mayanmar, found in Ayeyarwady, Rakhine Taninthayi, and Yangon.

###### Use.

*Leaf*: Used in fomentations.

###### Notes.

In Taiwan and Palau the leave are used as a remedy for fever; in the Philippines a decoction of the bark serves as an astringent applied to treat eczema and simple ulcers, also used as a febrifuge (Perry 1980).

The leaves have been found to contain an alkaloid, glucoside, tannin, and resins (Perry 1980).

###### Reference.

Perry (1980).

#### 4. *Litchi* Sonn.

##### Litchi chinensis

Taxon classificationPlantaeORDOFAMILIA

Sonn.

###### Names.

**Myanmar**: *kyetmauk*, *tayok-zi*, *wa-mayar.* **English**: leechee, litchi, litchi nut, lychee.

###### Range.

South China, Cambodia, Vietnam, and Philippines. Cultivated in Myanmar.

###### Uses.

*Fruit*: Heart, brain, and liver tonic. Also used as antidote in opium poisoning.

###### Notes.

Medicinal uses of this species in India are discussed in Jain and DeFilipps (1991). Medicinal uses of this species in China are discussed by Duke and Ayensu (1985). *Litchi chinensis* is reported to be used as a tonic, analgesic, anodyne, antitussive, and astringent; also for thirst, stomachache, adenopathy, anemia, angina, cancer, colic, diarrhea, eruptions, flux, gastralgia, gastritis, hernia, intestinal problems, neuralgia, orchitis, quinsy, smallpox, and tumor (Duke 2009).

###### Reference.

Nordal (1963).

#### 5. *Sapindus* L.

##### Sapindus saponaria

Taxon classificationPlantaeORDOFAMILIA

L. (= S. mukorossi Gaertn.)

###### Names.

**Myanmar**: *magyi-bauk*. **English**: false dogwood, jaboncillo, soapberry, soapnut.

###### Range.

Tropical America, North India. In Myanmar, found in Magway.

###### Uses.

*Fruit*: Used as treatment for epilepsy. *Fruit* and *Seed*: Used to treat skin diseases.

###### Notes.

In areas of the world where the plant is present, the fruit is used as soap (Perry 1980). In India the fruit is used an emetic and expectorant, for epilepsy, excessive salivation, and chlorosis; in China and Taiwan the flowers are a used for conjunctivitis and other eye diseases, a lotion made from the nuts is said to cause freckles and tan to disappear, the kernel is used to correct fetid breath and gum boils as well as to prevent tooth decay, a solution of macerated bark is used to wash the hairy parts of the body to kill lice and other vermin, and the seeds serve as a laxative and a decoction is taken as an expectorant (they are also used as a fish poison and insecticide) (Perry 1980). The medicinal uses of this species in India are discussed in Jain and DeFilipps (1991). Medicinal uses of this species in China are discussed in Duke and Ayensu (1985).

A 22% physiologically active saponin has been extracted from the plant. The fruit is the soap nut, containing a toxic saponin (Perry 1980).

###### References.

Nordal (1963), Perry (1980).

#### 6. *Schleichera* Willd.

##### Schleichera oleosa

Taxon classificationPlantaeORDOFAMILIA

(Lour.) Merr.

###### Names.

**Myanmar**: *gyo*, *mai-hkao*, *mai-kyang*, *thakabti*, *yun-ha*. **English**: Ceylon oak, gum-lac, lac tree.

###### Range.

Widespread from tropical and subtropical Asia to Australia. Widely distributed in Myanmar.

###### Uses.

*Bark*: An astringent. *Seed*: Oil a hair growth promoter.

###### Notes.

In Indo-China, used in a maceration or infusion, the bark is said to be anti-malarial; also used as a dressing for adenitis and immature boils, and made into a paste with rice water and powdered gypsum for spreading on lesions (Perry 1980). In Indonesia the bark is used as a for itch, wounds, and as a stimulating agent for cleansing the scalp and promoting hair growth (Perry 1980).

The seeds are more than half oil, in which a small part of prussic acid is found (Perry 1980).

###### Reference.

Perry (1980).

### Sapotaceae (Sapodilla family)

#### 1. *Manilkara* Adans.

##### Manilkara zapota

Taxon classificationPlantaeORDOFAMILIA

(L.) P. Royen (= Achras zapota L.)

###### Names.

**Myanmar**: *thagya*. **English**: chewing gum tree, chicle tree, sapodilla.

###### Range.

Central America. Cultivated in Myanmar.

###### Uses.

*Bark* and S*eed*: Used as a diuretic, tonic, and antipyretic.

###### Notes.

Medicinal uses of this species in India are discussed in Jain and DeFilipps (1991). Indigenous medicinal uses of this species in the Andaman and Nicobar Islands (India) are described by Dagar and Singh (1999).

Juice of the leaves and young fruits of *M. zapota* contain a saponin which, when ingested, causes diarrhea and mild skin irritation (Lan et al. 1998).

###### Reference.

Nordal (1963).

#### 2. *Mimusops* L.

##### Mimusops elengi

Taxon classificationPlantaeORDOFAMILIA

L.

###### Names.

**Myanmar**: *thitcho-khaya*, *khayay pin*, *chayar pin*, *sot-keen* (Mon). **English**: Spanish cherry, star flower tree.

###### Range.

Tropical. India, Malay Peninsula and Archipelago. Grows naturally around Myanmar; also cultivated.

###### Uses.

*Bark*: Liquid from boiling the bark together with the bark of *zee-hpyu* (*Phyllanthus emblica*) and *shah* (or *A. chundra*) is held in the mouth to treat thrush, inflamed gums, burns within the mouth, gingivitis, and other gum disorders. Liquid from boiling the bark is also used to clean cuts and wounds. *Bark, Flower* and *Fruit*: Used for heart problems, a decoction of the bark is taken, the flowers are inhaled, and the fruit is eaten. *Flower*: Fresh flowers are used for treating white vaginal discharge and dental diseases. Water from soaking them overnight is given to children for coughs. Dried flowers, ground together with *thanakha* (paste of bark of *Chloranthus erectus*, especially useful for its astringent properties), are applied to cure heat rashes and prickly heat. *Fruit* and *Seed*: Paste of seeds is made with cold water or the ripe fruits are ingested for persistent diarrhea.

###### Note.

The medicinal uses of this species in India are discussed in Jain and DeFilipps (1991).

###### Reference.

Agricultural Corporation (1980).

#### Scrophulariaceae (Snapdrgon family)

##### 1. *Buddleja* L.

###### Buddleja asiatica

Taxon classificationPlantaeORDOFAMILIA

Lour.

####### Names.

**Myanmar**: *kyaung-migo*. **English**: dogtail, white butterfly bush.

####### Range.

West Pakistan and central India to southern China, Taiwan, south to the Malay Archipelago and the Mariana Islands.

####### Uses.

*Leaf*: Used as an abortifacient and to treat skin diseases.

####### Notes.

This species is used as an abortifacient and intoxicant; for dermatosis, inflammation, malaria; and to treat tumors (Duke 2009). Where native, it is also used as a fish poison (Bailey and Bailey 1976).

####### Reference.

Nordal (1963).

#### Simaroubaceae (Tree of Heaven or Quassia family)

##### 1. *Eurycoma* Jack

###### Eurycoma longifolia

Taxon classificationPlantaeORDOFAMILIA

Jack

####### Name.

**English**: bittu bark.

####### Range.

Myanmar, Thailand, Indo-China, south into Indonesia. In Myanmar found in Kayin and Taninthayi.

####### Uses.

*Bark*: Very bitter, used for indigestion and as a vermifuge. *Fruit*: Antidysenteric.

####### Notes.

Medicinal uses of this species in Indo-China, where the native name of the tree is “tree of 100 maladies”; Vietnam, where it is “much used in the Vietnamese pharmacopeia”; Cambodia; and the Malay Peninsula are discussed in Perry (1980). The speices has been reported as used for headache, fever, malaria, parturition, smallpox, sores, syphilis, and wounds (Duke 2009).

####### Reference.

Perry (1980).

##### 2. *Picrasma* Blume

###### Picrasma javanica

Taxon classificationPlantaeORDOFAMILIA

Blume

####### Names.

**Myanmar**: *taung-kamaka*. **English**: quassia wood.

####### Range.

Distributed in tropical southeastern Asia as far as the Solomon Islands. Widely distributed in Myanmar.

####### Uses.

*Bark*: On account of the bitterness of quassin in the bark, it has been substituted for quinine in Myanmar. *Leaf*: Applied to festering sores.

####### Notes.

The species is reported to be used as an antidote and larvicide; also for dyspepsia and fever (Duke 2009). Perry (1980) discusses the medicinal uses of this species in East and Southeast Asia.

####### Reference.

Perry (1980).

##### 3. *Quassia* L.

###### Quassia indica

Taxon classificationPlantaeORDOFAMILIA

(Gaertn.) Noot. (= Samadera indica Gaertn.)

####### Names.

**Myanmar**: *le-seik-shin*, *kame*, *theban*. **English**: bitterwood, neepa bark, Rangoon creeper.

####### Range.

From Myanmar and Indo-China to the Solomon Islands, but not in Sumatra, Java, and the Lesser Sunda Islands; also cultivated. In Myanmar, found in Taninthayi.

####### Uses.

*Bark*: Utilized against fever. *Leaf*: Serves as a remedy for erysipelas. *Fruit*: Used to treat rheumatism.

####### Notes.

In Indonesia the bark, wood, and seeds serve as a febrifuge and tonic, and a decoction is prescribed for bilious fever; the seed, chewed or ground with water, is both emetic and purgative, and oil from the seeds is a constituent in an embrocation for rheumatism; leaves are crushed and applied to erysipelas, also an infusion of the leaves is used to kill insects, especially white ants (Perry 1980). In the Philippines the bark and wood, macerated in water, alcohol, or wine are said to have tonic, stomachic, anticholeric, antifebrile, and emmenagogue properties; juice from the pounded bark serves as a remedy for skin diseases, and the bark, scraped or powdered, is given in water or oil to treat “malignant fever” (Perry 1980). In the Solomon Islands water from the macerated bark is drunk as a remedy for constipation; macerated leaves mixed with coconut oil are applied to the hair to kill lice; and an infusion of the seeds is utilized as a febrifuge (Perry 1980).

The bitter principle is samaderin (Perry 1980).

####### Reference.

Perry (1980).

#### Smilacaceae (Catbrier family)

##### 1. *Smilax* L.

###### Smilax aspera

Taxon classificationPlantaeORDOFAMILIA

L.

####### Names.

**English**: catbrier, greenbrier.

####### Range.

Southern Europe to Asia in the Himalayas. In Myanmar, found in Chin, Kachin, and Shan.

####### Uses.

*Root*: Used as an emetic and diaphoretic.

####### Note.

In India the root is used on skin eruptions; also as a substitute for Indian sarsaparilla (*Hemidesmus* sp.) (Jain and DeFilipps 1991).

####### Reference.

Nordal (1963).

###### Smilax glabra

Taxon classificationPlantaeORDOFAMILIA

Roxb.

####### Names.

**Myanmar**: *katcho-gyi*. **English**: glabrous greenbrier.

####### Range.

Eastern Asia - China to the Himalayas. In Myanmar, found in Bago, Mandalay, and Taninthayi.

####### Uses.

*Root*: Used to treat venereal diseases.

####### Notes.

In India fresh roots are decocted for sores and venereal diseases (Jain and DeFilipps 1991). In China the aerial tuber, boiled in water, is used for abscesses, arthritis, boils, cystitis, diarrhea, dyspepsia, furuncles, lymphadenopathy, rheumatism, and syphilis; the rhizome is used to treat cancer, as well as for mercury poisoning, syphilis, and acute bacterial dysentery (Duke and Ayensu 1985). This species’ usage is sometimes confused with another species, *Smilax china*. In East and Souteast Asia the rhizome of *S. glabra* is used as an antidote for mercury poisoning; also to treat gout, scrofula, frambesia, and menorrhagia; a decoction is given as a parturifacient; additionally, the tubers are imported to the Malay Peninsula for treating venereal diseases (Perry 1980).

The plant is said to contain the antitumor hormones, beta-sitosterol and stigmasterol, and the tubers are nearly 70% starch; also, alcohol extracts contain a glucoside (Duke and Ayensu 1985).

####### Reference.

Nordal (1963).

###### Smilax guianensis

Taxon classificationPlantaeORDOFAMILIA

Vitman (= S. macrophylla Willd.)

####### Names.

**Myanmar**: *katcho*, *ku-ku*. **English**: wild sarsaparilla.

####### Range.

Throughout India, Myanmar, Malaya, and Sri Lanka.

####### Uses.

*Root*: Used as an emetic and diaphoretic; also to treat venereal disease.

####### Notes.

In India and Nepal, the root is used as a substitute for sarsaparilla in the treatment of syphilis and gonorrhea. Also, a decoction of the root is given for swellings, abscesses, and boils (Nadkarni 1976).

####### Reference.

Nordal (1963).

#### Solanaceae (Nightshade family)

##### 1. *Brugmansia* Pers.

###### Brugmansia arborea

Taxon classificationPlantaeORDOFAMILIA

(L.) Steud.

####### Name.

**English**: maikoa.

####### Range.

Andes (3050 – 3655 m), central Ecuador to northern Chile. In its natural range will not grow at low elevations. Cultivated in Myanmar.

####### Conservation status.

Extinct in the Wild [EW] (IUCN 2017).

####### Uses.

*Leaf*: Used as sedative and antiasthmatic.

####### Notes.

Duke (2009) reports that this species is used for treating asthma, pain, and tumor; and is used as a suppurative and fumitory; also, as an intoxicant, narcotic, poison, and psychedelic.

####### Reference.

Nordal (1963).

###### Brugmansia suaveolens

Taxon classificationPlantaeORDOFAMILIA

(Humb. & Bonpl. ex Willd.) Bercht. & J.Presl

####### Names.

**Myanmar**: *padaing*. **English**: angel’s trumpet, bell bush.

####### Range.

South America. Cultivated in Myanmar.

####### Conservation status.

Extinct in the Wild [EW] (IUCN 2017).

####### Uses.

*Leaf*: Used as a sedative and an antiasthmatic.

####### Notes.

In Dominica, it has been observed that the dried flowers, smoked in cigarettes, are hallucinogenic (Adjanohoun et al. 1985). Juice of *Brugmansia suaveolens* is the strongest hallucinogen used by the Shuar Jivaroan group of indigenous people in Amazonian Ecuador and Peru, who employ it to communicate with the spirits, and also use it medicinally to remedy menstrual pain, and against infections and weakness (Bennett 1992). Uses of “tree datura” (*Brugmansia*) species, cited as *Datura candida* (Persoon) Safford and *Datura sanguinea* Ruiz & Pavon, for medicinal and psychotropic (hallucinogenic, narcotic) purposes among Amerindians in the Valley of Sibundoy, Colombia are discussed by Bristol (1969, cf. Schultes 1981).

Leaves and fruits of *Brugmansia suaveolens* contain hyoscyamine which is highly toxic, anticholinergic, and used to treat motion sickness and induce anaesthesia; and also contain atropine, a highly toxic anticholinergic substance which causes delirium, blurred vision, vasodilation and suppressed salivation (Lan et al. 1998). Plants derived from cultivated stock of *Brugmansia suaveolens* are not known to set fruit; the leaves are very poisonous (Witherell 2001).

####### Reference.

Nordal (1963).

##### 2. *Capsicum* L.

###### Capsicum annuum

Taxon classificationPlantaeORDOFAMILIA

L. (= C. frutescens L.)

####### Names.

**Myanmar**: *ngayok*. **English**: bell pepper, cayenne pepper, chili pepper, hot pepper, red pepper, tabasco.

####### Range.

New World tropics. Cultivated in Myanmar.

####### Uses.

*Fruit*: Used as a rubefacient and hot spice.

####### Notes.

Worldwide medicinal usage, chemical composition, and toxicity of this species are discussed by Duke (1986). Medicinal uses of this species in India are discussed in Jain and DeFilipps (1991). Chemical constituents, pharmacological action, and medicinal uses of *Capsicum annuum* in Indian Ayurveda are discussed in detail by Kapoor (1990). Indigenous medicinal uses of this species (as dual entries *Capsicum annuum* and *Capsicum frutescens*) in the Andaman and Nicobar Islands (India) are described by Dagar and Singh (1999).

The medicinal uses of this plant in the Caribbean region, as well as its chemistry, biological activity, toxicity, and dosages, are discussed by Germosén-Robineau (1997).

The chemistry, pharmacology, toxicology, and use of this species (as *Capsicum frutescens*) for a hunting poison and medicinal plant in Africa are discussed by Neuwinger (1994). A pharmacognostical profile including medicinal uses of this plant (as *Capsicum annuum* and *Capsicum frutescens*) in Africa is given in Iwu (1993). Details of the active chemical compounds, effects, herbal usage, and pharmacological literature of “Cayenne pepper” are given in Fleming (2000).

As noted by Bertran (1997), in modern medicine, a purified extract of the common chili pepper is used in a cream. Its pain-relieving qualities are based on the active ingredient “capsaicin”, and capsaicin cream is used “as a substitute for the previously-required narcotic analgesics that were used to relieve the excruciating and often intractable pain of a condition that can follow shingles-postherpetic neuralgia. Capsaicin blocks pain signals that come from nerves just under the skin. Pain signals from tissues near the skin are greatly diminished or completely eliminated following continued application of capsaicin. No other compound is known to do this.”

####### Reference.

Nordal (1963).

##### 3. *Datura* L.

###### Datura metel

Taxon classificationPlantaeORDOFAMILIA

L. (= D. fastuosa L.)

####### Names.

**Myanmar**: *padaing*, *pa-daing-byu*, *pa-daing-khata*, *pa-daing-ni*. **English**: devil’s trumpet, Hindu datura, horn of plenty, thorn apple.

####### Range.

Native to the West Indies (Howard 1989), or to tropical Asia (Liogier 1994). Cultivated in Myanmar.

####### Uses.

*Leaf*: Used as a sedative and, when smoked, considered a valuable remedy for asthma. *Seed*: Mixed in curry and sweets, then employed as a narcotic (too high a dose *may kill*, the person may take some days to recover their faculties even at lower doses).

####### Notes.

The medicinal uses of this species in East and Southeast Asian countries are listed in Perry (1980). Medicinal uses of the species in India are discussed in Jain and DeFilipps (1991). Indigenous medicinal uses of the species in the Andaman and Nicobar Islands (India) are described by Dagar and Singh (1999). Medicinal uses of this species in China are discussed by Duke and Ayensu (1985).

The active principle is an alkaloid, hyoscine, found in both seeds and leaves; in too large quantities, it can cause delirium, *coma, and death* (Perry 1980). Chemical constituents, pharmacological action, and medicinal use of this species in Indian Ayurveda are discussed in detail by Kapoor (1990). A pharmacognostical profile including medicinal uses of this plant in Africa is given in Iwu (1993). The toxic properties, symptoms, treatment, and beneficial uses of this plant, parts of which are poisonous, are discussed by Nellis (1997). Worldwide medicinal usage, chemical composition, and toxicity of this species are discussed by Duke (1986).

####### References.

Nordal (1963), Perry (1980).

###### Datura stramonium

Taxon classificationPlantaeORDOFAMILIA

L.

####### Names.

**Myanmar**: *padaing-khat-ta*, *padaing-nyo*. **English**: Jamestown weed, jimson weed, mad apple, moonflower, stinkwort, stramonium, thorn apple.

####### Range.

Native of Mexico; now pantropical. Cultivated in Myanmar.

####### Uses.

*Leaf*: Used as a sedative and antiasthmatic. Liquid from crushed leaves taken with skimmed milk will cure gonorrhea. Crushed leaves mixed with turmeric powder can be used as a poultice to cure breast inflammation or boils in the breasts of women. Sun-dried leaves are incorporated into a smoking cheroot to treat asthma. Roasted and applied to cure inflammation of the joints and aching of bones. *Seed*: Used in the treatment of gonorrhea and dyspepsia. Crushed, ground, and pressed onto the gum to cure toothaches. Seed powder is soaked in sesamum oil for seven days; oil is applied and covered with a thin bandage to cure headaches, aching eyes, backache, leg and foot problems; oil is brushed onto the suprapubic region for menstrual cramps and aches. *Root*: To cure a patient with rabies, a root paste is given orally followed by eating dried roasted beef. *Seed* and *Root*: Used as a tonic to increase virility.

####### Notes.

Medicinal uses of this species in India are discussed in Jain and DeFilipps (1991). Indigenous medicinal uses of this species in the Andaman and Nicobar Islands (India) are described by Dagar and Singh (1999).

*Datura* has been prescribed as a homeopathic remedy for nymphomania; it was utilized by Native American Algonquins of the eastern United States to induce long-term amnesia in coming-of-age ceremonies; and,”atropine, one of the main alkaloids present in *Datura*, is absorbable through the skin, a property that is critical to the herb’s use by witches [in the Middle Ages], who made an ointment or salve with datura as its main ingredient and then applied it to their bodies [often to the sensitive vaginal membranes]” in order to produce sensations of flying and various hallucinations (Mann 1993). This plant contains scopolamine, a compound which is commercially extracted from *Datura inoxia* (see Note under that species) for use in the treatment of motion sickness (e.g., seasickness, airsickness, carsickness) to prevent vomiting and nausea (Davis 1983). The main alkaloid in this species is hyosciamine, the levo-form of atropine; it is a natural anticholinergic with sedative properties (Mors et al. 2000).

The medicinal uses of this plant in the Caribbean region, as well as its chemistry, biological activity, toxicity, and dosages, are discussed by Germosén-Robineau (1997). A pharmacognostical profile including medicinal uses of this plant in Africa is given in Iwu (1993). The toxic properties, symptoms, treatment, and beneficial uses of this plant, parts of which are poisonous, are discussed by Nellis (1997).

Worldwide medicinal usage, chemical constituents, and toxicity of this species are discussed by Duke (1986). A powder or tincture of this plant is used in the treatment of Parkinson’s disease in Europe, and a preparation of the plant in alcohol is used in China and Korea as an anesthetic (Neptune-Rouzier 1997). Details of the active chemical compounds, effects, herbal usage, and pharmacological literature of this plant are given in Fleming (2000). Toxicity of this species is discussed by Bruneton (1999).

####### References.

Nordal (1963), Agricultural Corporation (1980), Forest Department (1999).

##### 4. *Nicandra* Adans.

###### Nicandra physalodes

Taxon classificationPlantaeORDOFAMILIA

(L.) Gaertn.

####### Names.

**English**: apple of Peru, nicandra, shoo-fly plant.

####### Range.

Native to Peru; naturalized elsewhere. Escaped in United States and American tropics, often weedy. In Myanmar, found in Mandalay and Shan.

####### Use.

*Seed*: Used for fumigation of toothache.

####### Notes.

In India the whole plant is used as a diuretic (Jain and DeFilipps 1991). Medicinal uses reported for this species include: Diuretic and mydriatic (research has shown chemicals found in plant effective for this ailment); also *poison*, pediculicide, insecticide, and vermifuge (Duke 2009).

####### Reference.

Nordal (1963).

##### 5. *Physalis* L.

###### Physalis peruviana

Taxon classificationPlantaeORDOFAMILIA

L.

####### Names.

**Myanmar**: *hpaung-hpaung-thi*, *kala-myetsi-pinzauk-gyi*. **English**: cape gooseberry, cherry tomato, goldenberry, ground cherry, Peruvian winter cherry.

####### Range.

Northern and western tropical South America. Cultivated in Myanmar.

####### Use.

*Whole plant*: Used as a diuretic.

####### Note.

In India the leaf of this plant is used for abdominal troubles (Jain and DeFilipps 1991).

####### Reference.

Nordal (1963).

##### 6. *Solanum* L.

###### Solanum anguivi

Taxon classificationPlantaeORDOFAMILIA

Lam. (= S. indicum L.)

####### Names.

**Myanmar**: *khayan-kazaw-kha*, *kasawt-kha*, *haw hkan kaju* (Kachin). **English**: Indian nightshade.

####### Range.

Pantropical, subtropical.

####### Uses.

Preparations made from parts of this plant are used to dissolve phlegm, stimulate the appetite, and strengthen the heart, as well as to treat leprous sores, fever, asthma, gas, and rashes. *Whole plant*: The juice and the crushed parts are used to make a poultice to neutralize venom of snake and centipede bites; also for excessive white vaginal discharge. Additionally, the plant is chopped and boiled in water until the water is reduced to half the starting volume; after the cooked liquid is strained through a clean cloth and cooled, honey is added (about 5 ounces), and one-half cup of the mixture is drunk twice. *Fruit*: Smoke from burning fruit is directed into the ear to cause insects to emerge. *Root*: Used as a carminative and spasmolytic. Also used for toothaches, either in the form of a paste pressed into the tooth or as inhaled smoke from ground root powder. To stop nose bleeds a paste, made by grinding the root with rice washing water, is used. The root powder and boiled betel (*Piper betle*) leaf water is ingested as a major defense against cooking fumes.

####### Notes.

The medicinal uses of this species in India are discussed in Jain and DeFilipps (1991). Medicinal uses of this species in China are discussed in Duke and Ayensu (1985).

####### References.

Nordal (1963), Agricultural Corporation (1980), Forest Department (1999).

###### Solanum melongena

Taxon classificationPlantaeORDOFAMILIA

L.

####### Names.

**Myanmar**: *kayan*, *sin-kayan*. **English**: eggplant.

####### Range.

Africa and Asia. Widely cultivated in many countries.

####### Uses.

*Leaf*: Employed as a narcotic and as a stimulant.

####### Notes.

Medicinal uses of this species in India are discussed in Jain and DeFilipps (1991). Indigenous medicinal uses of this species in the Andaman and Nicobar Islands (India) are described by Dagar and Singh (1999). Medicinal uses of this species in China are discussed by Duke and Ayensu (1985).

####### Reference.

Nordal (1963).

###### Solanum rudepannum

Taxon classificationPlantaeORDOFAMILIA

Dunal (= S. torvum Sw.)

####### Names.

**Myanmar**: *kazaw-kha*, *kayan-kazaw*. **English**: wild eggplant.

####### Range.

New World tropics.

####### Use.

*Fruit*: Used to treat diabetes.

####### Notes.

Medicinal uses of this species in India are discussed in Jain and DeFilipps (1991).

The chemistry, pharmacology, toxicology, and use of this species as a hunting poison and medicinal plant in Africa are discussed by Neuwinger (1994).

####### Reference.

Nordal (1963).

#### Symplocaceae (Sweetleaf family)

##### 1. *Symplocos* Jacq.

###### Symplocos racemosa

Taxon classificationPlantaeORDOFAMILIA

Roxb.

####### Names.

***Myanmar***: *dauk-yut, mwet-kang, nle-prangkau, pya*. **English**: sweetleaf.

####### Range.

China, India, Myanmar, Thailand, and Vietnam. Widely distributed in Myanmar.

####### Use.

*Fruit* and *Bark*: Used to treat opthalmia.

####### Notes.

In India the bark is used for bronchitis, digestive and urinary disorders, menorrhagia, eye diseases, ulcers, bleeding gums, maturation of wounds, liver problems, elephantiasis, and fat in the urine (Jain and DeFilipps 1991).

The bark contains starch, calcium oxalate, alumina, alkaloid, tannin; but no saponin, oil, or fat (Perry 1980).

####### Reference.

Perry (1980).

#### Theaceae (Tea family)

##### 1. *Schima* Reinw. ex Blume

###### Schima wallichii

Taxon classificationPlantaeORDOFAMILIA

Choisy

####### Names.

**Myanmar**: *laukya*, *laukya-byu*, *mai-song*, *masa*, *meiksong*, *pan-ma*, *thitya-byu*, *thityah*, *thitya-ni*. **English**: Chinese guger tree, needle wood, schima.

####### Range.

India, east to Indonesia and Taiwan. In Myanmar, found in Bago, Chin, Kachin, Kayin, Mandalay, Rakhine, Sagaing, Shan, and Taninthayi.

####### Use.

*Bark*: Anthelmintic.

####### Note.

In India the bark is an anthelmintic and rubefacient, irritating to the skin (Jain and DeFilipps 1991).

####### Reference.

Nordal (1963).

#### Thymelaeaceae (Bitter Mahoe family)

##### 1. *Aquilaria* Lam.

###### Aquilaria malaccensis

Taxon classificationPlantaeORDOFAMILIA

Lam. (= A. agallocha Roxb.)

####### Names.

**Myanmar**: *akyaw*, *klaw* (Kayin), *thit-hmwe*. **English**: agarwood, aloewood, eaglewood.

####### Range.

Southeast Asia: Bangladesh, Bhutan, India, Indonesia, Iran, Laos, Malaysia, Myanmar, the Philippines, Singapore, and Thailand. In Myanmar, grows naturally along the Tanintharyi Yomas, and on islands in Beik district; found in Chin, Kachin, Mandalay, Mon, and Sagaing.

####### Conservation status.

Vulnerable [VU A1cd] (IUCN 2017).

####### Uses.

Preparations made from parts of this tree are used to control coughs and leprosy, stimulate weight gain, alleviate indigestion, treat eye and ear ailments, promote urinary flow, resolve liver and intestinal problems, and eliminate bad breath. *Sap*: Applied topically to make the body feel light and agile. *Wood*: Grated and used in various preparations, both external and internal, especially for illness during and after childbirth, but also to treat rheumatism, smallpox, abdominal illnesses, and other body pains; additionally, used as a cosmetic. The scented wood is employed as a stimulant, tonic, and carminative. It is also a constituent of medicine for heart palpitation, and other illnesses.

Inner wood is made into a paste which is inhaled, or burned to produce fumes for inhaling as a remedy for excessive dizziness; applied topically or ingested to cure vomiting, stop bleeding, and alleviate swollen joints; and applied at frequent intervals as a remedy for skin disorders and conditions arising from lack of hygiene. The paste, mixed with the root bark from *kyet-hsu* (*Ricinus communis*), is applied topically to alleviate stomachaches; ingested to treat asthma and vomiting; made from the wood of the black akyaw variety, is mixed with oil and applied topically to cure shooting stomach pains. The wood powder- mixed with honey, and ingested by licking, is considered a cure for heart disease and long-lasting fevers; rolled in *thanat-pet* (*Cordia dichotama*) leaves and smoked like a cigarette or in a pipe, is used to strengthen the heart and stomach. To stimulate proper healing, a mixture of the wood and sap from *Oh-htane-pin* (the scientific name of this plant could not be ascertained per Thi Thi Ta, *personal communication*) is placed on embers to produce smoke directed toward sores that have not healed, infected sores, and sores infested with maggots.

####### Notes.

In India the wood is an aphrodisiac, carminative, stimulant, and tonic; also used for snakebite, and as an astringent for treating vomiting and diarrhea (Jain and DeFilipps 1991). In China the leaf is used for malaria; the stem bark is used as an astringent and antidysenteric; and the root is also astringent (Duke and Ayensu 1985).

In East and Southeast Asia medicinal uses of this species are given as follows: In Mongolia “Bezoar” from the bark is employed to “remove the poison” of feverish illnesses; in China it is used as an aphrodisiac, a diuretic, and for the purposes mentioned in the previous paragraph; in Indo-China the heartwood is thought to be antifebrile and antimalarial, also a decoction of it is given for paralysis, and alcohol from macerating it is used as a remedy for vomiting, cholera, cough, anuria, and indigestion; on the Malay Peninsula an infusion from the grated root is given to treat general dropsy or anasarca, finely ground leaves are rubbed over swollen hands and legs of a someone with dropsy, and resin from the wood is an ingredient in sedatives; and in Indonesia the leaves, mixed with vinegar, salt, and charcoal, are used to treat vomiting (Perry 1980).

From the grated wood of *A. agallocha* (i.e., *A. malaccensis*) comes a drug with great antiquity, referred to in the Scriptures and all works dealing with Eastern Materia Medica. This drug has several current uses, both external and internal. It is used in various preparations for illness during and after childbirth; to treat rheumatism, smallpox, abdominal ills, and other body pains. The the scented wood is also said to have the properties of a stimulant, tonic, and carminative; as well as being a constituent of medicines for the heart palpitation (Perry 1980).

####### References.

Agricultural Corporation (1980), Perry (1980).

##### 2. *Linostoma* Wall. ex Endl.

###### Linostoma pauciflorum

Taxon classificationPlantaeORDOFAMILIA

Griff.

####### Range.

Asia. In Myanmar, found in Mon and Taninthayi.

####### Use.

Plant said to be used medicinally, but specific use not given.

####### Notes.

Another member of this genus, *Linostoma decandrum*, is used as a piscicide (Duke 2009). The genus, although used medicinally, is “chiefly *poisonous*” (Perry 1980).

####### Reference.

Perry (1980).

#### Ulmaceae (Elm family)

##### 1. *Holoptelea* Planch.

###### Holoptelea integrifolia

Taxon classificationPlantaeORDOFAMILIA

Planch.

####### Names.

**Myanmar**: *myauk-seik*, *pyauk-seik*. **English**: Indian elm.

####### Range.

India, Nepal, Sri Lanka; Cambodia, Laos, Myanmar, and Vietnam. Widely distributed in Myanmar.

####### Use.

*Bark*: Used to treat rheumatism.

####### Notes.

The bark and leaves are bitter, astringent, acrid, thermogenic, anti-inflammatory, digestive, carminative, laxative, anthelmintic, depurative, and revulsive; considered useful in vitiated conditions of kapha and pitta, inflammations, dyspepsia, flatulence, colic, helminthiasis, vomiting, skin diseases, leprosy, diabetes, hemorrhoids, and rheumatism (Warrier et al. 1994).

An aqueous extract of leaves of this species has shown antimicrobial activity (Sharma et al. 2009).

####### Reference.

Perry (1980).

#### Urticaceae (Nettle family)

##### 1. *Boehmeria* Jacq.

###### Boehmeria nivea

Taxon classificationPlantaeORDOFAMILIA

(L.) Gaudich.

####### Names.

**Myanmar**: *ban*, *gon*, *kya-sha*, *lashen*. **English**: China grass, Chinese silk plant, ramie.

####### Range.

Tropical Asia, where cultivated for fiber. Cultivated in Myanmar.

####### Use.

*Root*: Used as laxative.

####### Notes.

In India the leaf is used as a resolvent and the root as an aperient (Jain and DeFilipps 1991). In China the plant is used as a hemostat; the leaf is astringent, used for fluxes and wounds; the root is used as an antiabortifacient, for cooling, a demulcent, diuretic, resolvent, uterosedative, for insect and snakebite, and poisoned arrow wounds. A decoction of the leaf is astringent, antihemorrhagic, diuretic, styptic, and also used for rectal prolapse, leucorrhea, urogenital inflammation, insect and snakebite, puerperal fever, erysipelas, poisoned arrow, and rheumatism (Duke and Ayensu 1985).

####### Reference.

Nordal (1963).

##### 2. *Girardinia* Gaudich.

###### Girardinia diversifolia

Taxon classificationPlantaeORDOFAMILIA

(Link) Friis (= G. heterophylla (Vahl) Decne.)

####### Names.

**Myanmar**: *gwi-lakajawng*, *petya*, *petya-gyi*, *sin-petya*. **English**: Himalayan nettle.

####### Range.

China, Bhutan, India, Indonesia, Korea, Malaysia, Nepal, Sikkim, Sri Lanka; Africa, including Madagascar. Reported from Myanmar.

####### Uses.

*Leaf*: Used for headache and swollen joints, also used for fever.

####### Notes.

In India the leaf of this species is used for swollen joints and headache; also as a decoction for fever (Jain and DeFilipps 1991). In China a decoction of the root and basal part of the stem of this species, mixed with wine, is drunk to “cure malignant boils”; a broth made from cooking it with pork is used as a remedy for stomachache (Perry 1980). Medicinal uses of this species in China are also discussed in Duke and Ayensu (1985).

####### Reference.

Nordal (1963).

##### 3. *Urtica* L.

###### Urtica dioica

Taxon classificationPlantaeORDOFAMILIA

L.

####### Names.

**English**: ortie, stinging nettle.

####### Range.

China, Afghanistan, Central Himalayas; northern Africa, Europe, and North America. Widespread in temperate regions of both hemispheres.

####### Conservation status.

Least Concern [LC] (IUCN 2017).

####### Use.

*Root*: Used as a diuretic.

####### Notes.

In India the whole plant is used as an anthelmintic, a local irritant in paralysis, for nephritis, menorrhagia, jaundice, and a decoction is astringent: the leaf is used for wounds and boils, also locally for sprains and rheumatism; the leaf and root are used in an infusion for dandruff; the seed and root are used for diarrhea; and an unspecified plant part is used as a hemostatic for uterine hemorrhage and bleeding from the nose (Jain and DeFilipps 1991).

####### Reference.

Nordal (1963).

###### Urtica parviflora

Taxon classificationPlantaeORDOFAMILIA

Roxb.

####### Names.

**English**: Mousa nettle.

####### Range.

Eastern Asia - Himalayas (Bhutan, North India, Kashmir, Nepal, Sikkim).

####### Use.

*Root*: Oil from the root used as a stomachic.

####### Notes.

In India the whole plant is used in a decoction for fevers (Jain and DeFilipps 1991). The species is said to be used as a tonic and suppository; for fever, gout, parturition, and rheumatism; and as a counterirritant, for dislocation, fracture, sprain, and swelling (Duke 2009).

####### Reference.

Nordal (1963).

#### Verbenaceae (Vervain family)

##### 1. *Lantana* L.

###### Lantana × aculeata

Taxon classificationPlantaeORDOFAMILIA

L.

####### Names.

**Myanmar**: *seinnaban*, *nadaung-ban*. **English**: lantana, wild sage.

####### Range.

Native to Tropical America; introduced in the East, and now pantropic. Reported from Myanmar.

####### Uses.

*Whole plant*: Used as tonic, antispasmodic, and diaphoretic.

####### Notes.

Medicinal uses of this species in India are discussed in Jain and DeFilipps (1991). Indigenous medicinal uses of this species in the Andaman and Nicobar Islands (India) are described by Dagar and Singh (1999). Medicinal uses of this species in China are discussed by Duke and Ayensu (1985). Medicinal uses of this species in South China, Indo-China, the Malay Peninsula, Indonesia, and the Philippines are discussed in Perry (1980).

The medicinal uses of this plant in the Caribbean region, as well as its chemistry, biological activity, toxicity and dosages, are discussed by Germosén-Robineau (1997). The chemistry, pharmacology, history, and medicinal uses of this species in Latin America are discussed in detail by Gupta (1995). The chemical constituents, pharmacological activities, and traditional medicinal uses of this plant on a worldwide basis are discussed in detail by Ross (1999). The toxic properties, symptoms, treatment, and beneficial uses of this plant, parts of which are poisonous, are discussed by Nellis (1997).

Worldwide medicinal usage, chemical composition and toxicity of this species are noted by Duke (1986). Toxicity of this species is discussed by Bruneton (1999).

####### Reference.

Nordal (1963).

##### 2. *Stachytarpheta* Vahl

###### Stachytarpheta indica

Taxon classificationPlantaeORDOFAMILIA

(L.) Vahl (= S. jamaicensis var. indica (L.) H.J. Lam)

####### Names.

**Myanmar**: *aseik-taya*, *ye-chaung-pan*. **English**: Brazilian tea.

####### Range.

New World tropics. Widely dispersed in Myanmar.

####### Use.

*Leaf*: Used to treat ulcers.

####### Notes.

The medicinal uses of this species in India are discussed in Jain and DeFilipps (1991). Indigenous medicinal uses of this species in the Andaman and Nicobar Islands (India) are described by Dagar and Singh (1999).

The chemistry, pharmacology, history, and medicinal uses of this species in Latin America are discussed in detail by Gupta (1995).

####### Reference.

Nordal (1963).

##### 3. *Verbena* L.

###### Verbena officinalis

Taxon classificationPlantaeORDOFAMILIA

L.

####### Names.

**Myanmar**: *saung-daw-ku*. **English**: bluebird vine.

####### Range.

Widespread in temperate and subtropical regions. Cultivated in Myanmar.

####### Uses.

The plant is bitter, cooling, useful for congestion, and as an antidote for insect bites. *Leaf*: Rubefacient used for rheumatism.

####### Notes.

The medicinal uses of this species in India are discussed in Jain and DeFilipps (1991). Medicinal uses of this species in China are discussed in Duke and Ayensu (1985). Perry (1980) discusses the medicinal uses of the species in Korea, China, Taiwan, and Indo-China. In most of these countries the plant (above ground part) is collected in full flower. It has the properties of an emmenagogue, purgative, anthelmintic, antiscorbutic, antihemorrhagic, and a diaphoretic. It is used internally to treat colds, fever, various types of inflammation, digestive and intestinal trouble, disorders of the urinary tract, and uterine disorders; it also helps to quicken separation of the placenta and acts as a depurative after parturintion. It is used as a remedy for dropsy, tympanites, and anemia (when taken with molasses). Externally it serves either as a poultice or a wash for skin diseases, abscesses, and tumors, as well as severe wounds (pounded plant acts as a styptic); also an insecticide.

####### Reference.

Nordal (1963).

#### Vitaceae (Grape family)

##### 1. *Leea* D.Royen

###### Leea macrophylla

Taxon classificationPlantaeORDOFAMILIA

Roxb. ex Hornem.

####### Names.

**Myanmar**: *kya-hpetgyi*, *mai-sung-hkong-long*, *mak-tasu-long*. **English**: leea.

####### Range.

Tropical Asia and Africa. Widely distributed in Myanmar; also cultivated.

####### Uses.

*Root*: Astringent, and this property used in medication for guineaworm.

####### Notes.

This species is cultivated in Myanmar especially for its astringent property (Perry 1980). Recorded medicinal uses for the species include anodyne, astringent, cicatrizant, larvicide, vermicide, and verimifuge; also for guineaworm, ringworm, dysentery, neuralgia, and splenitis (Duke 2009).

####### Reference.

Perry (1980).

#### Zingiberaceae (Ginger family)

##### 1. *Alpinia* Roxb.

###### Alpinia galanga

Taxon classificationPlantaeORDOFAMILIA

(L.) Willd.

####### Names.

**Myanmar**: *padei-kaw gyi*, *kunsa-gamon*, *kawain-hnoot* (Mon). **English**: greater galangal.

####### Range.

India, Indonesia, Malaysia, Myanmar, Thailand, Vietnam. Reported from Myanmar.

####### Uses.

*Stem*: The hot, spicy, bitter rhizome is known for its heating properties, for blood and phlegm regulation, controlling cases of poisoning and inflammation, facilitating digestion, keeping the heart healthy, and stimulating the appetite. It is a major component in medications for dysentery, asthma, and heart disease. For difficulty in urination, the paste of the rhizome, made with or without water from washing rice, is taken orally as a diuretic for cases of inability to urinate even though the bladder is full, and for pain and discomfort in urination. With ginger juice and honey, the rhizome is taken as a cure for coughs. Powdered and mixed with *samone-byu* (*Anisochilus carnosus*) and roasted salt, it is taken for chest pains and stomach pains; mixed with equal amounts of dried ginger rock salt, the powdered rhizome it is a remedy for indigestion. Fevers are treated with the liquid from boiling the rhizome and an effective ngan-hsay (traditional medicine used for high fever).

####### Notes.

Medicinal uses of this species in India are discussed in Jain and DeFilipps (1991). Medicinal use of this species in China is discussed by Duke and Ayensu (1985).

####### References.

Agricultural Corporation (1980), Forest Department (1999).

###### Alpinia officinarum

Taxon classificationPlantaeORDOFAMILIA

Hance

####### Names.

**Myanmar**: *padegaw-gale*, *padei-kaw lay*, *kawaintoot* (Mon). **English**: lesser galangal.

####### Range.

Asia. In Myanmar, found in Bago and Yangon.

####### Uses.

The lesser galangal (*Alpinia officinarum*) does not have such strong and effective taste and smell as the greater galangal (*A. galanga*). *Stem*: Mature rhizomes sharp and bitter in taste with heating properties; used to whet the palate and regulate the bowels. The boiled rhizome is ingested to treat excessive urination. Oil from cooking the rhizome can be applied for heaviness of limbs and stiffness in the neck and back. To help prompt or improve speech, a small amount of rhizome paste is given to children for swallowing or rubbed on their tongues.

####### Notes.

Medicinal uses of this species in India are discussed in Jain and DeFilipps (1991). Medicinal use of this species in China is discussed by Duke and Ayensu (1985).

####### Reference.

Agricultural Corporation (1980).

##### 2. *Curcuma* L.

###### Curcuma comosa

Taxon classificationPlantaeORDOFAMILIA

Roxb.

####### Names.

**Myanmar**: *nanwinga*, *sanwinga*, *sanwin-yaing*. **English**: tumeric.

####### Range.

Tropical Asia. In Myanmar, found in Bago and Mandalay.

####### Use.

*Stem*: One tablespoon of the powdered dried rhizome mixed with honey is taken twice daily to lower blood pressure.

####### Notes.

The rhizomes are used externally in indigenous medicine in Thailand, and as an anti-inflammatory agent. Also, in combination with *Artemizia annua* and *Artistolochia tagala*, they are used to reduce malaria fever and as an aromatic stomachic (Khine 2006).

Five diphenylheptenoids have been extracted and tested for their inhibition of nematode activity. On the nematode *Caenorhabditis elegans*, it has been shown to be a most potent inhibitor of nematode motility. A phloracetophenone glucoside has also been isolated (Khine 2006).

####### References.

Mya Bwin and Sein Gwan 1973, Forest Department (1999).

###### Curcuma longa

Taxon classificationPlantaeORDOFAMILIA

L.

####### Names.

**Myanmar**: *nanwin*, *hsanwin*, *sa-nwin*, *namchying* (Kachin), *aihre* (Chin), *meet* (Mon). **English**: turmeric.

####### Range.

India. Widely cultivated in the tropics. Cultivated in Myanmar.

####### Uses.

*Stem (Rhizome)*: Hot, sharp, bitter, and savory, use of the rhizome known for reversing many ailments and increasing overall longevity. It is used in making different medicines, ointments, and smoke treatments (herbs scattered over glowing embers of charcoal and patient sits nearby with large basket over which blanket placed) for a variety of conditions, including digestive problems, very high fevers, eye problems, male-related troubles, coughs, asthma and bronchitis, and diarrhea. Powdered turmeric is mixed with water and ingested, burned to create fumes for inhaling, boiled in water for bathing, or tied in a cloth bundle applied to different areas of the body needing treatment. Turmeric reduces fevers, lowers post-partum high blood pressure, expels “bad blood” left in the body after childbirth, and purifies the blood. It relieves post-partum weakness, cold skin, breast aches or inflammation, bloating and edema associated with female disorders, itches, and rashes; and is also used to treat an unclean or infected uterus, aching of the eyes, colds and fevers. Mixed with powder from the bark of *let-htoke* (*Holarrhena antidysenterica*) and a moderate amount of honey, turmeric is stewed with water and taken as a remedy for dysentery and for vomiting or otherwise passing blood. Mixed with warm water and held in the mouth, it is used to treat inflamed gums and toothaches; alternatively it is mixed with salt and pressed into the root of the affected tooth. Taken with a small amount of salt three times daily, turmeric eases bloating and pain from flatulence. Three thin slices of the sun-dried rhizome daily alleviates gastritis. Mixed with lime, turmeric relieves cysts, knots in muscles, and bruises, and turmeric powder is applied to wounds to stop excessive bleeding. Ingesting a mixture of turmeric, brown rock sugar, and water from washing rice treats bladder stones; a mixture of turmeric, juice from *zee-hpyu* (*Phyllanthus emblica*) and honey relieves urinary infections.

####### Notes.

The medicinal uses of this species in India are discussed in Jain and DeFilipps (1991). Medicinal use of this species in China is discussed by Duke and Ayensu (1985). The various medicinal uses of this species are also discussed in Perry (1980). She notes that the main tubers, over a year old, are used in medicine while the lateral rhizomes are used in cooking.

####### Reference.

Agricultural Corporation (1980).

###### Curcuma zedoaria

Taxon classificationPlantaeORDOFAMILIA

(Christm.) Roscoe

####### Names.

**Myanmar**: *sa-nwin*. **English**: long zedoary, zedoary, round zedoary.

####### Range.

From the Himalayas to Chittagong south into Indonesia, especially northeastern India; cultivated elsewhere. Cultivated in Myanmar.

####### Uses.

*Stem (Rhizome)*: Used as tonic for the heart; also used as mouthwash.

####### Notes.

“The rhizome is official in many pharmacopeias. Everywhere it is regarded as a stomachic and carminative.” In China it is used as a tonic nutrient and a resolvent of swellings and contusions; it is also used to dissolve blood clots, promote circulation, and to reduce abdominal pain. In Taiwan it is used to treat heart complaints, cholera, gonorrhea, irregular menstruation, and snakebites. In Indo-China it is used as a tonic. In the Philippines, ash from the rhizome is applied to wounds and ulcers (Perry 1980).

The medicinal use of this species in India is discussed in Jain and DeFilipps (1991). Here the rhizome is crushed and mixed with water for making a bath to treat jaundice. Medicinal uses of this species in China are discussed in Duke and Ayensu (1985).

Reported constituents are volatile oil, cineole, camphene, zingiberene, borneol, camphor, curcumin, zedoarin, gum, resin, and starch (Perry 1980).

####### Reference.

Nordal (1963).

##### 3. *Elettaria* Maton

###### Elettaria cardamomum

Taxon classificationPlantaeORDOFAMILIA

(L.) Maton

####### Names.

**Myanmar**: *hparlar hpyu*. **English**: lesser cardamon.

####### Range.

Native to southern India; cultivated widely in the tropics. Cultivated in Myanmar.

####### Use.

*Seed*: Used to cure headaches. Eat roasted seeds with medicines to cure urinary disorders. Together with the roots of *peik-chin* (*Piper longum*) can be made into a powder, mixed with butter to cure heart disease. Used to make into medicines to treat irregular menstruation and menopause symptoms. Used to make into smallpox medicines. Crushed and mixed with honey to treat coughing, asthma, and sore throat.

####### Reference.

Agricultural Corporation (1980).

##### 4. *Kaempferia* L.

###### Kaempferia elegans

Taxon classificationPlantaeORDOFAMILIA

(Wall.) Baker

####### Names.

**Myanmar**: *kun-kado*. **English**: green ripple peacock ginger, purple-flowered resurection lily, resurection lily.

####### Range.

China (Sichuan), India, Malaysia, the Philippines, Thailand. In Myanmar, found in Bago, Mandalay, Mon, Taninthayi, and Yangon.

####### Use.

*Stem (Rhizome)*: Used to treat dysentery.

####### Notes.

Many *Kaempferia* species are utilized as medicinal plants throughout Southeast Asia. The rhizome of *Kaempferia* is ground into a paste and applied externally for the treatment of sprains (Burkill 1966).

####### Reference.

Nordal (1963).

##### 5. *Zingiber* Boehm.

###### Zingiber montanum

Taxon classificationPlantaeORDOFAMILIA

(J.Koenig) Link ex A.Dietr. (= Z. cassumunar Roxb.)

####### Names.

**Myanmar**: *meik-tha-lin*, *hta-nah* (Mon). **English**: wild ginger.

####### Range.

Tropical Asia. Widely distributed in Myanmar.

####### Uses.

Hot in taste, the species is used to regulate the blood, stimulate urination, and release gas. *Whole plant*: Its five parts are used in making up medicines to cure coughs, asthma, leprosy and other skin disorders, and in deworming; mixed with a little salt, the juice is used to stimulate menstruation; applying the juice mixed with a small amount of pepper used to prevent catching a cold and to treat the aches, pains, heaviness, and dullness of poor circulation; brewing in a moderate amount of water and ingesting the liquid is used as a remedy for diarrhea and diarrhea with shooting or dull pains. Taking about two tablespoons of the liquid from boiling the five parts in water, together with coriander seeds, and reducing the volume to half, alleviates severe diarrhea; crushing them, followed by boiling, yields a distillate that relieves internal hemorrhoids if taken regularly, two tablespoons at a time. For snakebites, the juice of the five parts is ingested and also externally applied to the wound. *Rhizome*: Crushed and tied on with a bandage, used as a poultice for wounds, aches, knotted muscles, and, in the elderly, inflamed joints, swollen knees, and swollen ankles.

####### Notes.

The medicinal uses of this species in India are discussed in Jain and DeFilipps (1991). Perry (1980) discusses the uses of this species in general, and in East and Southeast Asian countries.

####### Reference.

Agricultural Corporation (1980).

###### Zingiber officinale

Taxon classificationPlantaeORDOFAMILIA

Roscoe

####### Names.

**Myanmar**: *gyin*, *lacow-sacopf*, *lagoe-htaneg* (Mon). **English**: Canton ginger, common ginger, true ginger.

####### Range.

Tropical southeastern Asia. Also, cultivated in the tropics and in Myanmar.

####### Uses.

*Stem (Rhizome)*: Both sweet and bitter, the rhizome’s cooling properties stimulate appetite and regulate bowels, phlegm, and gall bladder function. Used as a diuretic and a poison antidote, the rhizome is also considered a remedy for laryngitis, chest and respiratory ailments, infected sores, and inflammation caused by injury. Rhizome juice- mixed with honey, used to treat colds, runny noses, coughs, asthma, and bronchitis; mixed with onion juice, taken for nausea and for hiccups; mixed in equal parts with juice from *pin-sein* (*Ocimum americanum*, lemon basil or *O. basilicum*) leaves and sweetened with honey, used to treat cholera. Warmed, pure rhizome juice is used as ear drops for earaches; also can be cooked together with sesame oil and used as a rub applied to inflamed joints to ease inflammation and pain. Chewed and kept in place at the affected areas, the rhizome alleviates toothaches. Boiled together with jaggery and betel (*Piper betle*) leaves, the rhizome liquid is taken as a cure for influenza, digestive aid, and blood purifier for new mothers.

####### Notes.

Medicinal uses of this species in India are discussed in Jain and DeFilipps (1991). Medicinal use of this species in China is discussed by Duke and Ayensu (1985). Perry (1980) also discussed the medicinal uses of the species.

####### Reference.

Agricultural Corporation (1980).

###### Zingiber zerumbet

Taxon classificationPlantaeORDOFAMILIA

(L.) Roscoe ex Sm.

####### Names.

**Myanmar**: *zinbyu-bin*, *linne-gyi*. **English**: ginger.

####### Range.

India. Cultivated in Myanmar.

####### Use.

*Stem*: The rhizome is used as a carminative.

####### Notes.

Medicinal uses of this species in India are discussed in Jain and DeFilipps (1991) as follows: The rhizome is used for cough, stomachache, asthma; as a vermifuge; and on leprosy and other skin diseases. The plant (part unspecified) is also used for mucus in the urine, bronchitis, and for asthma (in combination with several other species).

####### Reference.

Nordal (1963).

## Appendix 1

**Common names index**

achiote

*Bixa orellana*

adalut

*Canna indica*

Adam’s apple

*Tabernaemontana divaricate*

adlay

*Coix lacryma-jobi*

adlay millet

*Coix lacryma-jobi*

adulsa

*Justicia adhatoda*

aeginetia

*Aeginetia indica*

aerial yam

*Dioscorea bulbifera*

aerva

*Aerva javanica*

African marigold

*Tagetes erecta*

agarwood

*Aquilaria malaccensis*

agrimony

*Agrimonia eupatoria*

*ah-lu-thi*

*Dioscorea bulbifera*

*aihre*

*Curcuma longa*

air plant

*Bryophyllum pinnatum*

air potato

*Dioscorea bulbifera*

*aka-wadi*

*Passiflora quadrangularis*

*a-kyaw ta-htaung*

*Plantago major*

*akyaw*

*Aquilaria malaccensis*

alder

*Alnus nepalensis*

Alexandrian laurel

*Calophyllum inophyllum*

Alexandrian senna

*Senna alexandrina*

aloe

*Aloe vera*

aloewood

*Aquilaria malaccensis*

*alo-kyu*

*Arundo donax*

American arrowroot

*Maranta arundinacea*

American upland cotton

*Gossypium hirsutum*

amoora

*Aglaia cucullata*

angel’s trumpet

*Brugmansia suaveolens*

annatto

*Bixa orellana*

Annie’s lace

*Cyperus scariosus*

*antamray*

*Sinapis alba*

*anya-kokk*

*Albizia lebbeck*

apple of Peru

*Nicandra physalodes*

apricot blush foxglove

*Digitalis purpurea*

*ar ganui*

*Mesua ferrea*

Arabian coffee

*Coffea arabica*

Arabian senna

*Senna alexandrina*

Arabica coffee

*Coffea arabica*

aramina

*Urena lobata*

*ar-do*

*Canna indica*

arrowroot

*Maranta arundinacea*

*arthaw-ka*

*Polyalthia longifolia*

asana

*Bridelia retusa*

*aseik*

*Antiaris toxicaria*

*aseik-pye*

*Leucaena leucocephala*

*aseik-taya*

*Stachytarpheta indica*

ash pumpkin

*Benincasa hispida*

Asian markhamia

*Markhamia stipulata*

asoka tree

*Saraca indica*

asparagus

*Asparagus officinalis*

Assyrian plum

*Cordia myxa*

*aug-mai-hpyu*

*Clitoria ternatea*

*aukkyu*

*Bischofia javanica*

*aukkywe*

*Bischofia javanica*

*aung-me-nyo*

*Clitoria ternatea*

Australian asthma weed

*Euphorbia hirta*

Australian fever tree

*Eucalyptus globulus*

Australian pine

*Casuarina equisetifolia*

Australian red cedar

*Toona sureni*

avaram

*Senna auriculata*

*aweya*

*Leucaena leucocephala*

*awsa*

*Annona squamosa*

*awzar*

*Annona squamosa*

*azat*

*Annona squamosa*

Aztec marigold

*Tagetes erecta*

*babchi*

*Cullen corylifolium*

*babu*

*Acacia nilotica*

*babul*

*Acacia nilotica*

*badan*

*Terminalia catappa*

bael tree

*Aegle marmelos*

*bahon*

*Flemingia chappar*

*Phyllodium pulchellum*

*baing-daung*

*Rhizophora mucronata*

ball tree

*Aegle marmelos*

balloon vine

*Cardiospermum halicacabum*

balsam-apple

*Momordica charantia*

balsam-pear

*Momordica charantia*

*baluma-shaw*

*Kydia calycina*

*balu-wah*

*Abelmoschus moschatus*

*bamachet*

*Cratoxylum formosum*

*bambwe*

*Careya arborea*

*ban*

*Boehmeria nivea*

*banda*

*Terminalia catappa*

Barbados flower

*Caesalpinia pulcherrima*

Barbados nut

*Jatropha curcas*

*bar-kyaw pin*

*Plantago major*

barleria

*Barleria prionitis*

bastard indigo

*Tephrosia purpurea*

bastard jute

*Hibiscus cannabinus*

bastard sandalwood

*Mansonia gagei*

bastard-teak

*Butea monosperma*

*baw-sagaing*

*Leucaena leucocephala*

*bawzagaing*

*Leucaena leucocephala*

beach morning glory

*Ipomoea pes-caprae*

bead tree

*Adenanthera pavonina*

beautyberry

*Callicarpa macrophylla*

*bebya*

*Rotheca serrata*

*beda*

*Monochoria vaginalis*

beddome

*Terminalia tomentosa*

*beeda khutdai*

*Senna alata*

beefwood

*Casuarina equisetifolia*

begar’s-tick

*Tadehagi triquetrum*

*begyo*

*Rotheca serrata*

*bein-hpo*

*Jatropha multifida*

*bein-nwe*

*Hiptage benghalensis*

*bein-sa*

*Mitragyna speciosa*

bela tree

*Aegle marmelos*

bell bush

*Brugmansia suaveolens*

bell pepper

*Capsicum annuum*

belleric

*Terminalia bellirica*

bellyache bush

*Jatropha gossypiifolia*

Ben nut

*Moringa oleifera*

Bengal arum

*Typhonium trilobatum*

Bengal quince

*Aegle marmelos*

*beng-kong*

*Tamarindus indica*

Benjamin tree

*Ficus benjamina*

*ber-hrum*

*Glycine max*

betel

*Piper betle*

betel pepper

*Piper betle*

betel vine

*Piper betle*

*bezat*

*Chromolaena odorata*

*bhang*

*Cannabis sativa*

bicolor Persian violet

*Exacum tetragonum*

*bilat-chinbaung*

*Hibiscus sabdariffa*

bimli jute

*Hibiscus cannabinus*

bimlipatum jute

*Hibiscus cannabinus*

bird’s nest

*Daucus carota*

bishop’s weed

*Trachyspermum ammi*

bishop’s wood

*Bischofia javanica*

*bi-thawar*

*Linum usitatissimum*

bitter aloe

*Aloe vera*

bitter cucumber

*Momordica charantia*

bitter gourd

*Momordica charantia*

bitter melon

*Momordica charantia*

bitter orange

Citrus × aurantium

bitter stick

*Swertia chirayita*

bitterwood

*Quassia indica*

bittu bark

*Eurycoma longifolia*

*bizat*

*Chromolaena odorata*

black chuglam

*Terminalia citrina*

black creeper

*Ichnocarpus frutescens*

black cumin

*Nigella sativa*

black cutch

*Acacia catechu*

black oil plant

*Celastrus paniculatus*

black pepper

*Piper nigrum*

black plum

*Premna mollissima*

*Syzygium cumini*

blackberry tree

*Vitex glabrata*

black-eyed Susan vine

*Thunbergia erecta*

bleeding-heart vine

*Clerodendrum thomsoniae*

blimbing

*Averrhoa carambola*

blood flower

*Asclepias curassavica*

blue aloe

*Agave vera-cruz*

blue fountain bush

*Rotheca serrata*

blue gum

*Eucalyptus globulus*

blue pea

*Clitoria ternatea*

bluebird vine

*Verbena officinalis*

bo tree

*Ficus religiosa*

boat lily

*Tradescantia spathacea*

*bodhi nyaung*

*Ficus religiosa*

*bok-pyin*

*Diospyros malabarica*

*boktaung*

*Persicaria chinensis*

*Pteridium aquilinum*

bombax

*Bombax ceiba*

Bombay hemp

*Hibiscus cannabinus*

*bomma-yaza*

*Rauvolfia serpentina*

*bommayazar*

*Rauvolfia serpentina*

Bonavista bean

*Lablab purpureus*

*bonmathane-payoke*

*Blumea balsamifera*

boundary mark

*Cordyline fruticosa*

brake

*Pteridium aquilinum*

braken

*Pteridium aquilinum*

brank

*Fagopyrum esculentum*

Brazilian tea

*Stachytarpheta indica*

bread flower

*Vallaris solanacea*

bridal couch tree

*Hymenodictyon orixense*

broad-leaved plantain

*Plantago major*

*bu*

*Piper betle*

buckwheat

*Fagopyrum esculentum*

*budatharana*

*Canna indica*

*budi-nan*

*Mentha arvensis*

bur mallow

*Urena lobata*

Burma linseed

*Hygrophila phlomoides*

Burmese ironwood

*Xylia xylocarpa*

Burmese storax

*Altingia excelsa*

burrbush

*Triumfetta rhomboidea*

burrflower tree

*Neolamarckia cadamba*

*buru*

*Piper betle*

bush clock-vine

*Thunbergia erecta*

buttefly-weed

*Chromolaena odorata*

butterfly pea

*Clitoria ternatea*

butterfly tree

*Bauhinia purpurea*

butterfly weed

*Asclepias curassavica*

butterweed

*Senecio densiflorus*

*bwe-baung*

*Spondias pinnata*

*byin-gale*

*Memecylon edule*

*byu-chidauk*

*Rhizophora mucronata*

cabbage

*Brassica oleracea*

cadamba

*Neolamarckia cadamba*

Caesar weed

*Urena lobata*

cajeput

*Melaleuca cajuputi*

calamus

*Acorus calamus*

California cypress

*Cupressus goveniana*

camel’s foot tree

*Bauhinia purpurea*

camphor

*Cinnamomum camphora*

camphor tree

*Cinnamomum camphora*

cananga

*Cananga odorata*

candle bush

*Senna alata*

canna

*Canna indica*

cannonball mangrove

*Xylocarpus granatum*

*canone casaun*

*Allium cepa*

Canton ginger

*Zingiber officinale*

cape gooseberry

*Physalis peruviana*

caper bush

*Capparis flavicans*

capoc

*Ceiba pentandra*

carallia

*Carallia brachiata*

caramba

*Averrhoa carambola*

carambola

*Averrhoa carambola*

*caravalla*

*Cleome gynandra*

carrizo

*Phragmites karka*

cart-track-plant

*Plantago major*

*casauboh*

*Croton persimilis*

*casaun-phet-tine*

*Allium sativum*

cashew nut

*Anacardium occidentale*

cassie

*Acacia farnesiana*

castor bean

*Ricinus communis*

castor oil plant

*Ricinus communis*

casuarina

*Casuarina equisetifolia*

cat’s whiskers

*Orthosiphon aristatus*

catbrier

*Smilax aspera*

catechu

*Acacia catechu*

cayenne pepper

*Capsicum annuum*

ceiba

*Ceiba pentandra*

celery

*Apium graveolens*

Ceylon caper

*Capparis zeylanica*

Ceylon cinnamon

*Cinnamomum tamala*

Ceylon citronella

*Cymbopogon nardus*

Ceylon hydrolea

*Hydrolea zeylanica*

Ceylon ironwood

*Mesua ferrea*

Ceylon leadwort

*Plumbago zeylanica*

Ceylon oak

*Schleichera oleosa*

Ceylon rosewood

*Albizia odoratissima*

*cha li shu*

*Anneslea fragrans*

*chang zi ma quia*

*Strychnos wallichiana*

*cha-om*

*Acacia pennata*

chaulmoogra

*Hydnocarpus kurzii*

*chay-ahkya*

*Phyllanthus emblica*

*chaya-kaya*

*Aphanamixis polystachya*

*chayar pin*

*Mimusops elengi*

*chay-thee pin*

*Semecarpus anacardium*

*che*

*Semecarpus anacardium*

cheerojee-oil plant

*Buchanania lancifolia*

*chek-awn*

*Abrus precatorius*

cherry tomato

*Physalis peruviana*

chewing gum tree

*Manilkara zapota*

chicken eyes

*Abrus precatorius*

chicle tree

*Manilkara zapota*

chili pepper

*Capsicum annuum*

China grass

*Boehmeria nivea*

*chin-baung-gyi*

*Hibiscus cannabinus*

*chin-baung-kha*

*Hibiscus cannabinus*

*chinbaung-ni*

*Hibiscus sabdariffa*

*chin-bong*

*Hibiscus sabdariffa*

*chinebaune*

*Hibiscus sabdariffa*

Chinese banyan

*Ficus retusa*

Chinese bitter-cucumber

*Momordica cochinchinensis*

Chinese box tree

*Limonia acidissima*

Chinese date

*Ziziphus jujuba*

Chinese elder

*Sambucus javanica*

Chinese guger tree

*Schima wallichii*

Chinese honeysuckle

*Combretum indicum*

Chinese ixora

*Ixora chinensis*

Chinese jujube

*Ziziphus jujuba*

Chinese knotweed

*Persicaria chinensis*

Chinese mustard

*Sinapis alba*

Chinese parsley

*Coriandrum sativum*

Chinese silk plant

*Boehmeria nivea*

Chinese smartweed

*Persicaria chinensis*

Chinese waterspinach

*Ipomoea aquatica*

Chinese-cucumber

*Momordica cochinchinensis*

*chinyok*

*Garuga pinnata*

chirauli nut

*Buchanania lancifolia*

Chittagong wood

*Chukrasia tabularis*

chloranthus

*Chloranthus elatior*

*chota aura*

*Chamaecrista pumila*

*chyahkya*

*Phyllanthus emblica*

*chyamka*

*Magnolia champaca*

*chy-inbawngla*

*Zanthoxylum acanthopodium*

*chying-hkrang-ahpraw*

*Sinapis alba*

*chying-htawng-la*

*Colebrookea oppositifolia*

*chying-ma*

*Rhus chinensis*

cinnamon

*Cinnamomum verum*

citrine myrobalan

*Terminalia citrina*

citronella

*Cymbopogon nardus*

citronella grass

*Cymbopogon citratus*

*Cymbopogon nardus*

clammy cherry

*Cordia myxa*

claoxylon

*Claoxylon indicum*

clausena

*Clausena excavata*

clearing nut tree

*Strychnos potatorum*

*Swertia chirayita*

climbing lily

*Gloriosa superba*

climbing ylang-ylang

*Artabotrys hexapetalus*

clove

*Syzygium aromaticum*

clove tree

*Syzygium aromaticum*

clustered fishtail palm

*Caryota mitis*

cobra plant

*Amorphophallus paeoniifolius*

cobra’s saffron

*Mesua ferrea*

cock’s comb

*Celosia argentea*

cocklebur

*Agrimonia eupatoria*

coffee

*Coffea arabica*

common allamanda

*Allamanda cathartica*

common buckwheat

*Fagopyrum esculentum*

common cowitch

*Mucuna pruriens*

common emetic nut

*Catunaregam spinosa*

common foxglove

*Digitalis purpurea*

common ginger

*Zingiber officinale*

common ironwood

*Casuarina equisetifolia*

common jujube

*Ziziphus jujuba*

common plantain

*Plantago major*

common purslane

*Portulaca oleracea*

common reed

*Phragmites karka*

common sage

*Salvia officinalis*

common sesban

*Sesbania sesban*

congo-jute

*Urena lobata*

copperleaf

*Acalypha indica*

*Acalypha wilkesiana*

coral bush

*Jatropha multifida*

coral jasmine

*Nyctanthes arbor-tristis*

coral pea

*Adenanthera pavonina*

coral wood

*Adenanthera pavonina*

cordate monochoria

*Monochoria vaginalis*

coriander

*Coriandrum sativum*

corn mint

*Mentha arvensis*

corn

*Zea mays*

cotton tree

*Gossypium hirsutum*

country fig

*Ficus hispida*

country gooseberry

*Averrhoa carambola*

cow soapwort

*Vaccaria hispanica*

cowcockle

*Vaccaria hispanica*

cowhage

*Mucuna pruriens*

cowherb

*Vaccaria hispanica*

cowitch

*Mucuna pruriens*

crab eyes

*Abrus precatorius*

crape gardenia

*Tabernaemontana divaricate*

crape jasmine

*Tabernaemontana divaricate*

creat

*Andrographis paniculata*

creeper

*Trichosanthes tricuspidata*

crepe ginger

*Cheilocostus speciosus*

crested cock’s comb

*Celosia argentea*

creyat root

*Andrographis paniculata*

*crot-kyeei*

*Carica papaya*

croton

*Croton persimilis*

crown flower

*Calotropis gigantea*

cubeb pepper

*Piper cubeba*

cucha cara

*Elephantopus scaber*

cucumber

*Cucumis sativus*

cultivated celery

*Apium graveolens*

curltop ladysthumb

*Persicaria pulchra*

curlytop knotweed

*Persicaria pulchra*

curlytop smartweed

*Persicaria pulchra*

custard apple

*Annona squamosa*

cutch

*Acacia catechu*

cycad

*Cycas rumphii*

cynometra

*Cynometra ramiflora*

*da-ma-nge*

*Spatholobus parviflorus*

*dana-thuka*

*Scoparia dulcis*

dancing daisy

*Tanacetum cinerariifolium*

*dandagu*

*Dracaena angustifolia*

*dan-da-lun*

*Moringa oleifera*

*danghkyam kaba*

*Holarrhena pubescens*

*danghkyam-kaii*

*Wrightia arborea*

*dangkyam*

*Holarrhena pubescens*

*dangywe*

*Senna italica*

*Senna tora*

*dan-la-ku*

*Dracaena angustifolia*

*dantalet*

*Dracaena angustifolia*

*dant-kywei*

*Senna tora*

*danyin*

*Archidendron jiringa*

*dap*

*Terminalia tomentosa*

*dar-na-thu-kha*

*Scoparia dulcis*

dasheen

*Colocasia antiquorum*

*dauk-yut*

*Symplocos racemosa*

*daung-satpya*

*Callicarpa macrophylla*

*daung-sok*

*Caesalpinia pulcherrima*

*daungyan*

*Garcinia xanthochymus*

*dawe-hmaing-nwe*

*Combretum indicum*

*daw-hke*

*Clausena excavata*

*dawyan-ban*

*Garcinia xanthochymus*

dayflower

*Commelina paludosa*

Deckaner hemp

*Hibiscus cannabinus*

deer’s foot

*Convolvulus arvensis*

devil tree

*Alstonia scholaris*

devil’s claw

*Martynia annua*

devil’s cotton

*Abroma augustum*

devil’s horsewhip

*Achyranthes aspera*

devil’s plague

*Daucus carota*

devil’s trumpet

*Datura metel*

*dewali-pan*

*Tagetes erecta*

*dheniaani*

*Olax scandens*

*didok-chi*

*Dactyloctenium aegyptium*

diffuse hogweed

*Commicarpus chinensis*

digitalis

*Digitalis purpurea*

dill

*Anethum graveolens*

*dinghkri*

*Senna tora*

*ding-kok*

*Spondias pinnata*

dita bark

*Alstonia scholaris*

divine herb

*Sigesbeckia orientalis*

*do*

*Entada phaseoloides*

dock-leaf smartweed

*Persicaria pulchra*

dodder

*Cuscuta reflexa*

dog bush

*Blumea balsamifera*

dog fruit

*Archidendron jiringa*

dogtail

*Buddleja asiatica*

*dona-ban*

*Artemisia dracunculus*

donka

*Sandoricum koetjape*

downy jasmine

*Jasminum multiflorum*

dragon’s blood

*Cordyline fruticosa*

drooping fig

*Ficus semicordata*

drumstick tree

*Moringa oleifera*

duckweed

*Portulaca oleracea*

*dumsa-gyaw*

*Hymenodictyon orixense*

Dutchman’s pipe

*Aristolochia tagala*

*dwabok*

*Kydia calycina*

dwarf copperleaf

*Alternanthera sessilis*

dwarf poinciana

*Caesalpinia pulcherrima*

dwarf white bauhinia

*Bauhinia acuminata*

dysentery bush

*Grewia polygama*

eaglewood

*Aquilaria malaccensis*

earth nut

*Arachis hypogaea*

East Indian rosebay

*Tabernaemontana divaricate*

East Indian screw tree

*Helicteres isora*

eclipta

*Eclipta prostrata*

eddo

*Colocasia antiquorum*

*egayit*

*Mayodendron igneum*

*egayit-ni*

*Mayodendron igneum*

eggplant

*Solanum melongena*

Egyptian bean

*Lablab purpureus*

Egyptian grass

*Dactyloctenium aegyptium*

Egyptian rattlepod

*Sesbania sesban*

*eik-thara*

*Aristolochia indica*

*eik-tha-ra-muli*

*Aristolochia indica*

elderberry

*Sambucus javanica*

elephant apple

*Dillenia indica*

*Limonia acidissima*

elephant yam

*Amorphophallus paeoniifolius*

elephantopus

*Elephantopus scaber*

emblic

*Phyllanthus emblica*

emetic nut

*Catunaregam spinosa*

empress candle plant

*Senna alata*

emu-berry

*Grewia polygama*

*eng-si*

*Ziziphus jujuba*

estragon

*Artemisia dracunculus*

European dill

*Anethum graveolens*

exile oleander

*Cascabela thevetia*

falsa

*Grewia asiatica*

false ashoka

*Polyalthia longifolia*

false daisy

*Eclipta prostrata*

false dogwood

*Sapindus saponaria*

false pareira brava

*Cissampelos pareira*

false saffron

*Carthamus tinctorius*

false tarragon

*Artemisia dracunculus*

fennel

*Foeniculum vulgare*

fern asparagus

*Asparagus filicinus*

fetid passionflower

*Passiflora foetida*

fever grass

*Cymbopogon citratus*

field bindweed

*Convolvulus arvensis*

field mint

*Mentha arvensis*

fire plant

*Plumbago indica*

firedragon

*Acalypha wilkesiana*

fire-flame bush

*Woodfordia fruticosa*

fish poison climber

*Millettia pachycarpa*

fishtail palm

*Caryota mitis*

five-leaved chaste tree

*Vitex negundo*

five-leaved yam

*Dioscorea pentaphylla*

flagroot

*Acorus calamus*

flamboyant

*Delonix regia*

flame lily

*Gloriosa superba*

flame-of-the-forest

*Butea monosperma*

flame-of-the-woods

*Ixora coccinea*

flat-top mille grains

*Oldenlandia corymbosa*

flax

*Linum usitatissimum*

fleshy spurge

*Euphorbia antiquorum*

floppers

*Bryophyllum pinnatum*

*flor-de-joja*

*Phyllanthus niruri*

four o’clock

*Mirabilis jalapa*

fragrant padri-tree

*Stereospermum chelonoides*

frangipani

*Plumeria rubra*

French tarragon

*Artemisia dracunculus*

freshwater mangrove

*Carallia brachiata*

fringed hibiscus

*Hibiscus schizopetalus*

fritillaria

*Fritillaria cirrhosa*

*gaiyin*

*Momordica charantia*

gale of the wind

*Phyllanthus niruri*

gallow grass

*Cannabis sativa*

*gamone-kyet-thon-phyu*

*Fritillaria cirrhosa*

*gamon-kyeethun-phyu*

*Fritillaria cirrhosa*

*gangala*

*Cleome gynandra*

*Spermacoce hispida*

*gangawlwe*

*Anneslea fragrans*

garcinia

*Garcinia xanthochymus*

garden onion

*Allium cepa*

garden purslane

*Portulaca oleracea*

garden quinine

*Volkameria inermis*

garden sage

*Salvia officinalis*

gargu

*Heynea trijuga*

garlic

*Allium sativum*

garuga

*Garuga pinnata*

*gau-gau*

*Mesua ferrea*

*gawhgu*

*Cassia fistula*

giant dodder

*Cuscuta reflexa*

giant granadilla

*Passiflora quadrangularis*

giant reed

*Arundo donax*

giant swallowart

*Dregea volubilis*

giant thorny bamboo

*Bambusa bambos*

*ginbeik*

*Basella alba*

glabrous greenbrier

*Smilax glabra*

glory bower

*Volkameria inermis*

glory tree

*Clerodendrum thomsoniae*

gmelina

*Gmelina arborea*

goat’s foot creeper

*Ipomoea pes-caprae*

goatweed

*Ageratum conyzoides*

gold mohur

*Delonix regia*

golden apple

*Aegle marmelos*

golden cassia

*Senna italica*

golden champak

*Magnolia champaca*

golden mahogany

*Chukrasia tabularis*

golden shower tree

*Cassia fistula*

golden trumpet

*Allamanda cathartica*

goldenberry

*Physalis peruviana*

goldthread

*Coptis teeta*

*gon*

*Boehmeria nivea*

*gon-nyin*

*Entada phaseoloides*

gooseberry tree

*Phyllanthus acidus*

goosefoot

*Chenopodium album*

grass nut

*Arachis hypogaea*

grass

*Cannabis sativa*

gray mangrove

*Avicennia officinalis*

great bougainvillea

*Bougainvillea spectabilis*

great plantain

*Plantago major*

greater galangal

*Alpinia galanga*

Grecian foxglove

*Digitalis lanata*

green champa

*Polyalthia longifolia*

green ripple peacock ginger

*Kaempferia elegans*

green sagewart

*Artemisia dracunculus*

greenbrier

*Smilax aspera*

ground cherry

*Physalis peruviana*

groundnut

*Arachis hypogaea*

guava

*Psidium guajava*

guaymochil

*Pithecellobium dulce*

guest tree

*Kleinhovia hospita*

gum-arabic

*Acacia nilotica*

gumhar

*Gmelina arborea*

gum-lac

*Schleichera oleosa*

gumma

*Leucas cephalotes*

*guntgaw*

*Mesua ferrea*

*gwe*

*Spondias pinnata*

*gwedauk-nwe*

*Dregea volubilis*

*gwi-lakajawng*

*Girardinia diversifolia*

*gwin-nge*

*Mucuna pruriens*

*gyee baitwine*

*Trachyspermum ammi*

*gyin*

*Zingiber officinale*

*gyo*

*Schleichera oleosa*

*gyo-pan*

*Flemingia chappar*

*gyo-sagauk*

*Chrozophora plicata*

hairy fig

*Ficus hispida*

hairy indigo

*Grewia hirsuta*

*hangnan*

*Acacia pennata*

*haprut*

*Dillenia indica*

harvest-lice

*Agrimonia eupatoria*

*haw hkan kaju*

*Solanum anguivi*

*ha-yung*

*Croton persimilis*

heart’s pea

*Cardiospermum halicacabum*

heart-leaved moonseed

*Tinospora cordifolia*

heart-seed

*Cardiospermum halicacabum*

hedge euphorbia

*Euphorbia neriifolia*

heynea

*Walsura pinnata*

hibiscus burr

*Urena lobata*

*hi-ga-yone*

*Mimosa pudica*

hill glory bower

*Clerodendrum infortunatum*

Himalayan nettle

*Girardinia diversifolia*

Himalayan wild cherry

*Prunus cerasoides*

Hindu datura

*Datura metel*

*hingala*

*Cleome gynandra*

*hingalar*

*Oldenlandia corymbosa*

*hing-hang*

*Acacia concinna*

*hingut-pho*

*Stereospermum colais*

*hingut-po*

*Stereospermum colais*

*hin-nu-new-subauk*

*Amaranthus spinosus*

*hin-nu-nwe*

*Amaranthus cruentus*

hiptage

*Hiptage benghalensis*

*hkajang-nai*

*Streblus asper*

*hkala-shwang*

*Neolamarckia cadamba*

*hkam mai*

*Phyllanthus emblica*

*hkamari*

*Salix tetrasperma*

*hkang-awng*

*Antiaris toxicaria*

*hka-shatawi*

*Bischofia javanica*

*hko-ma-awn*

*Mucuna pruriens*

*hko-mak-awa*

*Mucuna pruriens*

*hkum-bang-pan*

*Cymbopogon citratus*

*hla brairot*

*Justicia adhatoda*

*hla cawi bactine*

*Coccinia grandis*

*hla ponyork*

*Morinda angustifolia*

*hla pruckkha*

*Acacia concinna*

*hla-crote kyee*

*Carica papaya*

*hlahnip chai*

*Centella asiatica*

*hla-kanin kyam*

*Ipomoea alba*

*hla-parite-baikayah*

*Citrus limon*

*hla-pruck-hka-hnoke*

*Acacia pennata*

*hle-kanan*

*Bridelia retusa*

*hmaing*

*Dodonaea viscosa*

*hmam-gyi*

*Sesamum indicum*

*hmandaw*

*Garcinia xanthochymus*

*hman-phyu*

*Tamilnadia uliginosa*

*hman-thein*

*Cinnamomum bejolghota*

*hmanthin*

*Cinnamomum verum*

*hmaw-yan*

*Strobilanthes auriculatus*

*hmya-seik*

*Antiaris toxicaria*

*hnan*

*Sesamum indicum*

*hnan-kyat*

*Linum usitatissimum*

*hnaw*

*Haldina cordifolia*

*hnget-chauk*

*Eucalyptus globulus*

*hnin-thi-pin*

*Syzygium jambos*

hoary basil

*Ocimum americanum*

hog plum

*Spondias pinnata*

hog-pasture brake

*Pteridium aquilinum*

holly-leaved acanthus

*Acanthus ilicifolius*

holy basil

*Ocimum tenuiflorum*

*hon*

*Millettia pachycarpa*

hopseed bush

*Dodonaea viscosa*

hopseed

*Dodonaea viscosa*

horn of plenty

*Datura metel*

horse bean

*Canavalia ensiformis*

horseradish tree

*Moringa oleifera*

hot pepper

*Capsicum annuum*

*hou-no*

*Careya arborea*

*hpadawng*

*Mallotus philippensis*

*hpah-ha*

*Acacia concinna*

*hpak ha*

*Acacia concinna*

*hpak-ha-awn*

*Acacia pennata*

*hpak-ko-suk*

*Celastrus paniculatus*

*hpak-lam-mon-long*

*Senna alata*

*hpak-mong*

*Cordia dichotoma*

*hpak-se-saw*

*Momordica cochinchinensis*

*hpak-si-so*

*Premna amplectens*

*hpan-kha-ngai*

*Terminalia citrina*

*hpan-khar-thee*

*Terminalia chebula*

*hparlar hpyu*

*Elettaria cardamomum*

*hpat*

*Xylia xylocarpa*

*hpaung-hpaung-thi*

*Physalis peruviana*

*hpawng-awn*

*Mallotus philippensis*

*hpon-mathein*

*Blumea balsamifera*

*hpun ja*

*Aegle marmelos*

*hpun*

*Carallia brachiata*

*hpun-hpawk*

*Mayodendron igneum*

*hpunnam-makawk*

*Spondias pinnata*

*hroirwk*

*Terminalia bellirica*

*hsan-to-nouk*

*Glycine max*

*hsanwin*

*Curcuma longa*

*hsay gandamar*

*Tanacetum cinerariifolium*

*hsay min kyaung*

*Euphorbia hirta*

*hsay-dan*

*Hygrophila phlomoides*

*hsay-kha gyi*

*Andrographis paniculata*

*hsay-kyaw gyi*

*Plantago major*

hsee mee-tauk

*Gloriosa superba*

*hsee-cho*

*Orthosiphon aristatus*

*hseik hpalu*

*Nyctanthes arbor-tristis*

*hset-hnayarthi*

*Cascabela thevetia*

*hsin-doan manwai*

*Tinospora cordifolia*

*hsu bok gyi*

*Acacia pennata*

*hsu pan*

*Carthamus tinctorius*

*htakyu*

*Phyllanthus emblica*

*htamone-chort*

*Millingtonia hortensis*

*hta-muck*

*Mimosa pudica*

*hta-nah*

*Zingiber montanum*

*htaura*

*Acacia pennata*

*htingra-hpraw*

*Justicia adhatoda*

hyacinth bean

*Lablab purpureus*

*hyang*

*Alnus nepalensis*

hygrophila

*Hygrophila auriculata*

iceplant

*Martynia annua*

Indian acalypha

*Acalypha indica*

Indian almond

*Terminalia catappa*

Indian bael

*Aegle marmelos*

Indian bean

*Lablab purpureus*

Indian birthwort

*Aristolochia indica*

*Aristolochia tagala*

Indian cherry

*Cordia myxa*

Indian coral tree

*Erythrina variegata*

Indian cork tree

*Millingtonia hortensis*

Indian dill

*Anethum graveolens*

Indian elm

*Holoptelea integrifolia*

Indian fir tree

*Polyalthia longifolia*

Indian gentian

*Swertia chirayita*

Indian goldthread

*Coptis teeta*

Indian gum tree

*Acacia nilotica*

Indian heliotrope

*Heliotropium indicum*

Indian hemp

*Abroma augustum*

*Hibiscus cannabinus*

Indian jalap

*Ipomoea alba*

Indian kamala

*Mallotus philippensis*

Indian kapok

*Bombax ceiba*

Indian laburnum

*Cassia fistula*

Indian laurel

*Calophyllum inophyllum*

*Ficus retusa*

Indian lilac

*Azadirachta indica*

Indian long pepper

*Piper longum*

Indian madder

*Rubia cordifolia*

Indian mast tree

*Polyalthia longifolia*

Indian mulberry

*Morinda citrifolia*

Indian nightshade

*Solanum anguivi*

Indian oak

*Barringtonia acutangula*

Indian pennywort

*Centella asiatica*

Indian persimmon

*Diospyros malabarica*

Indian privit

*Vitex negundo*

Indian red water-lily

*Nymphaea rubra*

Indian rhododendron

*Melastoma malabathricum*

Indian rose-chestnut

*Mesua ferrea*

Indian sandalwood

*Santalum album*

Indian senna

*Senna alexandrina*

Indian shot

*Canna indica*

Indian snakeroot

*Rauvolfia serpentina*

Indian sorrel

*Hibiscus sabdariffa*

Indian spinach

*Basella alba*

Indian spiral ginger

*Cheilocostus speciosus*

Indian spurgetree

*Euphorbia neriifolia*

Indian squirrel tail

*Colebrookea oppositifolia*

Indian trumpet flower

*Oroxylum indicum*

Indian weed

*Sigesbeckia orientalis*

Indian wild pepper

*Vitex trifolia*

Indian-gooseberry

*Phyllanthus emblica*

*in-kathit*

*Erythrina variegata*

ironwood tree

*Memecylon edule*

*Mesua ferrea*

irul

*Xylia xylocarpa*

ivy gourd

*Coccinia grandis*

iwarancusa grass

*Cymbopogon jwarancusa*

jaboncillo

*Sapindus saponaria*

jack bean

*Canavalia ensiformis*

jack in the bush

*Chromolaena odorata*

jackfruit

*Artocarpus heterophyllus*

Jacob’s coat

*Acalypha wilkesiana*

jail

*Lannea coromandelica*

*jai-nool*

*Mesua ferrea*

Jamaica sorrel

*Hibiscus sabdariffa*

*jamani-chon*

*Chromolaena odorata*

jambolan plum

*Syzygium cumini*

jambu

*Syzygium cumini*

Jamestown weed

*Datura stramonium*

*janah lapoot*

*Acacia concinna*

*jangbawngla*

*Zanthoxylum acanthopodium*

japanese mint

*Mentha arvensis*

Japanese pepper

*Zanthoxylum acanthopodium*

jasmine tree

*Millingtonia hortensis*

Java flower

*Ficus benjamina*

Java pepper

*Piper cubeba*

Java plum

*Syzygium cumini*

Java tea

*Orthosiphon aristatus*

Javanese elderberry

*Sambucus javanica*

*jaw-gale*

*Delonix regia*

jequirity

*Abrus precatorius*

jhingam poma

*Lannea coromandelica*

jhingam

*Lannea coromandelica*

jimson weed

*Datura stramonium*

*jingbawngla*

*Zanthoxylum acanthopodium*

Job’s tears

*Coix lacryma-jobi*

joyweed

*Alternanthera sessilis*

*jujube*

*Ziziphus jujuba*

jungle flame ixora

*Ixora coccinea*

jungle geranium

*Ixora coccinea*

*jyula*

*Mussaenda macrophylla*

*ka-aung*

*Ficus hispida*

*kabwi*

*Casuarina equisetifolia*

kadam tree

*Neolamarckia cadamba*

*kadat-ngan*

*Artabotrys hexapetalus*

*Cananga odorata*

*kadauk-sat*

*Monochoria vaginalis*

*kadawn*

*Jasminum multiflorum*

*kadawnla*

*Jasminum multiflorum*

*kado-po*

*Ageratum conyzoides*

*kadu-hpo*

*Ageratum conyzoides*

*kadung*

*Bombax ceiba*

*kadu-pyan*

*Cyanthillium cinereum*

*kadut*

*Ficus hispida*

*ka-dut*

*Ficus semicordata*

*kaisun*

*Allium cepa*

*kal*

*Cordia dichotoma*

*kalabin*

*Kopsia fruticosa*

*kala-magyi*

*Pithecellobium dulce*

*ka-la-mak*

*Mansonia gagei*

*kala-met*

*Mansonia gagei*

*kala-myetsi*

*Cardiospermum halicacabum*

*kala-myetsi-pinzauk-gyi*

*Physalis peruviana*

*kala-pan*

*Tagetes erecta*

*kala-pi-sein*

*Ocimum tenuiflorum*

*kalaw*

*Hydnocarpus kurzii*

*kalaw-so*

*Hydnocarpus kurzii*

*ka-leik*

*Coix lacryma-jobi*

*kalein*

*Coix lacryma-jobi*

*kalein-thi*

*Coix lacryma-jobi*

kalisar

*Ichnocarpus frutescens*

kalo

*Colocasia antiquorum*

*kam kan*

*Mesua ferrea*

kamala tree

*Mallotus philippensis*

*kame*

*Quassia indica*

*kanack champa*

*Pterospermum acerifolium*

*kana-hpaw*

*Enydra fluctuans*

*kanah-tanow pryin*

*Euphorbia hirta*

*kanakho*

*Croton tiglium*

*kangyok*

*Plumbago indica*

*kan-gyok-phyu*

*Plumbago zeylanica*

kannyut

*Asparagus officinalis*

*kant-balu*

*Trachyspermum roxburghianum*

*kant-choke-ni*

*Plumbago indica*

*kan-tin*

*Indigofera cassioides*

*ka-nyut*

*Asparagus filicinus*

*kao mai*

*Butea monosperma*

*kao-hko*

*Butea superba*

*ka-phi*

*Coffea arabica*

kapok bush

*Aerva javanica*

kapok

*Ceiba pentandra*

*kasawt-kha*

*Solanum anguivi*

*kashit-ka*

*Toona sureni*

*kasondeh*

*Cordia dichotoma*

kassod tree

*Senna siamea*

*katcho*

*Smilax guianensis*

*katcho-gyi*

*Smilax glabra*

*kathaw-pok*

*Senna italica*

*kathit*

*Erythrina variegata*

kathu

*Indigofera cassioides*

*kat-say-nei*

*Urena lobata*

*katsi-ne*

*Sida spinosa*

*kat-si-ne*

*Triumfetta rhomboidea*

*kat-sine*

*Urena lobata*

*katsine-galay*

*Triumfetta rhomboidea*

*ka-tu-pin*

*Elephantopus scaber*

*kauk-hlaing-ti*

*Aeginetia indica*

*kauk-yoe nwai*

*Convolvulus arvensis*

*kauk-yo-nwe*

*Convolvulus arvensis*

*kawain-hnoot*

*Alpinia galanga*

*kawaintoot*

*Alpinia officinarum*

*kawe-thi*

*Luffa cylindrica*

*kawl-tung-peng*

*Bombax ceiba*

*kaw-ta-nook*

*Mesua ferrea*

*kaya*

*Mimosa pudica*

*kaya-chon*

*Acanthus ilicifolius*

*kayan*

*Solanum melongena*

*kayan-kazaw*

*Solanum rudepannum*

*kazaw-kha*

*Solanum rudepannum*

*ka-zo*

*Cassia fistula*

*kazun yoe-n*

*Ipomoea aquatica*

*kazun-galay*

*Ipomoea aquatica*

*kazun-ywet*

*Ipomoea aquatica*

*kenaf*

*Hibiscus cannabinus*

key lime

*Citrus aurantiifolia*

*khabaung yay-kyi*

*Strychnos potatorum*

*khaing-shwe-wa*

*Berberis nepalensis*

*khan tauk*

*Coptis teeta*

*khan*

*Carissa spinarum*

*khanzat*

*Carissa spinarum*

*khar-grope*

*Amaranthus spinosus*

*khaung-yan*

*Hibiscus schizopetalus*

*khaung-yan-ywet-hla*

*Hibiscus schizopetalus*

*khaya*

*Argemone mexicana*

*khayan-kazaw-kha*

*Solanum anguivi*

*kha-yar*

*Acanthus ilicifolius*

*kha-yar-chon*

*Acanthus ilicifolius*

*khayay pin*

*Mimusops elengi*

*khinbok*

*Vallaris solanacea*

*khine-shwe-war*

*Berberis nepalensis*

*khu-than*

*Hymenodictyon orixense*

*khwar-nyo-gyi*

*Clematis smilacifolia*

*khwati*

*Dillenia indica*

*khwe-laya*

*Mucuna pruriens*

*khwele*

*Mucuna pruriens*

*khwe-ya*

*Mucuna pruriens*

*kia-bok*

*Aegle marmelos*

kidney cotton

*Gossypium barbadense*

kidney tea plant

*Orthosiphon aristatus*

*kikao*

*Butea monosperma*

*kin peint*

*Basella alba*

*kin pone*

*Coccinia grandis*

king of bitters

*Andrographis paniculata*

*kinmon*

*Coccinia grandis*

*kinmun-gyin*

*Acacia concinna*

*kin-pun chin*

*Acacia concinna*

*kin-thabut-gyi*

*Chukrasia tabularis*

kitchen sage

*Salvia officinalis*

*klaw*

*Aquilaria malaccensis*

*klor*

*Tectona grandis*

*kobi-dok*

*Brassica oleracea*

kohlrabi

*Brassica oleracea*

*kokko*

*Albizia lebbeck*

*kone-line*

*Cordyline fruticosa*

*kon-kado*

*Abelmoschus moschatus*

*kon-thabye*

*Syzygium nervosum*

*kosot-lot*

*Butea superba*

*koyan-gyi*

*Crinum asiaticum*

kratom

*Mitragyna speciosa*

*krek*

*Mangifera indica*

*kroik*

*Bombax ceiba*

*kruk*

*Mangifera indica*

*ku-hlu*

*Phyllanthus emblica*

*ku-ku*

*Smilax guianensis*

*kum-bomb-kroke*

*Apium graveolens*

*kun*

*Euphorbia antiquorum*

*Piper betle*

*kun-kado*

*Kaempferia elegans*

*kun-linne*

*Cordyline fruticosa*

*kunsa-gamon*

*Alpinia galanga*

*kwa-nyo*

*Thunbergia erecta*

*kway*

*Dioscorea bulbifera*

*kway-tauk nwai*

*Dregea volubilis*

*kwe*

*Markhamia stipulata*

*kwet*

*Limonia acidissima*

*kyabahon*

*Flemingia chappar*

*kyahin*

*Ipomoea alba*

*kya-hpetgyi*

*Leea macrophylla*

*kyakat-wa*

*Bambusa bambos*

*kyana*

*Xylocarpus moluccensis*

*kyan-hin pin*

*Ipomoea alba*

*kya-ni*

*Nymphaea rubra*

*kya-sha*

*Boehmeria nivea*

*kya-su*

*Terminalia citrina*

*kyate-hman*

*Eclipta prostrata*

*kyat-nan*

*Xylocarpus moluccensis*

*kyauk-hkwe-pin*

*Evolvulus alsinoides*

*kyauk-pha-yon*

*Benincasa hispida*

*kyauk-tinyu*

*Taxus baccata*

*kyaung shar*

*Oroxylum indicum*

*kyaungban-gyi*

*Vitex negundo*

*kyaung-migo*

*Buddleja asiatica*

*kyaung-pan*

*Vitex trifolia*

*kyaung-se-pin*

*Acalypha indica*

*kyaung-yo-thay pin*

*Acalypha indica*

*kyaung-yo-the*

*Acalypha indica*

kydia

*Kydia calycina*

*kyee-arh pin*

*Trichosanthes tricuspidata*

*kyeik*

*Coix lacryma-jobi*

*kyeik-hman*

*Eclipta prostrata*

*kyet-gale*

*Melastoma malabathricum*

*kyet-hinga*

*Momordica charantia*

*kyet-hin-kha*

*Momordica charantia*

*kyet-hsu yoe-ni*

*Ricinus communis*

*kyet-hsu*

*Ricinus communis*

*kyet-kadut*

*Ficus benjamina*

*kyet-kadut*

*Ficus semicordata*

*kyet-ma-oak*

*Ardisia humilis*

*kyet-maok*

*Ardisia humilis*

*kyet-mauk*

*Celosia argentea*

*kyetmauk*

*Litchi chinensis*

*kyet-mauk-pyan*

*Achyranthes aspera*

*kyet-mauk-sue-pyan*

*Achyranthes aspera*

*kyetsi-gyi*

*Jatropha curcas*

*kyetsu*

*Ricinus communis*

*kyet-su-gyi*

*Jatropha curcas*

*kyetsu-kanako*

*Jatropha gossypiifolia*

*kyet-tayaw*

*Grewia hirsuta*

*kyet-tha-hin*

*Phyllanthus niruri*

*kyet-thun hpyu*

*Allium sativum*

*kyet-thun-ni oo-gyi*

*Allium cepa*

*kyetyo*

*Premna mollissima*

*kyetyo-po*

*Flacourtia jangomas*

*kyi*

*Barringtonia acutangula*

*kyibaung*

*Viscum cruciatum*

*kyi-kan-hnok-thi*

*Thunbergia laurifolia*

*kyi-ni*

*Barringtonia acutangula*

*kyini-nwe*

*Thunbergia laurifolia*

*kyom par*

*Magnolia champaca*

*kyu*

*Arundo donax*

*Phragmites karka*

*kyu-a*

*Phragmites karka*

*kyu-kaing*

*Phragmites karka*

*kyu-ma*

*Arundo donax*

*kyun*

*Tectona grandis*

*kyun-nalin*

*Callicarpa macrophylla*

*Premna mollissima*

*kyun-pin*

*Tectona grandis*

*kyu-wa-kaing*

*Phragmites karka*

*kywai-kyaung min hsay*

*Euphorbia hirta*

*kywai-kyaung min thay*

*Euphorbia hirta*

*kyway-u*

*Dioscorea pentaphylla*

*kywe-ma-gyo-lein*

*Stereospermum colais*

*kywe-tho*

*Bischofia javanica*

*kywe-thwe*

*Premna serratifolia*

*kywet-nabaung*

*Cissampelos pareira*

*kywe-yan-nge*

*Volkameria inermis*

*labanru*

*Spatholobus parviflorus*

lablab bean

*Lablab purpureus*

lac tree

*Schleichera oleosa*

*lacow-sacopf*

*Zingiber officinale*

lady’s finger

*Abelmoschus esculentus*

*lagat*

*Ficus religiosa*

*lagoe-htaneg*

*Zingiber officinale*

*lahkylk*

*Callicarpa macrophylla*

*lamai*

*Ficus semicordata*

lambsquarters

*Chenopodium album*

lamtoro

*Leucaena leucocephala*

*la-mung*

*Mangifera indica*

landrina

*Spermacoce hispida*

*lan-salat*

*Zanthoxylum acanthopodium*

*lan-tama*

*Polyalthia longifolia*

lantana

Lantana × aculeata

*laran*

*Magnolia champaca*

laran

*Neolamarckia cadamba*

*lash-awng*

*Neolamarckia cadamba*

*lashen*

*Boehmeria nivea*

*lashi*

*Tabernaemontana divaricate*

*latang*

*Lannea coromandelica*

*latloot*

*Morinda angustifolia*

*latsai*

*Toona sureni*

*lauk-thay*

*Tadehagi triquetrum*

*laukya*

*Schima wallichii*

*laukya-byu*

*Schima wallichii*

*laupe*

*Lannea coromandelica*

laurel

*Ficus benjamina*

laurel clock vine

*Thunbergia laurifolia*

laurel-leaved clockvine

*Thunbergia laurifolia*

laurel-leaved thunbergia

*Thunbergia laurifolia*

laurel-wood

*Calophyllum inophyllum*

*lawihkri-shalwai*

*Citrus aurantiifolia*

*lay-hnyin*

*Syzygium aromaticum*

*lay-naryi pan*

*Mirabilis jalapa*

leaf of life

*Bryophyllum pinnatum*

leea

*Leea macrophylla*

leechee

*Litchi chinensis*

*lee-ko-kee*

*Memecylon edule*

Leichhardt-pine

*Nauclea orientalis*

*leik tha-shwe war*

*Barleria prionitis*

*leik-hsu shwe*

*Barleria prionitis*

*leik-su-ywe*

*Barleria prionitis*

*lein-maw*

Citrus × aurantium

*lelu*

*Mussaenda macrophylla*

*le-moh-pin*

*Ceiba pentandra*

lemon

*Citrus limon*

lemon grass

*Cymbopogon citratus*

*le-padauk*

*Monochoria vaginalis*

*le-padu*

*Hygrophila auriculata*

*lè-seik-shin*

*Crateva religiosa*

*le-seik-shin*

*Quassia indica*

lesser cardamon

*Elettaria cardamomum*

lesser galangal

*Alpinia officinarum*

*letpan*

*Bombax ceiba*

*letpan-ga*

*Alstonia scholaris*

*let-pau*

*Bombax ceiba*

*lettok-thein*

*Wrightia arborea*

leucaena

*Leucaena leucocephala*

*lewah*

*Ceiba pentandra*

liane savon

*Gouania leptostachya*

licorice weed

*Scoparia dulcis*

life plant

*Bryophyllum pinnatum*

lilac tasselflower

*Emilia sonchifolia*

lime

*Citrus aurantiifolia*

*linda-pabyin*

*Melastoma malabathricum*

*lin-lay*

*Acorus calamus*

*lin-ne*

*Acorus calamus*

linseed

*Linum usitatissimum*

linwheel flower

*Tabernaemontana divaricate*

lipstick-tree

*Bixa orellana*

litchi

*Litchi chinensis*

litchi nut

*Litchi chinensis*

little hogweed

*Portulaca oleracea*

little ironweed

*Cyanthillium cinereum*

lolly fruit

*Sandoricum koetjape*

long zedoary

*Curcuma zedoaria*

loosestrife

*Woodfordia fruticosa*

lovage

*Trachyspermum ammi*

love-in-a-mist

*Passiflora foetida*

low shoebutton

*Ardisia humilis*

lubia bean

*Lablab purpureus*

lucky nut

*Cascabela thevetia*

luffa

*Luffa cylindrica*

*lulin-gyaw*

*Cinnamomum bejolghota*

*lun-tha*

*Benincasa hispida*

lychee

*Litchi chinensis*

mace

*Myristica fragrans*

*machit oo*

*Fritillaria cirrhosa*

*ma-chyangai*

*Cratoxylum formosum*

*machyit*

*Fritillaria cirrhosa*

mad apple

*Datura stramonium*

Madagascar periwinkle

*Catharanthus roseus*

*madaw*

*Garcinia xanthochymus*

madras wormwood

*Grangea maderaspatana*

*magan*

*Kydia calycina*

*magan-kaja*

*Kydia calycina*

*magap*

*Kydia calycina*

*magwinapa*

*Pterospermum acerifolium*

*magyeng*

*Tamarindus indica*

*ma-gyi*

*Tamarindus indica*

*magyi-bauk*

*Sapindus saponaria*

*mahaga-kyansit*

*Persicaria pulchra*

*maha-gar-kyan-sit*

*Persicaria chinensis*

*maha-hlega-byu*

*Bauhinia acuminata*

*maha-hlega-byu*

*Bauhinia purpurea*

*maha-hlega-ni*

*Bauhinia purpurea*

*mahahlega-phyu*

*Bauhinia acuminata*

*mahkaw*

*Ziziphus jujuba*

*ma-hlwa*

*Markhamia stipulata*

mahonia

*Berberis nepalensis*

*mahuya-pein*

*Colocasia antiquorum*

*maiaw*

*Arundo donax*

*mai-aw-awn*

*Arundo donax*

*mai-awza*

*Annona squamosa*

*mai-bau*

*Alnus nepalensis*

*mai-chek*

*Adenanthera pavonina*

*mai-hen*

*Terminalia bellirica*

*mai-hkai*

*Salix tetrasperma*

*mai-hkam*

*Lannea coromandelica*

*mai-hkang*

*Croton tiglium*

*mai-hkao*

*Schleichera oleosa*

*mai-hkao-long*

*Holarrhena pubescens*

*mai-hkwai*

*Streblus asper*

*mai-hok-hpa*

*Terminalia tomentosa*

*mai-hpa*

*Callicarpa macrophylla*

*mai-hpang*

*Ficus semicordata*

*mai-hpawng-tun*

*Mallotus philippensis*

*mai-ka-aung*

*Semecarpus anacardium*

*maikao*

*Butea monosperma*

*mai-kawk*

*Spondias pinnata*

*mai-keik*

*Salix tetrasperma*

*mai-kham*

*Garuga pinnata*

maikoa

*Brugmansia arborea*

*mai-kokkyi*

*Rhus chinensis*

*mai-kokkyin*

*Rhus chinensis*

*mai-kong-leng*

*Ricinus communis*

*mai-kyaing*

*Tamarindus indica*

*mai-kyang*

*Schleichera oleosa*

*mai-kye*

*Markhamia stipulata*

*mai-kying-lwai*

*Albizia odoratissima*

*mai-lang*

*Kopsia fruticosa*

*Wrightia arborea*

*mai-long-ka-hkam*

*Millingtonia hortensis*

*mai-lum*

*Cassia fistula*

*mai-lusang*

*Ficus semicordata*

*mai-mahen*

*Terminalia bellirica*

*mai-mak-hat*

*Artocarpus lakoocha*

*mai-mak-hkam*

*Phyllanthus emblica*

*mai-mak-kawk*

*Spondias pinnata*

*mai-mak-na*

*Terminalia chebula*

*mai-man-nah*

*Terminalia chebula*

*mai-mupi*

*Anneslea fragrans*

*mai-mye-sili*

*Senna siamea*

*mai-nam-lawt*

*Capparis zeylanica*

*mai-naw*

*Terminalia bellirica*

*mai-nio*

*Bombax ceiba*

*mai-nyawng*

*Ficus religiosa*

*mai-pinngo*

*Careya arborea*

*mai-put*

*Callicarpa macrophylla*

*mai-pyit*

*Mayodendron igneum*

*mai-sak*

*Tectona grandis*

*mai-sa-lan*

*Tectona grandis*

*mai-salan*

*Xylia xylocarpa*

*mai-sat-lang*

*Croton persimilis*

*mai-saw*

*Gmelina arborea*

*maisen*

*Dillenia indica*

*mai-song*

*Schima wallichii*

*mai-son-pu*

*Hymenodictyon orixense*

*mai-sung-hkong-long*

*Leea macrophylla*

*mai-tawn*

*Albizia odoratissima*

*maiting*

*Mesua ferrea*

*maiyang*

*Holarrhena pubescens*

*mai-yang-hka-oaun*

*Wrightia arborea*

*mai-yum*

*Toona sureni*

maize

*Zea mays*

*makalaw*

*Terminalia bellirica*

*mak-hkam-sang-paw*

*Phyllanthus acidus*

*makhkaw-hku*

*Ziziphus jujuba*

*mak-hpung*

*Averrhoa carambola*

*mak-k yeng*

*Tamarindus indica*

*mak-kawng-tawn*

*Bridelia retusa*

*mak-kok*

*Ziziphus rugosa*

*mak-kyen*

*Flacourtia jangomas*

*mak-lang*

*Artocarpus heterophyllus*

*mak-lok-kaing*

*Vitex glabrata*

*makman-yoo*

*Jatropha curcas*

*mak-mong*

*Mangifera indica*

*mak-mong-sang-yip*

*Anacardium occidentale*

*makpa nakeching*

*Callicarpa macrophylla*

*mak-phyn*

*Aegle marmelos*

*mak-pyen-sum*

*Limonia acidissima*

*mak-sang-hpaw*

*Carica papaya*

*mak-spye*

*Syzygium jambos*

*maksun-ting*

*Citrus aurantiifolia*

*mak-tasu-long*

*Leea macrophylla*

Malabar almond

*Terminalia catappa*

Malabar nut tree

*Justicia adhatoda*

*malaka*

*Psidium guajava*

*malame*

*Cardiospermum halicacabum*

Malay banyan

*Ficus retusa*

Malay bush-beech

*Gmelina arborea*

Malay laurel

*Ficus retusa*

*maleinka*

*Oroxylum indicum*

*ma-monton*

*Mangifera indica*

*mamung*

*Mangifera indica*

*mana*

*Terminalia chebula*

*manglon*

*Tamarindus indica*

mango

*Mangifera indica*

mangosteen

Garcinia × mangostana

*mango-taukpa-tit*

*Strychnos potatorum*

*mangrai*

*Salix tetrasperma*

mangrove

*Rhizophora mucronata*

*maniawga*

*Carallia brachiata*

manila tamarind

*Pithecellobium dulce*

*mani-thanl-yet*

*Capparis zeylanica*

*mankala*

*Psidium guajava*

*mansi*

*Carica papaya*

maranta

*Maranta arundinacea*

*margosa*

*Azadirachta indica*

marigold

*Tagetes erecta*

marihuana

*Cannabis sativa*

markingnut tree

*Semecarpus anacardium*

marsh herb

*Enydra fluctuans*

marsh parsley

*Apium graveolens*

marvel of Peru

*Mirabilis jalapa*

*masa*

*Schima wallichii*

*mashawt pin*

*Euonymus kachinensis*

mataran tea

*Senna auriculata*

*mat-lay*

*Ipomoea hederifolia*

*ma-tu-pin*

*Elephantopus scaber*

*ma-u*

*Nauclea orientalis*

*Neolamarckia cadamba*

*ma-u-gyi*

*Nauclea orientalis*

*ma-u-kadon*

*Nauclea orientalis*

*maula*

*Spatholobus parviflorus*

*ma-u-let-tan-she*

*Neolamarckia cadamba*

*maulu*

*Spatholobus parviflorus*

*mau-phyu*

*Neolamarckia cadamba*

*mawk*

*Magnolia champaca*

*mawk nang-nang*

*Combretum indicum*

*mawk-hkam-long*

*Cascabela thevetia*

*mawk-kham*

*Indigofera cassioides*

*mawk-manu*

*Hibiscus schizopetalus*

*mawkmnae*

*Hibiscus schizopetalus*

*mawk-nawn-hkam*

*Acacia farnesiana*

*mawk-sam-ka*

*Plumeria rubra*

*mawk-sam-pailong*

*Plumeria rubra*

*mawrite nawa*

*Piper nigrum*

*mawsanku*

*Santalum album*

*mayauklok-ni*

*Artocarpus lakoocha*

*mayo*

*Calotropis gigantea*

*mayoe*

*Calotropis procera*

*mayu-de*

*Markhamia stipulata*

*maza*

*Cinnamomum bejolghota*

*me-byaung*

*Memecylon edule*

*meegyaung-kun-hpat*

*Hygrophila phlomoides*

*meet*

*Curcuma longa*

*meik-kye*

*Albizia odoratissima*

*meik-mahot*

*Artocarpus lakoocha*

*meiksong*

*Schima wallichii*

*meik-tha-lin*

*Zingiber montanum*

*meiktun*

*Anneslea fragrans*

*mejari*

*Senna siamea*

melastoma

*Melastoma malabathricum*

*men-khareek-leck-chuck*

*Aloe vera*

*merokwa*

*Terminalia tomentosa*

metal seed

*Senna tora*

Mexican lime

*Citrus aurantiifolia*

Mexican petunia

*Strobilanthes auriculatus*

Mexican prickly poppy

*Argemone mexicana*

Mexican tea

*Dysphania ambrosioides*

*me-yaing*

*Tephrosia purpurea*

*mezali*

*Senna siamea*

*mezali-gyi*

*Senna alata*

*miat*

*Memecylon edule*

michelia

*Magnolia champaca*

*mickyat*

*Kydia calycina*

microcos

*Grewia nervosa*

*mi-gwin-gamone*

*Tradescantia spathacea*

*migyaung-kunbat*

*Hygrophila phlomoides*

*Linum usitatissimum*

*mi-gyaung-nwe*

*Millettia pachycarpa*

milk weed

*Euphorbia hirta*

milkhedge

*Euphorbia antiquorum*

millet

*Cymbopogon jwarancusa*

millettia

*Millettia pachycarpa*

mimosa

*Mimosa pudica*

*mi-nauk*

*Memecylon edule*

*minbaw*

*Caryota mitis*

*mingut*

Garcinia × mangostana

mistletoe

*Viscum cruciatum*

moi

*Lannea coromandelica*

*moko-lanma*

*Tadehagi triquetrum*

*momakha*

*Salix tetrasperma*

*mondaing*

*Cycas rumphii*

monia

*Lannea coromandelica*

monkey nut

*Arachis hypogaea*

monkey-jack

*Artocarpus lakoocha*

*mon-la-ni*

*Daucus carota*

moon flower

*Ipomoea alba*

moonbeam

*Tabernaemontana divaricate*

moonflower

*Datura stramonium*

*moot maiboa*

*Cardiospermum halicacabum*

morinda

*Morinda angustifolia*

morning glory

*Convolvulus arvensis*

Moses in a cradle

*Tradescantia spathacea*

moulmein cedar

*Toona sureni*

mountain ebony

*Diospyros malabarica*

Mousa nettle

*Urtica parviflora*

*mung-dung*

*Artocarpus heterophyllus*

*mung-ting*

*Acacia catechu*

munjeet

*Rubia cordifolia*

musk mallow

*Abelmoschus moschatus*

mussaenda

*Mussaenda macrophylla*

mussoorie berry

*Coriaria nepalensis*

*mway-ma-naing*

*Abroma augustum*

*mway-say*

*Abroma augustum*

*mway-seik-phay-pin*

*Abroma augustum*

*mwet-kang*

*Symplocos racemosa*

*myat-y*a

*Grewia nervosa*

*myauk-laung*

*Artocarpus lakoocha*

*myauk-le-sik*

*Aglaia cucullata*

*myauk-lok*

*Artocarpus lakoocha*

*myauk-seik*

*Holoptelea integrifolia*

*myauk-zi*

*Ziziphus rugosa*

*mya-yar*

*Grewia nervosa*

*myay-byit*

*Portulaca oleracea*

*myay-pe*

*Arachis hypogaea*

*myay-pe-naw-nam*

*Senna tora*

*mye-mu-se*

*Cratoxylum formosum*

*myet-hmwe*

*Cymbopogon nardus*

*myet-hna-pan*

*Pavetta indica*

*myet-htauk*

*Portulaca oleracea*

*myet-lay-gwa*

*Dactyloctenium aegyptium*

*myet-na-myin-gyin*

*Pavetta indica*

*myetpye*

*Melastoma malabathricum*

*myet-sek*

Equisetum ramosissimum subsp. debile

*myinga*

*Cynometra ramiflora*

*myin-gaung-nayaung*

*Celastrus paniculatus*

*myin-gondaing*

*Celastrus paniculatus*

*myin-hkwa*

*Centella asiatica*

*myin-kahpan*

*Grewia nervosa*

*myin-khwar pin*

*Centella asiatica*

*myin-lauk-yaung*

*Celastrus paniculatus*

*myitzu pan pin*

*Mirabilis jalapa*

myrobalan

*Phyllanthus emblica*

*Terminalia bellirica*

*Terminalia chebula*

*myu*

*Chenopodium album*

*my-yar-gyi*

*Justicia adhatoda*

*nabe*

*Lannea coromandelica*

*nabu-nwe*

*Vallaris solanacea*

*nadaung-ban*

Lantana × aculeata

*nagbala*

*Sida spinosa*

nagi camphor

*Blumea balsamifera*

*nakzik*

*Cinnamomum bejolghota*

*na-lin-gyaw*

*Cinnamomum bejolghota*

*nam ya-hai-awn*

*Mimosa pudica*

*namchying*

*Curcuma longa*

nana cane

*Arundo donax*

*nanat-gyi*

*Agave sisalana*

*na-nat-shaw*

*Agave sisalana*

*nang-mu*

*Combretum indicum*

*nan-lon-kyaing*

*Acacia farnesiana*

*nannan*

*Coriandrum sativum*

*nantayok*

*Altingia excelsa*

*nanttha hpyu*

*Santalum album*

*nanwin*

*Curcuma longa*

*nanwinga*

*Curcuma comosa*

natal plum

*Carissa spinarum*

*natha hpyu*

*Santalum album*

*naukpo*

*Achyranthes aspera*

*nawnam*

*Senna italica*

*naywe*

*Flacourtia jangomas*

*nbau*

*Alnus nepalensis*

*ndung*

*Artocarpus heterophyllus*

needle wood

*Schima wallichii*

*neem*

*Azadirachta indica*

neepa bark

*Quassia indica*

*nee-par hsay-pin*

*Morinda coreia*

*nehle*

*Cullen corylifolium*

*new-ni*

*Celastrus paniculatus*

*new-nyo*

*Thunbergia laurifolia*

*nga-be*

*Abroma augustum*

*nga-chat-wa*

*Bambusa bambos*

*ngal-hjyang*

*Anneslea fragrans*

ngapi nut

*Archidendron jiringa*

*ngasee*

*Glycine max*

*ngayan-padu*

*Rotheca incisa*

*ngayant patu*

*Clerodendrum indicum*

*ngayok*

*Capsicum annuum*

*ngayoke kha*

*Andrographis paniculata*

*ngayoke-kaung*

*Piper nigrum*

*nga-yok-kaung*

*Piper longum*

*ngu*

*Cassia fistula*

*ngu pin*

*Cassia fistula*

*ngusat*

*Senna tora*

*ngu-shwe*

*Cassia fistula*

*ngushwe-ama*

*Cassia fistula*

*nhtau-ru*

*Millettia pachycarpa*

*nibase*

*Morinda citrifolia*

nicandra

*Nicandra physalodes*

night jasmine

*Nyctanthes arbor-tristis*

*ning-bau*

*Alnus nepalensis*

*nle-prangkau*

*Symplocos racemosa*

*nlung*

*Morinda angustifolia*

nodding smartweed

*Persicaria pulchra*

*noni*

*Morinda citrifolia*

notch-seeded buckwheat

*Fagopyrum esculentum*

nutgall tree

*Rhus chinensis*

nutmeg

*Myristica fragrans*

nutmeg flower

*Nigella sativa*

*nu-wah*

*Gossypium barbadense*

*nwai-pe*

*Lablab purpureus*

*nwamni-than-lyet*

*Capparis zeylanica*

nwar myay yinn

*Cyperus scariosus*

*nwar-mee-kat*

*Urena lobata*

*nwei thargi*

*Nerium oleander*

*nwe-kazun-phyu*

*Ipomoea alba*

*nwe-nathan-gwin*

*Hiptage benghalensis*

*nwe-ni*

*Spatholobus parviflorus*

*nya*

*Acacia catechu*

*nyagyi*

*Morinda citrifolia*

*nyaung bokdahae*

*Ficus religiosa*

*nyaung-bawdi*

*Ficus religiosa*

*nyaung-lun*

*Ficus benjamina*

*nyaung-ok*

*Ficus retusa*

*nyaung-phyu*

*Ficus rumphii*

*nyaung-thabye*

*Ficus benjamina*

*nyaung-ye-o-pan*

*Melastoma malabathricum*

*nygayan-padu*

*Clerodendrum indicum*

*o-dein*

*Kleinhovia hospita*

oilgrass

*Cymbopogon jwarancusa*

*okhne*

*Streblus asper*

*okshit*

*Aegle marmelos*

olax

*Olax scandens*

oleander

*Nerium oleander*

oleander-leaved euphorbia

*Euphorbia neriifolia*

*on-hnye*

*Aerva javanica*

onion

*Allium cepa*

opposite-leaf drysophylia

*Colebrookea oppositifolia*

ortie

*Urtica dioica*

Otaheite gooseberry

*Phyllanthus acidus*

oval-leaf monochoria

*Monochoria vaginalis*

oval-leaf pondweed

*Monochoria vaginalis*

oyster plant

*Tradescantia spathacea*

Pacific maple

*Aglaia cucullata*

*padaing*

*Brugmansia suaveolens*

*Datura metel*

*pa-daing-byu*

*Datura metel*

*pa-daing-khata*

*Datura metel*

*padaing-khat-ta*

*Datura stramonium*

*pa-daing-ni*

*Datura metel*

*padaing-nyo*

*Datura stramonium*

*padat-nygan*

*Artabotrys hexapetalus*

*padegaw-gale*

*Alpinia officinarum*

*padei-kaw gyi*

*Alpinia galanga*

*padei-kaw lay*

*Alpinia officinarum*

*pa-deing-ngo*

*Exacum tetragonum*

padri

*Stereospermum chelonoides*

pagoda tree

*Plumeria rubra*

*pahi*

*Tectona grandis*

*paiksan*

*Linum usitatissimum*

*palan*

*Bauhinia acuminata*

*palannwe*

*Mallotus philippensis*

*palan-taunghmwe*

*Cheilocostus speciosus*

pale smartweed

*Persicaria pulchra*

*pale-ban*

*Sambucus javanica*

*panameikli*

*Vitex glabrata*

*panga*

*Terminalia chebula*

panicled peristrophe

*Peristrophe bicalyculata*

*pan-le*

*Woodfordia fruticosa*

*pan-letwa*

*Phyllodium pulchellum*

*pan-ma*

*Anneslea fragrans*

*Schima wallichii*

*pa-noh*

*Artocarpus heterophyllus*

*panswe*

*Woodfordia fruticosa*

*pan-swe-le*

*Hibiscus schizopetalus*

*pan-thawka*

*Ixora coccinea*

*panwe*

*Artocarpus heterophyllus*

*pan-ye-sut-nwe*

*Thunbergia laurifolia*

*pan-zayeik*

*Ixora coccinea*

papaw

*Carica papaya*

papaya

*Carica papaya*

*pashu-phet-wun*

*Kleinhovia hospita*

pasture brake

*Pteridium aquilinum*

patana oak

*Careya arborea*

patchouli

*Pogostemon cablin*

*pattagyi*

*Woodfordia fruticosa*

*pauk-kyn*

*Markhamia stipulata*

*pauk-new*

*Butea superba*

*pauk-nwe*

*Spatholobus parviflorus*

*pauk-pan-byu*

*Sesbania grandiflora*

*paukpin*

*Butea monosperma*

*paung*

*Terminalia tomentosa*

*paung-thaung*

*Strobilanthes auriculatus*

*pawpan*

*Butea monosperma*

pawpaw

*Carica papaya*

*paw-tohkaw*

*Butea superba*

*pa-yan-na-war*

*Boerhavia diffusa*

*Commicarpus chinensis*

*payaung-pan*

*Cascabela thevetia*

*payoke-aye*

*Mentha arvensis*

*payoke-pin*

*Cinnamomum camphora*

*payon-ama*

*Rhizophora mucronata*

*payuk*

*Cinnamomum camphora*

*pazun-sar*

*Alternanthera sessilis*

*pazun-za*

*Alternanthera sessilis*

peanut

*Arachis hypogaea*

*pebok*

*Callicarpa macrophylla*

*pe-bok*

*Glycine max*

*pe-bok-new*

*Paederia foetida*

*pe-dalet*

*Canavalia ensiformis*

*pe-dama*

*Canavalia ensiformis*

*peepthong*

*Mayodendron igneum*

*peik-chin*

*Piper longum*

*peik-thingat*

*Senna auriculata*

*pein*

*Colocasia antiquorum*

*pein-gya*

*Pothos scandens*

*peinne*

*Artocarpus heterophyllus*

*pein-u*

*Colocasia antiquorum*

*pe-nauk-ni*

*Clitoria ternatea*

*pe-ngapi*

*Glycine max*

periwinkle

*Catharanthus roseus*

Peruvian winter cherry

*Physalis peruviana*

Peruvian yellow oleander

*Cascabela thevetia*

petari

*Mallotus nudiflorus*

*petya*

*Girardinia diversifolia*

*petya-gyi*

*Girardinia diversifolia*

phalsa

*Grewia asiatica*

*phan-kha*

*Terminalia chebula*

*phat-kyi*

*Coriandrum sativum*

*phat-swon-pan*

*Hibiscus sabdariffa*

*phet-wun-ni*

*Kydia calycina*

*phon-ma-thein*

*Blumea balsamifera*

physic nut

*Jatropha curcas*

physic nut

*Jatropha gossypiifolia*

physic nut

*Jatropha multifida*

pickerel weed

*Monochoria vaginalis*

pigweed

*Amaranthus spinosus*

*Chenopodium album*

*pi-khum*

*Gouania leptostachya*

*pin-gu-hteik-peik*

*Leucas cephalotes*

*pinle-kabwe*

*Casuarina equisetifolia*

*pinle-kazun*

*Ipomoea pes-caprae*

*pinle-kyauk-pan*

*Volkameria inermis*

*pinle-ohn*

*Xylocarpus moluccensis*

*pinle-on*

*Xylocarpus moluccensis*

*pinle-tinyu*

*Casuarina equisetifolia*

*pin-sein hmway*

*Ocimum americanum*

*pin-sein*

*Ocimum americanum*

*pin-sein-net*

*Ocimum tenuiflorum*

*pkhay*

*Xylia xylocarpa*

plantain

*Plantago major*

*po-gaungsa*

*Bischofia javanica*

poison bulb

*Crinum asiaticum*

poma

*Lannea coromandelica*

pomegranate

*Punica granatum*

*ponenyet*

*Calophyllum inophyllum*

*pon-na-yeik*

*Ixora chinensis*

*Ixora coccinea*

*ponnayeik*

*Pavetta indica*

*pon-nyet*

*Anneslea fragrans*

*popee*

*Urena lobata*

*pora-mat*

*Benincasa hispida*

porcupine flower

*Barleria prionitis*

pot

*Cannabis sativa*

potato yam

*Dioscorea bulbifera*

*po-thi-din*

*Mallotus philippensis*

pothos

*Pothos scandens*

*praing*

*Xylia xylocarpa*

prairie turnip

*Cullen corylifolium*

*pran*

*Xylia xylocarpa*

*prang-gadawn*

*Rotheca serrata*

prickly chaff

*Achyranthes aspera*

prickly fanpetals

*Sida spinosa*

prickly poppy

*Argemone mexicana*

pride of Barbados

*Caesalpinia pulcherrima*

pride of Burma

*Amherstia nobilis*

prince’s feather

*Amaranthus cruentus*

*prung*

*Nauclea orientalis*

*Neolamarckia cadamba*

*prway*

*Xylia xylocarpa*

*pu*

*Piper betle*

pudding pipe tree

*Cassia fistula*

puneala plum

*Flacourtia jangomas*

purging cassia

*Cassia fistula*

purging nut

*Jatropha curcas*

purple allamanda

*Thunbergia laurifolia*

purple amaranthus

*Amaranthus cruentus*

purple foxglove

*Digitalis purpurea*

purple-flowered resurection lily

*Kaempferia elegans*

purslane

*Portulaca oleracea*

pursley

*Portulaca oleracea*

*pusi-nan*

*Mentha arvensis*

*putsa-u*

*Dioscorea bulbifera*

*put-sa-u*

*Dioscorea pentaphylla*

puzzlenut tree

*Xylocarpus moluccensis*

*pwe-gaing*

*Senna alexandrina*

*pwekonclaw*

*Mucuna pruriens*

*pwesay-mezali*

*Senna alata*

*pwint-tu-ywet-tu*

*Mussaenda macrophylla*

*pya*

*Symplocos racemosa*

*pyauk-seik*

*Holoptelea integrifolia*

*pyaung-bu*

*Zea mays*

*pyiban-nyo*

*Senna sulfurea*

*pyidban-shwe*

*Senna sulfurea*

*pyin*

*Xylia xylocarpa*

*pyin-daw-thein*

*Clausena excavata*

*pyinkado*

*Xylia xylocarpa*

*pyinma-ywetthey*

*Lagerstroemia speciosa*

pyrethrum

*Tanacetum cinerariifolium*

*pyu*

*Rhizophora mucronata*

quassia wood

*Picrasma javanica*

Queen Anne’s lace

*Daucus carota*

queen of flowering trees

*Amherstia nobilis*

queen’s crape myrtle

*Lagerstroemia speciosa*

Queensland asthma herb

*Euphorbia hirta*

Queesland arrowroot

*Canna indica*

*ra*

*Mucuna pruriens*

rabbit greens

*Ipomoea aquatica*

*rai baitine*

*Sinapis alba*

*rai jamun*

*Syzygium nervosum*

ramie

*Boehmeria nivea*

Rangoon creeper

*Combretum indicum*

*Quassia indica*

*ranjneh hnah*

*Centella asiatica*

red amaranthus

*Amaranthus cruentus*

red bean vine

*Abrus precatorius*

red cedar

*Toona sureni*

red cottontree

*Bombax ceiba*

red milkweed

*Asclepias curassavica*

red morning-glory

*Ipomoea hederifolia*

red pepper

*Capsicum annuum*

red plumeria

*Plumeria rubra*

red sandalwood

*Adenanthera pavonina*

red santol

*Sandoricum koetjape*

red sarsaparilla

*Ichnocarpus frutescens*

red silk-cotton

*Bombax ceiba*

red tasselflower

*Emilia sonchifolia*

red-fruit passionflower

*Passiflora foetida*

red-root

*Cannabis sativa*

resurection lily

*Kaempferia elegans*

ribwort

*Plantago major*

rimil-beer

*Olax scandens*

ringworm cassia

*Senna alata*

ringworm shrub

*Senna alata*

rohituka

*Aphanamixis polystachya*

Roman coriander

*Nigella sativa*

rosary pea

*Abrus precatorius*

rose apple

*Syzygium jambos*

rose of China

*Hibiscus schizopetalus*

rose of Sharon

*Nerium oleander*

rosebay

*Holarrhena pubescens*

rosy leadwort

*Plumbago indica*

round zedoary

*Curcuma zedoaria*

royal poinciana

*Delonix regia*

rozelle

*Hibiscus sabdariffa*

*rubanru*

*Spatholobus parviflorus*

rubber tree

*Schefflera venulosa*

Rumphf’s fig tree

*Ficus rumphii*

running-pop

*Passiflora foetida*

*sabalin-hmwe*

*Cymbopogon nardus*

*sabe-hmwe-sok*

*Jasminum multiflorum*

sacred basil

*Ocimum tenuiflorum*

sacred fig tree

*Ficus religiosa*

sacred garlic pear

*Crateva religiosa*

safflower

*Carthamus tinctorius*

*sagale-amauk*

*Premna amplectens*

*saga-sein*

*Cananga odorata*

sage

*Salvia officinalis*

*sagyaw*

*Mangifera indica*

*sahkao*

*Melastoma malabathricum*

*saingnan*

*Strobilanthes auriculatus*

*saka-wah*

*Magnolia champaca*

*sakina*

*Indigofera cassioides*

*sam lung*

*Magnolia champaca*

*sameik*

*Anethum graveolens*

*sammankaw*

*Ziziphus rugosa*

*samon nyo*

*Anethum graveolens*

*samone hpyu*

*Trachyspermum ammi*

*samon-net*

*Nigella sativa*

*samon-nwe*

*Momordica cochinchinensis*

*samon-saba*

*Foeniculum vulgare*

*samon-sabar*

*Foeniculum vulgare*

*samut*

*Apium graveolens*

*sandakoo*

*Santalum album*

sandalwood

*Santalum album*

*sangawn-gmawt*

*Careya arborea*

*sang-hpaw*

*Carica papaya*

*sani kamat*

*Asparagus officinalis*

*san-phak*

*Limonia acidissima*

*sansph-ka*

*Limonia acidissima*

*santagu*

*Santalum album*

*santal*

*Sandoricum koetjape*

santol

*Sandoricum koetjape*

*sanut-khar*

*Limonia acidissima*

*sa-nwin*

*Curcuma longa*

*Curcuma zedoaria*

*sanwinga*

*Curcuma comosa*

*sanwin-yaing*

*Curcuma comosa*

*sapalin*

*Cymbopogon citratus*

sapistan

*Cordia myxa*

sapodilla

*Manilkara zapota*

sariva

*Ichnocarpus frutescens*

sarsaparilla

*Ichnocarpus frutescens*

*sa-thange-ohnauk*

*Cratoxylum formosum*

*saung-chan*

*Capparis flavicans*

*saung-daw-ku*

*Verbena officinalis*

*saungkyan*

*Capparis flavicans*

savannah fern

*Dicranopteris linearis*

*saydan-kya*

*Acalypha wilkesiana*

*say-my*

*Dysphania ambrosioides*

*sayo pin*

*Piper cubeba*

scarlet ixora

*Ixora coccinea*

scarlet leadwort

*Plumbago indica*

scarlet wisteria tree

*Sesbania grandiflora*

schima

*Schima wallichii*

scuffy pea

*Cullen corylifolium*

sea holly

*Acanthus ilicifolius*

*Eryngium caeruleum*

sea island cotton

*Gossypium barbadense*

Sebastian tree

*Cordia dichotoma*

*se-baung-gyan*

*Pavetta indica*

Sebesten plum

*Cordia myxa*

*sega-gyi*

*Andrographis paniculata*

*se-gyauk*

*Cannabis sativa*

*seik-chi*

*Bridelia retusa*

*seikchi-bo*

*Bridelia retusa*

*seik-nan*

*Clausena excavata*

*seiknan-gyi*

*Premna mollissima*

*seik-palu*

*Nyctanthes arbor-tristis*

*seinban*

*Delonix regia*

*seinnaban*

Lantana × aculeata

*sein-pan-gale*

*Caesalpinia pulcherrima*

*sein-takyu*

*Tecoma stans*

*se-kalon*

*Martynia annua*

*se-khar-gyi*

*Andrographis paniculata*

*sekku-pan*

*Bougainvillea spectabilis*

*se-laik-pya*

*Flemingia chappar*

*se-laik-pya*

*Flemingia strobilifera*

*se-leik-pya*

*Phyllodium pulchellum*

selu

*Cordia myxa*

*semakhan*

*Jatropha multifida*

*semein*

*Millettia pachycarpa*

sensitive plant

*Mimosa pudica*

sentol

*Sandoricum koetjape*

sentul

*Sandoricum koetjape*

serpent wood

*Rauvolfia serpentina*

sesame

*Sesamum indicum*

sessile joyweed

*Alternanthera sessilis*

*sethnayathi*

*Cascabela thevetia*

*set-hnit-ya-thi*

*Cascabela thevetia*

*setkadon*

*Mallotus nudiflorus*

*set-kalwe*

*Phyllanthus emblica*

*set-thalwe*

*Phyllanthus emblica*

Seville orange

Citrus × aurantium

*sha*

*Acacia catechu*

*shabyu*

*Phyllanthus emblica*

*shadwe*

*Ardisia humilis*

*shagan changgan*

*Butea monosperma*

shaggy button weed

*Spermacoce hispida*

*shagyaw*

*Mangifera indica*

*shaji*

*Acacia catechu*

*shakau*

*Allium cepa*

shame weed

*Mimosa pudica*

*shame*

*Melastoma malabathricum*

shan camphor

*Blumea balsamifera*

*shang hap-wsi*

*Carica papaya*

*shanghpaw*

*Carica papaya*

*shan-kazaw*

*Senna italica*

*shapawing*

*Ricinus communis*

*shari-mam*

*Fagopyrum esculentum*

*shauk*

*Citrus limon*

*shauk-waing*

*Citrus limon*

*sha-zaung-let-pat*

*Aloe vera*

*shazaung-myin-na*

*Euphorbia neriifolia*

*shewewa-pan*

*Allamanda cathartica*

*shinasoo*

*Amorphophallus paeoniifolius*

*shitkale*

*Anacardium occidentale*

shoe flower

*Hibiscus schizopetalus*

shoo-fly plant

*Nicandra physalodes*

shrub-vinca

*Kopsia fruticosa*

*shwedagon*

*Asclepias curassavica*

*shwe-gu-than-hlet*

*Tadehagi triquetrum*

*shwe-new*

*Cuscuta reflexa*

*shwe-nwe-pin*

*Cuscuta reflexa*

*shwe-pan-new*

*Allamanda cathartica*

Siamese cassia

*Senna siamea*

Siamese rough bush

*Streblus asper*

siamweed

*Chromolaena odorata*

*si-cho*

*Orthosiphon aristatus*

sicklepod

*Senna tora*

sigesbeckia

*Sigesbeckia orientalis*

*sigyi*

*Callicarpa macrophylla*

silk-cottontree

*Bombax ceiba*

*Ceiba pentandra*

silky wormwood

*Artemisia dracunculus*

silver cock’s comb

*Celosia argentea*

simal

*Bombax ceiba*

*sin-che*

*Elephantopus scaber*

*sindon-ma-nwe*

*Tinospora cordifolia*

*sin-gwe*

*Stereospermum colais*

*sin-hna-maung*

*Heliotropium indicum*

*sin-kayan*

*Solanum melongena*

*sin-let-maung*

*Heliotropium indicum*

*sinna*

*Pterospermum acerifolium*

*sin-petya*

*Girardinia diversifolia*

*sinyok*

*Garuga pinnata*

sisal

*Agave sisalana*

sisal hemp

*Agave sisalana*

*siyo-kyetsu*

*Jatropha curcas*

skunk vine

*Paederia foetida*

slender dwarf morning-glory

*Evolvulus alsinoides*

slow match tree

*Careya arborea*

small fennel

*Nigella sativa*

small-leaved rubber plant

*Ficus benjamina*

smartweed

*Persicaria pulchra*

smooth chastetree

*Vitex glabrata*

smooth loofah

*Luffa cylindrica*

smooth senna

*Senna sulfurea*

snow bush

*Aerva javanica*

soap pod

*Acacia concinna*

soapberry

*Sapindus saponaria*

soapnut

*Sapindus saponaria*

soft elephant’s-foot

*Elephantopus scaber*

soft hemp

*Cannabis sativa*

soja bean

*Glycine max*

*sok*

*Senna alata*

soldier-weed

*Amaranthus spinosus*

*sonpabataing*

*Plumeria rubra*

sorrel

*Hibiscus sabdariffa*

sorrowless tree

*Saraca indica*

*sort-htmaine*

*Moringa oleifera*

*sot lapoot*

*Acacia concinna*

*sot-cawee-katun*

*Momordica charantia*

*sot-crin*

*Syzygium jambos*

*sot-gren-itg*

*Oroxylum indicum*

*sot-keen*

*Mimusops elengi*

*sot-maroat*

*Annona squamosa*

*sot-parite-sanut*

*Citrus aurantiifolia*

*sot-talwe*

*Phyllanthus emblica*

sour orange

Citrus × aurantium

southernblue gum

*Eucalyptus globulus*

soy bean

*Glycine max*

soya bean

*Glycine max*

Spanish cane

*Arundo donax*

Spanish cherry

*Mimusops elengi*

speedwell

*Evolvulus alsinoides*

spiderflower

*Cleome gynandra*

spiderwisp

*Cleome gynandra*

spiny amaranthus

*Amaranthus cruentus*

*Amaranthus spinosus*

spiny bamboo

*Bambusa bambos*

spiny bitter-cucumber

*Momordica cochinchinensis*

spiny bittergourd

*Momordica cochinchinensis*

spiny pigweed

*Amaranthus spinosus*

sponge gourd

*Luffa cylindrica*

sponge-tree

*Acacia farnesiana*

spreading hogweed

*Boerhavia diffusa*

*Commicarpus chinensis*

star flower tree

*Mimusops elengi*

star gooseberry

*Phyllanthus acidus*

star ipomoea

*Ipomoea hederifolia*

starleaf

*Schefflera venulosa*

stinging nettle

*Urtica dioica*

stink vine

*Paederia foetida*

stinkwort

*Datura stramonium*

stinky opal berry

*Paederia foetida*

stramonium

*Datura stramonium*

strong-scented pigweed

*Dysphania ambrosioides*

*suboke-gyi*

*Acacia pennata*

*subyu*

*Acacia nilotica*

sugar apple

*Annona squamosa*

*suka*

*Passiflora foetida*

*sum-bawng*

*Hibiscus sabdariffa*

*sum-hkawn*

*Acacia concinna*

*sumhtung*

*Mayodendron igneum*

*sumtungh-kyeng*

*Mayodendron igneum*

*sum-tung-hpraw*

*Millingtonia hortensis*

suntwood

*Acacia nilotica*

*su-la-na-pha*

*Oldenlandia corymbosa*

*su-lar-na-phar*

*Oldenlandia corymbosa*

*su-padang*

*Hygrophila auriculata*

superb lily

*Gloriosa superba*

surfacea

*Premna amplectens*

*suyit*

*Acacia pennata*

swallow-wart

*Calotropis procera*

swamp daisy

*Eclipta prostrata*

swamp morning-glory

*Ipomoea aquatica*

*swe-daw*

*Bauhinia acuminata*

*Bauhinia purpurea*

*swedaw-ni*

*Bauhinia purpurea*

sweet acacia

*Acacia farnesiana*

sweet flag

*Acorus calamus*

sweetleaf

*Symplocos racemosa*

sweet-scented broom

*Scoparia dulcis*

sweetsop

*Annona squamosa*

switch cane

*Arundo donax*

sword bean

*Canavalia ensiformis*

*Entada phaseoloides*

*taagat-ta-gyi*

*Heynea trijuga*

tabasco

*Capsicum annuum*

*tabo*

*Kydia calycina*

*tabu*

*Zanthoxylum acanthopodium*

*tabyetse*

*Phyllodium pulchellum*

*tadaing-hmwe*

*Artabotrys hexapetalus*

*taesap*

*Garuga pinnata*

*ta-gat-net*

*Aphanamixis polystachya*

*taik-pan-gyi*bag-flower

*Clerodendrum thomsoniae*

*takau*

*Mangifera indica*

*tama*

*Azadirachta indica*

*tamaga*

*Azadirachta indica*

tamarind

*Tamarindus indica*

*tame*

*Syzygium cumini*

*Syzygium jambos*

*tamibaw*

*Caryota mitis*

tamilnadia

*Tamilnadia uliginosa*

*tanah toung*

*Ricinus communis*

*tanah-con-kamor*

*Plumbago zeylanica*

*tanah-pacow-kawaing angine*

*Combretum indicum*

*tanaung*

*Acacia leucophloea*

*ta-ner-hgaw*

*Coriandrum sativum*

Tanner’s cassia

*Senna auriculata*

Tanner’s tea

*Senna auriculata*

*tanom khapore*

*Butea monosperma*

*tanwhite*

*Piper longum*

*tanyin*

*Archidendron jiringa*

taro

*Colocasia antiquorum*

tarragon

*Artemisia dracunculus*

*tasha*

*Phyllanthus emblica*

Tasmanian blue gum

*Eucalyptus globulus*

tatea

*Premna amplectens*

*taukkyan*

*Terminalia tomentosa*

*tauk-kyant*

*Terminalia tomentosa*

*tauksha*

*Vitex glabrata*

*tauku-ywe*

*Cinnamomum bejolghota*

*taung-damin*

*Phyllodium pulchellum*

*taung-gnaw*

*Anneslea fragrans*

*taung-gwe*

*Lannea coromandelica*

*taung-kamaka*

*Picrasma javanica*

*taung-kyibaung*

*Viscum cruciatum*

*taung-magyi*

*Albizia odoratissima*

*taung-mayo*

*Alstonia scholaris*

*taung-meok*

*Alstonia scholaris*

*taung-petwun*

*Pterospermum acerifolium*

taung-sun

*M*aranta arundinacea

*taung-tama*

*Toona sureni*

*taung-tangyi*

*Premna serratifolia*

*taung-thanut*

*Cordia myxa*

*taung-thayet*

*Buchanania lancifolia*

*taung-zalut*

*Wrightia arborea*

*taw-bizat*

*Chromolaena odorata*

*tawbut*

*Luffa cylindrica*

*taw-hingala*

*Cleome gynandra*

*tawitho*

*Terminalia bellirica*

*taw-kalamet*

*Pterospermum acerifolium*

*taw-kanako*

*Jatropha gossypiifolia*

*taw-khan-pin*

*Carissa spinarum*

*taw-kinmon*

*Coccinia grandis*

*taw-ma-hnyo-lon*

*Grangea maderaspatana*

*taw-mevaing*

*Indigofera cassioides*

*taw-mezali*

*Senna siamea*

*tawngto-nao*

*Callicarpa macrophylla*

*taw-pilaw*

*Malvastrum coromandelianum*

*taw-sabe*

*Ichnocarpus frutescens*

*tawsabe*

*Jasminum multiflorum*

*taw-suka-ban*

*Passiflora foetida*

*taw-thabut*

*Momordica cochinchinensis*

*taw-thi-din*

*Mallotus philippensis*

*taw-wah*

*Abelmoschus moschatus*

*taw-yinma*

*Chukrasia tabularis*

*taw-zalat*

*Tabernaemontana divaricate*

*taw-zi*

*Ziziphus rugosa*

*taya*

*Phyllanthus emblica*

*tayaw*

*Grewia hirsuta*

*tayaw-ni*

*Kydia calycina*

*tayaw-nyo-nye*

*Gouania leptostachya*

*tayokenan-nan*

*Apium graveolens*

*tayoksaga-ani tayok-saga*

*Plumeria rubra*

*tayok-the*

*Bischofia javanica*

*tayok-zi*

*Litchi chinensis*

*ta-zaung*

*Euphorbia neriifolia*

*tazaung-gyi*

*Euphorbia antiquorum*

*tazaung-pyathat*

*Euphorbia antiquorum*

*te*

*Diospyros mollis*

teak

*Tectona grandis*

tellicherry bark

*Holarrhena pubescens*

*Terminalia chebula*

*thabye-kyet-chi*

*Syzygium cumini*

*thabye-phyu*

*Syzygium cumini*

*thabye-shin*

*Syzygium nervosum*

*thabyetsi-bin*

*Sida spinosa*

*thabyu*

*Dillenia indica*

*thabyu-thaby*

*Syzygium jambos*

*thabyu-thabye*

*Syzygium jambos*

*tha-dut*

*Ficus semicordata*

*thagya*

*Manilkara zapota*

*thagya-bin*

*Scoparia dulcis*

*thagya-hlandin*

*Tadehagi triquetrum*

*thagyar makike*

*Orthosiphon aristatus*

*thakabti*

*Schleichera oleosa*

*tha-khwar-thi*

*Cucumis sativus*

*thakut-pho*

*Stereospermum colais*

*thakut-po*

*Stereospermum colais*

*thale*

*Punica granatum*

*tha-min-sa-hpru-thi*

*Catunaregam spinosa*

*tha-myet*

*Momordica cochinchinensis*

*thanakha*

*Limonia acidissima*

*thanat*

*Cordia dichotoma*

*Cordia myxa*

*thanat-kha*

*Chloranthus elatior*

*thanat-pyit-see*

*Pogostemon cablin*

*thanbayar*

*Citrus aurantiifolia*

*than-bayo*

*Citrus limon*

*thande*

*Stereospermum colais*

*thankaungh*

*Terminalia chebula*

*than-manaing-kyauk-manaing*

*Alysicarpus vaginalis*

*than-tat*

*Stereospermum colais*

*than-tay*

*Stereospermum colais*

*than-that-gyi*

*Aphanamixis polystachya*

*thanut*

*Cordia dichotoma*

*thauk-kya*

*Vitex glabrata*

*thawka*

*Amherstia nobilis*

*Saraca indica*

*thawka-gyi*

*Amherstia nobilis*

*thawka-po*

*Saraca indica*

*thaya-muli*

*Aristolochia indica*

*thayet*

*Mangifera indica*

*thayet-phyu*

*Mangifera indica*

*thayet-thin-baung*

*Buchanania lancifolia*

*theban*

*Quassia indica*

*thebla*

*Gmelina arborea*

*thelaw*

*Careya arborea*

*thetyin-gyi*

*Croton persimilis*

*thi*

*Limonia acidissima*

*thiag-riang*

*Terminalia bellirica*

*thi-ha-yaza*

*Limonia acidissima*

*thiho-thayet*

*Anacardium occidentale*

*thinbauk-nanat*

*Agave sisalana*

*thinbaw*

*Carica papaya*

*thinbaw kyet-hsu*

*Ricinus communis*

*thinbaw-adalut*

*Maranta arundinacea*

*thinbaw-kanako*

*Jatropha gossypiifolia*

*thinbaw-kyetsu*

*Jatropha curcas*

*thin-baw-kyetsy*

*Jatropha curcas*

*thinbaw-letpan*

*Ceiba pentandra*

*thinbaw-ma-hnyoe*

*Catharanthus roseus*

*thinbaw-ma-hnyo-pan*

*Catharanthus roseus*

*thinbaw-ma-hnyo-pan-aphyu*

*Catharanthus roseus*

*thinbaw-mezali*

*Senna alata*

*Senna alexandrina*

*thin-baw-na-nat*

*Agave vera-cruz*

*thinbaw-te*

*Polyalthia longifolia*

*thinbaw-tidin*

*Bixa orellana*

*thinbaw-zalut*

*Kopsia fruticosa*

*thinbaw-zibyu*

*Phyllanthus acidus*

*thingbaung*

*Buchanania lancifolia*

*thingu-gyat*

*Flemingia strobilifera*

*thin-paung*

*Hibiscus vitifolius*

*thitcho-khaya*

*Mimusops elengi*

*thit-hmwe*

*Aquilaria malaccensis*

*thit-jaboe*

*Cinnamomum tamala*

*thit-kado*

*Toona sureni*

*thit-kyabo*

*Cinnamomum bejolghota*

*Cinnamomum verum*

*thit-magyi*

*Albizia odoratissima*

*thit-ni*

*Aglaia cucullata*

*Aphanamixis polystachya*

*thit-seint*

*Terminalia bellirica*

*thitsi-bo*

*Semecarpus anacardium*

*thitto*

*Sandoricum koetjape*

*thitya-byu*

*Schima wallichii*

*thityah*

*Schima wallichii*

*thitya-ni*

*Schima wallichii*

thorn apple

*Datura metel*

*Datura stramonium*

thorny amaranthus

*Amaranthus spinosus*

threelobe false mallow

*Malvastrum coromandelianum*

*thunge-che*

*Helicteres isora*

*thun-vong*

*Gmelina arborea*

ti plant

*Cordyline fruticosa*

tick clover

*Phyllodium pulchellum*

*Tadehagi triquetrum*

tick trefoil

*Phyllodium pulchellum*

*Tadehagi triquetrum*

tiger’s claw

*Martynia annua*

*tikayon*

*Mimosa pudica*

*tike-pan*

*Clerodendrum thomsoniae*

*tike-tot-grine*

*Convolvulus arvensis*

*tila-pup-hpi*

*Digitalis purpurea*

*tingkyut*

*Helicteres isora*

tinnevelly senna

*Senna alexandrina*

*tiu khon tree*

*Cratoxylum formosum*

*tnhlium*

*Salix tetrasperma*

*to-ma-awn*

*Mucuna pruriens*

*toon*

*Ricinus communis*

touch-me-not

*Mimosa pudica*

tree cotton

*Gossypium barbadense*

tree crinum

*Crinum asiaticum*

tropical almond

*Terminalia catappa*

tropical bleeding heart

*Clerodendrum thomsoniae*

tropical fanleaf

*Hibiscus vitifolius*

tropical laurel

*Ficus benjamina*

tropical rose mallow

*Hibiscus vitifolius*

tropical white morning-glory

*Ipomoea alba*

tropical whiteweed

*Ageratum conyzoides*

true ginger

*Zingiber officinale*

true hemp

*Cannabis sativa*

true sandalwood

*Santalum album*

trumpet flower

*Stereospermum colais*

trumpet-bush

*Tecoma stans*

tubeflower

*Clerodendrum indicum*

*Rotheca incisa*

tumeric

*Curcuma comosa*

tummy wood

*Careya arborea*

*tun-kong*

*Jatropha curcas*

*tun-paw-man*

*Cordia dichotoma*

*tun-sa-se*

*Acacia catechu*

turkey bush

*Grewia polygama*

turmeric

*Curcuma longa*

turnsole

*Heliotropium indicum*

turpeth root

*Ipomoea alba*

*twinnet*

*Ichnocarpus frutescens*

*twinnet-kado*

*Ichnocarpus frutescens*

*ulat-kam-bala*

*Abroma augustum*

*umawng*

*Croton persimilis*

umbrella tree

*Schefflera venulosa*

*umung*

*Mangifera indica*

upas tree

*Antiaris toxicaria*

*u-wa-yaing*

*Daucus carota*

varnishtree

*Semecarpus anacardium*

vegetable humming-bird

*Sesbania grandiflora*

vegetable sponge

*Luffa cylindrica*

velvet bean

*Mucuna pruriens*

velvet leaf

*Cissampelos pareira*

vinca

*Catharanthus roseus*

*wachyang*

*Melastoma malabathricum*

wadalee-gum tree

*Acacia catechu*

*wagangga*

*Melastoma malabathricum*

*wah*

*Gossypium hirsutum*

Wallich milk parsely

*Selinum wallichianum*

*wa-mayar*

*Litchi chinensis*

*wa-pasang*

*Syzygium jambos*

*wa-passan*

*Syzygium cumini*

water cress

*Enydra fluctuans*

water-filter nut

*Strychnos potatorum*

waterspinach

*Ipomoea aquatica*

*wa-u-bin*

*Amorphophallus paeoniifolius*

*wa-u-pin*

*Amorphophallus paeoniifolius*

wax gourd

*Benincasa hispida*

weak horsetail

Equisetum ramosissimum subsp. debile

*wee-ek*

*Premna amplectens*

weeping laurel

*Ficus benjamina*

West Indian almond

*Terminalia catappa*

West Indian blackthorn

*Acacia farnesiana*

West Indian pea tree

*Sesbania grandiflora*

Westland’s rhododendron

*Rhododendron moulmainense*

*wetkyein*

*Persicaria chinensis*

*Pteridium aquilinum*

*wet-myet-nyo*

*Cyperus scariosus*

white butterfly bush

*Buddleja asiatica*

white cedar

*Aphanamixis polystachya*

white eclipta

*Eclipta prostrata*

white gourd

*Benincasa hispida*

white heads

*Eclipta prostrata*

white leadwort

*Plumbago zeylanica*

white man’s foot

*Plantago major*

white mustard

*Sinapis alba*

white pavetta

*Pavetta indica*

white sandalwood

*Santalum album*

white silk-cottontree

*Ceiba pentandra*

white-barked acacia

*Acacia leucophloea*

wild balsam apple

*Momordica charantia*

wild cabbage

*Brassica oleracea*

wild carrot

*Daucus carota*

wild cassia

*Cinnamomum bejolghota*

wild celery

*Apium graveolens*

*Trachyspermum roxburghianum*

wild cock’s comb

*Celosia argentea*

wild eggplant

*Solanum rudepannum*

wild ginger

*Zingiber montanum*

wild indigo

*Tephrosia purpurea*

wild jujube

*Ziziphus rugosa*

wild mango

*Spondias pinnata*

wild mint

*Mentha arvensis*

wild oil nut

*Jatropha curcas*

wild okra

*Abelmoschus esculentus*

wild portulaca

*Portulaca oleracea*

wild saffron

*Carthamus tinctorius*

wild sage

Lantana × aculeata

wild sarsaparilla

*Smilax guianensis*

wild snake gourd

*Coccinia grandis*

wild tamarind

*Leucaena leucocephala*

wild water-lemon

*Passiflora foetida*

wildhops

*Flemingia strobilifera*

willow

*Salix tetrasperma*

wine palm

*Caryota mitis*

*wine-baing*

*Cymbopogon citratus*

winter cherry

*Cardiospermum halicacabum*

winterberry

*Euonymus kachinensis*

wodier

*Lannea coromandelica*

woman’s tongue

*Albizia lebbeck*

wonder-tree

*Ricinus communis*

wood apple

*Limonia acidissima*

woodfordia

*Woodfordia fruticosa*

woolly dyeing rosebay

*Wrightia arborea*

woolly foxglove

*Digitalis lanata*

wormseed

*Dysphania ambrosioides*

*yang-bau*

*Alnus nepalensis*

*yangmaw*

*Haldina cordifolia*

*yan-nung*

*Mucuna pruriens*

*yat*

*Carallia brachiata*

*yaung-ma-ywet*

*Phyllanthus niruri*

*ye-chaung-pan*

*Stachytarpheta indica*

*ye-hmyok*

*Mallotus nudiflorus*

*ye-ka-on*

*Ficus semicordata*

*ye-kazun*

*Ipomoea aquatica*

*ye-kyi*

*Barringtonia acutangula*

yellow champak

*Magnolia champaca*

yellow crown-head

*Sigesbeckia orientalis*

yellow jasmine

*Jasminum humile*

yellow oleander

*Cascabela thevetia*

yellow prickly poppy

*Argemone mexicana*

yellow snake tree

*Stereospermum colais*

yellow snakeroot

*Stereospermum chelonoides*

yellow teak

*Haldina cordifolia*

yellow trumpet-bush

*Tecoma stans*

yellow-bells

*Tecoma stans*

yellow-elder

*Tecoma stans*

yellowtop

*Senecio densiflorus*

*ye-magyi*

*Justicia adhatoda*

*yemane*

*Gmelina arborea*

*yema-u*

*Neolamarckia cadamba*

*ye-ma-u*

*Neolamarckia cadamba*

*ye-minga*

*Cynometra ramiflora*

*ye-mya-yar*

*Grewia nervosa*

*yene*

*Salix tetrasperma*

*yengan-bok*

*Diospyros malabarica*

*ye-padauk*

*Bischofia javanica*

*yepadon*

*Bischofia javanica*

yerba de caballo

*Elephantopus scaber*

yerba de tago

*Eclipta prostrata*

*ye-tazwet*

*Grangea maderaspatana*

*ye-thabye*

*Salix tetrasperma*

*Syzygium nervosum*

*ye-tha-gyi*

*Sesbania sesban*

*yetkyi*

*Woodfordia fruticosa*

yew

*Taxus baccata*

*yinbya*

*Rotheca serrata*

*yinbya-byu*

*Premna amplectens*

*yinbya-net*

*Rotheca serrata*

*yinma*

*Chukrasia tabularis*

ylang-ylang

*Cananga odorata*

*yonbade*

*Abelmoschus esculentus*

*yong*

*Senna sulfurea*

*yun-ha*

*Schleichera oleosa*

*yuzara*

*Chloranthus elatior*

*ywe*

*Abrus precatorius*

*Adenanthera pavonina*

*ywe-gyi*

*Adenanthera pavonina*

*ywenge*

*Abrus precatorius*

*ywe-nge*

*Abrus precatorius*

*ywe-ni*

*Adenanthera pavonina*

*ywe-nwe*

*Abrus precatorius*

*ywet-kya-pin-bauk*

*Bryophyllum pinnatum*

*za-gwe-pan*

*Pavetta indica*

*zalat*

*Tabernaemontana divaricate*

*zalat-pyu*

*Rhododendron moulmainense*

*zalat-seikya*

*Tabernaemontana divaricate*

*zalut-ni*

*Kopsia fruticosa*

*zalut-panyaung*

*Kopsia fruticosa*

*zar-date-hpo*

*Myristica fragrans*

*zar-pwint*

*Myristica fragrans*

*zaung-ya*

*Averrhoa carambola*

*zawgyi taung whay pin*

*Cordyline fruticosa*

*zawma*

*Cordyline fruticosa*

zedoary

*Curcuma zedoaria*

*zee-hpyu*

*Phyllanthus emblica*

*zi*

*Ziziphus jujuba*

*zibyu*

*Phyllanthus emblica*

*zi-daw-thi*

*Ziziphus jujuba*

*zi-ganauk*

*Ziziphus rugosa*

*zi-talaing*

*Ziziphus rugosa*

*zizaung*

*Euphorbia neriifolia*

*zun-burr*

*Lannea coromandelica*

## Appendix 2

**Species index**

*Abelmoschus esculentus* (Malvaceae)

*Abelmoschus moschatus* (Malvaceae)

*Abroma augustum* (Malvaceae)

*Abrus precatorius* (Fabaceae)

*Acacia catechu* (Fabaceae)

*Acacia concinna* (Fabaceae)

*Acacia farnesiana* (Fabaceae)

*Acacia leucophloea* (Fabaceae)

*Acacia nilotica* (Fabaceae)

*Acacia pennata* (Fabaceae)

*Acalypha indica* (Euphorbiaceae)

*Acalypha wilkesiana* (Euphorbiaceae)

*Acanthus ilicifolius* (Acanthaceae)

*Achyranthes aspera* (Amaranthaceae)

*Acorus calamus* (Acoraceae)

*Adenanthera pavonina* (Fabaceae)

*Aeginetia indica* (Orobanchaceae)

*Aegle marmelos* (Rutaceae)

*Aerva javanica* (Amaranthaceae)

*Agave sisalana* (Asparagaceae)

*Agave vera-cruz* (Asparagaceae)

*Aglaia cucullata* (Meliaceae)

*Ageratum conyzoides* (Asteraceae)

*Agrimonia eupatoria* (Rosaceae)

*Albizia lebbeck* (Fabaceae)

*Albizia odoratissima* (Fabaceae)

*Allamanda cathartica* (Apocynaceae)

*Allium cepa* (Amaryllidaceae)

*Allium sativum* (Amaryllidaceae)

*Alnus nepalensis* (Betulaceae)

*Aloe vera* (Asphodelaceae)

*Alpinia galanga* (Zingiberaceae)

*Alpinia officinarum* (Zingiberaceae)

*Alstonia scholaris* (Apocynaceae)

*Alternanthera sessilis* (Amaranthaceae)

*Altingia excelsa* (Altingiaceae)

*Alysicarpus vaginalis* (Fabaceae)

*Amaranthus cruentus* (Amaranthaceae)

*Amaranthus spinosus* (Amaranthaceae)

*Amherstia nobilis* (Fabaceae)

*Amorphophallus paeoniifolius* (Araceae)

*Anacardium occidentale* (Anacardiaceae)

*Andrographis paniculata* (Acanthaceae)

*Anethum graveolens* (Apiaceae)

*Anneslea fragrans* (Pentaphylacaceae)

*Annona squamosa* (Annonaceae)

*Antiaris toxicaria* (Moraceae)

*Aphanamixis polystachya* (Meliaceae)

*Apium graveolens* (Apiaceae)

*Aquilaria malaccensis* (Thymelaeaceae)

*Arachis hypogaea* (Fabaceae)

*Archidendron jiringa* (Fabaceae)

*Ardisia humilis* (Primulaceae)

*Argemone mexicana* (Papaveraceae)

*Aristolochia indica* (Aristolochiaceae)

*Aristolochia tagala* (Aristolochiaceae)

*Artabotrys hexapetalus* (Annonaceae)

*Artemisia dracunculus* (Asteraceae)

*Artocarpus heterophyllus* (Moraceae)

*Artocarpus lakoocha* (Moraceae)

*Arundo donax* (Poaceae)

*Asclepias curassavica* (Apocynaceae)

*Asparagus filicinus* (Asparagaceae)

*Asparagus officinalis* (Asparagaceae)

*Averrhoa carambola* (Oxalidaceae)

*Avicennia officinalis* (Acanthaceae)

*Azadirachta indica* (Meliaceae)

*Bambusa bambos* (Poaceae)

*Barleria prionitis* (Acanthaceae)

*Barringtonia acutangula* (Lecythidaceae)

*Basella alba* (Basellaceae)

*Bauhinia acuminata* (Fabaceae)

*Bauhinia purpurea* (Fabaceae)

*Benincasa hispida* (Cucurbitaceae)

*Berberis nepalensis* (Berberidaceae)

*Bischofia javanica* (Phyllanthaceae)

*Bixa orellana* (Bixaceae)

*Blumea balsamifera* (Asteraceae)

*Boehmeria nivea* (Urticaceae)

*Boerhavia diffusa* (Nyctaginaceae)

*Bombax ceiba* (Malvaceae)

*Bougainvillea spectabilis* (Nyctaginaceae)

*Brassica oleracea* (Brassicaceae)

*Bridelia retusa* (Phyllanthaceae)

*Brugmansia arborea* (Solanaceae)

*Brugmansia suaveolens* (Solanaceae)

*Bryophyllum pinnatum* (Crassulaceae)

*Buddleja asiatica* (Scrophulariaceae)

*Buchanania lancifolia* (Anacardiaceae)

*Butea monosperma* (Fabaceae)

*Butea superba* (Fabaceae)

*Caesalpinia pulcherrima* (Fabaceae)

*Callicarpa macrophylla* (Lamiaceae)

*Calophyllum inophyllum* (Calophyllaceae)

*Calotropis gigantea* (Apocynaceae)

*Calotropis procera* (Apocynaceae)

*Cananga odorata* (Annonaceae)

*Canavalia ensiformis* (Fabaceae)

*Canna indica* (Cannaceae)

*Cannabis sativa* (Cannabaceae)

*Capparis flavicans* (Capparaceae)

*Capparis zeylanica* (Capparaceae)

*Capsicum annuum* (Solanaceae)

*Carallia brachiata* (Rhizophoraceae)

*Cardiospermum halicacabum* (Sapindaceae)

*Careya arborea* (Lecythidaceae)

*Carica papaya* (Caricaceae)

*Carissa spinarum* (Apocynaceae)

*Carthamus tinctorius* (Asteraceae)

*Caryota mitis* (Arecaceae)

*Cascabela thevetia* (Apocynaceae)

*Cassia fistula* (Fabaceae)

*Casuarina equisetifolia* (Casuarinaceae)

*Catharanthus roseus* (Apocynaceae)

*Catunaregam spinosa* (Rubiaceae)

*Ceiba pentandra* (Malvaceae)

*Celastrus paniculatus* (Celastraceae)

*Celosia argentea* (Amaranthaceae)

*Centella asiatica* (Apiaceae)

*Chamaecrista pumila* (Fabaceae)

*Cheilocostus speciosus* (Costaceae)

*Chenopodium album* (Amaranthaceae)

*Chloranthus elatior* (Chloranthaceae)

*Chromolaena odorata* (Asteraceae)

*Chrozophora plicata* (Euphorbiaceae)

*Chukrasia tabularis* (Meliaceae)

*Cinnamomum bejolghota* (Lauraceae)

*Cinnamomum camphora* (Lauraceae)

*Cinnamomum tamala* (Lauraceae)

*Cinnamomum verum* (Lauraceae)

*Cissampelos pareira* (Menispermaceae)

*Citrus aurantiifolia* (Rutaceae)

Citrus × aurantium (Rutaceae)

*Citrus limon* (Rutaceae)

*Claoxylon indicum* (Euphorbiaceae)

*Clausena excavata* (Rutaceae)

*Clematis smilacifolia* (Ranunculaceae)

*Cleome gynandra* (Cleomaceae)

*Clerodendrum indicum* (Lamiaceae)

*Clerodendrum infortunatum* (Lamiaceae)

*Clerodendrum thomsoniae* (Lamiaceae)

*Clitoria ternatea* (Fabaceae)

*Coccinia grandis* (Crassulaceae)

*Coffea arabica* (Rubiaceae)

*Coix lacryma-jobi* (Poaceae)

*Colebrookea oppositifolia* (Lamiaceae)

*Colocasia antiquorum* (Araceae)

*Combretum indicum* (Combretaceae)

*Commelina paludosa* (Commelinaceae)

*Commicarpus chinensis* (Nyctaginaceae)

*Convolvulus arvensis* (Convolvulaceae)

*Coptis teeta* (Ranunculaceae)

*Cordia dichotoma* (Boraginaceae)

*Cordia myxa* (Boraginaceae)

*Cordyline fruticosa* (Laxmanniaceae)

*Coriandrum sativum* (Apiaceae)

*Coriaria nepalensis* (Coriariaceae)

*Crateva religiosa* (Capparaceae)

*Cratoxylum formosum* (Hypericaceae)

*Crinum asiaticum* (Amaryllidaceae)

*Croton persimilis* (Euphorbiaceae)

*Croton tiglium* (Euphorbiaceae)

*Cucumis sativus* (Cucurbitaceae)

*Cullen corylifolium* (Fabaceae)

*Cupressus goveniana* (Cupressaceae)

*Curcuma comosa* (Zingiberaceae)

*Curcuma longa* (Zingiberaceae)

*Curcuma zedoaria* (Zingiberaceae)

*Cuscuta reflexa* (Convolvulaceae)

*Cyanthillium cinereum* (Asteraceae)

*Cycas rumphii* (Cycadaceae)

*Cymbopogon citratus* (Poaceae)

*Cymbopogon jwarancusa* (Poaceae)

*Cymbopogon nardus* (Poaceae)

*Cynometra ramiflora* (Fabaceae)

*Cyperus scariosus* (Cyperaceae)

*Dactyloctenium aegyptium* (Poaceae)

*Datura metel* (Solanaceae)

*Datura stramonium* (Solanaceae)

*Daucus carota* (Apiaceae)

*Delonix regia* (Fabaceae)

*Dicranopteris linearis* (Gleicheniaceae)

*Digitalis lanata* (Plantaginaceae)

*Digitalis purpurea* (Plantaginaceae)

*Dillenia indica* (Dilleniaceae)

*Dimocarpus longan* (Sapindaceae)

*Diospyros malabarica* (Ebenaceae)

*Diospyros mollis* (Ebenaceae)

*Dioscorea bulbifera* (Dioscoreaceae)

*Dioscorea pentaphylla* (Dioscoreaceae)

*Dodonaea viscosa* (Sapindaceae)

*Dracaena angustifolia* (Asparagaceae)

*Dregea volubilis* (Apocynaceae)

*Dysphania ambrosioides* (Amaranthaceae)

*Eclipta prostrata* (Asteraceae)

*Elephantopus scaber* (Asteraceae)

*Elettaria cardamomum* (Zingiberaceae)

*Emilia sonchifolia* (Asteraceae)

*Enydra fluctuans* (Asteraceae)

*Entada phaseoloides* (Fabaceae)

Equisetum ramosissimum subsp. debile (Equisetaceae)

*Eryngium caeruleum* (Apiaceae)

*Erythrina variegata* (Fabaceae)

*Eucalyptus globulus* (Myrtaceae)

*Euonymus kachinensis* (Celastraceae)

*Euphorbia antiquorum* (Euphorbiaceae)

*Euphorbia hirta* (Euphorbiaceae)

*Euphorbia neriifolia* (Euphorbiaceae)

*Eurycoma longifolia* (Simaroubaceae)

*Evolvulus alsinoides* (Convolvulaceae)

*Exacum tetragonum* (Gentianaceae)

*Fagopyrum esculentum* (Polygonaceae)

*Ficus benjamina* (Moraceae)

*Ficus hispida* (Moraceae)

*Ficus religiosa* (Moraceae)

*Ficus retusa* (Moraceae)

*Ficus rumphii* (Moraceae)

*Ficus semicordata* (Moraceae)

*Flacourtia jangomas* (Salicaceae)

*Flemingia chappar* (Fabaceae)

*Flemingia strobilifera* (Fabaceae)

*Foeniculum vulgare* (Apiaceae)

*Fritillaria cirrhosa* (Liliaceae)

Garcinia × mangostana (Clusiaceae)

*Garcinia xanthochymus* (Clusiaceae)

*Garuga pinnata* (Burseraceae)

*Girardinia diversifolia* (Urticaceae)

*Gloriosa superba* (Colchicaceae)

*Glycine max* (Fabaceae)

*Gmelina arborea* (Lamiaceae)

*Gossypium barbadense* (Malvaceae)

*Gossypium hirsutum* (Malvaceae)

*Gouania leptostachya* (Rhamnaceae)

*Grangea maderaspatana* (Asteraceae)

*Grewia asiatica* (Malvaceae)

*Grewia hirsuta* (Malvaceae)

*Grewia nervosa* (Malvaceae)

*Grewia polygama* (Malvaceae)

*Haldina cordifolia* (Rubiaceae)

*Helicteres isora* (Malvaceae)

*Heliotropium indicum* (Boraginaceae)

*Heynea trijuga* (Meliaceae)

*Hibiscus cannabinus* (Malvaceae)

*Hibiscus sabdariffa* (Malvaceae)

*Hibiscus schizopetalus* (Malvaceae)

*Hibiscus vitifolius* (Malvaceae)

*Hiptage benghalensis* (Malpighiceae)

*Holarrhena pubescens* (Apocynaceae)

*Holoptelea integrifolia* (Ulmaceae)

*Hydnocarpus kurzii* (Achariaceae)

*Hydrolea zeylanica* (Hydroleaceae)

*Hygrophila auriculata* (Acanthaceae)

*Hygrophila phlomoides* (Acanthaceae)

*Hymenodictyon orixense* (Rubiaceae)

*Ichnocarpus frutescens* (Apocynaceae)

*Indigofera cassioides* (Fabaceae)

*Ipomoea alba* (Convolvulaceae)

*Ipomoea aquatica* (Convolvulaceae)

*Ipomoea hederifolia* (Convolvulaceae)

*Ipomoea pes-caprae* (Convolvulaceae)

*Ixora chinensis* (Rubiaceae)

*Ixora coccinea* (Rubiaceae)

*Jasminum humile* (Oleaceae)

*Jasminum multiflorum* (Oleaceae)

*Jatropha curcas* (Euphorbiaceae)

*Jatropha gossypiifolia* (Euphorbiaceae)

*Jatropha multifida* (Euphorbiaceae)

*Justicia adhatoda* (Acanthaceae)

*Kaempferia elegans* (Zingiberaceae)

*Kleinhovia hospita* (Malvaceae)

*Kopsia fruticosa* (Apocynaceae)

*Kydia calycina* (Malvaceae)

*Lablab purpureus* (Fabaceae)

*Lagerstroemia speciosa* (Lythraceae)

*Lannea coromandelica* (Anacardiaceae)

Lantana × aculeata (Verbenaceae)

*Leea macrophylla* (Vitaceae)

*Leucaena leucocephala* (Fabaceae)

*Leucas cephalotes* (Lamiaceae)

*Limonia acidissima* (Rutaceae)

*Linostoma pauciflorum* (Thymelaeaceae)

*Linum usitatissimum* (Linaceae)

*Litchi chinensis* (Sapindaceae)

*Luffa cylindrica* (Cucurbitaceae)

*Magnolia champaca* (Magnoliaceae)

*Mallotus nudiflorus* (Euphorbiaceae)

*Mallotus philippensis* (Euphorbiaceae)

*Malvastrum coromandelianum* (Malvaceae)

*Mangifera indica* (Anacardiaceae)

*Manilkara zapota* (Sapotaceae)

*Mansonia gagei* (Malvaceae)

*Maranta arundinacea* (Marantaceae)

*Markhamia stipulata* (Bignoniaceae)

*Martynia annua* (Martyniaceae)

*Mayodendron igneum* (Bignoniaceae)

*Melaleuca cajuputi* (Myrtaceae)

*Melastoma malabathricum* (Melastomataceae)

*Memecylon edule* (Melastomataceae)

*Mentha arvensis* (Lamiaceae)

*Mesua ferrea* (Calophyllaceae)

*Millettia pachycarpa* (Fabaceae)

*Millingtonia hortensis* (Bignoniaceae)

*Mimosa pudica* (Fabaceae)

*Mimusops elengi* (Sapotaceae)

*Mirabilis jalapa* (Nyctaginaceae)

*Mitragyna speciosa* (Rubiaceae)

*Momordica charantia* (Cucurbitaceae)

*Momordica cochinchinensis* (Cucurbitaceae)

*Monochoria vaginalis* (Pontederiaceae)

*Morinda angustifolia* (Rubiaceae)

*Morinda citrifolia* (Rubiaceae)

*Morinda coreia* (Rubiaceae)

*Moringa oleifera* (Moringaceae)

*Mucuna pruriens* (Fabaceae)

*Mussaenda macrophylla* (Rubiaceae)

*Myristica fragrans* (Myristicaceae)

*Nauclea orientalis* (Rubiaceae)

*Neolamarckia cadamba* (Rubiaceae)

*Nerium oleander* (Apocynaceae)

*Nicandra physalodes* (Solanaceae)

*Nigella sativa* (Ranunculaceae)

*Nyctanthes arbor-tristis* (Oleaceae)

*Nymphaea rubra* (Nymphaeaceae)

*Ocimum americanum* (Lamiaceae)

*Ocimum tenuiflorum* (Lamiaceae)

*Olax scandens* (Olacaceae)

*Oldenlandia corymbosa* (Rubiaceae)

*Oroxylum indicum* (Bignoniaceae)

*Orthosiphon aristatus* (Lamiaceae)

*Paederia foetida* (Rubiaceae)

*Passiflora foetida* (Passifloraceae)

*Passiflora quadrangularis* (Passifloraceae)

*Pavetta indica* (Rubiaceae)

*Peristrophe bicalyculata* (Acanthaceae)

*Persicaria chinensis* (Polygonaceae)

*Persicaria pulchra* (Polygonaceae)

*Phragmites karka* (Poaceae)

*Phyllanthus acidus* (Phyllanthaceae)

*Phyllanthus emblica* (Phyllanthaceae)

*Phyllanthus niruri* (Phyllanthaceae)

*Phyllodium pulchellum* (Fabaceae)

*Physalis peruviana* (Solanaceae)

*Picrasma javanica* (Simaroubaceae)

*Piper betle* (Piperaceae)

*Piper cubeba* (Piperaceae)

*Piper longum* (Piperaceae)

*Piper nigrum* (Piperaceae)

*Pithecellobium dulce* (Fabaceae)

*Plantago major* (Plantaginaceae)

*Plumbago indica* (Plumbaginaceae)

*Plumbago zeylanica* (Plumbaginaceae)

*Plumeria rubra* (Apocynaceae)

*Pogostemon cablin* (Lamiaceae)

*Polyalthia longifolia* (Annonaceae)

*Portulaca oleracea* (Portulaceae)

*Pothos scandens* (Araceae)

*Premna amplectens* (Lamiaceae)

*Premna mollissima* (Lamiaceae)

*Premna serratifolia* (Lamiaceae)

*Prunus cerasoides* (Rosaceae)

*Psidium guajava* (Myrtaceae)

*Pteridium aquilinum* (Dennstaedtiaceae)

*Pterospermum acerifolium* (Malvaceae)

*Punica granatum* (Lythraceae)

*Putranjiva roxburghii* (Putranjavaceae)

*Quassia indica* (Simaroubaceae)

*Rauvolfia serpentina* (Apocynaceae)

*Rhizophora mucronata* (Rhizophoraceae)

*Rhododendron moulmainense* (Ericaceae)

*Rhus chinensis* (Anacardiaceae)

*Ricinus communis* (Euphorbiaceae)

*Rotheca incisa* (Lamiaceae)

*Rotheca serrata* (Lamiaceae)

*Rubia cordifolia* (Rubiaceae)

*Salix tetrasperma* (Salicaceae)

*Salvia officinalis* (Lamiaceae)

*Sambucus javanica* (Adoxaceae)

*Sandoricum koetjape* (Meliaceae)

*Santalum album* (Santalaceae)

*Sapindus saponaria* (Sapindaceae)

*Saraca indica* (Fabaceae)

*Schefflera venulosa* (Araliaceae)

*Schima wallichii* (Theaceae)

*Schleichera oleosa* (Sapindaceae)

*Scoparia dulcis* (Plantaginaceae)

*Selinum wallichianum* (Apiaceae)

*Semecarpus anacardium* (Anacardiaceae)

*Senecio densiflorus* (Asteraceae)

*Senna alata* (Fabaceae)

*Senna alexandrina* (Fabaceae)

*Senna auriculata* (Fabaceae)

*Senna italica* (Fabaceae)

*Senna siamea* (Fabaceae)

*Senna sulfurea* (Fabaceae)

*Senna tora* (Fabaceae)

*Sesamum indicum* (Pedaliaceae)

*Sesbania grandiflora* (Fabaceae)

*Sesbania sesban* (Fabaceae)

*Sida spinosa* (Malvaceae)

*Sigesbeckia orientalis* (Asteraceae)

*Sinapis alba* (Brassicaceae)

*Smilax aspera* (Smilacaceae)

*Smilax glabra* (Smilacaceae)

*Smilax guianensis* (Smilacaceae)

*Solanum anguivi* (Solanaceae)

*Solanum melongena* (Solanaceae)

*Solanum rudepannum* (Solanaceae)

*Spatholobus parviflorus* (Fabaceae)

*Spermacoce hispida* (Rubiaceae)

*Spondias pinnata* (Anacardiaceae)

*Stachytarpheta indica* (Verbenaceae)

*Stereospermum chelonoides* (Bignoniaceae)

*Stereospermum colais* (Bignoniaceae)

*Streblus asper* (Moraceae)

*Strobilanthes auriculatus* (Acanthaceae)

*Strychnos potatorum* (Loganiaceae)

*Strychnos wallichiana* (Loganiaceae)

*Swertia chirayita* (Gentianaceae)

*Symplocos racemosa* (Symplocaceae)

*Syzygium aromaticum* (Myrtaceae)

*Syzygium cumini* (Myrtaceae)

*Syzygium jambos* (Myrtaceae)

*Syzygium nervosum* (Myrtaceae)

*Tabernaemontana divaricata* (Apocynaceae)

*Tadehagi triquetrum* (Fabaceae)

*Tagetes erecta* (Asteraceae)

*Tamarindus indica* (Fabaceae)

*Tamilnadia uliginosa* (Rubiaceae)

*Tanacetum cinerariifolium* (Asteraceae)

*Taxus baccata* (Taxaceae)

*Tecoma stans* (Bignoniaceae)

*Tectona grandis* (Lamiaceae)

*Tephrosia purpurea* (Fabaceae)

*Terminalia bellirica* (Combretaceae)

*Terminalia catappa* (Combretaceae)

*Terminalia chebula* (Combretaceae)

*Terminalia citrina* (Combretaceae)

*Terminalia tomentosa* (Combretaceae)

*Thunbergia erecta* (Acanthaceae)

*Thunbergia laurifolia* (Acanthaceae)

*Tinospora cordifolia* (Menispermaceae)

*Toona sureni* (Meliaceae)

*Trachyspermum ammi* (Apiaceae)

*Trachyspermum roxburghianum* (Apiaceae)

*Tradescantia spathacea* (Commelinaceae)

*Trichosanthes tricuspidata* (Cucurbitaceae)

*Triumfetta rhomboidea* (Malvaceae)

*Typhonium trilobatum* (Araceae)

*Urena lobata* (Malvaceae)

*Urtica dioica* (Urticaceae)

*Urtica parviflora* (Urticaceae)

*Vaccaria hispanica* (Caryophyllaceae)

*Vallaris solanacea* (Apocynaceae)

*Ventilago denticulata* (Rhamnaceae)

*Verbena officinalis* (Verbenaceae)

*Viscum cruciatum* (Santalaceae)

*Vitex glabrata* (Lamiaceae)

*Vitex negundo* (Lamiaceae)

*Vitex trifolia* (Lamiaceae)

*Volkameria inermis* (Lamiaceae)

*Walsura pinnata* (Meliaceae)

*Woodfordia fruticosa* (Lythraceae)

*Wrightia arborea* (Apocynaceae)

*Xylia xylocarpa* (Fabaceae)

*Xylocarpus granatum* (Meliaceae)

*Xylocarpus moluccensis* (Meliaceae)

*Zanthoxylum acanthopodium* (Rutaceae)

*Zea mays* (Poaceae)

*Zingiber montanum* (Zingiberaceae)

*Zingiber officinale* (Zingiberaceae)

*Zingiber zerumbet* (Zingiberaceae)

*Ziziphus jujuba* (Rhamnaceae)

*Ziziphus rugosa* (Rhamnaceae)

## Appendix 3

**Medicinal index**

**Abdominal illnesses**

*Aquilaria malaccensis* (Thymelaeaceae)

**Abortive**

*Buddleja asiatica* (Scrophulariaceae)

*Gloriosa superba* (Colchicaceae)

*Momordica charantia* (Cucurbitaceae)

*Pithecellobium dulce* (Fabaceae)

*Sesamum indicum* (Pedaliaceae)

**Abscesses**

*Alstonia scholaris* (Apocynaceae)

*Artocarpus heterophyllus* (Moraceae)

**Acne**

*Aerva javanica* (Amaranthaceae)

*Cardiospermum halicacabum* (Sapindaceae)

**Alcoholism**

*Croton persimilis* (Euphorbiaceae)

**Alertness**

*Annona squamosa* (Annonaceae)

**Analgesic**

*Cannabis sativa* (Cannabaceae)

*Elephantopus scaber* (Asteraceae)

*Flemingia chappar* (Fabaceae)

*Momordica charantia* (Cucurbitaceae)

*Tagetes erecta* (Asteraceae)

**Anemia**

*Asparagus officinalis* (Asparagaceae)

*Cardiospermum halicacabum* (Sapindaceae)

*Convolvulus arvensis* (Convolvulaceae)

*Fritillaria cirrhosa* (Liliaceae)

*Mansonia gagei* (Malvaceae)

*Momordica charantia* (Cucurbitaceae)

*Plumbago indica* (Plumbaginaceae)

*Rauvolfia serpentina* (Apocynaceae)

*Sesbania grandiflora* (Fabaceae)

*Terminalia bellirica* (Combretaceae)

*Terminalia citrina* (Combretaceae)

**Anodyne**

*Cannabis sativa* (Cannabaceae)

*Dactyloctenium aegyptium* (Poaceae)

**Anthelmintic**

*Acalypha indica* (Euphorbiaceae)

*Anacardium occidentale* (Anacardiaceae)

*Andrographis paniculata* (Acanthaceae)

*Asparagus filicinus* (Asparagaceae)

*Azadirachta indica* (Meliaceae)

*Calotropis gigantea* (Apocynaceae)

*Cordia dichotoma* (Boraginaceae)

*Cucumis sativus* (Cucurbitaceae)

*Dicranopteris linearis* (Gleicheniaceae)

*Diospyros mollis* (Ebenaceae)

*Dysphania ambrosioides* (Amaranthaceae)

*Emilia sonchifolia* (Asteraceae)

*Evolvulus alsinoides* (Convolvulaceae)

*Grangea maderaspatana* (Asteraceae)

*Jatropha curcas* (Euphorbiaceae)

*Mallotus philippensis* (Euphorbiaceae)

*Momordica charantia* (Cucurbitaceae)

*Oldenlandia corymbosa* (Rubiaceae)

*Pteridium aquilinum* (Dennstaedtiaceae)

*Punica granatum* (Lythraceae)

*Ricinus communis* (Euphorbiaceae)

*Saraca indica* (Fabaceae)

*Schima wallichii* (Theaceae)

*Selinum wallichianum* (Apiaceae)

*Tephrosia purpurea* (Fabaceae)

**Antiasthmatic**

*Achyranthes aspera* (Amaranthaceae)

*Artemisia dracunculus* (Asteraceae)

*Brugmansia arborea* (Solanaceae)

*Brugmansia suaveolens* (Solanaceae)

*Calotropis gigantea* (Apocynaceae)

*Cullen corylifolium* (Fabaceae)

*Cyanthillium cinereum* (Asteraceae)

*Datura stramonium* (Solanaceae)

*Dicranopteris linearis* (Gleicheniaceae)

*Evolvulus alsinoides* (Convolvulaceae)

*Mangifera indica* (Anacardiaceae)

*Pothos scandens* (Araceae)

*Semecarpus anacardium* (Anacardiaceae)

**Antidysenteric**

*Antiaris toxicaria* (Moraceae)

*Eurycoma longifolia* (Simaroubaceae)

*Terminalia chebula* (Combretaceae)

**Antiemetic**

*Ficus religiosa* (Moraceae)

*Syzygium aromaticum* (Myrtaceae)

**Antiherpetic**

*Cynometra ramiflora* (Fabaceae)

**Antihysteric**

*Abelmoschus moschatus* (Malvaceae)

**Antiparasitic**

*Tanacetum cinerariifolium* (Asteraceae)

**Antiphylogistic**

*Viscum cruciatum* (Santalaceae)

**Antipyretic**

*Andrographis paniculata* (Acanthaceae)

*Antiaris toxicaria* (Moraceae)

*Barleria prionitis* (Acanthaceae)

*Bixa orellana* (Bixaceae)

*Celosia argentea* (Amaranthaceae)

*Cissampelos pareira* (Menispermaceae)

*Cleome gynandra* (Cleomaceae)

*Clerodendrum infortunatum* (Lamiaceae)

*Commelina paludosa* (Commelinaceae)

*Delonix regia* (Fabaceae)

*Dicranopteris linearis* (Gleicheniaceae)

*Elephantopus scaber* (Asteraceae)

*Emilia sonchifolia* (Asteraceae)

*Entada phaseoloides* (Fabaceae)

*Erythrina variegata* (Fabaceae)

*Grangea maderaspatana* (Asteraceae)

*Hymenodictyon orixense* (Rubiaceae)

*Ichnocarpus frutescens* (Apocynaceae)

*Lablab purpureus* (Fabaceae)

*Manilkara zapota* (Sapotaceae)

*Momordica charantia* (Cucurbitaceae)

*Nauclea orientalis* (Rubiaceae)

*Neolamarckia cadamba* (Rubiaceae)

*Nymphaea rubra* (Nymphaeaceae)

*Ocimum americanum* (Lamiaceae)

*Ocimum tenuiflorum* (Lamiaceae)

*Oldenlandia corymbosa* (Rubiaceae)

*Polyalthia longifolia* (Annonaceae)

*Salix tetrasperma* (Salicaceae)

*Stereospermum chelonoides* (Bignoniaceae)

*Stereospermum colais* (Bignoniaceae)

*Swertia chirayita* (Gentianaceae)

*Syzygium aromaticum* (Myrtaceae)

*Tephrosia purpurea* (Fabaceae)

*Vitex trifolia* (Lamiaceae)

*Zanthoxylum acanthopodium* (Rutaceae)

**Antirheumatic**

*Clematis smilacifolia* (Ranunculaceae)

*Paederia foetida* (Rubiaceae)

*Sesamum indicum* (Pedaliaceae)

**Antiscorbutic**

*Persicaria chinensis* (Polygonaceae)

*Phyllanthus emblica* (Phyllanthaceae)

*Spondias pinnata* (Anacardiaceae)

**Antiseptic**

*Ageratum conyzoides* (Asteraceae)

*Annona squamosa* (Annonaceae)

*Artemisia dracunculus* (Asteraceae)

*Bischofia javanica* (Phyllanthaceae)

*Blumea balsamifera* (Asteraceae)

*Carissa spinarum* (Apocynaceae)

*Colebrookea oppositifolia* (Lamiaceae)

*Justicia adhatoda* (Acanthaceae)

*Mimosa pudica* (Fabaceae)

*Salvia officinalis* (Lamiaceae)

*Selinum wallichianum* (Apiaceae)

*Tagetes erecta* (Asteraceae)

**Antispasmodic**

*Abelmoschus moschatus* (Malvaceae)

*Anethum graveolens* (Apiaceae)

*Blumea balsamifera* (Asteraceae)

*Cinnamomum camphora* (Lauraceae)

*Clausena excavata* (Rutaceae)

*Cymbopogon nardus* (Poaceae)

*Dactyloctenium aegyptium* (Poaceae)

*Grangea maderaspatana* (Asteraceae)

*Lablab purpureus* (Fabaceae)

Lantana × aculeata (Verbenaceae)

*Salvia officinalis* (Lamiaceae)

*Solanum anguivi* (Solanaceae)

*Taxus baccata* (Taxaceae)

**Antisyphilitic**

*Tecoma stans* (Bignoniaceae)

**Anxiety**

*Tinospora cordifolia* (Menispermaceae)

**Aperient. *See* Laxative.**

**Aphrodisiac**

*Avicennia officinalis* (Acanthaceae)

*Celosia argentea* (Amaranthaceae)

*Cinnamomum verum* (Lauraceae)

*Cycas rumphii* (Cycadaceae)

*Hygrophila auriculata* (Acanthaceae)

*Momordica charantia* (Cucurbitaceae)

*Mucuna pruriens* (Fabaceae)

**Appetite improver**

*Acacia pennata* (Fabaceae)

*Allium cepa* (Amaryllidaceae)

*Allium sativum* (Amaryllidaceae)

*Alpinia galanga* (Zingiberaceae)

*Andrographis paniculata* (Acanthaceae)

*Arundo donax* (Poaceae)

*Canna indica* (Cannaceae)

*Citrus aurantiifolia* (Rutaceae)

*Citrus limon* (Rutaceae)

*Coix lacryma-jobi* (Poaceae)

*Croton tiglium* (Euphorbiaceae)

*Curcuma comosa* (Zingiberaceae)

*Cymbopogon citratus* (Poaceae)

*Cyperus scariosus* (Cyperaceae)

*Dregea volubilis* (Apocynaceae)

*Ficus religiosa* (Moraceae)

*Fritillaria cirrhosa* (Liliaceae)

*Ixora coccinea* (Rubiaceae)

*Momordica charantia* (Cucurbitaceae)

*Moringa oleifera* (Moringaceae)

*Myristica fragrans* (Myristicaceae)

*Ocimum americanum* (Lamiaceae)

*Piper betle* (Piperaceae)

*Piper nigrum* (Piperaceae)

*Plumbago indica* (Plumbaginaceae)

*Plumbago zeylanica* (Plumbaginaceae)

*Rotheca serrata* (Lamiaceae)

*Solanum anguivi* (Solanaceae)

*Tanacetum cinerariifolium* (Asteraceae)

*Tinospora cordifolia* (Menispermaceae)

*Trachyspermum ammi* (Apiaceae)

*Zingiber officinale* (Zingiberaceae)

**Arthritis**

*Croton persimilis* (Euphorbiaceae)

*Morinda citrifolia* (Rubiaceae)

**Ascites. *See also* Hydragogue.**

*Allamanda cathartica* (Apocynaceae)

**Asthma**

*Acacia pennata* (Fabaceae)

*Acalypha indica* (Euphorbiaceae)

*Aloe vera* (Asphodelaceae)

*Alpinia galanga* (Zingiberaceae)

*Alstonia scholaris* (Apocynaceae)

*Aquilaria malaccensis* (Thymelaeaceae)

*Benincasa hispida* (Cucurbitaceae)

*Boerhavia diffusa* (Nyctaginaceae)

*Calotropis procera* (Apocynaceae)

*Cinnamomum camphora* (Lauraceae)

*Citrus aurantiifolia* (Rutaceae)

*Citrus limon* (Rutaceae)

*Clerodendrum indicum* (Lamiaceae)

*Coccinia grandis* (Crassulaceae)

*Coptis teeta* (Ranunculaceae)

*Coriandrum sativum* (Apiaceae)

*Curcuma comosa* (Zingiberaceae)

*Curcuma longa* (Zingiberaceae)

*Cyperus scariosus* (Cyperaceae)

*Datura metel* (Solanaceae)

*Datura stramonium* (Solanaceae)

*Dregea volubilis* (Apocynaceae)

*Eclipta prostrata* (Asteraceae)

*Elettaria cardamomum* (Zingiberaceae)

*Eucalyptus globulus* (Myrtaceae)

*Euphorbia hirta* (Euphorbiaceae)

*Euphorbia neriifolia* (Euphorbiaceae)

*Ficus religiosa* (Moraceae)

*Fritillaria cirrhosa* (Liliaceae)

*Garuga pinnata* (Burseraceae)

*Justicia adhatoda* (Acanthaceae)

*Leucas cephalotes* (Lamiaceae)

*Mentha arvensis* (Lamiaceae)

*Mimosa pudica* (Fabaceae)

*Monochoria vaginalis* (Pontederiaceae)

*Morinda angustifolia* (Rubiaceae)

*Ocimum americanum* (Lamiaceae)

*Oroxylum indicum* (Bignoniaceae)

*Passiflora foetida* (Passifloraceae)

*Phyllanthus emblica* (Phyllanthaceae)

*Piper betle* (Piperaceae)

*Piper cubeba* (Piperaceae)

*Piper nigrum* (Piperaceae)

*Plumeria rubra* (Apocynaceae)

*Ricinus communis* (Euphorbiaceae)

*Rotheca serrata* (Lamiaceae)

*Senna alata* (Fabaceae)

*Solanum anguivi* (Solanaceae)

*Terminalia bellirica* (Combretaceae)

*Terminalia citrina* (Combretaceae)

*Trichosanthes tricuspidata* (Cucurbitaceae)

*Urena lobata* (Malvaceae)

*Volkameria inermis* (Lamiaceae)

*Zingiber montanum* (Zingiberaceae)

*Zingiber officinale* (Zingiberaceae)

**Astringent**

*Acacia catechu* (Fabaceae)

*Acacia leucophloea* (Fabaceae)

*Acacia nilotica* (Fabaceae)

*Acacia pennata* (Fabaceae)

*Agrimonia eupatoria* (Rosaceae)

*Alnus nepalensis* (Betulaceae)

*Aphanamixis polystachya* (Meliaceae)

*Artocarpus lakoocha* (Moraceae)

*Arundo donax* (Poaceae)

*Asclepias curassavica* (Apocynaceae)

*Bauhinia purpurea* (Fabaceae)

*Bixa orellana* (Bixaceae)

*Bombax ceiba* (Malvaceae)

*Bridelia retusa* (Phyllanthaceae)

*Butea monosperma* (Fabaceae)

*Caesalpinia pulcherrima* (Fabaceae)

*Ceiba pentandra* (Malvaceae)

*Chukrasia tabularis* (Meliaceae)

*Gloriosa superba* (Colchicaceae)

*Grewia asiatica* (Malvaceae)

*Ipomoea alba* (Convolvulaceae)

*Lagerstroemia speciosa* (Lythraceae)

*Lannea coromandelica* (Anacardiaceae)

*Leea macrophylla* (Vitaceae)

*Mangifera indica* (Anacardiaceae)

*Memecylon edule* (Melastomataceae)

*Mesua ferrea* (Calophyllaceae)

*Mimosa pudica* (Fabaceae)

*Momordica charantia* (Cucurbitaceae)

*Moringa oleifera* (Moringaceae)

*Oroxylum indicum* (Bignoniaceae)

*Phyllanthus emblica* (Phyllanthaceae)

*Phyllodium pulchellum* (Fabaceae)

*Pterospermum acerifolium* (Malvaceae)

*Punica granatum* (Lythraceae)

*Rauvolfia serpentina* (Apocynaceae)

*Rhus chinensis* (Anacardiaceae)

*Salvia officinalis* (Lamiaceae)

*Saraca indica* (Fabaceae)

*Schleichera oleosa* (Sapindaceae)

*Semecarpus anacardium* (Anacardiaceae)

*Senna alata* (Fabaceae)

*Senna auriculata* (Fabaceae)

*Senna sulfurea* (Fabaceae)

*Tectona grandis* (Lamiaceae)

*Terminalia catappa* (Combretaceae)

*Terminalia chebula* (Combretaceae)

*Terminalia tomentosa* (Combretaceae)

*Toona sureni* (Meliaceae)

*Vitex glabrata* (Lamiaceae)

*Xylia xylocarpa* (Fabaceae)

*Xylocarpus granatum* (Meliaceae)

*Xylocarpus moluccensis* (Meliaceae)

**Bad breath. *See* Halitosis.**

**Baldness**

*Eclipta prostrata* (Asteraceae)

**Beriberi**

*Alstonia scholaris* (Apocynaceae)

**Bile**

*Acacia pennata* (Fabaceae)

*Azadirachta indica* (Meliaceae)

*Benincasa hispida* (Cucurbitaceae)

*Calophyllum inophyllum* (Calophyllaceae)

*Carica papaya* (Caricaceae)

*Cinnamomum tamala* (Lauraceae)

*Citrus aurantiifolia* (Rutaceae)

*Coccinia grandis* (Crassulaceae)

*Coix lacryma-jobi* (Poaceae)

*Commelina paludosa* (Commelinaceae)

*Cuscuta reflexa* (Convolvulaceae)

*Ficus religiosa* (Moraceae)

*Flacourtia jangomas* (Salicaceae)

*Gossypium barbadense* (Malvaceae)

*Gossypium hirsutum* (Malvaceae)

*Holarrhena pubescens* (Apocynaceae)

*Justicia adhatoda* (Acanthaceae)

*Limonia acidissima* (Rutaceae)

*Magnolia champaca* (Magnoliaceae)

*Mimosa pudica* (Fabaceae)

*Momordica charantia* (Cucurbitaceae)

*Morinda angustifolia* (Rubiaceae)

*Ocimum americanum* (Lamiaceae)

*Plumbago zeylanica* (Plumbaginaceae)

*Ricinus communis* (Euphorbiaceae)

*Senna alexandrina* (Fabaceae)

*Senna sulfurea* (Fabaceae)

*Thunbergia erecta* (Acanthaceae)

*Tinospora cordifolia* (Menispermaceae)

*Urena lobata* (Malvaceae)

**Bites**

-cat

*Mentha arvensis* (Lamiaceae)

-insect

*Verbena officinalis* (Verbenaceae)

-rabid dog

*Momordica charantia* (Cucurbitaceae)

*Piper nigrum* (Piperaceae)

-snake

*Acacia concinna* (Fabaceae)

*Acanthus ilicifolius* (Acanthaceae)

*Careya arborea* (Lecythidaceae)

*Coptis teeta* (Ranunculaceae)

*Croton persimilis* (Euphorbiaceae)

*Gossypium barbadense* (Malvaceae)

*Gossypium hirsutum* (Malvaceae)

*Jasminum multiflorum* (Oleaceae)

*Mesua ferrea* (Calophyllaceae)

*Nerium oleander* (Apocynaceae)

*Zingiber montanum* (Zingiberaceae)

**Bitter**

*Arundo donax* (Poaceae)

*Azadirachta indica* (Meliaceae)

*Fritillaria cirrhosa* (Liliaceae)

*Gloriosa superba* (Colchicaceae)

*Hiptage benghalensis* (Malpighiceae)

*Senna sulfurea* (Fabaceae)

*Swertia chirayita* (Gentianaceae)

*Tinospora cordifolia* (Menispermaceae)

*Verbena officinalis* (Verbenaceae)

**Bladder disease**

*Allium sativum* (Amaryllidaceae)

*Amorphophallus paeoniifolius* (Araceae)

*Arundo donax* (Poaceae)

*Butea monosperma* (Fabaceae)

*Magnolia champaca* (Magnoliaceae)

*Orthosiphon aristatus* (Lamiaceae)

*Pogostemon cablin* (Lamiaceae)

*Ricinus communis* (Euphorbiaceae)

-stones

*Aloe vera* (Asphodelaceae)

*Curcuma longa* (Zingiberaceae)

*Mirabilis jalapa* (Nyctaginaceae)

**Bleeding**

*Acalypha indica* (Euphorbiaceae)

*Aquilaria malaccensis* (Thymelaeaceae)

*Benincasa hispida* (Cucurbitaceae)

*Butea monosperma* (Fabaceae)

*Cinnamomum tamala* (Lauraceae)

*Diospyros malabarica* (Ebenaceae)

*Ipomoea aquatica* (Convolvulaceae)

*Mucuna pruriens* (Fabaceae)

*Plantago major* (Plantaginaceae)

*Tanacetum cinerariifolium* (Asteraceae)

*Terminalia citrina* (Combretaceae)

*Terminalia chebula* (Combretaceae)

-ears

*Aegle marmelos* (Rutaceae)

-gums

*Acacia pennata* (Fabaceae)

*Aegle marmelos* (Rutaceae)

*Citrus aurantiifolia* (Rutaceae)

-in urine

*Piper longum* (Piperaceae)

*Pogostemon cablin* (Lamiaceae)

*Scoparia dulcis* (Plantaginaceae)

*Tadehagi triquetrum* (Fabaceae)

-menstrual

*Gossypium barbadense* (Malvaceae)

*Gossypium hirsutum* (Malvaceae)

*Mesua ferrea* (Calophyllaceae)

*Ipomoea aquatica* (Convolvulaceae)

-nosebleeds

*Amaranthus spinosus* (Amaranthaceae)

*Citrus aurantiifolia* (Rutaceae)

*Cordyline fruticosa* (Laxmanniaceae)

*Morinda angustifolia* (Rubiaceae)

*Piper nigrum* (Piperaceae)

*Solanum anguivi* (Solanaceae)

**Bloating**

*Acacia concinna* (Fabaceae)

*Acorus calamus* (Acoraceae)

*Boerhavia diffusa* (Nyctaginaceae)

*Cardiospermum halicacabum* (Sapindaceae)

*Citrus aurantiifolia* (Rutaceae)

*Curcuma longa* (Zingiberaceae)

*Eucalyptus globulus* (Myrtaceae)

*Gloriosa superba* (Colchicaceae)

*Plantago major* (Plantaginaceae)

*Plumbago indica* (Plumbaginaceae)

*Plumbago zeylanica* (Plumbaginaceae)

*Rotheca serrata* (Lamiaceae)

*Semecarpus anacardium* (Anacardiaceae)

*Syzygium cumini* (Myrtaceae)

*Tectona grandis* (Lamiaceae)

*Vitex trifolia* (Lamiaceae)

**Blood disorders**

*Acacia farnesiana* (Fabaceae)

*Barringtonia acutangula* (Lecythidaceae)

*Benincasa hispida* (Cucurbitaceae)

*Coccinia grandis* (Crassulaceae)

*Ficus religiosa* (Moraceae)

*Mesua ferrea* (Calophyllaceae)

-abnormal

*Clausena excavata* (Rutaceae)

-binding

*Calophyllum inophyllum* (Calophyllaceae)

-cleaning

*Ficus religiosa* (Moraceae)

*Sesbania grandiflora* (Fabaceae)

-clotting

*Croton persimilis* (Euphorbiaceae)

-irregulation

*Acacia pennata* (Fabaceae)

*Aristolochia indica* (Aristolochiaceae)

*Cuscuta reflexa* (Convolvulaceae)

*Terminalia bellirica* (Combretaceae)

-pressure

*Apium graveolens* (Apiaceae)

*Croton persimilis* (Euphorbiaceae)

*Curcuma comosa* (Zingiberaceae)

*Curcuma longa* (Zingiberaceae)

*Morinda angustifolia* (Rubiaceae)

*Moringa oleifera* (Moringaceae)

*Rauvolfia serpentina* (Apocynaceae)

-purification

*Acalypha indica* (Euphorbiaceae)

*Amaranthus cruentus* (Amaranthaceae)

*Amaranthus spinosus* (Amaranthaceae)

*Anneslea fragrans* (Pentaphylacaceae)

*Carica papaya* (Caricaceae)

*Citrus limon* (Rutaceae)

*Coix lacryma-jobi* (Poaceae)

*Curcuma longa* (Zingiberaceae)

*Dracaena angustifolia* (Asparagaceae)

*Hydnocarpus kurzii* (Achariaceae)

*Myristica fragrans* (Myristicaceae)

*Nymphaea rubra* (Nymphaeaceae)

*Piper cubeba* (Piperaceae)

*Plumbago zeylanica* (Plumbaginaceae)

*Terminalia chebula* (Combretaceae)

*Zingiber officinale* (Zingiberaceae)

*Ziziphus jujuba* (Rhamnaceae)

**Boils. *See also* Skin disorders, Skin sores.**

*Abelmoschus moschatus* (Malvaceae)

*Albizia lebbeck* (Fabaceae)

*Aloe vera* (Asphodelaceae)

*Alstonia scholaris* (Apocynaceae)

*Amaranthus spinosus* (Amaranthaceae)

*Azadirachta indica* (Meliaceae)

*Barleria prionitis* (Acanthaceae)

*Bryophyllum pinnatum* (Crassulaceae)

*Crateva religiosa* (Capparaceae)

*Croton persimilis* (Euphorbiaceae)

*Eclipta prostrata* (Asteraceae)

*Gossypium barbadense* (Malvaceae)

*Gossypium hirsutum* (Malvaceae)

*Heliotropium indicum* (Boraginaceae)

*Hydnocarpus kurzii* (Achariaceae)

*Magnolia champaca* (Magnoliaceae)

*Mesua ferrea* (Calophyllaceae)

*Moringa oleifera* (Moringaceae)

*Oroxylum indicum* (Bignoniaceae)

*Plumbago zeylanica* (Plumbaginaceae)

*Senecio densiflorus* (Asteraceae)

*Senna alata* (Fabaceae)

*Syzygium aromaticum* (Myrtaceae)

-breast

*Datura stramonium* (Solanaceae)

-genital

*Ficus religiosa* (Moraceae)

**Bone**

-broken/fractured

*Butea monosperma* (Fabaceae)

*Euonymus kachinensis* (Celastraceae)

*Jatropha multifida* (Euphorbiaceae)

*Mirabilis jalapa* (Nyctaginaceae)

-improvements

*Carica papaya* (Caricaceae)

**Bowels**

-complaints

*Aristolochia tagala* (Aristolochiaceae)

*Woodfordia fruticosa* (Lythraceae)

-disease

*Cymbopogon nardus* (Poaceae)

-loose bowel

*Nyctanthes arbor-tristis* (Oleaceae)

*Senna alata* (Fabaceae)

-movements

*Aegle marmelos* (Rutaceae)

*Allium sativum* (Amaryllidaceae)

*Alpinia officinarum* (Zingiberaceae)

*Andrographis paniculata* (Acanthaceae)

*Annona squamosa* (Annonaceae)

*Benincasa hispida* (Cucurbitaceae)

*Canna indica* (Cannaceae)

*Carica papaya* (Caricaceae)

*Cassia fistula* (Fabaceae)

*Centella asiatica* (Apiaceae)

*Coptis teeta* (Ranunculaceae)

*Cyperus scariosus* (Cyperaceae)

*Cordyline fruticosa* (Laxmanniaceae)

*Croton persimilis* (Euphorbiaceae)

*Cymbopogon nardus* (Poaceae)

*Dillenia indica* (Dilleniaceae)

*Dregea volubilis* (Apocynaceae)

*Leucaena leucocephala* (Fabaceae)

*Mentha arvensis* (Lamiaceae)

*Momordica charantia* (Cucurbitaceae)

*Oroxylum indicum* (Bignoniaceae)

*Piper betle* (Piperaceae)

*Plumeria rubra* (Apocynaceae)

*Ricinus communis* (Euphorbiaceae)

*Semecarpus anacardium* (Anacardiaceae)

*Terminalia bellirica* (Combretaceae)

*Terminalia citrina* (Combretaceae)

*Trichosanthes tricuspidata* (Cucurbitaceae)

*Vitex trifolia* (Lamiaceae)

*Zingiber officinale* (Zingiberaceae)

-sluggish

*Foeniculum vulgare* (Apiaceae)

**Brain**

*Vitex trifolia* (Lamiaceae)

**Breasts**

-drooping

*Urena lobata* (Malvaceae)

-problems

*Ficus religiosa* (Moraceae)

**Breathing**

-clear passages

*Acalypha indica* (Euphorbiaceae)

*Cardiospermum halicacabum* (Sapindaceae)

*Euphorbia hirta* (Euphorbiaceae)

*Senna alata* (Fabaceae)

**Bronchitis**

*Acacia pennata* (Fabaceae)

*Acalypha indica* (Euphorbiaceae)

*Aegle marmelos* (Rutaceae)

*Barleria prionitis* (Acanthaceae)

*Benincasa hispida* (Cucurbitaceae)

*Cardiospermum halicacabum* (Sapindaceae)

*Citrus limon* (Rutaceae)

*Clerodendrum indicum* (Lamiaceae)

*Coccinia grandis* (Crassulaceae)

*Coptis teeta* (Ranunculaceae)

*Coriandrum sativum* (Apiaceae)

*Curcuma longa* (Zingiberaceae)

*Eucalyptus globulus* (Myrtaceae)

*Euphorbia hirta* (Euphorbiaceae)

*Ficus religiosa* (Moraceae)

*Leucas cephalotes* (Lamiaceae)

*Limonia acidissima* (Rutaceae)

*Nyctanthes arbor-tristis* (Oleaceae)

*Oroxylum indicum* (Bignoniaceae)

*Phyllanthus emblica* (Phyllanthaceae)

*Piper cubeba* (Piperaceae)

*Piper nigrum* (Piperaceae)

*Rauvolfia serpentina* (Apocynaceae)

*Rotheca serrata* (Lamiaceae)

*Urena lobata* (Malvaceae)

*Zingiber officinale* (Zingiberaceae)

**Bruises**

*Bryophyllum pinnatum* (Crassulaceae)

*Curcuma longa* (Zingiberaceae)

**Bumps**

*Aegle marmelos* (Rutaceae)

*Azadirachta indica* (Meliaceae)

*Barleria prionitis* (Acanthaceae)

*Butea monosperma* (Fabaceae)

*Calotropis procera* (Apocynaceae)

*Citrus limon* (Rutaceae)

*Convolvulus arvensis* (Convolvulaceae)

*Mirabilis jalapa* (Nyctaginaceae)

**Burning sensation**

*Cyperus scariosus* (Cyperaceae)

*Ipomoea aquatica* (Convolvulaceae)

**Burns**

*Bryophyllum pinnatum* (Crassulaceae)

*Cascabela thevetia* (Apocynaceae)

*Eclipta prostrata* (Asteraceae)

*Eucalyptus globulus* (Myrtaceae)

*Ficus religiosa* (Moraceae)

*Gossypium barbadense* (Malvaceae)

*Gossypium hirsutum* (Malvaceae)

*Ipomoea aquatica* (Convolvulaceae)

*Phyllanthus emblica* (Phyllanthaceae)

*Rotheca serrata* (Lamiaceae)

*Santalum album* (Santalaceae)

*Terminalia citrina* (Combretaceae)

*Trachyspermum ammi* (Apiaceae)

*Tradescantia spathacea* (Commelinaceae)

-mouth

*Morinda angustifolia* (Rubiaceae)

*Mimusops elengi* (Sapotaceae)

**Cancer**

-skin

*Nerium oleander* (Apocynaceae)

-stomach

*Moringa oleifera* (Moringaceae)

-throat

*Plumbago indica* (Plumbaginaceae)

**Carminative**

*Anethum graveolens* (Apiaceae)

*Aquilaria malaccensis* (Thymelaeaceae)

*Ardisia humilis* (Primulaceae)

*Clausena excavata* (Rutaceae)

*Cymbopogon nardus* (Poaceae)

*Limonia acidissima* (Rutaceae)

*Nigella sativa* (Ranunculaceae)

*Ocimum tenuiflorum* (Lamiaceae)

*Premna serratifolia* (Lamiaceae)

*Rauvolfia serpentina* (Apocynaceae)

*Salvia officinalis* (Lamiaceae)

*Selinum wallichianum* (Apiaceae)

*Solanum anguivi* (Solanaceae)

*Syzygium aromaticum* (Myrtaceae)

*Terminalia chebula* (Combretaceae)

*Zingiber zerumbet* (Zingiberaceae)

**Cataracts**

*Cardiospermum halicacabum* (Sapindaceae)

*Clitoria ternatea* (Fabaceae)

*Strychnos potatorum* (Loganiaceae)

*Syzygium aromaticum* (Myrtaceae)

**Catarrh**

*Cordia dichotoma* (Boraginaceae)

-bladder

*Cardiospermum halicacabum* (Sapindaceae)

-urinary tract

*Cardiospermum halicacabum* (Sapindaceae)

**Cathartic. *See also* Hydragogue, Laxative, Purgative.**

*Allamanda cathartica* (Apocynaceae)

*Momordica charantia* (Cucurbitaceae)

**Cerebral Palsy**

*Alstonia scholaris* (Apocynaceae)

**Chest**

*Acacia catechu* (Fabaceae)

*Rauvolfia serpentina* (Apocynaceae)

*Zingiber officinale* (Zingiberaceae)

*Ziziphus jujuba* (Rhamnaceae)

**Chickenpox**

*Eclipta prostrata* (Asteraceae)

**Childbirth**

*Amaranthus spinosus* (Amaranthaceae)

*Aquilaria malaccensis* (Thymelaeaceae)

*Aristolochia indica* (Aristolochiaceae)

*Basella alba* (Basellaceae)

*Cratoxylum formosum* (Hypericaceae)

*Euphorbia hirta* (Euphorbiaceae)

*Gloriosa superba* (Colchicaceae)

*Leucaena leucocephala* (Fabaceae)

*Momordica cochinchinensis* (Cucurbitaceae)

*Premna amplectens* (Lamiaceae)

*Premna mollissima* (Lamiaceae)

*Ricinus communis* (Euphorbiaceae)

*Triumfetta rhomboidea* (Malvaceae)

*Vitex trifolia* (Lamiaceae)

*Volkameria inermis* (Lamiaceae)

**Chills**

*Cardiospermum halicacabum* (Sapindaceae)

*Momordica charantia* (Cucurbitaceae)

**Cholagogue. *See also* Bile.**

*Tinospora cordifolia* (Menispermaceae)

**Cholera**

*Artabotrys hexapetalus* (Annonaceae)

*Bryophyllum pinnatum* (Crassulaceae)

*Calotropis procera* (Apocynaceae)

*Capparis zeylanica* (Capparaceae)

*Cymbopogon citratus* (Poaceae)

*Holarrhena pubescens* (Apocynaceae)

*Momordica charantia* (Cucurbitaceae)

*Mucuna pruriens* (Fabaceae)

*Myristica fragrans* (Myristicaceae)

*Oroxylum indicum* (Bignoniaceae)

*Tamarindus indica* (Fabaceae)

*Xylocarpus granatum* (Meliaceae)

*Xylocarpus moluccensis* (Meliaceae)

*Zingiber officinale* (Zingiberaceae)

**Chronic diseases**

*Trichosanthes tricuspidata* (Cucurbitaceae)

**Circulation**

*Alstonia scholaris* (Apocynaceae)

*Anethum graveolens* (Apiaceae)

*Apium graveolens* (Apiaceae)

*Aristolochia indica* (Aristolochiaceae)

*Clerodendrum indicum* (Lamiaceae)

*Eclipta prostrata* (Asteraceae)

*Phyllanthus emblica* (Phyllanthaceae)

*Piper nigrum* (Piperaceae)

-problems

*Acacia concinna* (Fabaceae)

*Allium sativum* (Amaryllidaceae)

*Alpinia galanga* (Zingiberaceae)

*Arundo donax* (Poaceae)

*Coix lacryma-jobi* (Poaceae)

*Curcuma comosa* (Zingiberaceae)

*Curcuma longa* (Zingiberaceae)

*Cymbopogon citratus* (Poaceae)

*Foeniculum vulgare* (Apiaceae)

*Fritillaria cirrhosa* (Liliaceae)

*Syzygium aromaticum* (Myrtaceae)

*Vitex trifolia* (Lamiaceae)

*Zingiber montanum* (Zingiberaceae)

-varicose

*Cardiospermum halicacabum* (Sapindaceae)

*Cycas rumphii* (Cycadaceae)

*Vitex trifolia* (Lamiaceae)

**Colds**

*Aristolochia indica* (Aristolochiaceae)

*Bauhinia acuminata* (Fabaceae)

*Blumea balsamifera* (Asteraceae)

*Centella asiatica* (Apiaceae)

*Curcuma longa* (Zingiberaceae)

*Cymbopogon nardus* (Poaceae)

*Eclipta prostrata* (Asteraceae)

*Eucalyptus globulus* (Myrtaceae)

*Euphorbia hirta* (Euphorbiaceae)

*Piper cubeba* (Piperaceae)

*Rotheca serrata* (Lamiaceae)

*Sesbania grandiflora* (Fabaceae)

*Sinapis alba* (Brassicaceae)

*Vitex trifolia* (Lamiaceae)

*Zingiber montanum* (Zingiberaceae)

*Zingiber officinale* (Zingiberaceae)

**Colic**

*Cassia fistula* (Fabaceae)

*Fagopyrum esculentum* (Polygonaceae)

*Foeniculum vulgare* (Apiaceae)

*Ipomoea pes-caprae* (Convolvulaceae)

*Magnolia champaca* (Magnoliaceae)

*Myristica fragrans* (Myristicaceae)

*Rhus chinensis* (Anacardiaceae)

**Complexion**

*Aloe vera* (Asphodelaceae)

*Canna indica* (Cannaceae)

*Cordyline fruticosa* (Laxmanniaceae)

*Ficus religiosa* (Moraceae)

*Mesua ferrea* (Calophyllaceae)

**Congestion**

*Anethum graveolens* (Apiaceae)

*Claoxylon indicum* (Euphorbiaceae)

*Eclipta prostrata* (Asteraceae)

*Ficus religiosa* (Moraceae)

*Ocimum americanum* (Lamiaceae)

*Verbena officinalis* (Verbenaceae)

**Constipation**

*Acacia concinna* (Fabaceae)

*Acalypha indica* (Euphorbiaceae)

*Acorus calamus* (Acoraceae)

*Aegle marmelos* (Rutaceae)

*Calophyllum inophyllum* (Calophyllaceae)

*Cassia fistula* (Fabaceae)

*Cinnamomum bejolghota* (Lauraceae)

*Coptis teeta* (Ranunculaceae)

*Cyperus scariosus* (Cyperaceae)

*Eucalyptus globulus* (Myrtaceae)

*Sesbania grandiflora* (Fabaceae)

*Terminalia bellirica* (Combretaceae)

**Contusions. *See* Bruises, Bumps.**

**Cooling**

*Azadirachta indica* (Meliaceae)

*Cinnamomum bejolghota* (Lauraceae)

*Cissampelos pareira* (Menispermaceae)

*Coccinia grandis* (Crassulaceae)

*Cordia dichotoma* (Boraginaceae)

*Cordyline fruticosa* (Laxmanniaceae)

*Cyperus scariosus* (Cyperaceae)

*Ficus religiosa* (Moraceae)

*Gossypium barbadense* (Malvaceae)

*Gossypium hirsutum* (Malvaceae)

*Mansonia gagei* (Malvaceae)

*Monochoria vaginalis* (Pontederiaceae)

*Phyllanthus emblica* (Phyllanthaceae)

*Senna sulfurea* (Fabaceae)

*Verbena officinalis* (Verbenaceae)

**Coughs**

*Acacia pennata* (Fabaceae)

*Acorus calamus* (Acoraceae)

*Aegle marmelos* (Rutaceae)

*Albizia odoratissima* (Fabaceae)

*Allium cepa* (Amaryllidaceae)

*Allium sativum* (Amaryllidaceae)

*Alpinia galanga* (Zingiberaceae)

*Apium graveolens* (Apiaceae)

*Aquilaria malaccensis* (Thymelaeaceae)

*Aristolochia indica* (Aristolochiaceae)

*Barleria prionitis* (Acanthaceae)

*Bauhinia acuminata* (Fabaceae)

*Benincasa hispida* (Cucurbitaceae)

*Boerhavia diffusa* (Nyctaginaceae)

*Centella asiatica* (Apiaceae)

*Cinnamomum tamala* (Lauraceae)

*Citrus aurantiifolia* (Rutaceae)

*Citrus limon* (Rutaceae)

*Clerodendrum indicum* (Lamiaceae)

*Clitoria ternatea* (Fabaceae)

*Convolvulus arvensis* (Convolvulaceae)

*Coptis teeta* (Ranunculaceae)

*Curcuma comosa* (Zingiberaceae)

*Curcuma longa* (Zingiberaceae)

*Cymbopogon citratus* (Poaceae)

*Cymbopogon nardus* (Poaceae)

*Dillenia indica* (Dilleniaceae)

*Eclipta prostrata* (Asteraceae)

*Elettaria cardamomum* (Zingiberaceae)

*Enydra fluctuans* (Asteraceae)

*Euphorbia hirta* (Euphorbiaceae)

*Ficus religiosa* (Moraceae)

*Foeniculum vulgare* (Apiaceae)

*Fritillaria cirrhosa* (Liliaceae)

*Indigofera cassioides* (Fabaceae)

*Justicia adhatoda* (Acanthaceae)

*Leucas cephalotes* (Lamiaceae)

*Magnolia champaca* (Magnoliaceae)

*Mentha arvensis* (Lamiaceae)

*Mesua ferrea* (Calophyllaceae)

*Mimusops elengi* (Sapotaceae)

*Morinda angustifolia* (Rubiaceae)

*Ocimum americanum* (Lamiaceae)

*Phyllanthus emblica* (Phyllanthaceae)

*Piper betle* (Piperaceae)

*Piper cubeba* (Piperaceae)

*Piper nigrum* (Piperaceae)

*Ricinus communis* (Euphorbiaceae)

*Rotheca serrata* (Lamiaceae)

*Selinum wallichianum* (Apiaceae)

*Semecarpus anacardium* (Anacardiaceae)

*Senna alexandrina* (Fabaceae)

*Sinapis alba* (Brassicaceae)

*Terminalia bellirica* (Combretaceae)

*Tradescantia spathacea* (Commelinaceae)

*Trichosanthes tricuspidata* (Cucurbitaceae)

*Vitex trifolia* (Lamiaceae)

*Zingiber montanum* (Zingiberaceae)

*Zingiber officinale* (Zingiberaceae)

**Cuts**

*Mimusops elengi* (Sapotaceae)

**Cysts**

*Convolvulus arvensis* (Convolvulaceae)

*Curcuma longa* (Zingiberaceae)

*Eclipta prostrata* (Asteraceae)

*Fritillaria cirrhosa* (Liliaceae)

*Magnolia champaca* (Magnoliaceae)

*Rotheca serrata* (Lamiaceae)

*Sinapis alba* (Brassicaceae)

**Deafness**

*Allium sativum* (Amaryllidaceae)

**Demulcent**

*Azadirachta indica* (Meliaceae)

*Grewia asiatica* (Malvaceae)

**Deterioration**

*Terminalia bellirica* (Combretaceae)

**Diabetes**

*Aeginetia indica* (Orobanchaceae)

*Anacardium occidentale* (Anacardiaceae)

*Archidendron jiringa* (Fabaceae)

*Bombax ceiba* (Malvaceae)

*Catharanthus roseus* (Apocynaceae)

*Ceiba pentandra* (Malvaceae)

*Centella asiatica* (Apiaceae)

*Combretum indicum* (Combretaceae)

*Crateva religiosa* (Capparaceae)

*Ficus hispida* (Moraceae)

*Heliotropium indicum* (Boraginaceae)

*Lagerstroemia speciosa* (Lythraceae)

*Momordica charantia* (Cucurbitaceae)

*Morinda angustifolia* (Rubiaceae)

*Moringa oleifera* (Moringaceae)

*Orthosiphon aristatus* (Lamiaceae)

*Phyllanthus emblica* (Phyllanthaceae)

*Plantago major* (Plantaginaceae)

*Premna serratifolia* (Lamiaceae)

*Psidium guajava* (Myrtaceae)

*Putranjiva roxburghii* (Putranjavaceae)

*Scoparia dulcis* (Plantaginaceae)

*Sesbania grandiflora* (Fabaceae)

*Sesbania sesban* (Fabaceae)

*Solanum rudepannum* (Solanaceae)

*Syzygium cumini* (Myrtaceae)

*Syzygium jambos* (Myrtaceae)

**Diaphoretic**

*Cardiospermum halicacabum* (Sapindaceae)

*Cinnamomum camphora* (Lauraceae)

*Cymbopogon nardus* (Poaceae)

*Flacourtia jangomas* (Salicaceae)

*Lantana × aculeata* (Verbenaceae)

*Ocimum tenuiflorum* (Lamiaceae)

*Phragmites karka* (Poaceae)

*Salvia officinalis* (Lamiaceae)

*Sida spinosa* (Malvaceae)

*Smilax aspera* (Smilacaceae)

*Smilax guianensis* (Smilacaceae)

**Diarrhea**

*Acacia concinna* (Fabaceae)

*Acalypha indica* (Euphorbiaceae)

*Aegle marmelos* (Rutaceae)

*Allium cepa* (Amaryllidaceae)

*Alysicarpus vaginalis* (Fabaceae)

*Annona squamosa* (Annonaceae)

*Artocarpus heterophyllus* (Moraceae)

*Barringtonia acutangula* (Lecythidaceae)

*Basella alba* (Basellaceae)

*Butea monosperma* (Fabaceae)

*Canna indica* (Cannaceae)

*Carica papaya* (Caricaceae)

*Casuarina equisetifolia* (Casuarinaceae)

*Chenopodium album* (Amaranthaceae)

*Chukrasia tabularis* (Meliaceae)

*Cinnamomum bejolghota* (Lauraceae)

*Cinnamomum tamala* (Lauraceae)

*Cinnamomum verum* (Lauraceae)

*Coccinia grandis* (Crassulaceae)

*Combretum indicum* (Combretaceae)

*Coptis teeta* (Ranunculaceae)

*Cordyline fruticosa* (Laxmanniaceae)

*Croton persimilis* (Euphorbiaceae)

*Curcuma longa* (Zingiberaceae)

*Cyperus scariosus* (Cyperaceae)

*Diospyros malabarica* (Ebenaceae)

*Eclipta prostrata* (Asteraceae)

*Fagopyrum esculentum* (Polygonaceae)

*Foeniculum vulgare* (Apiaceae)

*Garcinia × mangostana* (Clusiaceae)

*Garcinia xanthochymus* (Clusiaceae)

*Gossypium barbadense* (Malvaceae)

*Gossypium hirsutum* (Malvaceae)

*Ipomoea aquatica* (Convolvulaceae)

*Justicia adhatoda* (Acanthaceae)

*Melastoma malabathricum* (Melastomataceae)

*Mimusops elengi* (Sapotaceae)

*Morinda angustifolia* (Rubiaceae)

*Myristica fragrans* (Myristicaceae)

*Nyctanthes arbor-tristis* (Oleaceae)

*Oroxylum indicum* (Bignoniaceae)

*Phyllanthus emblica* (Phyllanthaceae)

*Piper betle* (Piperaceae)

*Piper nigrum* (Piperaceae)

*Plumbago zeylanica* (Plumbaginaceae)

*Santalum album* (Santalaceae)

*Streblus asper* (Moraceae)

*Strychnos potatorum* (Loganiaceae)

*Syzygium cumini* (Myrtaceae)

*Tamarindus indica* (Fabaceae)

*Tectona grandis* (Lamiaceae)

*Terminalia bellirica* (Combretaceae)

*Terminalia chebula* (Combretaceae)

*Terminalia citrina* (Combretaceae)

*Terminalia tomentosa* (Combretaceae)

*Trachyspermum ammi* (Apiaceae)

*Walsura pinnata* (Meliaceae)

*Xylocarpus granatum* (Meliaceae)

*Zingiber montanum* (Zingiberaceae)

*Ziziphus jujuba* (Rhamnaceae)

**Digestion**

*Acacia concinna* (Fabaceae)

*Alpinia galanga* (Zingiberaceae)

*Andrographis paniculata* (Acanthaceae)

*Annona squamosa* (Annonaceae)

*Apium graveolens* (Apiaceae)

*Arundo donax* (Poaceae)

*Azadirachta indica* (Meliaceae)

*Butea monosperma* (Fabaceae)

*Canavalia ensiformis* (Fabaceae)

*Carica papaya* (Caricaceae)

*Carthamus tinctorius* (Asteraceae)

*Catharanthus roseus* (Apocynaceae)

*Cinnamomum verum* (Lauraceae)

*Citrus aurantiifolia* (Rutaceae)

Citrus × aurantium (Rutaceae)

*Citrus limon* (Rutaceae)

*Clausena excavata* (Rutaceae)

*Coix lacryma-jobi* (Poaceae)

*Coptis teeta* (Ranunculaceae)

*Coriandrum sativum* (Apiaceae)

*Curcuma comosa* (Zingiberaceae)

*Curcuma longa* (Zingiberaceae)

*Cymbopogon citratus* (Poaceae)

*Euphorbia hirta* (Euphorbiaceae)

*Foeniculum vulgare* (Apiaceae)

*Holarrhena pubescens* (Apocynaceae)

*Mentha arvensis* (Lamiaceae)

*Mesua ferrea* (Calophyllaceae)

*Momordica charantia* (Cucurbitaceae)

*Monochoria vaginalis* (Pontederiaceae)

*Moringa oleifera* (Moringaceae)

*Myristica fragrans* (Myristicaceae)

*Ocimum americanum* (Lamiaceae)

*Phyllanthus emblica* (Phyllanthaceae)

*Piper cubeba* (Piperaceae)

*Piper longum* (Piperaceae)

*Piper nigrum* (Piperaceae)

*Pithecellobium dulce* (Fabaceae)

*Plumbago indica* (Plumbaginaceae)

*Plumbago zeylanica* (Plumbaginaceae)

*Plumeria rubra* (Apocynaceae)

*Portulaca oleracea* (Portulaceae)

*Rauvolfia serpentina* (Apocynaceae)

*Rotheca serrata* (Lamiaceae)

*Semecarpus anacardium* (Anacardiaceae)

*Sinapis alba* (Brassicaceae)

*Syzygium aromaticum* (Myrtaceae)

*Tinospora cordifolia* (Menispermaceae)

*Trachyspermum ammi* (Apiaceae)

*Zingiber officinale* (Zingiberaceae)

**Diphtheria**

*Carica papaya* (Caricaceae)

*Cinnamomum bejolghota* (Lauraceae)

*Rotheca serrata* (Lamiaceae)

**Disease, prevention**

*Citrus aurantiifolia* (Rutaceae)

**Diuretic**

*Abelmoschus moschatus* (Malvaceae)

*Agrimonia eupatoria* (Rosaceae)

*Allium cepa* (Amaryllidaceae)

*Amaranthus cruentus* (Amaranthaceae)

*Amaranthus spinosus* (Amaranthaceae)

*Asparagus filicinus* (Asparagaceae)

*Arundo donax* (Poaceae)

*Barleria prionitis* (Acanthaceae)

*Berberis nepalensis* (Berberidaceae)

*Bombax ceiba* (Malvaceae)

*Brassica oleracea* (Brassicaceae)

*Cardiospermum halicacabum* (Sapindaceae)

*Carthamus tinctorius* (Asteraceae)

*Ceiba pentandra* (Malvaceae)

*Centella asiatica* (Apiaceae)

*Cissampelos pareira* (Menispermaceae)

*Clitoria ternatea* (Fabaceae)

*Coix lacryma-jobi* (Poaceae)

*Commelina paludosa* (Commelinaceae)

*Cordia dichotoma* (Boraginaceae)

*Cucumis sativus* (Cucurbitaceae)

*Cullen corylifolium* (Fabaceae)

*Curcuma comosa* (Zingiberaceae)

*Daucus carota* (Apiaceae)

*Heliotropium indicum* (Boraginaceae)

*Hygrophila auriculata* (Acanthaceae)

*Magnolia champaca* (Magnoliaceae)

*Manilkara zapota* (Sapotaceae)

*Mimosa pudica* (Fabaceae)

*Ocimum americanum* (Lamiaceae)

*Orthosiphon aristatus* (Lamiaceae)

*Phragmites karka* (Poaceae)

*Phyllanthus emblica* (Phyllanthaceae)

*Phyllanthus niruri* (Phyllanthaceae)

*Physalis peruviana* (Solanaceae)

*Pogostemon cablin* (Lamiaceae)

*Sambucus javanica* (Adoxaceae)

*Sesamum indicum* (Pedaliaceae)

*Terminalia tomentosa* (Combretaceae)

*Urena lobata* (Malvaceae)

*Urtica dioica* (Urticaceae)

*Zingiber officinale* (Zingiberaceae)

**Dizziness**

*Andrographis paniculata* (Acanthaceae)

*Annona squamosa* (Annonaceae)

*Aquilaria malaccensis* (Thymelaeaceae)

*Aristolochia indica* (Aristolochiaceae)

*Azadirachta indica* (Meliaceae)

*Cinnamomum camphora* (Lauraceae)

*Citrus aurantiifolia* (Rutaceae)

*Citrus limon* (Rutaceae)

*Commelina paludosa* (Commelinaceae)

*Mentha arvensis* (Lamiaceae)

*Millingtonia hortensis* (Bignoniaceae)

*Scoparia dulcis* (Plantaginaceae)

**Dropsy**

*Apium graveolens* (Apiaceae)

*Barleria prionitis* (Acanthaceae)

*Boerhavia diffusa* (Nyctaginaceae)

*Canna indica* (Cannaceae)

*Hygrophila auriculata* (Acanthaceae)

*Moringa oleifera* (Moringaceae)

*Pavetta indica* (Rubiaceae)

*Senna alexandrina* (Fabaceae)

**Dysentery**

*Albizia lebbeck* (Fabaceae)

*Allium cepa* (Amaryllidaceae)

*Alpinia galanga* (Zingiberaceae)

*Alstonia scholaris* (Apocynaceae)

*Alysicarpus vaginalis* (Fabaceae)

*Andrographis paniculata* (Acanthaceae)

*Annona squamosa* (Annonaceae)

*Aristolochia indica* (Aristolochiaceae)

*Arundo donax* (Poaceae)

*Asclepias curassavica* (Apocynaceae)

*Barringtonia acutangula* (Lecythidaceae)

*Bryophyllum pinnatum* (Crassulaceae)

*Calotropis gigantea* (Apocynaceae)

*Casuarina equisetifolia* (Casuarinaceae)

*Centella asiatica* (Apiaceae)

*Chromolaena odorata* (Asteraceae)

*Coix lacryma-jobi* (Poaceae)

*Combretum indicum* (Combretaceae)

*Cordyline fruticosa* (Laxmanniaceae)

*Croton persimilis* (Euphorbiaceae)

*Curcuma comosa* (Zingiberaceae)

*Curcuma longa* (Zingiberaceae)

*Diospyros malabarica* (Ebenaceae)

*Erythrina variegata* (Fabaceae)

*Euphorbia hirta* (Euphorbiaceae)

*Garcinia × mangostana* (Clusiaceae)

*Garcinia xanthochymus* (Clusiaceae)

*Grewia polygama* (Malvaceae)

*Holarrhena pubescens* (Apocynaceae)

*Ipomoea aquatica* (Convolvulaceae)

*Justicia adhatoda* (Acanthaceae)

*Kaempferia elegans* (Zingiberaceae)

*Kleinhovia hospita* (Malvaceae)

*Melastoma malabathricum* (Melastomataceae)

*Morinda coreia* (Rubiaceae)

*Mussaenda macrophylla* (Rubiaceae)

*Mucuna pruriens* (Fabaceae)

*Oroxylum indicum* (Bignoniaceae)

*Phyllanthus emblica* (Phyllanthaceae)

*Plumbago zeylanica* (Plumbaginaceae)

*Ricinus communis* (Euphorbiaceae)

*Sandoricum koetjape* (Meliaceae)

*Senna sulfurea* (Fabaceae)

*Spondias pinnata* (Anacardiaceae)

*Streblus asper* (Moraceae)

*Strychnos potatorum* (Loganiaceae)

*Syzygium cumini* (Myrtaceae)

*Tadehagi triquetrum* (Fabaceae)

*Tamarindus indica* (Fabaceae)

*Tamilnadia uliginosa* (Rubiaceae)

*Terminalia catappa* (Combretaceae)

*Terminalia chebula* (Combretaceae)

*Trachyspermum ammi* (Apiaceae)

*Tradescantia spathacea* (Commelinaceae)

*Vitex trifolia* (Lamiaceae)

*Walsura pinnata* (Meliaceae)

*Xylocarpus granatum* (Meliaceae)

*Xylocarpus moluccensis* (Meliaceae)

-male-related

*Myristica fragrans* (Myristicaceae)

**Dyspepsia**

*Datura stramonium* (Solanaceae)

*Leucas cephalotes* (Lamiaceae)

*Spondias pinnata* (Anacardiaceae)

**Earaches**

*Acalypha indica* (Euphorbiaceae)

*Aloe vera* (Asphodelaceae)

*Alstonia scholaris* (Apocynaceae)

*Calotropis procera* (Apocynaceae)

*Cinnamomum tamala* (Lauraceae)

*Holarrhena pubescens* (Apocynaceae)

*Moringa oleifera* (Moringaceae)

*Nerium oleander* (Apocynaceae)

*Plantago major* (Plantaginaceae)

*Sinapis alba* (Brassicaceae)

*Tadehagi triquetrum* (Fabaceae)

*Tamarindus indica* (Fabaceae)

*Tinospora cordifolia* (Menispermaceae)

*Vitex trifolia* (Lamiaceae)

*Zingiber officinale* (Zingiberaceae)

**Ear diseases/infections**

*Aquilaria malaccensis* (Thymelaeaceae)

*Croton tiglium* (Euphorbiaceae)

*Gloriosa superba* (Colchicaceae)

*Holarrhena pubescens* (Apocynaceae)

*Moringa oleifera* (Moringaceae)

*Plantago major* (Plantaginaceae)

*Tadehagi triquetrum* (Fabaceae)

*Vitex trifolia* (Lamiaceae)

-buzzing

*Carica papaya* (Caricaceae)

**Eczema**

*Allium sativum* (Amaryllidaceae)

*Cardiospermum halicacabum* (Sapindaceae)

*Mesua ferrea* (Calophyllaceae)

*Ocimum americanum* (Lamiaceae)

*Plumbago zeylanica* (Plumbaginaceae)

*Senna alata* (Fabaceae)

**Edema**

*Aegle marmelos* (Rutaceae)

*Aloe vera* (Asphodelaceae)

*Alysicarpus vaginalis* (Fabaceae)

*Andrographis paniculata* (Acanthaceae)

*Argemone mexicana* (Papaveraceae)

*Aristolochia indica* (Aristolochiaceae)

*Blumea balsamifera* (Asteraceae)

*Cardiospermum halicacabum* (Sapindaceae)

*Clitoria ternatea* (Fabaceae)

*Crinum asiaticum* (Amaryllidaceae)

*Canna indica* (Cannaceae)

*Coptis teeta* (Ranunculaceae)

*Croton persimilis* (Euphorbiaceae)

*Curcuma longa* (Zingiberaceae)

*Enydra fluctuans* (Asteraceae)

*Ipomoea alba* (Convolvulaceae)

*Leucaena leucocephala* (Fabaceae)

*Limonia acidissima* (Rutaceae)

*Mesua ferrea* (Calophyllaceae)

*Mimosa pudica* (Fabaceae)

*Momordica charantia* (Cucurbitaceae)

*Moringa oleifera* (Moringaceae)

*Mucuna pruriens* (Fabaceae)

*Oroxylum indicum* (Bignoniaceae)

*Plumbago indica* (Plumbaginaceae)

*Ricinus communis* (Euphorbiaceae)

*Rotheca serrata* (Lamiaceae)

*Senna alexandrina* (Fabaceae)

*Sesbania sesban* (Fabaceae)

*Sinapis alba* (Brassicaceae)

*Tectona grandis* (Lamiaceae)

*Terminalia bellirica* (Combretaceae)

*Urena lobata* (Malvaceae)

*Vitex trifolia* (Lamiaceae)

**Elephantiasis**

*Ficus religiosa* (Moraceae)

*Mucuna pruriens* (Fabaceae)

*Strychnos wallichiana* (Loganiaceae)

**Embrocation. See Liniment.**

**Emetic**

*Abrus precatorius* (Fabaceae)

*Acalypha indica* (Euphorbiaceae)

*Achyranthes aspera* (Amaranthaceae)

*Asclepias curassavica* (Apocynaceae)

*Cardiospermum halicacabum* (Sapindaceae)

*Catunaregam spinosa* (Rubiaceae)

*Dregea volubilis* (Apocynaceae)

*Entada phaseoloides* (Fabaceae)

*Ipomoea pes-caprae* (Convolvulaceae)

*Momordica charantia* (Cucurbitaceae)

*Phyllanthus acidus* (Phyllanthaceae)

*Smilax aspera* (Smilacaceae)

*Smilax guianensis* (Smilacaceae)

*Vitex trifolia* (Lamiaceae)

**Emmenagogue**

*Caesalpinia pulcherrima* (Fabaceae)

*Morinda citrifolia* (Rubiaceae)

*Mucuna pruriens* (Fabaceae)

*Sesamum indicum* (Pedaliaceae)

*Syzygium aromaticum* (Myrtaceae)

*Taxus baccata* (Taxaceae)

*Tabernaemontana divaricata* (Apocynaceae)

**Emollient**

*Abelmoschus esculentus* (Malvaceae)

*Arachis hypogaea* (Fabaceae)

*Hibiscus sabdariffa* (Malvaceae)

*Hibiscus schizopetalus* (Malvaceae)

*Hibiscus vitifolius* (Malvaceae)

*Malvastrum coromandelianum* (Malvaceae)

*Senecio densiflorus* (Asteraceae)

*Sesamum indicum* (Pedaliaceae)

**Energy, low**

*Urena lobata* (Malvaceae)

**Epilepsy**

*Acorus calamus* (Acoraceae)

*Annona squamosa* (Annonaceae)

*Benincasa hispida* (Cucurbitaceae)

*Calotropis procera* (Apocynaceae)

*Citrus limon* (Rutaceae)

*Colebrookea oppositifolia* (Lamiaceae)

*Cymbopogon nardus* (Poaceae)

*Dillenia indica* (Dilleniaceae)

*Flemingia strobilifera* (Fabaceae)

*Limonia acidissima* (Rutaceae)

*Sapindus saponaria* (Sapindaceae)

*Sesbania grandiflora* (Fabaceae)

*Strychnos wallichiana* (Loganiaceae)

**Erysipelas**

*Bryophyllum pinnatum* (Crassulaceae)

*Eucalyptus globulus* (Myrtaceae)

*Plantago major* (Plantaginaceae)

*Quassia indica* (Simaroubaceae)

*Tadehagi triquetrum* (Fabaceae)

**Excretory**

*Annona squamosa* (Annonaceae)

*Plumeria rubra* (Apocynaceae)

**Exhaustion**

*Acalypha indica* (Euphorbiaceae)

**Expectorant**

*Abrus precatorius* (Fabaceae)

*Acalypha indica* (Euphorbiaceae)

*Allium cepa* (Amaryllidaceae)

*Argemone mexicana* (Papaveraceae)

*Blumea balsamifera* (Asteraceae)

*Coccinia grandis* (Crassulaceae)

*Cordia dichotoma* (Boraginaceae)

*Dregea volubilis* (Apocynaceae)

*Hygrophila auriculata* (Acanthaceae)

*Ipomoea aquatica* (Convolvulaceae)

*Malvastrum coromandelianum* (Malvaceae)

*Ocimum tenuiflorum* (Lamiaceae)

*Plumbago indica* (Plumbaginaceae)

*Sesamum indicum* (Pedaliaceae)

*Urena lobata* (Malvaceae)

**Eye**

-disease

*Cardiospermum halicacabum* (Sapindaceae)

*Cinnamomum verum* (Lauraceae)

*Ipomoea alba* (Convolvulaceae)

*Magnolia champaca* (Magnoliaceae)

*Momordica charantia* (Cucurbitaceae)

*Nyctanthes arbor-tristis* (Oleaceae)

*Ocimum americanum* (Lamiaceae)

*Phyllodium pulchellum* (Fabaceae)

*Plumbago indica* (Plumbaginaceae)

*Sesbania grandiflora* (Fabaceae)

*Terminalia bellirica* (Combretaceae)

-health

*Allium sativum* (Amaryllidaceae)

*Aloe vera* (Asphodelaceae)

*Cananga odorata* (Annonaceae)

*Carthamus tinctorius* (Asteraceae)

*Centella asiatica* (Apiaceae)

*Coptis teeta* (Ranunculaceae)

*Curcuma longa* (Zingiberaceae)

*Eclipta prostrata* (Asteraceae)

*Hygrophila phlomoides* (Acanthaceae)

*Justicia adhatoda* (Acanthaceae)

*Rauvolfia serpentina* (Apocynaceae)

*Thunbergia laurifolia* (Acanthaceae)

-infection

*Aquilaria malaccensis* (Thymelaeaceae)

*Bryophyllum pinnatum* (Crassulaceae)

*Carallia brachiata* (Rhizophoraceae)

*Cascabela thevetia* (Apocynaceae)

*Emilia sonchifolia* (Asteraceae)

*Foeniculum vulgare* (Apiaceae)

*Phyllanthus emblica* (Phyllanthaceae)

*Sesbania sesban* (Fabaceae)

*Strychnos potatorum* (Loganiaceae)

*Terminalia bellirica* (Combretaceae)

-wash

*Azadirachta indica* (Meliaceae)

**Fatigue**

*Acacia concinna* (Fabaceae)

*Acalypha indica* (Euphorbiaceae)

*Boerhavia diffusa* (Nyctaginaceae)

*Butea monosperma* (Fabaceae)

*Cardiospermum halicacabum* (Sapindaceae)

*Cinnamomum bejolghota* (Lauraceae)

*Citrus aurantiifolia* (Rutaceae)

*Citrus limon* (Rutaceae)

*Dillenia indica* (Dilleniaceae)

*Oroxylum indicum* (Bignoniaceae)

*Santalum album* (Santalaceae)

*Syzygium aromaticum* (Myrtaceae)

-asthma-related

*Euphorbia hirta* (Euphorbiaceae)

-childbirth-related

*Ocimum americanum* (Lamiaceae)

**Febrifuge. *See* Antipyretic.**

**Feet, cracks on**

*Ficus religiosa* (Moraceae)

*Mesua ferrea* (Calophyllaceae)

*Semecarpus anacardium* (Anacardiaceae)

**Female disorders**

*Acacia pennata* (Fabaceae)

*Boerhavia diffusa* (Nyctaginaceae)

*Cinnamomum camphora* (Lauraceae)

*Clerodendrum indicum* (Lamiaceae)

*Ficus religiosa* (Moraceae)

*Limonia acidissima* (Rutaceae)

*Mucuna pruriens* (Fabaceae)

*Myristica fragrans* (Myristicaceae)

*Nyctanthes arbor-tristis* (Oleaceae)

*Piper cubeba* (Piperaceae)

*Rotheca serrata* (Lamiaceae)

*Terminalia chebula* (Combretaceae)

**Fertility**

*Paederia foetida* (Rubiaceae)

**Fetal disorders**

*Nyctanthes arbor-tristis* (Oleaceae)

**Fever**

*Acorus calamus* (Acoraceae)

*Aegle marmelos* (Rutaceae)

*Allium cepa* (Amaryllidaceae)

*Allium sativum* (Amaryllidaceae)

*Aloe vera* (Asphodelaceae)

*Alpinia galanga* (Zingiberaceae)

*Alstonia scholaris* (Apocynaceae)

*Andrographis paniculata* (Acanthaceae)

*Anethum graveolens* (Apiaceae)

*Annona squamosa* (Annonaceae)

*Aquilaria malaccensis* (Thymelaeaceae)

*Aristolochia indica* (Aristolochiaceae)

*Artocarpus heterophyllus* (Moraceae)

*Averrhoa carambola* (Oxalidaceae)

*Azadirachta indica* (Meliaceae)

*Barleria prionitis* (Acanthaceae)

*Basella alba* (Basellaceae)

*Boerhavia diffusa* (Nyctaginaceae)

*Canna indica* (Cannaceae)

*Carallia brachiata* (Rhizophoraceae)

*Cardiospermum halicacabum* (Sapindaceae)

*Cascabela thevetia* (Apocynaceae)

*Catunaregam spinosa* (Rubiaceae)

*Centella asiatica* (Apiaceae)

*Cinnamomum bejolghota* (Lauraceae)

*Citrus aurantiifolia* (Rutaceae)

*Clerodendrum indicum* (Lamiaceae)

*Coccinia grandis* (Crassulaceae)

*Commelina paludosa* (Commelinaceae)

*Coptis teeta* (Ranunculaceae)

*Crateva religiosa* (Capparaceae)

*Curcuma comosa* (Zingiberaceae)

*Curcuma longa* (Zingiberaceae)

*Cymbopogon citratus* (Poaceae)

*Cymbopogon jwarancusa* (Poaceae)

*Cymbopogon nardus* (Poaceae)

*Cyperus scariosus* (Cyperaceae)

*Dillenia indica* (Dilleniaceae)

*Enydra fluctuans* (Asteraceae)

*Eucalyptus globulus* (Myrtaceae)

*Euphorbia hirta* (Euphorbiaceae)

*Exacum tetragonum* (Gentianaceae)

*Ficus rumphii* (Moraceae)

*Foeniculum vulgare* (Apiaceae)

*Girardinia diversifolia* (Urticaceae)

*Holarrhena pubescens* (Apocynaceae)

*Hydnocarpus kurzii* (Achariaceae)

*Justicia adhatoda* (Acanthaceae)

*Leucas cephalotes* (Lamiaceae)

*Limonia acidissima* (Rutaceae)

*Mentha arvensis* (Lamiaceae)

*Mesua ferrea* (Calophyllaceae)

*Magnolia champaca* (Magnoliaceae)

*Momordica charantia* (Cucurbitaceae)

*Monochoria vaginalis* (Pontederiaceae)

*Morinda coreia* (Rubiaceae)

*Myristica fragrans* (Myristicaceae)

*Nyctanthes arbor-tristis* (Oleaceae)

*Ocimum americanum* (Lamiaceae)

*Olax scandens* (Olacaceae)

*Piper betle* (Piperaceae)

*Plantago major* (Plantaginaceae)

*Premna serratifolia* (Lamiaceae)

*Quassia indica* (Simaroubaceae)

*Rauvolfia serpentina* (Apocynaceae)

*Ricinus communis* (Euphorbiaceae)

*Rotheca serrata* (Lamiaceae)

*Santalum album* (Santalaceae)

*Scoparia dulcis* (Plantaginaceae)

*Semecarpus anacardium* (Anacardiaceae)

*Sesbania grandiflora* (Fabaceae)

*Sesbania sesban* (Fabaceae)

*Solanum anguivi* (Solanaceae)

*Strobilanthes auriculatus* (Acanthaceae)

*Swertia chirayita* (Gentianaceae)

*Syzygium aromaticum* (Myrtaceae)

*Tadehagi triquetrum* (Fabaceae)

*Tanacetum cinerariifolium* (Asteraceae)

*Tinospora cordifolia* (Menispermaceae)

*Urena lobata* (Malvaceae)

*Volkameria inermis* (Lamiaceae)

*Ziziphus jujuba* (Rhamnaceae)

-from chest colds/infections

*Acalypha indica* (Euphorbiaceae)

-dengue hemorrhagic fever

*Cinnamomum bejolghota* (Lauraceae)

-typhoid fever

*Boerhavia diffusa* (Nyctaginaceae)

*Cinnamomum tamala* (Lauraceae)

*Swertia chirayita* (Gentianaceae)

-urinary disease

*Ipomoea aquatica* (Convolvulaceae)

**Fistula**

*Ficus religiosa* (Moraceae)

**Flatulence. *See also* Carminative.**

*Boerhavia diffusa* (Nyctaginaceae)

*Hygrophila phlomoides* (Acanthaceae)

*Ipomoea alba* (Convolvulaceae)

*Millingtonia hortensis* (Bignoniaceae)

*Oroxylum indicum* (Bignoniaceae)

*Senna sulfurea* (Fabaceae)

*Sinapis alba* (Brassicaceae)

-control

*Acacia pennata* (Fabaceae)

*Allium cepa* (Amaryllidaceae)

*Cardiospermum halicacabum* (Sapindaceae)

*Crinum asiaticum* (Amaryllidaceae)

*Croton persimilis* (Euphorbiaceae)

*Croton tiglium* (Euphorbiaceae)

*Cymbopogon citratus* (Poaceae)

*Cymbopogon nardus* (Poaceae)

*Eucalyptus globulus* (Myrtaceae)

*Ficus religiosa* (Moraceae)

*Gloriosa superba* (Colchicaceae)

*Gossypium barbadense* (Malvaceae)

*Gossypium hirsutum* (Malvaceae)

*Holarrhena pubescens* (Apocynaceae)

*Leucaena leucocephala* (Fabaceae)

*Magnolia champaca* (Magnoliaceae)

*Mentha arvensis* (Lamiaceae)

*Moringa oleifera* (Moringaceae)

*Mucuna pruriens* (Fabaceae)

*Ocimum americanum* (Lamiaceae)

*Piper betle* (Piperaceae)

*Plumeria rubra* (Apocynaceae)

*Ricinus communis* (Euphorbiaceae)

-elimination

*Allium cepa* (Amaryllidaceae)

*Allium sativum* (Amaryllidaceae)

*Asparagus officinalis* (Asparagaceae)

*Azadirachta indica* (Meliaceae)

*Cassia fistula* (Fabaceae)

*Citrus limon* (Rutaceae)

*Coccinia grandis* (Crassulaceae)

*Dregea volubilis* (Apocynaceae)

*Foeniculum vulgare* (Apiaceae)

*Hydnocarpus kurzii* (Achariaceae)

*Morinda angustifolia* (Rubiaceae)

*Myristica fragrans* (Myristicaceae)

*Piper cubeba* (Piperaceae)

*Piper nigrum* (Piperaceae)

*Plumbago zeylanica* (Plumbaginaceae)

*Rauvolfia serpentina* (Apocynaceae)

*Rhus chinensis* (Anacardiaceae)

*Solanum anguivi* (Solanaceae)

*Syzygium cumini* (Myrtaceae)

*Termin alia citrina* (Combretaceae)

*Tinospora cordifolia* (Menispermaceae)

*Zingiber montanum* (Zingiberaceae)

**Fomentation**

*Dodonaea viscosa* (Sapindaceae)

*Memecylon edule* (Melastomataceae)

*Pavetta indica* (Rubiaceae)

*Syzygium nervosum* (Myrtaceae)

**Fortifier. *See also* Strengthen.**

*Alpinia galanga* (Zingiberaceae)

*Andrographis paniculata* (Acanthaceae)

*Annona squamosa* (Annonaceae)

*Asparagus officinalis* (Asparagaceae)

*Benincasa hispida* (Cucurbitaceae)

*Butea monosperma* (Fabaceae)

*Canna indica* (Cannaceae)

*Carthamus tinctorius* (Asteraceae)

*Centella asiatica* (Apiaceae)

*Dregea volubilis* (Apocynaceae)

*Ficus religiosa* (Moraceae)

*Limonia acidissima* (Rutaceae)

*Moringa oleifera* (Moringaceae)

*Mucuna pruriens* (Fabaceae)

*Myristica fragrans* (Myristicaceae)

*Plantago major* (Plantaginaceae)

*Plumbago indica* (Plumbaginaceae)

*Rotheca serrata* (Lamiaceae)

*Senna alata* (Fabaceae)

*Sesbania sesban* (Fabaceae)

*Tinospora cordifolia* (Menispermaceae)

*Vitex trifolia* (Lamiaceae)

**Freckles**

*Allium sativum* (Amaryllidaceae)

*Phyllanthus emblica* (Phyllanthaceae)

**Galactagogue**

*Alternanthera sessilis* (Amaranthaceae)

*Capparis flavicans* (Capparaceae)

*Coccinia grandis* (Crassulaceae)

*Commicarpus chinensis* (Nyctaginaceae)

*Dioscorea bulbifera* (Dioscoreaceae)

*Euphorbia hirta* (Euphorbiaceae)

*Foeniculum vulgare* (Apiaceae)

*Ipomoea aquatica* (Convolvulaceae)

*Jatropha curcas* (Euphorbiaceae)

*Nigella sativa* (Ranunculaceae)

**Gall bladder**

-disease

*Acacia concinna* (Fabaceae)

*Andrographis paniculata* (Acanthaceae)

*Asparagus officinalis* (Asparagaceae)

*Coix lacryma-jobi* (Poaceae)

*Cymbopogon citratus* (Poaceae)

*Cyperus scariosus* (Cyperaceae)

*Nyctanthes arbor-tristis* (Oleaceae)

*Zingiber officinale* (Zingiberaceae)

-function

*Anethum graveolens* (Apiaceae)

*Basella alba* (Basellaceae)

*Carthamus tinctorius* (Asteraceae)

*Mucuna pruriens* (Fabaceae)

*Trachyspermum ammi* (Apiaceae)

-stones

*Alysicarpus vaginalis* (Fabaceae)

*Asparagus officinalis* (Asparagaceae)

*Carica papaya* (Caricaceae)

*Holarrhena pubescens* (Apocynaceae)

*Mimosa pudica* (Fabaceae)

**Gas. *See* Flatulence.**

**Gastric problems**

*Acacia concinna* (Fabaceae)

*Alstonia scholaris* (Apocynaceae)

*Aristolochia indica* (Aristolochiaceae)

*Plumbago zeylanica* (Plumbaginaceae)

*Tamarindus indica* (Fabaceae)

*Trichosanthes tricuspidata* (Cucurbitaceae)

**Gastritis**

*Curcuma longa* (Zingiberaceae)

*Ocimum americanum* (Lamiaceae)

**Gastrointestinal functioning**

*Trachyspermum ammi* (Apiaceae)

**Giddiness**

*Momordica charantia* (Cucurbitaceae)

**Gingivitis**

*Mimusops elengi* (Sapotaceae)

*Phyllanthus emblica* (Phyllanthaceae)

*Plantago major* (Plantaginaceae)

**Goiter**

*Allium sativum* (Amaryllidaceae)

*Momordica charantia* (Cucurbitaceae)

*Oroxylum indicum* (Bignoniaceae)

**Gonorrhea**

*Acalypha indica* (Euphorbiaceae)

*Amaranthus spinosus* (Amaranthaceae)

*Arundo donax* (Poaceae)

*Boerhavia diffusa* (Nyctaginaceae)

*Calophyllum inophyllum* (Calophyllaceae)

*Ceiba pentandra* (Malvaceae)

*Chrozophora plicata* (Euphorbiaceae)

*Cinnamomum bejolghota* (Lauraceae)

*Cinnamomum verum* (Lauraceae)

*Clerodendrum indicum* (Lamiaceae)

*Coix lacryma-jobi* (Poaceae)

*Cyperus scariosus* (Cyperaceae)

*Datura stramonium* (Solanaceae)

*Diospyros malabarica* (Ebenaceae)

*Dregea volubilis* (Apocynaceae)

Equisetum ramosissimum subsp. debile (Equisetaceae)

*Gloriosa superba* (Colchicaceae)

*Heliotropium indicum* (Boraginaceae)

*Ipomoea aquatica* (Convolvulaceae)

*Magnolia champaca* (Magnoliaceae)

*Mirabilis jalapa* (Nyctaginaceae)

*Plantago major* (Plantaginaceae)

*Plumeria rubra* (Apocynaceae)

*Rauvolfia serpentina* (Apocynaceae)

*Santalum album* (Santalaceae)

*Scoparia dulcis* (Plantaginaceae)

*Senna sulfurea* (Fabaceae)

*Sida spinosa* (Malvaceae)

*Strychnos potatorum* (Loganiaceae)

*Tamarindus indica* (Fabaceae)

*Tanacetum cinerariifolium* (Asteraceae)

*Tectona grandis* (Lamiaceae)

**Gout**

*Cananga odorata* (Annonaceae)

*Mallotus nudiflorus* (Euphorbiaceae)

*Melaleuca cajuputi* (Myrtaceae)

**Granulation**

*Jatropha multifida* (Euphorbiaceae)

*Ventilago denticulata* (Rhamnaceae)

**Gums, inflated**

*Curcuma longa* (Zingiberaceae)

**Hair**

-growth and health

*Acacia concinna* (Fabaceae)

*Carthamus tinctorius* (Asteraceae)

*Eclipta prostrata* (Asteraceae)

*Ricinus communis* (Euphorbiaceae)

*Schleichera oleosa* (Sapindaceae)

*Terminalia bellirica* (Combretaceae)

-loss

*Bryophyllum pinnatum* (Crassulaceae)

*Citrus aurantiifolia* (Rutaceae)

*Cymbopogon nardus* (Poaceae)

*Nyctanthes arbor-tristis* (Oleaceae)

**Halitosis**

*Aquilaria malaccensis* (Thymelaeaceae)

**Headaches**

*Abrus precatorius* (Fabaceae)

*Allium sativum* (Amaryllidaceae)

*Andrographis paniculata* (Acanthaceae)

*Cananga odorata* (Annonaceae)

*Citrus aurantiifolia* (Rutaceae)

*Clitoria ternatea* (Fabaceae)

*Coriandrum sativum* (Apiaceae)

*Datura stramonium* (Solanaceae)

*Eclipta prostrata* (Asteraceae)

*Elettaria cardamomum* (Zingiberaceae)

*Eucalyptus globulus* (Myrtaceae)

*Girardinia diversifolia* (Urticaceae)

*Haldina cordifolia* (Rubiaceae)

*Leucas cephalotes* (Lamiaceae)

*Moringa oleifera* (Moringaceae)

*Nerium oleander* (Apocynaceae)

*Ocimum americanum* (Lamiaceae)

*Ricinus communis* (Euphorbiaceae)

*Sesbania grandiflora* (Fabaceae)

*Syzygium aromaticum* (Myrtaceae)

*Tadehagi triquetrum* (Fabaceae)

*Terminalia bellirica* (Combretaceae)

*Terminalia citrina* (Combretaceae)

-migraines

*Coffea arabica* (Rubiaceae)

**Healing, hasten**

*Alstonia scholaris* (Apocynaceae)

*Aquilaria malaccensis* (Thymelaeaceae)

*Basella alba* (Basellaceae)

*Dregea volubilis* (Apocynaceae)

*Euonymus kachinensis* (Celastraceae)

*Ficus religiosa* (Moraceae)

*Gossypium barbadense* (Malvaceae)

*Gossypium hirsutum* (Malvaceae)

*Rotheca serrata* (Lamiaceae)

**Health, general**

*Carica papaya* (Caricaceae)

*Eclipta prostrata* (Asteraceae)

*Ficus religiosa* (Moraceae)

*Spermacoce hispida* (Rubiaceae)

*Tinospora cordifolia* (Menispermaceae)

**Heart**

-disease

*Alpinia galanga* (Zingiberaceae)

*Alstonia scholaris* (Apocynaceae)

*Aquilaria malaccensis* (Thymelaeaceae)

*Aristolochia indica* (Aristolochiaceae)

*Arundo donax* (Poaceae)

*Boerhavia diffusa* (Nyctaginaceae)

*Cardiospermum halicacabum* (Sapindaceae)

*Carica papaya* (Caricaceae)

*Coix lacryma-jobi* (Poaceae)

*Curcuma comosa* (Zingiberaceae)

*Curcuma zedoaria* (Zingiberaceae)

*Cymbopogon citratus* (Poaceae)

*Elettaria cardamomum* (Zingiberaceae)

*Mansonia gagei* (Malvaceae)

*Mimusops elengi* (Sapotaceae)

*Morinda angustifolia* (Rubiaceae)

*Ocimum americanum* (Lamiaceae)

*Oroxylum indicum* (Bignoniaceae)

*Sesbania grandiflora* (Fabaceae)

*Tanacetum cinerariifolium* (Asteraceae)

*Terminalia bellirica* (Combretaceae)

*Terminalia tomentosa* (Combretaceae)

-functions

*Ficus religiosa* (Moraceae)

*Magnolia champaca* (Magnoliaceae)

*Piper betle* (Piperaceae)

-irregularities

*Cinnamomum bejolghota* (Lauraceae)

**Heat**

-body

*Arundo donax* (Poaceae)

*Fritillaria cirrhosa* (Liliaceae)

*Senna sulfurea* (Fabaceae)

-diminish

*Azadirachta indica* (Meliaceae)

*Coix lacryma-jobi* (Poaceae)

*Mimusops elengi* (Sapotaceae)

*Strychnos potatorum* (Loganiaceae)

-stroke

*Acacia concinna* (Fabaceae)

*Aegle marmelos* (Rutaceae)

*Piper betle* (Piperaceae)

**Heaviness**

*Aristolochia indica* (Aristolochiaceae)

*Blumea balsamifera* (Asteraceae)

*Cinnamomum bejolghota* (Lauraceae)

*Citrus limon* (Rutaceae)

*Enydra fluctuans* (Asteraceae)

**Hematuria**

*Rhizophora mucronata* (Rhizophoraceae)

**Hemorrhage. *See also* Bleeding.**

*Asparagus officinalis* (Asparagaceae)

*Calophyllum inophyllum* (Calophyllaceae)

*Cassia fistula* (Fabaceae)

*Catharanthus roseus* (Apocynaceae)

*Clitoria ternatea* (Fabaceae)

*Cordyline fruticosa* (Laxmanniaceae)

*Ficus religiosa* (Moraceae)

*Ixora chinensis* (Rubiaceae)

*Magnolia champaca* (Magnoliaceae)

*Mimosa pudica* (Fabaceae)

*Morinda angustifolia* (Rubiaceae)

*Nyctanthes arbor-tristis* (Oleaceae)

*Ocimum americanum* (Lamiaceae)

*Tadehagi triquetrum* (Fabaceae)

*Tectona grandis* (Lamiaceae)

*Terminalia bellirica* (Combretaceae)

**Hemorrhoids**

*Abrus precatorius* (Fabaceae)

*Acorus calamus* (Acoraceae)

*Allium cepa* (Amaryllidaceae)

*Averrhoa carambola* (Oxalidaceae)

*Boerhavia diffusa* (Nyctaginaceae)

*Bryophyllum pinnatum* (Crassulaceae)

*Calotropis procera* (Apocynaceae)

*Canna indica* (Cannaceae)

*Carica papaya* (Caricaceae)

*Carthamus tinctorius* (Asteraceae)

*Cascabela thevetia* (Apocynaceae)

*Cinnamomum verum* (Lauraceae)

*Croton persimilis* (Euphorbiaceae)

*Gloriosa superba* (Colchicaceae)

*Holarrhena pubescens* (Apocynaceae)

*Mesua ferrea* (Calophyllaceae)

*Mimosa pudica* (Fabaceae)

*Momordica charantia* (Cucurbitaceae)

*Myristica fragrans* (Myristicaceae)

*Ocimum americanum* (Lamiaceae)

*Oroxylum indicum* (Bignoniaceae)

*Piper cubeba* (Piperaceae)

*Piper nigrum* (Piperaceae)

*Plantago major* (Plantaginaceae)

*Semecarpus anacardium* (Anacardiaceae)

*Sinapis alba* (Brassicaceae)

*Strychnos potatorum* (Loganiaceae)

*Syzygium cumini* (Myrtaceae)

*Tadehagi triquetrum* (Fabaceae)

*Tamarindus indica* (Fabaceae)

*Terminalia bellirica* (Combretaceae)

*Terminalia chebula* (Combretaceae)

*Zingiber montanum* (Zingiberaceae)

**Hepalomegaly. *See* Liver.**

**Hepatitis**

*Croton persimilis* (Euphorbiaceae)

*Eclipta prostrata* (Asteraceae)

*Momordica charantia* (Cucurbitaceae)

*Ocimum americanum* (Lamiaceae)

**Herpes**

*Arundo donax* (Poaceae)

*Cassia fistula* (Fabaceae)

*Coix lacryma-jobi* (Poaceae)

*Cyperus scariosus* (Cyperaceae)

*Dregea volubilis* (Apocynaceae)

*Ficus religiosa* (Moraceae)

*Tectona grandis* (Lamiaceae)

**Hiccups**

*Apium graveolens* (Apiaceae)

*Clitoria ternatea* (Fabaceae)

*Piper nigrum* (Piperaceae)

*Santalum album* (Santalaceae)

*Zingiber officinale* (Zingiberaceae)

**Hunger, initiate**

*Carica papaya* (Caricaceae)

*Cymbopogon nardus* (Poaceae)

**Hydragogue. *See also* Carthatic, Laxative, Purgative.**

*Allamanda cathartica* (Apocynaceae)

**Hypertension**

*Acalypha indica* (Euphorbiaceae)

*Allium sativum* (Amaryllidaceae)

*Apium graveolens* (Apiaceae)

*Centella asiatica* (Apiaceae)

*Croton tiglium* (Euphorbiaceae)

*Mentha arvensis* (Lamiaceae)

*Millingtonia hortensis* (Bignoniaceae)

*Morinda angustifolia* (Rubiaceae)

*Rauvolfia serpentina* (Apocynaceae)

**Hypoglycemic**

*Zea mays* (Poaceae)

**Hysteria**

*Passiflora foetida* (Passifloraceae)

**Illnesses, infant**

*Acalypha indica* (Euphorbiaceae)

*Blumea balsamifera* (Asteraceae)

**Immune function**

*Plumeria rubra* (Apocynaceae)

**Impetigo**

*Bryophyllum pinnatum* (Crassulaceae)

*Eucalyptus globulus* (Myrtaceae)

*Plantago major* (Plantaginaceae)

*Santalum album* (Santalaceae)

*Tadehagi triquetrum* (Fabaceae)

**Indigestion**

*Acacia pennata* (Fabaceae)

*Aegle marmelos* (Rutaceae)

*Alpinia galanga* (Zingiberaceae)

*Andrographis paniculata* (Acanthaceae)

*Apium graveolens* (Apiaceae)

*Aquilaria malaccensis* (Thymelaeaceae)

*Aristolochia indica* (Aristolochiaceae)

*Artocarpus heterophyllus* (Moraceae)

*Boerhavia diffusa* (Nyctaginaceae)

*Cardiospermum halicacabum* (Sapindaceae)

*Carica papaya* (Caricaceae)

*Combretum indicum* (Combretaceae)

*Crateva religiosa* (Capparaceae)

*Croton persimilis* (Euphorbiaceae)

*Curcuma comosa* (Zingiberaceae)

*Eurycoma longifolia* (Simaroubaceae)

*Foeniculum vulgare* (Apiaceae)

*Gossypium barbadense* (Malvaceae)

*Gossypium hirsutum* (Malvaceae)

*Grewia nervosa* (Malvaceae)

*Hydnocarpus kurzii* (Achariaceae)

*Ipomoea aquatica* (Convolvulaceae)

*Morinda angustifolia* (Rubiaceae)

*Myristica fragrans* (Myristicaceae)

*Oroxylum indicum* (Bignoniaceae)

*Phyllanthus emblica* (Phyllanthaceae)

*Piper betle* (Piperaceae)

*Senna alexandrina* (Fabaceae)

*Senna sulfurea* (Fabaceae)

*Syzygium cumini* (Myrtaceae)

*Tadehagi triquetrum* (Fabaceae)

*Tamarindus indica* (Fabaceae)

*Trichosanthes tricuspidata* (Cucurbitaceae)

*Urena lobata* (Malvaceae)

*Vitex trifolia* (Lamiaceae)

**Infection**

*Acalypha indica* (Euphorbiaceae)

*Barleria prionitis* (Acanthaceae)

*Carallia brachiata* (Rhizophoraceae)

*Clitoria ternatea* (Fabaceae)

*Cordyline fruticosa* (Laxmanniaceae)

*Croton tiglium* (Euphorbiaceae)

*Diospyros malabarica* (Ebenaceae)

*Holarrhena pubescens* (Apocynaceae)

*Hydnocarpus kurzii* (Achariaceae)

*Ipomoea aquatica* (Convolvulaceae)

*Plumeria rubra* (Apocynaceae)

*Rotheca serrata* (Lamiaceae)

*Sesbania sesban* (Fabaceae)

*Zingiber officinale* (Zingiberaceae)

-ear

*Allium cepa* (Amaryllidaceae)

*Allium sativum* (Amaryllidaceae)

*Cassia fistula* (Fabaceae)

*Croton tiglium* (Euphorbiaceae)

*Gloriosa superba* (Colchicaceae)

*Gossypium barbadense* (Malvaceae)

*Gossypium hirsutum* (Malvaceae)

*Holarrhena pubescens* (Apocynaceae)

*Trichosanthes tricuspidata* (Cucurbitaceae)

-fungus

*Barleria prionitis* (Acanthaceae)

-gums

*Cuscuta reflexa* (Convolvulaceae)

-intestinal

*Plantago major* (Plantaginaceae)

-post childbirth

*Ocimum americanum* (Lamiaceae)

-skin

*Eucalyptus globulus* (Myrtaceae)

*Gloriosa superba* (Colchicaceae)

**Infirmity (Debility)**

*Hibiscus sabdariffa* (Malvaceae)

**Inflammations**

*Acalypha indica* (Euphorbiaceae)

*Aglaia cucullata* (Meliaceae)

*Alpinia galanga* (Zingiberaceae)

*Alstonia scholaris* (Apocynaceae)

*Anethum graveolens* (Apiaceae)

*Apium graveolens* (Apiaceae)

*Barleria prionitis* (Acanthaceae)

*Boerhavia diffusa* (Nyctaginaceae)

*Carthamus tinctorius* (Asteraceae)

*Centella asiatica* (Apiaceae)

*Cinnamomum bejolghota* (Lauraceae)

*Commelina paludosa* (Commelinaceae)

*Cordyline fruticosa* (Laxmanniaceae)

*Croton persimilis* (Euphorbiaceae)

*Croton tiglium* (Euphorbiaceae)

*Curcuma comosa* (Zingiberaceae)

*Curcuma longa* (Zingiberaceae)

*Cymbopogon citratus* (Poaceae)

*Cymbopogon nardus* (Poaceae)

*Diospyros malabarica* (Ebenaceae)

*Eclipta prostrata* (Asteraceae)

*Erythrina variegata* (Fabaceae)

*Ipomoea aquatica* (Convolvulaceae)

*Martynia annua* (Martyniaceae)

*Mimusops elengi* (Sapotaceae)

*Momordica charantia* (Cucurbitaceae)

*Moringa oleifera* (Moringaceae)

*Myristica fragrans* (Myristicaceae)

*Nerium oleander* (Apocynaceae)

*Rotheca serrata* (Lamiaceae)

*Semecarpus anacardium* (Anacardiaceae)

*Tectona grandis* (Lamiaceae)

*Zingiber officinale* (Zingiberaceae)

-abdomen

*Cuscuta reflexa* (Convolvulaceae)

-appendix

*Acalypha indica* (Euphorbiaceae)

-bladder

*Benincasa hispida* (Cucurbitaceae)

*Crateva religiosa* (Capparaceae)

-breast

*Datura stramonium* (Solanaceae)

-eye

*Albizia lebbeck* (Fabaceae)

*Cissampelos pareira* (Menispermaceae)

*Phyllanthus emblica* (Phyllanthaceae)

*Piper betle* (Piperaceae)

-gum

*Cuscuta reflexa* (Convolvulaceae)

*Ocimum americanum* (Lamiaceae)

*Senna alata* (Fabaceae)

*Terminalia bellirica* (Combretaceae)

-internal

*Rotheca serrata* (Lamiaceae)

-intestines

*Cinnamomum bejolghota* (Lauraceae)

-joint

*Acalypha indica* (Euphorbiaceae)

*Aristolochia indica* (Aristolochiaceae)

*Calotropis procera* (Apocynaceae)

*Crateva religiosa* (Capparaceae)

*Datura stramonium* (Solanaceae)

*Eclipta prostrata* (Asteraceae)

*Eucalyptus globulus* (Myrtaceae)

*Leucas cephalotes* (Lamiaceae)

*Mentha arvensis* (Lamiaceae)

*Mesua ferrea* (Calophyllaceae)

*Magnolia champaca* (Magnoliaceae)

*Nyctanthes arbor-tristis* (Oleaceae)

*Ocimum americanum* (Lamiaceae)

*Piper longum* (Piperaceae)

*Plantago major* (Plantaginaceae)

*Sesbania grandiflora* (Fabaceae)

*Sinapis alba* (Brassicaceae)

*Tinospora cordifolia* (Menispermaceae)

*Urena lobata* (Malvaceae)

-liver

*Cinnamomum bejolghota* (Lauraceae)

*Clitoria ternatea* (Fabaceae)

-oral

*Plantago major* (Plantaginaceae)

-spleen

*Citrus aurantiifolia* (Rutaceae)

*Clitoria ternatea* (Fabaceae)

*Myristica fragrans* (Myristicaceae)

*Sesbania sesban* (Fabaceae)

*Sinapis alba* (Brassicaceae)

*Syzygium cumini* (Myrtaceae)

-testes

*Acacia pennata* (Fabaceae)

*Clitoria ternatea* (Fabaceae)

*Gossypium barbadense* (Malvaceae)

*Gossypium hirsutum* (Malvaceae)

*Ricinus communis* (Euphorbiaceae)

**Influenza**

*Cinnamomum tamala* (Lauraceae)

*Piper longum* (Piperaceae)

*Sesbania grandiflora* (Fabaceae)

*Zingiber officinale* (Zingiberaceae)

**Insanity**

*Acorus calamus* (Acoraceae)

*Benincasa hispida* (Cucurbitaceae)

*Carthamus tinctorius* (Asteraceae)

*Gossypium barbadense* (Malvaceae)

*Gossypium hirsutum* (Malvaceae)

**Insomnia**

*Myristica fragrans* (Myristicaceae)

**Internal disease or disorder, general**

*Aquilaria malaccensis* (Thymelaeaceae)

*Aristolochia indica* (Aristolochiaceae)

*Schefflera venulosa* (Araliaceae)

**Intestines**

-amoebae

*Dysphania ambrosioides* (Amaranthaceae)

-function

*Coptis teeta* (Ranunculaceae)

*Selinum wallichianum* (Apiaceae)

-**infection**

*Cinnamomum bejolghota* (Lauraceae)

*Plantago major* (Plantaginaceae)

*Ocimum americanum* (Lamiaceae)

**Intoxication**

*Cannabis sativa* (Cannabaceae)

*Millingtonia hortensis* (Bignoniaceae)

**Irritant**

*Caryota mitis* (Arecaceae)

*Capparis zeylanica* (Capparaceae)

**Itch**

*Acacia farnesiana* (Fabaceae)

*Acalypha indica* (Euphorbiaceae)

*Arundo donax* (Poaceae)

*Azadirachta indica* (Meliaceae)

*Barleria prionitis* (Acanthaceae)

*Carallia brachiata* (Rhizophoraceae)

*Carica papaya* (Caricaceae)

*Carthamus tinctorius* (Asteraceae)

*Centella asiatica* (Apiaceae)

*Cinnamomum bejolghota* (Lauraceae)

*Citrus aurantiifolia* (Rutaceae)

*Coix lacryma-jobi* (Poaceae)

*Convolvulus arvensis* (Convolvulaceae)

*Curcuma longa* (Zingiberaceae)

*Cuscuta reflexa* (Convolvulaceae)

*Cyperus scariosus* (Cyperaceae)

*Ficus religiosa* (Moraceae)

*Leucas cephalotes* (Lamiaceae)

*Mansonia gagei* (Malvaceae)

*Mirabilis jalapa* (Nyctaginaceae)

*Moringa oleifera* (Moringaceae)

*Nerium oleander* (Apocynaceae)

*Nyctanthes arbor-tristis* (Oleaceae)

*Ocimum americanum* (Lamiaceae)

*Phyllanthus emblica* (Phyllanthaceae)

*Rotheca serrata* (Lamiaceae)

*Santalum album* (Santalaceae)

*Semecarpus anacardium* (Anacardiaceae)

*Sinapis alba* (Brassicaceae)

*Tectona grandis* (Lamiaceae)

*Terminalia bellirica* (Combretaceae)

*Trachyspermum ammi* (Apiaceae)

*Xylocarpus granatum* (Meliaceae)

*Xylocarpus moluccensis* (Meliaceae)

**Jaundice**

*Acacia concinna* (Fabaceae)

*Aloe vera* (Asphodelaceae)

*Canna indica* (Cannaceae)

*Carthamus tinctorius* (Asteraceae)

*Croton tiglium* (Euphorbiaceae)

*Cymbopogon citratus* (Poaceae)

*Hygrophila auriculata* (Acanthaceae)

*Leucas cephalotes* (Lamiaceae)

*Momordica charantia* (Cucurbitaceae)

*Oldenlandia corymbosa* (Rubiaceae)

*Tamarindus indica* (Fabaceae)

**Joint, dislocation**

*Bryophyllum pinnatum* (Crassulaceae)

*Mirabilis jalapa* (Nyctaginaceae)

**Kidney**

-disease

*Magnolia champaca* (Magnoliaceae)

*Ocimum americanum* (Lamiaceae)

*Ocimum tenuiflorum* (Lamiaceae)

*Orthosiphon aristatus* (Lamiaceae)

*Pogostemon cablin* (Lamiaceae)

*Portulaca oleracea* (Portulaceae)

-stones

*Alysicarpus vaginalis* (Fabaceae)

*Amaranthus spinosus* (Amaranthaceae)

*Arundo donax* (Poaceae)

*Asparagus officinalis* (Asparagaceae)

*Benincasa hispida* (Cucurbitaceae)

*Calotropis procera* (Apocynaceae)

*Carthamus tinctorius* (Asteraceae)

*Citrus limon* (Rutaceae)

*Coix lacryma-jobi* (Poaceae)

*Crateva religiosa* (Capparaceae)

*Ipomoea alba* (Convolvulaceae)

*Ipomoea aquatica* (Convolvulaceae)

*Prunus cerasoides* (Rosaceae)

*Terminalia bellirica* (Combretaceae)

**Lactation**

*Anethum graveolens* (Apiaceae)

*Alstonia scholaris* (Apocynaceae)

*Boerhavia diffusa* (Nyctaginaceae)

*Gossypium barbadense* (Malvaceae)

*Gossypium hirsutum* (Malvaceae)

*Morinda angustifolia* (Rubiaceae)

*Mucuna pruriens* (Fabaceae)

*Ocimum americanum* (Lamiaceae)

*Piper nigrum* (Piperaceae)

*Rotheca serrata* (Lamiaceae)

*Sesbania sesban* (Fabaceae)

*Tectona grandis* (Lamiaceae)

-induction

*Alstonia scholaris* (Apocynaceae)

*Sesbania sesban* (Fabaceae)

**Laryngitis**

*Cardiospermum halicacabum* (Sapindaceae)

*Citrus limon* (Rutaceae)

*Moringa oleifera* (Moringaceae)

*Zingiber officinale* (Zingiberaceae)

**Laxative. *See also* Carthatic, Hydragogue, Purgative.**

*Amaranthus cruentus* (Amaranthaceae)

*Amaranthus spinosus* (Amaranthaceae)

*Arachis hypogaea* (Fabaceae)

*Argemone mexicana* (Papaveraceae)

*Aristolochia tagala* (Aristolochiaceae)

*Barringtonia acutangula* (Lecythidaceae)

*Bauhinia acuminata* (Fabaceae)

*Bauhinia purpurea* (Fabaceae)

*Boehmeria nivea* (Urticaceae)

*Brassica oleracea* (Brassicaceae)

*Buchanania lancifolia* (Anacardiaceae)

*Cardiospermum halicacabum* (Sapindaceae)

*Cassia fistula* (Fabaceae)

*Ceiba pentandra* (Malvaceae)

*Centella asiatica* (Apiaceae)

*Chamaecrista pumila* (Fabaceae)

*Cheilocostus speciosus* (Costaceae)

*Coccinia grandis* (Crassulaceae)

*Convolvulus arvensis* (Convolvulaceae)

*Coriaria nepalensis* (Coriariaceae)

*Croton tiglium* (Euphorbiaceae)

*Cullen corylifolium* (Fabaceae)

*Eucalyptus globulus* (Myrtaceae)

*Hibiscus cannabinus* (Malvaceae)

*Ipomoea alba* (Convolvulaceae)

*Jatropha curcas* (Euphorbiaceae)

*Limonia acidissima* (Rutaceae)

*Luffa cylindrica* (Cucurbitaceae)

*Mallotus philippensis* (Euphorbiaceae)

*Mangifera indica* (Anacardiaceae)

*Momordica charantia* (Cucurbitaceae)

*Momordica cochinchinensis* (Cucurbitaceae)

*Morinda coreia* (Rubiaceae)

*Pavetta indica* (Rubiaceae)

*Phyllanthus acidus* (Phyllanthaceae)

*Phyllanthus emblica* (Phyllanthaceae)

*Plumeria rubra* (Apocynaceae)

*Portulaca oleracea* (Portulaceae)

*Premna serratifolia* (Lamiaceae)

*Ricinus communis* (Euphorbiaceae)

*Semecarpus anacardium* (Anacardiaceae)

*Senna alexandrina* (Fabaceae)

*Senna italica* (Fabaceae)

*Senna tora* (Fabaceae)

*Sesamum indicum* (Pedaliaceae)

*Swertia chirayita* (Gentianaceae)

*Tamarindus indica* (Fabaceae)

*Terminalia chebula* (Combretaceae)

*Ziziphus jujuba* (Rhamnaceae)

**Leprosy**

*Ageratum conyzoides* (Asteraceae)

*Aloe vera* (Asphodelaceae)

*Andrographis paniculata* (Acanthaceae)

*Aquilaria malaccensis* (Thymelaeaceae)

*Butea monosperma* (Fabaceae)

*Calophyllum inophyllum* (Calophyllaceae)

*Calotropis gigantea* (Apocynaceae)

*Calotropis procera* (Apocynaceae)

*Cascabela thevetia* (Apocynaceae)

*Cassia fistula* (Fabaceae)

*Centella asiatica* (Apiaceae)

*Clausena excavata* (Rutaceae)

*Clerodendrum indicum* (Lamiaceae)

*Cymbopogon nardus* (Poaceae)

*Cyperus scariosus* (Cyperaceae)

*Enydra fluctuans* (Asteraceae)

*Ficus religiosa* (Moraceae)

*Fritillaria cirrhosa* (Liliaceae)

*Gloriosa superba* (Colchicaceae)

*Holarrhena pubescens* (Apocynaceae)

*Hydnocarpus kurzii* (Achariaceae)

*Ipomoea alba* (Convolvulaceae)

*Ipomoea pes-caprae* (Convolvulaceae)

*Luffa cylindrica* (Cucurbitaceae)

*Momordica charantia* (Cucurbitaceae)

*Nerium oleander* (Apocynaceae)

*Plumbago indica* (Plumbaginaceae)

*Plumbago zeylanica* (Plumbaginaceae)

*Plumeria rubra* (Apocynaceae)

*Ricinus communis* (Euphorbiaceae)

*Semecarpus anacardium* (Anacardiaceae)

*Senna alata* (Fabaceae)

*Senna alexandrina* (Fabaceae)

*Sesbania grandiflora* (Fabaceae)

*Sesbania sesban* (Fabaceae)

*Sinapis alba* (Brassicaceae)

*Tectona grandis* (Lamiaceae)

*Terminalia bellirica* (Combretaceae)

*Trichosanthes tricuspidata* (Cucurbitaceae)

*Urena lobata* (Malvaceae)

*Zingiber montanum* (Zingiberaceae)

**Lesions. *See also* Skin sores.**

*Nerium oleander* (Apocynaceae)

**Leucoderma**

*Anacardium occidentale* (Anacardiaceae)

*Plumbago indica* (Plumbaginaceae)

*Plumbago zeylanica* (Plumbaginaceae)

**Lice**

*Cuscuta reflexa* (Convolvulaceae)

*Tadehagi triquetrum* (Fabaceae)

**Limb problems**

*Alpinia officinarum* (Zingiberaceae)

**Liniment**

*Cardiospermum halicacabum* (Sapindaceae)

*Helicteres isora* (Malvaceae)

*Kydia calycina* (Malvaceae)

*Syzygium nervosum* (Myrtaceae)

**Lips, cracked**

*Ficus religiosa* (Moraceae)

*Tanacetum cinerariifolium* (Asteraceae)

**Liver**

-disease

*Aloe vera* (Asphodelaceae)

*Aquilaria malaccensis* (Thymelaeaceae)

*Asparagus officinalis* (Asparagaceae)

*Croton persimilis* (Euphorbiaceae)

*Cuscuta reflexa* (Convolvulaceae)

*Erythrina variegata* (Fabaceae)

*Mentha arvensis* (Lamiaceae)

*Monochoria vaginalis* (Pontederiaceae)

*Premna serratifolia* (Lamiaceae)

*Senna alexandrina* (Fabaceae)

*Sesbania sesban* (Fabaceae)

-enlargement (hepalomegaly)

*Acacia concinna* (Fabaceae)

*Carica papaya* (Caricaceae)

*Croton persimilis* (Euphorbiaceae)

-health

*Enydra fluctuans* (Asteraceae)

*Piper nigrum* (Piperaceae)

**Longevity**

*Allium sativum* (Amaryllidaceae)

*Boerhavia diffusa* (Nyctaginaceae)

*Butea monosperma* (Fabaceae)

*Carica papaya* (Caricaceae)

*Curcuma longa* (Zingiberaceae)

*Cuscuta reflexa* (Convolvulaceae)

*Eclipta prostrata* (Asteraceae)

*Fritillaria cirrhosa* (Liliaceae)

*Moringa oleifera* (Moringaceae)

*Phyllanthus emblica* (Phyllanthaceae)

*Plumbago indica* (Plumbaginaceae)

*Terminalia chebula* (Combretaceae)

*Terminalia citrina* (Combretaceae)

*Tinospora cordifolia* (Menispermaceae)

**Lung disease. *See also* Pneumonia.**

*Acalypha indica* (Euphorbiaceae)

*Allium sativum* (Amaryllidaceae)

*Alstonia scholaris* (Apocynaceae)

*Benincasa hispida* (Cucurbitaceae)

*Clerodendrum indicum* (Lamiaceae)

*Coccinia grandis* (Crassulaceae)

*Cordyline fruticosa* (Laxmanniaceae)

*Ficus religiosa* (Moraceae)

*Ocimum americanum* (Lamiaceae)

*Plantago major* (Plantaginaceae)

*Plumbago zeylanica* (Plumbaginaceae)

*Sesbania grandiflora* (Fabaceae)

*Sesbania sesban* (Fabaceae)

*Syzygium aromaticum* (Myrtaceae)

*Zingiber officinale* (Zingiberaceae)

**Malaises**

*Allium sativum* (Amaryllidaceae)

-male

*Ricinus communis* (Euphorbiaceae)

-female

*Cinnamomum bejolghota* (Lauraceae)

**Malaria**

*Acacia concinna* (Fabaceae)

*Cananga odorata* (Annonaceae)

*Coptis teeta* (Ranunculaceae)

*Cymbopogon citratus* (Poaceae)

*Cymbopogon jwarancusa* (Poaceae)

*Momordica charantia* (Cucurbitaceae)

*Nyctanthes arbor-tristis* (Oleaceae)

*Picrasma javanica* (Simaroubaceae)

*Piper cubeba* (Piperaceae)

*Piper longum* (Piperaceae)

*Plantago major* (Plantaginaceae)

*Vitex trifolia* (Lamiaceae)

**Male diseases/maladies**

*Cinnamomum camphora* (Lauraceae)

*Clerodendrum indicum* (Lamiaceae)

*Croton persimilis* (Euphorbiaceae)

*Ipomoea alba* (Convolvulaceae)

*Leucaena leucocephala* (Fabaceae)

*Mucuna pruriens* (Fabaceae)

*Myristica fragrans* (Myristicaceae)

*Piper cubeba* (Piperaceae)

*Terminalia chebula* (Combretaceae)

-spermatorrhea

*Abelmoschus moschatus* (Malvaceae)

**Measles**

*Eclipta prostrata* (Asteraceae)

**Memory**

*Gossypium barbadense* (Malvaceae)

*Gossypium hirsutum* (Malvaceae)

**Menopause**

*Canna indica* (Cannaceae)

*Cordyline fruticosa* (Laxmanniaceae)

*Elettaria cardamomum* (Zingiberaceae)

**Menstruation**

*Allium sativum* (Amaryllidaceae)

*Aristolochia indica* (Aristolochiaceae)

*Calotropis procera* (Apocynaceae)

*Carica papaya* (Caricaceae)

*Clerodendrum indicum* (Lamiaceae)

*Coix lacryma-jobi* (Poaceae)

*Cordyline fruticosa* (Laxmanniaceae)

*Justicia adhatoda* (Acanthaceae)

*Mentha arvensis* (Lamiaceae)

*Millingtonia hortensis* (Bignoniaceae)

*Morinda citrifolia* (Rubiaceae)

*Ocimum americanum* (Lamiaceae)

*Oroxylum indicum* (Bignoniaceae)

*Vitex trifolia* (Lamiaceae)

*Zingiber montanum* (Zingiberaceae)

-disorders

*Abroma augustum* (Malvaceae)

*Aloe vera* (Asphodelaceae)

*Ardisia humilis* (Primulaceae)

*Arundo donax* (Poaceae)

*Canna indica* (Cannaceae)

*Eclipta prostrata* (Asteraceae)

*Elephantopus scaber* (Asteraceae)

*Elettaria cardamomum* (Zingiberaceae)

*Myristica fragrans* (Myristicaceae)

*Nauclea orientalis* (Rubiaceae)

*Plumbago indica* (Plumbaginaceae)

*Sesbania sesban* (Fabaceae)

*Tinospora cordifolia* (Menispermaceae)

*Vitex trifolia* (Lamiaceae)

-excessive

*Amaranthus spinosus* (Amaranthaceae)

*Nyctanthes arbor-tristis* (Oleaceae)

*Scoparia dulcis* (Plantaginaceae)

-menorrhagia

*Phyllanthus niruri* (Phyllanthaceae)

*Saraca indica* (Fabaceae)

*Ziziphus rugosa* (Rhamnaceae)

-residual discharge

*Clerodendrum indicum* (Lamiaceae)

-stimulation of,

*Moringa oleifera* (Moringaceae)

**Mental disorder**

*Gossypium barbadense* (Malvaceae)

*Gossypium hirsutum* (Malvaceae)

*Rauvolfia serpentina* (Apocynaceae)

**Mind, clearing/focusing**

*Eucalyptus globulus* (Myrtaceae)

*Gossypium barbadense* (Malvaceae)

*Gossypium hirsutum* (Malvaceae)

*Mentha arvensis* (Lamiaceae)

*Sesbania grandiflora* (Fabaceae)

**Morning-sickness**

*Aegle marmelos* (Rutaceae)

*Syzygium aromaticum* (Myrtaceae)

**Mosquito repellent**

*Cymbopogon citratus* (Poaceae)

**Mouth**

-blisters

*Aristolochia indica* (Aristolochiaceae)

*Convolvulus arvensis* (Convolvulaceae)

-dry

*Syzygium aromaticum* (Myrtaceae)

-sores

*Aegle marmelos* (Rutaceae)

*Phyllanthus emblica* (Phyllanthaceae)

*Syzygium cumini* (Myrtaceae)

**Mouthwash**

*Curcuma zedoaria* (Zingiberaceae)

*Neolamarckia cadamba* (Rubiaceae)

*Phyllanthus emblica* (Phyllanthaceae)

*Terminalia citrina* (Combretaceae)

**Mucus. *See also* Phlegm.**

*Aegle marmelos* (Rutaceae)

*Canna indica* (Cannaceae)

*Cyperus scariosus* (Cyperaceae)

*Euphorbia hirta* (Euphorbiaceae)

*Tradescantia spathacea* (Commelinaceae)

**Muscle**

*Carica papaya* (Caricaceae)

-knots

*Bryophyllum pinnatum* (Crassulaceae)

*Curcuma longa* (Zingiberaceae)

*Holarrhena pubescens* (Apocynaceae)

*Mirabilis jalapa* (Nyctaginaceae)

*Morinda coreia* (Rubiaceae)

*Zingiber montanum* (Zingiberaceae)

-spasms/twitches

*Mentha arvensis* (Lamiaceae)

*Sinapis alba* (Brassicaceae)

**Narcotic**

*Cycas rumphii* (Cycadaceae)

*Datura metel* (Solanaceae)

*Lagerstroemia speciosa* (Lythraceae)

*Rhododendron moulmainense* (Ericaceae)

*Solanum melongena* (Solanaceae)

**Nausea**

*Allium cepa* (Amaryllidaceae)

*Anathum graveolens* (Apiaceae)

*Cinnamomum tamala* (Lauraceae)

*Citrus limon* (Rutaceae)

*Coriandrum sativum* (Apiaceae)

*Cyperus scariosus* (Cyperaceae)

*Ficus religiosa* (Moraceae)

*Flacourtia jangomas* (Salicaceae)

*Foeniculum vulgare* (Apiaceae)

*Mentha arvensis* (Lamiaceae)

*Myristica fragrans* (Myristicaceae)

*Rotheca serrata* (Lamiaceae)

*Scoparia dulcis* (Plantaginaceae)

*Syzygium aromaticum* (Myrtaceae)

*Zingiber officinale* (Zingiberaceae)

**Nervine**

*Selinum wallichianum* (Apiaceae)

**Neuralgia**

*Premna serratifolia* (Lamiaceae)

**Neurological disease**

*Acalypha indica* (Euphorbiaceae)

**Night-blindness**

*Allium cepa* (Amaryllidaceae)

*Phyllanthus emblica* (Phyllanthaceae)

*Piper betle* (Piperaceae)

*Piper nigrum* (Piperaceae)

*Sesbania grandiflora* (Fabaceae)

**Numbness**

*Mucuna pruriens* (Fabaceae)

*Sinapis alba* (Brassicaceae)

**Nutritional/Nutritive**

*Sesamum indicum* (Pedaliaceae)

**Obesity**

*Amorphophallus paeoniifolius* (Araceae)

*Coix lacryma-jobi* (Poaceae)

**Ointments. *See also* Liniments.**

*Aloe vera* (Asphodelaceae)

*Linum usitatissimum* (Linaceae)

**Ophtalmia**

*Barringtonia acutangula* (Lecythidaceae)

*Cananga odorata* (Annonaceae)

*Symplocos racemosa* (Symplocaceae)

**Orchitus**

*Altingia excelsa* (Altingiaceae)

**Overeating**

*Apium graveolens* (Apiaceae)

**Pain/Ache. *See* also Analgesic.**

*Alstonia scholaris* (Apocynaceae)

*Anacardium occidentale* (Anacardiaceae)

*Aquilaria malaccensis* (Thymelaeaceae)

*Aristolochia indica* (Aristolochiaceae)

*Asclepias curassavica* (Apocynaceae)

*Barleria prionitis* (Acanthaceae)

*Calophyllum inophyllum* (Calophyllaceae)

*Calotropis procera* (Apocynaceae)

*Capparis zeylanica* (Capparaceae)

*Cardiospermum halicacabum* (Sapindaceae)

*Carica papaya* (Caricaceae)

*Centella asiatica* (Apiaceae)

*Cinnamomum bejolghota* (Lauraceae)

*Cinnamomum camphora* (Lauraceae)

*Clerodendrum indicum* (Lamiaceae)

*Combretum indicum* (Combretaceae)

*Crateva religiosa* (Capparaceae)

*Cymbopogon citratus* (Poaceae)

*Fritillaria cirrhosa* (Liliaceae)

*Hydnocarpus kurzii* (Achariaceae)

*Justicia adhatoda* (Acanthaceae)

*Leucaena leucocephala* (Fabaceae)

*Oroxylum indicum* (Bignoniaceae)

*Piper cubeba* (Piperaceae)

*Plantago major* (Plantaginaceae)

*Plumbago zeylanica* (Plumbaginaceae)

*Santalum album* (Santalaceae)

*Semecarpus anacardium* (Anacardiaceae)

*Sesbania grandiflora* (Fabaceae)

*Sesbania sesban* (Fabaceae)

*Vallaris solanacea* (Apocynaceae)

*Zingiber montanum* (Zingiberaceae)

-abdominal

*Morinda angustifolia* (Rubiaceae)

*Ricinus communis* (Euphorbiaceae)

*Sinapis alba* (Brassicaceae)

-ague

*Piper longum* (Piperaceae)

-back

*Canna indica* (Cannaceae)

*Crinum asiaticum* (Amaryllidaceae)

*Datura stramonium* (Solanaceae)

*Piper longum* (Piperaceae)

*Ricinus communis* (Euphorbiaceae)

-bladder

*Arundo donax* (Poaceae)

*Coix lacryma-jobi* (Poaceae)

*Mesua ferrea* (Calophyllaceae)

-body

*Lannea coromandelica* (Anacardiaceae)

-bone

*Datura stramonium* (Solanaceae)

*Convolvulus arvensis* (Convolvulaceae)

-bowel

*Canna indica* (Cannaceae)

-breast

*Alysicarpus vaginalis* (Fabaceae)

*Boerhavia diffusa* (Nyctaginaceae)

*Curcuma longa* (Zingiberaceae)

-chest

*Allium cepa* (Amaryllidaceae)

*Alpinia galanga* (Zingiberaceae)

*Anethum graveolens* (Apiaceae)

*Citrus aurantiifolia* (Rutaceae)

*Curcuma comosa* (Zingiberaceae)

*Dillenia indica* (Dilleniaceae)

*Euphorbia hirta* (Euphorbiaceae)

*Ipomoea alba* (Convolvulaceae)

*Magnolia champaca* (Magnoliaceae)

*Momordica cochinchinensis* (Cucurbitaceae)

*Piper betle* (Piperaceae)

*Piper longum* (Piperaceae)

*Terminalia bellirica* (Combretaceae)

-eye

*Cinnamomum bejolghota* (Lauraceae)

*Datura stramonium* (Solanaceae)

*Mentha arvensis* (Lamiaceae)

*Sinapis alba* (Brassicaceae)

*Terminalia chebula* (Combretaceae)

-flatulence

*Allium cepa* (Amaryllidaceae)

*Andrographis paniculata* (Acanthaceae)

*Curcuma longa* (Zingiberaceae)

*Morinda angustifolia* (Rubiaceae)

*Myristica fragrans* (Myristicaceae)

*Piper nigrum* (Piperaceae)

-gastric

*Acalypha indica* (Euphorbiaceae)

*Magnolia champaca* (Magnoliaceae)

-groin

*Cordyline fruticosa* (Laxmanniaceae)

*Morinda angustifolia* (Rubiaceae)

-heart

*Arundo donax* (Poaceae)

*Coix lacryma-jobi* (Poaceae)

*Coriandrum sativum* (Apiaceae)

*Mesua ferrea* (Calophyllaceae)

-intestinal

*Carica papaya* (Caricaceae)

-joint

*Abrus precatorius* (Fabaceae)

*Acalypha indica* (Euphorbiaceae)

*Aristolochia indica* (Aristolochiaceae)

*Azadirachta indica* (Meliaceae)

*Carthamus tinctorius* (Asteraceae)

*Cascabela thevetia* (Apocynaceae)

*Citrus aurantiifolia* (Rutaceae)

*Convolvulus arvensis* (Convolvulaceae)

*Coriandrum sativum* (Apiaceae)

*Croton persimilis* (Euphorbiaceae)

*Croton tiglium* (Euphorbiaceae)

*Eucalyptus globulus* (Myrtaceae)

*Oroxylum indicum* (Bignoniaceae)

*Orthosiphon aristatus* (Lamiaceae)

*Senna alexandrina* (Fabaceae)

*Sesbania sesban* (Fabaceae)

*Syzygium aromaticum* (Myrtaceae)

*Urena lobata* (Malvaceae)

-menstrual

*Datura stramonium* (Solanaceae)

*Piper betle* (Piperaceae)

*Pogostemon cablin* (Lamiaceae)

-muscle

*Abrus precatorius* (Fabaceae)

*Acorus calamus* (Acoraceae)

*Annona squamosa* (Annonaceae)

*Arundo donax* (Poaceae)

*Carthamus tinctorius* (Asteraceae)

*Coix lacryma-jobi* (Poaceae)

*Holarrhena pubescens* (Apocynaceae)

*Nyctanthes arbor-tristis* (Oleaceae)

-stomach

*Alysicarpus vaginalis* (Fabaceae)

*Aquilaria malaccensis* (Thymelaeaceae)

*Citrus aurantiifolia* (Rutaceae)

*Euphorbia hirta* (Euphorbiaceae)

*Holarrhena pubescens* (Apocynaceae)

*Plantago major* (Plantaginaceae)

*Pogostemon cablin* (Lamiaceae)

*Senna alexandrina* (Fabaceae)

*Tamarindus indica* (Fabaceae)

-tongue

*Cordyline fruticosa* (Laxmanniaceae)

-urinary

*Boerhavia diffusa* (Nyctaginaceae)

*Cassia fistula* (Fabaceae)

*Gossypium barbadense* (Malvaceae)

*Gossypium hirsutum* (Malvaceae)

*Magnolia champaca* (Magnoliaceae)

*Ocimum americanum* (Lamiaceae)

*Santalum album* (Santalaceae)

*Tadehagi triquetrum* (Fabaceae)

*Terminalia bellirica* (Combretaceae)

-uterus

*Arundo donax* (Poaceae)

*Coix lacryma-jobi* (Poaceae)

*Gossypium barbadense* (Malvaceae)

*Gossypium hirsutum* (Malvaceae)

*Terminalia bellirica* (Combretaceae)

**Palpitations**

*Amorphophallus paeoniifolius* (Araceae)

*Aquilaria malaccensis* (Thymelaeaceae)

*Millingtonia hortensis* (Bignoniaceae)

*Oroxylum indicum* (Bignoniaceae)

*Piper nigrum* (Piperaceae)

*Rauvolfia serpentina* (Apocynaceae)

*Tinospora cordifolia* (Menispermaceae)

**Paralysis**

*Acorus calamus* (Acoraceae)

*Alstonia scholaris* (Apocynaceae)

*Boerhavia diffusa* (Nyctaginaceae)

*Blumea balsamifera* (Asteraceae)

*Calotropis procera* (Apocynaceae)

*Cymbopogon nardus* (Poaceae)

*Eryngium caeruleum* (Apiaceae)

*Mucuna pruriens* (Fabaceae)

*Paederia foetida* (Rubiaceae)

*Rauvolfia serpentina* (Apocynaceae)

*Semecarpus anacardium* (Anacardiaceae)

-facial

*Croton tiglium* (Euphorbiaceae)

-partial

*Plumbago indica* (Plumbaginaceae)

-stroke-induced

*Ocimum americanum* (Lamiaceae)

**Parasites. *See also* Ringworms, Roundworms, Threadworms, Worms.**

*Trachyspermum ammi* (Apiaceae)

**Perspiration**

*Allium sativum* (Amaryllidaceae)

*Cinnamomum tamala* (Lauraceae)

*Cyperus scariosus* (Cyperaceae)

*Mesua ferrea* (Calophyllaceae)

*Momordica charantia* (Cucurbitaceae)

*Tamarindus indica* (Fabaceae)

**Phlegm. *See also* Mucus.**

*Acacia concinna* (Fabaceae)

*Aegle marmelos* (Rutaceae)

*Allium cepa* (Amaryllidaceae)

*Allium sativum* (Amaryllidaceae)

*Alpinia galanga* (Zingiberaceae)

*Andrographis paniculata* (Acanthaceae)

*Arundo donax* (Poaceae)

*Asparagus officinalis* (Asparagaceae)

*Azadirachta indica* (Meliaceae)

*Boerhavia diffusa* (Nyctaginaceae)

*Calophyllum inophyllum* (Calophyllaceae)

*Calotropis procera* (Apocynaceae)

*Carica papaya* (Caricaceae)

*Carthamus tinctorius* (Asteraceae)

*Cassia fistula* (Fabaceae)

*Catharanthus roseus* (Apocynaceae)

*Centella asiatica* (Apiaceae)

*Coccinia grandis* (Crassulaceae)

*Coix lacryma-jobi* (Poaceae)

*Coptis teeta* (Ranunculaceae)

*Crinum asiaticum* (Amaryllidaceae)

*Croton persimilis* (Euphorbiaceae)

*Croton tiglium* (Euphorbiaceae)

*Curcuma comosa* (Zingiberaceae)

*Cyperus scariosus* (Cyperaceae)

*Cymbopogon citratus* (Poaceae)

*Dillenia indica* (Dilleniaceae)

*Euphorbia hirta* (Euphorbiaceae)

*Ficus religiosa* (Moraceae)

*Foeniculum vulgare* (Apiaceae)

*Gloriosa superba* (Colchicaceae)

*Gossypium barbadense* (Malvaceae)

*Gossypium hirsutum* (Malvaceae)

*Holarrhena pubescens* (Apocynaceae)

*Justicia adhatoda* (Acanthaceae)

*Magnolia champaca* (Magnoliaceae)

*Mansonia gagei* (Malvaceae)

*Mentha arvensis* (Lamiaceae)

*Mesua ferrea* (Calophyllaceae)

*Mimosa pudica* (Fabaceae)

*Ocimum americanum* (Lamiaceae)

*Piper betle* (Piperaceae)

*Piper cubeba* (Piperaceae)

*Piper nigrum* (Piperaceae)

*Plumbago zeylanica* (Plumbaginaceae)

*Plumeria rubra* (Apocynaceae)

*Ricinus communis* (Euphorbiaceae)

*Semecarpus anacardium* (Anacardiaceae)

*Senna alexandrina* (Fabaceae)

*Sinapis alba* (Brassicaceae)

*Solanum anguivi* (Solanaceae)

*Tectona grandis* (Lamiaceae)

*Tinospora cordifolia* (Menispermaceae)

*Trichosanthes tricuspidata* (Cucurbitaceae)

*Urena lobata* (Malvaceae)

*Zingiber officinale* (Zingiberaceae)

**Piles. *See* Hemorrhoids.**

**Plague**

*Ficus religiosa* (Moraceae)

**Pleurisy**

*Acalypha indica* (Euphorbiaceae)

*Boerhavia diffusa* (Nyctaginaceae)

*Urena lobata* (Malvaceae)

**Pneumonitis**

*Cinnamomum bejolghota* (Lauraceae)

*Coptis teeta* (Ranunculaceae)

*Croton persimilis* (Euphorbiaceae)

**Poison**

*Acacia farnesiana* (Fabaceae)

*Acorus calamus* (Acoraceae)

*Alpinia galanga* (Zingiberaceae)

*Andrographis paniculata* (Acanthaceae)

*Aristolochia indica* (Aristolochiaceae)

*Asparagus officinalis* (Asparagaceae)

*Basella alba* (Basellaceae)

*Centella asiatica* (Apiaceae)

*Cinnamomum bejolghota* (Lauraceae)

*Clausena excavata* (Rutaceae)

*Crinum asiaticum* (Amaryllidaceae)

*Curcuma comosa* (Zingiberaceae)

*Eucalyptus globulus* (Myrtaceae)

*Hydnocarpus kurzii* (Achariaceae)

*Ipomoea aquatica* (Convolvulaceae)

*Leucaena leucocephala* (Fabaceae)

*Limonia acidissima* (Rutaceae)

*Mesua ferrea* (Calophyllaceae)

*Moringa oleifera* (Moringaceae)

*Piper betle* (Piperaceae)

*Piper cubeba* (Piperaceae)

*Plantago major* (Plantaginaceae)

*Rauvolfia serpentina* (Apocynaceae)

*Sesbania grandiflora* (Fabaceae)

*Sesbania sesban* (Fabaceae)

*Strychnos potatorum* (Loganiaceae)

*Terminalia bellirica* (Combretaceae)

*Tinospora cordifolia* (Menispermaceae)

*Vitex trifolia* (Lamiaceae)

*Zingiber officinale* (Zingiberaceae)

-alcohol

*Citrus aurantiifolia* (Rutaceae)

*Mayodendron igneum* (Bignoniaceae)

*Tecoma stans* (Bignoniaceae)

-animal

*Nerium oleander* (Apocynaceae)

*Piper longum* (Piperaceae)

*Tamarindus indica* (Fabaceae)

-arsenic

*Cinnamomum camphora* ((Lauraceae)

-centipede

*Mucuna pruriens* (Fabaceae)

*Solanum anguivi* (Solanaceae)

-food/medicines

*Citrus aurantiifolia* (Rutaceae)

*Cyperus scariosus* (Cyperaceae)

*Euonymus kachinensis* (Celastraceae)

-opium

*Celastrus paniculatus* (Celastraceae)

*Citrus aurantiifolia* (Rutaceae)

*Litchi chinensis* (Sapindaceae)

-rat

*Dregea volubilis* (Apocynaceae)

-scorpion

*Allium cepa* (Amaryllidaceae)

*Amaranthus spinosus* (Amaranthaceae)

*Aristolochia indica* (Aristolochiaceae)

*Barleria prionitis* (Acanthaceae)

*Bryophyllum pinnatum* (Crassulaceae)

*Calotropis procera* (Apocynaceae)

*Carica papaya* (Caricaceae)

*Carthamus tinctorius* (Asteraceae)

*Cinnamomum bejolghota* (Lauraceae)

*Cinnamomum camphora* (Lauraceae)

*Croton tiglium* (Euphorbiaceae)

*Cyperus scariosus* (Cyperaceae)

*Euonymus kachinensis* (Celastraceae)

*Gossypium barbadense* (Malvaceae)

*Gossypium hirsutum* (Malvaceae)

*Justicia adhatoda* (Acanthaceae)

*Mucuna pruriens* (Fabaceae)

*Nerium oleander* (Apocynaceae)

*Ricinus communis* (Euphorbiaceae)

*Sesbania grandiflora* (Fabaceae)

*Sesbania sesban* (Fabaceae)

*Syzygium cumini* (Myrtaceae)

*Tamarindus indica* (Fabaceae)

*Ziziphus jujuba* (Rhamnaceae)

-snake

*Acacia pennata* (Fabaceae)

*Amaranthus spinosus* (Amaranthaceae)

*Aristolochia indica* (Aristolochiaceae)

*Butea monosperma* (Fabaceae)

*Butea superba* (Fabaceae)

*Calotropis procera* (Apocynaceae)

*Carthamus tinctorius* (Asteraceae)

*Clitoria ternatea* (Fabaceae)

*Coptis teeta* (Ranunculaceae)

*Croton persimilis* (Euphorbiaceae)

*Cyperus scariosus* (Cyperaceae)

*Euonymus kachinensis* (Celastraceae)

*Leucaena leucocephala* (Fabaceae)

*Leucas cephalotes* (Lamiaceae)

*Limonia acidissima* (Rutaceae)

*Nerium oleander* (Apocynaceae)

*Nyctanthes arbor-tristis* (Oleaceae)

*Ocimum americanum* (Lamiaceae)

*Peristrophe bicalyculata* (Acanthaceae)

*Solanum anguivi* (Solanaceae)

*Strobilanthes auriculatus* (Acanthaceae)

*Tamarindus indica* (Fabaceae)

-spiders

*Cinnamomum bejolghota* (Lauraceae)

-stomach

*Limonia acidissima* (Rutaceae)

**Poultice**

*Abelmoschus moschatus* (Malvaceae)

*Abrus precatorius* (Fabaceae)

*Adenanthera pavonina* (Fabaceae)

*Aloe vera* (Asphodelaceae)

*Alstonia scholaris* (Apocynaceae)

*Amaranthus spinosus* (Amaranthaceae)

*Annona squamosa* (Annonaceae)

*Avicennia officinalis* (Acanthaceae)

*Bambusa bambos* (Poaceae)

*Bauhinia acuminata* (Fabaceae)

*Cassia fistula* (Fabaceae)

*Croton persimilis* (Euphorbiaceae)

*Datura stramonium* (Solanaceae)

*Dregea volubilis* (Apocynaceae)

*Euonymus kachinensis* (Celastraceae)

*Ficus hispida* (Moraceae)

*Hygrophila phlomoides* (Acanthaceae)

*Kopsia fruticosa* (Apocynaceae)

*Morinda coreia* (Rubiaceae)

*Ricinus communis* (Euphorbiaceae)

*Semecarpus anacardium* (Anacardiaceae)

*Sesbania sesban* (Fabaceae)

*Terminalia chebula* (Combretaceae)

*Zingiber montanum* (Zingiberaceae)

**Pox**

*Carallia brachiata* (Rhizophoraceae)

**Prolapsus uteri**

*Cissampelos pareira* (Menispermaceae)

**Psora**

*Azadirachta indica* (Meliaceae)

*Markhamia stipulata* (Bignoniaceae)

**Purgative. *See also* Carthatic, Hydragogue, Laxative.**

*Abrus precatorius* (Fabaceae)

*Agave sisalana* (Asparagaceae)

*Agave vera-cruz* (Asparagaceae)

*Artocarpus lakoocha* (Moraceae)

*Asclepias curassavica* (Apocynaceae)

*Caesalpinia pulcherrima* (Fabaceae)

*Calotropis gigantea* (Apocynaceae)

*Cardiospermum halicacabum* (Sapindaceae)

*Carissa spinarum* (Apocynaceae)

*Cascabela thevetia* (Apocynaceae)

*Cassia fistula* (Fabaceae)

*Claoxylon indicum* (Euphorbiaceae)

*Clitoria ternatea* (Fabaceae)

*Cordia dichotoma* (Boraginaceae)

*Croton persimilis* (Euphorbiaceae)

*Cynometra ramiflora* (Fabaceae)

*Euphorbia antiquorum* (Euphorbiaceae)

*Jatropha gossypiifolia* (Euphorbiaceae)

*Lagerstroemia speciosa* (Lythraceae)

*Limonia acidissima* (Rutaceae)

*Momordica charantia* (Cucurbitaceae)

*Mucuna pruriens* (Fabaceae)

*Phyllanthus acidus* (Phyllanthaceae)

*Sambucus javanica* (Adoxaceae)

**Pus**

*Alysicarpus vaginalis* (Fabaceae)

*Azadirachta indica* (Meliaceae)

*Dregea volubilis* (Apocynaceae)

**Pyexia**

*Croton persimilis* (Euphorbiaceae)

**Rabies**

*Datura stramonium* (Solanaceae)

*Dillenia indica* (Dilleniaceae)

*Dregea volubilis* (Apocynaceae)

**Rashes**

*Acalypha indica* (Euphorbiaceae)

*Azadirachta indica* (Meliaceae)

*Butea monosperma* (Fabaceae)

*Cardiospermum halicacabum* (Sapindaceae)

*Carica papaya* (Caricaceae)

*Carthamus tinctorius* (Asteraceae)

*Cascabela thevetia* (Apocynaceae)

*Centella asiatica* (Apiaceae)

*Citrus aurantiifolia* (Rutaceae)

*Convolvulus arvensis* (Convolvulaceae)

*Curcuma longa* (Zingiberaceae)

*Cuscuta reflexa* (Convolvulaceae)

*Cymbopogon nardus* (Poaceae)

*Ficus religiosa* (Moraceae)

*Mimusops elengi* (Sapotaceae)

*Moringa oleifera* (Moringaceae)

*Nyctanthes arbor-tristis* (Oleaceae)

*Phyllanthus emblica* (Phyllanthaceae)

*Semecarpus anacardium* (Anacardiaceae)

*Sinapis alba* (Brassicaceae)

*Solanum anguivi* (Solanaceae)

*Syzygium aromaticum* (Myrtaceae)

*Tamarindus indica* (Fabaceae)

*Tectona grandis* (Lamiaceae)

*Terminalia bellirica* (Combretaceae)

*Trachyspermum ammi* (Apiaceae)

**Renal complaints**

*Butea monosperma* (Fabaceae)

*Wrightia arborea* (Apocynaceae)

**Respiratory function**

*Alstonia scholaris* (Apocynaceae)

*Annona squamosa* (Annonaceae)

*Clerodendrum indicum* (Lamiaceae)

*Eclipta prostrata* (Asteraceae)

*Plumeria rubra* (Apocynaceae)

*Semecarpus anacardium* (Anacardiaceae)

*Ziziphus jujuba* (Rhamnaceae)

**Restlessness**

*Sesbania grandiflora* (Fabaceae)

**Restorative**

*Anacardium occidentale* (Anacardiaceae)

*Anethum graveolens* (Apiaceae)

*Ceiba pentandra* (Malvaceae)

**Rheumatism**

*Acanthus ilicifolius* (Acanthaceae)

*Aglaia cucullata* (Meliaceae)

*Aquilaria malaccensis* (Thymelaeaceae)

*Cardiospermum halicacabum* (Sapindaceae)

*Cymbopogon jwarancusa* (Poaceae)

*Holoptelea integrifolia* (Ulmaceae)

*Hygrophila auriculata* (Acanthaceae)

*Oroxylum indicum* (Bignoniaceae)

*Momordica charantia* (Cucurbitaceae)

*Premna serratifolia* (Lamiaceae)

*Quassia indica* (Simaroubaceae)

*Syzygium nervosum* (Myrtaceae)

*Urena lobata* (Malvaceae)

*Verbena officinalis* (Verbenaceae)

*Vitex trifolia* (Lamiaceae)

*Xylia xylocarpa* (Fabaceae)

**Ringworm**

*Acalypha indica* (Euphorbiaceae)

*Allium sativum* (Amaryllidaceae)

*Barleria prionitis* (Acanthaceae)

*Boerhavia diffusa* (Nyctaginaceae)

*Butea monosperma* (Fabaceae)

*Cardiospermum halicacabum* (Sapindaceae)

*Carica papaya* (Caricaceae)

*Cascabela thevetia* (Apocynaceae)

*Cassia fistula* (Fabaceae)

*Citrus aurantiifolia* (Rutaceae)

*Euphorbia hirta* (Euphorbiaceae)

*Jasminum humile* (Oleaceae)

*Nerium oleander* (Apocynaceae)

*Nyctanthes arbor-tristis* (Oleaceae)

*Ocimum americanum* (Lamiaceae)

*Phyllanthus emblica* (Phyllanthaceae)

*Plumbago zeylanica* (Plumbaginaceae)

*Semecarpus anacardium* (Anacardiaceae)

*Senna alata* (Fabaceae)

*Sinapis alba* (Brassicaceae)

**Roundworm**

*Gloriosa superba* (Colchicaceae)

*Magnolia champaca* (Magnoliaceae)

**Rubefacient**

*Capsicum annuum* (Solanaceae)

*Cleome gynandra* (Cleomaceae)

*Maranta arundinacea* (Marantaceae)

**Runny noses**

*Cinnamomum bejolghota* (Lauraceae)

*Euphorbia hirta* (Euphorbiaceae)

*Piper betle* (Piperaceae)

*Piper nigrum* (Piperaceae)

*Trichosanthes tricuspidata* (Cucurbitaceae)

*Zingiber officinale* (Zingiberaceae)

**Sagging belly**

*Amorphophallus paeoniifolius* (Araceae)

**Scabies**

*Acalypha indica* (Euphorbiaceae)

*Allium sativum* (Amaryllidaceae)

*Azadirachta indica* (Meliaceae)

*Cardiospermum halicacabum* (Sapindaceae)

*Cassia fistula* (Fabaceae)

*Cordyline fruticosa* (Laxmanniaceae)

*Croton persimilis* (Euphorbiaceae)

*Cymbopogon nardus* (Poaceae)

*Cyperus scariosus* (Cyperaceae)

*Euphorbia hirta* (Euphorbiaceae)

*Mesua ferrea* (Calophyllaceae)

*Ocimum americanum* (Lamiaceae)

*Plumbago indica* (Plumbaginaceae)

*Plumbago zeylanica* (Plumbaginaceae)

*Senna alata* (Fabaceae)

*Tadehagi triquetrum* (Fabaceae)

**Scalp, flaky**

*Santalum album* (Santalaceae)

*Tadehagi triquetrum* (Fabaceae)

**Scrofula**

*Volkameria inermis* (Lamiaceae)

**Sedative**

*Brugmansia arborea* (Solanaceae)

*Brugmansia suaveolens* (Solanaceae)

*Cannabis sativa* (Cannabaceae)

*Datura metel* (Solanaceae)

*Datura stramonium* (Solanaceae)

*Flemingia chappar* (Fabaceae)

*Phyllanthus emblica* (Phyllanthaceae)

*Rauvolfia serpentina* (Apocynaceae)

*Taxus baccata* (Taxaceae)

*Vitex negundo* (Lamiaceae)

**Semen**

-control

*Myristica fragrans* (Myristicaceae)

-excessive

*Rauvolfia serpentina* (Apocynaceae)

-increase

*Apium graveolens* (Apiaceae)

*Butea monosperma* (Fabaceae)

*Euphorbia hirta* (Euphorbiaceae)

*Magnolia champaca* (Magnoliaceae)

*Mucuna pruriens* (Fabaceae)

*Ricinus communis* (Euphorbiaceae)

*Urena lobata* (Malvaceae)

**Sexual functioning**

*Annona squamosa* (Annonaceae)

-impotence

*Allium cepa* (Amaryllidaceae)

*Allium sativum* (Amaryllidaceae)

*Arundo donax* (Poaceae)

*Canna indica* (Cannaceae)

*Cuscuta reflexa* (Convolvulaceae)

*Mirabilis jalapa* (Nyctaginaceae)

*Ocimum americanum* (Lamiaceae)

*Rauvolfia serpentina* (Apocynaceae)

*Sesbania grandiflora* (Fabaceae)

*Terminalia bellirica* (Combretaceae)

-lack of semen

*Cinnamomum tamala* (Lauraceae)

*Leucas cephalotes* (Lamiaceae)

*Rauvolfia serpentina* (Apocynaceae)

**Sickness, general**

*Nyctanthes arbor-tristis* (Oleaceae)

*Piper betle* (Piperaceae)

**Sinusitis**

*Annona squamosa* (Annonaceae)

*Cordyline fruticosa* (Laxmanniaceae)

*Sinapis alba* (Brassicaceae)

**Skin condition, support**

*Alstonia scholaris* (Apocynaceae)

*Mesua ferrea* (Calophyllaceae)

**Skin diseases. *See also individual diseases.***

*Acacia concinna* (Fabaceae)

*Ageratum conyzoides* (Asteraceae)

*Allium sativum* (Amaryllidaceae)

*Aloe vera* (Asphodelaceae)

*Anacardium occidentale* (Anacardiaceae)

*Aquilaria malaccensis* (Thymelaeaceae)

*Argemone mexicana* (Papaveraceae)

*Azadirachta indica* (Meliaceae)

*Barleria prionitis* (Acanthaceae)

*Brassica oleracea* (Brassicaceae)

*Buddleja asiatica* (Scrophulariaceae)

*Callicarpa macrophylla* (Lamiaceae)

*Calophyllum inophyllum* (Calophyllaceae)

*Calotropis gigantea* (Apocynaceae)

*Calotropis procera* (Apocynaceae)

*Carica papaya* (Caricaceae)

*Cascabela thevetia* (Apocynaceae)

*Cassia fistula* (Fabaceae)

*Centella asiatica* (Apiaceae)

*Colocasia antiquorum* (Araceae)

*Cycas rumphii* (Cycadaceae)

*Eclipta prostrata* (Asteraceae)

*Enydra fluctuans* (Asteraceae)

*Euphorbia hirta* (Euphorbiaceae)

*Ficus religiosa* (Moraceae)

*Fritillaria cirrhosa* (Liliaceae)

*Gossypium barbadense* (Malvaceae)

*Gossypium hirsutum* (Malvaceae)

*Grewia asiatica* (Malvaceae)

*Grewia nervosa* (Malvaceae)

*Hydnocarpus kurzii* (Achariaceae)

*Jasminum humile* (Oleaceae)

*Jatropha gossypiifolia* (Euphorbiaceae)

*Mesua ferrea* (Calophyllaceae)

*Nyctanthes arbor-tristis* (Oleaceae)

*Ocimum americanum* (Lamiaceae)

*Phyllanthus emblica* (Phyllanthaceae)

*Plantago major* (Plantaginaceae)

*Plumbago indica* (Plumbaginaceae)

*Plumbago zeylanica* (Plumbaginaceae)

*Pterospermum acerifolium* (Malvaceae)

*Sapindus saponaria* (Sapindaceae)

*Senna alata* (Fabaceae)

*Sesbania grandiflora* (Fabaceae)

*Sesbania sesban* (Fabaceae)

*Sigesbeckia orientalis* (Asteraceae)

*Sinapis alba* (Brassicaceae)

*Tadehagi triquetrum* (Fabaceae)

*Tectona grandis* (Lamiaceae)

*Urena lobata* (Malvaceae)

*Vaccaria hispanica* (Caryophyllaceae)

*Vitex trifolia* (Lamiaceae)

*Zingiber montanum* (Zingiberaceae)

-discoloration

*Allium sativum* (Amaryllidaceae)

*Aristolochia indica* (Aristolochiaceae)

*Calotropis procera* (Apocynaceae)

*Cardiospermum halicacabum* (Sapindaceae)

*Citrus aurantiifolia* (Rutaceae)

*Hygrophila phlomoides* (Acanthaceae)

-from blood impurities

*Acalypha indica* (Euphorbiaceae)

*Cassia fistula* (Fabaceae)

*Sesbania sesban* (Fabaceae)

-fungal infection

*Hygrophila phlomoides* (Acanthaceae)

*Phyllanthus emblica* (Phyllanthaceae)

-pimples

*Euphorbia hirta* (Euphorbiaceae)

*Myristica fragrans* (Myristicaceae)

*Syzygium aromaticum* (Myrtaceae)

*Terminalia chebula* (Combretaceae)

**Skin sores**

*Acacia farnesiana* (Fabaceae)

*Acalypha indica* (Euphorbiaceae)

*Aegle marmelos* (Rutaceae)

*Amaranthus spinosus* (Amaranthaceae)

*Aquilaria malaccensis* (Thymelaeaceae)

*Butea monosperma* (Fabaceae)

*Cardiospermum halicacabum* (Sapindaceae)

*Cinnamomum bejolghota* (Lauraceae)

*Convolvulus arvensis* (Convolvulaceae)

*Crateva religiosa* (Capparaceae)

*Cuscuta reflexa* (Convolvulaceae)

*Diospyros malabarica* (Ebenaceae)

*Eclipta prostrata* (Asteraceae)

*Eucalyptus globulus* (Myrtaceae)

*Ficus religiosa* (Moraceae)

*Gouania leptostachya* (Rhamnaceae)

*Ipomoea aquatica* (Convolvulaceae)

*Limonia acidissima* (Rutaceae)

*Magnolia champaca* (Magnoliaceae)

*Mirabilis jalapa* (Nyctaginaceae)

*Morinda coreia* (Rubiaceae)

*Moringa oleifera* (Moringaceae)

*Phyllanthus emblica* (Phyllanthaceae)

*Picrasma javanica* (Simaroubaceae)

*Rotheca serrata* (Lamiaceae)

*Sesbania sesban* (Fabaceae)

*Syzygium aromaticum* (Myrtaceae)

*Tadehagi triquetrum* (Fabaceae)

*Tamarindus indica* (Fabaceae)

*Tinospora cordifolia* (Menispermaceae)

*Urena lobata* (Malvaceae)

*Vitex trifolia* (Lamiaceae)

-cold

*Coccinia grandis* (Crassulaceae)

-inflamed sores

*Mimosa pudica* (Fabaceae)

-leprous

*Solanum anguivi* (Solanaceae)

-lesions

*Cycas rumphii* (Cycadaceae)

-remove maggots from,

*Aquilaria malaccensis* (Thymelaeaceae)

*Haldina cordifolia* (Rubiaceae)

-warts

*Euphorbia antiquorum* (Euphorbiaceae)

**Sleep disorders. *See also* Insomnia, Restlessness, Soporific.**

*Fritillaria cirrhosa* (Liliaceae)

*Curcuma longa* (Zingiberaceae)

*Centella asiatica* (Apiaceae)

**Smallpox**

*Aquilaria malaccensis* (Thymelaeaceae)

*Eclipta prostrata* (Asteraceae)

*Elettaria cardamomum* (Zingiberaceae)

*Enydra fluctuans* (Asteraceae)

*Holarrhena pubescens* (Apocynaceae)

*Pterospermum acerifolium* (Malvaceae)

**Soporific**

*Allium sativum* (Amaryllidaceae)

*Amaranthus cruentus* (Amaranthaceae)

*Amaranthus spinosus* (Amaranthaceae)

*Fritillaria cirrhosa* (Liliaceae)

*Rauvolfia serpentina* (Apocynaceae)

*Vitex trifolia* (Lamiaceae)

**Sore. *See also* Pain.**

*Calophyllum inophyllum* (Calophyllaceae)

*Capparis zeylanica* (Capparaceae)

*Terminalia citrina* (Combretaceae)

-eyes

*Cardiospermum halicacabum* (Sapindaceae)

*Clitoria ternatea* (Fabaceae)

*Coptis teeta* (Ranunculaceae)

*Datura stramonium* (Solanaceae)

*Millingtonia hortensis* (Bignoniaceae)

*Piper betle* (Piperaceae)

*Syzygium aromaticum* (Myrtaceae)

*Syzygium cumini* (Myrtaceae)

*Syzygium jambos* (Myrtaceae)

*Tamarindus indica* (Fabaceae)

-gums

*Aegle marmelos* (Rutaceae)

-joints

*Plumbago indica* (Plumbaginaceae)

-muscles

*Limonia acidissima* (Rutaceae)

**Sores. *See* Skin Sores.**

**Sore throat. *See* Throat, Sore.**

**Spasmolytic. *See* Antispasmodic.**

**Speech improvement**

*Alpinia officinarum* (Zingiberaceae)

**Spleen**

-diseases

*Mentha arvensis* (Lamiaceae)

*Nyctanthes arbor-tristis* (Oleaceae)

*Vitex trifolia* (Lamiaceae)

-enlargement

*Carica papaya* (Caricaceae)

*Sinapis alba* (Brassicaceae)

*Sesbania grandiflora* (Fabaceae)

*Terminalia bellirica* (Combretaceae)

**Splinters, remove**

*Alstonia scholaris* (Apocynaceae)

**Sternutative**

*Ipomoea hederifolia* (Convolvulaceae)

**Stiffness**

*Alpinia officinarum* (Zingiberaceae)

*Andrographis paniculata* (Acanthaceae)

*Annona squamosa* (Annonaceae)

*Arundo donax* (Poaceae)

*Calotropis procera* (Apocynaceae)

*Canna indica* (Cannaceae)

*Coix lacryma-jobi* (Poaceae)

-muscle

*Amaranthus spinosus* (Amaranthaceae)

*Carica papaya* (Caricaceae)

*Morinda coreia* (Rubiaceae)

-neck

*Dregea volubilis* (Apocynaceae)

*Piper longum* (Piperaceae)

*Sinapis alba* (Brassicaceae)

**Stimulant**

*Abelmoschus moschatus* (Malvaceae)

*Allium cepa* (Amaryllidaceae)

*Anethum graveolens* (Apiaceae)

*Aquilaria malaccensis* (Thymelaeaceae)

*Ardisia humilis* (Primulaceae)

*Azadirachta indica* (Meliaceae)

*Celastrus paniculatus* (Celastraceae)

*Chloranthus elatior* (Chloranthaceae)

*Cinnamomum camphora* (Lauraceae)

*Cycas rumphii* (Cycadaceae)

*Euonymus kachinensis* (Celastraceae)

*Melaleuca cajuputi* (Myrtaceae)

*Moringa oleifera* (Moringaceae)

*Salvia officinalis* (Lamiaceae)

*Sigesbeckia orientalis* (Asteraceae)

*Solanum melongena* (Solanaceae)

*Syzygium aromaticum* (Myrtaceae)

-brain

*Dimocarpus longan* (Sapindaceae)

**Sting. *See also* Bite, Poison.**

*Commelina paludosa* (Commelinaceae)

*Euonymus kachinensis* (Celastraceae)

*Mesua ferrea* (Calophyllaceae)

*Plantago major* (Plantaginaceae)

*Senna alata* (Fabaceae)

**Stomachache**

*Acacia concinna* (Fabaceae)

*Acalypha indica* (Euphorbiaceae)

*Acorus calamus* (Acoraceae)

*Allium sativum* (Amaryllidaceae)

*Alpinia galanga* (Zingiberaceae)

*Alstonia scholaris* (Apocynaceae)

*Apium graveolens* (Apiaceae)

*Aquilaria malaccensis* (Thymelaeaceae)

*Aristolochia indica* (Aristolochiaceae)

*Calotropis procera* (Apocynaceae)

*Carica papaya* (Caricaceae)

*Croton persimilis* (Euphorbiaceae)

*Curcuma comosa* (Zingiberaceae)

*Cymbopogon nardus* (Poaceae)

*Cyperus scariosus* (Cyperaceae)

*Mentha arvensis* (Lamiaceae)

*Morinda angustifolia* (Rubiaceae)

*Oroxylum indicum* (Bignoniaceae)

*Plumeria rubra* (Apocynaceae)

*Senna siamea* (Fabaceae)

*Sinapis alba* (Brassicaceae)

*Tadehagi triquetrum* (Fabaceae)

*Terminalia bellirica* (Combretaceae)

*Terminalia citrina* (Combretaceae)

*Trachyspermum ammi* (Apiaceae)

**Stomach**

-bloat

*Canna indica* (Cannaceae)

*Cassia fistula* (Fabaceae)

*Cinnamomum bejolghota* (Lauraceae)

*Cinnamomum tamala* (Lauraceae)

*Croton persimilis* (Euphorbiaceae)

*Holarrhena pubescens* (Apocynaceae)

*Mentha arvensis* (Lamiaceae)

*Moringa oleifera* (Moringaceae)

*Oroxylum indicum* (Bignoniaceae)

*Plumeria rubra* (Apocynaceae)

*Ricinus communis* (Euphorbiaceae)

*Sesbania grandiflora* (Fabaceae)

*Sesbania sesban* (Fabaceae)

*Tadehagi triquetrum* (Fabaceae)

-distention

*Apium graveolens* (Apiaceae)

*Cinnamomum bejolghota* (Lauraceae)

*Holarrhena pubescens* (Apocynaceae)

*Mentha arvensis* (Lamiaceae)

*Piper cubeba* (Piperaceae)

*Piper nigrum* (Piperaceae)

-function

*Selinum wallichianum* (Apiaceae)

-in children

*Persicaria pulchra* (Polygonaceae)

-problems

*Alstonia scholaris* (Apocynaceae)

*Clausena excavata* (Rutaceae)

*Croton tiglium* (Euphorbiaceae)

*Mesua ferrea* (Calophyllaceae)

*Monochoria vaginalis* (Pontederiaceae)

*Plumeria rubra* (Apocynaceae)

**Stomachic**

*Abelmoschus esculentus* (Malvaceae)

*Abelmoschus moschatus* (Malvaceae)

*Andrographis paniculata* (Acanthaceae)

*Anethum graveolens* (Apiaceae)

*Azadirachta indica* (Meliaceae)

*Blumea balsamifera* (Asteraceae)

*Callicarpa macrophylla* (Lamiaceae)

*Capparis zeylanica* (Capparaceae)

*Cissampelos pareira* (Menispermaceae)

*Crateva religiosa* (Capparaceae)

*Foeniculum vulgare* (Apiaceae)

*Gmelina arborea* (Lamiaceae)

*Helicteres isora* (Malvaceae)

*Hibiscus schizopetalus* (Malvaceae)

*Hibiscus vitifolius* (Malvaceae)

*Ixora coccinea* (Rubiaceae)

*Lablab purpureus* (Fabaceae)

*Limonia acidissima* (Rutaceae)

*Ocimum tenuiflorum* (Lamiaceae)

*Premna serratifolia* (Lamiaceae)

*Salvia officinalis* (Lamiaceae)

*Syzygium aromaticum* (Myrtaceae)

*Tinospora cordifolia* (Menispermaceae)

*Urtica parviflora* (Urticaceae)

**Stomatitis**

*Alstonia scholaris* (Apocynaceae)

*Flacourtia jangomas* (Salicaceae)

**Strengthener. *See also* Fortifier.**

-blood

*Allium sativum* (Amaryllidaceae)

*Asparagus officinalis* (Asparagaceae)

*Canna indica* (Cannaceae)

*Carallia brachiata* (Rhizophoraceae)

*Dregea volubilis* (Apocynaceae)

-gall bladder

*Allium sativum* (Amaryllidaceae)

*Asparagus officinalis* (Asparagaceae)

-heart

*Aquilaria malaccensis* (Thymelaeaceae)

*Myristica fragrans* (Myristicaceae)

*Solanum anguivi* (Solanaceae)

-kidney

*Mentha arvensis* (Lamiaceae)

-stomach

*Aquilaria malaccensis* (Thymelaeaceae)

*Senna sulfurea* (Fabaceae)

-teeth

*Azadirachta indica* (Meliaceae)

*Morinda coreia* (Rubiaceae)

**Styptic. *See* Astringent.**

**Stroke**

*Benincasa hispida* (Cucurbitaceae)

*Calotropis procera* (Apocynaceae)

**Stupor, induction**

*Mitragyna speciosa* (Rubiaceae)

**Sudorific. *See also* Diaphoretic.**

*Asclepias curassavica* (Apocynaceae)

*Limonia acidissima* (Rutaceae)

*Plumbago zeylanica* (Plumbaginaceae)

*Zanthoxylum acanthopodium* (Rutaceae)

**Swelling**

*Abelmoschus moschatus* (Malvaceae)

*Barleria prionitis* (Acanthaceae)

*Dioscorea pentaphylla* (Dioscoreaceae)

*Dregea volubilis* (Apocynaceae)

*Justicia adhatoda* (Acanthaceae)

*Lannea coromandelica* (Anacardiaceae)

*Tectona grandis* (Lamiaceae)

*Terminalia bellirica* (Combretaceae)

*Xylocarpus granatum* (Meliaceae)

*Xylocarpus moluccensis* (Meliaceae)

-abdominal

*Andrographis paniculata* (Acanthaceae)

-ankle

*Zingiber montanum* (Zingiberaceae)

-joint

*Abrus precatorius* (Fabaceae)

*Acorus calamus* (Acoraceae)

*Aquilaria malaccensis* (Thymelaeaceae)

*Girardinia diversifolia* (Urticaceae)

*Morinda coreia* (Rubiaceae)

*Sesbania sesban* (Fabaceae)

*Zingiber montanum* (Zingiberaceae)

*Zingiber officinale* (Zingiberaceae)

-knee

*Crinum asiaticum* (Amaryllidaceae)

*Zingiber montanum* (Zingiberaceae)

-penis

*Convolvulus arvensis* (Convolvulaceae)

-windpipe

*Acalypha indica* (Euphorbiaceae)

**Syphilis. *See also* Antisyphilitic, Venereal Disease.**

*Cyperus scariosus* (Cyperaceae)

-rheumatism caused by

*Clerodendrum indicum* (Lamiaceae)

**Taste, loss of**

*Syzygium aromaticum* (Myrtaceae)

**Testes enlargement**

*Acacia pennata* (Fabaceae)

*Clitoria ternatea* (Fabaceae)

*Gossypium barbadense* (Malvaceae)

*Gossypium hirsutum* (Malvaceae)

*Ricinus communis* (Euphorbiaceae)

**Tetanus**

*Ficus religiosa* (Moraceae)

**Thirst**

*Cyperus scariosus* (Cyperaceae)

*Gossypium barbadense* (Malvaceae)

*Gossypium hirsutum* (Malvaceae)

*Ipomoea aquatica* (Convolvulaceae)

*Mentha arvensis* (Lamiaceae)

*Strychnos potatorum* (Loganiaceae)

**Thorn remover**

*Alstonia scholaris* (Apocynaceae)

*Plantago major* (Plantaginaceae)

**Threadworms**

*Annona squamosa* (Annonaceae)

*Cinnamomum bejolghota* (Lauraceae)

*Gloriosa superba* (Colchicaceae)

*Magnolia champaca* (Magnoliaceae)

**Throat**

*Cardiospermum halicacabum* (Sapindaceae)

*Clitoria ternatea* (Fabaceae)

*Sesbania grandiflora* (Fabaceae)

*Sesbania sesban* (Fabaceae)

-blisters

*Aristolochia indica* (Aristolochiaceae)

**Throat, sore**

*Abrus precatorius* (Fabaceae)

*Acalypha indica* (Euphorbiaceae)

*Acorus calamus* (Acoraceae)

*Bauhinia acuminata* (Fabaceae)

*Canna indica* (Cannaceae)

*Citrus aurantiifolia* (Rutaceae)

*Coriandrum sativum* (Apiaceae)

*Cymbopogon citratus* (Poaceae)

*Dregea volubilis* (Apocynaceae)

*Elettaria cardamomum* (Zingiberaceae)

*Mentha arvensis* (Lamiaceae)

*Moringa oleifera* (Moringaceae)

*Oroxylum indicum* (Bignoniaceae)

*Ricinus communis* (Euphorbiaceae)

*Salvia officinalis* (Lamiaceae)

*Terminalia bellirica* (Combretaceae)

*Trichosanthes tricuspidata* (Cucurbitaceae)

**Thrush**

*Cardiospermum halicacabum* (Sapindaceae)

*Mimusops elengi* (Sapotaceae)

**Tightness**

*Claoxylon indicum* (Euphorbiaceae)

**Tongue**,

-cracked

*Ficus religiosa* (Moraceae)

-sores

*Acacia pennata* (Fabaceae)

**Tonic**

*Abelmoschus moschatus* (Malvaceae)

*Andrographis paniculata* (Acanthaceae)

*Anethum graveolens* (Apiaceae)

*Annona squamosa* (Annonaceae)

*Aquilaria malaccensis* (Thymelaeaceae)

*Aristolochia tagala* (Aristolochiaceae)

*Artemisia dracunculus* (Asteraceae)

*Azadirachta indica* (Meliaceae)

*Bombax ceiba* (Malvaceae)

*Butea monosperma* (Fabaceae)

*Canavalia ensiformis* (Fabaceae)

*Ceiba pentandra* (Malvaceae)

*Centella asiatica* (Apiaceae)

*Cissampelos pareira* (Menispermaceae)

*Cyanthillium cinereum* (Asteraceae)

*Datura stramonium* (Solanaceae)

*Eclipta prostrata* (Asteraceae)

*Elephantopus scaber* (Asteraceae)

*Eryngium caeruleum* (Apiaceae)

*Evolvulus alsinoides* (Convolvulaceae)

*Glycine max* (Fabaceae)

*Heynea trijuga* (Meliaceae)

*Hymenodictyon orixense* (Rubiaceae)

*Ichnocarpus frutescens* (Apocynaceae)

*Lantana × aculeata* (Verbenaceae)

*Mangifera indica* (Anacardiaceae)

*Manilkara zapota* (Sapotaceae)

*Mesua ferrea* (Calophyllaceae)

*Mucuna pruriens* (Fabaceae)

*Myristica fragrans* (Myristicaceae)

*Nauclea orientalis* (Rubiaceae)

*Oroxylum indicum* (Bignoniaceae)

*Piper cubeba* (Piperaceae)

*Pterospermum acerifolium* (Malvaceae)

*Rauvolfia serpentina* (Apocynaceae)

*Rubia cordifolia* (Rubiaceae)

*Semecarpus anacardium* (Anacardiaceae)

*Senna siamea* (Fabaceae)

*Sida spinosa* (Malvaceae)

*Swertia chirayita* (Gentianaceae)

*Syzygium aromaticum* (Myrtaceae)

*Tabernaemontana divaricata* (Apocynaceae)

*Tamarindus indica* (Fabaceae)

*Terminalia bellirica* (Combretaceae)

*Terminalia chebula* (Combretaceae)

-brain

*Litchi chinensis* (Sapindaceae)

-heart

*Antiaris toxicaria* (Moraceae)

*Cascabela thevetia* (Apocynaceae)

*Digitalis lanata* (Plantaginaceae)

*Digitalis purpurea* (Plantaginaceae)

*Litchi chinensis* (Sapindaceae)

-liver

*Litchi chinensis* (Sapindaceae)

**Toothache**

*Acalypha indica* (Euphorbiaceae)

*Alstonia scholaris* (Apocynaceae)

*Azadirachta indica* (Meliaceae)

*Blumea balsamifera* (Asteraceae)

*Calotropis procera* (Apocynaceae)

*Cinnamomum camphora* (Lauraceae)

*Cinnamomum tamala* (Lauraceae)

*Citrus aurantiifolia* (Rutaceae)

*Curcuma longa* (Zingiberaceae)

*Datura stramonium* (Solanaceae)

*Diospyros malabarica* (Ebenaceae)

*Ficus religiosa* (Moraceae)

*Gossypium barbadense* (Malvaceae)

*Gossypium hirsutum* (Malvaceae)

*Ipomoea aquatica* (Convolvulaceae)

*Justicia adhatoda* (Acanthaceae)

*Monochoria vaginalis* (Pontederiaceae)

*Nicandra physalodes* (Solanaceae)

*Ocimum americanum* (Lamiaceae)

*Piper longum* (Piperaceae)

*Scoparia dulcis* (Plantaginaceae)

*Solanum anguivi* (Solanaceae)

*Syzygium aromaticum* (Myrtaceae)

*Terminalia bellirica* (Combretaceae)

*Urena lobata* (Malvaceae)

*Zingiber officinale* (Zingiberaceae)

**Tooth disease**

*Barleria prionitis* (Acanthaceae)

*Eclipta prostrata* (Asteraceae)

*Justicia adhatoda* (Acanthaceae)

*Mimusops elengi* (Sapotaceae)

*Moringa oleifera* (Moringaceae)

*Terminalia citrina* (Combretaceae)

**Toxic, opium. *See also* Poison.**

*Celastrus paniculatus* (Celastraceae)

*Oroxylum indicum* (Bignoniaceae)

*Syzygium cumini* (Myrtaceae)

**Toxins, purge. *See also* Poison, Purgative.**

*Dregea volubilis* (Apocynaceae)

*Mesua ferrea* (Calophyllaceae)

**Tranquilizer**

*Rauvolfia serpentina* (Apocynaceae)

**Trembling**

*Rauvolfia serpentina* (Apocynaceae)

**Trim body**

*Amorphophallus paeoniifolius* (Araceae)

**Tuberculosis**

*Centella asiatica* (Apiaceae)

*Ixora chinensis* (Rubiaceae)

*Martynia annua* (Martyniaceae)

**Tumor**

*Aloe vera* (Asphodelaceae)

*Cardiospermum halicacabum* (Sapindaceae)

*Citrus limon* (Rutaceae)

*Myristica fragrans* (Myristicaceae)

*Sesbania grandiflora* (Fabaceae)

*Sesbania sesban* (Fabaceae)

*Sinapis alba* (Brassicaceae)

**Ulcers**

*Acacia catechu* (Fabaceae)

*Albizia odoratissima* (Fabaceae)

*Alstonia scholaris* (Apocynaceae)

*Artocarpus heterophyllus* (Moraceae)

*Bauhinia acuminata* (Fabaceae)

*Careya arborea* (Lecythidaceae)

*Cycas rumphii* (Cycadaceae)

*Ficus benjamina* (Moraceae)

*Gloriosa superba* (Colchicaceae)

*Gmelina arborea* (Lamiaceae)

*Heliotropium indicum* (Boraginaceae)

*Hydrolea zeylanica* (Hydroleaceae)

*Jasminum multiflorum* (Oleaceae)

*Linum usitatissimum* (Linaceae)

*Momordica charantia* (Cucurbitaceae)

*Stachytarpheta indica* (Verbenaceae)

*Streblus asper* (Moraceae)

-canker sores

*Aegle marmelos* (Rutaceae)

*Aristolochia indica* (Aristolochiaceae)

*Ficus semicordata* (Moraceae)

**Unconsciousness**

*Aristolochia indica* (Aristolochiaceae)

**Urinary concretions**

*Bridelia retusa* (Phyllanthaceae)

**Urinary system**

*Aloe vera* (Asphodelaceae)

*Amaranthus spinosus* (Amaranthaceae)

*Aquilaria malaccensis* (Thymelaeaceae)

*Butea monosperma* (Fabaceae)

*Carica papaya* (Caricaceae)

*Clerodendrum indicum* (Lamiaceae)

*Convolvulus arvensis* (Convolvulaceae)

*Foeniculum vulgare* (Apiaceae)

*Gloriosa superba* (Colchicaceae)

*Gossypium barbadense* (Malvaceae)

*Gossypium hirsutum* (Malvaceae)

*Ipomoea aquatica* (Convolvulaceae)

*Mentha arvensis* (Lamiaceae)

*Mimosa pudica* (Fabaceae)

*Phyllanthus emblica* (Phyllanthaceae)

*Piper nigrum* (Piperaceae)

*Plantago major* (Plantaginaceae)

*Senna alata* (Fabaceae)

*Tadehagi triquetrum* (Fabaceae)

*Tanacetum cinerariifolium* (Asteraceae)

-difficulty in passing urine

*Alysicarpus vaginalis* (Fabaceae)

*Citrus aurantiifolia* (Rutaceae)

*Holarrhena pubescens* (Apocynaceae)

*Senna sulfurea* (Fabaceae)

-excessive

*Abrus precatorius* (Fabaceae)

*Aegle marmelos* (Rutaceae)

*Cinnamomum verum* (Lauraceae)

*Moringa oleifera* (Moringaceae)

*Nyctanthes arbor-tristis* (Oleaceae)

*Strychnos potatorum* (Loganiaceae)

*Tamarindus indica* (Fabaceae)

-infection

*Allium cepa* (Amaryllidaceae)

*Alpinia galanga* (Zingiberaceae)

*Annona squamosa* (Annonaceae)

*Arundo donax* (Poaceae)

*Azadirachta indica* (Meliaceae)

*Carica papaya* (Caricaceae)

*Centella asiatica* (Apiaceae)

*Cinnamomum bejolghota* (Lauraceae)

*Coix lacryma-jobi* (Poaceae)

*Curcuma comosa* (Zingiberaceae)

*Curcuma longa* (Zingiberaceae)

*Foeniculum vulgare* (Apiaceae)

*Justicia adhatoda* (Acanthaceae)

*Mirabilis jalapa* (Nyctaginaceae)

*Phyllanthus emblica* (Phyllanthaceae)

*Urena lobata* (Malvaceae)

-loose stool

*Euphorbia hirta* (Euphorbiaceae)

-retention

*Acorus calamus* (Acoraceae)

*Allium cepa* (Amaryllidaceae)

*Alpinia galanga* (Zingiberaceae)

*Alpinia officinarum* (Zingiberaceae)

*Annona squamosa* (Annonaceae)

*Arundo donax* (Poaceae)

*Bryophyllum pinnatum* (Crassulaceae)

*Calotropis procera* (Apocynaceae)

*Canna indica* (Cannaceae)

*Carthamus tinctorius* (Asteraceae)

*Centella asiatica* (Apiaceae)

*Coix lacryma-jobi* (Poaceae)

*Crinum asiaticum* (Amaryllidaceae)

*Curcuma comosa* (Zingiberaceae)

*Cyperus scariosus* (Cyperaceae)

*Dregea volubilis* (Apocynaceae)

*Holarrhena pubescens* (Apocynaceae)

*Mimosa pudica* (Fabaceae)

*Plumeria rubra* (Apocynaceae)

*Zingiber montanum* (Zingiberaceae)

-too little urine

*Gloriosa superba* (Colchicaceae)

*Ocimum americanum* (Lamiaceae)

**Urinary tract disorder**

*Acacia pennata* (Fabaceae)

*Asparagus officinalis* (Asparagaceae)

*Basella alba* (Basellaceae)

*Butea monosperma* (Fabaceae)

*Canna indica* (Cannaceae)

*Cardiospermum halicacabum* (Sapindaceae)

*Cassia fistula* (Fabaceae)

*Elettaria cardamomum* (Zingiberaceae)

*Enydra fluctuans* (Asteraceae)

*Ipomoea alba* (Convolvulaceae)

*Ipomoea aquatica* (Convolvulaceae)

*Magnolia champaca* (Magnoliaceae)

*Mansonia gagei* (Malvaceae)

*Momordica charantia* (Cucurbitaceae)

*Mucuna pruriens* (Fabaceae)

*Ocimum americanum* (Lamiaceae)

*Orthosiphon aristatus* (Lamiaceae)

*Plantago major* (Plantaginaceae)

*Tamarindus indica* (Fabaceae)

*Tectona grandis* (Lamiaceae)

*Tinospora cordifolia* (Menispermaceae)

*Trichosanthes tricuspidata* (Cucurbitaceae)

**Uterus**

-discharge

*Euphorbia antiquorum* (Euphorbiaceae)

*Ficus religiosa* (Moraceae)

-infection

*Cardiospermum halicacabum* (Sapindaceae)

*Curcuma longa* (Zingiberaceae)

-leimyomas

*Rotheca serrata* (Lamiaceae)

**Vaginal discharge**

*Abrus precatorius* (Fabaceae)

*Alysicarpus vaginalis* (Fabaceae)

*Amaranthus spinosus* (Amaranthaceae)

*Diospyros malabarica* (Ebenaceae)

*Gossypium barbadense* (Malvaceae)

*Gossypium hirsutum* (Malvaceae)

*Mimusops elengi* (Sapotaceae)

*Plantago major* (Plantaginaceae)

*Solanum anguivi* (Solanaceae)

*Syzygium cumini* (Myrtaceae)

*Tectona grandis* (Lamiaceae)

**Vascular fuctioning. *See also* Circulatory system.**

*Annona squamosa* (Annonaceae)

**Venereal disease. *See also* Gonorrhea, Herpes, Syphilis.**

*Bryophyllum pinnatum* (Crassulaceae)

*Eclipta prostrata* (Asteraceae)

*Momordica charantia* (Cucurbitaceae)

*Mucuna pruriens* (Fabaceae)

*Plumbago indica* (Plumbaginaceae)

*Rauvolfia serpentina* (Apocynaceae)

*Rotheca incisa* (Lamiaceae)

*Senna alata* (Fabaceae)

*Smilax glabra* (Smilacaceae)

*Smilax guianensis* (Smilacaceae)

*Urena lobata* (Malvaceae)

*Volkameria inermis* (Lamiaceae)

-joint inflammation from

*Clerodendrum indicum* (Lamiaceae)

-sores

*Carica papaya* (Caricaceae)

*Ficus religiosa* (Moraceae)

*Plumeria rubra* (Apocynaceae)

*Syzygium cumini* (Myrtaceae)

-vaginal discharge due to,

*Amaranthus spinosus* (Amaranthaceae)

*Syzygium cumini* (Myrtaceae)

*Tamarindus indica* (Fabaceae)

**Vermifuge**

*Asclepias curassavica* (Apocynaceae)

*Calotropis gigantea* (Apocynaceae)

*Eurycoma longifolia* (Simaroubaceae)

*Passiflora quadrangularis* (Passifloraceae)

*Senna tora* (Fabaceae)

*Solanum anguivi* (Solanaceae)

*Syzygium aromaticum* (Myrtaceae)

**Vesicant**

*Cleome gynandra* (Cleomaceae)

**Virility**

*Acalypha indica* (Euphorbiaceae)

*Allium sativum* (Amaryllidaceae)

*Arundo donax* (Poaceae)

*Coix lacryma-jobi* (Poaceae)

*Cuscuta reflexa* (Convolvulaceae)

*Datura stramonium* (Solanaceae)

*Diospyros malabarica* (Ebenaceae)

*Dregea volubilis* (Apocynaceae)

*Gossypium barbadense* (Malvaceae)

*Gossypium hirsutum* (Malvaceae)

*Ipomoea aquatica* (Convolvulaceae)

*Mirabilis jalapa* (Nyctaginaceae)

*Piper betle* (Piperaceae)

*Urena lobata* (Malvaceae)

**Vitality**

*Acalypha indica* (Euphorbiaceae)

*Allium cepa* (Amaryllidaceae)

*Coptis teeta* (Ranunculaceae)

*Diospyros malabarica* (Ebenaceae)

*Dregea volubilis* (Apocynaceae)

*Eclipta prostrata* (Asteraceae)

*Ipomoea aquatica* (Convolvulaceae)

*Mucuna pruriens* (Fabaceae)

*Piper betle* (Piperaceae)

*Piper cubeba* (Piperaceae)

*Senna alata* (Fabaceae)

*Sesbania sesban* (Fabaceae)

*Vitex trifolia* (Lamiaceae)

**Vitiligo**

*Clitoria ternatea* (Fabaceae)

*Millingtonia hortensis* (Bignoniaceae)

*Terminalia bellirica* (Combretaceae)

**Vomit**

*Acacia concinna* (Fabaceae)

*Acalypha indica* (Euphorbiaceae)

*Amaranthus spinosus* (Amaranthaceae)

*Apium graveolens* (Apiaceae)

*Aquilaria malaccensis* (Thymelaeaceae)

*Arundo donax* (Poaceae)

*Basella alba* (Basellaceae)

*Cinnamomum tamala* (Lauraceae)

*Citrus aurantiifolia* (Rutaceae)

*Coix lacryma-jobi* (Poaceae)

*Coriandrum sativum* (Apiaceae)

*Curcuma longa* (Zingiberaceae)

*Cymbopogon citratus* (Poaceae)

*Euphorbia hirta* (Euphorbiaceae)

*Foeniculum vulgare* (Apiaceae)

*Hydnocarpus kurzii* (Achariaceae)

*Justicia adhatoda* (Acanthaceae)

*Ocimum americanum* (Lamiaceae)

*Rauvolfia serpentina* (Apocynaceae)

*Strychnos potatorum* (Loganiaceae)

*Syzygium aromaticum* (Myrtaceae)

*Tanacetum cinerariifolium* (Asteraceae)

*Terminalia citrina* (Combretaceae)

*Trachyspermum ammi* (Apiaceae)

-of blood

*Mimosa pudica* (Fabaceae)

*Sinapis alba* (Brassicaceae)

*Tinospora cordifolia* (Menispermaceae)

*Tradescantia spathacea* (Commelinaceae)

**Vulnerary. *See also* Wounds.**

*Celosia argentea* (Amaranthaceae)

**Wasting**

*Plumbago zeylanica* (Plumbaginaceae)

**Weakness**

*Alstonia scholaris* (Apocynaceae)

*Boerhavia diffusa* (Nyctaginaceae)

*Cardiospermum halicacabum* (Sapindaceae)

*Cinnamomum bejolghota* (Lauraceae)

*Vitex trifolia* (Lamiaceae)

-during menstruation

*Citrus limon* (Rutaceae)

**Weight gain**

*Alstonia scholaris* (Apocynaceae)

*Aquilaria malaccensis* (Thymelaeaceae)

*Catharanthus roseus* (Apocynaceae)

*Ficus religiosa* (Moraceae)

*Gossypium barbadense* (Malvaceae)

*Gossypium hirsutum* (Malvaceae)

*Mucuna pruriens* (Fabaceae)

*Nyctanthes arbor-tristis* (Oleaceae)

*Oroxylum indicum* (Bignoniaceae)

*Plumbago indica* (Plumbaginaceae)

*Senna alata* (Fabaceae)

**Weight loss**

*Urena lobata* (Malvaceae)

*Vitex trifolia* (Lamiaceae)

**Whooping cough**

*Boerhavia diffusa* (Nyctaginaceae)

*Croton tiglium* (Euphorbiaceae)

*Eucalyptus globulus* (Myrtaceae)

*Piper betle* (Piperaceae)

*Rotheca serrata* (Lamiaceae)

*Senna alata* (Fabaceae)

*Syzygium aromaticum* (Myrtaceae)

**Worms**

*Acorus calamus* (Acoraceae)

*Annona squamosa* (Annonaceae)

*Azadirachta indica* (Meliaceae)

*Clerodendrum indicum* (Lamiaceae)

*Coccinia grandis* (Crassulaceae)

*Combretum indicum* (Combretaceae)

*Eclipta prostrata* (Asteraceae)

*Holarrhena pubescens* (Apocynaceae)

*Momordica charantia* (Cucurbitaceae)

*Mucuna pruriens* (Fabaceae)

*Nyctanthes arbor-tristis* (Oleaceae)

*Piper longum* (Piperaceae)

*Plumbago zeylanica* (Plumbaginaceae)

*Tadehagi triquetrum* (Fabaceae)

*Zingiber montanum* (Zingiberaceae)

-guinea worm

*Leea macrophylla* (Vitaceae)

-intestinal worms

*Aegle marmelos* (Rutaceae)

-threadworms, roundworms

*Alstonia scholaris* (Apocynaceae)

*Gloriosa superba* (Colchicaceae)

*Moringa oleifera* (Moringaceae)

**Wounds**

*Allium sativum* (Amaryllidaceae)

*Asclepias curassavica* (Apocynaceae)

*Calophyllum inophyllum* (Calophyllaceae)

*Curcuma longa* (Zingiberaceae)

*Cycas rumphii* (Cycadaceae)

*Diospyros malabarica* (Ebenaceae)

*Eucalyptus globulus* (Myrtaceae)

*Ficus retusa* (Moraceae)

*Heliotropium indicum* (Boraginaceae)

*Mimusops elengi* (Sapotaceae)

*Zingiber montanum* (Zingiberaceae)

*Zingiber officinale* (Zingiberaceae)

**Yaws**

*Alstonia scholaris* (Apocynaceae)

**Exact purposes not given**

**Culinary purposes**:

*Trachyspermum roxburghianum* (Apiaceae)

**Medicinal values/purposes**:

*Acalypha wilkesiana* (Euphorbiaceae)

*Amherstia nobilis* (Fabaceae)

*Bougainvillea spectabilis* (Nyctaginaceae)

*Clerodendrum thomsoniae* (Lamiaceae)

*Delonix regia* (Fabaceae)

*Eucalyptus globulus* (Myrtaceae)

*Grewia asiatica* (Malvaceae) (root)

*Grewia hirsuta* (Malvaceae)

*Kopsia fruticosa* (Apocynaceae)

*Linostoma pauciflorum* (Thymelaeaceae)

*Spatholobus parviflorus* (Fabaceae)

*Terminalia catappa* (Combretaceae)

*Trachyspermum roxburghianum* (Apiaceae)

**Oral medications**:

*Ricinus communis* (Euphorbiaceae)
